# Identification of genomic diversity and selection signatures in Luxi cattle using whole-genome sequencing data

**DOI:** 10.5713/ab.23.0304

**Published:** 2024-01-20

**Authors:** Mingyue Hu, Lulu Shi, Wenfeng Yi, Feng Li, Shouqing Yan

**Affiliations:** 1 Department of Animal Science, Jilin University, Changchun 130062, China; 2 Shandong Binzhou Animal Science & Veterinary Medicine Academy, Binzhou, 256600, China

**Keywords:** Luxi Cattle, Genetic Diversity, Selection Signature, Whole-genome Sequencing

## Abstract

**Objective:**

The objective of this study was to investigate the genetic diversity, population structure and whole-genome selection signatures of Luxi cattle to reveal its genomic characteristics in terms of meat and carcass traits, skeletal muscle development, body size, and other traits.

**Methods:**

To further analyze the genomic characteristics of Luxi cattle, this study sequenced the whole-genome of 16 individuals from the core conservation farm in Shandong region, and collected 174 published genomes of cattle for conjoint analysis. Furthermore, three different statistics (*pi*, *F*_st_, and XP-EHH) were used to detect potential positive selection signatures related to selection in Luxi cattle. Moreover, gene ontology and Kyoto encyclopedia of genes and genomes pathway enrichment analyses were performed to reveal the potential biological function of candidate genes harbored in selected regions.

**Results:**

The results showed that Luxi cattle had high genomic diversity and low inbreeding levels. Using three complementary methods (*pi*, *F*_st_, and XP-EHH) to detect the signatures of selection in the Luxi cattle genome, there were 2,941, 2,221 and 1,304 potentially selected genes identified, respectively. Furthermore, there were 45 genes annotated in common overlapping genomic regions covered 0.723 Mb, including PLAG1 zinc finger (*PLAG1*), dedicator of cytokinesis 3 (*DOCK3*), ephrin A2 (*EFNA2*), DAZ associated protein 1 (*DAZAP1*), Ral GTPase activating protein catalytic subunit alpha 1 (*RALGAPA1*), mediator complex subunit 13 (*MED13*), and decaprenyl diphosphate synthase subunit 2 (*PDSS2*), most of which were enriched in pathways related to muscle growth and differentiation and immunity.

**Conclusion:**

In this study, we provided a series of genes associated with important economic traits were found in positive selection regions, and a scientific basis for the scientific conservation and genetic improvement of Luxi cattle.

## INTRODUCTION

Domestic cattle can not only provide meat, skin, hair, and other living materials, but also the main source of livestock power for farming and transportation, which gives them a pivotal role in world agricultural [1,2]. According to different morphological characteristics and living habits, domestic cattle can be divided into two subspecies, humpless taurine (*Bos taurus*) and humped indicine (*Bos indicus*) [3]. *Bos taurus* are mainly distributed in the whole Eurasian continent, North Africa, and West Africa, while *Bos indicus* are mainly distributed in the Indian subcontinent and Southeast Asia, but also in the Middle East and Southern Africa, and the Americas [4]. Among them, native breeds have higher genetic diversity compared with commercial breeds, which reflects in their ability to adapt the local conditions and used as an important genetic resource for cattle breeding and improvement [5]. However, with the rapid development of urbanization and other factors, the variety and quantity of indigenous breeds are significantly reduced, and the risk of their disappearance is increasing. Therefore, it is of significance to determine the genetic diversity characteristics and optimize the managements of indigenous breeds.

For the last decades, molecular markers have been widely used in animal breeding programs and several different types of markers are available [6]. However, their application has been limited in the past due to low density, high labor intensity, high technical requirements, and high cost of large-scale analysis. The advent of Whole-genome sequencing (WGS) technology has led to an exponential increase in the number of genetic variants found in a single experiment. Especially, in recent years, with the development of sequencing technology and the reduction of sequencing cost, WGS technology has greatly promoted the mining of high-throughput genetic variants which have been applied to identify the genetic diversity, population structure, candidate genes for major economic traits, and the screening of molecular markers [7]. At present, a wide range of systematic WGS studies especially in the indigenous breeds have been carried out in pigs [8], cattle [9], sheep [10], and other livestock, and many remarkable scientific achievements have been achieved.

Luxi cattle (LUX), as one of the important indigenous breeds mainly distributed in Shandong province of China, are an important genetic resource noted for its large body size, fine-quality meat, and good strength for draft [11]. Over the past 30 years, LUX has been intensively bred for beef, leading to genetic improvement in production traits. In 2022, the results of Ge et al [12] showed the values of average carcass weight and slaughter rate in LUX were much higher than those in the past, and Luxi beef was rich in amino acids and had a high content of essential amino acids. Genetic diversity is an important component of biological diversity and a key factor for species adaptation to the varied environments or artificial selection [13]. Changes in genomic structural features caused by artificial or natural selections are called selection signals. These signals are closely related to the breeding direction of animals and the adaptation mechanism of domestication. Therefore, the detection of selection signals is of great significance for the genetic improvement of livestock and poultry, which helps to mine the genes related to the economic traits of animals, understand the potential genetic bases of the formation of traits, and analyze the phenotypic differences among populations or breeds. Up to date, however, the genomic diversity and the genetic basis of prominent characteristics of LUX are still unknown.

In this study, at the whole-genome level, single nucleotide polymorphisms (SNPs) were identified and annotated in LUX based on sequencing data of a total of 190 individuals and searched for selection signatures and candidate genes in LUX by three methods (*pi*, *F*_st_, and XP-EHH) were analyzed. These results will help us to understand the process of breed formation and provide a theoretical basis for understanding the genetic mechanism of LUX breed-specific characteristics.

## MATERIALS AND METHODS

### Animal care

All experiments were conducted in accordance with the Institutional Animal Care and Use Committee guidelines (IACUC No. SY202306004) under currently approved protocols at Jilin University.

### Sample collection, DNA extraction and whole-genome sequencing

Sixteen unrelated adult LUX individuals were collected according to the pedigree information from Hongxiang Animal Husbandry Co., Ltd in Juancheng County, Heze City, Shandong Province of China. The genomic DNA was extracted with TIANamp blood DNA kit from TIANGEN Biotech (Beijing, China) Co., Ltd. After extraction, the 1.0% agarose gel was used to detect the quality of genomic DNA fragments, and the Nanodrop ND-2000 was used to detect the purity of DNA samples (OD260/OD280 = 1.8–2.0). A paired-end library was constructed for each individual and the DNA was subjected to a 2×150 bp model using DNBSEQ-T7 platform at the Novogene Bioinformatics, Beijing, China. Furthermore, the genomic data of 174 individuals from 15 breeds available in the SRA database were downloaded, including Luxi (LUX, n = 6), Angus (ANG, n = 20), Shorthorn (SHO, n = 17), Mishima (MIS, n = 8), Kazakh (KAZ, n = 10), Mongolian (MON, n = 13), Hanwoo (HAN, n = 20), Tibetan (TIB, n = 11), Chaidamu (CHA, n = 8), Lingnan (LIN, n = 8), Wenling (WEN, n = 11), Wenshan (WES, n = 7), Xiangxi (XIA, n = 14), Zhoushan (ZHO, n = 6), and Brahman (BRA, n = 15). In total, the data from 190 individuals were used for the subsequent analysis (Supplementary Table S1).

### Reads mapping, single nucleotide polymorphism calling and annotation

Clean reads from all 190 cattle were mapped against the *Bos taurus* reference genome (ARS-UCD1.2) using the BWA-MEM (v0.7.13) with ‘bwa mem’ parameters, which resulted in removing low-quality alignments and discarded low-quality sites [14]. The Picard tool was used to mark potential duplicate reads (REMOVE_DUPLICATES). Variant calling was performed by Genome Analysis Toolkit (GATK v4.1.4) and the module of “HaplotypeCaller”, “GenotypeGVCFs” and “SelectVariants” was performed for downstream SNP calling [15]. All autosomal biallelic SNPs were filtered using the module “Variant Filtration” of GATK to obtain high-quality SNPs based on the following criteria: ‘QD<2.0 || FS>60.0 || MQ<40.0 || SOR >3.0 || MQRankSum <–12.5 || ReadPosRankSum <–8.0’. Finally, a total of 78,727,342 autosomal biallelic SNPs in 190 samples were identified in the genome through GATK’s official website recommendation process.

In addition, the distribution and functional annotation of the autosomal SNPs of LUX were carried out using SnpEff (v5.1) based on the reference assembly (ARS-UCD1.2), and the deleterious mutations were identified in our population based on the sorting intolerant from tolerant (SIFT v5.2.2) scores using the Variant Effect Predictor tool (VEP v.91.3) with default parameter settings. The mutations with SIFT less than 0.05 were adjudged as deleterious to protein function and then mapped to their location in the protein-coding genes.

### Genetic diversity analysis and linkage disequilibrium

Genetic diversity was assessed based on the SNPs filtered using PLINK software (v1.9) with parameters ‘--geno 0.05 --maf 0.03 --mind 0.1’. The expected heterozygosity (*H*_E_) and observed heterozygosity (*H*_O_) were estimated using the ‘--hardy’ option, and minor allele frequency (MAF) was estimated using the ‘--freq’ option implemented in the PLINK (v1.9). Further, the nucleotide diversity (*pi*) was estimated using VCFtools with the parameters ‘--window-pi 50,000 --window-pi-step 25,000’. By using the PLINK (v1.9), the homozygosity inbreeding coefficient (*F*_HOM_) was calculated using the ‘--het’ option, and the runs of homozygosity (ROHs) were measured using the ‘--homozyg’ option [16]. The runs of homozygosity-based inbreeding coefficient (*F*_ROH_) were calculated for each animal as *F*_ROH_ = ∑L_ROH_/L_GENOME_, where ∑L_ROH_ is the length of all ROH longer and L_GENOME_ is the length of the genome covered by SNPs, which is 2,489,370,000 bp in our data [17]. The degree of linkage disequilibrium decay (LD) for each breed was measured by taking the correlation coefficients (r^2^) of pairwise SNPs using PopLDdecay with default parameters.

### Population structure and phylogenetic analysis

After pruning in PLINK (v1.9) with the parameter ‘--indep-pair-wise 50 25 0.2’, a set of SNPs in high-level pair-wise LD were removed to reduce the SNP dependency [16]. This option removed one of each pair of SNPs with a pairwise r^2^>0.2 within a window of 50 SNPs and shifted the window by a step size of 25 SNPs, and the remaining 2,618,449 SNPs in approximate LD were extracted from the subset of 30,041,713 SNPs and used for population structure analysis. Principal component analysis (PCA) was performed by the GCTA (v1.92.3) with an option ‘--grm’ to discern genetic relationships among breeds [18]. The graphical representation of PCA was depicted using the plot function in R (v3.6.1). In addition, population structure analysis was carried out with genetic clusters *K* ranging from 2 to 7 using the ADMIXTURE (v1.3) software with the parameters ‘admixture --cv’ to estimate ancestral populations among our dataset, and the graphical representation of the ADMIXTURE results was generated using R (v3.6.1) [19]. Based on the pairwise genetic distance matrix using PLINK (v.1.9), the model of neighbor-joining (NJ) tree used for phylogenetic reconstruction was constructed using MEGA (v7.0), and was visualized using iTOL [20].

### Analysis and identification of selective sweeps

To detect potential genomic regions related to selection in LUX, three different statistics were used in this study. The nucleotide diversity (*pi*) and the fixation index (*F*_st_) were estimated based on a sliding window with 50 kb and 25 kb step using VCFtools [21], and the parameters are ‘--window-pi 500,000 --window-pi-step 25,000’ and ‘--fst-window-size 50,000 --fst-window-step 25,000’, respectively. Furthermore, haplotype phasing was performed using BEAGLE (v5.2), and the cross-population extended haplotype homozygosity (XP-EHH) using selscan (v1.1) was also performed to detect the positive selection signatures between LUX and Angus [22]. The average of standardized XP-EHH scores for each 50 kb region were chosen for XP-EHH selection scans. Then, the windows of the top 5% score of the three methods are selected for the next analysis. To make the results more reliable, the overlapping of genomic regions identified by at least two methods was determined as potential candidate regions of selection using BEDTools (v2.30.0) with the parameters ‘intersect’ and was annotated based on ARS-UCD1.2 by BioMart tool (http://asia.ensembl.org/index.html) [10]. To reveal the potential biological function of candidate genes harbored in selected regions, gene ontology (GO) and Kyoto encyclopedia of genes and genomes (KEGG) pathway enrichment analyses were performed using the web-based platform KEGG Orthology Based Annotation System (KOBAS v3.0) [23]. The enriched GO terms and pathways were selected stringently with an adjusted probability (p<0.05) and were used for further analysis in this study. Moreover, the quantitative trait locus (QTL) was downloaded from the Cattle QTLdb and used to identify the overlapping regions associated with the most plausible trait-associated selective signatures [24]. The number and function of genes in candidate regions were determined after annotation.

## RESULTS

### Sequencing and SNPs calling

A total of 4,055,161,272 clean reads were generated after genome sequencing in 16 LUX samples and the detailed information on sequencing data was shown in Supplementary Table S2. The clean reads were aligned to the reference genome (ARS-UCD1.2) with an average depth of 13.51×. Further, we annotated 32,462,568 biallelic SNPs that were discovered in LUX, and the average genome-wide nucleotide diversity within the LUX was 13.04 SNPs/kb. The frequency of SNPs on each chromosome was different, and those on chromosome 27 were the highest (15.03 SNPs/kb). Among these SNPs, functional annotation of the polymorphic sites revealed that the vast majority of SNPs existed in the intergenic region (21.537%) and intronic region (66.158%) (Supplementary Table S3). Furthermore, 2,151 stop gain, 298 stop lost, 381 start lost, 1,401 splice donor and 1,075 splice acceptor variants were detected. According to the SIFT scoring, 30,203 missense SNPs were classified as likely deleterious to protein function (SIFT score <0.05) which will highlight the genetic variants having a potential influence on the function of the protein encoded.

### Genetic diversity analysis and linkage disequilibrium

**Figure 1:**
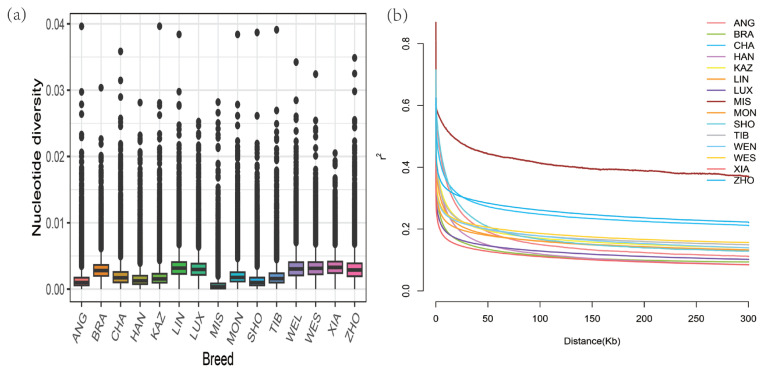
Genetic diversity among 190 individuals from 15 populations. (a) Box plots of the nucleotide diversity for each group. The black line in the boxplot is the median line and the outside points are outliers. (b) Decay of linkage disequilibrium on cattle autosomes was estimated from each breed. Different colored lines represented different breeds. ANG, Angus; BRA, Brahman; CHA, Chaidamu; HAN, Hanwoo; KAZ, Kazakh; LIN, Lingnan; LUX, Luxi; MIS, Mishima_Ushi; MON, Mongolian; SHO, Shorthorn; TIB, Tibetan; WEN, Wenling; WES, Wenshan; XIA, Xiangxi; ZHO, Zhoushan.

In total, 30,041,713 common autosomal biallelic SNPs were obtained in all breeds after filtering. To elucidate the genomic characteristics, the nucleotide diversity (*pi*) was calculated, and the result indicated that the pi values ranged from 0.00063 to 0.00333 (Supplementary Table S4). Among them, breeds belonging to indicine (followed by XIA, LIN, WES, and WEN) had the highest nucleotide diversity, and the value *pi* of LUX cattle was higher than that in most taurine breeds but lower than that indicine breeds (Figure 1a). Among all groups in this study, the *H*_O_ of LUX (0.2556) was higher than all breeds, and the *H*_E_ of LUX (0.2482) was between BRA (0.2316) and WEN (0.2494), with relatively higher heterozygosity. Overall, these results indicated high genetic diversity in LUX. It can be observed from the figure that the LD pattern of each variety is different in subpopulations, and the LD level of indicine breeds was lower than that of taurine breeds which is consistent with previous studies (Figure 1b). The LD attenuation distance of BRA population is smaller than most breeds, which may be implied the highest genetic diversity. At distances between markers (>50 kb), XIA and BRA had the lowest LD level, followed by LUX. Additionally, two inbreeding coefficients were calculated based on the genomic data of all tested individuals. As shown in Supplementary Table S4, the results showed that the *F*_HOM_ (−0.0298) in LUX was very close to zero. In addition, LUX has the lowest *F*_ROH_ (0.1765) compared to all other breeds. This result indicated the existing breeding programs effectively avoid inbreeding.

### Population structure and admixture analysis

**Figure 2:**
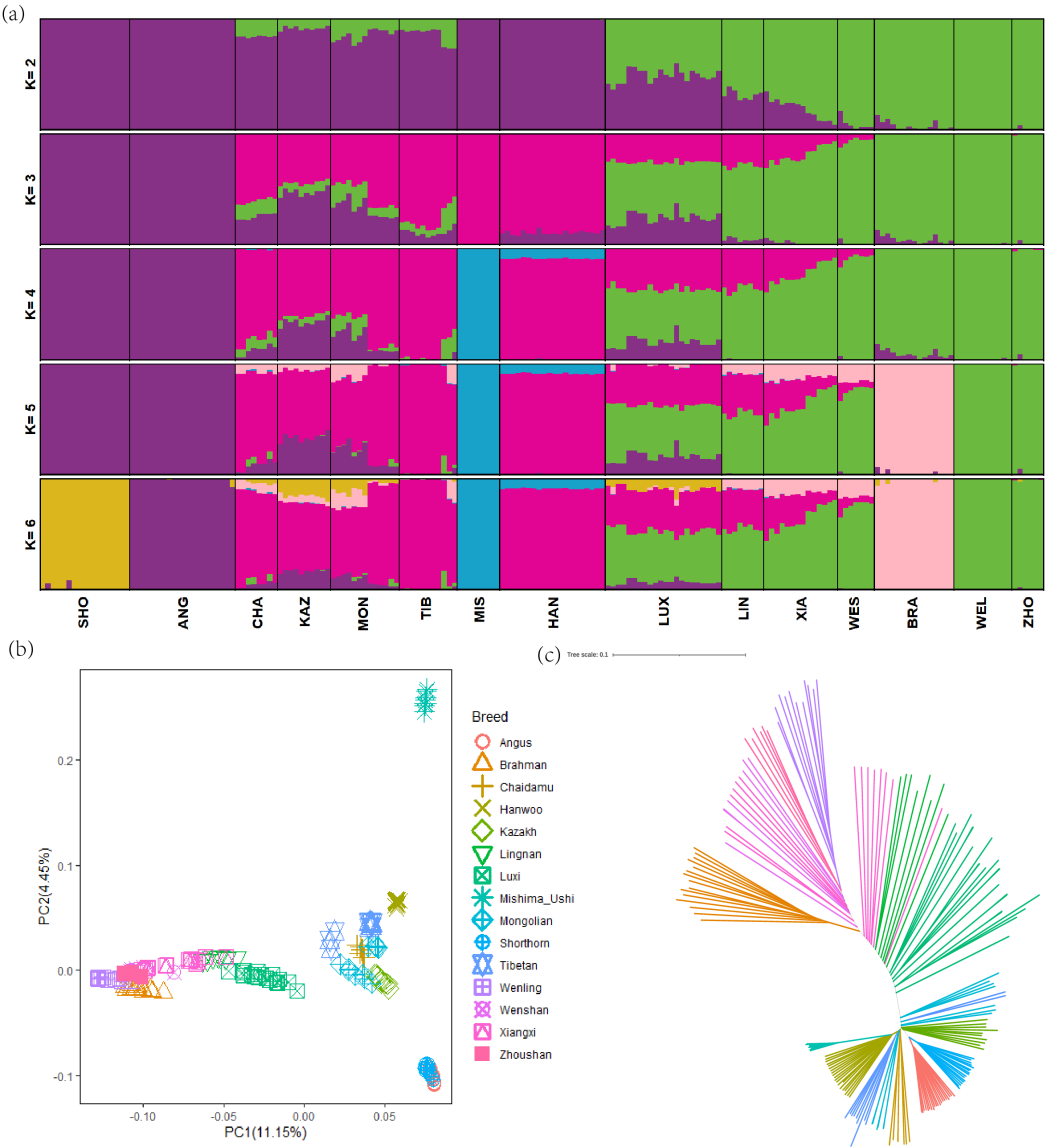
Population structure of Luxi cattle and its relationship compared with other breeds. (a) Model-based clustering among different breeds using ADMIXTURE (*K* = 2 to 6). (b) the results of principal component analysis. (c) Neighbor-joining tree of the relationships among breeds (190 individuals in total).

To investigate the genetic relationships among LUX and other cattle breeds, a total of 2,618,449 autosomal SNPs which generated from the pruning of pair-wise LD for further analysis. The analysis was run with *K* ranging from 2 to 7 to reflect the genetic background of the 15 breeds, with the lowest cross-validation error value (0.3959) being 6 (Figure 2a). There was a clear pattern of geographical distribution among these cattle breeds, which is consistent with the previous studies [25]. When *K* = 2, LUX showed mixed ancestral component: *Bos taurus* and *Bos indicus* ancestry; and when *K* = 3, LUX showed genetic heterogeneity with shared genome ancestry with East Asian taurine (HAN and MIS), European taurine (SHO and ANG) and Chinese indicine (WEL and ZHO) genetic background (Figure 2a). Furthermore, the genetic relationships among the 15 cattle breeds revealed using PCA are shown in Figure 2. In PCA analysis, PC1 divided these breeds into *Bos taurus* and *Bos indicus* and can explain 11.15% of the total variations (Figure 2b). In particular, PC1 obviously separated LUX from other breeds, which had the greatest explanatory power. The NJ tree indicated that LUX was clustered together with the LIN which was consistent with PCA and ADMIXTURE results (Figure 2c).

### Genome-wide scanning for selection signatures

**Figure 3:**
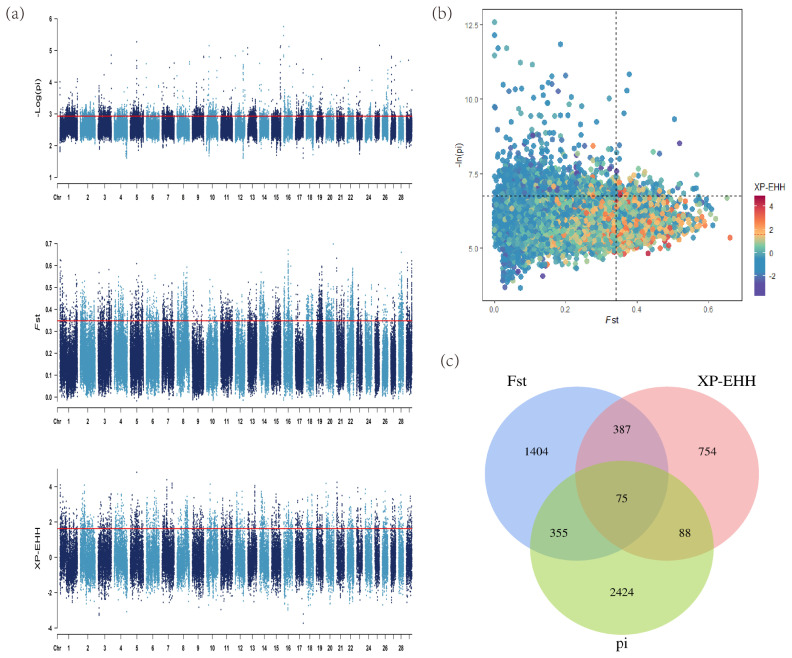
Analysis of the signatures of positive selection in the genome of Luxi cattle. Red line displays the threshold levels of 5%. (a) Manhattan plot of selective sweeps in Luxi cattle. Alternating colors distinguish markers on neighboring chromosomes. (b) Conjoint analysis of the signatures of *pi*, *F*_st_, and XP-EHH of Luxi cattle. (c) Venn diagram showing the genes overlap among pi, Fst, and XP-EHH significant selection region.

In the selection signal analyses, *pi*, *F*_st_ and XP-EHH methods (top 5%) were performed to identify the candidate regions and genes. There were 6,017 regions containing 2,941 genes, 4,804 regions containing 2,221 genes and 1,920 regions containing 1,304 genes identified, respectively. The detailed information including the particular candidate regions under selection were identified by different methods and shown in Figure 3a and Supplementary Table S5–S7. Conjoint analysis of the signatures of *pi*, *F*_st_, and XP-EHH are shown in Figure 3b. Of these, 75 overlapped genes were detected by all three methods (Figure 3c). Furthermore, among the candidate genes identified by at least two methods, several genes with overlapped regions were strongly selected in LUX and related to muscle development (dedicator of cytokinesis 3 [*DOCK3*]) [26], production traits (Ral GTPase activating protein catalytic subunit alpha 1 [*RALGAPA1*] and DAZ associated protein 1 [*DAZAP1*]) [27,28], body length (PLAG1 zinc finger [*PLAG1*]) [29], marbling-related (myosin binding protein C1 [*MYBPC1*]) [30] (Table 1).

After integration, 45 common regions within 45 candidate genes were detected in all three methods, indicating that these genes can be considered as potential candidates for positive selection in LUX (Supplementary Table S8). These genes were further enriched in KEGG pathways and GO terms to gain a better understanding of their functions and signaling pathways (Supplementary Table S9). In KEGG analysis, 14 significant enriched pathways were obtained, including the oxidative phosphorylation pathway (bta00190; p = 0.00109), the adipocytokine signaling pathway (bta04920; p = 0.00495), and the ubiquitin mediated proteolysis pathway (bta04120; p = 0.01726), which might be related to immunity and fat metabolism in LUX. Furthermore, a total of 169 significantly enriched GO terms with p-value <0.05 were observed, such as regulation of lipid metabolic process (GO:0019216; p = 0.03343), embryonic skeletal system development (GO:0048706; p = 0.04025), and bone remodeling (GO:0046849; p = 0.02102), which are related to metabolism and protein synthesis and skeletal muscle development.

Moreover, the QTL database was used to identify which genetic markers of candidate regions are most correlated with quantitative traits. The results showed that a significant region might overlap with several QTLs associated with different traits, 271 QTLs were located within or overlapping with these 45 candidate regions (Supplementary Table S10). Simultaneously, most of the candidate regions detected in the present study contain several QTLs for economically important traits, such as carcass traits, milk traits, and body weight traits. Among 45 candidate regions, 91.11% (41 candidate regions) were related to 65 QTLs for production traits, and 95.56% (43 candidate regions) were related to 61 meat and carcass traits suggesting the strong selection for growth and meat performance traits of LUX. Notably, 88.89% (40 candidate regions) were associated with 59 QTLs for milk traits, suggesting the potential for selection of milk traits during the breeding of LUX.

## DISCUSSION

Initially, LUX was a dual purpose cattle breed that was used as a draft animal and for beef production and an important part of a farm’s assets [31]. Based on the whole-genome sequencing data, this study conducted an overall evaluation of the genetic diversity and population structure of LUX, which is helpful to accurately understand the genetic diversity and breeding situation of current LUX. Compared with microsatellite markers, genome-wide SNP markers can more objectively reflect genetic differences between individuals and are increasingly used to analyze the genetic diversity of populations [32]. Genetic diversity is a key factor for population survival and evolution, and based on the analysis of the whole genome sequencing results, we can develop a rational breeding program to reduce inbreeding and maintain the genetic diversity of LUX [33]. In this study, the *H*_O_ and *H*_E_ of LUX were 0.2556 and 0.2482 respectively, and it was found that *H*_O_ was greater than *H*_E_ which indicates that the genetic characteristics of LUX may have high genetic diversity among the 15 cattle populations. In population structure analysis, the ADMIXTURE analysis showed that LUX was located between taurine cattle and indicine cattle, which was also consistent with the situation of hybrid breeds in central China, such as Bohai-black, Qinchuan and Jiaxian cattle while the ratio of ancestral component is different [34-36]. Furthermore, the NJ tree showed LUX were separated from other cattle breeds, and the PCA result provides similar results to the above mentioned. Based on the study of autosomal SNPs, results showed that LUX have the second highest genetic diversity, probably due to being a cross between the *Bos taurus* and *Bos indicus*. In addition, different populations of the same species have large differences in LD decay rates due to different genetic backgrounds, and domestication selection will lead to a decrease in population genetic diversity and an increase in loci linkage. Interestingly, LUX had the lowest LD levels than other commercial varieties and higher than that of XIA and HAN cattle, which may be due to the lower selection intensity experienced by the LUX herd and there are still many breeding strategies to choose from in the future.

With the improvement of whole-genome sequencing technology, the biological basic research on the formation of important traits and the demand for improving the efficiency of livestock breeding are gradually increasing [37]. The “1,000 Bull Genome” project has greatly met people’s demand for functional gene mining of important economic traits and provided a theoretical basis and technical support for the development of efficient cattle breeding technology [38]. However, the current sequencing objects are mainly commercial varieties, and limited attention is paid to the genomic diversity and whole-genome scanning footprint of positive selection characteristics of indigenous varieties [39]. This study lays a foundation for the study of genetic diversity and genetic characteristics of other indigenous cattle in China in the future.

To improve detection efficiency and reduce false positives, we used *pi*, *F*_st_ and XP-EHH methods to identify plausible positive selection regions associated with important economic traits in LUX, and if a gene is clearly detected by at least two methods, then it will be used as a candidate gene for further analysis. When comparing the selection signatures of LUX with ANG cattle, 45 genes were identified by all three methods, indicating that these genes are strongly selected in LUX. Among them, a series of genes in LUX with strong signals for regulating skeletal muscle regeneration to varying degrees (*DOCK3* and mediator complex subunit 13 [*MED13*]) have also been reported [26,40]. Furthermore, we also identified several genes related to production and growth traits (*RALGAPA1* and *DAZAP1*) [27,28]. *INSL6* is associated with mammalian reproduction, nutrient metabolism and immune response [41]. *PLAG1* in candidate regions is associated with the body weight/height in animals including cattle, pigs, horses, and so on [42–44]. In addition, most of the annotated genes among genomic regions are uncharacterized symbols, and their biological importance remains to be elucidated. Enrichment analysis is used for identifying the likelihood of biological processes which understand the function and complex pathways of candidate genes highly related to the biological phenomena under study. In this study, GO terms (e.g., regulation of lipid metabolic process, embryonic skeletal system development, and bone remodeling) were enriched, involving metabolism and protein synthesis and skeletal muscle development related genes, such as ephrin A2 (*EFNA2*) and golgi membrane protein 1 (*GOLM1*) [45]. Most of KEGG analysis are concentrated on immunity and fat metabolism pathways, further study of LUX is needed to understand the role of genetic variants resistant to disease and strong reproductive performance traits.

Due to the change of breeding direction from draft-beef dual-purpose to beef cattle in LUX, some measures focusing on improving its defects such as late body maturity and low daily gain have been taken by crossing with some commercial breed such as Simmental. For purebred LUX, we should further strengthen the management of breeding areas, take effective measures to prevent the number of purebred cattle from decreasing, and avoid blind hybridization.

## CONCLUSION

In conclusion, this study not only provides novel insights into LUX genomic diversity, phylogenetic relationship, and selection sweep, but also provides theoretical bases regarding the genetic mechanism underlying LUX characteristics and molecular breeding strategies of the composite cattle clades in the future. In addition, a series of candidate genes were identified that may be important for body size, skeletal muscle development traits, and production of meat and milk traits of this breed. These results will give an extensive reference for other important indigenous beef cattle and contribute to understanding the genetic mechanism behind artificial selection in the future.

## Figures and Tables

**Table 1:** Potential selected genes associated with important economic traits in Luxi cattle

Chr	Candidate genes	Methods	Traits
22	Dedicator of cytokinesis 3, *DOCK3*	*pi*, *F*_st_, XP-EHH	Muscle development
14	PLAG1 zinc finger, *PLAG1*	*pi*, *F*_st_, XP-EHH	Body length
21	Ral GTPase activating protein cat-alytic subunit alpha 1, *RALGAPA1*	*pi*, *F*_st_, XP-EHH	Growth
8	Insulin like 6, *INSL6*	*pi*, *F*_st_, XP-EHH	Reproduction
12	Caudal type Homeobox 2, *CDX2*	*pi*, *F*_st_	Embryo development
20	Growth hormone receptor, *GHR*	*F*_st_, XP-EHH	Growth and carcass
18	Cyclin dependent kinase 10, *CDK10*	*pi*, *F*_st_	Immune system
5	Myosin binding protein C1, *MYBPC1*	*pi*, *F*_st_, XP-EHH	Marbling-related
23	KH RNA binding domain con-taining, signal transduction asso-ciated 2, *KHDRBS2*	*pi*, XP-EHH	Reproduction

## SUPPLEMENTARY MATERIAL

**Supplementary Table S1**. Summary of 174 cattle sample information

**Supplementary Table S2**. Summary of sequencing data

**Supplementary Table S3**. Distribution of SNPs identified in LUX annotated by SnpEff

**Supplementary Table S4**. Genetic diversity of the 15 cattle breeds used in this study

**Supplementary Table S5**. A summary of genes from pi in LUX

**Supplementary Table S6**. A summary of genes from Fst in LUX

**Supplementary Table S7**. A summary of genes from XP-EHH in LUX

**Supplementary Table S8**. A summary of genes overlapped by pi, Fst and XP-EHH methods in common regions

**Supplementary Table S9**. GO and KEGG enrichment analysis of LUX candidate genes by three methods

**Supplementary Table S10**. QTLs overlapped with candidate selected regions
