# Supplementary material for: Identification of genomic diversity and selection signatures in Luxi cattle using whole-genome sequencing data
Source: Anim Biosci. 2024 Jan 20;37(3):461–70. doi: 10.5713/ab.23.0304 (PMC10915192; doi:10.5713/ab.23.0304)
Supplement: Supplementary file 1 [file ab-23-0304-Supplementary-Table-S1.pdf]

Supplementary Table S1. Summary of 174 cattle sample information

Sample ID	BioProject	Biosample	Breed	Total reads	Mapping reads	Mapping rate(%)	Mean depth(X)
SRR1346376	PRJNA176557	SAMN02841106	Angus	395875946	391561718	0.9891	14.1109
SRR1348580	PRJNA176557	SAMN02842718	Angus	372181591	366949587	0.9859	13.2785
SRR1355237	PRJNA176557	SAMN02843061	Angus	428909861	423375265	0.9871	15.3859
SRR1355244	PRJNA176557	SAMN02843079	Angus	377910910	375172448	0.9928	13.2139
SRR1355255	PRJNA176557	SAMN02843054	Angus	364202030	358981695	0.9857	13.0172
SRR1365132	PRJNA176557	SAMN02843101	Angus	406552664	399474057	0.9826	13.8106
SRR1365144	PRJNA176557	SAMN02843103	Angus	514057821	507517745	0.9873	18.1356
SRR1425124	PRJNA176557	SAMN02843152	Angus	528060557	521712423	0.988	18.7112
SRR8587811	PRJNA343262	SAMN10940450	Angus	538360187	530116926	0.9847	26.0498
SRR8587919	PRJNA343262	SAMN10940535	Angus	536067382	527009445	0.9831	25.9978
SRR8587920	PRJNA343262	SAMN10940537	Angus	631558461	617722630	0.9781	30.2106
SRR8587922	PRJNA343262	SAMN10940536	Angus	525246602	517789823	0.9858	25.6285
SRR8587923	PRJNA343262	SAMN10940538	Angus	649770832	628450702	0.9672	30.7457
SRR8587924	PRJNA343262	SAMN10940532	Angus	538210984	530233068	0.9852	26.0952
SRR8587992	PRJNA343262	SAMN10940498	Angus	683686107	673336510	0.9849	32.4308
SRR8588014	PRJNA343262	SAMN10940528	Angus	524270846	516002043	0.9842	24.9798
SRR8588042	PRJNA343262	SAMN10940457	Angus	521999445	513449007	0.9836	24.8335
SRR8588100	PRJNA343262	SAMN10940474	Angus	534604801	525575173	0.9831	25.7992
SRR8588211	PRJNA343262	SAMN10940508	Angus	547784305	540258588	0.9863	26.7315
SRR8588263	PRJNA343262	SAMN10940525	Angus	525588655	517024645	0.9837	25.5302
SRR10800466	PRJNA597241	SAMN13671047	Chaidamu	894043787	884751774	0.9896	37.2431
SRR10800467	PRJNA597241	SAMN13671046	Chaidamu	837138476	826036683	0.9867	35.6819
SRR10800468	PRJNA597241	SAMN13671045	Chaidamu	867272103	859428210	0.991	36.3879
SRR5507268	PRJNA379859	SAMN06698978	Chaidamu	217583818	216425236	0.9947	10.9403
SRR5507269	PRJNA379859	SAMN06698977	Chaidamu	276563866	274976672	0.9943	13.725
SRR5507270	PRJNA379859	SAMN06698976	Chaidamu	240952291	238660370	0.9905	11.8091
SRR5507271	PRJNA379859	SAMN06698975	Chaidamu	208783436	207332692	0.9931	10.409
SRR5507272	PRJNA379859	SAMN06698974	Chaidamu	253050556	251328006	0.9932	12.5389
SRR926762	PRJNA210523	SAMN02225723	Hanwoo	467084399	464429007	0.9943	11.8107
SRR934395	PRJNA210523	SAMN02225724	Hanwoo	435780749	433444025	0.9946	10.4954
SRR934396	PRJNA210523	SAMN02225725	Hanwoo	469840142	467183558	0.9943	12.6553
SRR934397	PRJNA210523	SAMN02225726	Hanwoo	375025606	373120908	0.9949	9.09755
SRR934398	PRJNA210523	SAMN02225727	Hanwoo	427453340	424502969	0.9931	11.0113
SRR934399	PRJNA210523	SAMN02225728	Hanwoo	474742086	472169736	0.9946	11.8638
SRR934400	PRJNA210523	SAMN02225729	Hanwoo	436556925	434133315	0.9944	13.7851
SRR934401	PRJNA210523	SAMN02225732	Hanwoo	425675993	423260609	0.9943	15.0463
SRR934402	PRJNA210523	SAMN02225733	Hanwoo	493594977	488780948	0.9902	12.4987
SRR934403	PRJNA210523	SAMN02225730	Hanwoo	408008000	405477775	0.9938	11.197
SRR934404	PRJNA210523	SAMN02225731	Hanwoo	439415243	437067687	0.9947	13.0677
SRR934415	PRJNA210519	SAMN02225744	Hanwoo	410096280	406416389	0.991	14.7427
SRR934416	PRJNA210519	SAMN02225745	Hanwoo	437502857	433467598	0.9908	15.6693
SRR934417	PRJNA210519	SAMN02225746	Hanwoo	407082711	403162700	0.9904	14.5942
SRR934418	PRJNA210519	SAMN02225747	Hanwoo	430167607	425593525	0.9894	15.4027
SRR934419	PRJNA210519	SAMN02225748	Hanwoo	394936167	391093222	0.9903	14.2426
SRR934432	PRJNA210519	SAMN02225750	Hanwoo	428387065	424330027	0.9905	15.376
SRR934433	PRJNA210519	SAMN02225751	Hanwoo	386207169	379841591	0.9835	13.7911
SRR934434	PRJNA210519	SAMN02225752	Hanwoo	394568542	391009637	0.991	14.1337
SRR934435	PRJNA210519	SAMN02225753	Hanwoo	354612968	351291492	0.9906	12.7532
SRR5507252	PRJNA379859	SAMN06698994	Kazakh	211990019	210515246	0.993	10.6456

SRR5507253	PRJNA379859	SAMN06698993	Kazakh	242428972	241036539	0.9943	12.2418
SRR5507254	PRJNA379859	SAMN06698992	Kazakh	211284901	210211630	0.9949	10.8124
SRR5507255	PRJNA379859	SAMN06698991	Kazakh	222815730	221313983	0.9933	11.2327
SRR5507257	PRJNA379859	SAMN06698989	Kazakh	239591952	238180156	0.9941	12.1755
SRR5507258	PRJNA379859	SAMN06698988	Kazakh	272749720	271229917	0.9944	13.1077
SRR5507259	PRJNA379859	SAMN06698987	Kazakh	255056634	253850575	0.9953	12.4932
SRR5507260	PRJNA379859	SAMN06698986	Kazakh	274979877	273691065	0.9953	13.4489
SRR6234772	PRJNA396672	SAMN07431003	Kazakh	382990230	380860266	0.9944	12.6842
SRR6234773	PRJNA396672	SAMN07431002	Kazakh	356124728	354122655	0.9944	11.8923
SAMD00013611	PRJDB2660	SAMD00013611	Mishima_Ushi	685104468	591002743	0.8626	19.3947
SAMD00013608	PRJDB2660	SAMD00013608	Mishima_Ushi	644753314	532638284	0.8261	16.8402
SAMD00013613	PRJDB2660	SAMD00013613	Mishima_Ushi	594518201	511358969	0.8601	16.5398
SAMD00013610	PRJDB2660	SAMD00013610	Mishima_Ushi	392185147	368327155	0.9392	12.2628
SAMD00013607	PRJDB2660	SAMD00013607	Mishima_Ushi	450765777	426042978	0.9452	14.2032
SAMD00013612	PRJDB2660	SAMD00013612	Mishima_Ushi	521540023	493461935	0.9462	16.4843
SAMD00013609	PRJDB2660	SAMD00013609	Mishima_Ushi	479680457	452652889	0.9437	15.0837
SAMD00013606	PRJDB2660	SAMD00013606	Mishima_Ushi	438363906	412837252	0.9418	13.7926
SRR10809649	PRJNA598339	SAMN13703162	Mongolian	316202295	314430849	0.9944	13.1751
SRR10809650	PRJNA598339	SAMN13703161	Mongolian	199833849	198337074	0.9925	9.25478
SRR10809656	PRJNA598339	SAMN13703170	Mongolian	287851171	286429120	0.9951	13.2804
SRR10809658	PRJNA598339	SAMN13703168	Mongolian	246112216	244686904	0.9942	11.626
SRR10809660	PRJNA598339	SAMN13703166	Mongolian	208850407	207767691	0.9948	10.0581
SRR10809661	PRJNA598339	SAMN13703165	Mongolian	204192517	203128105	0.9948	9.83417
SRR5507261	PRJNA379859	SAMN06698985	Mongolian	223316768	222066444	0.9944	11.3768
SRR5507262	PRJNA379859	SAMN06698984	Mongolian	254082123	252707821	0.9946	12.9084
SRR5507263	PRJNA379859	SAMN06698983	Mongolian	251155374	249771608	0.9945	12.7815
SRR5507264	PRJNA379859	SAMN06698982	Mongolian	247762714	246265793	0.994	12.7879
SRR5507265	PRJNA379859	SAMN06698981	Mongolian	262583284	261067506	0.9942	13.3996
SRR5507266	PRJNA379859	SAMN06698980	Mongolian	250133567	248620378	0.994	12.2014
SRR5507267	PRJNA379859	SAMN06698979	Mongolian	245820705	244684012	0.9954	12.0139
SAMN05216071	PRJNA324822	SAMN05216071	Shorthorn	309831488	305665646	0.9866	15.8806
SRR10752668	PRJNA343262	SAMN13655882	Shorthorn	565218172	529966728	0.9376	25.9321
SRR10752669	PRJNA343262	SAMN13655881	Shorthorn	563365756	560810789	0.9955	26.7084
SRR10752670	PRJNA343262	SAMN13655880	Shorthorn	612492098	563683739	0.9203	27.7077
SRR10752671	PRJNA343262	SAMN13655879	Shorthorn	628452778	624172275	0.9932	30.4353
SRR10752672	PRJNA343262	SAMN13655878	Shorthorn	655533750	640847143	0.9776	31.2185
SRR10752673	PRJNA343262	SAMN13655877	Shorthorn	577851053	575133858	0.9953	28.2055
SRR10752674	PRJNA343262	SAMN13655876	Shorthorn	603367023	561120790	0.93	27.425
SRR10752676	PRJNA343262	SAMN13655874	Shorthorn	566164771	563117088	0.9946	27.2779
SRR10752678	PRJNA343262	SAMN13655873	Shorthorn	619275381	615902864	0.9946	30.1995
SRR10752679	PRJNA343262	SAMN13655872	Shorthorn	561716037	556991029	0.9916	27.2257
SRR10752680	PRJNA343262	SAMN13655871	Shorthorn	635610572	631204439	0.9931	30.7516
SRR10752681	PRJNA343262	SAMN13655870	Shorthorn	624282714	612285745	0.9808	30.136
SRR10752682	PRJNA343262	SAMN13655869	Shorthorn	616102290	611644070	0.9928	30.2563
SRR10752683	PRJNA343262	SAMN13655868	Shorthorn	595824373	585832062	0.9832	27.5841
SRR10752684	PRJNA343262	SAMN13655867	Shorthorn	390250409	388284506	0.995	18.843
SRR10752685	PRJNA343262	SAMN13655866	Shorthorn	606646277	602360489	0.9929	29.5873
SRR5507243	PRJNA379859	SAMN06699003	Tibetan	291454465	289999684	0.995	14.618
SRR5507244	PRJNA379859	SAMN06699002	Tibetan	233910592	232688302	0.9948	11.7153
SRR5507245	PRJNA379859	SAMN06699001	Tibetan	227569692	226425108	0.995	11.5445
SRR5507246	PRJNA379859	SAMN06699000	Tibetan	207289233	206122187	0.9944	10.4396

SRR5507247	PRJNA379859	SAMN06698999	Tibetan	230925713	229799144	0.9951	11.6493
SRR5507248	PRJNA379859	SAMN06698998	Tibetan	269813666	268428825	0.9949	13.5936
SRR5507249	PRJNA379859	SAMN06698997	Tibetan	223502745	222445769	0.9953	11.3357
SRR5507250	PRJNA379859	SAMN06698996	Tibetan	233281092	231860864	0.9939	11.7343
SRR5507251	PRJNA379859	SAMN06698995	Tibetan	230592394	229316765	0.9945	11.6335
SRR6234784	PRJNA396672	SAMN07431022	Tibetan	278226327	276823087	0.995	10.1501
SRR6234785	PRJNA396672	SAMN07431023	Tibetan	361518783	359621805	0.9948	13.0162
SRR5507215	PRJNA379859	SAMN06699031	Lingnan	220446742	218406957	0.9907	10.9552
SRR5507216	PRJNA379859	SAMN06699030	Lingnan	220063041	218527384	0.993	11.2247
SRR5507217	PRJNA379859	SAMN06699029	Lingnan	193294523	191835926	0.9925	9.73377
SRR5507218	PRJNA379859	SAMN06699028	Lingnan	189338652	188107316	0.9935	9.71742
SRR5507219	PRJNA379859	SAMN06699027	Lingnan	231684973	230309909	0.9941	11.2136
SRR5507220	PRJNA379859	SAMN06699026	Lingnan	231320294	229437613	0.9919	11.0968
SRR5507221	PRJNA379859	SAMN06699025	Lingnan	187391057	185784060	0.9914	9.39849
SRR5507222	PRJNA379859	SAMN06699024	Lingnan	236449010	235090406	0.9943	11.6367
SRR5507238	PRJNA379859	SAMN06699008	Luxi	225412375	223797426	0.9928	11.2876
SRR5507239	PRJNA379859	SAMN06699007	Luxi	186526429	184987895	0.9918	9.29899
SRR5507241	PRJNA379859	SAMN06699005	Luxi	227965202	226066904	0.9917	11.0163
SRR5507242	PRJNA379859	SAMN06699004	Luxi	246185985	244746661	0.9942	12.0031
SRR6234786	PRJNA396672	SAMN07431024	Luxi	356983895	355324133	0.9954	12.7511
SRR6234787	PRJNA396672	SAMN07431025	Luxi	333509269	331917644	0.9952	11.8983
SRR14765445	PRJNA431934	SAMN19491791	Brahman	334555110	332417543	0.9936	16.7671
SRR14765456	PRJNA431934	SAMN19491790	Brahman	449565758	446710559	0.9936	22.1346
SRR14765513	PRJNA431934	SAMN19491789	Brahman	265364163	264042583	0.995	12.8522
SRR6649997	PRJNA432125	SAMN08435316	Brahman	220823636	218371560	0.9889	11.6139
SRR6649998	PRJNA432125	SAMN08435295	Brahman	211493536	210811034	0.9968	11.2339
SRR6650019	PRJNA432125	SAMN08435284	Brahman	195827557	194596632	0.9937	10.6093
SRR6650020	PRJNA432125	SAMN08435281	Brahman	418909851	413220557	0.9864	16.0781
SRR6650021	PRJNA432125	SAMN08435282	Brahman	410379649	402731938	0.9814	15.5361
SRR6650022	PRJNA432125	SAMN08435279	Brahman	423429073	416247844	0.983	16.1252
SRR6650023	PRJNA432125	SAMN08435280	Brahman	423726581	418765607	0.9883	16.2363
SRR6650024	PRJNA432125	SAMN08435317	Brahman	227009483	224812098	0.9903	11.9445
SRR6650026	PRJNA432125	SAMN08435327	Brahman	237771445	236548444	0.9949	12.7438
SRR6650029	PRJNA432125	SAMN08435322	Brahman	206687504	205132999	0.9925	11.1547
SRR6650031	PRJNA432125	SAMN08435324	Brahman	446655476	441927388	0.9894	17.5568
SRR6650032	PRJNA432125	SAMN08435323	Brahman	228354933	227253360	0.9952	12.1243
DRR295117	PRJDB10918	SAMD00324137	Wenling	236514302	235504002	0.9957	10.283
DRR295118	PRJDB10918	SAMD00324138	Wenling	254811213	253594675	0.9952	11.0008
DRR295119	PRJDB10918	SAMD00324139	Wenling	209585337	208667012	0.9956	9.34973
DRR295120	PRJDB10918	SAMD00324140	Wenling	234575562	233514156	0.9955	10.113
DRR295121	PRJDB10918	SAMD00324141	Wenling	245590547	244468526	0.9954	10.7623
DRR295122	PRJDB10918	SAMD00324142	Wenling	219406825	218292245	0.9949	9.66524
DRR295123	PRJDB10918	SAMD00324143	Wenling	241745217	240687756	0.9956	10.5905
DRR295124	PRJDB10918	SAMD00324144	Wenling	221195322	220231915	0.9956	9.7298
DRR295125	PRJDB10918	SAMD00324145	Wenling	207058985	205838279	0.9941	9.14163
SRR6234762	PRJNA396672	SAMN07431013	Wenling	363679335	361648878	0.9944	12.4333
SRR6234763	PRJNA396672	SAMN07431012	Wenling	361688409	359546320	0.9941	12.329
SRR6024562	PRJNA379859	SAMN07622457	Wenshan	249396868	247067912	0.9907	11.1771
SRR6024569	PRJNA379859	SAMN07622458	Wenshan	260371785	256239244	0.9841	11.0827
SRR6024570	PRJNA379859	SAMN07622459	Wenshan	264460002	262406569	0.9922	11.7051
SRR6024575	PRJNA379859	SAMN07622462	Wenshan	257523084	255594335	0.9925	11.0839

SRR6024576	PRJNA379859	SAMN07622463	Wenshan	209037365	207486192	0.9926	9.79379
SRR6024577	PRJNA379859	SAMN07622460	Wenshan	261555149	259524466	0.9922	11.7409
SRR6024578	PRJNA379859	SAMN07622461	Wenshan	332699688	330347171	0.9929	14.604
SRR16949978	PRJNA779877	SAMN23078533	Xiangxi	245108915	244556582	0.9977	10.8861
SRR16949980	PRJNA779877	SAMN23078531	Xiangxi	253703749	253088593	0.9976	10.9304
SRR16949982	PRJNA779877	SAMN23078529	Xiangxi	262469228	261750302	0.9973	11.736
SRR16949983	PRJNA779877	SAMN23078528	Xiangxi	268301334	267664201	0.9976	12.0215
SRR16949984	PRJNA779877	SAMN23078527	Xiangxi	260237570	259391297	0.9967	11.5686
SRR16949986	PRJNA779877	SAMN23078545	Xiangxi	237991601	236554569	0.994	11.1644
SRR16949987	PRJNA779877	SAMN23078544	Xiangxi	223135082	221653053	0.9934	10.4947
SRR16949989	PRJNA779877	SAMN23078543	Xiangxi	224188433	222884119	0.9942	10.4412
SRR16949992	PRJNA779877	SAMN23078540	Xiangxi	250144088	249505775	0.9974	10.5045
SRR16949994	PRJNA779877	SAMN23078538	Xiangxi	289927429	289113010	0.9972	12.0444
SRR16949995	PRJNA779877	SAMN23078537	Xiangxi	275525129	274871984	0.9976	12.2069
SRR16949998	PRJNA779877	SAMN23078534	Xiangxi	278846237	278137466	0.9975	12.2576
SRR16949999	PRJNA779877	SAMN23078525	Xiangxi	240867254	240037783	0.9966	10.6959
SRR16950000	PRJNA779877	SAMN23078524	Xiangxi	241298509	240609487	0.9971	10.6823
DRR295126	PRJDB10918	SAMD00324146	Zhoushan	225266614	224331515	0.9958	9.87764
DRR295127	PRJDB10918	SAMD00324147	Zhoushan	211035818	210072556	0.9954	9.34666
DRR295128	PRJDB10918	SAMD00324148	Zhoushan	229421786	228421569	0.9956	10.0926
DRR295129	PRJDB10918	SAMD00324149	Zhoushan	238440681	237327887	0.9953	10.482
DRR295130	PRJDB10918	SAMD00324150	Zhoushan	226563931	226098157	0.9979	10.1143
DRR295131	PRJDB10918	SAMD00324151	Zhoushan	222411184	221959113	0.998	9.96424
