# Supplementary material for: Identification of genomic diversity and selection signatures in Luxi cattle using whole-genome sequencing data
Source: Anim Biosci. 2024 Jan 20;37(3):461–70. doi: 10.5713/ab.23.0304 (PMC10915192; doi:10.5713/ab.23.0304)
Supplement: Supplementary file 2 [file ab-23-0304-Supplementary-Table-S2.pdf]

Supplementary Table S2. Summary of sequencing data

Sample ID	Breed	Tissue	Raw Reads	Clean Reads	Raw Base(G)	Clean Base(G)	Effective Rate(%)	Error Rate(%)	Q20(%)	Q30(%)	GC Content(%)	Mapping ratio(%)	Depth
Luxi-1	Luxi	blood	225705438	224957214	33.86	33.74	99.67	0.03	97.68	92.97	43.53	99.72	12.0987
Luxi-2	Luxi	blood	381,834,922	378,431,764	57.28	56.76	99.11	0.05	96.01	89.52	43.12	99.86	20.0201
Luxi-3	Luxi	blood	227,519,650	223,416,212	34.13	33.51	98.20	0.04	96.24	89.86	42.53	99.85	11.8223
Luxi-4	Luxi	blood	216,401,210	212,732,256	32.46	31.91	98.30	0.04	96.13	89.49	42.90	99.87	11.3496
Luxi-5	Luxi	blood	211,831,464	208,269,308	31.77	31.24	98.32	0.04	95.91	88.87	42.81	99.84	11.1888
Luxi-6	Luxi	blood	225,317,702	224,097,752	33.80	33.61	99.46	0.05	97.42	92.27	43.14	99.86	11.9117
Luxi-7	Luxi	blood	207,688,570	206,711,370	31.15	31.01	99.53	0.05	97.02	90.99	42.61	99.86	10.8858
Luxi-8	Luxi	blood	222956276	218929366	33.44	32.84	98.19	0.04	96.19	89.72	43.43	99.86	11.6742
Luxi-9	Luxi	blood	325808630	325149892	48.87	48.77	99.80	0.03	96.62	90.40	44.25	99.66	17.3121
Luxi-10	Luxi	blood	287,083,490	286,573,590	43.06	42.99	99.82	0.03	96.09	88.79	43.94	99.74	15.2362
Luxi-11	Luxi	blood	263,658,264	263,137,714	39.55	39.47	99.80	0.03	96.99	90.59	43.81	99.55	14.0839
Luxi-12	Luxi	blood	240,494,606	240,012,300	36.07	36.00	99.80	0.03	96.87	90.35	43.84	99.77	12.9371
Luxi-13	Luxi	blood	242,974,042	242,405,688	36.45	36.36	99.77	0.04	95.82	87.43	43.92	99.70	13.0384
Luxi-14	Luxi	blood	254,719,428	254,197,654	38.21	38.13	99.80	0.03	96.44	89.13	43.89	99.73	13.6366
Luxi-15	Luxi	blood	279,489,154	278,805,210	41.92	41.82	99.76	0.03	96.07	88.14	44.00	99.47	14.7887
Luxi-16	Luxi	blood	267980356	267333982	40.20	40.10	99.76	0.03	96.23	88.52	44.03	99.44	14.2236
