# Supplementary material for: Identification of genomic diversity and selection signatures in Luxi cattle using whole-genome sequencing data
Source: Anim Biosci. 2024 Jan 20;37(3):461–70. doi: 10.5713/ab.23.0304 (PMC10915192; doi:10.5713/ab.23.0304)
Supplement: Supplementary file 3 [file ab-23-0304-Supplementary-Table-S3.pdf]

**Supplementary Table S3.** Distribution of SNPs identified in LUX annotated by SnpEff.

| Variants Type | Count | Percent |
|---|---|---|
| 3_prime_UTR_variant | 648, 748 | 0.783% |
| 5_prime_UTR_premature_start_codon_gain_variant | 25,965 | 0.031% |
| 5_prime_UTR_variant | 149,518 | 0.180% |
| downstream_gene_variant | 4,169,606 | 5.031% |
| initiator_codon_variant | 42 | 0.000% |
| intergenic_region | 17,829,235 | 21.514% |
| intragenic_variant | 277,815 | 0.335% |
| intron_variant | 54,840,227 | 66.173% |
| missense_variant | 239,036 | 0.288% |
| non_coding_transcript_exon_variant | 243,335 | 0.294% |
| splice_acceptor_variant | 1,075 | 0.001% |
| splice_donor_variant | 1,401 | 0.002% |
| splice_region_variant | 90,096 | 0.109% |
| start_lost | 381 | 0.000% |
| start_retained_variant | 53 | 0.000% |
| stop_gained | 2,151 | 0.003% |
| stop_lost | 298 | 0.000% |
| stop_retained_variant | 384 | 0.000% |
| synonymous_variant | 501,711 | 0.605% |
| upstream_gene_variant | 3,852,636 | 4.649% |

| Region Type | Count | Percent |
|---|---|---|
| DOWNSTREAM | 4,169,606 | 5.037% |
| EXON | 974,840 | 1.178% |
| INTERGENIC | 17,829,235 | 21.537% |
| INTRON | 54,768,088 | 66.158% |
| SPLICE_SITE_ACCEPTOR | 1,075 | 0.001% |
| SPLICE_SITE_DONOR | 1,380 | 0.002% |
| SPLICE_SITE_REGION | 85,324 | 0.103% |
| TRANSCRIPT | 277,815 | 0.336% |
| UPSTREAM | 3,852,636 | 4.654% |
| UTR_3_PRIME | 648,748 | 0.784% |
| UTR_5_PRIME | 175,483 | 0.212% |
