# Supplementary material for: Identification of genomic diversity and selection signatures in Luxi cattle using whole-genome sequencing data
Source: Anim Biosci. 2024 Jan 20;37(3):461–70. doi: 10.5713/ab.23.0304 (PMC10915192; doi:10.5713/ab.23.0304)
Supplement: Supplementary file 4 [file ab-23-0304-Supplementary-Table-S4.pdf]

Supplementary Table S4. Genetic diversity of the 15 cattle breeds used in this study.

Breed	Code	Sample Size	*pi*	H_{O}	H_{E}	F_{HOM}	F_{ROH}
Luxi	LUX	22	0.00305	0.2556	0.2482	-0.02982	0.1765
Angus	ANG	20	0.00127	0.1046	0.1028	-0.01806	0.4555
Brahman	BRA	15	0.00287	0.2312	0.2316	0.00008	0.2384
Chaidamu	CHA	8	0.00192	0.1593	0.1492	-0.06760	0.3164
Hanwoo	HAN	20	0.00151	0.1223	0.1225	0.00022	0.3611
Kazakh	KAZ	10	0.00175	0.1374	0.1392	0.01253	0.3373
Lingnan	LIN	8	0.00325	0.2360	0.2553	0.07559	0.2375
Mishima_Ushi	MIS	8	0.00063	0.0510	0.0486	-0.05183	0.7226
Mongolian	MON	13	0.00195	0.1560	0.1566	0.00340	0.2868
Shorthorn	SHO	17	0.00127	0.1090	0.1023	-0.06462	0.4409
Tibetan	TIB	11	0.00179	0.1398	0.1438	0.02609	0.3421
Wenling	WEL	11	0.00313	0.2441	0.2494	0.02086	0.2979
Wenshan	WES	7	0.00321	0.2521	0.2505	-0.00797	0.2088
Xiangxi	XIA	14	0.00333	0.2544	0.2680	0.04985	0.1931
Zhoushan	ZHO	6	0.00298	0.2408	0.2280	-0.05656	0.2967
