# Supplementary material for: Identification of genomic diversity and selection signatures in Luxi cattle using whole-genome sequencing data
Source: Anim Biosci. 2024 Jan 20;37(3):461–70. doi: 10.5713/ab.23.0304 (PMC10915192; doi:10.5713/ab.23.0304)
Supplement: Supplementary file 5 [file ab-23-0304-Supplementary-Table-S5.pdf]

Supplementary Table S5. A summary of genes from *pi* in LUX.

Chromosome	Starting position	Ending position	*pi*	Gene
1	99750001	99800000	0.00117726	*PDCD10*
24	43350001	43400000	0.00117714	*LDLRAD4*
3	62500001	62550000	0.00117707	*ADGRL2*
6	72425001	72475000	0.00117701	*IGFBP7*
3	96075001	96125000	0.00117691	*ELAVL4*
8	59825001	59875000	0.00117683	*TPM2*
8	59825001	59875000	0.00117683	*CA9*
8	59825001	59875000	0.00117683	*TLN1*
8	59825001	59875000	0.00117683	*ARHGEF39*
8	59825001	59875000	0.00117683	*CCDC107*
8	59825001	59875000	0.00117683	*SIT1*
2	87875001	87925000	0.00117678	*SATB2*
12	40550001	40600000	0.00117661	*PCDH9*
1	136975001	137025000	0.0011766	*ACAD11*
3	75000001	75050000	0.0011766	*LRRC40*
3	75000001	75050000	0.0011766	*LRRC7*
1	136975001	137025000	0.0011766	*ACKR4*
11	61225001	61275000	0.00117659	*EHBP1*
6	44150001	44200000	0.00117654	*DHX15*
11	29425001	29475000	0.00117649	*TTC7A*
20	23475001	23525000	0.00117647	*SLC38A9*
13	38250001	38300000	0.00117598	*OVOL2*
13	38250001	38300000	0.00117598	*MGME1*
6	83300001	83350000	0.00117538	*STAP1*
6	83300001	83350000	0.00117538	*UBA6*
3	99725001	99775000	0.00117523	*NSUN4*
3	99725001	99775000	0.00117523	*FAAH*
14	62075001	62125000	0.00117511	*UBR5*
1	80525001	80575000	0.00117492	*FETUB*
1	80525001	80575000	0.00117492	*HRG*
1	80525001	80575000	0.00117492	*KNG1*
10	59750001	59800000	0.00117489	*USP8*
10	59750001	59800000	0.00117489	*USP50*
10	59750001	59800000	0.00117489	*TRPM7*
10	59750001	59800000	0.00117489	*U6*
20	14200001	14250000	0.00117481	*ADAMTS6*
2	119925001	119975000	0.00117456	*DIS3L2*
9	60325001	60375000	0.00117449	*BACH2*
9	60325001	60375000	0.00117449	*GJA10*
9	60325001	60375000	0.00117449	*CASP8AP2*
12	36300001	36350000	0.00117384	*PSPC1*
25	26750001	26800000	0.00117362	*FBRS*
18	53275001	53325000	0.00117362	*DMPK*
25	26750001	26800000	0.00117362	*PRR14*
25	26750001	26800000	0.00117362	*ZNF689*
18	53275001	53325000	0.00117362	*SIX5*
18	53275001	53325000	0.00117362	*DMWD*
3	32275001	32325000	0.00117345	*DRAM2*
9	103875001	103925000	0.00117341	*DLL1*
20	14625001	14675000	0.0011734	*CWC27*

2	71575001	71625000	0.00117337	*PTPN4*
10	85500001	85550000	0.00117325	*LIN52*
19	33775001	33825000	0.00117318	*AKAP10*
19	33775001	33825000	0.00117318	*ULK2*
6	44175001	44225000	0.00117307	*DHX15*
3	83850001	83900000	0.00117303	*PATJ*
11	93975001	94025000	0.00117299	*RC3H2*
11	93975001	94025000	0.00117299	*PDCL*
15	29475001	29525000	0.0011726	*BCL9L*
15	29475001	29525000	0.0011726	*CXCR5*
18	44875001	44925000	0.00117253	*WTIP*
8	59850001	59900000	0.00117237	*TLN1*
8	59850001	59900000	0.00117237	*TPM2*
8	59850001	59900000	0.00117237	*CA9*
1	145450001	145500000	0.00117233	*PCBP3*
7	46050001	46100000	0.0011723	*JADE2*
1	69575001	69625000	0.00117226	*SLC12A8*
12	24875001	24925000	0.00117211	*SERTM1*
5	107725001	107775000	0.00117205	*RAD52*
2	106500001	106550000	0.00117197	*USP37*
2	106500001	106550000	0.00117197	*CNOT9*
14	21025001	21075000	0.00117194	*PCMTD1*
23	625001	675000	0.00117181	*KHDRBS2*
10	21550001	21600000	0.00117179	*EFS*
10	21550001	21600000	0.00117179	*PABPN1*
10	21550001	21600000	0.00117179	*SLC22A17*
10	21550001	21600000	0.00117179	*IL25*
18	18700001	18750000	0.0011715	*BRD7*
18	18700001	18750000	0.0011715	*ADCY7*
21	41225001	41275000	0.00117146	*G2E3*
21	41225001	41275000	0.00117146	*SCFD1*
22	50350001	50400000	0.00117141	*MST1R*
22	50350001	50400000	0.00117141	*CAMKV*
22	50350001	50400000	0.00117141	*MON1A*
22	50350001	50400000	0.00117141	*ACTL11*
22	50350001	50400000	0.00117141	*TRAIP*
1	58600001	58650000	0.00117127	*GRAMD1C*
1	58600001	58650000	0.00117127	*CCDC191*
1	58600001	58650000	0.00117127	*ZDHHC23*
15	36025001	36075000	0.00117116	*SOX6*
2	106250001	106300000	0.00117107	*ARPC2*
2	106250001	106300000	0.00117107	*AAMP*
2	106250001	106300000	0.00117107	*GPBAR1*
2	106250001	106300000	0.00117107	*PNKD*
13	56650001	56700000	0.00117105	*SYCP2*
13	56650001	56700000	0.00117105	*PHACTR3*
3	94900001	94950000	0.00117102	*EPS15*
11	87850001	87900000	0.00117102	*YWHAQ*
4	112275001	112325000	0.00117086	*ZNF398*
3	50850001	50900000	0.00117063	*EVI5*
4	61225001	61275000	0.00117044	*SEPTIN7*
4	61225001	61275000	0.00117044	*U6*

10	61900001	61950000	0.00117031	*FBN1*
2	119625001	119675000	0.00117001	*DIS3L2*
2	119625001	119675000	0.00117001	*SNORA62*
19	43450001	43500000	0.00116979	*ETV4*
2	71700001	71750000	0.00116965	*PTPN4*
5	67700001	67750000	0.00116927	*HCFC2*
5	56750001	56800000	0.0011692	*PTGES3*
5	56750001	56800000	0.0011692	*ATP5F1B*
5	56750001	56800000	0.0011692	*BAZ2A*
5	56750001	56800000	0.0011692	*SNORD59A*
23	350001	400000	0.001169	*KHDRBS2*
1	83050001	83100000	0.00116898	*HTR3C*
7	52750001	52800000	0.00116888	*ARAP3*
7	52750001	52800000	0.00116888	*U4*
8	59875001	59925000	0.00116883	*TLN1*
8	59875001	59925000	0.00116883	*GBA2*
8	59875001	59925000	0.00116883	*RGP1*
8	59875001	59925000	0.00116883	*CREB3*
8	59875001	59925000	0.00116883	*MSMP*
16	50925001	50975000	0.00116882	*NADK*
16	50925001	50975000	0.00116882	*SLC35E2*
16	50925001	50975000	0.00116882	*GNB1*
2	53875001	53925000	0.00116867	*KYNU*
10	12400001	12450000	0.00116864	*DPP8*
10	12400001	12450000	0.00116864	*HACD3*
3	101100001	101150000	0.00116846	*EIF2B3*
26	44325001	44375000	0.00116839	*FAM53B*
7	43975001	44025000	0.00116839	*TCF3*
26	44325001	44375000	0.00116839	*EEF1AKMT2*
7	43975001	44025000	0.00116839	*UQCR11*
12	28625001	28675000	0.00116821	*BRCA2*
12	28625001	28675000	0.00116821	*ZAR1L*
5	81350001	81400000	0.0011681	*CCDC91*
5	81350001	81400000	0.0011681	*U6*
29	48925001	48975000	0.00116806	*KCNQ1*
26	31525001	31575000	0.00116806	*SHOC2*
5	119525001	119575000	0.00116806	*MOV10L1*
5	119525001	119575000	0.00116806	*TRABD*
5	119525001	119575000	0.00116806	*PANX2*
5	119525001	119575000	0.00116806	*SELENOO*
9	93775001	93825000	0.00116803	*ARID1B*
5	35275001	35325000	0.00116802	*DBX2*
1	126650001	126700000	0.00116794	*XRN1*
8	200001	250000	0.00116782	*MFSD14B*
13	18775001	18825000	0.00116779	*PARD3*
21	27425001	27475000	0.00116778	*MTMR10*
21	27425001	27475000	0.00116778	*FAN1*
5	86125001	86175000	0.00116774	*SOX5*
14	29325001	29375000	0.00116732	*CYP7B1*
1	2850001	2900000	0.00116722	*SYNJ1*
5	36875001	36925000	0.00116712	*ADAMTS20*
17	69225001	69275000	0.00116696	*LIF*

3	95000001	95050000	0.00116686	*RNF11*
3	95000001	95050000	0.00116686	*TTC39A*
7	82075001	82125000	0.00116684	*ATG10*
14	19250001	19300000	0.00116659	*SPIDR*
17	56825001	56875000	0.00116634	*SUDS3*
14	64650001	64700000	0.00116632	*VPS13B*
2	125850001	125900000	0.00116623	*WASF2*
2	125850001	125900000	0.00116623	*GPR3*
2	125850001	125900000	0.00116623	*U6*
16	44775001	44825000	0.00116578	*RERE*
2	107100001	107150000	0.00116576	*NHEJ1*
2	107100001	107150000	0.00116576	*SLC23A3*
2	107100001	107150000	0.00116576	*RETREG2*
2	107100001	107150000	0.00116576	*CNPPD1*
5	34300001	34350000	0.00116565	*ARID2*
5	34300001	34350000	0.00116565	*SCAF11*
5	29125001	29175000	0.00116554	*ATF1*
13	38425001	38475000	0.0011655	*DZANK1*
8	77150001	77200000	0.00116541	*KIF27*
8	77150001	77200000	0.00116541	*C8H9orf64*
14	700001	750000	0.00116534	*MAF1*
14	700001	750000	0.00116534	*SHARPIN*
14	700001	750000	0.00116534	*HGH1*
14	700001	750000	0.00116534	*WDR97*
14	700001	750000	0.00116534	*TSSK5*
14	700001	750000	0.00116534	*MROH1*
14	700001	750000	0.00116534	*CYC1*
15	77300001	77350000	0.00116532	*CELF1*
2	88000001	88050000	0.00116521	*SATB2*
22	45400001	45450000	0.001165	*ERC2*
11	27175001	27225000	0.00116475	*CAMKMT*
19	32950001	33000000	0.00116472	*TVP23B*
2	91725001	91775000	0.0011647	*RAPH1*
12	29250001	29300000	0.00116428	*RXFP2*
11	29575001	29625000	0.00116428	*CALM2*
7	600001	650000	0.00116399	*CNOT6*
5	25850001	25900000	0.00116396	*SMUG1*
13	22875001	22925000	0.0011639	*MLLT10*
16	29700001	29750000	0.00116371	*ITPKB*
3	33450001	33500000	0.00116342	*CSF1*
7	50675001	50725000	0.00116337	*DNAJC18*
7	50675001	50725000	0.00116337	*ECSCR*
7	50675001	50725000	0.00116337	*SPATA24*
25	36050001	36100000	0.00116322	*TSC22D4*
25	36050001	36100000	0.00116322	*NYAP1*
25	36050001	36100000	0.00116322	*SPACDR*
4	43650001	43700000	0.00116311	*PTPN12*
2	126425001	126475000	0.00116308	*ARID1A*
7	45850001	45900000	0.00116306	*PPP2CA*
19	20800001	20850000	0.00116306	*GIT1*
19	20800001	20850000	0.00116306	*TP53I13*
19	20800001	20850000	0.00116306	*ABHD15*

19	20800001	20850000	0.00116306	*ANKRD13B*
19	20800001	20850000	0.00116306	*TAOK1*
21	22725001	22775000	0.00116304	*PDE8A*
3	100450001	100500000	0.00116297	*NASP*
3	100450001	100500000	0.00116297	*GPBP1L1*
3	100450001	100500000	0.00116297	*CCDC17*
26	22650001	22700000	0.00116259	*ARMH3*
12	28725001	28775000	0.00116248	*FRY*
21	7750001	7800000	0.00116247	*IGF1R*
27	33475001	33525000	0.00116213	*NSD3*
3	20150001	20200000	0.00116177	*TARS2*
3	20150001	20200000	0.00116177	*ECM1*
3	20150001	20200000	0.00116177	*ADAMTSL4*
10	53925001	53975000	0.00116172	*MNS1*
10	48900001	48950000	0.00116165	*RORA*
9	39025001	39075000	0.00116158	*REV3L*
22	44825001	44875000	0.00116153	*ERC2*
5	56250001	56300000	0.00116138	*LRP1*
5	81325001	81375000	0.00116131	*CCDC91*
5	81325001	81375000	0.00116131	*U6*
4	80900001	80950000	0.00116092	*SUGCT*
4	80900001	80950000	0.00116092	*MPLKIP*
11	73200001	73250000	0.00116079	*HADHB*
11	73200001	73250000	0.00116079	*ADGRF3*
5	34275001	34325000	0.00116076	*SCAF11*
5	34275001	34325000	0.00116076	*ARID2*
22	49450001	49500000	0.00116071	*DOCK3*
2	44625001	44675000	0.00116038	*NEB*
2	44625001	44675000	0.00116038	*RIF1*
16	72050001	72100000	0.00116034	*RCOR3*
25	20475001	20525000	0.00116028	*HS3ST2*
5	35975001	36025000	0.00116026	*TMEM117*
25	27175001	27225000	0.00116023	*ZNF646*
25	27175001	27225000	0.00116023	*ZNF668*
25	27175001	27225000	0.00116023	*KAT8*
25	27175001	27225000	0.00116023	*PRSS53*
25	27175001	27225000	0.00116023	*BCKDK*
25	27175001	27225000	0.00116023	*VKORC1*
9	18625001	18675000	0.00116019	*PHIP*
21	6875001	6925000	0.00116005	*MEF2A*
5	56225001	56275000	0.00116004	*LRP1*
5	56225001	56275000	0.00116004	*NXPH4*
10	59275001	59325000	0.00116	*TNFAIP8L3*
21	2150001	2200000	0.00115996	*SNORD115*
21	2150001	2200000	0.00115996	*SNORD115*
21	2150001	2200000	0.00115996	*SNORD115*
21	2150001	2200000	0.00115996	*SNORD115*
21	2150001	2200000	0.00115996	*SNORD115*
21	2150001	2200000	0.00115996	*SNORD115*
5	23500001	23550000	0.00115986	*CRADD*
13	28000001	28050000	0.0011597	*SEPHS1*
23	41700001	41750000	0.0011595	*JARID2*

12	82300001	82350000	0.00115941	*ARGLU1*
11	43475001	43525000	0.00115914	*PAPOLG*
10	21025001	21075000	0.00115908	*PCK2*
10	21025001	21075000	0.00115908	*CPNE6*
10	21025001	21075000	0.00115908	*DCAF11*
10	21025001	21075000	0.00115908	*CARMIL3*
10	21025001	21075000	0.00115908	*NRL*
10	21025001	21075000	0.00115908	*PSME1*
10	73825001	73875000	0.00115906	*HIF1A*
7	41400001	41450000	0.0011588	*OR2AK3*
22	50400001	50450000	0.00115879	*TRAIP*
22	50400001	50450000	0.00115879	*UBA7*
22	50400001	50450000	0.00115879	*CDHR4*
22	50400001	50450000	0.00115879	*IP6K1*
22	50400001	50450000	0.00115879	*INKA1*
2	24750001	24800000	0.00115849	*DYNC1I2*
10	37150001	37200000	0.00115849	*MAPKBP1*
10	37150001	37200000	0.00115849	*MGA*
15	52975001	53025000	0.00115845	*FAM168A*
2	71650001	71700000	0.00115834	*PTPN4*
3	20250001	20300000	0.00115813	*RPRD2*
2	106000001	106050000	0.00115812	*TNS1*
22	29900001	29950000	0.00115795	*EIF4E3*
2	122600001	122650000	0.00115792	*PUM1*
5	26400001	26450000	0.00115791	*ATF7*
12	46525001	46575000	0.00115782	*DACH1*
5	26575001	26625000	0.0011578	*SP1*
5	26575001	26625000	0.0011578	*AMHR2*
13	60300001	60350000	0.00115767	*SLC52A3*
11	74450001	74500000	0.00115761	*NCOA1*
2	125825001	125875000	0.00115758	*WASF2*
2	125825001	125875000	0.00115758	*U6*
10	21950001	22000000	0.00115757	*MMP14*
10	21950001	22000000	0.00115757	*SLC7A7*
10	21950001	22000000	0.00115757	*LRP10*
10	21950001	22000000	0.00115757	*MRPL52*
7	54325001	54375000	0.00115745	*NR3C1*
11	94075001	94125000	0.00115727	*RABGAP1*
11	94075001	94125000	0.00115727	*ZBTB6*
11	94075001	94125000	0.00115727	*ZBTB26*
9	42650001	42700000	0.00115722	*PDSS2*
5	57450001	57500000	0.0011572	*SARNP*
27	33425001	33475000	0.0011572	*NSD3*
11	98550001	98600000	0.00115713	*ST6GALNAC6*
11	98550001	98600000	0.00115713	*ST6GALNAC4*
11	98550001	98600000	0.00115713	*PIP5KL1*
11	98575001	98625000	0.00115695	*FAM102A*
11	14650001	14700000	0.00115695	*DPY30*
11	98575001	98625000	0.00115695	*PIP5KL1*
11	98575001	98625000	0.00115695	*ST6GALNAC4*
11	14650001	14700000	0.00115695	*MEMO1*
11	14650001	14700000	0.00115695	*SPAST*

11	98575001	98625000	0.00115695	*DPM2*
19	12750001	12800000	0.00115678	*USP32*
1	71150001	71200000	0.00115673	*NRROS*
6	84075001	84125000	0.00115663	*YTHDC1*
16	42825001	42875000	0.00115647	*CASZ1*
9	94925001	94975000	0.00115643	*TULP4*
9	28725001	28775000	0.00115618	*SERINC1*
9	28725001	28775000	0.00115618	*HSF2*
21	33775001	33825000	0.00115618	*FAM219B*
21	33775001	33825000	0.00115618	*SCAMP2*
21	33775001	33825000	0.00115618	*MPI*
21	33775001	33825000	0.00115618	*COX5A*
26	18475001	18525000	0.00115615	*ARHGAP19*
12	19400001	19450000	0.0011561	*SPRYD7*
12	19400001	19450000	0.0011561	*TRIM13*
8	74850001	74900000	0.00115606	*B4GALT1*
13	33850001	33900000	0.00115589	*ZEB1*
16	64500001	64550000	0.00115585	*SMG7*
22	17150001	17200000	0.00115585	*SETD5*
16	64500001	64550000	0.00115585	*NMNAT2*
18	45225001	45275000	0.00115574	*U6*
4	56875001	56925000	0.00115554	*IMMP2L*
11	43625001	43675000	0.00115554	*REL*
11	43625001	43675000	0.00115554	*PUS10*
3	22500001	22550000	0.00115549	*PRKAB2*
3	22500001	22550000	0.00115549	*FMO5*
1	72175001	72225000	0.00115536	*ACAP2*
3	52525001	52575000	0.00115525	*BARHL2*
4	30225001	30275000	0.00115508	*SP4*
5	45625001	45675000	0.00115489	*IFNG*
15	53000001	53050000	0.00115478	*FAM168A*
3	91875001	91925000	0.00115464	*SSBP3*
19	22525001	22575000	0.00115446	*YWHAE*
19	22525001	22575000	0.00115446	*CRK*
3	58425001	58475000	0.0011544	*ZNHIT6*
7	60225001	60275000	0.00115438	*ADRB2*
4	35600001	35650000	0.00115432	*SEMA3D*
10	20950001	21000000	0.00115427	*IPO4*
10	20950001	21000000	0.00115427	*RNF31*
10	20950001	21000000	0.00115427	*PSME2*
10	20950001	21000000	0.00115427	*IRF9*
10	20950001	21000000	0.00115427	*REC8*
10	20950001	21000000	0.00115427	*TSSK4*
10	20950001	21000000	0.00115427	*TM9SF1*
10	20950001	21000000	0.00115427	*CHMP4A*
10	20950001	21000000	0.00115427	*PSME1*
7	51175001	51225000	0.00115378	*NRG2*
15	52300001	52350000	0.00115372	*STARD10*
15	52300001	52350000	0.00115372	*ARAP1*
1	71325001	71375000	0.00115371	*PAK2*
1	71325001	71375000	0.00115371	*SENP5*
1	57725001	57775000	0.00115361	*NEPRO*

1	57725001	57775000	0.00115361	*GTPBP8*
5	55675001	55725000	0.0011536	*EEF1AKMT3*
5	55675001	55725000	0.0011536	*TSFM*
5	55675001	55725000	0.0011536	*CYP27B1*
5	55675001	55725000	0.0011536	*MARCHF9*
5	55675001	55725000	0.0011536	*METTL1*
5	55675001	55725000	0.0011536	*CDK4*
5	55675001	55725000	0.0011536	*AGAP2*
5	55675001	55725000	0.0011536	*TSPAN31*
5	55675001	55725000	0.0011536	*AVIL*
4	93850001	93900000	0.00115338	*KLHDC10*
14	69025001	69075000	0.00115318	*CFAP418*
29	45975001	46025000	0.00115301	*PPP6R3*
10	18800001	18850000	0.00115268	*MYO9A*
21	33350001	33400000	0.0011526	*PTPN9*
21	33350001	33400000	0.0011526	*SIN3A*
18	56175001	56225000	0.00115252	*MED25*
18	56175001	56225000	0.00115252	*PTOV1*
18	56175001	56225000	0.00115252	*AKT1S1*
18	56175001	56225000	0.00115252	*PNKP*
11	95025001	95075000	0.00115211	*DENND1A*
15	35825001	35875000	0.00115208	*SOX6*
4	85450001	85500000	0.00115205	*ING3*
4	85450001	85500000	0.00115205	*CPED1*
2	91250001	91300000	0.00115202	*CARF*
21	2100001	2150000	0.00115193	*SNORD116*
21	2100001	2150000	0.00115193	*SNORD116*
21	2100001	2150000	0.00115193	*SNORD116*
7	51000001	51050000	0.00115188	*PSD2*
7	51000001	51050000	0.00115188	*NRG2*
1	57700001	57750000	0.00115187	*GTPBP8*
1	57700001	57750000	0.00115187	*NEPRO*
5	111500001	111550000	0.00115183	*TNRC6B*
12	26225001	26275000	0.00115142	*NBEA*
14	51125001	51175000	0.00115113	*CSMD3*
20	39000001	39050000	0.00115113	*PRLR*
4	94375001	94425000	0.0011511	*COPG2*
16	39300001	39350000	0.00115101	*DNM3*
4	106725001	106775000	0.00115082	*CLCN1*
4	106725001	106775000	0.00115082	*FAM131B*
10	29800001	29850000	0.00115072	*FMN1*
26	31500001	31550000	0.00115072	*SHOC2*
13	46350001	46400000	0.00115069	*LARP4B*
13	46350001	46400000	0.00115069	*U6*
3	45425001	45475000	0.00115067	*DPYD*
19	40175001	40225000	0.00115062	*IKZF3*
19	40175001	40225000	0.00115062	*ZPBP2*
15	17600001	17650000	0.00115058	*CUL5*
5	73475001	73525000	0.00115004	*HMGXB4*
5	70300001	70350000	0.00115003	*CRY1*
17	13400001	13450000	0.00114958	*HHIP*
17	58750001	58800000	0.00114924	*MED13L*

11	73950001	74000000	0.00114923	*DNMT3A*
5	70600001	70650000	0.00114898	*BTBD11*
9	38175001	38225000	0.00114879	*LAMA4*
2	36700001	36750000	0.00114869	*BAZ2B*
12	26125001	26175000	0.00114864	*NBEA*
9	73825001	73875000	0.00114861	*PDE7B*
6	40050001	40100000	0.00114846	*SLIT2*
7	3900001	3950000	0.00114842	*MAU2*
7	3900001	3950000	0.00114842	*GATAD2A*
7	3900001	3950000	0.00114842	*U6*
9	15350001	15400000	0.00114838	*SENP6*
3	103525001	103575000	0.00114825	*PPIH*
3	103525001	103575000	0.00114825	*YBX1*
3	103525001	103575000	0.00114825	*CCDC30*
9	38775001	38825000	0.00114816	*FYN*
9	38775001	38825000	0.00114816	*TRAF3IP2*
23	9575001	9625000	0.00114807	*FKBP5*
22	33925001	33975000	0.00114802	*SUCLG2*
5	67750001	67800000	0.00114794	*HCFC2*
13	52275001	52325000	0.00114794	*PTPRA*
5	67750001	67800000	0.00114794	*NFYB*
13	52275001	52325000	0.00114794	*VPS16*
7	16675001	16725000	0.0011479	*TIMM44*
19	38400001	38450000	0.0011479	*CBX1*
19	38400001	38450000	0.0011479	*NFE2L1*
7	16675001	16725000	0.0011479	*ELAVL1*
19	38400001	38450000	0.0011479	*COPZ2*
7	16675001	16725000	0.0011479	*SNAPC2*
1	99700001	99750000	0.00114789	*SERPINI1*
1	99700001	99750000	0.00114789	*PDCD10*
13	38000001	38050000	0.0011478	*RRBP1*
12	25975001	26025000	0.00114762	*NBEA*
12	25975001	26025000	0.00114762	*MAB21L1*
17	69075001	69125000	0.00114737	*MTMR3*
27	15600001	15650000	0.00114723	*LRP2BP*
27	15600001	15650000	0.00114723	*SNX25*
21	22400001	22450000	0.00114703	*ZNF592*
5	36525001	36575000	0.00114698	*TMEM117*
3	110400001	110450000	0.00114697	*ZMYM4*
19	50800001	50850000	0.00114691	*GPS1*
19	50800001	50850000	0.00114691	*DUS1L*
19	50800001	50850000	0.00114691	*RFNG*
5	32600001	32650000	0.00114657	*RPAP3*
7	65925001	65975000	0.00114649	*LARP1*
3	32825001	32875000	0.00114622	*KCNA2*
2	122550001	122600000	0.00114601	*PUM1*
9	74700001	74750000	0.00114587	*MAP3K5*
5	66675001	66725000	0.00114585	*PAH*
19	23050001	23100000	0.00114562	*SMG6*
19	23050001	23100000	0.00114562	*DPH1*
19	23050001	23100000	0.00114562	*HIC1*
19	23050001	23100000	0.00114562	*RTN4RL1*

19	23050001	23100000	0.00114562	*OVCA2*
22	34425001	34475000	0.00114557	*KBTBD8*
18	26325001	26375000	0.00114534	*CNOT1*
18	26325001	26375000	0.00114534	*SNORA50A*
18	26325001	26375000	0.00114534	*SNORA50A*
26	21650001	21700000	0.00114507	*PAX2*
26	21650001	21700000	0.00114507	*SNORA70*
11	92600001	92650000	0.00114495	*DAB2IP*
25	27475001	27525000	0.00114488	*SLC5A2*
25	27475001	27525000	0.00114488	*TGFB1I1*
25	27475001	27525000	0.00114488	*ARMC5*
25	27475001	27525000	0.00114488	*ITGAD*
25	27475001	27525000	0.00114488	*COX6A2*
25	27475001	27525000	0.00114488	*RUSF1*
20	39900001	39950000	0.00114456	*ADAMTS12*
9	18500001	18550000	0.00114447	*PHIP*
26	23075001	23125000	0.0011444	*SUFU*
26	23075001	23125000	0.0011444	*ACTR1A*
26	23075001	23125000	0.0011444	*MFSD13A*
16	40050001	40100000	0.00114429	*SUCO*
12	30475001	30525000	0.00114419	*KATNAL1*
6	20700001	20750000	0.00114418	*CXXC4*
2	119775001	119825000	0.00114413	*DIS3L2*
9	41100001	41150000	0.00114413	*SESN1*
9	41100001	41150000	0.00114413	*U12*
1	70850001	70900000	0.00114403	*PCYT1A*
1	70850001	70900000	0.00114403	*DYNLT2B*
2	71600001	71650000	0.00114396	*PTPN4*
3	83100001	83150000	0.00114393	*DOCK7*
3	83100001	83150000	0.00114393	*USP1*
7	17125001	17175000	0.00114391	*MYO1F*
7	17125001	17175000	0.00114391	*ADAMTS10*
7	17125001	17175000	0.00114391	*SNORA70*
9	40725001	40775000	0.00114377	*PPIL6*
9	40725001	40775000	0.00114377	*CD164*
25	29000001	29050000	0.00114374	*CALN1*
11	61350001	61400000	0.00114364	*EHBP1*
15	52550001	52600000	0.00114364	*FCHSD2*
11	87875001	87925000	0.00114357	*YWHAQ*
1	80250001	80300000	0.00114357	*ST6GAL1*
11	87875001	87925000	0.00114357	*ADAM17*
2	44350001	44400000	0.00114345	*ARL5A*
3	94825001	94875000	0.00114344	*EPS15*
9	28750001	28800000	0.00114317	*HSF2*
9	28750001	28800000	0.00114317	*SERINC1*
26	18200001	18250000	0.00114306	*LCOR*
5	55850001	55900000	0.00114305	*PIP4K2C*
5	55850001	55900000	0.00114305	*ARHGEF25*
5	55850001	55900000	0.00114305	*B4GALNT1*
5	55850001	55900000	0.00114305	*SLC26A10*
5	55850001	55900000	0.00114305	*DTX3*
2	91275001	91325000	0.00114294	*CARF*

2	91275001	91325000	0.00114294	*NBEAL1*
10	43375001	43425000	0.00114278	*MAP4K5*
5	70575001	70625000	0.00114271	*BTBD11*
2	131575001	131625000	0.00114269	*EIF4G3*
3	9100001	9150000	0.00114257	*CD84*
17	35225001	35275000	0.00114256	*U6*
13	51875001	51925000	0.00114254	*DNAAF9*
25	41775001	41825000	0.00114239	*ADAP1*
25	41775001	41825000	0.00114239	*GET4*
25	41775001	41825000	0.00114239	*SUN1*
11	24550001	24600000	0.00114238	*EML4*
8	75425001	75475000	0.00114225	*UBAP2*
23	31225001	31275000	0.00114208	*POM121L2*
1	68000001	68050000	0.00114202	*MYLK*
5	30700001	30750000	0.00114194	*LMBR1L*
5	30700001	30750000	0.00114194	*DHH*
5	30700001	30750000	0.00114194	*TUBA1B*
5	25600001	25650000	0.00114185	*GTSF1*
26	21125001	21175000	0.00114178	*CWF19L1*
26	21125001	21175000	0.00114178	*CHUK*
26	21125001	21175000	0.00114178	*BLOC1S2*
26	21125001	21175000	0.00114178	*SNORA12*
1	84150001	84200000	0.00114173	*ATP11B*
2	47500001	47550000	0.00114135	*EPC2*
2	47500001	47550000	0.00114135	*U6*
15	30825001	30875000	0.00114119	*POU2F3*
22	6100001	6150000	0.00114101	*STT3B*
5	30250001	30300000	0.00114085	*KCNH3*
5	30250001	30300000	0.00114085	*MCRS1*
10	37850001	37900000	0.00114081	*SNAP23*
10	37850001	37900000	0.00114081	*HAUS2*
10	37850001	37900000	0.00114081	*LRRC57*
1	67025001	67075000	0.00114071	*PARP14*
1	67025001	67075000	0.00114071	*HSPBAP1*
5	28300001	28350000	0.00114034	*SLC4A8*
22	42400001	42450000	0.00114033	*CFAP20DC*
10	72975001	73025000	0.00114018	*MNAT1*
23	9650001	9700000	0.00114	*FKBP5*
4	56200001	56250000	0.00113986	*DOCK4*
14	29350001	29400000	0.00113971	*CYP7B1*
7	12725001	12775000	0.00113967	*HOOK2*
7	12725001	12775000	0.00113967	*RNASEH2A*
7	12725001	12775000	0.00113967	*BEST2*
7	12725001	12775000	0.00113967	*GET3*
7	12725001	12775000	0.00113967	*PRDX2*
7	12725001	12775000	0.00113967	*JUNB*
8	79225001	79275000	0.00113965	*NAA35*
8	79225001	79275000	0.00113965	*GOLM1*
4	118200001	118250000	0.00113961	*LMBR1*
15	29850001	29900000	0.00113955	*CBL*
15	29850001	29900000	0.00113955	*MCAM*
26	15025001	15075000	0.00113949	*FRA10AC1*

15	35275001	35325000	0.00113917	*PIK3C2A*
1	70250001	70300000	0.00113913	*LRCH3*
11	72275001	72325000	0.00113911	*GTF3C2*
11	72275001	72325000	0.00113911	*PPM1G*
11	72275001	72325000	0.00113911	*SNX17*
11	72275001	72325000	0.00113911	*EIF2B4*
11	72275001	72325000	0.00113911	*ZNF513*
11	72275001	72325000	0.00113911	*MPV17*
1	69950001	70000000	0.00113905	*OSBPL11*
10	36275001	36325000	0.00113899	*RAD51*
10	36275001	36325000	0.00113899	*RMDN3*
10	36275001	36325000	0.00113899	*DNAJC17*
10	36275001	36325000	0.00113899	*GCHFR*
10	36275001	36325000	0.00113899	*C10H15orf62*
7	57650001	57700000	0.00113895	*TCERG1*
11	64925001	64975000	0.00113895	*MEIS1*
21	22625001	22675000	0.00113888	*PDE8A*
21	22625001	22675000	0.00113888	*U5*
13	52250001	52300000	0.00113884	*PTPRA*
19	23100001	23150000	0.00113882	*SMG6*
19	23100001	23150000	0.00113882	*U6*
2	37100001	37150000	0.00113873	*WDSUB1*
2	37100001	37150000	0.00113873	*TANC1*
22	17125001	17175000	0.00113863	*SETD5*
2	125975001	126025000	0.00113858	*WDTC1*
17	62125001	62175000	0.00113845	*TMEM116*
17	62125001	62175000	0.00113845	*ERP29*
17	62125001	62175000	0.00113845	*NAA25*
17	62125001	62175000	0.00113845	*U6*
28	17875001	17925000	0.00113802	*ARID5B*
8	22075001	22125000	0.001138	*CDKN2A*
8	22075001	22125000	0.001138	*CDKN2B*
19	20600001	20650000	0.00113792	*NUFIP2*
19	20600001	20650000	0.00113792	*U6*
7	52550001	52600000	0.00113784	*PCDHGC3*
7	52550001	52600000	0.00113784	*PCDHGC4*
11	68425001	68475000	0.00113783	*PCBP1*
11	68425001	68475000	0.00113783	*C11H2orf42*
19	43275001	43325000	0.00113783	*U2*
19	43275001	43325000	0.00113783	*U2*
19	43275001	43325000	0.00113783	*U2*
19	43275001	43325000	0.00113783	*U2*
19	43275001	43325000	0.00113783	*U2*
13	54050001	54100000	0.00113773	*RTEL1*
13	54050001	54100000	0.00113773	*STMN3*
13	54050001	54100000	0.00113773	*ARFRP1*
13	54050001	54100000	0.00113773	*TNFRSF6B*
4	102775001	102825000	0.00113763	*UBN2*
27	14125001	14175000	0.00113756	*WWC2*
4	69250001	69300000	0.00113755	*SKAP2*
3	102300001	102350000	0.00113735	*ST3GAL3*
3	102300001	102350000	0.00113735	*KDM4A*

2	91025001	91075000	0.00113734	*FAM117B*
15	66750001	66800000	0.00113722	*PRR5L*
15	66750001	66800000	0.00113722	*COMMD9*
5	22000001	22050000	0.00113716	*BTG1*
6	116750001	116800000	0.00113675	*FGFR3*
6	116750001	116800000	0.00113675	*LETM1*
9	74375001	74425000	0.00113667	*MAP7*
5	62725001	62775000	0.00113649	*IKBIP*
5	62725001	62775000	0.00113649	*SLC25A3*
5	62725001	62775000	0.00113649	*SNORA53*
2	67600001	67650000	0.00113637	*DPP10*
3	89500001	89550000	0.00113597	*PRKAA2*
5	95450001	95500000	0.00113582	*ATF7IP*
16	44825001	44875000	0.00113581	*RERE*
2	126450001	126500000	0.00113574	*ARID1A*
22	45350001	45400000	0.00113548	*ERC2*
17	58775001	58825000	0.00113522	*MED13L*
7	57450001	57500000	0.00113497	*RBM27*
13	38050001	38100000	0.00113482	*BANF2*
4	46250001	46300000	0.00113464	*KMT2E*
4	46250001	46300000	0.00113464	*SRPK2*
11	73625001	73675000	0.00113443	*ASXL2*
21	22675001	22725000	0.00113437	*PDE8A*
25	35900001	35950000	0.00113425	*TFR2*
25	35900001	35950000	0.00113425	*ACTL6B*
25	35900001	35950000	0.00113425	*GIGYF1*
25	35900001	35950000	0.00113425	*GNB2*
25	35900001	35950000	0.00113425	*MOSPD3*
3	95550001	95600000	0.00113423	*FAF1*
23	9600001	9650000	0.00113418	*FKBP5*
10	78900001	78950000	0.00113416	*GPHN*
26	43500001	43550000	0.00113407	*CPXM2*
3	100950001	101000000	0.00113404	*ZSWIM5*
9	103950001	104000000	0.00113375	*FAM120B*
5	42925001	42975000	0.00113373	*PTPRB*
3	99775001	99825000	0.00113335	*LRRC41*
3	99775001	99825000	0.00113335	*UQCRH*
3	99775001	99825000	0.00113335	*RAD54L*
5	56850001	56900000	0.00113331	*RBMS2*
5	56850001	56900000	0.00113331	*SPRYD4*
8	225001	275000	0.00113292	*MFSD14B*
7	5850001	5900000	0.00113276	*MYO9B*
7	5850001	5900000	0.00113276	*NR2F6*
7	5850001	5900000	0.00113276	*USHBP1*
7	5850001	5900000	0.00113276	*USE1*
7	5850001	5900000	0.00113276	*OCEL1*
14	62025001	62075000	0.00113269	*UBR5*
15	52900001	52950000	0.00113264	*FAM168A*
15	52900001	52950000	0.00113264	*U6*
10	67500001	67550000	0.00113263	*SOCS4*
10	67500001	67550000	0.00113263	*MAPK1IP1L*
10	67500001	67550000	0.00113263	*WDHD1*

8	48175001	48225000	0.00113256	*C8H9orf85*
1	44650001	44700000	0.00113252	*TOMM70*
1	44650001	44700000	0.00113252	*LNP1*
2	111925001	111975000	0.00113245	*AP1S3*
22	11600001	11650000	0.00113243	*OXSR1*
22	11600001	11650000	0.00113243	*ACAA1*
22	11600001	11650000	0.00113243	*MYD88*
1	64975001	65025000	0.00113241	*GPR156*
5	119475001	119525000	0.0011324	*MOV10L1*
25	34900001	34950000	0.00113223	*CUX1*
6	116800001	116850000	0.00113215	*TACC3*
6	116800001	116850000	0.00113215	*SLBP*
6	116800001	116850000	0.00113215	*TMEM129*
7	16700001	16750000	0.00113187	*ELAVL1*
7	62725001	62775000	0.00113177	*FAT2*
5	25200001	25250000	0.00113172	*MUCL1*
2	125800001	125850000	0.0011317	*WASF2*
3	48300001	48350000	0.00113163	*TLCD4*
21	21575001	21625000	0.00113163	*SEMA4B*
21	21575001	21625000	0.00113163	*CIB1*
21	21575001	21625000	0.00113163	*GDPGP1*
11	86575001	86625000	0.00113157	*ROCK2*
11	86575001	86625000	0.00113157	*SLC66A3*
19	12550001	12600000	0.00113106	*USP32*
17	67600001	67650000	0.0011309	*TTC28*
13	36375001	36425000	0.00113067	*MPP7*
24	49200001	49250000	0.00113054	*LIPG*
12	36275001	36325000	0.00113035	*PSPC1*
1	126550001	126600000	0.0011302	*ATR*
1	126550001	126600000	0.0011302	*XRN1*
13	52200001	52250000	0.00113018	*PTPRA*
3	63075001	63125000	0.00113001	*ADGRL2*
7	12750001	12800000	0.00112993	*GET3*
7	12750001	12800000	0.00112993	*TNPO2*
7	12750001	12800000	0.00112993	*HOOK2*
7	12750001	12800000	0.00112993	*BEST2*
7	12750001	12800000	0.00112993	*TRIR*
7	12750001	12800000	0.00112993	*SNORD41*
9	65550001	65600000	0.00112986	*RIPPLY2*
10	69750001	69800000	0.00112983	*NAA30*
5	65100001	65150000	0.00112983	*SLC5A8*
18	36075001	36125000	0.00112959	*CDH1*
18	36075001	36125000	0.00112959	*TANGO6*
25	18675001	18725000	0.00112948	*LYRM1*
25	18675001	18725000	0.00112948	*DCUN1D3*
25	18675001	18725000	0.00112948	*DNAH3*
25	25925001	25975000	0.00112945	*ATP2A1*
25	25925001	25975000	0.00112945	*SH2B1*
25	25925001	25975000	0.00112945	*RABEP2*
25	25925001	25975000	0.00112945	*TUFM*
25	25925001	25975000	0.00112945	*ATXN2L*
15	36125001	36175000	0.00112938	*SOX6*

19	35525001	35575000	0.00112936	*MBTD1*
3	19725001	19775000	0.00112923	*PRUNE1*
3	19725001	19775000	0.00112923	*MINDY1*
3	19725001	19775000	0.00112923	*BNIPL*
3	19725001	19775000	0.00112923	*CDC42SE1*
3	19725001	19775000	0.00112923	*C3H1orf56*
3	19725001	19775000	0.00112923	*MLLT11*
22	48625001	48675000	0.00112919	*WDR82*
22	48625001	48675000	0.00112919	*TWF2*
22	48625001	48675000	0.00112919	*PPM1M*
22	48625001	48675000	0.00112919	*MIRLET7G*
11	14350001	14400000	0.00112913	*SRD5A2*
10	57575001	57625000	0.00112909	*ONECUT1*
7	93975001	94025000	0.00112902	*SLF1*
3	110375001	110425000	0.00112894	*ZMYM4*
3	53275001	53325000	0.00112889	*LRRC8D*
26	23700001	23750000	0.00112877	*CNNM2*
26	23700001	23750000	0.00112877	*NT5C2*
2	106700001	106750000	0.00112871	*CYP27A1*
2	106700001	106750000	0.00112871	*TTLL4*
11	94400001	94450000	0.00112866	*STRBP*
4	61250001	61300000	0.00112858	*SEPTIN7*
4	61250001	61300000	0.00112858	*U6*
7	58475001	58525000	0.0011285	*STK32A*
8	75925001	75975000	0.0011283	*CNTFR*
11	99200001	99250000	0.00112825	*ZER1*
11	99200001	99250000	0.00112825	*PKN3*
11	99200001	99250000	0.00112825	*SET*
11	99200001	99250000	0.00112825	*ZDHHC12*
5	56525001	56575000	0.00112818	*SDR9C7*
5	56525001	56575000	0.00112818	*RDH16*
16	63900001	63950000	0.00112813	*DHX9*
16	63900001	63950000	0.00112813	*SHCBP1L*
15	52225001	52275000	0.00112802	*ARAP1*
15	52225001	52275000	0.00112802	*PDE2A*
3	50350001	50400000	0.001128	*MTF2*
1	82975001	83025000	0.00112788	*DVL3*
1	82975001	83025000	0.00112788	*EIF2B5*
1	82975001	83025000	0.00112788	*AP2M1*
11	66650001	66700000	0.00112765	*PPP3R1*
11	64800001	64850000	0.0011276	*MEIS1*
11	95725001	95775000	0.00112751	*NR6A1*
22	13725001	13775000	0.00112744	*U6*
9	34325001	34375000	0.00112735	*DSE*
11	38050001	38100000	0.00112728	*CCDC88A*
17	35300001	35350000	0.00112717	*ADAD1*
21	1250001	1300000	0.00112708	*NDN*
10	5075001	5125000	0.0011269	*SIMC1*
2	44575001	44625000	0.00112659	*NEB*
2	79550001	79600000	0.00112657	*STAT4*
25	13700001	13750000	0.00112657	*PDXDC1*
25	13700001	13750000	0.00112657	*NTAN1*

25	13700001	13750000	0.00112657	*RRN3*
2	79550001	79600000	0.00112657	*STAT1*
3	110000001	110050000	0.00112656	*CLSPN*
8	38250001	38300000	0.00112636	*GLDC*
22	50150001	50200000	0.00112633	*SEMA3F*
22	50150001	50200000	0.00112633	*GNAT1*
25	36825001	36875000	0.00112623	*ZNF655*
25	36825001	36875000	0.00112623	*ZNF789*
25	36825001	36875000	0.00112623	*FAM200A*
17	61775001	61825000	0.00112619	*PTPN11*
17	61775001	61825000	0.00112619	*U6*
15	74300001	74350000	0.00112615	*EXT2*
7	46075001	46125000	0.00112606	*JADE2*
18	34475001	34525000	0.00112601	*TERB1*
18	34475001	34525000	0.00112601	*NAE1*
18	34475001	34525000	0.00112601	*DYNC1LI2*
5	7575001	7625000	0.00112593	*NAV3*
9	73975001	74025000	0.00112585	*PDE7B*
8	76950001	77000000	0.00112558	*GKAP1*
2	7950001	8000000	0.00112542	*U1*
2	7950001	8000000	0.00112542	*U6*
25	35000001	35050000	0.00112541	*CUX1*
10	21925001	21975000	0.00112531	*RBM23*
10	21925001	21975000	0.00112531	*MMP14*
10	21925001	21975000	0.00112531	*LRP10*
10	21925001	21975000	0.00112531	*REM2*
23	18775001	18825000	0.00112502	*RUNX2*
1	67975001	68025000	0.00112502	*MYLK*
15	29925001	29975000	0.00112499	*USP2*
15	29925001	29975000	0.00112499	*MFRP*
2	86050001	86100000	0.00112489	*MOB4*
16	60150001	60200000	0.00112481	*RALGPS2*
11	43275001	43325000	0.00112476	*BCL11A*
21	22350001	22400000	0.0011247	*SEC11A*
21	22350001	22400000	0.0011247	*ZNF592*
15	36150001	36200000	0.00112468	*SOX6*
2	90700001	90750000	0.00112458	*KIAA2012*
2	90700001	90750000	0.00112458	*SUMO1*
9	60050001	60100000	0.00112443	*BACH2*
4	94400001	94450000	0.00112395	*COPG2*
4	94400001	94450000	0.00112395	*TSGA13*
12	20800001	20850000	0.00112382	*INTS6*
12	20800001	20850000	0.00112382	*SERPINE3*
2	37075001	37125000	0.00112368	*WDSUB1*
8	82125001	82175000	0.00112338	*PTCH1*
5	34200001	34250000	0.00112329	*SCAF11*
21	68000001	68050000	0.00112325	*MARK3*
6	60650001	60700000	0.0011232	*PHOX2B*
21	45850001	45900000	0.00112308	*RALGAPA1*
19	33800001	33850000	0.00112291	*ULK2*
3	52500001	52550000	0.00112274	*BARHL2*
2	90725001	90775000	0.00112265	*SUMO1*

2	90725001	90775000	0.00112265	*KIAA2012*
3	94850001	94900000	0.00112258	*EPS15*
13	42950001	43000000	0.00112238	*TASOR2*
13	42950001	43000000	0.00112238	*GDI2*
7	51200001	51250000	0.00112237	*NRG2*
18	22075001	22125000	0.00112225	*FTO*
7	43800001	43850000	0.00112216	*DAZAP1*
7	43800001	43850000	0.00112216	*NDUFS7*
7	43800001	43850000	0.00112216	*PWWP3A*
7	43800001	43850000	0.00112216	*GAMT*
7	43800001	43850000	0.00112216	*RPS15*
17	54550001	54600000	0.00112202	*PPP1CC*
10	54275001	54325000	0.00112195	*RFX7*
3	57675001	57725000	0.00112192	*ODF2L*
13	20300001	20350000	0.0011218	*ARL5B*
13	75050001	75100000	0.00112163	*SLC35C2*
13	75050001	75100000	0.00112163	*ELMO2*
9	81375001	81425000	0.00112157	*PLAGL1*
4	93875001	93925000	0.00112149	*KLHDC10*
20	70775001	70825000	0.0011213	*IRX4*
27	21400001	21450000	0.00112109	*TUSC3*
13	20275001	20325000	0.00112099	*ARL5B*
17	52875001	52925000	0.00112098	*KNTC1*
10	17600001	17650000	0.00112098	*UACA*
17	52875001	52925000	0.00112098	*RSRC2*
20	37275001	37325000	0.00112092	*NIPBL*
15	76075001	76125000	0.00112083	*PHF21A*
14	34075001	34125000	0.00112074	*NCOA2*
6	116675001	116725000	0.00112074	*NSD2*
14	34075001	34125000	0.00112074	*U6*
12	35375001	35425000	0.0011207	*FGF9*
5	69950001	70000000	0.00112061	*RFX4*
3	20225001	20275000	0.00112049	*RPRD2*
20	23200001	23250000	0.00112046	*IL6ST*
10	37825001	37875000	0.0011203	*SNAP23*
10	37825001	37875000	0.0011203	*LRRC57*
25	22825001	22875000	0.00112024	*ZKSCAN2*
25	33675001	33725000	0.00112018	*BAZ1B*
13	22850001	22900000	0.00112016	*MLLT10*
1	45075001	45125000	0.00111999	*ADGRG7*
26	22525001	22575000	0.00111981	*OGA*
26	22525001	22575000	0.00111981	*KCNIP2*
26	22525001	22575000	0.00111981	*ARMH3*
29	43775001	43825000	0.00111951	*PCNX3*
29	43775001	43825000	0.00111951	*MAP3K11*
29	43775001	43825000	0.00111951	*SIPA1*
29	43775001	43825000	0.00111951	*KCNK7*
19	27700001	27750000	0.00111909	*ALOX12B*
19	27700001	27750000	0.00111909	*ALOXE3*
19	27700001	27750000	0.00111909	*ALOX15B*
16	71200001	71250000	0.00111908	*PPP2R5A*
16	71200001	71250000	0.00111908	*U6*

11	62375001	62425000	0.00111907	*VPS54*
9	43825001	43875000	0.00111902	*PRDM1*
16	28550001	28600000	0.00111876	*ENAH*
9	103500001	103550000	0.00111876	*DYNLT2*
9	103500001	103550000	0.00111876	*PHF10*
9	103500001	103550000	0.00111876	*ERMARD*
9	75100001	75150000	0.00111862	*IFNGR1*
7	61025001	61075000	0.00111854	*PPARGC1B*
13	37950001	38000000	0.00111853	*DSTN*
13	37950001	38000000	0.00111853	*RRBP1*
16	61425001	61475000	0.00111848	*LHX4*
19	10700001	10750000	0.00111841	*VMP1*
28	17725001	17775000	0.00111806	*CABCOCO1*
13	62500001	62550000	0.00111779	*BPIFA2A*
28	13750001	13800000	0.00111773	*FXYD4*
7	47500001	47550000	0.00111749	*TGFBI*
7	47500001	47550000	0.00111749	*Vault*
10	61050001	61100000	0.00111743	*GALK2*
10	61050001	61100000	0.00111743	*COPS2*
21	2075001	2125000	0.00111732	*SNORD116*
21	2075001	2125000	0.00111732	*SNORD116*
21	2075001	2125000	0.00111732	*SNORD116*
21	2075001	2125000	0.00111732	*SNORD116*
21	2075001	2125000	0.00111732	*SNORD116*
21	2075001	2125000	0.00111732	*SNORD116*
21	2075001	2125000	0.00111732	*SNORD116*
15	55825001	55875000	0.00111724	*EMSY*
15	55825001	55875000	0.00111724	*GVQW3*
19	34975001	35025000	0.00111717	*MPRIP*
16	44575001	44625000	0.00111716	*U6*
16	42475001	42525000	0.00111703	*MTOR*
16	42475001	42525000	0.00111703	*EXOSC10*
7	19675001	19725000	0.00111688	*CHAF1A*
7	19675001	19725000	0.00111688	*SH3GL1*
26	14300001	14350000	0.00111665	*EXOC6*
4	94025001	94075000	0.00111649	*CPA4*
4	94025001	94075000	0.00111649	*CPA5*
10	61000001	61050000	0.00111643	*GALK2*
20	23625001	23675000	0.00111638	*PLPP1*
1	81075001	81125000	0.00111615	*DGKG*
4	32075001	32125000	0.00111609	*IGF2BP3*
4	32075001	32125000	0.00111609	*TRA2A*
14	14950001	15000000	0.00111605	*NSMCE2*
2	85450001	85500000	0.00111593	*PGAP1*
10	13350001	13400000	0.00111574	*ZWILCH*
10	13350001	13400000	0.00111574	*RPL4*
10	13350001	13400000	0.00111574	*SNAPC5*
10	13350001	13400000	0.00111574	*MAP2K1*
10	13350001	13400000	0.00111574	*SNORD16*
10	13350001	13400000	0.00111574	*SNORD18*
10	13350001	13400000	0.00111574	*SNORD18*
10	13350001	13400000	0.00111574	*SNORD18*

2	119900001	119950000	0.0011156	*DIS3L2*
27	33400001	33450000	0.0011155	*NSD3*
27	33400001	33450000	0.0011155	*DDHD2*
27	33400001	33450000	0.0011155	*PLPP5*
18	19000001	19050000	0.00111543	*NKD1*
14	64675001	64725000	0.0011153	*VPS13B*
13	33525001	33575000	0.0011153	*ARHGAP12*
2	120325001	120375000	0.00111528	*PHC2*
3	32500001	32550000	0.00111517	*CD53*
7	82050001	82100000	0.00111515	*ATG10*
3	78350001	78400000	0.00111501	*MIER1*
5	77075001	77125000	0.00111489	*PKP2*
7	50475001	50525000	0.00111484	*SIL1*
23	41675001	41725000	0.00111475	*JARID2*
22	7775001	7825000	0.00111464	*CLASP2*
20	66700001	66750000	0.00111457	*UBE2QL1*
3	67000001	67050000	0.0011145	*ZZZ3*
13	46850001	46900000	0.00111433	*ZMYND11*
20	41050001	41100000	0.00111427	*SUB1*
15	37800001	37850000	0.0011135	*CALCB*
5	95375001	95425000	0.00111346	*ATF7IP*
5	95375001	95425000	0.00111346	*U6*
19	26625001	26675000	0.00111326	*PELP1*
19	26625001	26675000	0.00111326	*ARRB2*
2	53025001	53075000	0.0011132	*ARHGAP15*
25	27500001	27550000	0.00111306	*RUSF1*
25	27500001	27550000	0.00111306	*SLC5A2*
25	27500001	27550000	0.00111306	*TGFB1I1*
25	27500001	27550000	0.00111306	*AHSP*
25	27500001	27550000	0.00111306	*ARMC5*
2	20700001	20750000	0.00111305	*HOXD3*
2	20700001	20750000	0.00111305	*HOXD1*
2	43525001	43575000	0.00111282	*FMNL2*
2	43525001	43575000	0.00111282	*PRPF40A*
7	12550001	12600000	0.00111265	*NFIX*
7	43250001	43300000	0.00111249	*RNF126*
7	43250001	43300000	0.00111249	*FSTL3*
7	43250001	43300000	0.00111249	*PRSS57*
7	43250001	43300000	0.00111249	*FGF22*
7	43250001	43300000	0.00111249	*PALM*
5	29700001	29750000	0.00111247	*LIMA1*
5	29700001	29750000	0.00111247	*CERS5*
8	38350001	38400000	0.00111242	*UHRF2*
17	53425001	53475000	0.00111219	*PSMD9*
17	53425001	53475000	0.00111219	*HPD*
5	71400001	71450000	0.00111215	*TIMP3*
7	19625001	19675000	0.00111214	*HDGFL2*
7	19625001	19675000	0.00111214	*UBXN6*
7	19625001	19675000	0.00111214	*PLIN4*
19	45525001	45575000	0.00111207	*NSF*
4	56900001	56950000	0.00111167	*IMMP2L*
11	15500001	15550000	0.00111157	*LTBP1*

7	65525001	65575000	0.00111128	*GALNT10*
14	600001	650000	0.00111125	*HSF1*
14	600001	650000	0.00111125	*BOP1*
14	600001	650000	0.00111125	*DGAT1*
14	600001	650000	0.00111125	*SCX*
14	600001	650000	0.00111125	*SCRT1*
20	14175001	14225000	0.00111124	*ADAMTS6*
9	73425001	73475000	0.00111107	*AHI1*
11	18900001	18950000	0.00111092	*CRIM1*
15	39200001	39250000	0.0011109	*BTBD10*
13	60675001	60725000	0.00111072	*ZCCHC3*
13	60675001	60725000	0.00111072	*NRSN2*
13	60675001	60725000	0.00111072	*SOX12*
13	60675001	60725000	0.00111072	*C13H20orf96*
11	99275001	99325000	0.00111063	*TBC1D13*
11	99275001	99325000	0.00111063	*KYAT1*
11	99275001	99325000	0.00111063	*ENDOG*
11	99275001	99325000	0.00111063	*SPOUT1*
9	42075001	42125000	0.00111049	*OSTM1*
9	65325001	65375000	0.00111044	*MRAP2*
9	73850001	73900000	0.00111028	*PDE7B*
5	57525001	57575000	0.00111019	*ITGA7*
5	57525001	57575000	0.00111019	*CD63*
5	57525001	57575000	0.00111019	*RDH5*
5	57525001	57575000	0.00111019	*BLOC1S1*
5	57525001	57575000	0.00111019	*METTL7B*
13	22925001	22975000	0.00110991	*MLLT10*
13	22925001	22975000	0.00110991	*DNAJC1*
23	52050001	52100000	0.00110982	*EXOC2*
14	47525001	47575000	0.0011098	*UTP23*
14	47525001	47575000	0.0011098	*EIF3H*
21	26050001	26100000	0.00110971	*ZFAND6*
3	99700001	99750000	0.00110959	*FAAH*
29	20275001	20325000	0.00110954	*LUZP2*
16	77050001	77100000	0.00110927	*NEK7*
19	43075001	43125000	0.00110921	*BRCA1*
10	53350001	53400000	0.00110871	*U6*
11	66575001	66625000	0.00110869	*PPP3R1*
11	66575001	66625000	0.00110869	*DNAAF10*
11	66575001	66625000	0.00110869	*PNO1*
14	27900001	27950000	0.00110866	*NKAIN3*
10	19375001	19425000	0.00110853	*ARIH1*
22	55975001	56025000	0.00110829	*TMCC1*
22	55975001	56025000	0.00110829	*U6*
22	55975001	56025000	0.00110829	*U6*
17	13550001	13600000	0.00110828	*HHIP*
18	39425001	39475000	0.00110824	*TAT*
18	39425001	39475000	0.00110824	*MARVELD3*
18	39425001	39475000	0.00110824	*U6*
10	46175001	46225000	0.00110813	*HERC1*
4	80975001	81025000	0.00110792	*CDK13*
2	91225001	91275000	0.00110764	*CARF*

2	91225001	91275000	0.00110764	*WDR12*
13	54850001	54900000	0.0011076	*LAMA5*
13	54850001	54900000	0.0011076	*CABLES2*
13	54850001	54900000	0.0011076	*RBBP8NL*
13	54850001	54900000	0.0011076	*RPS21*
22	50325001	50375000	0.00110745	*MST1R*
22	50325001	50375000	0.00110745	*MON1A*
22	50325001	50375000	0.00110745	*RBM6*
1	109325001	109375000	0.00110727	*RSRC1*
1	29300001	29350000	0.0011072	*GBE1*
2	22750001	22800000	0.00110711	*SP3*
9	41725001	41775000	0.00110702	*AFG1L*
19	26800001	26850000	0.00110688	*BCL6B*
19	26800001	26850000	0.00110688	*C19H17orf49*
19	26800001	26850000	0.00110688	*CLEC10A*
19	26800001	26850000	0.00110688	*SLC16A13*
19	26800001	26850000	0.00110688	*SLC16A11*
19	26800001	26850000	0.00110688	*RNASEK*
19	26800001	26850000	0.00110688	*ALOX12*
1	82800001	82850000	0.0011068	*CLCN2*
1	82800001	82850000	0.0011068	*CHRD*
1	82800001	82850000	0.0011068	*POLR2H*
1	82800001	82850000	0.0011068	*FAM131A*
1	82800001	82850000	0.0011068	*THPO*
3	50100001	50150000	0.00110677	*DR1*
22	45450001	45500000	0.00110671	*ERC2*
21	22375001	22425000	0.00110665	*ZNF592*
13	51850001	51900000	0.00110657	*DNAAF9*
1	76700001	76750000	0.00110649	*IL1RAP*
7	70575001	70625000	0.00110648	*EBF1*
7	18925001	18975000	0.00110612	*PTPRS*
7	47600001	47650000	0.00110602	*SMAD5*
7	47600001	47650000	0.00110602	*SMIM32*
7	18675001	18725000	0.00110596	*SAFB2*
2	89575001	89625000	0.00110595	*FAM126B*
2	89575001	89625000	0.00110595	*ORC2*
3	49050001	49100000	0.00110586	*ABCD3*
13	72075001	72125000	0.00110577	*L3MBTL1*
13	72075001	72125000	0.00110577	*SRSF6*
8	39175001	39225000	0.00110567	*PDCD1LG2*
2	121300001	121350000	0.0011055	*EIF3I*
2	121300001	121350000	0.0011055	*TMEM234*
2	121300001	121350000	0.0011055	*DCDC2B*
2	121300001	121350000	0.0011055	*CCDC28B*
2	121300001	121350000	0.0011055	*IQCC*
11	74825001	74875000	0.00110542	*ITSN2*
19	34625001	34675000	0.00110541	*RAI1*
19	34625001	34675000	0.00110541	*SREBF1*
19	34625001	34675000	0.00110541	*TOM1L2*
19	39850001	39900000	0.00110515	*CDK12*
19	39850001	39900000	0.00110515	*MED1*
14	64775001	64825000	0.00110493	*VPS13B*

5	30675001	30725000	0.00110489	*LMBR1L*
5	30675001	30725000	0.00110489	*TUBA1B*
7	50575001	50625000	0.00110468	*MATR3*
7	50575001	50625000	0.00110468	*SNORA74*
7	50575001	50625000	0.00110468	*SNORA74*
25	36800001	36850000	0.00110454	*ZNF655*
25	36800001	36850000	0.00110454	*ZSCAN25*
25	36800001	36850000	0.00110454	*TMEM225B*
9	41850001	41900000	0.00110437	*SNX3*
9	41850001	41900000	0.00110437	*AFG1L*
28	25050001	25100000	0.00110436	*CCAR1*
5	25625001	25675000	0.00110416	*ITGA5*
5	25625001	25675000	0.00110416	*GTSF1*
20	23500001	23550000	0.00110402	*SLC38A9*
3	34250001	34300000	0.00110397	*CFAP276*
3	34250001	34300000	0.00110397	*TMEM167B*
3	34250001	34300000	0.00110397	*SCARNA2*
11	92625001	92675000	0.0011039	*DAB2IP*
19	34650001	34700000	0.00110383	*RAI1*
9	48225001	48275000	0.00110373	*GRIK2*
15	53025001	53075000	0.00110371	*FAM168A*
1	71000001	71050000	0.00110346	*RNF168*
1	71000001	71050000	0.00110346	*SMCO1*
8	25700001	25750000	0.00110316	*ADAMTSL1*
24	43400001	43450000	0.0011031	*LDLRAD4*
13	27975001	28025000	0.00110306	*PHYH*
13	27975001	28025000	0.00110306	*SEPHS1*
1	69675001	69725000	0.00110303	*ZNF148*
27	42100001	42150000	0.001103	*UBE2E1*
3	95525001	95575000	0.00110294	*FAF1*
9	33850001	33900000	0.00110239	*KPNA5*
9	33850001	33900000	0.00110239	*FAM162B*
23	9350001	9400000	0.00110237	*PPARD*
10	16950001	17000000	0.00110236	*TLE3*
26	15550001	15600000	0.00110233	*PLCE1*
7	44525001	44575000	0.00110222	*AFF4*
7	44525001	44575000	0.00110222	*ZCCHC10*
7	44525001	44575000	0.00110222	*U6*
21	57925001	57975000	0.00110211	*BTBD7*
18	55075001	55125000	0.00110195	*GRIN2D*
18	55075001	55125000	0.00110195	*KDELR1*
18	55075001	55125000	0.00110195	*SYNGR4*
18	55075001	55125000	0.00110195	*GRWD1*
14	62050001	62100000	0.0011018	*UBR5*
7	49325001	49375000	0.00110168	*MYOT*
7	49325001	49375000	0.00110168	*PKD2L2*
7	43675001	43725000	0.00110138	*MIDN*
7	43675001	43725000	0.00110138	*CIRBP*
7	43675001	43725000	0.00110138	*CBARP*
7	43675001	43725000	0.00110138	*FAM174C*
7	43675001	43725000	0.00110138	*EFNA2*
7	43675001	43725000	0.00110138	*ATP5F1D*

3	51625001	51675000	0.00110133	*TGFBR3*
7	70650001	70700000	0.00110124	*EBF1*
1	111325001	111375000	0.00110123	*KCNAB1*
2	112650001	112700000	0.00110105	*CUL3*
1	114550001	114600000	0.00110103	*RAP2B*
17	62400001	62450000	0.00110101	*BICDL1*
23	2825001	2875000	0.0011008	*PRIM2*
8	38925001	38975000	0.00110067	*RIC1*
8	38925001	38975000	0.00110067	*ERMP1*
14	8475001	8525000	0.00110057	*PHF20L1*
17	35250001	35300000	0.00110035	*ADAD1*
17	35250001	35300000	0.00110035	*U6*
1	1875001	1925000	0.00110031	*ITSN1*
1	1875001	1925000	0.00110031	*CRYZL1*
12	29200001	29250000	0.00110025	*RXFP2*
6	116775001	116825000	0.00110022	*FGFR3*
24	35000001	35050000	0.00110021	*ROCK1*
18	24400001	24450000	0.00109993	*GNAO1*
24	42775001	42825000	0.00109962	*AFG3L2*
24	42775001	42825000	0.00109962	*CIDEA*
24	42775001	42825000	0.00109962	*TUBB6*
11	94575001	94625000	0.00109947	*DENND1A*
29	12475001	12525000	0.00109934	*PCF11*
3	104975001	105025000	0.00109911	*SCMH1*
3	104975001	105025000	0.00109911	*U6*
4	43625001	43675000	0.00109869	*PTPN12*
13	74750001	74800000	0.00109847	*SLC12A5*
13	74750001	74800000	0.00109847	*MMP9*
13	74750001	74800000	0.00109847	*NCOA5*
6	58675001	58725000	0.00109838	*SMIM14*
11	62400001	62450000	0.0010983	*VPS54*
3	87225001	87275000	0.00109828	*JUN*
16	50850001	50900000	0.00109821	*GNB1*
10	61250001	61300000	0.00109807	*SHC4*
3	79250001	79300000	0.00109754	*PDE4B*
3	79250001	79300000	0.00109754	*MGC137454*
12	36025001	36075000	0.00109722	*GJB6*
9	74175001	74225000	0.00109712	*PDE7B*
2	91550001	91600000	0.0010971	*ABI2*
13	76025001	76075000	0.00109677	*NCOA3*
13	60475001	60525000	0.00109661	*CSNK2A1*
21	41175001	41225000	0.00109657	*G2E3*
5	29725001	29775000	0.0010965	*CERS5*
5	29725001	29775000	0.0010965	*LIMA1*
3	32400001	32450000	0.00109637	*LRIF1*
13	32675001	32725000	0.00109632	*CACNB2*
3	62950001	63000000	0.00109617	*ADGRL2*
2	53800001	53850000	0.00109595	*KYNU*
20	23775001	23825000	0.0010959	*MTREX*
12	32375001	32425000	0.00109585	*LNX2*
10	52275001	52325000	0.00109572	*ALDH1A2*
10	52275001	52325000	0.00109572	*U6*

11	99550001	99600000	0.00109559	*PTPA*
11	99550001	99600000	0.00109559	*CRAT*
11	99550001	99600000	0.00109559	*DOLPP1*
8	59325001	59375000	0.00109556	*FAM214B*
8	59325001	59375000	0.00109556	*VCP*
8	59325001	59375000	0.00109556	*PIGO*
8	59325001	59375000	0.00109556	*FANCG*
8	59325001	59375000	0.00109556	*STOML2*
8	59325001	59375000	0.00109556	*UNC13B*
25	25900001	25950000	0.00109553	*ATP2A1*
5	70225001	70275000	0.00109553	*TMEM263*
5	70225001	70275000	0.00109553	*CRY1*
25	25900001	25950000	0.00109553	*RABEP2*
25	25900001	25950000	0.00109553	*CD19*
5	70225001	70275000	0.00109553	*MTERF2*
1	81250001	81300000	0.00109538	*TRA2B*
16	51125001	51175000	0.00109524	*CCNL2*
16	51125001	51175000	0.00109524	*AURKAIP1*
16	51125001	51175000	0.00109524	*MRPL20*
16	51125001	51175000	0.00109524	*ANKRD65*
16	51125001	51175000	0.00109524	*MXRA8*
29	28300001	28350000	0.00109521	*ROBO3*
17	62375001	62425000	0.00109517	*BICDL1*
5	66700001	66750000	0.00109502	*PAH*
5	66700001	66750000	0.00109502	*ASCL1*
1	73075001	73125000	0.001095	*ATP13A3*
4	30275001	30325000	0.00109482	*SP4*
4	30275001	30325000	0.00109482	*DNAH11*
7	50600001	50650000	0.00109478	*MATR3*
7	50600001	50650000	0.00109478	*PAIP2*
7	50600001	50650000	0.00109478	*SLC23A1*
16	42125001	42175000	0.00109467	*DISP3*
7	6400001	6450000	0.00109452	*MED26*
16	51950001	52000000	0.00109447	*DNAJC16*
16	51950001	52000000	0.00109447	*AGMAT*
5	37075001	37125000	0.00109441	*ADAMTS20*
9	86850001	86900000	0.0010942	*LATS1*
9	86850001	86900000	0.0010942	*KATNA1*
8	77125001	77175000	0.00109417	*KIF27*
2	125750001	125800000	0.00109407	*AHDC1*
19	36825001	36875000	0.00109392	*KAT7*
19	36825001	36875000	0.00109392	*FAM117A*
18	38075001	38125000	0.00109391	*ZFHX3*
13	50650001	50700000	0.00109383	*HAO1*
6	72325001	72375000	0.00109373	*NOA1A*
6	72325001	72375000	0.00109373	*POLR2B*
6	72325001	72375000	0.00109373	*RESTB*
18	36925001	36975000	0.0010937	*WWP2*
18	36925001	36975000	0.0010937	*MIR140*
13	51425001	51475000	0.00109341	*HSPA12B*
13	51425001	51475000	0.00109341	*C13H20orf27*
13	51425001	51475000	0.00109341	*SPEF1*

13	54075001	54125000	0.00109318	*GMEB2*
13	54075001	54125000	0.00109318	*STMN3*
13	54075001	54125000	0.00109318	*SRMS*
13	54075001	54125000	0.00109318	*FNDC11*
13	54075001	54125000	0.00109318	*RTEL1*
18	36275001	36325000	0.00109278	*TANGO6*
18	36275001	36325000	0.00109278	*HAS3*
20	2875001	2925000	0.00109265	*RANBP17*
2	119750001	119800000	0.00109259	*DIS3L2*
10	65050001	65100000	0.00109236	*SPATA5L1*
10	65050001	65100000	0.00109236	*GATM*
19	34275001	34325000	0.0010923	*PRPSAP2*
1	109375001	109425000	0.00109223	*RSRC1*
1	109375001	109425000	0.00109223	*SHOX2*
15	76725001	76775000	0.00109221	*CKAP5*
2	36675001	36725000	0.00109219	*BAZ2B*
7	7825001	7875000	0.00109212	*BRD4*
3	95625001	95675000	0.00109209	*FAF1*
1	158300001	158350000	0.0010919	*TMEM39B*
1	158300001	158350000	0.0010919	*KHDRBS1*
7	7375001	7425000	0.00109185	*CYP4F2*
7	7750001	7800000	0.00109168	*AKAP8*
7	7750001	7800000	0.00109168	*AKAP8L*
25	3450001	3500000	0.00109162	*TFAP4*
25	3450001	3500000	0.00109162	*GLIS2*
10	82125001	82175000	0.00109159	*MED6*
10	82125001	82175000	0.00109159	*ADAM21*
22	49725001	49775000	0.00109144	*MAPKAPK3*
22	49725001	49775000	0.00109144	*HEMK1*
22	49725001	49775000	0.00109144	*CISH*
2	24775001	24825000	0.0010914	*DYNC1I2*
21	24375001	24425000	0.00109116	*ADAMTSL3*
7	18575001	18625000	0.00109112	*LONP1*
7	18575001	18625000	0.00109112	*CATSPERD*
7	18575001	18625000	0.00109112	*HSD11B1L*
7	18575001	18625000	0.00109112	*SAFB*
7	18575001	18625000	0.00109112	*MICOS13*
7	18575001	18625000	0.00109112	*RPL36*
13	60450001	60500000	0.00109098	*TCF15*
7	38975001	39025000	0.00109095	*DBN1*
7	38975001	39025000	0.00109095	*DDX41*
7	38975001	39025000	0.00109095	*DOK3*
7	38975001	39025000	0.00109095	*FAM193B*
5	36425001	36475000	0.00109086	*TMEM117*
3	20200001	20250000	0.00109085	*RPRD2*
3	20200001	20250000	0.00109085	*TARS2*
5	36225001	36275000	0.00109079	*TMEM117*
19	50925001	50975000	0.00109072	*NOTUM*
19	50925001	50975000	0.00109072	*MAFG*
19	50925001	50975000	0.00109072	*PYCR1*
19	50925001	50975000	0.00109072	*MYADML2*
11	68150001	68200000	0.00109062	*GMCL1*

11	68150001	68200000	0.00109062	*ANXA4*
23	275001	325000	0.00109057	*KHDRBS2*
9	50325001	50375000	0.00109052	*PNISR*
9	50325001	50375000	0.00109052	*USP45*
5	51200001	51250000	0.00109033	*USP15*
1	70600001	70650000	0.00109005	*TNK2*
3	33875001	33925000	0.00108987	*GNAI3*
3	33875001	33925000	0.00108987	*GPR61*
22	45375001	45425000	0.00108985	*ERC2*
5	107550001	107600000	0.00108982	*WNK1*
11	94000001	94050000	0.00108976	*RC3H2*
11	94000001	94050000	0.00108976	*SNORD90*
1	132275001	132325000	0.00108965	*SLC35G2*
1	132275001	132325000	0.00108965	*NCK1*
16	64550001	64600000	0.00108963	*SMG7*
9	73900001	73950000	0.00108958	*PDE7B*
29	41175001	41225000	0.00108948	*SLC3A2*
18	39400001	39450000	0.00108948	*PHLPP2*
18	39400001	39450000	0.00108948	*TAT*
18	39400001	39450000	0.00108948	*MARVELD3*
29	41175001	41225000	0.00108948	*CHRM1*
21	30850001	30900000	0.00108935	*IREB2*
21	30850001	30900000	0.00108935	*HYKK*
12	36250001	36300000	0.00108932	*PSPC1*
12	36250001	36300000	0.00108932	*ZMYM5*
9	73950001	74000000	0.00108905	*PDE7B*
11	72375001	72425000	0.00108899	*CAD*
11	72375001	72425000	0.00108899	*SLC30A3*
11	72375001	72425000	0.00108899	*SLC5A6*
11	72375001	72425000	0.00108899	*ATRAID*
19	50625001	50675000	0.00108865	*CCDC57*
19	50625001	50675000	0.00108865	*SLC16A3*
19	50625001	50675000	0.00108865	*CSNK1D*
23	9325001	9375000	0.00108864	*PPARD*
2	85300001	85350000	0.0010886	*CCDC150*
6	116725001	116775000	0.00108842	*LETM1*
6	116725001	116775000	0.00108842	*FGFR3*
19	27125001	27175000	0.00108828	*POLR2A*
19	27125001	27175000	0.00108828	*ZBTB4*
19	27125001	27175000	0.00108828	*CHRNB1*
19	27125001	27175000	0.00108828	*SLC35G6*
7	88375001	88425000	0.00108801	*MEF2C*
5	30275001	30325000	0.00108786	*SPATS2*
5	30275001	30325000	0.00108786	*KCNH3*
10	86100001	86150000	0.0010876	*DLST*
10	86100001	86150000	0.0010876	*PROX2*
16	39525001	39575000	0.00108753	*DNM3*
13	12225001	12275000	0.00108729	*DHTKD1*
13	12225001	12275000	0.00108729	*SEC61A2*
2	79425001	79475000	0.00108687	*GLS*
10	19350001	19400000	0.00108679	*ARIH1*
2	85350001	85400000	0.00108668	*GTF3C3*

13	51375001	51425000	0.00108666	*CDC25B*
13	51375001	51425000	0.00108666	*AP5S1*
13	51375001	51425000	0.00108666	*CENPB*
13	51375001	51425000	0.00108666	*SPEF1*
12	24900001	24950000	0.00108645	*SERTM1*
9	103975001	104025000	0.00108642	*FAM120B*
28	30750001	30800000	0.00108641	*KAT6B*
11	62350001	62400000	0.00108631	*VPS54*
19	11075001	11125000	0.00108622	*MED13*
25	41675001	41725000	0.00108617	*C25H7orf50*
25	41675001	41725000	0.00108617	*GPR146*
21	45125001	45175000	0.00108587	*BAZ1A*
10	53975001	54025000	0.00108579	*TEX9*
10	53975001	54025000	0.00108579	*MNS1*
22	18625001	18675000	0.00108576	*GRM7*
2	26625001	26675000	0.00108562	*PPIG*
2	26625001	26675000	0.00108562	*CFAP210*
3	20275001	20325000	0.00108546	*RPRD2*
3	20275001	20325000	0.00108546	*PRPF3*
2	32750001	32800000	0.00108536	*FIGN*
6	5725001	5775000	0.00108533	*PDE5A*
7	50175001	50225000	0.00108505	*CTNNA1*
7	50175001	50225000	0.00108505	*LRRTM2*
13	51325001	51375000	0.00108484	*MAVS*
18	21750001	21800000	0.00108481	*CHD9*
3	54575001	54625000	0.00108479	*GBP6*
8	61925001	61975000	0.00108473	*DCAF10*
6	72225001	72275000	0.00108469	*RESTB*
6	72225001	72275000	0.00108469	*NOA1A*
9	44925001	44975000	0.00108457	*LIN28B*
9	44925001	44975000	0.00108457	*BVES*
18	225001	275000	0.00108448	*OR4P4*
16	51425001	51475000	0.00108443	*AGRN*
16	51425001	51475000	0.00108443	*ISG15*
16	51425001	51475000	0.00108443	*HES4*
3	16500001	16550000	0.0010843	*DENND4B*
18	15125001	15175000	0.00108428	*C18H16orf87*
16	41225001	41275000	0.0010842	*VPS13D*
13	12200001	12250000	0.00108416	*SEC61A2*
13	12200001	12250000	0.00108416	*DHTKD1*
13	12200001	12250000	0.00108416	*NUDT5*
18	38325001	38375000	0.0010841	*ZFHX3*
13	36100001	36150000	0.00108392	*WAC*
3	19875001	19925000	0.00108366	*ARNT*
5	29450001	29500000	0.00108356	*LARP4*
2	115625001	115675000	0.00108353	*AGFG1*
3	53250001	53300000	0.00108352	*ZNF326*
7	53325001	53375000	0.00108324	*SPRY4*
7	22800001	22850000	0.00108318	*RAPGEF6*
7	22800001	22850000	0.00108318	*FNIP1*
17	53550001	53600000	0.00108314	*TMEM120B*
17	53550001	53600000	0.00108314	*MORN3*

8	27025001	27075000	0.00108298	*CNTLN*
2	119650001	119700000	0.00108258	*DIS3L2*
1	120875001	120925000	0.00108256	*ZIC4*
1	120875001	120925000	0.00108256	*ZIC1*
1	122325001	122375000	0.00108249	*PLOD2*
26	44450001	44500000	0.00108247	*ZRANB1*
25	18650001	18700000	0.00108233	*DCUN1D3*
13	61875001	61925000	0.00108226	*NOL4L*
27	34400001	34450000	0.00108211	*ADAM32*
7	39275001	39325000	0.00108192	*PHYKPL*
7	39275001	39325000	0.00108192	*COL23A1*
7	39275001	39325000	0.00108192	*HNRNPAB*
7	39275001	39325000	0.00108192	*U6*
7	49650001	49700000	0.00108186	*GFRA3*
11	86500001	86550000	0.00108183	*ROCK2*
17	35275001	35325000	0.00108179	*ADAD1*
10	53900001	53950000	0.00108178	*MNS1*
2	122250001	122300000	0.00108178	*FABP3*
2	122250001	122300000	0.00108178	*SERINC2*
2	122250001	122300000	0.00108178	*ZCCHC17*
13	50575001	50625000	0.00108161	*HAO1*
13	32100001	32150000	0.00108156	*STAM*
13	32100001	32150000	0.00108156	*SNORD62*
5	39375001	39425000	0.00108137	*PDZRN4*
25	41750001	41800000	0.00108134	*ADAP1*
25	41750001	41800000	0.00108134	*COX19*
25	41750001	41800000	0.00108134	*CYP2W1*
5	29150001	29200000	0.0010811	*ATF1*
5	29150001	29200000	0.0010811	*DIP2B*
5	55775001	55825000	0.00108098	*OS9*
4	95150001	95200000	0.00108072	*MKLN1*
7	50000001	50050000	0.00108052	*CTNNA1*
5	57500001	57550000	0.00108047	*GDF11*
5	57500001	57550000	0.00108047	*CD63*
5	57500001	57550000	0.00108047	*RDH5*
5	57500001	57550000	0.00108047	*BLOC1S1*
5	57500001	57550000	0.00108047	*ITGA7*
20	23750001	23800000	0.00108039	*MTREX*
1	70825001	70875000	0.00108007	*PCYT1A*
1	70825001	70875000	0.00108007	*SLC51A*
9	86625001	86675000	0.00107984	*TAB2*
3	80475001	80525000	0.00107983	*JAK1*
28	30600001	30650000	0.00107979	*KAT6B*
13	17850001	17900000	0.00107955	*ABI1*
5	54525001	54575000	0.00107885	*LRIG3*
6	58775001	58825000	0.00107866	*UBE2K*
14	50775001	50825000	0.00107851	*CSMD3*
21	26525001	26575000	0.00107848	*ABHD17C*
21	41700001	41750000	0.00107828	*HECTD1*
10	60775001	60825000	0.00107828	*FGF7*
21	56475001	56525000	0.0010782	*TC2N*
16	37725001	37775000	0.00107793	*NTMT2*

8	38075001	38125000	0.00107773	*KDM4C*
3	18950001	19000000	0.00107745	*RORC*
3	18950001	19000000	0.00107745	*TDRKH*
3	18950001	19000000	0.00107745	*LINGO4*
7	46450001	46500000	0.00107729	*PITX1*
7	46450001	46500000	0.00107729	*CATSPER3*
9	33725001	33775000	0.00107718	*RFX6*
21	6000001	6050000	0.00107716	*LINS1*
21	6000001	6050000	0.00107716	*ASB7*
26	22325001	22375000	0.00107705	*DPCD*
26	22325001	22375000	0.00107705	*FBXW4*
26	22325001	22375000	0.00107705	*POLL*
10	19000001	19050000	0.00107697	*MYO9A*
11	74800001	74850000	0.00107679	*ITSN2*
11	74800001	74850000	0.00107679	*U3*
17	62475001	62525000	0.00107653	*GCN1*
17	62475001	62525000	0.00107653	*RAB35*
16	41950001	42000000	0.00107609	*DRAXIN*
16	41950001	42000000	0.00107609	*MAD2L2*
19	26300001	26350000	0.00107597	*ZFP3*
19	26300001	26350000	0.00107597	*SCIMP*
13	29525001	29575000	0.00107554	*SUV39H2*
13	29525001	29575000	0.00107554	*DCLRE1C*
3	59525001	59575000	0.00107545	*SPATA1*
2	35175001	35225000	0.00107541	*TANK*
13	33450001	33500000	0.00107521	*ARHGAP12*
10	54725001	54775000	0.00107519	*PRTG*
10	71600001	71650000	0.00107511	*DAAM1*
7	22750001	22800000	0.00107508	*FNIP1*
7	22750001	22800000	0.00107508	*U6*
6	67775001	67825000	0.00107504	*DCUN1D4*
1	80625001	80675000	0.00107483	*DNAJB11*
1	80625001	80675000	0.00107483	*TBCCD1*
3	33950001	34000000	0.00107466	*SYPL2*
3	33950001	34000000	0.00107466	*ATXN7L2*
3	33950001	34000000	0.00107466	*CYB561D1*
6	116700001	116750000	0.00107461	*LETM1*
1	80850001	80900000	0.00107452	*DGKG*
3	20600001	20650000	0.00107447	*OTUD7B*
2	71550001	71600000	0.0010744	*PTPN4*
11	19725001	19775000	0.00107425	*PRKD3*
11	29175001	29225000	0.00107418	*SOCS5*
17	58650001	58700000	0.00107413	*MED13L*
1	64950001	65000000	0.0010741	*GPR156*
10	21600001	21650000	0.00107408	*RNF212B*
10	21600001	21650000	0.00107408	*HOMEZ*
10	21600001	21650000	0.00107408	*BCL2L2*
10	21600001	21650000	0.00107408	*PPP1R3E*
2	38725001	38775000	0.0010738	*ACVR1C*
17	29575001	29625000	0.00107369	*LARP1B*
13	47400001	47450000	0.00107343	*TMEM230*
13	47400001	47450000	0.00107343	*CDS2*

13	47400001	47450000	0.00107343	*PCNA*
1	122350001	122400000	0.00107324	*PLOD2*
3	63025001	63075000	0.00107323	*ADGRL2*
22	56025001	56075000	0.0010731	*TMCC1*
3	49950001	50000000	0.00107282	*FNBP1L*
3	63050001	63100000	0.00107267	*ADGRL2*
20	66725001	66775000	0.00107265	*UBE2QL1*
17	72900001	72950000	0.0010725	*ARVCF*
17	72900001	72950000	0.0010725	*COMT*
17	72900001	72950000	0.0010725	*TANGO2*
17	72900001	72950000	0.0010725	*TXNRD2*
14	67025001	67075000	0.00107245	*CPQ*
28	30675001	30725000	0.00107206	*KAT6B*
9	65275001	65325000	0.00107182	*MRAP2*
8	103350001	103400000	0.00107162	*COL27A1*
18	36000001	36050000	0.00107156	*CDH3*
18	36000001	36050000	0.00107156	*CDH1*
27	33375001	33425000	0.00107138	*DDHD2*
27	33375001	33425000	0.00107138	*PLPP5*
6	59450001	59500000	0.00107132	*RBM47*
19	12700001	12750000	0.00107056	*USP32*
17	58675001	58725000	0.0010702	*MED13L*
5	43150001	43200000	0.00107016	*CNOT2*
13	36075001	36125000	0.00107012	*WAC*
7	22725001	22775000	0.00107009	*FNIP1*
1	65075001	65125000	0.00107009	*LRRC58*
22	51150001	51200000	0.00107007	*IP6K2*
22	51150001	51200000	0.00107007	*PRKAR2A*
9	81300001	81350000	0.00106984	*ZC2HC1B*
9	81300001	81350000	0.00106984	*LTV1*
13	32075001	32125000	0.00106932	*STAM*
13	32075001	32125000	0.00106932	*HACD1*
12	36625001	36675000	0.00106928	*ATP12A*
5	107600001	107650000	0.00106913	*WNK1*
3	74900001	74950000	0.00106899	*ANKRD13C*
3	74900001	74950000	0.00106899	*SRSF11*
9	63850001	63900000	0.00106896	*SNX14*
8	75200001	75250000	0.00106882	*UBE2R2*
8	75200001	75250000	0.00106882	*NOL6*
8	75200001	75250000	0.00106882	*AQP3*
8	250001	300000	0.00106858	*MFSD14B*
5	36000001	36050000	0.00106848	*TMEM117*
1	120900001	120950000	0.00106814	*ZIC4*
1	120900001	120950000	0.00106814	*ZIC1*
5	36600001	36650000	0.00106809	*TMEM117*
5	30175001	30225000	0.001068	*FMNL3*
5	30175001	30225000	0.001068	*PRPF40B*
11	10125001	10175000	0.00106796	*CCDC142*
11	10125001	10175000	0.00106796	*MOGS*
11	10125001	10175000	0.00106796	*INO80B*
11	10125001	10175000	0.00106796	*MRPL53*
11	10125001	10175000	0.00106796	*WBP1*

11	10125001	10175000	0.00106796	*PCGF1*
11	10125001	10175000	0.00106796	*LBX2*
5	30325001	30375000	0.00106789	*SPATS2*
22	23675001	23725000	0.00106783	*CNTN4*
24	22850001	22900000	0.00106783	*NOL4*
10	36550001	36600000	0.00106781	*INO80*
13	50675001	50725000	0.00106768	*HAO1*
17	54300001	54350000	0.00106767	*ANAPC7*
17	54300001	54350000	0.00106767	*ARPC3*
17	54300001	54350000	0.00106767	*GPN3*
6	77050001	77100000	0.00106764	*ADGRL3*
10	21400001	21450000	0.0010675	*THTPA*
10	21400001	21450000	0.0010675	*ZFHX2*
21	1975001	2025000	0.00106729	*SNORD116*
5	66225001	66275000	0.00106726	*IGF1*
3	20175001	20225000	0.00106684	*RPRD2*
3	20175001	20225000	0.00106684	*TARS2*
3	20175001	20225000	0.00106684	*ECM1*
11	74475001	74525000	0.00106677	*NCOA1*
16	50725001	50775000	0.00106676	*GABRD*
9	39775001	39825000	0.00106665	*DDO*
9	39775001	39825000	0.00106665	*SLC22A16*
1	126000001	126050000	0.0010666	*PAQR9*
1	107225001	107275000	0.00106651	*C1H3orf80*
3	16275001	16325000	0.00106609	*UBAP2L*
3	16275001	16325000	0.00106609	*C3H1orf43*
3	16275001	16325000	0.00106609	*TPM3*
3	16275001	16325000	0.00106609	*CFAP141*
5	51225001	51275000	0.00106576	*USP15*
22	11675001	11725000	0.00106548	*OXSR1*
13	31500001	31550000	0.00106529	*CUBN*
1	79575001	79625000	0.00106525	*BCL6*
9	103900001	103950000	0.00106525	*DLL1*
9	103900001	103950000	0.00106525	*FAM120B*
14	23325001	23375000	0.00106515	*PLAG1*
7	53300001	53350000	0.00106513	*SPRY4*
1	126625001	126675000	0.00106504	*XRN1*
2	91575001	91625000	0.0010647	*ABI2*
1	52100001	52150000	0.00106461	*BBX*
9	73475001	73525000	0.00106429	*AHI1*
10	35250001	35300000	0.00106428	*FSIP1*
18	38100001	38150000	0.00106418	*ZFHX3*
25	41800001	41850000	0.00106409	*SUN1*
25	41800001	41850000	0.00106409	*GET4*
25	41800001	41850000	0.00106409	*ADAP1*
9	41675001	41725000	0.00106391	*AFG1L*
7	70625001	70675000	0.0010639	*EBF1*
7	50375001	50425000	0.00106382	*SIL1*
20	31175001	31225000	0.00106372	*NNT*
23	16300001	16350000	0.00106361	*UBR2*
21	6925001	6975000	0.00106313	*MEF2A*
16	43700001	43750000	0.0010631	*CLSTN1*

14	34950001	35000000	0.00106297	*EYA1*
25	35875001	35925000	0.00106288	*GIGYF1*
25	35875001	35925000	0.00106288	*GNB2*
25	35875001	35925000	0.00106288	*POP7*
25	35875001	35925000	0.00106288	*ACTL6B*
25	35875001	35925000	0.00106288	*EPO*
15	58750001	58800000	0.00106281	*KIF18A*
12	11200001	11250000	0.00106265	*ELF1*
12	11200001	11250000	0.00106265	*SNORA70*
29	46025001	46075000	0.00106253	*PPP6R3*
1	111275001	111325000	0.00106219	*KCNAB1*
1	109350001	109400000	0.00106218	*RSRC1*
29	40875001	40925000	0.00106208	*AHNAK*
29	40875001	40925000	0.00106208	*EEF1G*
15	29125001	29175000	0.00106203	*KMT2A*
15	29125001	29175000	0.00106203	*SNORA70*
18	39450001	39500000	0.00106195	*TAT*
18	39450001	39500000	0.00106195	*U6*
7	43275001	43325000	0.00106184	*PALM*
7	43275001	43325000	0.00106184	*FSTL3*
7	43275001	43325000	0.00106184	*PRSS57*
15	52525001	52575000	0.00106177	*FCHSD2*
17	54575001	54625000	0.00106165	*PPP1CC*
10	58550001	58600000	0.0010613	*TMOD2*
2	53000001	53050000	0.00106117	*ARHGAP15*
6	36650001	36700000	0.00106115	*PKD2*
6	36650001	36700000	0.00106115	*SPP1*
9	63775001	63825000	0.00106103	*SYNCRIP*
9	63775001	63825000	0.00106103	*SNORD50B*
13	23575001	23625000	0.00106094	*PIP4K2A*
1	65400001	65450000	0.0010605	*RABL3*
1	65400001	65450000	0.0010605	*GTF2E1*
1	65400001	65450000	0.0010605	*SNORA70*
28	17900001	17950000	0.00106048	*ARID5B*
3	23250001	23300000	0.00106043	*NOTCH2*
23	25575001	25625000	0.00106028	*BOLA-DQB*
20	14225001	14275000	0.00106026	*ADAMTS6*
7	82025001	82075000	0.00106025	*ATG10*
13	51575001	51625000	0.00106025	*ATRN*
12	36600001	36650000	0.00106012	*ATP12A*
25	34925001	34975000	0.00105997	*CUX1*
13	52225001	52275000	0.00105988	*PTPRA*
10	58800001	58850000	0.00105969	*DMXL2*
5	69775001	69825000	0.00105958	*POLR3B*
19	11125001	11175000	0.00105954	*MED13*
1	158350001	158400000	0.00105954	*KHDRBS1*
1	158350001	158400000	0.00105954	*ASMT*
19	11125001	11175000	0.00105954	*INTS2*
1	1900001	1950000	0.00105908	*CRYZL1*
1	1900001	1950000	0.00105908	*ITSN1*
1	1900001	1950000	0.00105908	*DONSON*
8	22050001	22100000	0.0010588	*CDKN2B*

8	22050001	22100000	0.0010588	*CDKN2A*
14	21000001	21050000	0.00105819	*PCMTD1*
13	46400001	46450000	0.00105768	*LARP4B*
1	58400001	58450000	0.00105767	*NAA50*
1	58400001	58450000	0.00105767	*ATP6V1A*
25	41700001	41750000	0.00105755	*C25H7orf50*
25	41700001	41750000	0.00105755	*GPR146*
25	41700001	41750000	0.00105755	*CYP2W1*
19	27275001	27325000	0.00105751	*SENP3*
19	27275001	27325000	0.00105751	*TNFSF12*
19	27275001	27325000	0.00105751	*EIF4A1*
19	27275001	27325000	0.00105751	*MPDU1*
19	27275001	27325000	0.00105751	*TNFSF13*
19	27275001	27325000	0.00105751	*FXR2*
19	27275001	27325000	0.00105751	*CD68*
19	27275001	27325000	0.00105751	*SOX15*
19	27275001	27325000	0.00105751	*SNORD10*
19	27275001	27325000	0.00105751	*SNORA48*
22	7625001	7675000	0.00105737	*UBP1*
22	7625001	7675000	0.00105737	*FBXL2*
20	14000001	14050000	0.00105726	*CENPK*
20	14000001	14050000	0.00105726	*ADAMTS6*
26	21750001	21800000	0.00105709	*SLF2*
5	56625001	56675000	0.001057	*HSD17B6*
4	102800001	102850000	0.00105686	*UBN2*
19	23075001	23125000	0.00105667	*SMG6*
8	61900001	61950000	0.00105667	*DCAF10*
19	23075001	23125000	0.00105667	*HIC1*
19	34600001	34650000	0.00105651	*TOM1L2*
19	34600001	34650000	0.00105651	*SREBF1*
1	70625001	70675000	0.00105639	*TFRC*
1	70625001	70675000	0.00105639	*TNK2*
3	58850001	58900000	0.00105615	*SYDE2*
9	96000001	96050000	0.00105613	*WTAP*
9	96000001	96050000	0.00105613	*ACAT2*
9	96000001	96050000	0.00105613	*TCP1*
5	119500001	119550000	0.00105591	*MOV10L1*
5	119500001	119550000	0.00105591	*PANX2*
2	107350001	107400000	0.0010559	*SPEG*
2	107350001	107400000	0.0010559	*DES*
10	51425001	51475000	0.00105582	*MINDY2*
10	51425001	51475000	0.00105582	*U6*
26	31425001	31475000	0.00105578	*PDCD4*
26	31425001	31475000	0.00105578	*BBIP1*
7	51025001	51075000	0.00105494	*NRG2*
7	51025001	51075000	0.00105494	*PSD2*
3	99525001	99575000	0.00105474	*MKNK1*
10	54450001	54500000	0.00105467	*NEDD4*
8	48150001	48200000	0.00105459	*C8H9orf85*
8	48150001	48200000	0.00105459	*ABHD17B*
2	125700001	125750000	0.00105458	*FGR*
3	16200001	16250000	0.0010544	*ATP8B2*

3	16200001	16250000	0.0010544	*AQP10*
15	42525001	42575000	0.00105404	*SBF2*
2	120350001	120400000	0.001054	*PHC2*
2	79475001	79525000	0.0010539	*GLS*
2	79475001	79525000	0.0010539	*STAT1*
8	10300001	10350000	0.00105377	*FBXO16*
8	10300001	10350000	0.00105377	*ZNF395*
1	80025001	80075000	0.00105376	*MASP1*
6	116650001	116700000	0.00105358	*NSD2*
3	30700001	30750000	0.00105356	*ST7L*
3	30700001	30750000	0.00105356	*CAPZA1*
13	46900001	46950000	0.00105341	*ZMYND11*
3	108350001	108400000	0.00105314	*MEAF6*
3	108350001	108400000	0.00105314	*SNIP1*
10	43350001	43400000	0.00105305	*MAP4K5*
6	21925001	21975000	0.00105293	*CISD2*
6	21925001	21975000	0.00105293	*SLC9B1*
7	53350001	53400000	0.00105285	*SPRY4*
6	67675001	67725000	0.00105276	*DCUN1D4*
6	67675001	67725000	0.00105276	*CWH43*
5	47950001	48000000	0.00105267	*HMGA2*
16	79200001	79250000	0.00105258	*KIF14*
16	79200001	79250000	0.00105258	*U6*
16	79200001	79250000	0.00105258	*U6*
19	44250001	44300000	0.00105249	*GPATCH8*
13	56625001	56675000	0.00105238	*SYCP2*
7	83150001	83200000	0.00105233	*XRCC4*
10	60200001	60250000	0.00105206	*ATP8B4*
2	122525001	122575000	0.0010517	*PUM1*
18	14650001	14700000	0.00105165	*SPIRE2*
18	14650001	14700000	0.00105165	*TCF25*
19	44275001	44325000	0.00105156	*GPATCH8*
12	32075001	32125000	0.00105141	*CDX2*
12	32075001	32125000	0.00105141	*PDX1*
12	32075001	32125000	0.00105141	*URAD*
17	53950001	54000000	0.00105128	*P2RX7*
11	97475001	97525000	0.00105125	*LMX1B*
2	107000001	107050000	0.00105112	*NHEJ1*
2	107000001	107050000	0.00105112	*IHH*
3	94875001	94925000	0.00105103	*EPS15*
3	34350001	34400000	0.00105095	*WDR47*
3	34350001	34400000	0.00105095	*CLCC1*
20	6425001	6475000	0.00105093	*MSX2*
19	39875001	39925000	0.0010506	*CDK12*
8	39000001	39050000	0.00105049	*RIC1*
10	59900001	59950000	0.00105031	*GABPB1*
7	60425001	60475000	0.00105003	*SH3TC2*
8	75775001	75825000	0.00104991	*FAM219A*
29	41075001	41125000	0.00104989	*STX5*
29	41075001	41125000	0.00104989	*NXF1*
29	41075001	41125000	0.00104989	*TAF6L*
29	41075001	41125000	0.00104989	*POLR2G*

29	41075001	41125000	0.00104989	*TMEM179B*
29	41075001	41125000	0.00104989	*TMEM223*
1	117675001	117725000	0.00104988	*TSC22D2*
3	64600001	64650000	0.00104977	*U6*
17	54500001	54550000	0.00104967	*HVCN1*
17	54500001	54550000	0.00104967	*TCTN1*
1	84125001	84175000	0.00104961	*ATP11B*
2	4750001	4800000	0.00104929	*WDR33*
2	4750001	4800000	0.00104929	*SFT2D3*
4	76825001	76875000	0.00104907	*OGDH*
4	76825001	76875000	0.00104907	*DDX56*
4	76825001	76875000	0.00104907	*TMED4*
17	54425001	54475000	0.00104899	*PPTC7*
1	80425001	80475000	0.00104886	*RFC4*
1	80425001	80475000	0.00104886	*EIF4A2*
1	80425001	80475000	0.00104886	*ADIPOQ*
1	80425001	80475000	0.00104886	*SNORA81*
1	80425001	80475000	0.00104886	*SNORA63*
1	80425001	80475000	0.00104886	*SNORD2*
2	90550001	90600000	0.00104876	*FZD7*
23	31950001	32000000	0.00104866	*SLC17A2*
23	31950001	32000000	0.00104866	*SLC17A3*
3	92750001	92800000	0.00104835	*DMRTB1*
20	4000001	4050000	0.00104829	*UBTD2*
7	44150001	44200000	0.00104805	*REXO1*
7	44150001	44200000	0.00104805	*KLF16*
7	44150001	44200000	0.00104805	*ABHD17A*
7	31825001	31875000	0.00104804	*SRFBP1*
4	56075001	56125000	0.00104793	*ZNF277*
4	56075001	56125000	0.00104793	*DOCK4*
4	43275001	43325000	0.00104774	*PHTF2*
7	70600001	70650000	0.00104769	*EBF1*
6	86550001	86600000	0.00104702	*SLC4A4*
10	37050001	37100000	0.00104698	*MGA*
5	36650001	36700000	0.00104698	*IRAK4*
5	36650001	36700000	0.00104698	*TWF1*
9	74200001	74250000	0.0010469	*MTFR2*
9	62675001	62725000	0.00104681	*ZNF292*
10	20400001	20450000	0.00104678	*NPTN*
2	127550001	127600000	0.00104646	*MACO1*
18	7775001	7825000	0.0010463	*PKD1L2*
3	16525001	16575000	0.0010463	*GATAD2B*
6	66550001	66600000	0.00104622	*CORIN*
6	66550001	66600000	0.00104622	*NFXL1*
29	12400001	12450000	0.00104617	*ANKRD42*
29	12400001	12450000	0.00104617	*CCDC90B*
3	18625001	18675000	0.00104616	*TCHHL1*
3	18625001	18675000	0.00104616	*TCHH*
3	45375001	45425000	0.00104541	*DPYD*
18	36900001	36950000	0.0010454	*WWP2*
5	69975001	70025000	0.00104539	*RFX4*
1	78850001	78900000	0.00104536	*LPP*

9	63875001	63925000	0.00104526	*SNX14*
5	60525001	60575000	0.00104525	*ELK3*
5	60525001	60575000	0.00104525	*CDK17*
16	41800001	41850000	0.001045	*CLCN6*
16	41800001	41850000	0.001045	*NPPA*
16	41800001	41850000	0.001045	*NPPB*
16	50950001	51000000	0.00104461	*CDK11B*
16	50950001	51000000	0.00104461	*SLC35E2*
16	50950001	51000000	0.00104461	*NADK*
7	87725001	87775000	0.00104452	*TMEM161B*
3	62975001	63025000	0.00104402	*ADGRL2*
2	90525001	90575000	0.00104365	*FZD7*
1	55925001	55975000	0.0010436	*NECTIN3*
17	53600001	53650000	0.00104351	*ORAI1*
17	53600001	53650000	0.00104351	*MORN3*
24	49100001	49150000	0.0010434	*DYM*
24	49100001	49150000	0.0010434	*C24H18orf32*
25	18625001	18675000	0.0010433	*DCUN1D3*
10	20925001	20975000	0.00104296	*NEDD8*
10	20925001	20975000	0.00104296	*IPO4*
10	20925001	20975000	0.00104296	*GMPR2*
10	20925001	20975000	0.00104296	*TSSK4*
10	20925001	20975000	0.00104296	*TINF2*
10	20925001	20975000	0.00104296	*TM9SF1*
10	20925001	20975000	0.00104296	*CHMP4A*
10	20925001	20975000	0.00104296	*MDP1*
9	103925001	103975000	0.00104294	*FAM120B*
9	103925001	103975000	0.00104294	*DLL1*
5	66725001	66775000	0.00104287	*PAH*
5	66725001	66775000	0.00104287	*ASCL1*
22	48425001	48475000	0.00104275	*SEMA3G*
22	48425001	48475000	0.00104275	*PHF7*
22	48425001	48475000	0.00104275	*NISCH*
22	48425001	48475000	0.00104275	*TNNC1*
13	38550001	38600000	0.00104266	*SEC23B*
13	38550001	38600000	0.00104266	*RBBP9*
13	38550001	38600000	0.00104266	*SMIM26*
16	39350001	39400000	0.00104256	*DNM3*
5	119750001	119800000	0.00104248	*SBF1*
5	119750001	119800000	0.00104248	*ADM2*
5	119750001	119800000	0.00104248	*MIOX*
5	119750001	119800000	0.00104248	*PPP6R2*
6	86500001	86550000	0.00104238	*SLC4A4*
5	112525001	112575000	0.00104222	*TOB2*
5	112525001	112575000	0.00104222	*PHF5A*
5	112525001	112575000	0.00104222	*ACO2*
21	2275001	2325000	0.00104218	*UBE3A*
24	48725001	48775000	0.00104217	*DYM*
10	61225001	61275000	0.00104206	*SECISBP2L*
4	46325001	46375000	0.0010417	*SRPK2*
7	20425001	20475000	0.0010417	*NFIC*
7	20425001	20475000	0.0010417	*SMIM24*

7	20425001	20475000	0.0010417	*DOHH*
12	33875001	33925000	0.00104169	*NUP58*
10	76625001	76675000	0.00104165	*ZBTB1*
10	76625001	76675000	0.00104165	*ZBTB25*
4	102825001	102875000	0.00104157	*UBN2*
1	72200001	72250000	0.00104155	*ACAP2*
26	18500001	18550000	0.00104152	*ARHGAP19*
9	43725001	43775000	0.00104129	*ATG5*
8	38050001	38100000	0.00104126	*KDM4C*
7	11800001	11850000	0.00104106	*ZSWIM4*
7	11800001	11850000	0.00104106	*BRME1*
7	11800001	11850000	0.00104106	*NANOS3*
19	30350001	30400000	0.00104047	*DNAH9*
2	36725001	36775000	0.00104044	*BAZ2B*
12	31875001	31925000	0.00104036	*PAN3*
26	23225001	23275000	0.00104032	*SUFU*
26	23225001	23275000	0.00104032	*TRIM8*
11	15475001	15525000	0.0010403	*LTBP1*
11	15475001	15525000	0.0010403	*U6*
9	104050001	104100000	0.00104025	*PSMB1*
9	104050001	104100000	0.00104025	*TBP*
19	46225001	46275000	0.00104016	*CDC27*
14	40000001	40050000	0.00104012	*ZFHX4*
7	94000001	94050000	0.00104005	*MCTP1*
7	94000001	94050000	0.00104005	*SLF1*
13	12150001	12200000	0.00103992	*CDC123*
13	12150001	12200000	0.00103992	*NUDT5*
12	35350001	35400000	0.00103988	*FGF9*
19	12675001	12725000	0.00103982	*USP32*
19	12675001	12725000	0.00103982	*U6*
9	50300001	50350000	0.00103963	*USP45*
9	50300001	50350000	0.00103963	*PNISR*
4	76725001	76775000	0.00103949	*ZMIZ2*
4	76725001	76775000	0.00103949	*OGDH*
7	57500001	57550000	0.00103944	*POU4F3*
21	22325001	22375000	0.00103934	*SEC11A*
11	43525001	43575000	0.00103933	*PAPOLG*
16	39050001	39100000	0.00103932	*PRRC2C*
16	39050001	39100000	0.00103932	*MYOC*
2	119700001	119750000	0.00103931	*DIS3L2*
13	50550001	50600000	0.00103918	*HAO1*
9	96525001	96575000	0.001039	*SLC22A3*
21	59600001	59650000	0.00103884	*GSC*
9	94725001	94775000	0.00103877	*GTF2H5*
15	76100001	76150000	0.00103866	*PHF21A*
17	72950001	73000000	0.00103866	*TANGO2*
17	72950001	73000000	0.00103866	*DGCR8*
17	72950001	73000000	0.00103866	*RANBP1*
17	72950001	73000000	0.00103866	*TRMT2A*
17	72950001	73000000	0.00103866	*MIR3618*
17	72950001	73000000	0.00103866	*MIR1306*
17	72950001	73000000	0.00103866	*MIR185*

17	67675001	67725000	0.00103858	*TTC28*
18	54600001	54650000	0.00103857	*BICRA*
18	54600001	54650000	0.00103857	*EHD2*
18	54600001	54650000	0.00103857	*NOP53*
27	14100001	14150000	0.00103841	*WWC2*
5	95425001	95475000	0.00103836	*ATF7IP*
5	56825001	56875000	0.00103832	*RBMS2*
5	56825001	56875000	0.00103832	*BAZ2A*
8	39800001	39850000	0.00103809	*AK3*
8	39800001	39850000	0.00103809	*CDC37L1*
8	39800001	39850000	0.00103809	*SPATA6L*
9	41600001	41650000	0.00103806	*FOXO3*
3	112450001	112500000	0.00103802	*GIGYF2*
17	62150001	62200000	0.00103802	*TMEM116*
17	62150001	62200000	0.00103802	*U6*
20	3675001	3725000	0.00103783	*FBXW11*
10	53875001	53925000	0.00103767	*MNS1*
3	74925001	74975000	0.00103763	*SRSF11*
13	65975001	66025000	0.00103747	*RBL1*
13	65975001	66025000	0.00103747	*SAMHD1*
13	46875001	46925000	0.00103719	*ZMYND11*
13	33425001	33475000	0.00103701	*ARHGAP12*
14	1550001	1600000	0.00103699	*CYP11B1*
2	91350001	91400000	0.00103688	*NBEAL1*
13	57450001	57500000	0.00103688	*GNAS*
13	57450001	57500000	0.00103688	*GNAS*
15	14325001	14375000	0.00103668	*MTMR2*
19	38825001	38875000	0.00103626	*NPEPPS*
19	38825001	38875000	0.00103626	*U6*
18	36250001	36300000	0.00103563	*TANGO6*
13	33475001	33525000	0.00103553	*ARHGAP12*
19	37000001	37050000	0.0010355	*SPOP*
19	37000001	37050000	0.0010355	*NXPH3*
9	94775001	94825000	0.00103538	*TULP4*
10	49575001	49625000	0.00103527	*RORA*
7	51225001	51275000	0.00103519	*NRG2*
7	51225001	51275000	0.00103519	*U6*
7	51625001	51675000	0.00103495	*U6*
11	94050001	94100000	0.00103484	*RC3H2*
11	94050001	94100000	0.00103484	*RABGAP1*
11	94050001	94100000	0.00103484	*ZBTB6*
11	94050001	94100000	0.00103484	*ZBTB26*
7	43300001	43350000	0.00103478	*PALM*
7	43300001	43350000	0.00103478	*MISP*
20	62800001	62850000	0.0010347	*MARCHF6*
20	62800001	62850000	0.0010347	*ROPN1L*
27	14250001	14300000	0.00103458	*CDKN2AIP*
10	36925001	36975000	0.0010343	*RPAP1*
10	36925001	36975000	0.0010343	*TYRO3*
10	36925001	36975000	0.0010343	*ITPKA*
3	112525001	112575000	0.00103405	*GIGYF2*
1	52225001	52275000	0.00103404	*BBX*

12	18725001	18775000	0.00103401	*FNDC3A*
1	83025001	83075000	0.00103398	*HTR3C*
2	47725001	47775000	0.00103379	*MBD5*
18	36300001	36350000	0.00103357	*HAS3*
18	36300001	36350000	0.00103357	*CHTF8*
18	36300001	36350000	0.00103357	*UTP4*
18	36300001	36350000	0.00103357	*TANGO6*
2	58575001	58625000	0.00103356	*SPOPL*
5	71675001	71725000	0.00103332	*MGC137211*
29	40175001	40225000	0.00103312	*MYRF*
29	40175001	40225000	0.00103312	*DAGLA*
4	55275001	55325000	0.00103291	*GPR85*
15	56025001	56075000	0.00103283	*LRRC32*
21	6900001	6950000	0.00103247	*MEF2A*
17	56850001	56900000	0.00103237	*SUDS3*
2	85325001	85375000	0.00103219	*GTF3C3*
2	85325001	85375000	0.00103219	*CCDC150*
15	1750001	1800000	0.00103202	*MSANTD4*
18	23925001	23975000	0.00103193	*CES1*
18	23925001	23975000	0.00103193	*SLC6A2*
3	12550001	12600000	0.00103183	*FCRL1*
22	42375001	42425000	0.00103166	*CFAP20DC*
5	62675001	62725000	0.0010315	*TMPO*
9	96225001	96275000	0.00103149	*IGF2R*
19	10725001	10775000	0.00103141	*VMP1*
13	60250001	60300000	0.00103134	*FAM110A*
13	60250001	60300000	0.00103134	*SLC52A3*
1	65425001	65475000	0.00103109	*GTF2E1*
1	65425001	65475000	0.00103109	*RABL3*
1	65425001	65475000	0.00103109	*SNORA70*
5	93250001	93300000	0.00103107	*LMO3*
3	69050001	69100000	0.00103102	*ACADM*
3	69050001	69100000	0.00103102	*RABGGTB*
3	69050001	69100000	0.00103102	*MSH4*
3	69050001	69100000	0.00103102	*SNORD45A*
3	69050001	69100000	0.00103102	*SNORD45C*
19	12725001	12775000	0.00103093	*USP32*
23	9625001	9675000	0.00103062	*FKBP5*
3	20750001	20800000	0.00103053	*H4C14*
3	20750001	20800000	0.00103053	*H2AC18*
3	20750001	20800000	0.00103053	*H3C13*
3	20750001	20800000	0.00103053	*H2BC18*
16	68950001	69000000	0.00103047	*CENPF*
12	20275001	20325000	0.00103041	*DLEU7*
25	27150001	27200000	0.00103039	*ZNF646*
25	27150001	27200000	0.00103039	*ZNF668*
25	27150001	27200000	0.00103039	*STX4*
25	27150001	27200000	0.00103039	*PRSS53*
7	53150001	53200000	0.0010302	*NDFIP1*
7	38950001	39000000	0.00103015	*DBN1*
7	38950001	39000000	0.00103015	*PRR7*
4	46375001	46425000	0.00103007	*SRPK2*

2	99250001	99300000	0.00102988	*ERBB4*
10	71925001	71975000	0.00102988	*RTN1*
29	12450001	12500000	0.00102979	*ANKRD42*
29	12450001	12500000	0.00102979	*PCF11*
3	59975001	60025000	0.00102973	*PRKACB*
4	53775001	53825000	0.00102967	*FOXP2*
19	44225001	44275000	0.00102965	*GPATCH8*
27	36400001	36450000	0.00102956	*GOLGA7*
25	18550001	18600000	0.00102945	*REXO5*
25	18550001	18600000	0.00102945	*ERI2*
8	59575001	59625000	0.00102943	*UNC13B*
5	43100001	43150000	0.00102942	*KCNMB4*
5	43100001	43150000	0.00102942	*CNOT2*
16	51075001	51125000	0.00102941	*ATAD3A*
16	51075001	51125000	0.00102941	*TMEM240*
16	51075001	51125000	0.00102941	*VWA1*
16	51075001	51125000	0.00102941	*TMEM88B*
16	51075001	51125000	0.00102941	*SSU72*
2	44650001	44700000	0.00102904	*RIF1*
2	44650001	44700000	0.00102904	*NEB*
8	38000001	38050000	0.00102898	*KDM4C*
9	39800001	39850000	0.00102897	*DDO*
10	37075001	37125000	0.00102889	*MGA*
11	24575001	24625000	0.00102861	*EML4*
16	12200001	12250000	0.00102828	*UCHL5*
12	19450001	19500000	0.00102817	*TRIM13*
12	19450001	19500000	0.00102817	*KCNRG*
3	66700001	66750000	0.0010279	*FUBP1*
29	45200001	45250000	0.0010279	*ANKRD13D*
29	45200001	45250000	0.0010279	*GRK2*
29	45200001	45250000	0.0010279	*SSH3*
3	66700001	66750000	0.0010279	*DNAJB4*
3	66700001	66750000	0.0010279	*NEXN*
4	12600001	12650000	0.00102787	*PON3*
4	12600001	12650000	0.00102787	*PON2*
19	26425001	26475000	0.00102747	*ENO3*
19	26425001	26475000	0.00102747	*CAMTA2*
19	26425001	26475000	0.00102747	*RNF167*
19	26425001	26475000	0.00102747	*SPAG7*
19	26425001	26475000	0.00102747	*PFN1*
19	26425001	26475000	0.00102747	*SLC25A11*
19	26425001	26475000	0.00102747	*GP1BA*
1	64650001	64700000	0.00102739	*GSK3B*
11	18950001	19000000	0.00102732	*CRIM1*
1	132800001	132850000	0.00102722	*STAG1*
22	50250001	50300000	0.0010272	*RBM6*
3	101075001	101125000	0.00102708	*EIF2B3*
22	11550001	11600000	0.001027	*DLEC1*
22	11550001	11600000	0.001027	*ACAA1*
5	120050001	120100000	0.00102668	*RABL2B*
4	29000001	29050000	0.00102657	*ITGB8*
5	26775001	26825000	0.00102649	*RARG*

5	26775001	26825000	0.00102649	*ITGB7*
5	26775001	26825000	0.00102649	*ZNF740*
5	26775001	26825000	0.00102649	*CSAD*
8	75225001	75275000	0.00102636	*UBE2R2*
24	48950001	49000000	0.00102612	*DYM*
20	30500001	30550000	0.00102605	*FGF10*
5	57425001	57475000	0.00102605	*SARNP*
5	57425001	57475000	0.00102605	*DNAJC14*
5	57425001	57475000	0.00102605	*MMP19*
5	57425001	57475000	0.00102605	*TMEM198B*
5	57425001	57475000	0.00102605	*ORMDL2*
8	59675001	59725000	0.00102586	*RUSC2*
18	35700001	35750000	0.00102558	*SMPD3*
18	35700001	35750000	0.00102558	*PRMT7*
7	22675001	22725000	0.00102522	*FNIP1*
8	39200001	39250000	0.0010252	*PDCD1LG2*
8	39200001	39250000	0.0010252	*CD274*
10	60825001	60875000	0.00102517	*FGF7*
5	57025001	57075000	0.00102512	*CS*
5	57025001	57075000	0.00102512	*PAN2*
5	57025001	57075000	0.00102512	*COQ10A*
5	57025001	57075000	0.00102512	*CNPY2*
5	57025001	57075000	0.00102512	*ANKRD52*
10	58575001	58625000	0.00102484	*TMOD2*
11	73575001	73625000	0.00102464	*ASXL2*
4	78725001	78775000	0.00102463	*GLI3*
25	34975001	35025000	0.00102451	*CUX1*
19	38175001	38225000	0.00102448	*SKAP1*
15	350001	400000	0.00102375	*OR4C174*
3	99800001	99850000	0.00102369	*RAD54L*
3	99800001	99850000	0.00102369	*LRRC41*
11	7800001	7850000	0.00102369	*U6*
7	50400001	50450000	0.00102368	*SIL1*
2	20875001	20925000	0.00102358	*LNPK*
11	38025001	38075000	0.00102341	*CCDC88A*
4	70075001	70125000	0.00102326	*U6*
10	61725001	61775000	0.001023	*FBN1*
11	30225001	30275000	0.00102274	*FBXO11*
12	46150001	46200000	0.00102263	*DACH1*
25	41850001	41900000	0.0010226	*SUN1*
25	41850001	41900000	0.0010226	*DNAAF5*
21	41725001	41775000	0.00102259	*HECTD1*
23	8350001	8400000	0.00102247	*NUDT3*
2	34950001	35000000	0.0010223	*PSMD14*
2	34950001	35000000	0.0010223	*TBR1*
10	34050001	34100000	0.00102229	*FAM98B*
10	34050001	34100000	0.00102229	*U6*
1	64675001	64725000	0.00102214	*GSK3B*
4	29475001	29525000	0.00102181	*SP8*
10	54625001	54675000	0.0010218	*PRTG*
22	49625001	49675000	0.00102169	*DOCK3*
2	106875001	106925000	0.00102169	*CDK5R2*

2	106875001	106925000	0.00102169	*FEV*
4	46300001	46350000	0.00102165	*SRPK2*
18	44950001	45000000	0.00102163	*U6*
11	63025001	63075000	0.00102151	*SERTAD2*
19	44000001	44050000	0.00102123	*UBTF*
19	44000001	44050000	0.00102123	*ATXN7L3*
19	44000001	44050000	0.00102123	*TMUB2*
2	71525001	71575000	0.00102085	*PTPN4*
3	82000001	82050000	0.00102075	*EFCAB7*
11	94250001	94300000	0.00102073	*STRBP*
11	94250001	94300000	0.00102073	*RABGAP1*
9	38850001	38900000	0.00102065	*TRAF3IP2*
1	126675001	126725000	0.00102052	*XRN1*
13	57975001	58025000	0.00102047	*RAB22A*
3	103550001	103600000	0.00102025	*CCDC30*
3	103550001	103600000	0.00102025	*PPIH*
5	95400001	95450000	0.00102016	*ATF7IP*
21	32600001	32650000	0.00101989	*HMG20A*
10	19400001	19450000	0.00101988	*ARIH1*
17	63075001	63125000	0.00101969	*SPPL3*
17	63075001	63125000	0.00101969	*Metazoa_SRP*
17	69050001	69100000	0.00101968	*MTMR3*
3	83275001	83325000	0.00101961	*KANK4*
9	93500001	93550000	0.00101933	*ARID1B*
17	54325001	54375000	0.00101908	*GPN3*
17	54325001	54375000	0.00101908	*ARPC3*
17	54325001	54375000	0.00101908	*FAM216A*
17	54325001	54375000	0.00101908	*VPS29*
21	26025001	26075000	0.0010189	*ZFAND6*
18	44850001	44900000	0.00101884	*UBA2*
18	44850001	44900000	0.00101884	*WTIP*
2	104675001	104725000	0.00101879	*IGFBP5*
7	22650001	22700000	0.0010186	*FNIP1*
19	56625001	56675000	0.00101856	*TMEM104*
19	56625001	56675000	0.00101856	*SLC9A3R1*
19	56625001	56675000	0.00101856	*NAT9*
19	56625001	56675000	0.00101856	*RAB37*
19	56625001	56675000	0.00101856	*U6*
14	20975001	21025000	0.00101853	*PCMTD1*
14	20975001	21025000	0.00101853	*U6*
5	26725001	26775000	0.00101847	*ESPL1*
5	26725001	26775000	0.00101847	*PFDN5*
5	26725001	26775000	0.00101847	*MFSD5*
5	26725001	26775000	0.00101847	*RARG*
8	38975001	39025000	0.00101829	*RIC1*
25	28650001	28700000	0.00101828	*CALN1*
15	53450001	53500000	0.00101798	*C2CD3*
15	53450001	53500000	0.00101798	*UCP3*
17	67650001	67700000	0.0010179	*TTC28*
18	19125001	19175000	0.00101778	*CYLD*
12	36325001	36375000	0.0010176	*MPHOSPH8*
22	50850001	50900000	0.0010176	*LAMB2*

22	50850001	50900000	0.0010176	*KLHDC8B*
22	50850001	50900000	0.0010176	*C22H3orf84*
22	50850001	50900000	0.0010176	*CCDC71*
22	50850001	50900000	0.0010176	*USP19*
11	94350001	94400000	0.00101742	*STRBP*
11	94350001	94400000	0.00101742	*U6*
2	53375001	53425000	0.00101739	*ARHGAP15*
5	29400001	29450000	0.00101729	*LARP4*
5	29400001	29450000	0.00101729	*DIP2B*
10	36250001	36300000	0.00101712	*RAD51*
10	36250001	36300000	0.00101712	*RMDN3*
5	55875001	55925000	0.001017	*KIF5A*
5	55875001	55925000	0.001017	*PIP4K2C*
7	18700001	18750000	0.001017	*SAFB2*
5	55875001	55925000	0.001017	*DTX3*
5	55875001	55925000	0.001017	*ARHGEF25*
16	44750001	44800000	0.00101673	*RERE*
15	48900001	48950000	0.00101671	*OR51A4*
15	48900001	48950000	0.00101671	*OR51A7*
15	48900001	48950000	0.00101671	*U6*
8	75250001	75300000	0.00101656	*UBE2R2*
5	75850001	75900000	0.00101654	*MFNG*
9	65800001	65850000	0.00101646	*THEMIS*
10	57800001	57850000	0.00101565	*ARPP19*
10	57800001	57850000	0.00101565	*FAM214A*
10	57800001	57850000	0.00101565	*MYO5A*
8	22525001	22575000	0.00101545	*IFNE*
17	53575001	53625000	0.00101534	*ORAI1*
17	53575001	53625000	0.00101534	*MORN3*
4	43600001	43650000	0.00101531	*PTPN12*
4	69100001	69150000	0.00101531	*SKAP2*
6	49950001	50000000	0.00101519	*PCDH7*
15	17700001	17750000	0.00101498	*CUL5*
15	17700001	17750000	0.00101498	*ACAT1*
15	17700001	17750000	0.00101498	*U6*
5	26475001	26525000	0.00101478	*ATF7*
5	26475001	26525000	0.00101478	*TARBP2*
5	26475001	26525000	0.00101478	*NPFF*
3	45400001	45450000	0.00101454	*DPYD*
16	63925001	63975000	0.0010144	*SHCBP1L*
16	63925001	63975000	0.0010144	*DHX9*
1	70275001	70325000	0.00101414	*LRCH3*
18	38350001	38400000	0.00101395	*ZFHX3*
5	77450001	77500000	0.00101394	*FGD4*
5	77450001	77500000	0.00101394	*BICD1*
13	23500001	23550000	0.00101357	*SPAG6*
8	77175001	77225000	0.00101356	*C8H9orf64*
8	77175001	77225000	0.00101356	*HNRNPK*
8	77175001	77225000	0.00101356	*KIF27*
8	77175001	77225000	0.00101356	*MIR7-1*
21	2025001	2075000	0.00101341	*SNORD116*
21	2025001	2075000	0.00101341	*SNORD116*

21	2025001	2075000	0.00101341	*SNORD116*
21	2025001	2075000	0.00101341	*SNORD116*
21	2025001	2075000	0.00101341	*SNORD116*
21	2025001	2075000	0.00101341	*SNORD116*
23	18525001	18575000	0.00101307	*SUPT3H*
5	107625001	107675000	0.00101299	*WNK1*
11	43750001	43800000	0.00101285	*PEX13*
11	43750001	43800000	0.00101285	*PUS10*
5	42900001	42950000	0.0010126	*PTPRB*
7	46025001	46075000	0.0010126	*JADE2*
26	15575001	15625000	0.00101257	*PLCE1*
17	13100001	13150000	0.00101234	*OTUD4*
11	95375001	95425000	0.00101233	*NEK6*
11	95375001	95425000	0.00101233	*PSMB7*
17	69325001	69375000	0.00101229	*SF3A1*
17	69325001	69375000	0.00101229	*TBC1D10A*
17	69325001	69375000	0.00101229	*CCDC157*
13	74775001	74825000	0.00101215	*NCOA5*
13	74775001	74825000	0.00101215	*SLC12A5*
5	69825001	69875000	0.00101208	*POLR3B*
4	94425001	94475000	0.00101204	*TSGA13*
4	94425001	94475000	0.00101204	*COPG2*
16	50700001	50750000	0.00101204	*GABRD*
22	10650001	10700000	0.00101198	*TRANK1*
10	54750001	54800000	0.00101187	*PRTG*
8	39375001	39425000	0.00101159	*JAK2*
3	59900001	59950000	0.00101158	*PRKACB*
17	53500001	53550000	0.00101149	*TMEM120B*
17	53500001	53550000	0.00101149	*RHOF*
1	67850001	67900000	0.00101147	*HACD2*
2	35125001	35175000	0.0010114	*TANK*
3	59050001	59100000	0.00101109	*MCOLN3*
2	90950001	91000000	0.001011	*BMPR2*
6	59425001	59475000	0.00101098	*RBM47*
28	17600001	17650000	0.00101083	*CABCOCO1*
20	35350001	35400000	0.00101056	*RICTOR*
4	114625001	114675000	0.00101027	*KMT2C*
6	84125001	84175000	0.00101026	*YTHDC1*
5	55900001	55950000	0.00101013	*KIF5A*
5	55900001	55950000	0.00101013	*DCTN2*
5	55900001	55950000	0.00101013	*MBD6*
24	47475001	47525000	0.00101002	*SMAD2*
2	52275001	52325000	0.00100981	*ZEB2*
3	34375001	34425000	0.0010098	*CLCC1*
3	34375001	34425000	0.0010098	*WDR47*
14	41775001	41825000	0.00100962	*PKIA*
14	19600001	19650000	0.00100907	*UBE2V2*
23	15925001	15975000	0.00100904	*TRERF1*
23	15925001	15975000	0.00100904	*MRPS10*
19	43050001	43100000	0.00100897	*BRCA1*
5	119850001	119900000	0.00100897	*SYCE3*
5	119850001	119900000	0.00100897	*CPT1B*

19	43050001	43100000	0.00100897	*RND2*
5	119850001	119900000	0.00100897	*CHKB*
5	119850001	119900000	0.00100897	*KLHDC7B*
19	43050001	43100000	0.00100897	*VAT1*
8	325001	375000	0.00100886	*MFSD14B*
1	84075001	84125000	0.00100885	*ATP11B*
8	28775001	28825000	0.00100841	*PSIP1*
10	54500001	54550000	0.0010083	*NEDD4*
3	34200001	34250000	0.00100814	*ELAPOR1*
7	60350001	60400000	0.00100806	*SH3TC2*
4	31600001	31650000	0.00100794	*FAM126A*
2	43450001	43500000	0.00100792	*PRPF40A*
2	43450001	43500000	0.00100792	*ARL6IP6*
2	43450001	43500000	0.00100792	*U6*
10	54650001	54700000	0.00100774	*PRTG*
8	38950001	39000000	0.00100756	*RIC1*
5	30925001	30975000	0.00100746	*DDX23*
5	30925001	30975000	0.00100746	*CACNB3*
5	30925001	30975000	0.00100746	*RND1*
9	60000001	60050000	0.00100739	*BACH2*
9	62700001	62750000	0.00100691	*ZNF292*
2	34200001	34250000	0.00100667	*FAP*
4	46500001	46550000	0.00100658	*SRPK2*
2	106650001	106700000	0.00100546	*TTLL4*
2	106650001	106700000	0.00100546	*STK36*
25	26550001	26600000	0.00100536	*ZNF771*
25	26550001	26600000	0.00100536	*TBC1D10B*
25	26550001	26600000	0.00100536	*ZNF48*
25	26550001	26600000	0.00100536	*DCTPP1*
25	26550001	26600000	0.00100536	*SEPTIN1*
25	26550001	26600000	0.00100536	*MYL11*
11	43675001	43725000	0.00100527	*PUS10*
14	35000001	35050000	0.00100514	*EYA1*
5	119825001	119875000	0.00100501	*SYCE3*
5	119825001	119875000	0.00100501	*ODF3B*
5	119825001	119875000	0.00100501	*KLHDC7B*
8	38025001	38075000	0.00100499	*KDM4C*
12	34650001	34700000	0.00100489	*SGCG*
12	34650001	34700000	0.00100489	*SACS*
8	59700001	59750000	0.00100479	*RUSC2*
3	32375001	32425000	0.00100448	*LRIF1*
3	9500001	9550000	0.00100443	*ATP1A2*
3	9500001	9550000	0.00100443	*ATP1A4*
3	9500001	9550000	0.00100443	*U6*
9	62650001	62700000	0.00100411	*ZNF292*
1	81100001	81150000	0.00100398	*ETV5*
18	15550001	15600000	0.00100391	*ITFG1*
21	21600001	21650000	0.00100384	*SEMA4B*
21	21600001	21650000	0.00100384	*GDPGP1*
21	21600001	21650000	0.00100384	*CIB1*
21	21600001	21650000	0.00100384	*NGRN*
21	21600001	21650000	0.00100384	*VPS33B*

19	27725001	27775000	0.00100382	*ALOXE3*
19	27725001	27775000	0.00100382	*ALOX12B*
19	27725001	27775000	0.00100382	*HES7*
19	27725001	27775000	0.00100382	*U6*
5	81300001	81350000	0.00100362	*CCDC91*
5	38025001	38075000	0.00100354	*PRICKLE1*
7	93050001	93100000	0.00100332	*FAM172A*
7	93050001	93100000	0.00100332	*NR2F1*
16	51100001	51150000	0.00100329	*CCNL2*
16	51100001	51150000	0.00100329	*VWA1*
16	51100001	51150000	0.00100329	*TMEM88B*
16	51100001	51150000	0.00100329	*MRPL20*
16	51100001	51150000	0.00100329	*ANKRD65*
16	51100001	51150000	0.00100329	*AURKAIP1*
16	51100001	51150000	0.00100329	*ATAD3A*
2	94600001	94650000	0.0010028	*ZDBF2*
16	42800001	42850000	0.00100273	*CASZ1*
17	61900001	61950000	0.00100258	*HECTD4*
7	51600001	51650000	0.00100256	*U6*
22	48250001	48300000	0.00100251	*PBRM1*
22	48250001	48300000	0.00100251	*NT5DC2*
22	48250001	48300000	0.00100251	*SMIM4*
9	25250001	25300000	0.00100232	*TRMT11*
24	49475001	49525000	0.00100227	*MYO5B*
24	49475001	49525000	0.00100227	*ACAA2*
5	30225001	30275000	0.00100211	*FAM186B*
5	30225001	30275000	0.00100211	*PRPF40B*
5	30225001	30275000	0.00100211	*MCRS1*
5	30225001	30275000	0.00100211	*KCNH3*
26	21150001	21200000	0.00100208	*CWF19L1*
26	21150001	21200000	0.00100208	*PKD2L1*
26	21150001	21200000	0.00100208	*BLOC1S2*
10	25675001	25725000	0.0010017	*RAB2B*
10	25675001	25725000	0.0010017	*TOX4*
10	25675001	25725000	0.0010017	*METTL3*
2	71800001	71850000	0.00100117	*EPB41L5*
13	27950001	28000000	0.0010011	*PHYH*
23	8425001	8475000	0.00100109	*NUDT3*
10	34075001	34125000	0.00100107	*RASGRP1*
18	36950001	37000000	0.00100107	*WWP2*
10	34075001	34125000	0.00100107	*FAM98B*
18	36950001	37000000	0.00100107	*PSMD7*
18	36950001	37000000	0.00100107	*MIR140*
14	64600001	64650000	0.00100104	*VPS13B*
3	32950001	33000000	0.00100093	*PROK1*
3	32950001	33000000	0.00100093	*CYM*
7	49625001	49675000	0.00100073	*CDC23*
19	40150001	40200000	0.00100071	*IKZF3*
9	74000001	74050000	0.00100068	*PDE7B*
15	35650001	35700000	0.00100066	*PLEKHA7*
15	35650001	35700000	0.00100066	*C15H11orf58*
3	59075001	59125000	0.00100062	*MCOLN3*

3	59075001	59125000	0.00100062	*MCOLN2*
9	74125001	74175000	0.00100061	*PDE7B*
3	62525001	62575000	0.00100059	*ADGRL2*
15	29500001	29550000	0.00100048	*BCL9L*
15	29500001	29550000	0.00100048	*UPK2*
17	45325001	45375000	0.00100016	*SFSWAP*
8	75975001	76025000	0.00100016	*DCTN3*
8	75975001	76025000	0.00100016	*IL11RA*
8	75975001	76025000	0.00100016	*ARID3C*
8	75975001	76025000	0.00100016	*GALT*
8	75975001	76025000	0.00100016	*SIGMAR1*
8	75975001	76025000	0.00100016	*RPP25L*
11	30650001	30700000	0.00100007	*FOXN2*
1	84100001	84150000	0.000999982	*ATP11B*
3	20125001	20175000	0.000999738	*ADAMTSL4*
3	20125001	20175000	0.000999738	*MCL1*
14	34350001	34400000	0.000999713	*XKR9*
14	34350001	34400000	0.000999713	*LACTB2*
9	65300001	65350000	0.000999676	*MRAP2*
7	20475001	20525000	0.000999581	*NFIC*
9	41900001	41950000	0.000999415	*SNX3*
9	41900001	41950000	0.000999415	*NR2E1*
15	73900001	73950000	0.000999398	*HSD17B12*
15	73900001	73950000	0.000999398	*U6*
8	28800001	28850000	0.000999334	*PSIP1*
8	28800001	28850000	0.000999334	*SNAPC3*
12	32300001	32350000	0.000999287	*LNX2*
12	32300001	32350000	0.000999287	*POLR1D*
19	12575001	12625000	0.00099892	*USP32*
1	64600001	64650000	0.000998739	*NR1I2*
1	64600001	64650000	0.000998739	*GSK3B*
15	40350001	40400000	0.000998727	*MICAL2*
9	104000001	104050000	0.00099823	*FAM120B*
14	550001	600000	0.000998218	*ADCK5*
14	550001	600000	0.000998218	*CPSF1*
14	550001	600000	0.000998218	*SCRT1*
14	550001	600000	0.000998218	*FBXL6*
14	550001	600000	0.000998218	*SLC52A2*
14	550001	600000	0.000998218	*TMEM249*
25	41825001	41875000	0.000997785	*SUN1*
7	47575001	47625000	0.000997724	*SMAD5*
2	119675001	119725000	0.000997557	*DIS3L2*
10	86175001	86225000	0.000997479	*PGF*
10	38025001	38075000	0.00099734	*TTBK2*
10	38025001	38075000	0.00099734	*CDAN1*
12	19425001	19475000	0.000997335	*TRIM13*
12	19425001	19475000	0.000997335	*KCNRG*
12	19425001	19475000	0.000997335	*SPRYD7*
1	132850001	132900000	0.000997306	*STAG1*
1	81275001	81325000	0.000997243	*TRA2B*
22	13875001	13925000	0.00099723	*ULK4*
3	105025001	105075000	0.00099722	*SCMH1*

7	43900001	43950000	0.000997014	*ADAMTSL5*
7	43900001	43950000	0.000997014	*MEX3D*
7	43900001	43950000	0.000997014	*REEP6*
7	18650001	18700000	0.000996929	*SAFB2*
7	18650001	18700000	0.000996929	*SAFB*
10	30975001	31025000	0.000996818	*DPH6*
11	95350001	95400000	0.000996803	*NEK6*
5	56175001	56225000	0.000996736	*R3HDM2*
5	56175001	56225000	0.000996736	*NXPH4*
5	56175001	56225000	0.000996736	*STAC3*
5	56175001	56225000	0.000996736	*SHMT2*
5	56175001	56225000	0.000996736	*NDUFA4L2*
5	107575001	107625000	0.000996718	*WNK1*
11	74925001	74975000	0.000996407	*FAM228B*
11	74925001	74975000	0.000996407	*FAM228A*
11	74925001	74975000	0.000996407	*PFN4*
29	46000001	46050000	0.000996265	*PPP6R3*
7	51450001	51500000	0.00099614	*PFDN1*
3	95575001	95625000	0.000996075	*FAF1*
8	59725001	59775000	0.000996015	*RUSC2*
8	77025001	77075000	0.000996013	*GKAP1*
9	59700001	59750000	0.000995846	*MAP3K7*
3	109525001	109575000	0.000995615	*THRAP3*
3	109525001	109575000	0.000995615	*SH3D21*
3	109525001	109575000	0.000995615	*STK40*
3	109525001	109575000	0.000995615	*EVA1B*
8	59200001	59250000	0.000995449	*PHF24*
20	31250001	31300000	0.000995342	*PAIP1*
2	106825001	106875000	0.000995284	*WNT10A*
2	106825001	106875000	0.000995284	*WNT6*
20	14100001	14150000	0.000995164	*ADAMTS6*
18	29750001	29800000	0.000995164	*CDH8*
5	56775001	56825000	0.000994922	*BAZ2A*
5	56775001	56825000	0.000994922	*PTGES3*
5	56775001	56825000	0.000994922	*ATP5F1B*
5	56775001	56825000	0.000994922	*SNORD59A*
8	61175001	61225000	0.000994914	*ZCCHC7*
8	61175001	61225000	0.000994914	*PAX5*
9	86600001	86650000	0.00099447	*TAB2*
7	22775001	22825000	0.000994322	*FNIP1*
7	22775001	22825000	0.000994322	*U6*
2	71675001	71725000	0.000994297	*PTPN4*
10	51450001	51500000	0.000994276	*MINDY2*
10	59625001	59675000	0.000994256	*TRPM7*
10	59625001	59675000	0.000994256	*SPPL2A*
22	30475001	30525000	0.000994191	*FOXP1*
18	26300001	26350000	0.000994126	*CNOT1*
18	26300001	26350000	0.000994126	*SETD6*
18	26300001	26350000	0.000994126	*SNORA50A*
18	26300001	26350000	0.000994126	*SNORA50A*
7	43825001	43875000	0.000994102	*APC2*
7	43825001	43875000	0.000994102	*DAZAP1*

7	43825001	43875000	0.000994102	*RPS15*
1	158325001	158375000	0.000994024	*KHDRBS1*
3	102075001	102125000	0.000993957	*IPO13*
3	102075001	102125000	0.000993957	*B4GALT2*
3	102075001	102125000	0.000993957	*DPH2*
3	102075001	102125000	0.000993957	*ATP6V0B*
3	102075001	102125000	0.000993957	*ARTN*
17	29875001	29925000	0.000993605	*SLC25A31*
17	29875001	29925000	0.000993605	*INTU*
17	29875001	29925000	0.000993605	*HSPA4L*
24	34625001	34675000	0.000993496	*ESCO1*
23	10125001	10175000	0.000993416	*BRPF3*
6	72450001	72500000	0.000993413	*IGFBP7*
5	107650001	107700000	0.000993184	*WNK1*
21	30550001	30600000	0.00099306	*ACSBG1*
21	30550001	30600000	0.00099306	*IDH3A*
4	95100001	95150000	0.00099243	*MKLN1*
6	102075001	102125000	0.000992424	*KLHL8*
7	14800001	14850000	0.000992384	*ICAM1*
7	14800001	14850000	0.000992384	*ICAM5*
7	14800001	14850000	0.000992384	*FDX2*
7	14800001	14850000	0.000992384	*RAVER1*
7	14800001	14850000	0.000992384	*ZGLP1*
7	14800001	14850000	0.000992384	*ICAM4*
8	38450001	38500000	0.000992056	*IL33*
17	67800001	67850000	0.000991954	*TTC28*
1	78900001	78950000	0.000991894	*LPP*
14	34375001	34425000	0.000991846	*XKR9*
6	70925001	70975000	0.000991698	*CLOCK*
1	84025001	84075000	0.000991466	*DCUN1D1*
11	95425001	95475000	0.000991362	*PSMB7*
26	31450001	31500000	0.000991268	*SHOC2*
26	31450001	31500000	0.000991268	*BBIP1*
26	31450001	31500000	0.000991268	*PDCD4*
10	86125001	86175000	0.000991216	*DLST*
10	86125001	86175000	0.000991216	*RPS6KL1*
6	99575001	99625000	0.000990981	*WDFY3*
22	10725001	10775000	0.000990867	*DCLK3*
14	28150001	28200000	0.000990495	*YTHDF3*
15	41825001	41875000	0.000990305	*EIF4G2*
5	56650001	56700000	0.000990095	*HSD17B6*
14	81800001	81850000	0.000990012	*MTBP*
14	81800001	81850000	0.000990012	*MRPL13*
19	27475001	27525000	0.000989826	*DNAH2*
8	77050001	77100000	0.000989741	*KIF27*
8	77050001	77100000	0.000989741	*GKAP1*
9	60550001	60600000	0.000989738	*ANKRD6*
9	60550001	60600000	0.000989738	*LYRM2*
9	60550001	60600000	0.000989738	*MDN1*
20	41475001	41525000	0.000989679	*PDZD2*
20	41475001	41525000	0.000989679	*GOLPH3*
8	39350001	39400000	0.000989642	*JAK2*

8	39350001	39400000	0.000989642	*INSL6*
3	59500001	59550000	0.000989593	*SPATA1*
3	59500001	59550000	0.000989593	*CTBS*
1	84050001	84100000	0.000989567	*DCUN1D1*
22	50000001	50050000	0.000989354	*HYAL2*
22	50000001	50050000	0.000989354	*IFRD2*
22	50000001	50050000	0.000989354	*RASSF1*
22	50000001	50050000	0.000989354	*HYAL3*
22	50000001	50050000	0.000989354	*ZMYND10*
22	50000001	50050000	0.000989354	*HYAL1*
22	50000001	50050000	0.000989354	*NPRL2*
22	50000001	50050000	0.000989354	*NAA80*
15	77100001	77150000	0.000989121	*DDB2*
15	77100001	77150000	0.000989121	*NR1H3*
15	77100001	77150000	0.000989121	*MADD*
15	77100001	77150000	0.000989121	*ACP2*
11	95050001	95100000	0.000988886	*DENND1A*
13	31825001	31875000	0.000988658	*ST8SIA6*
12	19475001	19525000	0.000988397	*MIR15A*
25	9175001	9225000	0.000988379	*ATF7IP2*
25	9175001	9225000	0.000988379	*U6*
16	38200001	38250000	0.000988053	*PRRX1*
10	57775001	57825000	0.000988037	*FAM214A*
10	57775001	57825000	0.000988037	*ARPP19*
3	20700001	20750000	0.000987955	*SV2A*
3	20700001	20750000	0.000987955	*H2AC20*
3	20700001	20750000	0.000987955	*BOLA1*
3	20700001	20750000	0.000987955	*H2AC19*
3	20700001	20750000	0.000987955	*H2AC21*
17	73000001	73050000	0.000987916	*ZDHHC8*
17	73000001	73050000	0.000987916	*CCDC188*
17	73000001	73050000	0.000987916	*RANBP1*
13	33925001	33975000	0.000987815	*ZEB1*
19	26775001	26825000	0.000987798	*ALOX12*
19	26775001	26825000	0.000987798	*BCL6B*
19	26775001	26825000	0.000987798	*C19H17orf49*
19	26775001	26825000	0.000987798	*SLC16A13*
19	26775001	26825000	0.000987798	*RNASEK*
3	33475001	33525000	0.000987556	*CSF1*
4	79275001	79325000	0.000987502	*INHBA*
18	15575001	15625000	0.000987424	*ITFG1*
11	68400001	68450000	0.000987363	*PCBP1*
1	69750001	69800000	0.000987151	*ZNF148*
10	42600001	42650000	0.000987089	*LRR1*
10	42600001	42650000	0.000987089	*RPS29*
10	42600001	42650000	0.000987089	*Metazoa_SRP*
12	32325001	32375000	0.000986859	*LNX2*
17	67775001	67825000	0.00098673	*TTC28*
3	87475001	87525000	0.000986693	*OMA1*
2	52150001	52200000	0.000986607	*ZEB2*
8	37800001	37850000	0.000986055	*KDM4C*
3	110200001	110250000	0.000985941	*KIAA0319L*

3	110200001	110250000	0.000985941	*NCDN*
10	53950001	54000000	0.000985828	*MNS1*
10	53950001	54000000	0.000985828	*TEX9*
2	89600001	89650000	0.000985779	*FAM126B*
2	89600001	89650000	0.000985779	*U6*
11	95675001	95725000	0.000985354	*NR6A1*
12	34600001	34650000	0.000985271	*SACS*
9	42050001	42100000	0.000984922	*OSTM1*
11	40700001	40750000	0.000984889	*VRK2*
6	84100001	84150000	0.000984723	*YTHDC1*
1	64925001	64975000	0.000984374	*GPR156*
2	85425001	85475000	0.000984355	*PGAP1*
3	21525001	21575000	0.000984297	*RNF115*
3	21525001	21575000	0.000984297	*POLR3C*
17	69300001	69350000	0.000984237	*TBC1D10A*
17	69300001	69350000	0.000984237	*CASTOR1*
17	69300001	69350000	0.000984237	*SF3A1*
13	23450001	23500000	0.00098423	*SPAG6*
18	55050001	55100000	0.000984144	*TMEM143*
18	55050001	55100000	0.000984144	*SYNGR4*
18	55050001	55100000	0.000984144	*KDELR1*
18	55050001	55100000	0.000984144	*GRIN2D*
25	41725001	41775000	0.000984099	*C25H7orf50*
25	41725001	41775000	0.000984099	*COX19*
25	41725001	41775000	0.000984099	*CYP2W1*
25	41725001	41775000	0.000984099	*ADAP1*
25	41725001	41775000	0.000984099	*GPR146*
11	24625001	24675000	0.000984072	*EML4*
2	36525001	36575000	0.000984012	*CD302*
2	36525001	36575000	0.000984012	*LY75*
4	29025001	29075000	0.000983992	*ITGB8*
13	22900001	22950000	0.000983917	*MLLT10*
7	80000001	80050000	0.000983798	*TENM2*
17	67625001	67675000	0.000983763	*TTC28*
20	23675001	23725000	0.000983709	*PLPP1*
2	107325001	107375000	0.000983578	*SPEG*
2	107325001	107375000	0.000983578	*DES*
23	28350001	28400000	0.000983554	*PPP1R10*
23	28350001	28400000	0.000983554	*ATAT1*
23	28350001	28400000	0.000983554	*MRPS18B*
23	28350001	28400000	0.000983554	*ABCF1*
23	28350001	28400000	0.000983554	*C23H6orf136*
1	148375001	148425000	0.000983522	*MGC127133*
26	15975001	16025000	0.000983436	*TBC1D12*
26	15975001	16025000	0.000983436	*HELLS*
7	54075001	54125000	0.000983242	*ARHGAP26*
5	70150001	70200000	0.000983067	*RIC8B*
11	94950001	95000000	0.000982648	*DENND1A*
27	33500001	33550000	0.000982507	*NSD3*
27	33500001	33550000	0.000982507	*LETM2*
27	33500001	33550000	0.000982507	*FGFR1*
17	53450001	53500000	0.000982455	*SETD1B*

17	53450001	53500000	0.000982455	*HPD*
2	126400001	126450000	0.000982429	*ARID1A*
2	126400001	126450000	0.000982429	*PIGV*
3	82100001	82150000	0.000982375	*ITGB3BP*
3	82100001	82150000	0.000982375	*ALG6*
5	43175001	43225000	0.000982252	*CNOT2*
13	57525001	57575000	0.000982204	*GNAS*
19	26975001	27025000	0.000982165	*YBX2*
19	26975001	27025000	0.000982165	*SLC2A4*
19	26975001	27025000	0.000982165	*EIF5A*
19	26975001	27025000	0.000982165	*GPS2*
19	26975001	27025000	0.000982165	*NEURL4*
1	57400001	57450000	0.000982035	*CCDC80*
28	13925001	13975000	0.000981868	*BICC1*
2	106850001	106900000	0.000981771	*WNT10A*
2	106850001	106900000	0.000981771	*CDK5R2*
21	25975001	26025000	0.00098158	*BCL2A1*
14	34975001	35025000	0.000981552	*EYA1*
3	50075001	50125000	0.000981513	*DR1*
19	40100001	40150000	0.000981503	*IKZF3*
19	40100001	40150000	0.000981503	*GRB7*
3	21500001	21550000	0.000981468	*RNF115*
3	21500001	21550000	0.000981468	*POLR3C*
3	21500001	21550000	0.000981468	*PIAS3*
3	21500001	21550000	0.000981468	*NUDT17*
16	43075001	43125000	0.000981326	*PEX14*
16	43075001	43125000	0.000981326	*DFFA*
5	62700001	62750000	0.000981283	*TMPO*
5	62700001	62750000	0.000981283	*SLC25A3*
15	20450001	20500000	0.00098124	*FDX1*
22	48450001	48500000	0.000981209	*PHF7*
22	48450001	48500000	0.000981209	*DNAH1*
22	48450001	48500000	0.000981209	*BAP1*
22	48450001	48500000	0.000981209	*SEMA3G*
18	20950001	21000000	0.000981069	*TOX3*
11	72550001	72600000	0.000981015	*AGBL5*
11	72550001	72600000	0.000981015	*EMILIN1*
11	72550001	72600000	0.000981015	*TMEM214*
29	12425001	12475000	0.000980912	*ANKRD42*
2	20825001	20875000	0.000980197	*EVX2*
3	69075001	69125000	0.000980148	*ACADM*
13	50700001	50750000	0.000979863	*HAO1*
24	45925001	45975000	0.000979651	*C24H18orf25*
5	69750001	69800000	0.000979642	*POLR3B*
18	36225001	36275000	0.00097953	*TANGO6*
5	26600001	26650000	0.000979456	*SP1*
6	102050001	102100000	0.000979369	*KLHL8*
3	83975001	84025000	0.000979181	*TM2D1*
26	44475001	44525000	0.000978612	*ZRANB1*
19	43150001	43200000	0.000978553	*NBR1*
19	43150001	43200000	0.000978553	*TMEM106A*
19	43150001	43200000	0.000978553	*U2*

22	11525001	11575000	0.000978552	*DLEC1*
18	8000001	8050000	0.000978246	*CMIP*
9	74675001	74725000	0.000978235	*MAP3K5*
13	33200001	33250000	0.000978187	*EPC1*
1	136950001	137000000	0.000978026	*ACAD11*
1	136950001	137000000	0.000978026	*ACKR4*
1	82625001	82675000	0.000977669	*EPHB3*
10	49550001	49600000	0.000977567	*RORA*
16	44950001	45000000	0.000977505	*RERE*
11	43725001	43775000	0.00097741	*PUS10*
11	43725001	43775000	0.00097741	*PEX13*
13	46825001	46875000	0.000977384	*ZMYND11*
4	46350001	46400000	0.000977361	*SRPK2*
7	43650001	43700000	0.000977352	*STK11*
7	43650001	43700000	0.000977352	*MIDN*
7	43650001	43700000	0.000977352	*CBARP*
7	43650001	43700000	0.000977352	*ATP5F1D*
14	35075001	35125000	0.000977301	*EYA1*
12	19500001	19550000	0.000977126	*MIR15A*
3	105050001	105100000	0.000976246	*SCMH1*
7	46100001	46150000	0.000976142	*JADE2*
7	46100001	46150000	0.000976142	*SAR1B*
7	46100001	46150000	0.000976142	*U6*
3	20625001	20675000	0.000976061	*OTUD7B*
3	20625001	20675000	0.000976061	*MTMR11*
8	38475001	38525000	0.000975506	*IL33*
29	9525001	9575000	0.000975419	*PICALM*
1	70025001	70075000	0.000975402	*OSBPL11*
3	94400001	94450000	0.000975338	*NRDC*
3	94400001	94450000	0.000975338	*RAB3B*
22	33950001	34000000	0.000975148	*SUCLG2*
11	43700001	43750000	0.000975097	*PUS10*
21	6025001	6075000	0.00097506	*LINS1*
1	69650001	69700000	0.000975035	*SLC12A8*
1	69650001	69700000	0.000975035	*ZNF148*
3	56675001	56725000	0.000974788	*LMO4*
20	71625001	71675000	0.000974668	*EXOC3*
20	71625001	71675000	0.000974668	*SLC9A3*
13	31850001	31900000	0.000974593	*ST8SIA6*
17	29825001	29875000	0.000974531	*HSPA4L*
2	90625001	90675000	0.000974494	*KIAA2012*
3	34100001	34150000	0.000974313	*CELSR2*
3	34100001	34150000	0.000974313	*MYBPHL*
3	34100001	34150000	0.000974313	*PSRC1*
3	34100001	34150000	0.000974313	*SORT1*
13	17875001	17925000	0.000974172	*ABI1*
13	17875001	17925000	0.000974172	*PDSS1*
16	61400001	61450000	0.000974036	*LHX4*
16	61400001	61450000	0.000974036	*QSOX1*
25	26425001	26475000	0.000973958	*KIF22*
25	26425001	26475000	0.000973958	*PAGR1*
25	26425001	26475000	0.000973958	*MAZ*

25	26425001	26475000	0.000973958	*PRRT2*
25	26425001	26475000	0.000973958	*U6*
16	41825001	41875000	0.000973539	*CLCN6*
16	41825001	41875000	0.000973539	*MTHFR*
16	41825001	41875000	0.000973539	*NPPA*
21	32450001	32500000	0.000973349	*PEAK1*
21	1900001	1950000	0.00097326	*SNRPN*
16	51225001	51275000	0.000972982	*UBE2J2*
16	51225001	51275000	0.000972982	*SCNN1D*
16	51225001	51275000	0.000972982	*SDF4*
16	51225001	51275000	0.000972982	*ACAP3*
16	51225001	51275000	0.000972982	*C1QTNF12*
16	51225001	51275000	0.000972982	*B3GALT6*
9	50275001	50325000	0.000972842	*USP45*
9	50275001	50325000	0.000972842	*TSTD3*
13	60700001	60750000	0.000972834	*C13H20orf96*
13	60700001	60750000	0.000972834	*ZCCHC3*
10	86450001	86500000	0.000972753	*FOS*
29	45225001	45275000	0.000972707	*CLCF1*
29	45225001	45275000	0.000972707	*SSH3*
29	45225001	45275000	0.000972707	*POLD4*
7	3975001	4025000	0.000972429	*SUGP1*
7	3975001	4025000	0.000972429	*TM6SF2*
7	3975001	4025000	0.000972429	*HAPLN4*
7	3975001	4025000	0.000972429	*NCAN*
15	36175001	36225000	0.000972359	*SOX6*
10	72450001	72500000	0.000972279	*PPM1A*
21	45825001	45875000	0.00097183	*RALGAPA1*
3	53225001	53275000	0.000971554	*ZNF326*
11	95000001	95050000	0.000971118	*DENND1A*
21	22425001	22475000	0.00097091	*ZNF592*
21	22425001	22475000	0.00097091	*ALPK3*
4	55300001	55350000	0.000970814	*GPR85*
18	36850001	36900000	0.000970629	*WWP2*
24	48925001	48975000	0.000970504	*DYM*
10	49525001	49575000	0.000970484	*RORA*
8	38100001	38150000	0.000970368	*KDM4C*
13	42975001	43025000	0.000969989	*TASOR2*
13	51825001	51875000	0.000969984	*DNAAF9*
6	68300001	68350000	0.000969739	*USP46*
3	20100001	20150000	0.000969542	*ADAMTSL4*
3	20100001	20150000	0.000969542	*MCL1*
3	20100001	20150000	0.000969542	*ENSA*
3	57700001	57750000	0.000969443	*ODF2L*
13	38575001	38625000	0.000969293	*DTD1*
13	38575001	38625000	0.000969293	*SEC23B*
13	38575001	38625000	0.000969293	*SMIM26*
8	59550001	59600000	0.000969124	*UNC13B*
2	91125001	91175000	0.00096886	*ICA1L*
2	91125001	91175000	0.00096886	*FAM117B*
5	75950001	76000000	0.000968851	*USP18*
5	75950001	76000000	0.000968851	*U6*

10	60800001	60850000	0.000968678	*FGF7*
1	139550001	139600000	0.000968534	*SH3BGR*
1	139550001	139600000	0.000968534	*U6*
18	53050001	53100000	0.000968518	*RTN2*
18	53050001	53100000	0.000968518	*FOSB*
18	53050001	53100000	0.000968518	*PPM1N*
4	112600001	112650000	0.000968473	*SSPO*
4	112600001	112650000	0.000968473	*ZNF467*
23	9175001	9225000	0.000968446	*SCUBE3*
5	60800001	60850000	0.000967683	*CFAP54*
10	25700001	25750000	0.000967644	*CHD8*
10	25700001	25750000	0.000967644	*RAB2B*
26	31475001	31525000	0.000967482	*SHOC2*
26	31475001	31525000	0.000967482	*BBIP1*
6	6075001	6125000	0.000967473	*MYOZ2*
6	83150001	83200000	0.000967343	*CENPC*
8	75400001	75450000	0.000967221	*UBAP2*
7	22700001	22750000	0.000967197	*FNIP1*
2	22800001	22850000	0.000967149	*SP3*
5	119900001	119950000	0.000966896	*MAPK8IP2*
5	119900001	119950000	0.000966896	*ARSA*
5	119900001	119950000	0.000966896	*CHKB*
2	35150001	35200000	0.000966742	*TANK*
3	84400001	84450000	0.000966727	*NFIA*
9	42475001	42525000	0.000966682	*SOBP*
22	55950001	56000000	0.000966418	*TMCC1*
12	35550001	35600000	0.000966418	*ZDHHC20*
12	35550001	35600000	0.000966418	*SKA3*
12	35550001	35600000	0.000966418	*MRPL57*
4	46275001	46325000	0.000966374	*SRPK2*
4	46275001	46325000	0.000966374	*KMT2E*
11	43500001	43550000	0.000966221	*PAPOLG*
3	94550001	94600000	0.000966175	*OSBPL9*
11	95625001	95675000	0.000966091	*NR6A1*
25	1575001	1625000	0.000966013	*TSC2*
25	1575001	1625000	0.000966013	*SLC9A3R2*
25	1575001	1625000	0.000966013	*NTHL1*
13	57200001	57250000	0.000965604	*ZNF831*
12	32350001	32400000	0.000965296	*LNX2*
7	7700001	7750000	0.000965221	*WIZ*
7	7700001	7750000	0.000965221	*AKAP8L*
7	7700001	7750000	0.000965221	*RASAL3*
3	95500001	95550000	0.000965209	*FAF1*
7	43325001	43375000	0.000965153	*MISP*
7	43325001	43375000	0.000965153	*PTBP1*
3	34275001	34325000	0.000965144	*TAF13*
3	34275001	34325000	0.000965144	*CFAP276*
3	34275001	34325000	0.000965144	*TMEM167B*
3	34275001	34325000	0.000965144	*SCARNA2*
2	44325001	44375000	0.000965056	*CACNB4*
2	44325001	44375000	0.000965056	*ARL5A*
8	6925001	6975000	0.000964975	*GLRA3*

2	27525001	27575000	0.000964785	*CERS6*
29	9500001	9550000	0.000964348	*PICALM*
7	625001	675000	0.000963811	*CNOT6*
1	132250001	132300000	0.000963354	*NCK1*
1	132250001	132300000	0.000963354	*SLC35G2*
19	50875001	50925000	0.000963287	*ASPSCR1*
19	50875001	50925000	0.000963287	*LRRC45*
19	50875001	50925000	0.000963287	*RAC3*
19	50875001	50925000	0.000963287	*CENPX*
19	50875001	50925000	0.000963287	*DCXR*
13	60275001	60325000	0.000963285	*SLC52A3*
3	63000001	63050000	0.000963239	*ADGRL2*
23	18475001	18525000	0.000963164	*SUPT3H*
9	50150001	50200000	0.000963039	*PRDM13*
16	50975001	51025000	0.000962859	*CDK11B*
16	50975001	51025000	0.000962859	*MIB2*
16	50975001	51025000	0.000962859	*SLC35E2*
16	50975001	51025000	0.000962859	*MMP23*
7	7875001	7925000	0.000962437	*NOTCH3*
7	7875001	7925000	0.000962437	*BRD4*
7	7875001	7925000	0.000962437	*EPHX3*
10	20475001	20525000	0.000961518	*CD276*
19	35000001	35050000	0.000961097	*MPRIP*
15	42500001	42550000	0.00096105	*SBF2*
3	34075001	34125000	0.000960641	*SORT1*
3	34075001	34125000	0.000960641	*MYBPHL*
3	34075001	34125000	0.000960641	*PSRC1*
10	20375001	20425000	0.000960493	*NPTN*
7	44400001	44450000	0.000960203	*SHROOM1*
7	44400001	44450000	0.000960203	*GDF9*
7	44400001	44450000	0.000960203	*SOWAHA*
7	44400001	44450000	0.000960203	*UQCRQ*
1	72875001	72925000	0.000960116	*LSG1*
1	72875001	72925000	0.000960116	*TMEM44*
8	79450001	79500000	0.000959945	*TUT7*
8	79450001	79500000	0.000959945	*ISCA1*
17	61850001	61900000	0.000959795	*HECTD4*
3	32725001	32775000	0.000959735	*KCNA3*
2	58600001	58650000	0.000959732	*SPOPL*
3	20650001	20700000	0.000959508	*OTUD7B*
3	20650001	20700000	0.000959508	*MTMR11*
3	20650001	20700000	0.000959508	*SF3B4*
3	20650001	20700000	0.000959508	*SV2A*
29	45250001	45300000	0.000959399	*CLCF1*
29	45250001	45300000	0.000959399	*RAD9A*
29	45250001	45300000	0.000959399	*POLD4*
29	45250001	45300000	0.000959399	*TBC1D10C*
29	45250001	45300000	0.000959399	*PPP1CA*
12	26200001	26250000	0.000959191	*NBEA*
5	26675001	26725000	0.000959184	*AAAS*
5	26675001	26725000	0.000959184	*SP7*
5	26675001	26725000	0.000959184	*MYG1*

5	26675001	26725000	0.000959184	*PFDN5*
10	51225001	51275000	0.000959171	*RNF111*
10	72875001	72925000	0.000959115	*MNAT1*
10	72875001	72925000	0.000959115	*SIX4*
24	48550001	48600000	0.000958854	*SMAD7*
2	36775001	36825000	0.000958825	*BAZ2B*
14	8500001	8550000	0.000958789	*PHF20L1*
26	21775001	21825000	0.00095874	*SLF2*
26	21775001	21825000	0.00095874	*SEMA4G*
26	21775001	21825000	0.00095874	*MRPL43*
16	64525001	64575000	0.000958272	*SMG7*
13	38500001	38550000	0.000958197	*DZANK1*
13	38500001	38550000	0.000958197	*POLR3F*
13	38500001	38550000	0.000958197	*RBBP9*
7	20600001	20650000	0.000957945	*CELF5*
7	20600001	20650000	0.000957945	*NCLN*
8	77075001	77125000	0.00095792	*KIF27*
7	43625001	43675000	0.000957764	*STK11*
7	43625001	43675000	0.000957764	*SBNO2*
7	43625001	43675000	0.000957764	*CBARP*
15	17625001	17675000	0.000957618	*CUL5*
17	72975001	73025000	0.000957357	*DGCR8*
17	72975001	73025000	0.000957357	*ZDHHC8*
17	72975001	73025000	0.000957357	*CCDC188*
17	72975001	73025000	0.000957357	*RANBP1*
17	72975001	73025000	0.000957357	*TRMT2A*
17	72975001	73025000	0.000957357	*MIR3618*
17	72975001	73025000	0.000957357	*MIR1306*
4	46400001	46450000	0.000956982	*SRPK2*
8	39025001	39075000	0.000956792	*RIC1*
7	17050001	17100000	0.000956732	*HNRNPM*
7	17050001	17100000	0.000956732	*MARCHF2*
7	17050001	17100000	0.000956732	*PRAM1*
25	28675001	28725000	0.00095659	*CALN1*
3	34575001	34625000	0.000956329	*STXBP3*
5	30300001	30350000	0.000956009	*SPATS2*
18	34575001	34625000	0.000955882	*CDH16*
18	34575001	34625000	0.000955882	*PDP2*
25	2300001	2350000	0.00095566	*FLYWCH1*
25	2300001	2350000	0.00095566	*FLYWCH2*
25	2300001	2350000	0.00095566	*SRRM2*
10	42625001	42675000	0.000955597	*LRR1*
10	42625001	42675000	0.000955597	*DNAAF2*
10	42625001	42675000	0.000955597	*RPS29*
10	42625001	42675000	0.000955597	*MGAT2*
10	42625001	42675000	0.000955597	*POLE2*
10	42625001	42675000	0.000955597	*RPL36AL*
10	42625001	42675000	0.000955597	*Metazoa_SRP*
15	82400001	82450000	0.000955548	*OR5AN2C*
15	82400001	82450000	0.000955548	*OR5AN1L*
7	49675001	49725000	0.000955536	*CDC25C*
7	49675001	49725000	0.000955536	*GFRA3*

8	38175001	38225000	0.000955507	*GLDC*
1	132875001	132925000	0.0009551	*STAG1*
1	132875001	132925000	0.0009551	*PCCB*
1	75200001	75250000	0.000954614	*FGF12*
1	106650001	106700000	0.000954392	*PPM1L*
10	59600001	59650000	0.00095405	*SPPL2A*
10	59600001	59650000	0.00095405	*TRPM7*
2	71875001	71925000	0.000953562	*EPB41L5*
9	73450001	73500000	0.000953515	*AHI1*
15	51375001	51425000	0.000953276	*ART1*
6	67950001	68000000	0.000953264	*SPATA18*
2	47425001	47475000	0.000953087	*EPC2*
25	18575001	18625000	0.000952996	*REXO5*
15	84350001	84400000	0.000952876	*OR4A2I*
23	10075001	10125000	0.000952483	*BRPF3*
18	250001	300000	0.00095238	*OR4P4*
24	48975001	49025000	0.000952184	*DYM*
3	108375001	108425000	0.000952125	*MEAF6*
3	108375001	108425000	0.000952125	*ZC3H12A*
10	36175001	36225000	0.00095211	*KNL1*
13	46800001	46850000	0.00095198	*DIP2C*
13	46800001	46850000	0.00095198	*ZMYND11*
9	48250001	48300000	0.000951911	*GRIK2*
3	34675001	34725000	0.000951467	*HENMT1*
3	34675001	34725000	0.000951467	*PRPF38B*
3	95600001	95650000	0.000951428	*FAF1*
1	57600001	57650000	0.000951009	*CD200R1L*
7	50850001	50900000	0.000950992	*CXXC5*
20	26200001	26250000	0.000950858	*ITGA1*
3	20675001	20725000	0.000950519	*SV2A*
3	20675001	20725000	0.000950519	*MTMR11*
3	20675001	20725000	0.000950519	*H2AC20*
3	20675001	20725000	0.000950519	*SF3B4*
3	20675001	20725000	0.000950519	*BOLA1*
3	20675001	20725000	0.000950519	*H2AC21*
11	86525001	86575000	0.000950467	*ROCK2*
2	71825001	71875000	0.000950281	*EPB41L5*
2	71825001	71875000	0.000950281	*U4*
13	22800001	22850000	0.000950173	*MLLT10*
8	79200001	79250000	0.000949986	*NAA35*
22	50925001	50975000	0.000949926	*QRICH1*
22	50925001	50975000	0.000949926	*IMPDH2*
22	50925001	50975000	0.000949926	*DALRD3*
22	50925001	50975000	0.000949926	*NDUFAF3*
22	50925001	50975000	0.000949926	*MIR191*
9	62725001	62775000	0.000949882	*ZNF292*
3	15575001	15625000	0.000949689	*ZBTB7B*
3	15575001	15625000	0.000949689	*SHC1*
3	15575001	15625000	0.000949689	*FLAD1*
3	15575001	15625000	0.000949689	*CKS1B*
9	33350001	33400000	0.000949526	*ROS1*
3	87525001	87575000	0.000949143	*OMA1*

9	43750001	43800000	0.000949115	*ATG5*
9	62600001	62650000	0.000948504	*ZNF292*
6	49925001	49975000	0.000948491	*PCDH7*
1	64625001	64675000	0.00094848	*GSK3B*
1	64625001	64675000	0.00094848	*NR1I2*
10	20975001	21025000	0.000948354	*PSME1*
10	20975001	21025000	0.000948354	*PSME2*
10	20975001	21025000	0.000948354	*RNF31*
10	20975001	21025000	0.000948354	*IRF9*
10	20975001	21025000	0.000948354	*REC8*
10	20975001	21025000	0.000948354	*DCAF11*
10	20975001	21025000	0.000948354	*FITM1*
10	20975001	21025000	0.000948354	*EMC9*
10	20975001	21025000	0.000948354	*IPO4*
17	54175001	54225000	0.000948241	*IFT81*
17	54175001	54225000	0.000948241	*ATP2A2*
2	32725001	32775000	0.000948218	*FIGN*
13	38625001	38675000	0.000947457	*DTD1*
6	99600001	99650000	0.000947232	*WDFY3*
15	84100001	84150000	0.000947182	*OR4A47WP*
2	90600001	90650000	0.000947154	*KIAA2012*
19	27300001	27350000	0.000947108	*FXR2*
19	27300001	27350000	0.000947108	*EIF4A1*
19	27300001	27350000	0.000947108	*SENP3*
19	27300001	27350000	0.000947108	*SHBG*
19	27300001	27350000	0.000947108	*MPDU1*
19	27300001	27350000	0.000947108	*CD68*
19	27300001	27350000	0.000947108	*SOX15*
19	27300001	27350000	0.000947108	*SAT2*
19	27300001	27350000	0.000947108	*SNORD10*
19	27300001	27350000	0.000947108	*SNORA48*
4	95125001	95175000	0.000947072	*MKLN1*
20	37250001	37300000	0.000947031	*NIPBL*
14	30850001	30900000	0.000947013	*VCPIP1*
14	30850001	30900000	0.000947013	*MYBL1*
29	40975001	41025000	0.000946951	*BSCL2*
29	40975001	41025000	0.000946951	*GANAB*
29	40975001	41025000	0.000946951	*C29H11orf98*
29	40975001	41025000	0.000946951	*INTS5*
29	40975001	41025000	0.000946951	*UBXN1*
29	40975001	41025000	0.000946951	*LRRN4CL*
29	40975001	41025000	0.000946951	*CSKMT*
29	40975001	41025000	0.000946951	*UQCC3*
29	40975001	41025000	0.000946951	*SNORA57*
29	40975001	41025000	0.000946951	*GNG3*
21	21775001	21825000	0.00094671	*FURIN*
21	21775001	21825000	0.00094671	*FES*
17	72925001	72975000	0.000946648	*TANGO2*
17	72925001	72975000	0.000946648	*ARVCF*
17	72925001	72975000	0.000946648	*MIR185*
5	69700001	69750000	0.000946605	*POLR3B*
5	69700001	69750000	0.000946605	*TCP11L2*

11	24650001	24700000	0.000946561	*EML4*
23	18300001	18350000	0.00094597	*SUPT3H*
1	136925001	136975000	0.000945865	*ACAD11*
2	91650001	91700000	0.000945704	*RAPH1*
23	8375001	8425000	0.000945598	*NUDT3*
5	29425001	29475000	0.000945546	*LARP4*
5	43200001	43250000	0.000945442	*CNOT2*
13	65950001	66000000	0.000945123	*SAMHD1*
7	51475001	51525000	0.0009451	*PFDN1*
12	36225001	36275000	0.000944935	*ZMYM5*
12	36225001	36275000	0.000944935	*PSPC1*
11	68175001	68225000	0.000944847	*GMCL1*
4	69225001	69275000	0.000944829	*SKAP2*
14	375001	425000	0.00094434	*ARHGAP39*
14	375001	425000	0.00094434	*LRRC24*
14	375001	425000	0.00094434	*C14H8orf82*
14	375001	425000	0.00094434	*LRRC14*
19	11100001	11150000	0.000944131	*MED13*
16	27550001	27600000	0.000943705	*WDR26*
19	37850001	37900000	0.000943304	*HOXB9*
19	37850001	37900000	0.000943304	*HOXB7*
19	37850001	37900000	0.000943304	*HOXB8*
19	37850001	37900000	0.000943304	*HOXB6*
19	37850001	37900000	0.000943304	*HOXB5*
13	51800001	51850000	0.000943232	*DNAAF9*
2	125725001	125775000	0.000943097	*AHDC1*
18	50000001	50050000	0.000942553	*ITPKC*
18	50000001	50050000	0.000942553	*SNRPA*
18	50000001	50050000	0.000942553	*RAB4B*
18	50000001	50050000	0.000942553	*C18H19orf54*
18	50000001	50050000	0.000942553	*MIA*
18	50000001	50050000	0.000942553	*EGLN2*
2	91300001	91350000	0.000942505	*NBEAL1*
2	91300001	91350000	0.000942505	*CARF*
21	32575001	32625000	0.000942404	*HMG20A*
13	33950001	34000000	0.000942404	*ZEB1*
5	51175001	51225000	0.000942274	*USP15*
11	74950001	75000000	0.000942142	*FAM228B*
11	74950001	75000000	0.000942142	*SF3B6*
11	74950001	75000000	0.000942142	*TP53I3*
11	74950001	75000000	0.000942142	*PFN4*
10	34100001	34150000	0.000941705	*RASGRP1*
13	54900001	54950000	0.000941688	*LAMA5*
13	54900001	54950000	0.000941688	*ADRM1*
18	54125001	54175000	0.000941669	*ZC3H4*
18	54125001	54175000	0.000941669	*SAE1*
3	50325001	50375000	0.000941449	*MTF2*
11	95300001	95350000	0.000941116	*NEK6*
7	49550001	49600000	0.000940978	*NME5*
7	49550001	49600000	0.000940978	*BRD8*
26	14950001	15000000	0.000940639	*FRA10AC1*
26	14950001	15000000	0.000940639	*PDE6C*

19	27100001	27150000	0.000940601	*ZBTB4*
19	27100001	27150000	0.000940601	*CHRNB1*
19	27100001	27150000	0.000940601	*TMEM102*
19	27100001	27150000	0.000940601	*SPEM2*
19	27100001	27150000	0.000940601	*SLC35G6*
19	27100001	27150000	0.000940601	*SPEM1*
7	50150001	50200000	0.000940068	*CTNNA1*
7	50150001	50200000	0.000940068	*LRRTM2*
26	23100001	23150000	0.00094006	*SUFU*
26	23100001	23150000	0.00094006	*ACTR1A*
16	21725001	21775000	0.000939973	*RRP15*
8	23175001	23225000	0.000939782	*IFNB3*
8	23175001	23225000	0.000939782	*IFNB3*
8	23175001	23225000	0.000939782	*IFNB3*
8	23175001	23225000	0.000939782	*IFNB3*
8	23175001	23225000	0.000939782	*IFNB3*
8	23175001	23225000	0.000939782	*IFNB3*
9	104025001	104075000	0.00093976	*PSMB1*
22	14775001	14825000	0.000939719	*NKTR*
22	14775001	14825000	0.000939719	*ZBTB47*
22	14775001	14825000	0.000939719	*KLHL40*
22	14775001	14825000	0.000939719	*HHATL*
9	74100001	74150000	0.000939594	*PDE7B*
3	87500001	87550000	0.000939184	*OMA1*
11	30750001	30800000	0.000938911	*PPP1R21*
26	21675001	21725000	0.000938835	*PAX2*
26	21675001	21725000	0.000938835	*SNORA70*
18	44575001	44625000	0.000938718	*LSM14A*
1	87875001	87925000	0.000938511	*PIK3CA*
2	20725001	20775000	0.000938446	*HOXD3*
2	20725001	20775000	0.000938446	*HOXD4*
2	20725001	20775000	0.000938446	*HOXD1*
13	38525001	38575000	0.000938294	*SEC23B*
13	38525001	38575000	0.000938294	*POLR3F*
13	38525001	38575000	0.000938294	*DZANK1*
13	38525001	38575000	0.000938294	*RBBP9*
11	73250001	73300000	0.000938104	*HADHA*
11	73250001	73300000	0.000938104	*HADHB*
5	26625001	26675000	0.000938101	*SP1*
8	78950001	79000000	0.000937655	*AGTPBP1*
18	44650001	44700000	0.000937566	*LSM14A*
18	44650001	44700000	0.000937566	*GARRE1*
18	44650001	44700000	0.000937566	*U1*
18	44650001	44700000	0.000937566	*U6*
2	106600001	106650000	0.000937418	*ZNF142*
2	106600001	106650000	0.000937418	*STK36*
2	106600001	106650000	0.000937418	*RNF25*
2	106600001	106650000	0.000937418	*BCS1L*
9	43800001	43850000	0.000937286	*PRDM1*
6	14825001	14875000	0.000937179	*PITX2*
17	67700001	67750000	0.000937008	*TTC28*
7	51550001	51600000	0.000936982	*SLC4A9*

1	132825001	132875000	0.00093693	*STAG1*
8	82100001	82150000	0.000936809	*PTCH1*
1	122250001	122300000	0.000936583	*PLSCR4*
3	58400001	58450000	0.000936351	*ZNHIT6*
4	45200001	45250000	0.000936125	*RELN*
8	61225001	61275000	0.000935956	*ZCCHC7*
16	12175001	12225000	0.000935953	*UCHL5*
16	12175001	12225000	0.000935953	*RO60*
3	50050001	50100000	0.000935822	*DR1*
11	29125001	29175000	0.000935765	*SOCS5*
3	34125001	34175000	0.000935289	*CELSR2*
3	34125001	34175000	0.000935289	*SARS1*
3	34125001	34175000	0.000935289	*PSRC1*
16	44925001	44975000	0.000935219	*RERE*
20	14575001	14625000	0.000935112	*CWC27*
19	48825001	48875000	0.000935069	*SMURF2*
20	14150001	14200000	0.000934994	*ADAMTS6*
5	36200001	36250000	0.000934975	*TMEM117*
3	112475001	112525000	0.00093496	*GIGYF2*
3	112475001	112525000	0.00093496	*KCNJ13*
11	40725001	40775000	0.000934404	*VRK2*
11	30625001	30675000	0.00093438	*FOXN2*
22	49975001	50025000	0.00093345	*NPRL2*
22	49975001	50025000	0.00093345	*CACNA2D2*
22	49975001	50025000	0.00093345	*RASSF1*
22	49975001	50025000	0.00093345	*HYAL2*
22	49975001	50025000	0.00093345	*ZMYND10*
22	49975001	50025000	0.00093345	*CYB561D2*
18	7750001	7800000	0.000933287	*PKD1L2*
18	7750001	7800000	0.000933287	*GCSH*
18	7750001	7800000	0.000933287	*C18H16orf46*
18	7750001	7800000	0.000933287	*U6*
11	74900001	74950000	0.000933238	*FAM228A*
11	74900001	74950000	0.000933238	*FAM228B*
11	29150001	29200000	0.000933228	*SOCS5*
23	31925001	31975000	0.000933158	*SLC17A2*
23	31925001	31975000	0.000933158	*TRIM38*
26	22700001	22750000	0.000933056	*ARMH3*
19	42775001	42825000	0.000933027	*WNK4*
19	42775001	42825000	0.000933027	*EZH1*
19	42775001	42825000	0.000933027	*VPS25*
19	42775001	42825000	0.000933027	*RAMP2*
18	45675001	45725000	0.000933004	*ZNF792*
3	32525001	32575000	0.000932734	*CD53*
7	550001	600000	0.000932663	*CNOT6*
3	66650001	66700000	0.000932316	*DNAJB4*
3	66650001	66700000	0.000932316	*U6*
18	36475001	36525000	0.00093223	*TERF2*
18	36475001	36525000	0.00093223	*COG8*
18	36475001	36525000	0.00093223	*TMED6*
18	36475001	36525000	0.00093223	*NIP7*
19	27325001	27375000	0.000932224	*FXR2*

19	27325001	27375000	0.000932224	*ATP1B2*
19	27325001	27375000	0.000932224	*SHBG*
19	27325001	27375000	0.000932224	*SAT2*
14	30825001	30875000	0.000932176	*MYBL1*
14	30825001	30875000	0.000932176	*VCPIP1*
3	95350001	95400000	0.000932168	*FAF1*
24	43275001	43325000	0.000931722	*CEP192*
24	43275001	43325000	0.000931722	*LDLRAD4*
16	51725001	51775000	0.000931667	*SPEN*
18	44825001	44875000	0.000931573	*UBA2*
18	44825001	44875000	0.000931573	*PDCD2L*
15	29100001	29150000	0.000931568	*KMT2A*
3	30750001	30800000	0.000931191	*ST7L*
3	30750001	30800000	0.000931191	*WNT2B*
23	18500001	18550000	0.000930878	*SUPT3H*
1	65000001	65050000	0.000930615	*GPR156*
4	94175001	94225000	0.00093061	*CEP41*
3	51650001	51700000	0.000930499	*TGFBR3*
10	36050001	36100000	0.000930358	*BAHD1*
10	36050001	36100000	0.000930358	*CHST14*
22	42350001	42400000	0.000930339	*CFAP20DC*
16	39450001	39500000	0.000930228	*DNM3*
1	1925001	1975000	0.000929922	*SON*
1	1925001	1975000	0.000929922	*CRYZL1*
1	1925001	1975000	0.000929922	*DONSON*
13	46675001	46725000	0.000929919	*DIP2C*
16	43425001	43475000	0.000929901	*UBE4B*
16	51150001	51200000	0.000929782	*DVL1*
16	51150001	51200000	0.000929782	*MXRA8*
16	51150001	51200000	0.000929782	*TAS1R3*
16	51150001	51200000	0.000929782	*CPTP*
16	51150001	51200000	0.000929782	*AURKAIP1*
11	30725001	30775000	0.000929563	*PPP1R21*
10	19425001	19475000	0.000929543	*ARIH1*
20	35425001	35475000	0.000929164	*RICTOR*
19	36975001	37025000	0.000929125	*SPOP*
28	25025001	25075000	0.000928489	*CCAR1*
28	25025001	25075000	0.000928489	*TET1*
15	62550001	62600000	0.000928446	*PAX6*
15	62550001	62600000	0.000928446	*ELP4*
22	45500001	45550000	0.000928278	*ERC2*
22	45500001	45550000	0.000928278	*WNT5A*
2	4725001	4775000	0.000927749	*WDR33*
11	94375001	94425000	0.00092753	*STRBP*
11	94375001	94425000	0.00092753	*U6*
7	50100001	50150000	0.000927336	*CTNNA1*
18	14575001	14625000	0.000927203	*FANCA*
18	14575001	14625000	0.000927203	*ZNF276*
18	14575001	14625000	0.000927203	*VPS9D1*
18	14575001	14625000	0.000927203	*SPATA2L*
18	14575001	14625000	0.000927203	*CDK10*
22	10675001	10725000	0.000927078	*TRANK1*

21	26000001	26050000	0.00092705	*ZFAND6*
9	38600001	38650000	0.000927009	*FYN*
21	2050001	2100000	0.000926857	*SNORD116*
21	2050001	2100000	0.000926857	*SNORD116*
21	2050001	2100000	0.000926857	*SNORD116*
21	2050001	2100000	0.000926857	*SNORD116*
21	2050001	2100000	0.000926857	*SNORD116*
21	2050001	2100000	0.000926857	*SNORD116*
21	2050001	2100000	0.000926857	*SNORD116*
18	54150001	54200000	0.000926697	*SAE1*
22	50225001	50275000	0.000926671	*RBM6*
22	50225001	50275000	0.000926671	*RBM5*
22	50225001	50275000	0.000926671	*U6*
14	675001	725000	0.000926461	*MROH1*
14	675001	725000	0.000926461	*HGH1*
14	675001	725000	0.000926461	*WDR97*
14	675001	725000	0.000926461	*TSSK5*
3	108300001	108350000	0.000925871	*GNL2*
3	108300001	108350000	0.000925871	*SNIP1*
3	108300001	108350000	0.000925871	*DNALI1*
3	95425001	95475000	0.000925396	*FAF1*
9	34600001	34650000	0.000925313	*FRK*
2	36900001	36950000	0.000924931	*BAZ2B*
10	86350001	86400000	0.000924744	*TMED10*
10	86350001	86400000	0.000924744	*NEK9*
10	36950001	37000000	0.00092471	*TYRO3*
10	36950001	37000000	0.00092471	*RPAP1*
1	126575001	126625000	0.000924698	*XRN1*
1	126575001	126625000	0.000924698	*ATR*
9	73075001	73125000	0.000924611	*HBS1L*
16	51050001	51100000	0.000924483	*SSU72*
16	51050001	51100000	0.000924483	*ATAD3A*
16	51050001	51100000	0.000924483	*TMEM240*
19	34225001	34275000	0.000923994	*SLC5A10*
19	34225001	34275000	0.000923994	*FAM83G*
19	34225001	34275000	0.000923994	*PRPSAP2*
11	68800001	68850000	0.000923919	*GALNT14*
1	8950001	9000000	0.000923859	*H4C3*
14	39950001	40000000	0.000923853	*ZFHX4*
21	26575001	26625000	0.000923747	*ABHD17C*
13	33500001	33550000	0.000923581	*ARHGAP12*
10	75825001	75875000	0.000923179	*WDR89*
3	94475001	94525000	0.000922751	*NRDC*
17	61925001	61975000	0.000922658	*HECTD4*
10	66500001	66550000	0.000922636	*BMP4*
12	35575001	35625000	0.00092257	*SKA3*
12	35575001	35625000	0.00092257	*SAP18*
12	35575001	35625000	0.00092257	*ZDHHC20*
12	35575001	35625000	0.00092257	*MRPL57*
1	1950001	2000000	0.000922394	*SON*
1	1950001	2000000	0.000922394	*GART*
1	1950001	2000000	0.000922394	*DONSON*

16	12050001	12100000	0.000922392	*CDC73*
16	12050001	12100000	0.000922392	*B3GALT2*
9	59675001	59725000	0.000922378	*MAP3K7*
9	28775001	28825000	0.000921602	*HSF2*
13	57950001	58000000	0.000921594	*VAPB*
13	57950001	58000000	0.000921594	*RAB22A*
9	33875001	33925000	0.000921233	*KPNA5*
9	33875001	33925000	0.000921233	*ZUP1*
25	18600001	18650000	0.000921015	*DCUN1D3*
25	18600001	18650000	0.000921015	*REXO5*
7	43475001	43525000	0.000921009	*ARID3A*
7	43475001	43525000	0.000921009	*GRIN3B*
7	43475001	43525000	0.000921009	*WDR18*
7	43475001	43525000	0.000921009	*TMEM259*
7	20575001	20625000	0.000920708	*CELF5*
7	49525001	49575000	0.000920621	*NME5*
7	49525001	49575000	0.000920621	*WNT8A*
8	75325001	75375000	0.00092029	*UBAP2*
8	75325001	75375000	0.00092029	*UBE2R2*
8	75325001	75375000	0.00092029	*SNORD121A*
19	48725001	48775000	0.00092001	*SMURF2*
1	82825001	82875000	0.000919578	*EIF4G1*
1	82825001	82875000	0.000919578	*CLCN2*
1	82825001	82875000	0.000919578	*FAM131A*
1	82825001	82875000	0.000919578	*POLR2H*
1	82825001	82875000	0.000919578	*SNORD66*
3	63100001	63150000	0.00091918	*ADGRL2*
3	94450001	94500000	0.000918946	*NRDC*
5	30650001	30700000	0.000918514	*TUBA1A*
5	30650001	30700000	0.000918514	*TUBA1B*
17	62250001	62300000	0.0009183	*MAPKAPK5*
19	50900001	50950000	0.000917897	*ASPSCR1*
19	50900001	50950000	0.000917897	*NOTUM*
7	49600001	49650000	0.000917831	*CDC23*
7	49600001	49650000	0.000917831	*BRD8*
7	49600001	49650000	0.000917831	*KIF20A*
29	8475001	8525000	0.000917661	*TMEM135*
5	112250001	112300000	0.000917323	*EP300*
16	51675001	51725000	0.000917068	*SPEN*
10	21375001	21425000	0.00091671	*THTPA*
10	21375001	21425000	0.00091671	*ZFHX2*
10	21375001	21425000	0.00091671	*AP1G2*
10	21375001	21425000	0.00091671	*JPH4*
25	2275001	2325000	0.00091644	*SRRM2*
25	2275001	2325000	0.00091644	*ELOB*
25	2275001	2325000	0.00091644	*FLYWCH1*
25	2275001	2325000	0.00091644	*FLYWCH2*
9	104075001	104125000	0.000915781	*PSMB1*
9	104075001	104125000	0.000915781	*TBP*
9	104075001	104125000	0.000915781	*PDCD2*
1	26150001	26200000	0.00091571	*ROBO1*
13	22775001	22825000	0.000915694	*MLLT10*

19	26950001	27000000	0.000915573	*ELP5*
19	26950001	27000000	0.000915573	*YBX2*
19	26950001	27000000	0.000915573	*SLC2A4*
19	26950001	27000000	0.000915573	*CTDNEP1*
19	26950001	27000000	0.000915573	*PHF23*
19	26950001	27000000	0.000915573	*CLDN7*
19	26950001	27000000	0.000915573	*GABARAP*
9	45250001	45300000	0.000915546	*HACE1*
3	21550001	21600000	0.000915503	*RNF115*
16	42925001	42975000	0.000915433	*CASZ1*
16	42925001	42975000	0.000915433	*PEX14*
11	44675001	44725000	0.000915061	*RANBP2*
11	44675001	44725000	0.000915061	*LIMS1*
19	36650001	36700000	0.000915006	*DLX4*
19	36650001	36700000	0.000915006	*DLX3*
19	42800001	42850000	0.000914981	*WNK4*
19	42800001	42850000	0.000914981	*BECN1*
19	42800001	42850000	0.000914981	*CNTD1*
19	42800001	42850000	0.000914981	*VPS25*
19	42800001	42850000	0.000914981	*RAMP2*
19	42800001	42850000	0.000914981	*COA3*
6	86525001	86575000	0.000914739	*SLC4A4*
6	70850001	70900000	0.000914212	*CLOCK*
6	70850001	70900000	0.000914212	*TMEM165*
2	91625001	91675000	0.00091404	*RAPH1*
2	91625001	91675000	0.00091404	*ABI2*
1	52150001	52200000	0.000913749	*BBX*
2	91150001	91200000	0.000913664	*ICA1L*
10	38000001	38050000	0.000913536	*CDAN1*
10	38000001	38050000	0.000913536	*TTBK2*
1	79875001	79925000	0.000913329	*RTP4*
9	73800001	73850000	0.000913259	*PDE7B*
4	68925001	68975000	0.000913195	*HOXA3*
4	68925001	68975000	0.000913195	*HOXA1*
4	68925001	68975000	0.000913195	*HOXA2*
26	23250001	23300000	0.000913044	*TRIM8*
26	23250001	23300000	0.000913044	*ARL3*
26	23250001	23300000	0.000913044	*SUFU*
7	98100001	98150000	0.000912944	*CHD1*
23	18550001	18600000	0.000912732	*SUPT3H*
23	18550001	18600000	0.000912732	*RUNX2*
3	94575001	94625000	0.000912602	*OSBPL9*
10	51350001	51400000	0.000912418	*SLTM*
14	65300001	65350000	0.000912415	*OSR2*
2	53150001	53200000	0.000912368	*ARHGAP15*
8	59750001	59800000	0.000912256	*RUSC2*
8	59750001	59800000	0.000912256	*TESK1*
8	59750001	59800000	0.000912256	*FAM166B*
8	59750001	59800000	0.000912256	*CD72*
5	36625001	36675000	0.000912104	*TWF1*
29	9575001	9625000	0.000911737	*PICALM*
23	400001	450000	0.000911735	*KHDRBS2*

1	64900001	64950000	0.000911699	*GPR156*
13	38600001	38650000	0.000911425	*DTD1*
16	42900001	42950000	0.000911348	*CASZ1*
3	92250001	92300000	0.000911329	*HSPB11*
3	92250001	92300000	0.000911329	*LRRC42*
7	60375001	60425000	0.000911103	*SH3TC2*
8	75950001	76000000	0.000910916	*CNTFR*
8	75950001	76000000	0.000910916	*DCTN3*
8	75950001	76000000	0.000910916	*ARID3C*
8	75950001	76000000	0.000910916	*RPP25L*
5	81275001	81325000	0.000910868	*CCDC91*
16	76575001	76625000	0.000910842	*DENND1B*
28	17700001	17750000	0.000910555	*CABCOCO1*
1	65175001	65225000	0.000910434	*FSTL1*
10	58925001	58975000	0.000910351	*GLDN*
5	67725001	67775000	0.000910002	*HCFC2*
5	67725001	67775000	0.000910002	*NFYB*
5	75975001	76025000	0.000909776	*USP18*
20	14125001	14175000	0.000909411	*ADAMTS6*
10	58650001	58700000	0.000909265	*SCG3*
7	53125001	53175000	0.000909205	*NDFIP1*
3	52050001	52100000	0.000909016	*HFM1*
7	17025001	17075000	0.000908777	*HNRNPM*
7	17025001	17075000	0.000908777	*MARCHF2*
7	17025001	17075000	0.000908777	*RAB11B*
18	44600001	44650000	0.000908582	*LSM14A*
19	37900001	37950000	0.000908389	*HOXB3*
19	37900001	37950000	0.000908389	*HOXB4*
19	37900001	37950000	0.000908389	*HOXB2*
19	37900001	37950000	0.000908389	*HOXB5*
13	40675001	40725000	0.000908317	*XRN2*
13	40675001	40725000	0.000908317	*NKX2-4*
15	38000001	38050000	0.000908185	*PDE3B*
5	119875001	119925000	0.000908081	*SYCE3*
5	119875001	119925000	0.000908081	*CPT1B*
5	119875001	119925000	0.000908081	*MAPK8IP2*
5	119875001	119925000	0.000908081	*CHKB*
13	22825001	22875000	0.000907975	*MLLT10*
10	59825001	59875000	0.000907848	*USP8*
3	34150001	34200000	0.000907718	*SARS1*
3	34150001	34200000	0.000907718	*CELSR2*
25	26825001	26875000	0.000907455	*PHKG2*
25	26825001	26875000	0.000907455	*TMEM265*
25	26825001	26875000	0.000907455	*U6*
2	20975001	21025000	0.000907374	*LNPK*
7	49575001	49625000	0.000907367	*BRD8*
7	49575001	49625000	0.000907367	*CDC23*
7	49575001	49625000	0.000907367	*KIF20A*
7	49575001	49625000	0.000907367	*NME5*
9	39075001	39125000	0.000907017	*REV3L*
13	57625001	57675000	0.000906908	*NPEPL1*
5	31050001	31100000	0.000906822	*CCNT1*

5	31050001	31100000	0.000906822	*KANSL2*
9	86575001	86625000	0.000906734	*TAB2*
2	115675001	115725000	0.000906645	*AGFG1*
5	56925001	56975000	0.000906414	*TIMELESS*
5	56925001	56975000	0.000906414	*MIP*
5	56925001	56975000	0.000906414	*SPRYD4*
1	133150001	133200000	0.000906346	*PPP2R3A*
10	36225001	36275000	0.000906079	*RAD51*
10	36225001	36275000	0.000906079	*KNL1*
5	70125001	70175000	0.00090603	*RIC8B*
8	25001	75000	0.000905404	*OR2T13*
20	37225001	37275000	0.000905357	*NIPBL*
12	46500001	46550000	0.000905341	*DACH1*
9	29725001	29775000	0.000904976	*GJA1*
16	38225001	38275000	0.000904781	*PRRX1*
22	7800001	7850000	0.000904344	*CLASP2*
3	103875001	103925000	0.000904109	*FOXJ3*
11	73600001	73650000	0.00090403	*ASXL2*
9	41575001	41625000	0.000903623	*FOXO3*
7	43500001	43550000	0.000903353	*GRIN3B*
7	43500001	43550000	0.000903353	*CNN2*
7	43500001	43550000	0.000903353	*WDR18*
7	43500001	43550000	0.000903353	*TMEM259*
7	43500001	43550000	0.000903353	*ABCA7*
22	13775001	13825000	0.000902991	*CTNNB1*
26	32675001	32725000	0.000902857	*GPAM*
6	59125001	59175000	0.000902675	*N4BP2*
4	118175001	118225000	0.000902651	*LMBR1*
9	45100001	45150000	0.000901955	*HACE1*
2	500001	550000	0.000901935	*OCA2*
20	41200001	41250000	0.000901706	*ZFR*
19	23175001	23225000	0.000901688	*SMG6*
8	38375001	38425000	0.000901584	*UHRF2*
15	49100001	49150000	0.000901272	*OR52S22*
15	49100001	49150000	0.000901272	*OR52S23*
15	49100001	49150000	0.000901272	*OR52AB10*
1	106950001	107000000	0.000901233	*KPNA4*
1	106950001	107000000	0.000901233	*SCARNA7*
28	17625001	17675000	0.000901153	*CABCOCO1*
9	40750001	40800000	0.000900977	*CD164*
7	50125001	50175000	0.000900851	*CTNNA1*
18	18725001	18775000	0.000900633	*BRD7*
11	73225001	73275000	0.000900239	*HADHB*
11	73225001	73275000	0.000900239	*HADHA*
3	82075001	82125000	0.000900145	*ITGB3BP*
2	44425001	44475000	0.000900073	*NEB*
5	112300001	112350000	0.000899971	*EP300*
2	350001	400000	0.000899922	*LGSN*
18	21925001	21975000	0.000899839	*RPGRIP1L*
6	58800001	58850000	0.000899669	*UBE2K*
26	22875001	22925000	0.000899338	*GBF1*
26	22875001	22925000	0.000899338	*PITX3*

2	69725001	69775000	0.000899289	*INSIG2*
19	21075001	21125000	0.000899271	*SSH2*
2	119425001	119475000	0.00089913	*PDE6D*
5	23475001	23525000	0.00089901	*CRADD*
1	65125001	65175000	0.000898788	*FSTL1*
2	106625001	106675000	0.000898729	*STK36*
2	106625001	106675000	0.000898729	*TTLL4*
2	106625001	106675000	0.000898729	*RNF25*
7	87750001	87800000	0.000898663	*TMEM161B*
2	79450001	79500000	0.000898128	*GLS*
12	36050001	36100000	0.000898115	*GJB6*
12	36050001	36100000	0.000898115	*GJB2*
18	34175001	34225000	0.00089765	*BEAN1*
3	74875001	74925000	0.000897611	*ANKRD13C*
17	54350001	54400000	0.000897574	*RAD9B*
17	54350001	54400000	0.000897574	*GPN3*
17	54350001	54400000	0.000897574	*FAM216A*
17	54350001	54400000	0.000897574	*VPS29*
10	17575001	17625000	0.00089745	*UACA*
17	13125001	13175000	0.000897424	*OTUD4*
17	13125001	13175000	0.000897424	*ABCE1*
2	91200001	91250000	0.000897027	*WDR12*
2	91200001	91250000	0.000897027	*CARF*
13	60500001	60550000	0.000896816	*CSNK2A1*
9	74150001	74200000	0.000896808	*PDE7B*
22	11575001	11625000	0.000896759	*ACAA1*
22	11575001	11625000	0.000896759	*DLEC1*
22	11575001	11625000	0.000896759	*MYD88*
18	35875001	35925000	0.00089663	*ZFP90*
5	30950001	31000000	0.000896485	*CACNB3*
5	30950001	31000000	0.000896485	*ADCY6*
7	50625001	50675000	0.000896079	*PAIP2*
7	50625001	50675000	0.000896079	*SLC23A1*
7	50625001	50675000	0.000896079	*PROB1*
7	50625001	50675000	0.000896079	*SPATA24*
7	50625001	50675000	0.000896079	*MZB1*
19	12025001	12075000	0.000896056	*BCAS3*
3	103925001	103975000	0.00089599	*FOXJ3*
2	53050001	53100000	0.000895929	*ARHGAP15*
26	21800001	21850000	0.000895636	*SEMA4G*
26	21800001	21850000	0.000895636	*LZTS2*
26	21800001	21850000	0.000895636	*TWNK*
26	21800001	21850000	0.000895636	*SLF2*
26	21800001	21850000	0.000895636	*MRPL43*
6	72200001	72250000	0.000895609	*RESTB*
6	72200001	72250000	0.000895609	*NOA1A*
19	37950001	38000000	0.000895568	*HOXB1*
22	14750001	14800000	0.000895333	*NKTR*
22	14750001	14800000	0.000895333	*ZBTB47*
16	51200001	51250000	0.000895207	*ACAP3*
16	51200001	51250000	0.000895207	*UBE2J2*
16	51200001	51250000	0.000895207	*INTS11*

16	51200001	51250000	0.000895207	*SCNN1D*
16	51200001	51250000	0.000895207	*PUSL1*
16	51200001	51250000	0.000895207	*CPTP*
16	42975001	43025000	0.000894562	*PEX14*
10	102300001	102350000	0.000894267	*TTC7B*
3	32925001	32975000	0.000894119	*CYM*
3	32925001	32975000	0.000894119	*PROK1*
3	49975001	50025000	0.000894108	*FNBP1L*
18	21800001	21850000	0.000893991	*RBL2*
18	21800001	21850000	0.000893991	*AKTIP*
10	19450001	19500000	0.000893986	*ARIH1*
10	19450001	19500000	0.000893986	*BBS4*
18	21550001	21600000	0.000893803	*CHD9*
10	37725001	37775000	0.000893662	*CAPN3*
10	37725001	37775000	0.000893662	*ZNF106*
3	108325001	108375000	0.0008934	*SNIP1*
3	108325001	108375000	0.0008934	*DNALI1*
3	108325001	108375000	0.0008934	*MEAF6*
3	108325001	108375000	0.0008934	*GNL2*
12	35600001	35650000	0.000893373	*SAP18*
10	54775001	54825000	0.00089337	*PYGO1*
10	54775001	54825000	0.00089337	*PRTG*
6	13325001	13375000	0.000893197	*FAM241A*
10	59850001	59900000	0.000893033	*GABPB1*
13	28525001	28575000	0.000892989	*FRMD4A*
2	107075001	107125000	0.000892982	*NHEJ1*
2	107075001	107125000	0.000892982	*SLC23A3*
2	107075001	107125000	0.000892982	*CNPPD1*
16	41250001	41300000	0.000892898	*VPS13D*
4	71975001	72025000	0.000892223	*STK31*
1	79050001	79100000	0.000892034	*LPP*
10	60975001	61025000	0.000891977	*GALK2*
10	59650001	59700000	0.000891752	*TRPM7*
3	94525001	94575000	0.000891633	*OSBPL9*
3	94525001	94575000	0.000891633	*NRDC*
16	38725001	38775000	0.000891626	*FMO2*
8	77100001	77150000	0.000891506	*KIF27*
19	26900001	26950000	0.000891346	*DLG4*
19	26900001	26950000	0.000891346	*DVL2*
19	26900001	26950000	0.000891346	*ACADVL*
19	26900001	26950000	0.000891346	*PHF23*
6	25050001	25100000	0.000891059	*TRMT10A*
6	25050001	25100000	0.000891059	*MTTP*
2	48325001	48375000	0.000890685	*ACVR2A*
10	35725001	35775000	0.000890642	*BMF*
15	30850001	30900000	0.000890144	*POU2F3*
19	37925001	37975000	0.000890039	*HOXB3*
19	37925001	37975000	0.000890039	*HOXB2*
19	37925001	37975000	0.000890039	*HOXB1*
5	112275001	112325000	0.000889777	*EP300*
24	48400001	48450000	0.000889715	*CTIF*
1	78875001	78925000	0.000889692	*LPP*

7	46125001	46175000	0.000889561	*SAR1B*
7	46125001	46175000	0.000889561	*SEC24A*
7	46125001	46175000	0.000889561	*U6*
8	75300001	75350000	0.000889167	*UBE2R2*
8	75300001	75350000	0.000889167	*UBAP2*
8	29825001	29875000	0.000889082	*NFIB*
1	80450001	80500000	0.000889034	*RFC4*
1	80450001	80500000	0.000889034	*EIF4A2*
1	80450001	80500000	0.000889034	*SNORA81*
1	80450001	80500000	0.000889034	*SNORA63*
1	80450001	80500000	0.000889034	*SNORD2*
19	44175001	44225000	0.000889024	*GPATCH8*
19	44175001	44225000	0.000889024	*ITGA2B*
7	50550001	50600000	0.00088851	*MATR3*
7	50550001	50600000	0.00088851	*SNORA74*
7	50550001	50600000	0.00088851	*SNORA74*
7	54000001	54050000	0.000888253	*ARHGAP26*
1	65100001	65150000	0.000887998	*FSTL1*
3	62850001	62900000	0.000887935	*ADGRL2*
22	50900001	50950000	0.000887838	*QRICH1*
22	50900001	50950000	0.000887838	*USP19*
22	50900001	50950000	0.000887838	*QARS1*
3	20325001	20375000	0.000887814	*MRPS21*
3	20325001	20375000	0.000887814	*C3H1orf54*
3	20325001	20375000	0.000887814	*PRPF3*
3	20325001	20375000	0.000887814	*CIART*
3	20325001	20375000	0.000887814	*APH1A*
7	20500001	20550000	0.000887588	*NFIC*
11	32700001	32750000	0.000887445	*NRXN1*
21	32425001	32475000	0.000887203	*PEAK1*
17	61875001	61925000	0.000887173	*HECTD4*
9	45225001	45275000	0.00088711	*HACE1*
10	57750001	57800000	0.000887043	*FAM214A*
25	34950001	35000000	0.000886881	*CUX1*
26	44500001	44550000	0.000886454	*ZRANB1*
26	44500001	44550000	0.000886454	*CTBP2*
3	94600001	94650000	0.000886284	*OSBPL9*
10	42750001	42800000	0.000886282	*NEMF*
10	42750001	42800000	0.000886282	*KLHDC2*
7	38925001	38975000	0.000886107	*DBN1*
7	38925001	38975000	0.000886107	*GRK6*
7	38925001	38975000	0.000886107	*PRR7*
7	54050001	54100000	0.000886074	*ARHGAP26*
19	27350001	27400000	0.000885662	*TP53*
19	27350001	27400000	0.000885662	*WRAP53*
19	27350001	27400000	0.000885662	*ATP1B2*
19	27350001	27400000	0.000885662	*SHBG*
20	14075001	14125000	0.00088523	*ADAMTS6*
11	68200001	68250000	0.000885112	*GMCL1*
11	68200001	68250000	0.000885112	*SNRNP27*
2	107025001	107075000	0.000884922	*NHEJ1*
8	61450001	61500000	0.000884675	*ZCCHC7*

7	41150001	41200000	0.000884591	*OR6AN1*
7	65325001	65375000	0.000884399	*MFAP3*
7	65325001	65375000	0.000884399	*FAM114A2*
7	65325001	65375000	0.000884399	*U6*
10	42775001	42825000	0.000884322	*NEMF*
10	42775001	42825000	0.000884322	*Metazoa_SRP*
13	36125001	36175000	0.000884314	*WAC*
7	42925001	42975000	0.000884251	*OR2AZ3B*
7	42925001	42975000	0.000884251	*OR2AZ1*
25	28400001	28450000	0.000884102	*TYW1*
12	31825001	31875000	0.000884084	*PAN3*
12	31825001	31875000	0.000884084	*FLT1*
9	65900001	65950000	0.000884064	*THEMIS*
3	20725001	20775000	0.000883727	*H4C14*
3	20725001	20775000	0.000883727	*H2AC18*
3	20725001	20775000	0.000883727	*H2AC19*
3	20725001	20775000	0.000883727	*H2AC20*
7	54025001	54075000	0.000883116	*ARHGAP26*
5	30825001	30875000	0.000883075	*ARF3*
5	30825001	30875000	0.000883075	*WNT10B*
5	30825001	30875000	0.000883075	*WNT1*
9	73025001	73075000	0.000883033	*HBS1L*
9	42025001	42075000	0.000883	*OSTM1*
19	27550001	27600000	0.000882941	*CHD3*
19	27550001	27600000	0.000882941	*CYB5D1*
19	27550001	27600000	0.000882941	*RNF227*
18	44625001	44675000	0.000882566	*LSM14A*
18	44625001	44675000	0.000882566	*U1*
18	44625001	44675000	0.000882566	*U6*
4	94350001	94400000	0.000882341	*COPG2*
16	42450001	42500000	0.000882273	*MTOR*
16	42450001	42500000	0.000882273	*ANGPTL7*
17	54275001	54325000	0.000881787	*ANAPC7*
3	34300001	34350000	0.000881615	*WDR47*
3	34300001	34350000	0.000881615	*TAF13*
20	23725001	23775000	0.000881423	*MTREX*
20	23725001	23775000	0.000881423	*PLPP1*
24	43150001	43200000	0.000881405	*PTPN2*
24	43150001	43200000	0.000881405	*SEH1L*
13	29475001	29525000	0.000881363	*HSPA14*
13	29475001	29525000	0.000881363	*CDNF*
13	29475001	29525000	0.000881363	*SNORD22*
3	22475001	22525000	0.000881239	*FMO5*
9	63825001	63875000	0.000881026	*SYNCRIP*
9	63825001	63875000	0.000881026	*SNX14*
21	22700001	22750000	0.000880901	*PDE8A*
10	42700001	42750000	0.00088015	*KLHDC1*
10	42700001	42750000	0.00088015	*POLE2*
10	42700001	42750000	0.00088015	*KLHDC2*
12	35900001	35950000	0.000880076	*CRYL1*
12	35900001	35950000	0.000880076	*IFT88*
12	76700001	76750000	0.000879817	*ZIC5*

12	76700001	76750000	0.000879817	*ZIC2*
12	35525001	35575000	0.00087971	*ZDHHC20*
17	58600001	58650000	0.000879648	*MED13L*
13	23475001	23525000	0.00087959	*SPAG6*
15	37775001	37825000	0.000879499	*CALCB*
10	42725001	42775000	0.000879133	*NEMF*
10	42725001	42775000	0.000879133	*KLHDC1*
10	42725001	42775000	0.000879133	*KLHDC2*
5	56725001	56775000	0.0008787	*NACA*
5	56725001	56775000	0.0008787	*PTGES3*
5	56725001	56775000	0.0008787	*PRIM1*
6	72125001	72175000	0.000878666	*RESTB*
13	12175001	12225000	0.000878587	*NUDT5*
13	12175001	12225000	0.000878587	*CDC123*
13	12175001	12225000	0.000878587	*SEC61A2*
3	112500001	112550000	0.000878082	*GIGYF2*
3	112500001	112550000	0.000878082	*KCNJ13*
25	18525001	18575000	0.000877868	*ERI2*
25	18525001	18575000	0.000877868	*REXO5*
25	18525001	18575000	0.000877868	*ACSM3*
11	12975001	13025000	0.000877618	*DYSF*
13	32700001	32750000	0.000877601	*CACNB2*
6	67925001	67975000	0.000877534	*SPATA18*
2	20800001	20850000	0.000877478	*EVX2*
2	20800001	20850000	0.000877478	*HOXD11*
2	20800001	20850000	0.000877478	*HOXD13*
2	20800001	20850000	0.000877478	*HOXD12*
12	54575001	54625000	0.000877295	*NDFIP2*
18	35850001	35900000	0.000877154	*ZFP90*
13	32175001	32225000	0.000877066	*TMEM236*
7	43600001	43650000	0.000877056	*SBNO2*
12	36650001	36700000	0.000876888	*ATP12A*
28	17650001	17700000	0.000876628	*CABCOCO1*
1	81475001	81525000	0.000876536	*IGF2BP2*
3	20300001	20350000	0.000876373	*PRPF3*
3	20300001	20350000	0.000876373	*MRPS21*
3	20300001	20350000	0.000876373	*RPRD2*
11	30675001	30725000	0.000876224	*FOXN2*
28	14000001	14050000	0.000876004	*BICC1*
28	13775001	13825000	0.000875958	*HNRNPF*
28	13775001	13825000	0.000875958	*FXYD4*
4	43300001	43350000	0.000875734	*PHTF2*
21	41200001	41250000	0.000875484	*G2E3*
13	60550001	60600000	0.000875339	*TBC1D20*
13	60550001	60600000	0.000875339	*CSNK2A1*
9	66825001	66875000	0.000875236	*U6*
15	82500001	82550000	0.000875101	*OR5AN2*
15	82500001	82550000	0.000875101	*OR5AN1K*
4	112325001	112375000	0.000875094	*ZNF212*
1	111350001	111400000	0.000874905	*KCNAB1*
21	2300001	2350000	0.000874891	*UBE3A*
13	39200001	39250000	0.000874664	*SLC24A3*

12	35650001	35700000	0.000874347	*LATS2*
2	71850001	71900000	0.000874309	*EPB41L5*
2	71850001	71900000	0.000874309	*U4*
27	15625001	15675000	0.000874162	*UFSP2*
27	15625001	15675000	0.000874162	*LRP2BP*
27	15625001	15675000	0.000874162	*ANKRD37*
18	49050001	49100000	0.000873442	*SAMD4B*
18	49050001	49100000	0.000873442	*MED29*
18	49050001	49100000	0.000873442	*PAF1*
9	44975001	45025000	0.000873437	*LIN28B*
29	9550001	9600000	0.000873386	*PICALM*
10	36525001	36575000	0.000873309	*INO80*
9	60450001	60500000	0.000872969	*MDN1*
2	20750001	20800000	0.000872465	*HOXD3*
2	20750001	20800000	0.000872465	*HOXD10*
2	20750001	20800000	0.000872465	*HOXD4*
2	20750001	20800000	0.000872465	*HOXD9*
2	20750001	20800000	0.000872465	*HOXD8*
22	6075001	6125000	0.000872293	*STT3B*
18	36975001	37025000	0.000872286	*PSMD7*
5	26500001	26550000	0.000872104	*MAP3K12*
5	26500001	26550000	0.000872104	*PCBP2*
5	26500001	26550000	0.000872104	*ATF7*
5	26500001	26550000	0.000872104	*TARBP2*
5	26500001	26550000	0.000872104	*NPFF*
2	90750001	90800000	0.000871655	*SUMO1*
2	90750001	90800000	0.000871655	*NOP58*
2	90750001	90800000	0.000871655	*SNORD70*
2	90750001	90800000	0.000871655	*SNORD70B*
18	53150001	53200000	0.000871255	*OPA3*
18	53150001	53200000	0.000871255	*GPR4*
1	81400001	81450000	0.00087113	*IGF2BP2*
1	79000001	79050000	0.000871104	*LPP*
7	7850001	7900000	0.000871077	*BRD4*
7	7850001	7900000	0.000871077	*EPHX3*
18	53075001	53125000	0.000871055	*VASP*
18	53075001	53125000	0.000871055	*RTN2*
18	53075001	53125000	0.000871055	*FOSB*
18	53075001	53125000	0.000871055	*PPM1N*
3	94500001	94550000	0.000869687	*NRDC*
3	94500001	94550000	0.000869687	*OSBPL9*
3	8675001	8725000	0.00086917	*CD244*
13	57500001	57550000	0.000869148	*GNAS*
5	76375001	76425000	0.000869073	*SYT10*
11	29550001	29600000	0.000868965	*STPG4*
11	29550001	29600000	0.000868965	*CALM2*
15	53475001	53525000	0.000868756	*C2CD3*
17	57025001	57075000	0.00086868	*TAOK3*
28	13975001	14025000	0.000868437	*BICC1*
21	32550001	32600000	0.000868399	*HMG20A*
21	32550001	32600000	0.000868399	*PEAK1*
10	58425001	58475000	0.000868255	*TMOD3*

10	58425001	58475000	0.000868255	*LEO1*
18	21900001	21950000	0.000868028	*RPGRIP1L*
3	95475001	95525000	0.000867832	*FAF1*
2	22775001	22825000	0.000867699	*SP3*
10	86150001	86200000	0.000867698	*RPS6KL1*
10	86150001	86200000	0.000867698	*DLST*
16	69525001	69575000	0.000867406	*PROX1*
5	112875001	112925000	0.000867299	*SREBF2*
5	112875001	112925000	0.000867299	*CENPM*
5	112875001	112925000	0.000867299	*SHISA8*
5	112875001	112925000	0.000867299	*TNFRSF13C*
19	47000001	47050000	0.000867295	*TLK2*
19	47000001	47050000	0.000867295	*MRC2*
1	116250001	116300000	0.000865892	*Y_RNA*
26	22500001	22550000	0.000865852	*OGA*
26	22500001	22550000	0.000865852	*FGF8*
26	22500001	22550000	0.000865852	*NPM3*
4	43550001	43600000	0.000865627	*PTPN12*
17	53750001	53800000	0.000865602	*RNF34*
17	53750001	53800000	0.000865602	*KDM2B*
7	47550001	47600000	0.00086536	*SMAD5*
5	43125001	43175000	0.000864512	*CNOT2*
3	21575001	21625000	0.000864004	*RNF115*
9	45000001	45050000	0.000863644	*LIN28B*
15	62525001	62575000	0.000863499	*ELP4*
15	62525001	62575000	0.000863499	*PAX6*
22	49325001	49375000	0.000863478	*DCAF1*
22	49325001	49375000	0.000863478	*RBM15B*
3	119575001	119625000	0.000863389	*AKAP17A*
3	119575001	119625000	0.000863389	*OR9S36*
6	67725001	67775000	0.000863111	*DCUN1D4*
3	83950001	84000000	0.000863093	*TM2D1*
20	14600001	14650000	0.000862862	*CWC27*
23	8400001	8450000	0.000862718	*NUDT3*
5	7550001	7600000	0.000862652	*NAV3*
10	42800001	42850000	0.000862185	*NEMF*
10	42800001	42850000	0.000862185	*Metazoa_SRP*
10	42800001	42850000	0.000862185	*Metazoa_SRP*
8	77000001	77050000	0.000861847	*GKAP1*
24	34575001	34625000	0.000861805	*ABHD3*
24	34575001	34625000	0.000861805	*SNRPD1*
18	36875001	36925000	0.000861314	*WWP2*
7	18625001	18675000	0.000861104	*SAFB*
19	40350001	40400000	0.000860905	*THRA*
19	40350001	40400000	0.000860905	*MED24*
19	40350001	40400000	0.000860905	*NR1D1*
8	275001	325000	0.000860897	*MFSD14B*
10	37800001	37850000	0.000860695	*SNAP23*
10	37800001	37850000	0.000860695	*ZNF106*
19	21475001	21525000	0.000860619	*CPD*
5	60825001	60875000	0.000860616	*CFAP54*
2	107175001	107225000	0.000860538	*GLB1L*

2	107175001	107225000	0.000860538	*TUBA4A*
2	107175001	107225000	0.000860538	*ANKZF1*
2	107175001	107225000	0.000860538	*TUBA1D*
2	107175001	107225000	0.000860538	*ATG9A*
2	107175001	107225000	0.000860538	*STK16*
22	51125001	51175000	0.000860261	*PRKAR2A*
11	44650001	44700000	0.00086026	*RANBP2*
11	73925001	73975000	0.000860246	*DTNB*
11	73925001	73975000	0.000860246	*DNMT3A*
11	24425001	24475000	0.0008599	*PKDCC*
2	43475001	43525000	0.00085952	*PRPF40A*
19	30375001	30425000	0.000859334	*DNAH9*
7	47525001	47575000	0.000859308	*SMAD5*
5	31025001	31075000	0.000858887	*CCNT1*
5	31025001	31075000	0.000858887	*TEX49*
7	19600001	19650000	0.000858853	*HDGFL2*
7	19600001	19650000	0.000858853	*PLIN4*
7	19600001	19650000	0.000858853	*PLIN5*
5	56275001	56325000	0.000858703	*LRP1*
5	56275001	56325000	0.000858703	*SNORA62*
7	45750001	45800000	0.000858676	*TCF7*
15	55850001	55900000	0.000858673	*EMSY*
2	53125001	53175000	0.000858408	*ARHGAP15*
11	24950001	25000000	0.000858105	*MTA3*
11	24950001	25000000	0.000858105	*U6*
3	51950001	52000000	0.00085809	*HFM1*
7	86950001	87000000	0.000857172	*RASA1*
2	7925001	7975000	0.000857155	*GULP1*
2	7925001	7975000	0.000857155	*U1*
19	11325001	11375000	0.000857116	*BRIP1*
8	23750001	23800000	0.000856989	*MLLT3*
9	98900001	98950000	0.000856898	*QKI*
18	35225001	35275000	0.000856835	*GFOD2*
18	35225001	35275000	0.000856835	*RANBP10*
3	95375001	95425000	0.000856527	*FAF1*
6	70900001	70950000	0.000856516	*CLOCK*
13	32050001	32100000	0.000856285	*HACD1*
13	32050001	32100000	0.000856285	*STAM*
18	34450001	34500000	0.000856128	*DYNC1LI2*
18	34450001	34500000	0.000856128	*TERB1*
9	25225001	25275000	0.000855945	*TRMT11*
1	64700001	64750000	0.000855923	*GSK3B*
26	22775001	22825000	0.000855663	*PPRC1*
26	22775001	22825000	0.000855663	*LDB1*
26	22775001	22825000	0.000855663	*NOLC1*
26	22775001	22825000	0.000855663	*SNORD22*
8	22550001	22600000	0.000855554	*IFNT3*
17	29850001	29900000	0.000855443	*HSPA4L*
17	29850001	29900000	0.000855443	*SLC25A31*
19	27000001	27050000	0.000855367	*NEURL4*
19	27000001	27050000	0.000855367	*KCTD11*
19	27000001	27050000	0.000855367	*EIF5A*

19	27000001	27050000	0.000855367	*GPS2*
10	36975001	37025000	0.00085528	*TYRO3*
10	36975001	37025000	0.00085528	*U6*
7	49300001	49350000	0.000855222	*MYOT*
3	58750001	58800000	0.000854518	*DDAH1*
7	50075001	50125000	0.000854465	*CTNNA1*
13	29500001	29550000	0.00085418	*SUV39H2*
13	29500001	29550000	0.00085418	*HSPA14*
13	29500001	29550000	0.00085418	*SNORD22*
23	31275001	31325000	0.000853875	*PRSS16*
1	115925001	115975000	0.000853851	*SUCNR1*
9	41775001	41825000	0.000853597	*AFG1L*
1	126600001	126650000	0.000853386	*XRN1*
18	24425001	24475000	0.000853375	*GNAO1*
10	57700001	57750000	0.000853195	*FAM214A*
5	57300001	57350000	0.000853085	*RAB5B*
5	57300001	57350000	0.000853085	*PMEL*
5	57300001	57350000	0.000853085	*CDK2*
5	57300001	57350000	0.000853085	*SUOX*
5	57300001	57350000	0.000853085	*IKZF4*
21	10525001	10575000	0.000853005	*NR2F2*
3	34175001	34225000	0.00085297	*SARS1*
3	34175001	34225000	0.00085297	*ELAPOR1*
11	30125001	30175000	0.000852901	*FBXO11*
11	30125001	30175000	0.000852901	*MSH6*
7	43450001	43500000	0.000852875	*ARID3A*
7	43450001	43500000	0.000852875	*KISS1R*
6	70875001	70925000	0.000852564	*CLOCK*
9	98850001	98900000	0.000851632	*QKI*
16	51650001	51700000	0.000851631	*SPEN*
1	148350001	148400000	0.000851555	*CBR1*
10	86075001	86125000	0.000851418	*YLPM1*
10	86075001	86125000	0.000851418	*PROX2*
5	120000001	120050000	0.000851413	*RABL2B*
5	120000001	120050000	0.000851413	*ACR*
5	120000001	120050000	0.000851413	*SHANK3*
1	2825001	2875000	0.000851377	*SYNJ1*
1	81500001	81550000	0.000850974	*IGF2BP2*
16	21775001	21825000	0.000850918	*TGFB2*
16	21775001	21825000	0.000850918	*RRP15*
9	44950001	45000000	0.000850873	*LIN28B*
10	60925001	60975000	0.000850817	*GALK2*
17	58725001	58775000	0.00085079	*MED13L*
22	16950001	17000000	0.000850464	*BRPF1*
22	16950001	17000000	0.000850464	*CPNE9*
22	16950001	17000000	0.000850464	*CAMK1*
22	16950001	17000000	0.000850464	*OGG1*
2	36500001	36550000	0.000850331	*LY75*
7	51500001	51550000	0.000850278	*HBEGF*
7	51500001	51550000	0.000850278	*PFDN1*
19	27750001	27800000	0.000850105	*ALOXE3*
19	27750001	27800000	0.000850105	*PER1*

19	27750001	27800000	0.000850105	*VAMP2*
19	27750001	27800000	0.000850105	*HES7*
19	27750001	27800000	0.000850105	*U6*
3	50025001	50075000	0.000849921	*FNBP1L*
9	15325001	15375000	0.000849847	*SENP6*
4	94150001	94200000	0.000849721	*CEP41*
19	43425001	43475000	0.000849687	*DHX8*
19	43425001	43475000	0.000849687	*ETV4*
22	49275001	49325000	0.000849548	*DCAF1*
22	49275001	49325000	0.000849548	*RAD54L2*
22	6050001	6100000	0.000849497	*STT3B*
10	21575001	21625000	0.000849338	*PABPN1*
10	21575001	21625000	0.000849338	*BCL2L2*
10	21575001	21625000	0.000849338	*HOMEZ*
10	21575001	21625000	0.000849338	*SLC22A17*
10	21575001	21625000	0.000849338	*PPP1R3E*
16	60425001	60475000	0.000849333	*TOR3A*
16	60425001	60475000	0.000849333	*ABL2*
7	98075001	98125000	0.000849222	*CHD1*
13	23425001	23475000	0.000849171	*SPAG6*
13	23425001	23475000	0.000849171	*BMI1*
13	23425001	23475000	0.000849171	*COMMD3*
26	37800001	37850000	0.000848472	*EMX2*
22	10900001	10950000	0.000848464	*ITGA9*
2	91500001	91550000	0.000848399	*CYP20A1*
2	91500001	91550000	0.000848399	*ABI2*
5	30975001	31025000	0.000848362	*ADCY6*
5	30975001	31025000	0.000848362	*TEX49*
19	38150001	38200000	0.000848224	*SKAP1*
29	40200001	40250000	0.000848076	*MYRF*
29	40200001	40250000	0.000848076	*FADS1*
29	40200001	40250000	0.000848076	*FEN1*
29	40200001	40250000	0.000848076	*TMEM258*
10	75850001	75900000	0.000847993	*WDR89*
1	126225001	126275000	0.000847936	*TRPC1*
1	126225001	126275000	0.000847936	*PCOLCE2*
22	13750001	13800000	0.000847893	*CTNNB1*
15	53575001	53625000	0.000847718	*PPME1*
15	53575001	53625000	0.000847718	*C2CD3*
2	34975001	35025000	0.000847705	*PSMD14*
7	60400001	60450000	0.00084749	*SH3TC2*
7	50050001	50100000	0.000847362	*CTNNA1*
17	52250001	52300000	0.00084671	*SBNO1*
3	9350001	9400000	0.000846607	*COPA*
3	9350001	9400000	0.000846607	*NCSTN*
3	9350001	9400000	0.000846607	*PEX19*
16	38950001	39000000	0.000846478	*PRRC2C*
3	3100001	3150000	0.000846373	*TMCO1*
16	42950001	43000000	0.000845936	*PEX14*
16	42950001	43000000	0.000845936	*CASZ1*
20	62850001	62900000	0.000845814	*MARCHF6*
8	75275001	75325000	0.000845547	*UBE2R2*

29	43750001	43800000	0.000845472	*MAP3K11*
29	43750001	43800000	0.000845472	*EHBP1L1*
29	43750001	43800000	0.000845472	*PCNX3*
29	43750001	43800000	0.000845472	*KCNK7*
29	43750001	43800000	0.000845472	*FAM89B*
29	43750001	43800000	0.000845472	*ZNRD2*
1	82850001	82900000	0.000845301	*EIF4G1*
1	82850001	82900000	0.000845301	*PSMD2*
1	82850001	82900000	0.000845301	*ECE2*
1	82850001	82900000	0.000845301	*FAM131A*
1	82850001	82900000	0.000845301	*SNORD66*
1	81550001	81600000	0.00084527	*IGF2BP2*
1	81550001	81600000	0.00084527	*SENP2*
1	132200001	132250000	0.000844857	*NCK1*
1	132200001	132250000	0.000844857	*IL20RB*
26	21725001	21775000	0.000844848	*SLF2*
2	36750001	36800000	0.000844659	*BAZ2B*
26	23150001	23200000	0.000844645	*SUFU*
18	21950001	22000000	0.000844444	*RPGRIP1L*
16	60525001	60575000	0.000844405	*ABL2*
26	15900001	15950000	0.000844307	*TBC1D12*
7	43850001	43900000	0.000843771	*APC2*
7	43850001	43900000	0.000843771	*PCSK4*
7	43850001	43900000	0.000843771	*C7H19orf25*
7	43850001	43900000	0.000843771	*REEP6*
10	58625001	58675000	0.000843682	*SCG3*
10	58625001	58675000	0.000843682	*LYSMD2*
7	5825001	5875000	0.000843366	*NR2F6*
7	5825001	5875000	0.000843366	*USHBP1*
7	5825001	5875000	0.000843366	*ANKLE1*
7	5825001	5875000	0.000843366	*BABAM1*
7	5825001	5875000	0.000843366	*ABHD8*
7	5825001	5875000	0.000843366	*OCEL1*
20	41250001	41300000	0.00084331	*ZFR*
7	51525001	51575000	0.000843259	*SLC4A9*
7	51525001	51575000	0.000843259	*HBEGF*
13	46375001	46425000	0.000843158	*LARP4B*
13	46375001	46425000	0.000843158	*U6*
2	128125001	128175000	0.000843135	*CLIC4*
10	25650001	25700000	0.000843118	*METTL3*
10	25650001	25700000	0.000843118	*TOX4*
10	25650001	25700000	0.000843118	*RAB2B*
10	25650001	25700000	0.000843118	*SALL2*
12	35625001	35675000	0.000842063	*LATS2*
3	105100001	105150000	0.00084206	*SCMH1*
11	38000001	38050000	0.000841802	*CCDC88A*
11	38000001	38050000	0.000841802	*PRORSD1*
9	73050001	73100000	0.000841719	*HBS1L*
10	42650001	42700000	0.00084164	*POLE2*
10	42650001	42700000	0.00084164	*DNAAF2*
10	42650001	42700000	0.00084164	*LRR1*
10	42650001	42700000	0.00084164	*MGAT2*

10	42650001	42700000	0.00084164	*RPL36AL*
21	22450001	22500000	0.000841389	*ALPK3*
21	22450001	22500000	0.000841389	*ZNF592*
13	22700001	22750000	0.000840955	*SKIDA1*
13	22700001	22750000	0.000840955	*MLLT10*
10	42675001	42725000	0.000840384	*POLE2*
10	42675001	42725000	0.000840384	*KLHDC1*
5	70275001	70325000	0.000840154	*CRY1*
10	33925001	33975000	0.000839331	*SPRED1*
11	27150001	27200000	0.000838972	*CAMKMT*
5	51150001	51200000	0.000838727	*USP15*
5	119975001	120025000	0.000838498	*SHANK3*
5	119975001	120025000	0.000838498	*ACR*
6	67900001	67950000	0.000838185	*SPATA18*
6	67900001	67950000	0.000838185	*SGCB*
8	59525001	59575000	0.000838061	*UNC13B*
8	94475001	94525000	0.000837984	*OR13C1*
17	54225001	54275000	0.000837874	*ATP2A2*
8	38425001	38475000	0.000837723	*TPD52L3*
8	38425001	38475000	0.000837723	*IL33*
22	49300001	49350000	0.000837486	*DCAF1*
3	87325001	87375000	0.000837024	*MYSM1*
13	33150001	33200000	0.000836788	*EPC1*
7	49975001	50025000	0.000835968	*CTNNA1*
4	46425001	46475000	0.000835711	*SRPK2*
8	38400001	38450000	0.000834689	*TPD52L3*
10	54250001	54300000	0.00083457	*RFX7*
6	67750001	67800000	0.000834492	*DCUN1D4*
28	17675001	17725000	0.000834378	*CABCOCO1*
14	37575001	37625000	0.000834263	*JPH1*
25	9150001	9200000	0.000834246	*ATF7IP2*
25	9150001	9200000	0.000834246	*U6*
16	39375001	39425000	0.000834157	*DNM3*
14	23350001	23400000	0.000833872	*PLAG1*
14	23350001	23400000	0.000833872	*CHCHD7*
4	80950001	81000000	0.000833682	*CDK13*
10	57725001	57775000	0.000833658	*FAM214A*
10	71575001	71625000	0.000833449	*DAAM1*
9	45050001	45100000	0.000833382	*LIN28B*
3	95400001	95450000	0.000832848	*FAF1*
24	43000001	43050000	0.000832525	*SPIRE1*
1	64575001	64625000	0.000832357	*NR1I2*
9	45150001	45200000	0.000831231	*HACE1*
7	50825001	50875000	0.000830788	*UBE2D2*
22	50875001	50925000	0.000830514	*LAMB2*
22	50875001	50925000	0.000830514	*USP19*
22	50875001	50925000	0.000830514	*QARS1*
22	50875001	50925000	0.000830514	*QRICH1*
16	42425001	42475000	0.000830471	*MTOR*
16	42425001	42475000	0.000830471	*ANGPTL7*
25	38875001	38925000	0.000829742	*TNRC18*
1	133125001	133175000	0.000829348	*PPP2R3A*

16	12075001	12125000	0.000828887	*CDC73*
10	82275001	82325000	0.00082783	*TTC9*
5	119775001	119825000	0.000827642	*NCAPH2*
5	119775001	119825000	0.000827642	*SBF1*
5	119775001	119825000	0.000827642	*LMF2*
5	119775001	119825000	0.000827642	*ADM2*
5	119775001	119825000	0.000827642	*MIOX*
5	119775001	119825000	0.000827642	*SCO2*
5	30725001	30775000	0.000827245	*KMT2D*
5	30725001	30775000	0.000827245	*DHH*
5	30725001	30775000	0.000827245	*RHEBL1*
5	30725001	30775000	0.000827245	*LMBR1L*
18	35200001	35250000	0.000827177	*GFOD2*
18	35200001	35250000	0.000827177	*CARMIL2*
18	35200001	35250000	0.000827177	*ACD*
18	35200001	35250000	0.000827177	*ENKD1*
18	35200001	35250000	0.000827177	*PARD6A*
18	35200001	35250000	0.000827177	*C18H16orf86*
3	95300001	95350000	0.00082717	*CDKN2C*
18	22000001	22050000	0.000827143	*RPGRIP1L*
18	22000001	22050000	0.000827143	*FTO*
18	14700001	14750000	0.000827068	*DEF8*
18	14700001	14750000	0.000827068	*TUBB3*
18	14700001	14750000	0.000827068	*TCF25*
18	14700001	14750000	0.000827068	*MC1R*
24	47500001	47550000	0.000826895	*SMAD2*
18	45650001	45700000	0.000826693	*ZNF792*
7	19925001	19975000	0.000826648	*ZBTB7A*
7	19925001	19975000	0.000826648	*MAP2K2*
5	69800001	69850000	0.00082658	*POLR3B*
26	18225001	18275000	0.000826518	*LCOR*
26	18225001	18275000	0.000826518	*SLIT1*
3	59875001	59925000	0.000826484	*PRKACB*
5	31000001	31050000	0.000826353	*TEX49*
5	31000001	31050000	0.000826353	*ADCY6*
3	95450001	95500000	0.000826241	*FAF1*
19	40375001	40425000	0.000825708	*THRA*
19	40375001	40425000	0.000825708	*NR1D1*
19	40375001	40425000	0.000825708	*MSL1*
17	54400001	54450000	0.000825056	*PPTC7*
17	54400001	54450000	0.000825056	*RAD9B*
13	60525001	60575000	0.000825015	*CSNK2A1*
7	49850001	49900000	0.000824994	*ETF1*
7	49850001	49900000	0.000824994	*HSPA9*
7	49850001	49900000	0.000824994	*SNORD63*
7	49850001	49900000	0.000824994	*SNORD63*
8	75350001	75400000	0.000824815	*UBAP2*
8	75350001	75400000	0.000824815	*SNORD121A*
13	47325001	47375000	0.000824681	*SLC23A2*
9	26375001	26425000	0.000824412	*NKAIN2*
21	10550001	10600000	0.000824242	*NR2F2*
5	70025001	70075000	0.000824148	*RFX4*

5	70025001	70075000	0.000824148	*RIC8B*
5	55925001	55975000	0.00082412	*MARS1*
5	55925001	55975000	0.00082412	*DCTN2*
5	55925001	55975000	0.00082412	*KIF5A*
5	55925001	55975000	0.00082412	*MBD6*
5	55925001	55975000	0.00082412	*DDIT3*
8	39050001	39100000	0.000823948	*RIC1*
8	39050001	39100000	0.000823948	*PDCD1LG2*
2	91050001	91100000	0.000823885	*FAM117B*
1	82375001	82425000	0.00082381	*VPS8*
8	59225001	59275000	0.000823631	*PHF24*
8	59225001	59275000	0.000823631	*U6*
8	59250001	59300000	0.000823097	*PHF24*
8	59250001	59300000	0.000823097	*DNAJB5*
8	59250001	59300000	0.000823097	*U6*
3	21700001	21750000	0.000822949	*GPR89A*
24	49000001	49050000	0.000822673	*DYM*
10	65025001	65075000	0.000822673	*SPATA5L1*
10	61100001	61150000	0.000822268	*COPS2*
8	75375001	75425000	0.00082224	*UBAP2*
7	93025001	93075000	0.000822236	*NR2F1*
12	26150001	26200000	0.000822233	*NBEA*
16	51175001	51225000	0.000821454	*DVL1*
16	51175001	51225000	0.000821454	*INTS11*
16	51175001	51225000	0.000821454	*ACAP3*
16	51175001	51225000	0.000821454	*CPTP*
16	51175001	51225000	0.000821454	*TAS1R3*
16	51175001	51225000	0.000821454	*PUSL1*
16	51175001	51225000	0.000821454	*MXRA8*
19	26925001	26975000	0.000821334	*DVL2*
19	26925001	26975000	0.000821334	*ELP5*
19	26925001	26975000	0.000821334	*DLG4*
19	26925001	26975000	0.000821334	*CTDNEP1*
19	26925001	26975000	0.000821334	*ACADVL*
19	26925001	26975000	0.000821334	*PHF23*
19	26925001	26975000	0.000821334	*CLDN7*
19	26925001	26975000	0.000821334	*GABARAP*
3	9075001	9125000	0.000821234	*CD84*
9	66850001	66900000	0.000821199	*U6*
6	13050001	13100000	0.000821024	*NEUROG2*
6	13050001	13100000	0.000821024	*ALPK1*
3	30725001	30775000	0.000820914	*ST7L*
3	66675001	66725000	0.000820114	*DNAJB4*
3	66675001	66725000	0.000820114	*FUBP1*
3	66675001	66725000	0.000820114	*U6*
5	76400001	76450000	0.000820098	*SYT10*
15	49925001	49975000	0.000820009	*OR51L1B*
15	49925001	49975000	0.000820009	*OR51P1B*
6	17075001	17125000	0.000819524	*LEF1*
15	17650001	17700000	0.000819456	*CUL5*
11	95525001	95575000	0.000819303	*NR6A1*
11	95525001	95575000	0.000819303	*NR5A1*

16	41275001	41325000	0.000819135	*VPS13D*
7	575001	625000	0.000819122	*CNOT6*
5	119950001	120000000	0.000819065	*SHANK3*
11	95600001	95650000	0.000818923	*NR6A1*
10	54475001	54525000	0.00081879	*NEDD4*
3	94425001	94475000	0.000818677	*NRDC*
5	119925001	119975000	0.000818573	*SHANK3*
5	119925001	119975000	0.000818573	*ARSA*
5	119925001	119975000	0.000818573	*MAPK8IP2*
15	17675001	17725000	0.00081844	*CUL5*
3	83925001	83975000	0.000818071	*TM2D1*
3	83925001	83975000	0.000818071	*PATJ*
26	22750001	22800000	0.000818029	*LDB1*
26	22750001	22800000	0.000818029	*PPRC1*
26	22750001	22800000	0.000818029	*HPS6*
4	44925001	44975000	0.000817868	*RELN*
7	5800001	5850000	0.000817669	*DDA1*
7	5800001	5850000	0.000817669	*ABHD8*
7	5800001	5850000	0.000817669	*ANKLE1*
7	5800001	5850000	0.000817669	*BABAM1*
7	5800001	5850000	0.000817669	*ANO8*
7	5800001	5850000	0.000817669	*USHBP1*
7	5800001	5850000	0.000817669	*MRPL34*
12	35675001	35725000	0.000817602	*XPO4*
12	35675001	35725000	0.000817602	*LATS2*
10	82375001	82425000	0.000817215	*MAP3K9*
1	64550001	64600000	0.000816953	*CFAP91*
11	61500001	61550000	0.000816902	*EHBP1*
11	61500001	61550000	0.000816902	*OTX1*
2	43500001	43550000	0.000816578	*PRPF40A*
2	43500001	43550000	0.000816578	*FMNL2*
9	34350001	34400000	0.000816226	*DSE*
17	54200001	54250000	0.000816043	*ATP2A2*
17	54200001	54250000	0.000816043	*IFT81*
13	32125001	32175000	0.000815694	*STAM*
13	32125001	32175000	0.000815694	*SNORD62*
13	49150001	49200000	0.000815225	*BMP2*
24	48750001	48800000	0.000815211	*DYM*
16	39425001	39475000	0.000814818	*DNM3*
9	94425001	94475000	0.000814681	*SNX9*
7	49825001	49875000	0.000814539	*ETF1*
7	49825001	49875000	0.000814539	*EGR1*
9	45025001	45075000	0.000814258	*LIN28B*
11	30700001	30750000	0.00081402	*FOXN2*
12	36400001	36450000	0.000813666	*PARP4*
5	30800001	30850000	0.000813449	*PRKAG1*
5	30800001	30850000	0.000813449	*WNT10B*
5	30800001	30850000	0.000813449	*DDN*
5	30800001	30850000	0.000813449	*WNT1*
11	74775001	74825000	0.000813449	*U3*
10	86200001	86250000	0.00081291	*PGF*
24	43550001	43600000	0.000812908	*MC2R*

1	106900001	106950000	0.000812797	*KPNA4*
1	86275001	86325000	0.00081273	*CCDC39*
17	67725001	67775000	0.000812262	*TTC28*
9	62625001	62675000	0.000812137	*ZNF292*
17	67750001	67800000	0.000812054	*TTC28*
7	45775001	45825000	0.000811958	*TCF7*
7	45775001	45825000	0.000811958	*SKP1*
20	26225001	26275000	0.000811755	*ITGA1*
20	26225001	26275000	0.000811755	*PELO*
13	33175001	33225000	0.000811229	*EPC1*
10	36500001	36550000	0.000811173	*INO80*
7	18600001	18650000	0.000810598	*SAFB*
7	18600001	18650000	0.000810598	*LONP1*
7	18600001	18650000	0.000810598	*HSD11B1L*
7	18600001	18650000	0.000810598	*MICOS13*
7	18600001	18650000	0.000810598	*RPL36*
18	14675001	14725000	0.000810422	*TCF25*
18	14675001	14725000	0.000810422	*TUBB3*
18	14675001	14725000	0.000810422	*SPIRE2*
18	14675001	14725000	0.000810422	*DEF8*
18	14675001	14725000	0.000810422	*MC1R*
25	41975001	42025000	0.000810265	*PRKAR1B*
25	41975001	42025000	0.000810265	*PDGFA*
16	51400001	51450000	0.00081015	*AGRN*
16	51400001	51450000	0.00081015	*C16H1orf159*
16	51400001	51450000	0.00081015	*RNF223*
11	44625001	44675000	0.00080973	*RANBP2*
11	44625001	44675000	0.00080973	*CCDC138*
9	18525001	18575000	0.000809628	*PHIP*
16	39700001	39750000	0.000808901	*DNM3*
9	45175001	45225000	0.000808629	*HACE1*
26	21550001	21600000	0.000808135	*PAX2*
9	50250001	50300000	0.000807236	*USP45*
9	50250001	50300000	0.000807236	*CCNC*
9	50250001	50300000	0.000807236	*TSTD3*
10	52225001	52275000	0.000807179	*ALDH1A2*
17	62225001	62275000	0.000806794	*MAPKAPK5*
29	40900001	40950000	0.000806731	*EEF1G*
29	40900001	40950000	0.000806731	*TUT1*
29	40900001	40950000	0.000806731	*MTA2*
29	40900001	40950000	0.000806731	*AHNAK*
29	40900001	40950000	0.000806731	*EML3*
1	79025001	79075000	0.000806623	*LPP*
10	53025001	53075000	0.000806603	*TCF12*
9	33325001	33375000	0.000806291	*ROS1*
9	33325001	33375000	0.000806291	*DCBLD1*
18	7300001	7350000	0.000806285	*DYNLRB2*
9	15250001	15300000	0.000806261	*SENP6*
11	95650001	95700000	0.000806027	*NR6A1*
25	26850001	26900000	0.000805569	*RNF40*
25	26850001	26900000	0.000805569	*PHKG2*
25	26850001	26900000	0.000805569	*CFAP119*

25	26850001	26900000	0.000805569	*TMEM265*
25	26850001	26900000	0.000805569	*U6*
12	29225001	29275000	0.000805356	*RXFP2*
1	126250001	126300000	0.000805311	*TRPC1*
10	82300001	82350000	0.00080525	*TTC9*
5	30200001	30250000	0.000805243	*PRPF40B*
5	30200001	30250000	0.000805243	*FAM186B*
5	30200001	30250000	0.000805243	*FMNL3*
5	76350001	76400000	0.000804767	*SYT10*
9	15300001	15350000	0.000804746	*SENP6*
9	15300001	15350000	0.000804746	*U6*
13	76050001	76100000	0.000804599	*NCOA3*
13	32150001	32200000	0.000804489	*TMEM236*
13	32150001	32200000	0.000804489	*STAM*
10	72475001	72525000	0.000803559	*PPM1A*
12	35450001	35500000	0.000803229	*MICU2*
11	97450001	97500000	0.00080316	*LMX1B*
1	158200001	158250000	0.000802733	*TXLNA*
1	158200001	158250000	0.000802733	*KPNA6*
5	57175001	57225000	0.000802568	*ESYT1*
5	57175001	57225000	0.000802568	*ERBB3*
5	57175001	57225000	0.000802568	*ZC3H10*
5	57175001	57225000	0.000802568	*PA2G4*
16	51700001	51750000	0.000802465	*SPEN*
12	26175001	26225000	0.000802276	*NBEA*
4	79250001	79300000	0.000801703	*INHBA*
3	9375001	9425000	0.000801043	*COPA*
3	9375001	9425000	0.000801043	*PEX19*
3	9375001	9425000	0.000801043	*DCAF8*
24	43125001	43175000	0.000800853	*PTPN2*
10	61075001	61125000	0.000800637	*COPS2*
10	61075001	61125000	0.000800637	*GALK2*
21	2325001	2375000	0.000800609	*UBE3A*
10	54700001	54750000	0.000800493	*PRTG*
16	51600001	51650000	0.000800371	*SPEN*
16	51600001	51650000	0.000800371	*ZBTB17*
15	55875001	55925000	0.000800006	*EMSY*
1	81425001	81475000	0.000799648	*IGF2BP2*
11	61525001	61575000	0.000799501	*EHBP1*
11	61525001	61575000	0.000799501	*OTX1*
1	82875001	82925000	0.000798943	*ECE2*
1	82875001	82925000	0.000798943	*PSMD2*
1	82875001	82925000	0.000798943	*ECE2*
1	82875001	82925000	0.000798943	*CAMK2N2*
12	36475001	36525000	0.000798751	*CENPJ*
12	36475001	36525000	0.000798751	*RNF17*
10	33900001	33950000	0.000798603	*SPRED1*
15	35950001	36000000	0.00079846	*SOX6*
16	69550001	69600000	0.000798122	*PROX1*
22	56050001	56100000	0.000797997	*TMCC1*
3	49750001	49800000	0.000797872	*BCAR3*
5	33850001	33900000	0.000797432	*SLC38A2*

8	37775001	37825000	0.000797043	*KDM4C*
1	82950001	83000000	0.000796963	*DVL3*
1	82950001	83000000	0.000796963	*ABCF3*
1	82950001	83000000	0.000796963	*AP2M1*
3	50300001	50350000	0.000796689	*MTF2*
3	50300001	50350000	0.000796689	*TMED5*
5	57150001	57200000	0.00079661	*ESYT1*
5	57150001	57200000	0.00079661	*SMARCC2*
5	57150001	57200000	0.00079661	*MYL6*
5	57150001	57200000	0.00079661	*MYL6B*
5	57150001	57200000	0.00079661	*ZC3H10*
15	42475001	42525000	0.000796286	*SBF2*
17	29600001	29650000	0.000796183	*LARP1B*
5	76325001	76375000	0.000795893	*SYT10*
12	35725001	35775000	0.00079572	*XPO4*
3	22450001	22500000	0.000795667	*FMO5*
10	18975001	19025000	0.000795342	*MYO9A*
22	23700001	23750000	0.000794891	*CNTN4*
9	41925001	41975000	0.00079451	*NR2E1*
7	7725001	7775000	0.000794047	*AKAP8L*
7	7725001	7775000	0.000794047	*AKAP8*
7	7725001	7775000	0.000794047	*WIZ*
11	97425001	97475000	0.00079401	*LMX1B*
17	58625001	58675000	0.000793815	*MED13L*
2	21000001	21050000	0.000793663	*LNPK*
14	575001	625000	0.000793219	*HSF1*
14	575001	625000	0.000793219	*DGAT1*
14	575001	625000	0.000793219	*SCRT1*
14	575001	625000	0.000793219	*FBXL6*
14	575001	625000	0.000793219	*SLC52A2*
14	575001	625000	0.000793219	*TMEM249*
29	27425001	27475000	0.000792898	*OR8C26*
8	23775001	23825000	0.000792328	*MLLT3*
17	58800001	58850000	0.00079146	*MED13L*
10	54675001	54725000	0.000791285	*PRTG*
5	34225001	34275000	0.000791273	*SCAF11*
26	22725001	22775000	0.000791023	*ARMH3*
26	22725001	22775000	0.000791023	*HPS6*
11	95550001	95600000	0.000790688	*NR6A1*
3	21325001	21375000	0.000790575	*HJV*
3	21325001	21375000	0.000790575	*U1*
3	21325001	21375000	0.000790575	*U1*
3	9225001	9275000	0.000790393	*VANGL2*
16	51025001	51075000	0.000790259	*SSU72*
16	51025001	51075000	0.000790259	*FNDC10*
2	88475001	88525000	0.00079009	*TYW5*
2	88475001	88525000	0.00079009	*C2H2orf69*
2	88475001	88525000	0.00079009	*MAIP1*
13	69925001	69975000	0.000789901	*ZHX3*
13	69925001	69975000	0.000789901	*LPIN3*
1	71775001	71825000	0.000789455	*DLG1*
3	56825001	56875000	0.000789284	*SNORA62*

15	58775001	58825000	0.000789157	*KIF18A*
15	58775001	58825000	0.000789157	*METTL15*
3	50000001	50050000	0.000789051	*FNBP1L*
18	35075001	35125000	0.000788806	*ATP6V0D1*
18	35075001	35125000	0.000788806	*RIPOR1*
18	35075001	35125000	0.000788806	*AGRP*
4	43575001	43625000	0.000788271	*PTPN12*
7	17100001	17150000	0.000788172	*MYO1F*
7	17100001	17150000	0.000788172	*PRAM1*
7	17100001	17150000	0.000788172	*ZNF414*
7	17100001	17150000	0.000788172	*SNORA70*
7	44475001	44525000	0.000787894	*AFF4*
8	61200001	61250000	0.000786989	*ZCCHC7*
5	57075001	57125000	0.00078697	*RNF41*
5	57075001	57125000	0.00078697	*ANKRD52*
5	57075001	57125000	0.00078697	*NABP2*
5	57075001	57125000	0.00078697	*SLC39A5*
2	115650001	115700000	0.000786918	*AGFG1*
22	49600001	49650000	0.000786436	*DOCK3*
23	10100001	10150000	0.000786378	*BRPF3*
16	38675001	38725000	0.000786124	*FMO3*
11	95400001	95450000	0.000785893	*PSMB7*
11	95400001	95450000	0.000785893	*NEK6*
10	54600001	54650000	0.000785867	*PRTG*
20	41225001	41275000	0.000785711	*ZFR*
12	31850001	31900000	0.00078434	*PAN3*
10	86600001	86650000	0.000784024	*JDP2*
10	36200001	36250000	0.000783775	*KNL1*
12	54550001	54600000	0.000783359	*NDFIP2*
5	26050001	26100000	0.000782687	*HOXC13*
5	26050001	26100000	0.000782687	*HOXC11*
5	26050001	26100000	0.000782687	*HOXC12*
18	19150001	19200000	0.000782438	*CYLD*
20	51175001	51225000	0.000782056	*CDH12*
18	35275001	35325000	0.000781469	*RANBP10*
7	43875001	43925000	0.000780601	*ADAMTSL5*
7	43875001	43925000	0.000780601	*PCSK4*
7	43875001	43925000	0.000780601	*REEP6*
7	43875001	43925000	0.000780601	*C7H19orf25*
7	43875001	43925000	0.000780601	*APC2*
2	114975001	115025000	0.000780118	*IRS1*
2	114975001	115025000	0.000780118	*RHBDD1*
7	86975001	87025000	0.000779853	*RASA1*
17	62175001	62225000	0.000779834	*TMEM116*
9	18575001	18625000	0.000779646	*PHIP*
9	18575001	18625000	0.000779646	*U6*
9	34425001	34475000	0.000779442	*NT5DC1*
9	34425001	34475000	0.000779442	*TSPYL4*
9	34425001	34475000	0.000779442	*TSPYL1*
7	44500001	44550000	0.00077906	*AFF4*
3	63125001	63175000	0.000778923	*ADGRL2*
16	41300001	41350000	0.00077891	*VPS13D*

11	61300001	61350000	0.000778421	*EHBP1*
3	50275001	50325000	0.000778248	*TMED5*
3	50275001	50325000	0.000778248	*CCDC18*
4	114200001	114250000	0.000778008	*PRKAG2*
7	49775001	49825000	0.000777973	*KDM3B*
7	49775001	49825000	0.000777973	*REEP2*
10	52250001	52300000	0.000777921	*ALDH1A2*
26	22400001	22450000	0.000777433	*FBXW4*
16	43450001	43500000	0.000776454	*UBE4B*
5	119800001	119850000	0.000776108	*NCAPH2*
5	119800001	119850000	0.000776108	*LMF2*
5	119800001	119850000	0.000776108	*ODF3B*
5	119800001	119850000	0.000776108	*SCO2*
18	50025001	50075000	0.000776065	*RAB4B*
18	50025001	50075000	0.000776065	*EGLN2*
18	50025001	50075000	0.000776065	*SNRPA*
18	50025001	50075000	0.000776065	*CYP2F1*
18	50025001	50075000	0.000776065	*MIA*
5	57200001	57250000	0.000775763	*ERBB3*
5	57200001	57250000	0.000775763	*PA2G4*
5	57200001	57250000	0.000775763	*ZC3H10*
3	21475001	21525000	0.000775695	*ANKRD35*
3	21475001	21525000	0.000775695	*PIAS3*
3	21475001	21525000	0.000775695	*NUDT17*
3	21475001	21525000	0.000775695	*POLR3C*
3	21475001	21525000	0.000775695	*ITGA10*
17	47425001	47475000	0.000775084	*TMEM132D*
5	70250001	70300000	0.000774847	*CRY1*
5	70250001	70300000	0.000774847	*MTERF2*
15	35900001	35950000	0.000774275	*SOX6*
5	30625001	30675000	0.000774175	*TUBA1A*
9	15275001	15325000	0.000773984	*SENP6*
9	15275001	15325000	0.000773984	*U6*
1	133050001	133100000	0.000773967	*MSL2*
7	43525001	43575000	0.000773702	*ABCA7*
7	43525001	43575000	0.000773702	*ARHGAP45*
7	43525001	43575000	0.000773702	*CNN2*
7	43525001	43575000	0.000773702	*TMEM259*
17	58700001	58750000	0.000773242	*MED13L*
3	34325001	34375000	0.00077324	*WDR47*
10	59575001	59625000	0.000773093	*SPPL2A*
9	98875001	98925000	0.00077292	*QKI*
20	35450001	35500000	0.000772754	*RICTOR*
5	70000001	70050000	0.000772733	*RFX4*
21	2350001	2400000	0.000772442	*UBE3A*
16	36725001	36775000	0.000772139	*ATP1B1*
16	36725001	36775000	0.000772139	*SNORA66*
10	60125001	60175000	0.000771955	*ATP8B4*
5	56800001	56850000	0.000771809	*BAZ2A*
5	56800001	56850000	0.000771809	*RBMS2*
2	107050001	107100000	0.000771642	*NHEJ1*
19	44200001	44250000	0.000771249	*GPATCH8*

9	40100001	40150000	0.000771003	*WASF1*
2	119375001	119425000	0.000770679	*PTMA*
5	55950001	56000000	0.000770635	*MARS1*
5	55950001	56000000	0.000770635	*ARHGAP9*
5	55950001	56000000	0.000770635	*GLI1*
5	55950001	56000000	0.000770635	*DDIT3*
5	55950001	56000000	0.000770635	*MBD6*
20	38025001	38075000	0.000770407	*SKP2*
20	38025001	38075000	0.000770407	*NADK2*
10	58400001	58450000	0.000769714	*LEO1*
8	38725001	38775000	0.000769309	*KIAA2026*
5	57050001	57100000	0.000768892	*CS*
5	57050001	57100000	0.000768892	*ANKRD52*
5	57050001	57100000	0.000768892	*SLC39A5*
5	57050001	57100000	0.000768892	*COQ10A*
5	57050001	57100000	0.000768892	*NABP2*
7	49800001	49850000	0.000768256	*REEP2*
7	49800001	49850000	0.000768256	*KDM3B*
7	49800001	49850000	0.000768256	*EGR1*
16	51000001	51050000	0.000768102	*MIB2*
16	51000001	51050000	0.000768102	*CDK11B*
16	51000001	51050000	0.000768102	*MMP23*
16	51000001	51050000	0.000768102	*FNDC10*
19	40125001	40175000	0.000767824	*IKZF3*
10	82350001	82400000	0.000767742	*MAP3K9*
2	36625001	36675000	0.000767708	*MARCHF7*
12	35750001	35800000	0.000767695	*XPO4*
12	35750001	35800000	0.000767695	*EEF1AKMT1*
13	47300001	47350000	0.000767595	*SLC23A2*
2	122275001	122325000	0.000767569	*ZCCHC17*
2	122275001	122325000	0.000767569	*FABP3*
12	36350001	36400000	0.000767474	*MPHOSPH8*
12	36350001	36400000	0.000767474	*PARP4*
4	94225001	94275000	0.000767469	*MEST*
4	94225001	94275000	0.000767469	*COPG2*
4	112300001	112350000	0.000767394	*ZNF212*
12	36375001	36425000	0.000766883	*PARP4*
12	36375001	36425000	0.000766883	*MPHOSPH8*
13	57925001	57975000	0.000766878	*VAPB*
3	87350001	87400000	0.000766853	*MYSM1*
3	16250001	16300000	0.000766643	*UBAP2L*
3	16250001	16300000	0.000766643	*HAX1*
3	16250001	16300000	0.000766643	*SNORA58B*
7	45800001	45850000	0.000766627	*SKP1*
7	45800001	45850000	0.000766627	*PPP2CA*
7	45800001	45850000	0.000766627	*TCF7*
3	16225001	16275000	0.000766538	*UBAP2L*
3	16225001	16275000	0.000766538	*ATP8B2*
3	16225001	16275000	0.000766538	*AQP10*
3	16225001	16275000	0.000766538	*HAX1*
3	16225001	16275000	0.000766538	*SNORA58B*
24	43025001	43075000	0.000766418	*SPIRE1*

24	43025001	43075000	0.000766418	*CEP76*
24	43025001	43075000	0.000766418	*PSMG2*
26	15925001	15975000	0.000766227	*TBC1D12*
8	76975001	77025000	0.000765845	*GKAP1*
14	39825001	39875000	0.000765486	*ZFHX4*
5	26750001	26800000	0.000765101	*RARG*
5	26750001	26800000	0.000765101	*ITGB7*
5	26750001	26800000	0.000765101	*MFSD5*
5	26750001	26800000	0.000765101	*ESPL1*
15	84150001	84200000	0.000764949	*OR4A2*
4	46475001	46525000	0.000764762	*SRPK2*
10	37000001	37050000	0.000764698	*U6*
5	56200001	56250000	0.000764573	*LRP1*
5	56200001	56250000	0.000764573	*NXPH4*
5	56200001	56250000	0.000764573	*SHMT2*
5	56200001	56250000	0.000764573	*NDUFA4L2*
5	56200001	56250000	0.000764573	*STAC3*
9	41125001	41175000	0.000763684	*SESN1*
11	95325001	95375000	0.000763684	*NEK6*
9	41125001	41175000	0.000763684	*U12*
7	50800001	50850000	0.000763198	*UBE2D2*
9	65850001	65900000	0.000763094	*THEMIS*
7	16650001	16700000	0.000762619	*TIMM44*
7	16650001	16700000	0.000762619	*MAP2K7*
7	16650001	16700000	0.000762619	*LRRC8E*
7	16650001	16700000	0.000762619	*SNAPC2*
7	50875001	50925000	0.00076209	*CXXC5*
19	36950001	37000000	0.000761818	*SPOP*
8	38200001	38250000	0.000761764	*GLDC*
17	54250001	54300000	0.000761701	*ATP2A2*
17	54250001	54300000	0.000761701	*ANAPC7*
9	41550001	41600000	0.000761399	*FOXO3*
7	49875001	49925000	0.000761234	*HSPA9*
7	49875001	49925000	0.000761234	*ETF1*
7	49875001	49925000	0.000761234	*CTNNA1*
7	49875001	49925000	0.000761234	*SNORD63*
7	49875001	49925000	0.000761234	*SNORD63*
28	13950001	14000000	0.000760687	*BICC1*
1	107025001	107075000	0.000760665	*SMC4*
1	107025001	107075000	0.000760665	*TRIM59*
7	16975001	17025000	0.000760542	*ANGPTL4*
7	16975001	17025000	0.000760542	*RAB11B*
7	16975001	17025000	0.000760542	*KANK3*
3	105075001	105125000	0.00075979	*SCMH1*
12	34625001	34675000	0.000759528	*SACS*
12	34625001	34675000	0.000759528	*SGCG*
11	94325001	94375000	0.000759514	*STRBP*
9	98925001	98975000	0.000759287	*QKI*
1	133100001	133150000	0.000759137	*PPP2R3A*
1	133100001	133150000	0.000759137	*MSL2*
17	73125001	73175000	0.000759074	*DGCR6L*
1	52125001	52175000	0.00075827	*BBX*

14	35125001	35175000	0.000758021	*EYA1*
5	57100001	57150000	0.000757615	*RNF41*
5	57100001	57150000	0.000757615	*SMARCC2*
5	57100001	57150000	0.000757615	*NABP2*
8	38700001	38750000	0.000757471	*KIAA2026*
24	47550001	47600000	0.000757092	*SMAD2*
5	34250001	34300000	0.000756557	*SCAF11*
16	39025001	39075000	0.000756454	*PRRC2C*
17	53475001	53525000	0.000755985	*SETD1B*
17	53475001	53525000	0.000755985	*RHOF*
17	53475001	53525000	0.000755985	*TMEM120B*
29	43725001	43775000	0.000755958	*EHBP1L1*
29	43725001	43775000	0.000755958	*LTBP3*
29	43725001	43775000	0.000755958	*FAM89B*
29	43725001	43775000	0.000755958	*ZNRD2*
29	43725001	43775000	0.000755958	*KCNK7*
18	34625001	34675000	0.000755801	*CES3*
18	34625001	34675000	0.000755801	*CES2*
18	34625001	34675000	0.000755801	*RRAD*
18	34625001	34675000	0.000755801	*CIAO2B*
24	43375001	43425000	0.000755546	*LDLRAD4*
7	19950001	20000000	0.000755533	*PIAS4*
7	19950001	20000000	0.000755533	*ZBTB7A*
10	58750001	58800000	0.000755469	*DMXL2*
7	51325001	51375000	0.0007553	*CYSTM1*
24	43175001	43225000	0.000755251	*SEH1L*
24	43175001	43225000	0.000755251	*CEP192*
9	45125001	45175000	0.000754995	*HACE1*
2	52975001	53025000	0.00075497	*ARHGAP15*
15	35925001	35975000	0.000754824	*SOX6*
7	44450001	44500000	0.000754359	*AFF4*
7	44450001	44500000	0.000754359	*LEAP2*
3	50250001	50300000	0.00075358	*CCDC18*
3	50250001	50300000	0.00075358	*TMED5*
16	12025001	12075000	0.000752836	*CDC73*
16	12025001	12075000	0.000752836	*B3GALT2*
2	20775001	20825000	0.000752646	*HOXD10*
2	20775001	20825000	0.000752646	*HOXD11*
2	20775001	20825000	0.000752646	*HOXD13*
2	20775001	20825000	0.000752646	*HOXD9*
2	20775001	20825000	0.000752646	*HOXD8*
2	20775001	20825000	0.000752646	*HOXD12*
24	43225001	43275000	0.000752311	*CEP192*
7	51250001	51300000	0.000752192	*PURA*
7	51250001	51300000	0.000752192	*U6*
1	64725001	64775000	0.000751721	*GSK3B*
19	46200001	46250000	0.000751343	*CDC27*
1	81525001	81575000	0.000750996	*IGF2BP2*
10	37100001	37150000	0.000750666	*MGA*
7	50650001	50700000	0.000750304	*DNAJC18*
7	50650001	50700000	0.000750304	*SLC23A1*
7	50650001	50700000	0.000750304	*PROB1*

7	50650001	50700000	0.000750304	*SPATA24*
7	50650001	50700000	0.000750304	*MZB1*
7	43425001	43475000	0.000750146	*MED16*
7	43425001	43475000	0.000750146	*R3HDM4*
7	43425001	43475000	0.000750146	*KISS1R*
7	43425001	43475000	0.000750146	*ARID3A*
7	43425001	43475000	0.000750146	*U6*
12	35500001	35550000	0.000749773	*ZDHHC20*
12	35500001	35550000	0.000749773	*MICU2*
10	86625001	86675000	0.000749712	*JDP2*
9	50225001	50275000	0.000749415	*CCNC*
9	50225001	50275000	0.000749415	*USP45*
9	50225001	50275000	0.000749415	*TSTD3*
9	50225001	50275000	0.000749415	*U6*
9	41500001	41550000	0.00074939	*FOXO3*
4	80925001	80975000	0.00074935	*CDK13*
4	80925001	80975000	0.00074935	*SUGCT*
4	80925001	80975000	0.00074935	*MPLKIP*
1	136850001	136900000	0.000748781	*UBA5*
1	136850001	136900000	0.000748781	*NPHP3*
17	29625001	29675000	0.000748736	*LARP1B*
29	40950001	41000000	0.000748617	*GANAB*
29	40950001	41000000	0.000748617	*EML3*
29	40950001	41000000	0.000748617	*C29H11orf98*
29	40950001	41000000	0.000748617	*B3GAT3*
29	40950001	41000000	0.000748617	*INTS5*
29	40950001	41000000	0.000748617	*ROM1*
29	40950001	41000000	0.000748617	*CSKMT*
29	40950001	41000000	0.000748617	*SNORA57*
9	73875001	73925000	0.000748375	*PDE7B*
7	43350001	43400000	0.000747471	*PTBP1*
7	43350001	43400000	0.000747471	*PLPPR3*
7	43350001	43400000	0.000747471	*AZU1*
7	43350001	43400000	0.000747471	*U6*
1	111075001	111125000	0.000747422	*KCNAB1*
1	111075001	111125000	0.000747422	*SSR3*
6	67700001	67750000	0.000747404	*DCUN1D4*
15	53600001	53650000	0.000747295	*PPME1*
15	53600001	53650000	0.000747295	*P4HA3*
12	35475001	35525000	0.000746245	*MICU2*
12	35475001	35525000	0.000746245	*ZDHHC20*
9	63800001	63850000	0.000744656	*SYNCRIP*
7	49700001	49750000	0.000744592	*CDC25C*
7	49700001	49750000	0.000744592	*FAM53C*
7	49700001	49750000	0.000744592	*SLBP2*
7	49700001	49750000	0.000744592	*KDM3B*
3	51975001	52025000	0.000744429	*HFM1*
5	36450001	36500000	0.000744306	*TMEM117*
9	60075001	60125000	0.000743573	*BACH2*
5	34325001	34375000	0.000743447	*ARID2*
7	43575001	43625000	0.000742986	*SBNO2*
7	43575001	43625000	0.000742986	*POLR2E*

7	43575001	43625000	0.000742986	*GPX4*
7	43575001	43625000	0.000742986	*ARHGAP45*
2	52175001	52225000	0.000742881	*ZEB2*
5	39400001	39450000	0.000742735	*PDZRN4*
1	26175001	26225000	0.000742581	*ROBO1*
11	74850001	74900000	0.000742183	*ITSN2*
13	72200001	72250000	0.000741155	*MYBL2*
13	72200001	72250000	0.000741155	*IFT52*
5	56325001	56375000	0.000740535	*NEMP1*
5	56325001	56375000	0.000740535	*STAT6*
5	56325001	56375000	0.000740535	*NAB2*
18	35975001	36025000	0.000739861	*CDH3*
5	26650001	26700000	0.000739721	*SP7*
7	50750001	50800000	0.000739438	*UBE2D2*
9	59650001	59700000	0.000739224	*MAP3K7*
24	42925001	42975000	0.000738465	*SPIRE1*
12	35700001	35750000	0.000738405	*XPO4*
18	34650001	34700000	0.000738232	*CES4A*
18	34650001	34700000	0.000738232	*CES3*
18	34650001	34700000	0.000738232	*CES2*
7	43400001	43450000	0.000736783	*MED16*
7	43400001	43450000	0.000736783	*R3HDM4*
7	43400001	43450000	0.000736783	*PRTN3*
7	43400001	43450000	0.000736783	*ELANE*
7	43400001	43450000	0.000736783	*CFD*
7	43400001	43450000	0.000736783	*U6*
24	43250001	43300000	0.000736561	*CEP192*
2	43425001	43475000	0.000736117	*ARL6IP6*
2	43425001	43475000	0.000736117	*PRPF40A*
2	43425001	43475000	0.000736117	*U6*
12	36500001	36550000	0.000735929	*RNF17*
12	36500001	36550000	0.000735929	*CENPJ*
10	60150001	60200000	0.000735782	*ATP8B4*
19	27375001	27425000	0.000735688	*WRAP53*
19	27375001	27425000	0.000735688	*TP53*
19	27375001	27425000	0.000735688	*DNAH2*
19	27375001	27425000	0.000735688	*EFNB3*
2	325001	375000	0.000735678	*LGSN*
4	76750001	76800000	0.000735399	*OGDH*
7	51700001	51750000	0.000735205	*APBB3*
7	51700001	51750000	0.000735205	*SRA1*
7	51700001	51750000	0.000735205	*SLC35A4*
7	51700001	51750000	0.000735205	*EIF4EBP3*
10	21000001	21050000	0.000735165	*PSME1*
10	21000001	21050000	0.000735165	*PCK2*
10	21000001	21050000	0.000735165	*DCAF11*
10	21000001	21050000	0.000735165	*PSME2*
10	21000001	21050000	0.000735165	*RNF31*
10	21000001	21050000	0.000735165	*FITM1*
10	21000001	21050000	0.000735165	*EMC9*
1	82925001	82975000	0.000735005	*VWA5B2*
1	82925001	82975000	0.000735005	*ABCF3*

1	82925001	82975000	0.000735005	*ALG3*
1	82925001	82975000	0.000735005	*AP2M1*
1	82925001	82975000	0.000735005	*ECE2*
18	35050001	35100000	0.000734827	*ATP6V0D1*
18	35050001	35100000	0.000734827	*HSD11B2*
18	35050001	35100000	0.000734827	*AGRP*
19	27400001	27450000	0.000734735	*DNAH2*
19	27400001	27450000	0.000734735	*EFNB3*
19	27400001	27450000	0.000734735	*WRAP53*
11	24925001	24975000	0.000734389	*MTA3*
11	24925001	24975000	0.000734389	*U6*
5	34500001	34550000	0.000733601	*ARID2*
3	34025001	34075000	0.000733303	*SORT1*
3	34025001	34075000	0.000733303	*PSMA5*
8	79175001	79225000	0.000732396	*NAA35*
3	9150001	9200000	0.000731744	*SLAMF6*
24	42850001	42900000	0.00073159	*SPIRE1*
24	42850001	42900000	0.00073159	*PRELID3A*
1	57675001	57725000	0.000731438	*GTPBP8*
1	57675001	57725000	0.000731438	*CD200R1L*
22	13800001	13850000	0.000731164	*CTNNB1*
16	38975001	39025000	0.000730492	*PRRC2C*
5	39425001	39475000	0.000730404	*PDZRN4*
4	68800001	68850000	0.000730221	*HOXA13*
4	68800001	68850000	0.000730221	*EVX1*
10	60950001	61000000	0.00073016	*GALK2*
7	45825001	45875000	0.000729497	*PPP2CA*
13	69775001	69825000	0.00072878	*PLCG1*
13	69775001	69825000	0.00072878	*TOP1*
13	69775001	69825000	0.00072878	*U6*
5	57125001	57175000	0.000728696	*SMARCC2*
5	57125001	57175000	0.000728696	*RNF41*
5	57125001	57175000	0.000728696	*MYL6*
5	57125001	57175000	0.000728696	*MYL6B*
24	42800001	42850000	0.000728388	*AFG3L2*
24	42800001	42850000	0.000728388	*TUBB6*
4	46450001	46500000	0.000728059	*SRPK2*
9	18600001	18650000	0.000727933	*PHIP*
9	18600001	18650000	0.000727933	*U6*
13	22750001	22800000	0.00072714	*MLLT10*
6	66600001	66650000	0.00072595	*NFXL1*
16	38250001	38300000	0.000725877	*PRRX1*
20	14050001	14100000	0.000725863	*ADAMTS6*
5	56675001	56725000	0.000725823	*HSD17B6*
5	56675001	56725000	0.000725823	*PRIM1*
5	56675001	56725000	0.000725823	*NACA*
21	45800001	45850000	0.000725124	*RALGAPA1*
1	64750001	64800000	0.000725082	*GSK3B*
23	375001	425000	0.000725075	*KHDRBS2*
8	22575001	22625000	0.000724755	*IFNT3*
8	22575001	22625000	0.000724755	*IFNT2*
14	475001	525000	0.000724544	*CYHR1*

14	475001	525000	0.000724544	*TONSL*
14	475001	525000	0.000724544	*VPS28*
14	475001	525000	0.000724544	*KIFC2*
11	95575001	95625000	0.000724067	*NR6A1*
2	124700001	124750000	0.000723412	*YTHDF2*
2	124700001	124750000	0.000723412	*U6*
2	44550001	44600000	0.000723264	*NEB*
13	38025001	38075000	0.000722693	*BANF2*
22	45475001	45525000	0.000722135	*ERC2*
14	39975001	40025000	0.000721903	*ZFHX4*
19	37875001	37925000	0.000721887	*HOXB3*
19	37875001	37925000	0.000721887	*HOXB7*
19	37875001	37925000	0.000721887	*HOXB8*
19	37875001	37925000	0.000721887	*HOXB4*
19	37875001	37925000	0.000721887	*HOXB5*
19	37875001	37925000	0.000721887	*HOXB6*
14	450001	500000	0.000721104	*CYHR1*
14	450001	500000	0.000721104	*KIFC2*
14	450001	500000	0.000721104	*TONSL*
14	450001	500000	0.000721104	*FOXH1*
24	48475001	48525000	0.000720958	*CTIF*
24	48475001	48525000	0.000720958	*U6*
24	48900001	48950000	0.000720816	*DYM*
16	51375001	51425000	0.000720804	*C16H1orf159*
16	51375001	51425000	0.000720804	*AGRN*
16	51375001	51425000	0.000720804	*RNF223*
16	39475001	39525000	0.000720575	*DNM3*
3	52025001	52075000	0.000720301	*HFM1*
12	35775001	35825000	0.000720208	*EEF1AKMT1*
12	35775001	35825000	0.000720208	*XPO4*
12	35775001	35825000	0.000720208	*IL17D*
10	59125001	59175000	0.000720203	*CYP19A1*
20	62825001	62875000	0.000720128	*MARCHF6*
4	85075001	85125000	0.000719906	*KCND2*
15	35800001	35850000	0.000719203	*SOX6*
7	50775001	50825000	0.000719036	*UBE2D2*
29	40925001	40975000	0.00071841	*TUT1*
29	40925001	40975000	0.00071841	*EML3*
29	40925001	40975000	0.00071841	*MTA2*
29	40925001	40975000	0.00071841	*GANAB*
29	40925001	40975000	0.00071841	*B3GAT3*
29	40925001	40975000	0.00071841	*ROM1*
29	40925001	40975000	0.00071841	*EEF1G*
24	42900001	42950000	0.000718377	*SPIRE1*
6	66575001	66625000	0.000718355	*NFXL1*
6	66575001	66625000	0.000718355	*CORIN*
21	26600001	26650000	0.000718354	*ABHD17C*
11	74875001	74925000	0.000718336	*ITSN2*
1	132225001	132275000	0.000717469	*NCK1*
3	9475001	9525000	0.000717343	*ATP1A4*
3	9475001	9525000	0.000717343	*CASQ1*
3	9475001	9525000	0.000717343	*U6*

9	18550001	18600000	0.000717113	*PHIP*
6	25075001	25125000	0.000716622	*TRMT10A*
6	25075001	25125000	0.000716622	*C6H4orf17*
9	45200001	45250000	0.000716449	*HACE1*
1	113750001	113800000	0.000716093	*Metazoa_SRP*
1	64850001	64900000	0.000715973	*GSK3B*
1	64775001	64825000	0.000715838	*GSK3B*
1	8925001	8975000	0.000715669	*H4C3*
24	42825001	42875000	0.000715281	*PRELID3A*
24	42825001	42875000	0.000715281	*AFG3L2*
4	69200001	69250000	0.000715184	*SKAP2*
18	20975001	21025000	0.000715145	*TOX3*
3	34050001	34100000	0.000715118	*SORT1*
9	65825001	65875000	0.00071497	*THEMIS*
26	22375001	22425000	0.000714717	*FBXW4*
16	42400001	42450000	0.000714463	*MTOR*
16	42400001	42450000	0.000714463	*ANGPTL7*
18	35900001	35950000	0.000713894	*ZFP90*
13	57475001	57525000	0.000712566	*GNAS*
13	57475001	57525000	0.000712566	*GNAS*
24	49025001	49075000	0.000710807	*DYM*
9	65875001	65925000	0.000709965	*THEMIS*
1	111100001	111150000	0.000709856	*KCNAB1*
1	111100001	111150000	0.000709856	*SSR3*
13	57650001	57700000	0.000708573	*NPEPL1*
13	57650001	57700000	0.000708573	*STX16*
7	17075001	17125000	0.000708141	*HNRNPM*
7	17075001	17125000	0.000708141	*PRAM1*
7	17075001	17125000	0.000708141	*MYO1F*
7	17075001	17125000	0.000708141	*ZNF414*
13	57850001	57900000	0.000708137	*APCDD1L*
9	41150001	41200000	0.000707999	*SESN1*
9	41150001	41200000	0.000707999	*ARMC2*
9	41825001	41875000	0.000707913	*AFG1L*
9	41825001	41875000	0.000707913	*SNX3*
8	79125001	79175000	0.000707491	*NAA35*
8	79150001	79200000	0.000706899	*NAA35*
7	49750001	49800000	0.000706893	*KDM3B*
2	90925001	90975000	0.000706482	*BMPR2*
24	34600001	34650000	0.000706175	*ESCO1*
24	34600001	34650000	0.000706175	*SNRPD1*
5	70100001	70150000	0.000706111	*RIC8B*
3	109550001	109600000	0.000705943	*THRAP3*
3	109550001	109600000	0.000705943	*SH3D21*
8	38750001	38800000	0.000705816	*KIAA2026*
2	91075001	91125000	0.000705615	*FAM117B*
1	71750001	71800000	0.000705415	*DLG1*
9	94450001	94500000	0.000704893	*SNX9*
1	81450001	81500000	0.000704526	*IGF2BP2*
14	35100001	35150000	0.000704314	*EYA1*
19	34250001	34300000	0.000704302	*PRPSAP2*
19	34250001	34300000	0.000704302	*SLC5A10*

19	34250001	34300000	0.000704302	*FAM83G*
10	60175001	60225000	0.000704023	*ATP8B4*
10	59675001	59725000	0.000703916	*TRPM7*
11	68250001	68300000	0.000703794	*MXD1*
11	94975001	95025000	0.000703743	*DENND1A*
16	39000001	39050000	0.000703597	*PRRC2C*
24	42875001	42925000	0.000703322	*SPIRE1*
20	14025001	14075000	0.000703283	*ADAMTS6*
20	14025001	14075000	0.000703283	*CENPK*
5	34375001	34425000	0.000703092	*ARID2*
13	51400001	51450000	0.000702837	*C13H20orf27*
13	51400001	51450000	0.000702837	*HSPA12B*
13	51400001	51450000	0.000702837	*CDC25B*
13	51400001	51450000	0.000702837	*SPEF1*
13	51400001	51450000	0.000702837	*CENPB*
10	61750001	61800000	0.000702609	*FBN1*
1	69975001	70025000	0.000699476	*OSBPL11*
7	16600001	16650000	0.000699177	*EVI5L*
7	16600001	16650000	0.000699177	*PRR36*
10	58975001	59025000	0.000698907	*GLDN*
23	30275001	30325000	0.000698799	*ZSCAN12*
4	68875001	68925000	0.000698647	*HOXA3*
4	68875001	68925000	0.000698647	*HOXA6*
4	68875001	68925000	0.000698647	*HOXA9*
4	68875001	68925000	0.000698647	*HOXA5*
4	68875001	68925000	0.000698647	*HOXA7*
4	68875001	68925000	0.000698647	*HOXA4*
16	51625001	51675000	0.000698336	*SPEN*
16	51625001	51675000	0.000698336	*ZBTB17*
9	41800001	41850000	0.000697676	*AFG1L*
26	15175001	15225000	0.000697206	*ASMTL*
26	15175001	15225000	0.000697206	*SLC25A6*
8	38500001	38550000	0.000697193	*IL33*
24	43100001	43150000	0.000697131	*PTPN2*
5	76300001	76350000	0.00069705	*SYT10*
26	23125001	23175000	0.00069675	*SUFU*
7	44425001	44475000	0.000696642	*AFF4*
7	44425001	44475000	0.000696642	*GDF9*
7	44425001	44475000	0.000696642	*UQCRQ*
7	44425001	44475000	0.000696642	*LEAP2*
1	110950001	111000000	0.000696369	*TIPARP*
18	7725001	7775000	0.00069572	*GCSH*
18	7725001	7775000	0.00069572	*ATMIN*
18	7725001	7775000	0.00069572	*C18H16orf46*
18	7725001	7775000	0.00069572	*U6*
5	34350001	34400000	0.000695457	*ARID2*
8	300001	350000	0.000694401	*MFSD14B*
10	59400001	59450000	0.000693661	*AP4E1*
5	58050001	58100000	0.000693439	*OR6C202*
5	58050001	58100000	0.000693439	*OR6C207*
2	91100001	91150000	0.000693363	*FAM117B*
2	91100001	91150000	0.000693363	*ICA1L*

22	13850001	13900000	0.000693112	*ULK4*
19	11300001	11350000	0.000692944	*BRIP1*
1	111125001	111175000	0.000692889	*KCNAB1*
1	126275001	126325000	0.000692798	*TRPC1*
26	32700001	32750000	0.000692773	*GPAM*
11	30150001	30200000	0.000692669	*FBXO11*
19	27025001	27075000	0.000692526	*KCTD11*
19	27025001	27075000	0.000692526	*NEURL4*
19	27025001	27075000	0.000692526	*TNK1*
19	27025001	27075000	0.000692526	*PLSCR3*
19	27025001	27075000	0.000692526	*TMEM95*
17	13150001	13200000	0.000692271	*ABCE1*
17	13150001	13200000	0.000692271	*ANAPC10*
17	13150001	13200000	0.000692271	*OTUD4*
17	13150001	13200000	0.000692271	*U6*
22	7850001	7900000	0.000692033	*CLASP2*
3	34000001	34050000	0.000691928	*PSMA5*
3	34000001	34050000	0.000691928	*SORT1*
7	52725001	52775000	0.000691526	*ARAP3*
7	52725001	52775000	0.000691526	*FCHSD1*
7	52725001	52775000	0.000691526	*HDAC3*
7	52725001	52775000	0.000691526	*RELL2*
13	57875001	57925000	0.000691284	*APCDD1L*
13	57875001	57925000	0.000691284	*VAPB*
13	57900001	57950000	0.000691132	*VAPB*
13	57900001	57950000	0.000691132	*APCDD1L*
1	126200001	126250000	0.000690955	*PCOLCE2*
21	58925001	58975000	0.000690938	*SERPINA6*
9	74075001	74125000	0.000690895	*PDE7B*
5	69725001	69775000	0.000690403	*POLR3B*
1	107050001	107100000	0.000690265	*SMC4*
1	107050001	107100000	0.000690265	*IFT80*
9	74050001	74100000	0.000690198	*PDE7B*
10	18775001	18825000	0.000690086	*MYO9A*
11	68225001	68275000	0.000690058	*MXD1*
11	68225001	68275000	0.000690058	*SNRNP27*
11	68225001	68275000	0.000690058	*GMCL1*
9	60425001	60475000	0.000690007	*MDN1*
9	41475001	41525000	0.000689583	*FOXO3*
24	42975001	43025000	0.000689559	*SPIRE1*
5	56700001	56750000	0.000688152	*NACA*
5	56700001	56750000	0.000688152	*PRIM1*
9	96175001	96225000	0.000688109	*IGF2R*
4	94250001	94300000	0.000687034	*COPG2*
4	94250001	94300000	0.000687034	*MEST*
1	88300001	88350000	0.000686837	*PLCXD1*
26	23000001	23050000	0.000685255	*PSD*
26	23000001	23050000	0.000685255	*GBF1*
26	23000001	23050000	0.000685255	*NFKB2*
26	23000001	23050000	0.000685255	*CUEDC2*
26	23000001	23050000	0.000685255	*FBXL15*
26	15950001	16000000	0.000685057	*TBC1D12*

5	70050001	70100000	0.000684909	*RIC8B*
5	70050001	70100000	0.000684909	*RFX4*
21	33375001	33425000	0.000684402	*SIN3A*
21	33375001	33425000	0.000684402	*PTPN9*
19	36925001	36975000	0.000684082	*SPOP*
13	57700001	57750000	0.000683984	*STX16*
18	35525001	35575000	0.000683701	*NFATC3*
15	84125001	84175000	0.00068317	*OR4A2*
17	56875001	56925000	0.000682824	*SUDS3*
3	109900001	109950000	0.000682793	*AGO1*
3	109900001	109950000	0.000682793	*AGO4*
20	38125001	38175000	0.000682581	*UGT3A2*
20	38125001	38175000	0.000682581	*LMBRD2*
10	58950001	59000000	0.000682289	*GLDN*
24	48575001	48625000	0.000681975	*SMAD7*
5	11975001	12025000	0.000681928	*METTL25*
10	59375001	59425000	0.000681844	*AP4E1*
16	39725001	39775000	0.000681387	*DNM3*
16	21750001	21800000	0.00068134	*RRP15*
3	32250001	32300000	0.000681155	*DRAM2*
3	32250001	32300000	0.000681155	*CEPT1*
7	52675001	52725000	0.000679705	*HDAC3*
8	78975001	79025000	0.000679622	*AGTPBP1*
8	38675001	38725000	0.0006791	*KIAA2026*
8	38675001	38725000	0.0006791	*RANBP6*
17	52225001	52275000	0.000678675	*SBNO1*
17	52225001	52275000	0.000678675	*KMT5A*
3	50175001	50225000	0.000677703	*CCDC18*
26	22900001	22950000	0.000677674	*GBF1*
12	36200001	36250000	0.000677522	*ZMYM5*
26	22975001	23025000	0.000677401	*GBF1*
26	22975001	23025000	0.000677401	*NFKB2*
7	51300001	51350000	0.000676439	*PURA*
7	51300001	51350000	0.000676439	*IGIP*
11	97300001	97350000	0.000676361	*MVB12B*
24	43200001	43250000	0.000674892	*CEP192*
24	43200001	43250000	0.000674892	*SEH1L*
16	12150001	12200000	0.000674677	*RO60*
16	12150001	12200000	0.000674677	*GLRX2*
16	12150001	12200000	0.000674677	*UCHL5*
22	23725001	23775000	0.000673914	*CNTN4*
1	71725001	71775000	0.000672817	*DLG1*
1	71725001	71775000	0.000672817	*U6*
4	94275001	94325000	0.000671968	*COPG2*
24	43050001	43100000	0.000671822	*CEP76*
24	43050001	43100000	0.000671822	*PSMG2*
24	43050001	43100000	0.000671822	*PTPN2*
4	68850001	68900000	0.000671709	*HOXA10*
4	68850001	68900000	0.000671709	*HOXA11*
4	68850001	68900000	0.000671709	*HOXA6*
4	68850001	68900000	0.000671709	*HOXA9*
4	68850001	68900000	0.000671709	*HOXA7*

4	68850001	68900000	0.000671709	*HOXA5*
9	34400001	34450000	0.00067136	*TSPYL1*
9	34400001	34450000	0.00067136	*TSPYL4*
18	35300001	35350000	0.000671222	*RANBP10*
18	35300001	35350000	0.000671222	*TSNAXIP1*
18	35300001	35350000	0.000671222	*CENPT*
18	35300001	35350000	0.000671222	*THAP11*
9	74025001	74075000	0.000670995	*PDE7B*
21	33400001	33450000	0.000670847	*SIN3A*
22	7825001	7875000	0.000670407	*CLASP2*
3	119600001	119650000	0.000670119	*OR9S36*
3	119600001	119650000	0.000670119	*OR9S40*
3	119600001	119650000	0.000670119	*OR9S15*
20	23700001	23750000	0.000669475	*PLPP1*
20	23700001	23750000	0.000669475	*MTREX*
11	94275001	94325000	0.00066922	*STRBP*
22	23775001	23825000	0.000669092	*CNTN4*
17	57000001	57050000	0.000668826	*TAOK3*
17	54375001	54425000	0.000668183	*RAD9B*
17	54375001	54425000	0.000668183	*PPTC7*
17	54375001	54425000	0.000668183	*VPS29*
10	60025001	60075000	0.00066751	*SLC27A2*
1	136900001	136950000	0.000666876	*ACAD11*
1	136900001	136950000	0.000666876	*UBA5*
3	50150001	50200000	0.000665948	*CCDC18*
1	107150001	107200000	0.000665264	*IFT80*
18	14725001	14775000	0.000665003	*DEF8*
18	14725001	14775000	0.000665003	*DBNDD1*
18	14725001	14775000	0.000665003	*GAS8*
1	64800001	64850000	0.00066438	*GSK3B*
9	74225001	74275000	0.000664019	*MTFR2*
9	74225001	74275000	0.000664019	*BCLAF1*
17	29450001	29500000	0.000662987	*PGRMC2*
1	82900001	82950000	0.000662667	*VWA5B2*
1	82900001	82950000	0.000662667	*ECE2*
1	82900001	82950000	0.000662667	*ECE2*
1	82900001	82950000	0.000662667	*ALG3*
1	82900001	82950000	0.000662667	*CAMK2N2*
9	50200001	50250000	0.000661661	*CCNC*
9	50200001	50250000	0.000661661	*PRDM13*
9	50200001	50250000	0.000661661	*U6*
10	18700001	18750000	0.000661148	*MYO9A*
9	50175001	50225000	0.000660772	*PRDM13*
24	47525001	47575000	0.000660194	*SMAD2*
5	26025001	26075000	0.000659926	*HOXC10*
5	26025001	26075000	0.000659926	*HOXC9*
5	26025001	26075000	0.000659926	*HOXC11*
5	26025001	26075000	0.000659926	*MIR196A2*
1	133075001	133125000	0.000659082	*MSL2*
1	133075001	133125000	0.000659082	*PPP2R3A*
1	87225001	87275000	0.000659035	*PEX5L*
12	35800001	35850000	0.000658419	*IL17D*

12	35800001	35850000	0.000658419	*EEF1AKMT1*
5	30775001	30825000	0.000658026	*KMT2D*
5	30775001	30825000	0.000658026	*PRKAG1*
5	30775001	30825000	0.000658026	*DDN*
18	35025001	35075000	0.00065769	*ATP6V0D1*
18	35025001	35075000	0.00065769	*HSD11B2*
18	35025001	35075000	0.00065769	*ZDHHC1*
11	68825001	68875000	0.000657639	*GALNT14*
3	33975001	34025000	0.000657299	*PSMA5*
3	33975001	34025000	0.000657299	*SYPL2*
10	58300001	58350000	0.000657127	*MAPK6*
10	80900001	80950000	0.000656451	*DCAF5*
18	34725001	34775000	0.000656367	*CBFB*
18	34725001	34775000	0.000656367	*PHAF1*
14	400001	450000	0.000655792	*ARHGAP39*
14	400001	450000	0.000655792	*LRRC24*
14	400001	450000	0.000655792	*RECQL4*
14	400001	450000	0.000655792	*PPP1R16A*
14	400001	450000	0.000655792	*GPT*
14	400001	450000	0.000655792	*LRRC14*
14	400001	450000	0.000655792	*C14H8orf82*
14	400001	450000	0.000655792	*MFSD3*
11	30200001	30250000	0.000655584	*FBXO11*
26	50175001	50225000	0.000655417	*CYP2E1*
26	50175001	50225000	0.000655417	*OR13A29*
18	35500001	35550000	0.000654758	*NFATC3*
18	35500001	35550000	0.000654758	*DUS2*
18	44800001	44850000	0.000654237	*PDCD2L*
18	44800001	44850000	0.000654237	*GPI*
18	44800001	44850000	0.000654237	*UBA2*
4	54050001	54100000	0.000654192	*FOXP2*
12	25950001	26000000	0.000653831	*NBEA*
12	25950001	26000000	0.000653831	*MAB21L1*
12	36075001	36125000	0.000653522	*GJB2*
12	36075001	36125000	0.000653522	*GJA3*
9	74275001	74325000	0.000653279	*BCLAF1*
2	91475001	91525000	0.000653171	*CYP20A1*
3	99675001	99725000	0.000653159	*FAAH*
9	60350001	60400000	0.000652119	*CASP8AP2*
9	60350001	60400000	0.000652119	*GJA10*
9	60350001	60400000	0.000652119	*U7*
11	30175001	30225000	0.000651971	*FBXO11*
18	34700001	34750000	0.000651549	*CBFB*
2	121350001	121400000	0.000651494	*U6*
18	35600001	35650000	0.00065128	*PLA2G15*
18	35600001	35650000	0.00065128	*NFATC3*
18	35600001	35650000	0.00065128	*SLC7A6*
18	35600001	35650000	0.00065128	*ESRP2*
5	76275001	76325000	0.000651025	*SYT10*
28	16550001	16600000	0.000650162	*CDK1*
8	38225001	38275000	0.000648299	*GLDC*
10	59425001	59475000	0.000647676	*AP4E1*

10	59725001	59775000	0.000646683	*TRPM7*
10	59725001	59775000	0.000646683	*USP8*
18	35250001	35300000	0.000646028	*RANBP10*
18	35250001	35300000	0.000646028	*GFOD2*
22	13825001	13875000	0.000645944	*ULK4*
22	13825001	13875000	0.000645944	*CTNNB1*
10	58600001	58650000	0.000645928	*LYSMD2*
10	58600001	58650000	0.000645928	*TMOD2*
10	58600001	58650000	0.000645928	*SCG3*
26	17475001	17525000	0.000644507	*BLNK*
26	17475001	17525000	0.000644507	*ZNF518A*
3	84200001	84250000	0.000644421	*NFIA*
15	35875001	35925000	0.000643809	*SOX6*
24	49075001	49125000	0.000643666	*DYM*
10	59925001	59975000	0.000643112	*GABPB1*
14	425001	475000	0.000642769	*RECQL4*
14	425001	475000	0.000642769	*PPP1R16A*
14	425001	475000	0.000642769	*KIFC2*
14	425001	475000	0.000642769	*GPT*
14	425001	475000	0.000642769	*LRRC14*
14	425001	475000	0.000642769	*MFSD3*
14	425001	475000	0.000642769	*FOXH1*
24	49050001	49100000	0.000642308	*DYM*
10	58350001	58400000	0.000642308	*MAPK6*
1	107100001	107150000	0.000642269	*IFT80*
9	33475001	33525000	0.000641546	*VGLL2*
9	33475001	33525000	0.000641546	*ROS1*
6	67875001	67925000	0.000641334	*SGCB*
6	67875001	67925000	0.000641334	*SPATA18*
6	67875001	67925000	0.000641334	*LRRC66*
7	65300001	65350000	0.000641214	*FAM114A2*
7	65300001	65350000	0.000641214	*MFAP3*
7	65300001	65350000	0.000641214	*U6*
12	36525001	36575000	0.000640574	*RNF17*
10	58725001	58775000	0.000640351	*DMXL2*
9	41950001	42000000	0.000640257	*NR2E1*
19	36900001	36950000	0.000639221	*SPOP*
19	36900001	36950000	0.000639221	*FAM117A*
19	36900001	36950000	0.000639221	*SLC35B1*
19	36900001	36950000	0.000639221	*SNORA70*
26	37825001	37875000	0.000639204	*EMX2*
7	43375001	43425000	0.000638995	*PLPPR3*
7	43375001	43425000	0.000638995	*MED16*
7	43375001	43425000	0.000638995	*PRTN3*
7	43375001	43425000	0.000638995	*PTBP1*
7	43375001	43425000	0.000638995	*ELANE*
7	43375001	43425000	0.000638995	*AZU1*
7	43375001	43425000	0.000638995	*CFD*
7	43375001	43425000	0.000638995	*U6*
9	39975001	40025000	0.000638499	*CDC40*
9	39975001	40025000	0.000638499	*METTL24*
9	41700001	41750000	0.000638439	*AFG1L*

1	65150001	65200000	0.000638361	*FSTL1*
1	26200001	26250000	0.000638184	*ROBO1*
7	9975001	10025000	0.000637886	*OR7A88*
1	71475001	71525000	0.000637719	*MELTF*
1	71475001	71525000	0.000637719	*DLG1*
2	88450001	88500000	0.000637698	*C2H2orf69*
2	88450001	88500000	0.000637698	*TYW5*
1	71500001	71550000	0.000637466	*DLG1*
1	71500001	71550000	0.000637466	*MELTF*
2	91175001	91225000	0.000637361	*WDR12*
2	91175001	91225000	0.000637361	*ICA1L*
18	35550001	35600000	0.00063717	*NFATC3*
18	35550001	35600000	0.00063717	*U6*
7	41675001	41725000	0.000637021	*OR2AJ9*
10	33825001	33875000	0.000636818	*SPRED1*
10	33825001	33875000	0.000636818	*SNORA70*
12	36550001	36600000	0.000636346	*RNF17*
7	17000001	17050000	0.000636167	*MARCHF2*
7	17000001	17050000	0.000636167	*RAB11B*
7	17000001	17050000	0.000636167	*ANGPTL4*
24	48425001	48475000	0.000636014	*CTIF*
21	45775001	45825000	0.000635859	*RALGAPA1*
4	68825001	68875000	0.000634514	*HOXA10*
4	68825001	68875000	0.000634514	*HOXA11*
4	68825001	68875000	0.000634514	*HOXA13*
9	38975001	39025000	0.000634489	*REV3L*
26	22350001	22400000	0.000634444	*FBXW4*
26	22350001	22400000	0.000634444	*DPCD*
5	56300001	56350000	0.000634189	*STAT6*
5	56300001	56350000	0.000634189	*LRP1*
5	56300001	56350000	0.000634189	*NAB2*
5	56300001	56350000	0.000634189	*SNORA62*
24	48775001	48825000	0.000634149	*DYM*
19	27500001	27550000	0.000634091	*DNAH2*
19	27500001	27550000	0.000634091	*KDM6B*
19	27500001	27550000	0.000634091	*NAA38*
19	27500001	27550000	0.000634091	*CYB5D1*
19	27500001	27550000	0.000634091	*TMEM88*
24	42950001	43000000	0.000633981	*SPIRE1*
18	35375001	35425000	0.000633668	*PSKH1*
18	35375001	35425000	0.000633668	*EDC4*
18	35375001	35425000	0.000633668	*PSMB10*
18	35375001	35425000	0.000633668	*LCAT*
18	35375001	35425000	0.000633668	*NRN1L*
3	21400001	21450000	0.000633475	*RBM8A*
3	21400001	21450000	0.000633475	*ANKRD34A*
3	21400001	21450000	0.000633475	*POLR3GL*
3	21400001	21450000	0.000633475	*GNRHR2*
13	22725001	22775000	0.000632749	*MLLT10*
13	22725001	22775000	0.000632749	*SKIDA1*
26	22950001	23000000	0.000632051	*GBF1*
18	35150001	35200000	0.000631895	*CTCF*

18	35150001	35200000	0.000631895	*CARMIL2*
10	59550001	59600000	0.00063176	*SPPL2A*
16	12000001	12050000	0.000631715	*CDC73*
18	35775001	35825000	0.000631526	*SMPD3*
24	48600001	48650000	0.000631299	*SMAD7*
19	27050001	27100000	0.000631288	*NLGN2*
19	27050001	27100000	0.000631288	*TNK1*
19	27050001	27100000	0.000631288	*KCTD11*
19	27050001	27100000	0.000631288	*PLSCR3*
19	27050001	27100000	0.000631288	*TMEM95*
19	27050001	27100000	0.000631288	*TMEM256*
19	27050001	27100000	0.000631288	*SPEM1*
7	52700001	52750000	0.000631206	*HDAC3*
7	52700001	52750000	0.000631206	*FCHSD1*
7	52700001	52750000	0.000631206	*RELL2*
7	52700001	52750000	0.000631206	*ARAP3*
5	70075001	70125000	0.000630911	*RIC8B*
6	100001	150000	0.000630598	*APELA*
10	33875001	33925000	0.000630564	*SPRED1*
26	22925001	22975000	0.00063	*GBF1*
9	60375001	60425000	0.00062988	*CASP8AP2*
9	60375001	60425000	0.00062988	*MDN1*
9	60375001	60425000	0.00062988	*U7*
18	34600001	34650000	0.000628867	*CES2*
18	34600001	34650000	0.000628867	*CDH16*
18	34600001	34650000	0.000628867	*RRAD*
18	34600001	34650000	0.000628867	*CIAO2B*
18	35925001	35975000	0.000626766	*CDH3*
5	57775001	57825000	0.000626368	*OR6C22*
5	57775001	57825000	0.000626368	*OR6C264*
10	58525001	58575000	0.000626243	*TMOD3*
10	58525001	58575000	0.000626243	*TMOD2*
18	35675001	35725000	0.000626008	*PRMT7*
18	35675001	35725000	0.000626008	*SLC7A6OS*
18	35675001	35725000	0.000626008	*SMPD3*
5	55975001	56025000	0.000625949	*MARS1*
5	55975001	56025000	0.000625949	*INHBC*
5	55975001	56025000	0.000625949	*GLI1*
5	55975001	56025000	0.000625949	*ARHGAP9*
5	55975001	56025000	0.000625949	*INHBE*
3	52000001	52050000	0.000625353	*HFM1*
1	107125001	107175000	0.00062533	*IFT80*
18	34675001	34725000	0.000625142	*CBFB*
18	34675001	34725000	0.000625142	*CES4A*
11	29525001	29575000	0.000625099	*STPG4*
11	29525001	29575000	0.000625099	*TTC7A*
7	51275001	51325000	0.000624661	*PURA*
7	51275001	51325000	0.000624661	*IGIP*
18	34750001	34800000	0.000624189	*PHAF1*
18	34750001	34800000	0.000624189	*TRADD*
18	34750001	34800000	0.000624189	*B3GNT9*
18	34750001	34800000	0.000624189	*FBXL8*

18	34750001	34800000	0.000624189	*CBFB*
1	106925001	106975000	0.000623873	*KPNA4*
1	106925001	106975000	0.000623873	*SCARNA7*
11	61325001	61375000	0.000623279	*EHBP1*
17	56950001	57000000	0.000623069	*TAOK3*
18	35000001	35050000	0.000621222	*ZDHHC1*
18	35000001	35050000	0.000621222	*HSD11B2*
18	35000001	35050000	0.000621222	*TPPP3*
18	35000001	35050000	0.000621222	*ATP6V0D1*
3	95325001	95375000	0.000620724	*FAF1*
3	95325001	95375000	0.000620724	*CDKN2C*
10	59350001	59400000	0.000620191	*TNFAIP8L3*
10	59350001	59400000	0.000620191	*AP4E1*
16	39500001	39550000	0.000619474	*DNM3*
19	27075001	27125000	0.000618815	*NLGN2*
19	27075001	27125000	0.000618815	*CHRNB1*
19	27075001	27125000	0.000618815	*PLSCR3*
19	27075001	27125000	0.000618815	*TMEM102*
19	27075001	27125000	0.000618815	*SPEM2*
19	27075001	27125000	0.000618815	*SPEM1*
19	27075001	27125000	0.000618815	*TMEM256*
3	109875001	109925000	0.000618415	*AGO1*
13	72225001	72275000	0.000617624	*MYBL2*
11	97350001	97400000	0.000617496	*LMX1B*
7	43550001	43600000	0.000617329	*ARHGAP45*
7	43550001	43600000	0.000617329	*ABCA7*
7	43550001	43600000	0.000617329	*SBNO2*
7	43550001	43600000	0.000617329	*POLR2E*
7	43550001	43600000	0.000617329	*GPX4*
9	60400001	60450000	0.000616663	*MDN1*
9	60400001	60450000	0.000616663	*CASP8AP2*
7	49725001	49775000	0.000616512	*KDM3B*
7	49725001	49775000	0.000616512	*FAM53C*
7	49725001	49775000	0.000616512	*CDC25C*
7	49725001	49775000	0.000616512	*SLBP2*
10	58450001	58500000	0.000616367	*TMOD3*
10	58375001	58425000	0.000615945	*LEO1*
8	38525001	38575000	0.000614318	*IL33*
10	18825001	18875000	0.000614196	*MYO9A*
10	59300001	59350000	0.000613938	*TNFAIP8L3*
16	39800001	39850000	0.000613321	*DNM3*
10	85975001	86025000	0.000611488	*AREL1*
10	85975001	86025000	0.000611488	*FCF1*
10	85975001	86025000	0.000611488	*YLPM1*
26	23475001	23525000	0.000610889	*AS3MT*
1	71700001	71750000	0.000609834	*DLG1*
1	71700001	71750000	0.000609834	*U6*
16	39400001	39450000	0.000609609	*DNM3*
24	43075001	43125000	0.000609538	*PTPN2*
24	43075001	43125000	0.000609538	*PSMG2*
10	59150001	59200000	0.000609073	*CYP19A1*
18	35575001	35625000	0.000608908	*NFATC3*

18	35575001	35625000	0.000608908	*ESRP2*
18	35575001	35625000	0.000608908	*PLA2G15*
18	35575001	35625000	0.000608908	*U6*
13	57675001	57725000	0.000608656	*STX16*
13	57675001	57725000	0.000608656	*NPEPL1*
3	50225001	50275000	0.00060861	*CCDC18*
4	68900001	68950000	0.000608455	*HOXA3*
4	68900001	68950000	0.000608455	*HOXA1*
4	68900001	68950000	0.000608455	*HOXA4*
4	68900001	68950000	0.000608455	*HOXA2*
4	68900001	68950000	0.000608455	*HOXA5*
9	39000001	39050000	0.000608205	*REV3L*
17	58850001	58900000	0.000607913	*MED13L*
9	39550001	39600000	0.000607822	*CDK19*
26	50425001	50475000	0.000607662	*OR12J2*
3	50200001	50250000	0.000606707	*CCDC18*
16	60550001	60600000	0.000605796	*ABL2*
18	35625001	35675000	0.000605144	*SLC7A6*
18	35625001	35675000	0.000605144	*PLA2G15*
18	35625001	35675000	0.000605144	*SLC7A6OS*
11	97400001	97450000	0.000604499	*LMX1B*
9	38900001	38950000	0.00060428	*REV3L*
3	100975001	101025000	0.000603239	*ZSWIM5*
3	100975001	101025000	0.000603239	*UROD*
18	35450001	35500000	0.000602253	*DUS2*
18	35450001	35500000	0.000602253	*DPEP2*
18	35450001	35500000	0.000602253	*DPEP3*
18	35450001	35500000	0.000602253	*DDX28*
1	64825001	64875000	0.000602209	*GSK3B*
10	86225001	86275000	0.000601088	*EIF2B2*
8	61275001	61325000	0.000601046	*ZCCHC7*
1	71675001	71725000	0.000600681	*DLG1*
18	35100001	35150000	0.000598983	*RIPOR1*
18	35100001	35150000	0.000598983	*CTCF*
10	58775001	58825000	0.000597997	*DMXL2*
10	33850001	33900000	0.000597383	*SPRED1*
10	33850001	33900000	0.000597383	*SNORA70*
18	35650001	35700000	0.000596451	*SLC7A6*
18	35650001	35700000	0.000596451	*PRMT7*
18	35650001	35700000	0.000596451	*SLC7A6OS*
17	58825001	58875000	0.00059637	*MED13L*
18	35125001	35175000	0.000596126	*CTCF*
18	35125001	35175000	0.000596126	*RIPOR1*
9	39450001	39500000	0.00059569	*AMD1*
9	39450001	39500000	0.00059569	*CDK19*
25	7675001	7725000	0.000595529	*CARHSP1*
25	7675001	7725000	0.000595529	*PMM2*
25	7675001	7725000	0.000595529	*USP7*
24	48625001	48675000	0.000595358	*SMAD7*
10	58675001	58725000	0.000595014	*SCG3*
10	86325001	86375000	0.000594464	*NEK9*
10	86325001	86375000	0.000594464	*ZC2HC1C*

10	86325001	86375000	0.000594464	*TMED10*
19	43225001	43275000	0.000594256	*U2*
19	43225001	43275000	0.000594256	*U2*
19	43225001	43275000	0.000594256	*U2*
19	43225001	43275000	0.000594256	*U2*
19	43225001	43275000	0.000594256	*U2*
19	43225001	43275000	0.000594256	*U2*
19	43225001	43275000	0.000594256	*U2*
11	18925001	18975000	0.000593893	*CRIM1*
3	103900001	103950000	0.00059377	*FOXJ3*
1	107075001	107125000	0.00059352	*IFT80*
1	107075001	107125000	0.00059352	*SMC4*
9	33500001	33550000	0.000592414	*VGLL2*
4	94325001	94375000	0.000590641	*COPG2*
3	109850001	109900000	0.000588862	*AGO3*
3	109850001	109900000	0.000588862	*AGO1*
16	12100001	12150000	0.000588698	*CDC73*
16	12100001	12150000	0.000588698	*GLRX2*
9	87150001	87200000	0.00058838	*ULBP17*
1	70000001	70050000	0.000587169	*OSBPL11*
18	45500001	45550000	0.000585333	*ZNF181*
18	45500001	45550000	0.000585333	*ZNF599*
18	35950001	36000000	0.000585249	*CDH3*
13	49125001	49175000	0.00058408	*BMP2*
17	56975001	57025000	0.000582266	*TAOK3*
16	39825001	39875000	0.000581651	*DNM3*
5	59000001	59050000	0.000581205	*OR6C266*
5	59000001	59050000	0.000581205	*OR6C74*
9	38950001	39000000	0.000580745	*REV3L*
24	48825001	48875000	0.00058066	*DYM*
10	59325001	59375000	0.000579726	*TNFAIP8L3*
1	110925001	110975000	0.000579686	*TIPARP*
1	126300001	126350000	0.000577216	*PLS1*
1	126300001	126350000	0.000577216	*TRPC1*
2	90900001	90950000	0.000576818	*BMPR2*
12	35825001	35875000	0.000576582	*IL17D*
12	35825001	35875000	0.000576582	*IFT88*
7	51675001	51725000	0.000576007	*EIF4EBP3*
7	51675001	51725000	0.000576007	*SRA1*
9	39475001	39525000	0.000575034	*CDK19*
9	39475001	39525000	0.000575034	*AMD1*
1	71600001	71650000	0.0005744	*DLG1*
11	94300001	94350000	0.000573784	*STRBP*
18	35425001	35475000	0.00057361	*SLC12A4*
18	35425001	35475000	0.00057361	*DPEP2*
18	35425001	35475000	0.00057361	*DPEP3*
18	35425001	35475000	0.00057361	*LCAT*
25	7700001	7750000	0.000573051	*USP7*
8	61250001	61300000	0.000571716	*ZCCHC7*
1	71525001	71575000	0.00057159	*DLG1*
10	59450001	59500000	0.000571419	*AP4E1*
28	13800001	13850000	0.00057096	*HNRNPF*

18	35475001	35525000	0.000570638	*DUS2*
18	35475001	35525000	0.000570638	*NFATC3*
18	35475001	35525000	0.000570638	*DDX28*
5	34400001	34450000	0.000569157	*ARID2*
3	21425001	21475000	0.000568337	*RBM8A*
3	21425001	21475000	0.000568337	*ITGA10*
3	21425001	21475000	0.000568337	*PEX11B*
3	21425001	21475000	0.000568337	*GNRHR2*
10	59075001	59125000	0.000567616	*CYP19A1*
9	38925001	38975000	0.000567008	*REV3L*
18	35175001	35225000	0.000566334	*CTCF*
18	35175001	35225000	0.000566334	*CARMIL2*
18	35175001	35225000	0.000566334	*ACD*
18	35175001	35225000	0.000566334	*ENKD1*
18	35175001	35225000	0.000566334	*PARD6A*
18	35175001	35225000	0.000566334	*C18H16orf86*
18	35175001	35225000	0.000566334	*GFOD2*
10	25725001	25775000	0.000565269	*CHD8*
10	25725001	25775000	0.000565269	*SUPT16H*
10	25725001	25775000	0.000565269	*SNORD8*
10	25725001	25775000	0.000565269	*SNORD9*
2	90775001	90825000	0.000564505	*NOP58*
2	90775001	90825000	0.000564505	*SNORD70*
2	90775001	90825000	0.000564505	*SNORD70B*
2	90775001	90825000	0.000564505	*SNORD11B*
19	31250001	31300000	0.000563714	*ARHGAP44*
9	96200001	96250000	0.000563694	*IGF2R*
18	34875001	34925000	0.000563497	*SLC9A5*
18	34875001	34925000	0.000563497	*KCTD19*
18	34875001	34925000	0.000563497	*PLEKHG4*
18	21000001	21050000	0.000562352	*TOX3*
24	48850001	48900000	0.000559775	*DYM*
3	21450001	21500000	0.000559649	*ITGA10*
3	21450001	21500000	0.000559649	*ANKRD35*
3	21450001	21500000	0.000559649	*PEX11B*
3	21450001	21500000	0.000559649	*GNRHR2*
16	39850001	39900000	0.000558755	*DNM3*
16	39775001	39825000	0.000558694	*DNM3*
10	59700001	59750000	0.000555775	*TRPM7*
11	97375001	97425000	0.000554827	*LMX1B*
18	21675001	21725000	0.000554378	*CHD9*
10	58325001	58375000	0.000554274	*MAPK6*
1	136875001	136925000	0.000553512	*UBA5*
1	136875001	136925000	0.000553512	*ACAD11*
26	14975001	15025000	0.000553347	*FRA10AC1*
1	71625001	71675000	0.000547212	*DLG1*
9	41525001	41575000	0.000546554	*FOXO3*
17	13175001	13225000	0.000546499	*ANAPC10*
17	13175001	13225000	0.000546499	*ABCE1*
17	13175001	13225000	0.000546499	*U6*
18	21625001	21675000	0.000545714	*CHD9*
5	30750001	30800000	0.000544157	*KMT2D*

5	30750001	30800000	0.000544157	*RHEBL1*
5	30750001	30800000	0.000544157	*PRKAG1*
18	21700001	21750000	0.000543848	*CHD9*
5	46475001	46525000	0.000542499	*CAND1*
3	9050001	9100000	0.000542392	*CD84*
18	35400001	35450000	0.00054143	*SLC12A4*
18	35400001	35450000	0.00054143	*PSKH1*
18	35400001	35450000	0.00054143	*PSMB10*
18	35400001	35450000	0.00054143	*LCAT*
18	44675001	44725000	0.000538952	*GARRE1*
10	86050001	86100000	0.000538756	*YLPM1*
12	36175001	36225000	0.000537593	*ZMYM2*
3	32125001	32175000	0.00053753	*CHI3L2*
10	86275001	86325000	0.000535058	*MLH3*
10	86275001	86325000	0.000535058	*ACYP1*
10	86275001	86325000	0.000535058	*ZC2HC1C*
18	34900001	34950000	0.000534998	*KCTD19*
18	34900001	34950000	0.000534998	*LRRC36*
18	34900001	34950000	0.000534998	*PLEKHG4*
26	15200001	15250000	0.000534741	*LGI1*
12	36100001	36150000	0.000534094	*ZMYM2*
12	36100001	36150000	0.000534094	*GJA3*
1	111250001	111300000	0.000533763	*KCNAB1*
1	71650001	71700000	0.000532309	*DLG1*
3	32150001	32200000	0.000531782	*CHI3L2*
12	35875001	35925000	0.000531719	*IFT88*
1	71550001	71600000	0.000531292	*DLG1*
17	29425001	29475000	0.000531145	*PGRMC2*
18	35800001	35850000	0.000531145	*SMPD3*
12	35850001	35900000	0.000530514	*IFT88*
16	36750001	36800000	0.00052922	*ATP1B1*
16	36750001	36800000	0.00052922	*NME7*
16	36750001	36800000	0.00052922	*SNORA66*
12	36150001	36200000	0.000528653	*ZMYM2*
5	26000001	26050000	0.000528596	*HOXC6*
5	26000001	26050000	0.000528596	*HOXC10*
5	26000001	26050000	0.000528596	*HOXC8*
5	26000001	26050000	0.000528596	*HOXC9*
5	26000001	26050000	0.000528596	*HOXC5*
5	26000001	26050000	0.000528596	*MIR196A2*
8	50850001	50900000	0.00052859	*TRPM6*
10	86250001	86300000	0.000527238	*MLH3*
10	86250001	86300000	0.000527238	*EIF2B2*
4	114225001	114275000	0.00052522	*PRKAG2*
3	9400001	9450000	0.000523488	*DCAF8*
3	9400001	9450000	0.000523488	*PEX19*
18	21975001	22025000	0.000523153	*RPGRIP1L*
23	16550001	16600000	0.000522861	*CNPY3*
23	16550001	16600000	0.000522861	*GNMT*
23	16550001	16600000	0.000522861	*PTCRA*
10	60000001	60050000	0.000521735	*SLC27A2*
10	60000001	60050000	0.000521735	*HDC*

5	25975001	26025000	0.000521518	*HOXC6*
5	25975001	26025000	0.000521518	*HOXC8*
5	25975001	26025000	0.000521518	*HOXC4*
5	25975001	26025000	0.000521518	*HOXC5*
18	44775001	44825000	0.000521105	*GPI*
18	44775001	44825000	0.000521105	*PDCD2L*
18	44775001	44825000	0.000521105	*GARRE1*
18	34850001	34900000	0.000520862	*SLC9A5*
18	34850001	34900000	0.000520862	*FHOD1*
18	34850001	34900000	0.000520862	*TMEM208*
18	35350001	35400000	0.000520792	*NUTF2*
18	35350001	35400000	0.000520792	*PSKH1*
18	35350001	35400000	0.000520792	*EDC4*
18	35350001	35400000	0.000520792	*THAP11*
18	35350001	35400000	0.000520792	*NRN1L*
10	25750001	25800000	0.000520571	*SUPT16H*
10	25750001	25800000	0.000520571	*CHD8*
10	25750001	25800000	0.000520571	*SNORD8*
10	25750001	25800000	0.000520571	*SNORD9*
18	21650001	21700000	0.000518695	*CHD9*
3	8875001	8925000	0.000518592	*CD48*
2	90850001	90900000	0.000517638	*BMPR2*
9	74250001	74300000	0.000517125	*BCLAF1*
9	74250001	74300000	0.000517125	*MTFR2*
16	39750001	39800000	0.000515988	*DNM3*
1	82600001	82650000	0.000515441	*EPHB3*
1	71575001	71625000	0.000514672	*DLG1*
18	34775001	34825000	0.000514197	*PHAF1*
18	34775001	34825000	0.000514197	*KIAA0895L*
18	34775001	34825000	0.000514197	*TRADD*
18	34775001	34825000	0.000514197	*HSF4*
18	34775001	34825000	0.000514197	*EXOC3L1*
18	34775001	34825000	0.000514197	*NOL3*
18	34775001	34825000	0.000514197	*FBXL8*
18	34775001	34825000	0.000514197	*B3GNT9*
22	23750001	23800000	0.000512875	*CNTN4*
16	12125001	12175000	0.00050992	*GLRX2*
16	12125001	12175000	0.00050992	*RO60*
4	94300001	94350000	0.000508359	*COPG2*
1	158050001	158100000	0.000507636	*ZSCAN23*
1	158050001	158100000	0.000507636	*OR2B28*
2	90800001	90850000	0.000507275	*NOP58*
2	90800001	90850000	0.000507275	*SNORD11B*
3	8700001	8750000	0.000506624	*CD244*
10	86300001	86350000	0.000503936	*NEK9*
10	86300001	86350000	0.000503936	*ZC2HC1C*
10	86300001	86350000	0.000503936	*ACYP1*
10	86300001	86350000	0.000503936	*MLH3*
16	60400001	60450000	0.000502566	*TOR3A*
16	60400001	60450000	0.000502566	*FAM20B*
18	21575001	21625000	0.000502332	*CHD9*
3	9325001	9375000	0.000501758	*NCSTN*

3	9325001	9375000	0.000501758	*COPA*
18	21725001	21775000	0.000500465	*CHD9*
11	99325001	99375000	0.000500441	*KYAT1*
10	86000001	86050000	0.00050028	*YLPM1*
24	48875001	48925000	0.000500238	*DYM*
2	121325001	121375000	0.000499072	*U6*
10	59100001	59150000	0.000496712	*CYP19A1*
2	91450001	91500000	0.000492304	*NBEAL1*
2	91450001	91500000	0.000492304	*CYP20A1*
22	10875001	10925000	0.000490192	*ITGA9*
22	10875001	10925000	0.000490192	*GOLGA4*
2	90825001	90875000	0.000489305	*BMPR2*
16	60350001	60400000	0.000488335	*FAM20B*
18	34975001	35025000	0.000488147	*LRRC36*
18	34975001	35025000	0.000488147	*ZDHHC1*
18	34975001	35025000	0.000488147	*TPPP3*
23	575001	625000	0.000484892	*KHDRBS2*
7	16625001	16675000	0.000481765	*EVI5L*
7	16625001	16675000	0.000481765	*MAP2K7*
7	16625001	16675000	0.000481765	*LRRC8E*
7	16625001	16675000	0.000481765	*PRR36*
7	16625001	16675000	0.000481765	*SNAPC2*
9	39500001	39550000	0.000481713	*CDK19*
18	45475001	45525000	0.000479689	*ZNF181*
18	45475001	45525000	0.000479689	*ZNF599*
29	8500001	8550000	0.000479029	*TMEM135*
19	48750001	48800000	0.000478776	*SMURF2*
9	39175001	39225000	0.000478206	*SLC16A10*
6	72150001	72200000	0.00047806	*RESTB*
15	49075001	49125000	0.000477463	*OR52S22*
6	72175001	72225000	0.000476641	*RESTB*
6	72175001	72225000	0.000476641	*NOA1A*
1	110900001	110950000	0.000476449	*TIPARP*
15	47225001	47275000	0.000475619	*OR52N2H*
15	47225001	47275000	0.000475619	*OR52E18*
9	39150001	39200000	0.000475012	*SLC16A10*
26	51975001	52025000	0.000474696	*PWWP2B*
18	44750001	44800000	0.000473942	*GARRE1*
18	44750001	44800000	0.000473942	*GPI*
13	69700001	69750000	0.000472835	*TOP1*
13	69700001	69750000	0.000472835	*U6*
20	38100001	38150000	0.000472052	*LMBRD2*
23	16575001	16625000	0.000470214	*PEX6*
23	16575001	16625000	0.000470214	*GNMT*
20	38050001	38100000	0.000469854	*SKP2*
10	37775001	37825000	0.000469115	*ZNF106*
12	36125001	36175000	0.000468844	*ZMYM2*
25	7750001	7800000	0.000466393	*USP7*
2	119400001	119450000	0.000460195	*PDE6D*
2	119400001	119450000	0.000460195	*PTMA*
9	39225001	39275000	0.00045884	*SLC16A10*
24	48800001	48850000	0.000457526	*DYM*

2	90875001	90925000	0.000455672	*BMPR2*
18	34800001	34850000	0.000454788	*KIAA0895L*
18	34800001	34850000	0.000454788	*E2F4*
18	34800001	34850000	0.000454788	*EXOC3L1*
18	34800001	34850000	0.000454788	*HSF4*
18	34800001	34850000	0.000454788	*ELMO3*
18	34800001	34850000	0.000454788	*NOL3*
18	34800001	34850000	0.000454788	*FBXL8*
24	48450001	48500000	0.000454593	*CTIF*
24	48450001	48500000	0.000454593	*U6*
2	91400001	91450000	0.000453214	*NBEAL1*
5	34475001	34525000	0.0004529	*ARID2*
3	32175001	32225000	0.000452649	*DENND2D*
3	32175001	32225000	0.000452649	*CHI3L2*
18	34825001	34875000	0.00045196	*FHOD1*
18	34825001	34875000	0.00045196	*E2F4*
18	34825001	34875000	0.00045196	*ELMO3*
18	34825001	34875000	0.00045196	*TMEM208*
18	34825001	34875000	0.00045196	*EXOC3L1*
2	91375001	91425000	0.000451438	*NBEAL1*
3	119325001	119375000	0.000449058	*OR9S44P*
3	119325001	119375000	0.000449058	*OR9S36B*
19	36875001	36925000	0.000449008	*FAM117A*
19	36875001	36925000	0.000449008	*SLC35B1*
19	36875001	36925000	0.000449008	*SNORA70*
10	58500001	58550000	0.000448187	*TMOD3*
10	37125001	37175000	0.000447531	*MGA*
10	37125001	37175000	0.000447531	*MAPKBP1*
18	21600001	21650000	0.000446747	*CHD9*
10	37750001	37800000	0.000446704	*ZNF106*
10	37750001	37800000	0.000446704	*CAPN3*
1	82575001	82625000	0.00044586	*EPHB3*
10	59950001	60000000	0.000443743	*GABPB1*
10	59950001	60000000	0.000443743	*HDC*
19	36725001	36775000	0.000442877	*U6*
25	7725001	7775000	0.000436497	*USP7*
18	35325001	35375000	0.000435847	*NUTF2*
18	35325001	35375000	0.000435847	*TSNAXIP1*
18	35325001	35375000	0.000435847	*CENPT*
18	35325001	35375000	0.000435847	*EDC4*
18	35325001	35375000	0.000435847	*THAP11*
18	35325001	35375000	0.000435847	*RANBP10*
3	9275001	9325000	0.000435161	*VANGL2*
3	9275001	9325000	0.000435161	*NCSTN*
9	39525001	39575000	0.0004348	*CDK19*
5	34425001	34475000	0.000433954	*ARID2*
9	39200001	39250000	0.000433469	*SLC16A10*
18	34925001	34975000	0.000433386	*LRRC36*
18	34925001	34975000	0.000433386	*KCTD19*
3	9250001	9300000	0.000431081	*VANGL2*
9	40050001	40100000	0.000431019	*WASF1*
11	99300001	99350000	0.000430122	*KYAT1*

11	99300001	99350000	0.000430122	*SPOUT1*
11	99300001	99350000	0.000430122	*ENDOG*
10	58475001	58525000	0.000429961	*TMOD3*
17	13200001	13250000	0.000428691	*ANAPC10*
18	34950001	35000000	0.000424591	*LRRC36*
18	34950001	35000000	0.000424591	*TPPP3*
1	111225001	111275000	0.000423913	*KCNAB1*
9	40025001	40075000	0.000423724	*WASF1*
9	40025001	40075000	0.000423724	*CDC40*
20	38075001	38125000	0.000423256	*LMBRD2*
20	38075001	38125000	0.000423256	*SKP2*
2	91425001	91475000	0.000420233	*NBEAL1*
13	69675001	69725000	0.000418987	*TOP1*
13	69675001	69725000	0.000418987	*U6*
10	59975001	60025000	0.000416576	*HDC*
10	59975001	60025000	0.000416576	*SLC27A2*
19	27525001	27575000	0.000414216	*KDM6B*
19	27525001	27575000	0.000414216	*CHD3*
19	27525001	27575000	0.000414216	*CYB5D1*
19	27525001	27575000	0.000414216	*NAA38*
19	27525001	27575000	0.000414216	*TMEM88*
3	9450001	9500000	0.000413408	*DCAF8*
3	9450001	9500000	0.000413408	*ATP1A4*
3	9450001	9500000	0.000413408	*PEA15*
3	9450001	9500000	0.000413408	*CASQ1*
9	40075001	40125000	0.000412689	*WASF1*
27	6950001	7000000	0.0004102	*DEFB*
9	40000001	40050000	0.000408648	*CDC40*
9	40000001	40050000	0.000408648	*WASF1*
27	6925001	6975000	0.000408447	*DEFB*
5	46500001	46550000	0.000404658	*CAND1*
13	69750001	69800000	0.000403998	*TOP1*
13	69750001	69800000	0.000403998	*PLCG1*
13	69750001	69800000	0.000403998	*U6*
13	69750001	69800000	0.000403998	*U6*
21	59000001	59050000	0.000402559	*SERPINA6*
3	101000001	101050000	0.000400574	*UROD*
3	101000001	101050000	0.000400574	*ZSWIM5*
3	101000001	101050000	0.000400574	*HECTD3*
9	104100001	104150000	0.000399603	*TBP*
9	104100001	104150000	0.000399603	*PDCD2*
1	111200001	111250000	0.000398775	*KCNAB1*
3	8850001	8900000	0.000397763	*SLAMF7*
16	60375001	60425000	0.000396875	*FAM20B*
16	60375001	60425000	0.000396875	*TOR3A*
3	9300001	9350000	0.000396366	*NCSTN*
18	44725001	44775000	0.000395122	*GARRE1*
5	34450001	34500000	0.000386809	*ARID2*
3	9425001	9475000	0.000374591	*DCAF8*
3	9425001	9475000	0.000374591	*PEA15*
3	9425001	9475000	0.000374591	*CASQ1*
3	8900001	8950000	0.00037228	*CD48*

10	18725001	18775000	0.000371714	*MYO9A*
16	38275001	38325000	0.000367781	*PRRX1*
9	39250001	39300000	0.000365838	*SLC16A10*
7	150001	200000	0.000364547	*U6*
10	10025001	10075000	0.000364209	*DMGDH*
13	69725001	69775000	0.00036408	*TOP1*
13	69725001	69775000	0.00036408	*U6*
19	36850001	36900000	0.000358141	*FAM117A*
3	8750001	8800000	0.000356028	*LY9*
3	8750001	8800000	0.000356028	*CD244*
1	157950001	158000000	0.000353215	*GPX5*
19	48800001	48850000	0.000350787	*SMURF2*
22	10750001	10800000	0.000348056	*GOLGA4*
22	10750001	10800000	0.000348056	*DCLK3*
28	2400001	2450000	0.000344189	*PGBD5*
11	82975001	83025000	0.000341229	*NBAS*
11	82975001	83025000	0.000341229	*PGGHG*
17	13225001	13275000	0.000341017	*ANAPC10*
9	39375001	39425000	0.00034093	*AMD1*
15	79100001	79150000	0.000339414	*OR8K67*
15	79100001	79150000	0.000339414	*OR8J12*
15	79100001	79150000	0.000339414	*OR8J2E*
3	8775001	8825000	0.000336831	*LY9*
11	95125001	95175000	0.000333892	*LHX2*
10	58700001	58750000	0.000333638	*DMXL2*
21	58975001	59025000	0.000331405	*SERPINA6*
10	86025001	86075000	0.000330109	*YLPM1*
26	38250001	38300000	0.000328437	*RAB11FIP2*
7	125001	175000	0.000328373	*U6*
19	36675001	36725000	0.000324019	*DLX4*
28	325001	375000	0.00032296	*OR5AS1*
8	111975001	112025000	0.000321693	*MYT1L*
3	8725001	8775000	0.00032053	*CD244*
3	8725001	8775000	0.00032053	*LY9*
1	77525001	77575000	0.000316682	*TP63*
3	32200001	32250000	0.000312201	*CEPT1*
3	32200001	32250000	0.000312201	*DENND2D*
18	44700001	44750000	0.000311036	*GARRE1*
3	8825001	8875000	0.000306512	*SLAMF7*
21	58950001	59000000	0.000305528	*SERPINA6*
11	95100001	95150000	0.000301779	*LHX2*
9	39425001	39475000	0.000295816	*AMD1*
9	39400001	39450000	0.000289832	*AMD1*
23	600001	650000	0.000283934	*KHDRBS2*
26	38275001	38325000	0.00028203	*RAB11FIP2*
3	8800001	8850000	0.000281312	*SLAMF7*
1	158025001	158075000	0.000280042	*OR2B28*
8	50825001	50875000	0.000278393	*TRPM6*
17	13250001	13300000	0.000277528	*ANAPC10*
7	110250001	110300000	0.000269982	*CAMK4*
3	8575001	8625000	0.00026979	*ITLN2*
10	10000001	10050000	0.00026047	*DMGDH*

10	10000001	10050000	0.00026047	*ARSB*
10	18950001	19000000	0.000257888	*MYO9A*
3	32225001	32275000	0.000253615	*CEPT1*
3	32225001	32275000	0.000253615	*DRAM2*
15	79425001	79475000	0.000250889	*OR5T2*
20	51200001	51250000	0.000248628	*CDH12*
10	18750001	18800000	0.000241565	*MYO9A*
22	10850001	10900000	0.000240761	*GOLGA4*
19	36700001	36750000	0.000237273	*U6*
26	38300001	38350000	0.000230021	*RAB11FIP2*
1	157975001	158025000	0.000225202	*GPX5*
15	82475001	82525000	0.000224609	*OR5AN19P*
15	82475001	82525000	0.000224609	*OR5AN2*
5	58850001	58900000	0.000216871	*OR6C1N*
5	58850001	58900000	0.000216871	*OR6C5*
9	39350001	39400000	0.000216723	*RPF2*
19	43250001	43300000	0.000213998	*U2*
19	43250001	43300000	0.000213998	*U2*
19	43250001	43300000	0.000213998	*U2*
19	43250001	43300000	0.000213998	*U2*
19	43250001	43300000	0.000213998	*U2*
19	43250001	43300000	0.000213998	*U2*
3	9000001	9050000	0.000200594	*SLAMF1*
9	39275001	39325000	0.000193067	*SLC16A10*
9	39275001	39325000	0.000193067	*U6*
5	58025001	58075000	0.000186745	*OR6C202*
8	4450001	4500000	0.00018664	*GALNTL6*
3	8925001	8975000	0.000182792	*CD48*
3	8925001	8975000	0.000182792	*SLAMF1*
3	8925001	8975000	0.000182792	*U6*
12	86925001	86975000	0.000180658	*RASA3*
29	50975001	51025000	0.000178734	*IFITM3*
14	82350001	82400000	0.000175497	*SLC2A5*
14	82325001	82375000	0.000175497	*SLC2A5*
15	79400001	79450000	0.000173721	*OR8K66*
10	18900001	18950000	0.000164588	*MYO9A*
14	50001	100000	0.000160423	*OR5D70P*
14	50001	100000	0.000160423	*U6*
3	8950001	9000000	0.000154123	*SLAMF1*
3	8950001	9000000	0.000154123	*U6*
7	42900001	42950000	0.000150444	*OR2AZ3B*
7	42900001	42950000	0.000150444	*OR2AZ1*
15	79450001	79500000	0.000149113	*OR8K63*
15	79450001	79500000	0.000149113	*OR9G4*
15	79450001	79500000	0.000149113	*OR5T2*
9	87125001	87175000	0.000148409	*ULBP17*
6	75001	125000	0.00014592	*APELA*
9	39300001	39350000	0.000141099	*RPF2*
9	39300001	39350000	0.000141099	*U6*
15	79475001	79525000	0.000139471	*OR8K63*
15	79475001	79525000	0.000139471	*OR9G4*
3	8975001	9025000	0.000130699	*SLAMF1*

10	18925001	18975000	0.000122516	*MYO9A*
9	39325001	39375000	0.000106998	*RPF2*
15	79500001	79550000	0.000106131	*OR8K63*
1	158175001	158225000	0.0000963	*TXLNA*
22	10825001	10875000	0.0000934	*GOLGA4*
22	10775001	10825000	0.0000925	*GOLGA4*
22	10800001	10850000	0.0000886	*GOLGA4*
15	79375001	79425000	0.0000803	*OR8K65P*
15	79375001	79425000	0.0000803	*OR8K66*
10	18850001	18900000	0.0000802	*MYO9A*
5	59075001	59125000	0.0000771	*OR6C7F*
5	59100001	59150000	0.0000771	*OR6C7F*
5	59075001	59125000	0.0000771	*OR6C33*
15	79350001	79400000	0.0000722	*OR8K62*
15	79350001	79400000	0.0000722	*OR8K65P*
10	93775001	93825000	0.0000708	*WDR36*
17	69650001	69700000	0.0000509	*OSBP2*
15	79325001	79375000	0.0000502	*OR8K62*
15	79325001	79375000	0.0000502	*OR5AL8*
10	18875001	18925000	0.0000469	*MYO9A*
10	93800001	93850000	0.0000441	*WDR36*
23	25600001	25650000	0.0000428	*BOLA-DQB*
23	25600001	25650000	0.0000428	*BOLA-DQA5*
15	82425001	82475000	0.0000283	*OR5AN1L*
6	25001	75000	0.0000266	*U6*
14	25001	75000	0.0000259	*OR5D70P*
14	25001	75000	0.0000259	*U6*
28	16525001	16575000	0.0000225	*CDK1*
5	58650001	58700000	0.0000211	*OR6C7G*
5	58675001	58725000	0.0000211	*OR6C7G*
29	20250001	20300000	0.0000203	*LUZP2*
15	79300001	79350000	0.0000197	*OR5AL8*
19	48775001	48825000	0.0000188	*SMURF2*
15	79250001	79300000	0.00000812	*OR8J3F*
15	79250001	79300000	0.00000812	*OR8J16*
15	79250001	79300000	0.00000812	*OR8J19*
15	82450001	82500000	0.00000719	*OR5AN19P*
5	59150001	59200000	0.00000533	*OR6C69H*
5	59150001	59200000	0.00000533	*OR6C74D*
