# Supplementary material for: Identification of genomic diversity and selection signatures in Luxi cattle using whole-genome sequencing data
Source: Anim Biosci. 2024 Jan 20;37(3):461–70. doi: 10.5713/ab.23.0304 (PMC10915192; doi:10.5713/ab.23.0304)
Supplement: Supplementary file 6 [file ab-23-0304-Supplementary-Table-S6.pdf]

Supplementary Table S6. A summary of genes from *F*st in LUX.

Chromosome	Starting position	Ending position	*F*st	Gene
28	25350001	25400000	0.660119	*U6*
1	8925001	8975000	0.622037	*H4C3*
29	49025001	49075000	0.621121	*KCNQ1*
16	41150001	41200000	0.608319	*DHRS3*
16	41175001	41225000	0.608115	*VPS13D*
16	41175001	41225000	0.608115	*SNORA59A*
29	48825001	48875000	0.605938	*KCNQ1*
16	40800001	40850000	0.605393	*TNFSF4*
16	41200001	41250000	0.600382	*VPS13D*
16	41200001	41250000	0.600382	*SNORA59A*
16	40775001	40825000	0.597396	*TNFSF4*
16	65025001	65075000	0.597019	*COLGALT2*
19	50925001	50975000	0.596358	*NOTUM*
19	50925001	50975000	0.596358	*MAFG*
19	50925001	50975000	0.596358	*PYCR1*
19	50925001	50975000	0.596358	*MYADML2*
19	50950001	51000000	0.594159	*PCYT2*
19	50950001	51000000	0.594159	*SIRT7*
19	50950001	51000000	0.594159	*MAFG*
19	50950001	51000000	0.594159	*ANAPC11*
19	50950001	51000000	0.594159	*PYCR1*
19	50950001	51000000	0.594159	*MYADML2*
19	50950001	51000000	0.594159	*NPB*
19	50950001	51000000	0.594159	*ALYREF*
1	2150001	2200000	0.592622	*IFNAR1*
8	89725001	89775000	0.592401	*SPIN1*
19	50975001	51025000	0.59128	*PCYT2*
19	50975001	51025000	0.59128	*SIRT7*
19	50975001	51025000	0.59128	*ANAPC11*
19	50975001	51025000	0.59128	*ALYREF*
19	50975001	51025000	0.59128	*ARHGDIA*
19	50975001	51025000	0.59128	*NPB*
29	49000001	49050000	0.587503	*KCNQ1*
22	51025001	51075000	0.58735	*SLC25A20*
22	51025001	51075000	0.58735	*ARIH2*
22	50050001	50100000	0.581334	*IFRD2*
22	50050001	50100000	0.581334	*SEMA3B*
22	50050001	50100000	0.581334	*LSMEM2*
22	50050001	50100000	0.581334	*GNAI2*
16	41425001	41475000	0.580941	*VPS13D*
16	41400001	41450000	0.578198	*VPS13D*
16	41375001	41425000	0.576027	*VPS13D*
29	48850001	48900000	0.575118	*KCNQ1*
1	8900001	8950000	0.574349	*H4C3*
16	41275001	41325000	0.573166	*VPS13D*
22	50950001	51000000	0.572441	*P4HTM*
22	50950001	51000000	0.572441	*QRICH1*
22	50950001	51000000	0.572441	*WDR6*
22	50950001	51000000	0.572441	*IMPDH2*
22	50950001	51000000	0.572441	*DALRD3*

22	50950001	51000000	0.572441	*NDUFAF3*
22	50950001	51000000	0.572441	*MIR191*
19	51000001	51050000	0.570748	*P4HB*
19	51000001	51050000	0.570748	*ALYREF*
19	51000001	51050000	0.570748	*ARHGDIA*
19	51000001	51050000	0.570748	*PPP1R27*
19	51000001	51050000	0.570748	*ANAPC11*
22	50975001	51025000	0.570264	*ARIH2*
22	50975001	51025000	0.570264	*P4HTM*
22	50975001	51025000	0.570264	*WDR6*
22	50975001	51025000	0.570264	*DALRD3*
16	40825001	40875000	0.569441	*TNFSF4*
22	51050001	51100000	0.568363	*SLC25A20*
22	51050001	51100000	0.568363	*PRKAR2A*
3	8975001	9025000	0.567579	*SLAMF1*
29	49050001	49100000	0.566729	*KCNQ1*
22	47925001	47975000	0.566645	*SFMBT1*
22	50075001	50125000	0.566316	*GNAI2*
22	50075001	50125000	0.566316	*SEMA3B*
8	61250001	61300000	0.566306	*ZCCHC7*
8	89700001	89750000	0.566019	*SPIN1*
16	41350001	41400000	0.565634	*VPS13D*
16	41250001	41300000	0.565425	*VPS13D*
1	1975001	2025000	0.565336	*GART*
1	1975001	2025000	0.565336	*SON*
16	41225001	41275000	0.564163	*VPS13D*
19	29650001	29700000	0.56375	*SCO1*
19	29650001	29700000	0.56375	*TMEM220*
19	29650001	29700000	0.56375	*ADPRM*
28	12800001	12850000	0.56314	*ZNF37A*
19	50900001	50950000	0.558897	*ASPSCR1*
19	50900001	50950000	0.558897	*NOTUM*
22	51000001	51050000	0.557747	*ARIH2*
22	51000001	51050000	0.557747	*SLC25A20*
8	61225001	61275000	0.556879	*ZCCHC7*
19	29600001	29650000	0.556702	*MYH3*
19	29600001	29650000	0.556702	*SCO1*
1	1950001	2000000	0.556025	*SON*
1	1950001	2000000	0.556025	*GART*
1	1950001	2000000	0.556025	*DONSON*
22	47900001	47950000	0.554712	*SFMBT1*
29	48875001	48925000	0.553574	*KCNQ1*
22	47950001	48000000	0.553322	*SFMBT1*
22	47950001	48000000	0.553322	*TMEM110*
28	28500001	28550000	0.553209	*MICU1*
5	77925001	77975000	0.552635	*RESF1*
16	41325001	41375000	0.550865	*VPS13D*
16	41075001	41125000	0.549904	*DHRS3*
16	41075001	41125000	0.549904	*AADACL4*
29	48800001	48850000	0.547706	*KCNQ1*
28	28525001	28575000	0.547092	*MICU1*
1	2300001	2350000	0.545516	*IFNAR2*

1	2300001	2350000	0.545516	*IL10RB*
22	50925001	50975000	0.543835	*QRICH1*
22	50925001	50975000	0.543835	*IMPDH2*
22	50925001	50975000	0.543835	*DALRD3*
22	50925001	50975000	0.543835	*NDUFAF3*
22	50925001	50975000	0.543835	*MIR191*
8	78900001	78950000	0.543522	*AGTPBP1*
28	28475001	28525000	0.542924	*MICU1*
28	28450001	28500000	0.537284	*MICU1*
28	12825001	12875000	0.537215	*ZNF37A*
19	52675001	52725000	0.536926	*CBX4*
22	10775001	10825000	0.536746	*GOLGA4*
1	2000001	2050000	0.536734	*GART*
1	2000001	2050000	0.536734	*TMEM50B*
1	2000001	2050000	0.536734	*DNAJC28*
16	40625001	40675000	0.536124	*TNFSF18*
19	29625001	29675000	0.53602	*SCO1*
19	29625001	29675000	0.53602	*ADPRM*
10	30400001	30450000	0.535624	*AQR*
5	58600001	58650000	0.534981	*OR6C7H*
22	50200001	50250000	0.534343	*RBM5*
22	50200001	50250000	0.534343	*RBM6*
22	50200001	50250000	0.534343	*U6*
1	149350001	149400000	0.533777	*TTC3*
22	7325001	7375000	0.53314	*GLB1*
19	52700001	52750000	0.532445	*CBX4*
19	52700001	52750000	0.532445	*CBX8*
28	25375001	25425000	0.532185	*U6*
28	28550001	28600000	0.531518	*MICU1*
1	1900001	1950000	0.529224	*CRYZL1*
1	1900001	1950000	0.529224	*ITSN1*
1	1900001	1950000	0.529224	*DONSON*
28	28400001	28450000	0.528911	*MICU1*
2	38900001	38950000	0.52859	*CYTIP*
28	28425001	28475000	0.52745	*MICU1*
28	25900001	25950000	0.527142	*FAM241B*
28	25900001	25950000	0.527142	*NEUROG3*
22	50025001	50075000	0.525419	*IFRD2*
22	50025001	50075000	0.525419	*HYAL3*
22	50025001	50075000	0.525419	*HYAL1*
22	50025001	50075000	0.525419	*LSMEM2*
22	50025001	50075000	0.525419	*HYAL2*
22	50025001	50075000	0.525419	*NAA80*
1	1925001	1975000	0.52516	*SON*
1	1925001	1975000	0.52516	*CRYZL1*
1	1925001	1975000	0.52516	*DONSON*
14	37075001	37125000	0.524614	*UBE2W*
5	103850001	103900000	0.524443	*NCAPD2*
5	103850001	103900000	0.524443	*IFFO1*
5	103850001	103900000	0.524443	*NOP2*
5	103850001	103900000	0.524443	*GAPDH*
5	103850001	103900000	0.524443	*SCARNA10*

22	48175001	48225000	0.524206	*PBRM1*
22	48175001	48225000	0.524206	*GNL3*
22	45875001	45925000	0.523756	*CACNA2D3*
14	32525001	32575000	0.523123	*C14H8orf34*
16	41125001	41175000	0.522975	*DHRS3*
8	89675001	89725000	0.522508	*SPIN1*
2	18975001	19025000	0.52208	*PDE11A*
29	48650001	48700000	0.521542	*NAP1L4*
29	48650001	48700000	0.521542	*SLC22A18*
29	48650001	48700000	0.521542	*PHLDA2*
28	25250001	25300000	0.520217	*KIFBP*
16	41300001	41350000	0.520161	*VPS13D*
29	48900001	48950000	0.519382	*KCNQ1*
22	50300001	50350000	0.519016	*RBM6*
22	50300001	50350000	0.519016	*MON1A*
8	78925001	78975000	0.518967	*AGTPBP1*
22	47875001	47925000	0.518797	*SFMBT1*
3	9000001	9050000	0.518579	*SLAMF1*
22	50275001	50325000	0.517812	*RBM6*
16	65050001	65100000	0.517766	*COLGALT2*
7	90500001	90550000	0.517566	*ADGRV1*
1	149250001	149300000	0.517483	*RIPPLY3*
1	149250001	149300000	0.517483	*U6*
8	61200001	61250000	0.517321	*ZCCHC7*
14	32550001	32600000	0.51724	*C14H8orf34*
3	98475001	98525000	0.516828	*TRABD2B*
20	20150001	20200000	0.5164	*PDE4D*
22	50100001	50150000	0.516141	*GNAI2*
22	50100001	50150000	0.516141	*SLC38A3*
8	89650001	89700000	0.515341	*SPIN1*
8	60975001	61025000	0.515312	*PAX5*
22	48200001	48250000	0.515186	*PBRM1*
22	48375001	48425000	0.515068	*NISCH*
16	41100001	41150000	0.514956	*DHRS3*
1	149400001	149450000	0.514784	*TTC3*
13	77950001	78000000	0.51424	*SLC9A8*
13	77950001	78000000	0.51424	*SPATA2*
28	28375001	28425000	0.513539	*MICU1*
28	28375001	28425000	0.513539	*DNAJB12*
22	50900001	50950000	0.512841	*QRICH1*
22	50900001	50950000	0.512841	*USP19*
22	50900001	50950000	0.512841	*QARS1*
25	34050001	34100000	0.512821	*CCL24*
29	48625001	48675000	0.512548	*NAP1L4*
19	29675001	29725000	0.512402	*TMEM220*
19	29675001	29725000	0.512402	*ADPRM*
19	51025001	51075000	0.511571	*P4HB*
19	51025001	51075000	0.511571	*GCGR*
19	51025001	51075000	0.511571	*MCRIP1*
19	51025001	51075000	0.511571	*ARHGDIA*
19	51025001	51075000	0.511571	*PPP1R27*
16	41025001	41075000	0.511489	*AADACL4*

29	48975001	49025000	0.511472	*KCNQ1*
23	15075001	15125000	0.511272	*TREM2*
23	15075001	15125000	0.511272	*TREML2*
23	15075001	15125000	0.511272	*TREML1*
24	57400001	57450000	0.511024	*NEDD4L*
8	61375001	61425000	0.509984	*ZCCHC7*
1	149375001	149425000	0.509578	*TTC3*
22	35125001	35175000	0.508854	*SLC25A26*
28	29275001	29325000	0.508819	*CFAP70*
28	29275001	29325000	0.508819	*FAM149B1*
28	29275001	29325000	0.508819	*DNAJC9*
28	29275001	29325000	0.508819	*MRPS16*
19	42550001	42600000	0.507621	*ATP6V0A1*
19	50875001	50925000	0.507352	*ASPSCR1*
19	50875001	50925000	0.507352	*LRRC45*
19	50875001	50925000	0.507352	*RAC3*
19	50875001	50925000	0.507352	*CENPX*
19	50875001	50925000	0.507352	*DCXR*
5	112375001	112425000	0.506995	*RANGAP1*
5	112375001	112425000	0.506995	*CHADL*
5	112375001	112425000	0.506995	*L3MBTL2*
5	112350001	112400000	0.506817	*L3MBTL2*
5	112350001	112400000	0.506817	*CHADL*
5	112350001	112400000	0.506817	*RANGAP1*
23	13550001	13600000	0.506316	*KIF6*
14	37100001	37150000	0.505955	*UBE2W*
8	89625001	89675000	0.505844	*SPIN1*
7	58900001	58950000	0.505619	*JAKMIP2*
20	20175001	20225000	0.505439	*PDE4D*
4	55825001	55875000	0.505288	*IFRD1*
4	55825001	55875000	0.505288	*LSMEM1*
5	112450001	112500000	0.505219	*ZC3H7B*
5	112450001	112500000	0.505219	*TEF*
29	48675001	48725000	0.50521	*SLC22A18*
29	48675001	48725000	0.50521	*NAP1L4*
29	48675001	48725000	0.50521	*PHLDA2*
22	50175001	50225000	0.504729	*SEMA3F*
22	50175001	50225000	0.504729	*RBM5*
22	10800001	10850000	0.504562	*GOLGA4*
1	149275001	149325000	0.504532	*PIGP*
1	149275001	149325000	0.504532	*TTC3*
1	149325001	149375000	0.504519	*TTC3*
28	26275001	26325000	0.504099	*MACROH2A2*
16	6425001	6475000	0.503602	*KCNT2*
21	2850001	2900000	0.503277	*ATP10A*
8	61000001	61050000	0.502843	*PAX5*
5	77950001	78000000	0.502836	*RESF1*
22	50450001	50500000	0.502521	*IP6K1*
22	50450001	50500000	0.502521	*RNF123*
22	50450001	50500000	0.502521	*GMPPB*
22	50450001	50500000	0.502521	*AMIGO3*
2	38375001	38425000	0.502503	*U6*

22	50125001	50175000	0.502491	*SLC38A3*
22	50125001	50175000	0.502491	*SEMA3F*
22	50125001	50175000	0.502491	*GNAT1*
8	86125001	86175000	0.502236	*ROR2*
19	27450001	27500000	0.501937	*DNAH2*
28	29250001	29300000	0.501631	*FAM149B1*
28	29250001	29300000	0.501631	*DNAJC9*
28	29250001	29300000	0.501631	*MRPS16*
28	29250001	29300000	0.501631	*CFAP70*
8	11550001	11600000	0.50119	*DDX58*
3	97950001	98000000	0.501185	*SLC5A9*
15	39850001	39900000	0.501158	*TEAD1*
28	29100001	29150000	0.501124	*P4HA1*
24	57425001	57475000	0.501071	*NEDD4L*
22	48975001	49025000	0.500429	*IQCF2*
22	48975001	49025000	0.500429	*RRP9*
22	48975001	49025000	0.500429	*PARP3*
22	48975001	49025000	0.500429	*IQCF1*
22	48975001	49025000	0.500429	*GPR62*
22	48975001	49025000	0.500429	*IQCF5*
22	48975001	49025000	0.500429	*PCBP4*
22	50000001	50050000	0.499428	*HYAL2*
22	50000001	50050000	0.499428	*IFRD2*
22	50000001	50050000	0.499428	*RASSF1*
22	50000001	50050000	0.499428	*HYAL3*
22	50000001	50050000	0.499428	*ZMYND10*
22	50000001	50050000	0.499428	*HYAL1*
22	50000001	50050000	0.499428	*NPRL2*
22	50000001	50050000	0.499428	*NAA80*
4	64625001	64675000	0.499328	*PDE1C*
16	45525001	45575000	0.49876	*UTS2*
8	11525001	11575000	0.498146	*DDX58*
8	11525001	11575000	0.498146	*TOPORS*
22	48225001	48275000	0.497643	*PBRM1*
22	46100001	46150000	0.49762	*CACNA2D3*
8	78875001	78925000	0.496933	*AGTPBP1*
8	89600001	89650000	0.496754	*NXNL2*
8	89600001	89650000	0.496754	*SPIN1*
1	2325001	2375000	0.496627	*IFNAR2*
16	43600001	43650000	0.496571	*CTNNBIP1*
16	43600001	43650000	0.496571	*LZIC*
16	43600001	43650000	0.496571	*NMNAT1*
28	25875001	25925000	0.496509	*NEUROG3*
26	25975001	26025000	0.495376	*SORCS3*
16	40650001	40700000	0.494966	*TNFSF18*
16	3425001	3475000	0.494965	*NUCKS1*
14	10375001	10425000	0.494911	*ASAP1*
29	48525001	48575000	0.494749	*CARS1*
29	48525001	48575000	0.494749	*U6*
8	61350001	61400000	0.49422	*ZCCHC7*
4	80600001	80650000	0.492841	*SUGCT*
24	57375001	57425000	0.49284	*NEDD4L*

5	112475001	112525000	0.492774	*TEF*
5	112475001	112525000	0.492774	*ZC3H7B*
22	35100001	35150000	0.492688	*SLC25A26*
19	27425001	27475000	0.49267	*DNAH2*
20	69300001	69350000	0.492197	*IRX1*
8	90700001	90750000	0.491042	*PLPPR1*
16	41050001	41100000	0.491024	*AADACL4*
1	2025001	2075000	0.490419	*TMEM50B*
1	2025001	2075000	0.490419	*DNAJC28*
23	13525001	13575000	0.489972	*KIF6*
18	43325001	43375000	0.489851	*CEP89*
22	48350001	48400000	0.489295	*NISCH*
22	48350001	48400000	0.489295	*STAB1*
13	77925001	77975000	0.488903	*SLC9A8*
19	29575001	29625000	0.488674	*MYH3*
8	83425001	83475000	0.488256	*ZNF782*
2	17975001	18025000	0.488059	*CCDC141*
29	49075001	49125000	0.488018	*KCNQ1*
4	64650001	64700000	0.487918	*PDE1C*
29	48550001	48600000	0.487805	*CARS1*
16	3450001	3500000	0.487305	*NUCKS1*
22	35150001	35200000	0.487055	*SLC25A26*
29	48700001	48750000	0.486576	*SLC22A18*
29	48700001	48750000	0.486576	*CDKN1C*
8	82650001	82700000	0.486541	*ERCC6L2*
16	6400001	6450000	0.486316	*KCNT2*
8	82675001	82725000	0.486295	*ERCC6L2*
19	50850001	50900000	0.485644	*RAC3*
19	50850001	50900000	0.485644	*LRRC45*
19	50850001	50900000	0.485644	*ASPSCR1*
19	50850001	50900000	0.485644	*CENPX*
19	50850001	50900000	0.485644	*DCXR*
19	50850001	50900000	0.485644	*DCXR*
16	6450001	6500000	0.48543	*KCNT2*
28	25575001	25625000	0.485396	*HKDC1*
28	25575001	25625000	0.485396	*SUPV3L1*
21	2875001	2925000	0.485356	*ATP10A*
21	2875001	2925000	0.485356	*U6*
4	12375001	12425000	0.485246	*PPP1R9A*
19	46125001	46175000	0.484919	*KANSL1*
22	48950001	49000000	0.484714	*PCBP4*
22	48950001	49000000	0.484714	*RRP9*
22	48950001	49000000	0.484714	*PARP3*
22	48950001	49000000	0.484714	*ABHD14B*
22	48950001	49000000	0.484714	*ACY1*
22	48950001	49000000	0.484714	*ABHD14A*
22	48950001	49000000	0.484714	*IQCF2*
22	48950001	49000000	0.484714	*GPR62*
22	45225001	45275000	0.484674	*ERC2*
11	100001	150000	0.484525	*ZC3H6*
16	43775001	43825000	0.48378	*PIK3CD*
16	43775001	43825000	0.48378	*CLSTN1*

16	43775001	43825000	0.48378	*U6*
10	27425001	27475000	0.483671	*OR4L1*
3	103600001	103650000	0.483476	*CCDC30*
5	47825001	47875000	0.48336	*HMGA2*
5	69600001	69650000	0.483177	*CKAP4*
22	50425001	50475000	0.482986	*IP6K1*
22	50425001	50475000	0.482986	*CDHR4*
22	50425001	50475000	0.482986	*UBA7*
22	50425001	50475000	0.482986	*INKA1*
25	18775001	18825000	0.482833	*DNAH3*
6	19025001	19075000	0.482658	*TBCK*
3	15950001	16000000	0.482556	*ADAR*
2	26625001	26675000	0.48239	*PPIG*
2	26625001	26675000	0.48239	*CFAP210*
19	26375001	26425000	0.482208	*KIF1C*
19	26375001	26425000	0.482208	*CAMTA2*
19	26375001	26425000	0.482208	*INCA1*
19	31075001	31125000	0.482116	*MYOCD*
19	42750001	42800000	0.481819	*EZH1*
19	42750001	42800000	0.481819	*CNTNAP1*
19	42750001	42800000	0.481819	*RAMP2*
16	3400001	3450000	0.481458	*SLC45A3*
2	26700001	26750000	0.481369	*FASTKD1*
2	26700001	26750000	0.481369	*KLHL41*
5	53775001	53825000	0.481036	*SLC16A7*
16	43800001	43850000	0.481025	*PIK3CD*
16	43800001	43850000	0.481025	*U6*
19	42725001	42775000	0.480981	*EZH1*
19	42725001	42775000	0.480981	*CNTNAP1*
19	42725001	42775000	0.480981	*PLEKHH3*
19	42725001	42775000	0.480981	*CCR10*
19	42725001	42775000	0.480981	*TUBG2*
16	45450001	45500000	0.480937	*TNFRSF9*
19	51225001	51275000	0.480814	*FAAP100*
19	51225001	51275000	0.480814	*FSCN2*
19	51225001	51275000	0.480814	*ACTG1*
1	152825001	152875000	0.480797	*ANKRD28*
22	7225001	7275000	0.480633	*TRIM71*
16	41500001	41550000	0.480384	*TNFRSF1B*
28	25800001	25850000	0.480073	*TSPAN15*
1	2100001	2150000	0.480041	*IFNGR2*
8	39375001	39425000	0.479899	*JAK2*
28	29125001	29175000	0.479634	*P4HA1*
8	59975001	60025000	0.479612	*TMEM8B*
8	59975001	60025000	0.479612	*FAM221B*
8	59975001	60025000	0.479612	*HINT2*
8	59975001	60025000	0.479612	*SPAG8*
28	25550001	25600000	0.47935	*SUPV3L1*
28	25550001	25600000	0.47935	*HKDC1*
28	25550001	25600000	0.47935	*VPS26A*
14	37125001	37175000	0.479081	*UBE2W*
29	48775001	48825000	0.478841	*KCNQ1*

28	28350001	28400000	0.478792	*DNAJB12*
8	86525001	86575000	0.478626	*AUH*
3	700001	750000	0.47834	*DCAF6*
22	50225001	50275000	0.478197	*RBM6*
22	50225001	50275000	0.478197	*RBM5*
22	50225001	50275000	0.478197	*U6*
19	26275001	26325000	0.478153	*SCIMP*
22	50475001	50525000	0.477928	*RNF123*
22	50475001	50525000	0.477928	*APEH*
22	50475001	50525000	0.477928	*IP6K1*
22	50475001	50525000	0.477928	*MST1*
22	50475001	50525000	0.477928	*GMPPB*
22	50475001	50525000	0.477928	*AMIGO3*
20	20200001	20250000	0.47792	*PDE4D*
14	37050001	37100000	0.477192	*STAU2*
22	50875001	50925000	0.476978	*LAMB2*
22	50875001	50925000	0.476978	*USP19*
22	50875001	50925000	0.476978	*QARS1*
22	50875001	50925000	0.476978	*QRICH1*
2	38350001	38400000	0.476932	*U6*
11	3675001	3725000	0.476588	*INPP4A*
13	17400001	17450000	0.476231	*RBM17*
4	69600001	69650000	0.476132	*SNX10*
21	2750001	2800000	0.475646	*ATP10A*
11	3700001	3750000	0.475356	*INPP4A*
7	90625001	90675000	0.475275	*ADGRV1*
15	40800001	40850000	0.475143	*USP47*
20	14725001	14775000	0.475141	*SREK1IP1*
20	14725001	14775000	0.475141	*SHISAL2B*
20	14725001	14775000	0.475141	*CWC27*
10	30375001	30425000	0.475111	*AQR*
7	90400001	90450000	0.47507	*ADGRV1*
8	61275001	61325000	0.475059	*ZCCHC7*
15	39825001	39875000	0.47499	*TEAD1*
7	58875001	58925000	0.474778	*JAKMIP2*
5	112425001	112475000	0.47471	*ZC3H7B*
5	112425001	112475000	0.47471	*U6*
5	112425001	112475000	0.47471	*RANGAP1*
19	42575001	42625000	0.474552	*ATP6V0A1*
19	42575001	42625000	0.474552	*NAGLU*
5	47800001	47850000	0.474503	*HMGA2*
22	52625001	52675000	0.474193	*MYL3*
22	52625001	52675000	0.474193	*PRSS42*
4	69625001	69675000	0.474166	*SNX10*
19	52625001	52675000	0.474003	*TBC1D16*
5	77900001	77950000	0.473977	*RESF1*
2	17950001	18000000	0.47381	*CCDC141*
29	48600001	48650000	0.473565	*NAP1L4*
29	48600001	48650000	0.473565	*CARS1*
5	112500001	112550000	0.473265	*TEF*
5	112500001	112550000	0.473265	*TOB2*
3	19650001	19700000	0.47322	*SEMA6C*

3	19650001	19700000	0.47322	*LYSMD1*
3	19650001	19700000	0.47322	*TNFAIP8L2*
3	19650001	19700000	0.47322	*GABPB2*
19	42525001	42575000	0.472844	*ATP6V0A1*
13	77975001	78025000	0.472801	*RNF114*
13	77975001	78025000	0.472801	*SPATA2*
13	77975001	78025000	0.472801	*SNORA70*
28	25275001	25325000	0.47278	*KIFBP*
8	39400001	39450000	0.472595	*JAK2*
28	28900001	28950000	0.472349	*OIT3*
28	28900001	28950000	0.472349	*PLA2G12B*
28	28900001	28950000	0.472349	*MCU*
3	675001	725000	0.47223	*DCAF6*
8	61175001	61225000	0.471965	*ZCCHC7*
8	61175001	61225000	0.471965	*PAX5*
22	50850001	50900000	0.471937	*LAMB2*
22	50850001	50900000	0.471937	*KLHDC8B*
22	50850001	50900000	0.471937	*C22H3orf84*
22	50850001	50900000	0.471937	*CCDC71*
22	50850001	50900000	0.471937	*USP19*
3	120600001	120650000	0.471894	*FARP2*
3	120600001	120650000	0.471894	*STK25*
3	120600001	120650000	0.471894	*BOK*
22	48150001	48200000	0.471465	*PBRM1*
22	48150001	48200000	0.471465	*GLT8D1*
22	48150001	48200000	0.471465	*GNL3*
22	48150001	48200000	0.471465	*SPCS1*
22	48150001	48200000	0.471465	*NEK4*
22	48150001	48200000	0.471465	*SNORD19B*
22	48150001	48200000	0.471465	*SNORD69*
22	48150001	48200000	0.471465	*SNORD19*
22	48150001	48200000	0.471465	*SNORD19C*
8	8925001	8975000	0.471252	*MSRA*
7	90375001	90425000	0.471087	*ADGRV1*
28	3650001	3700000	0.470728	*TTC13*
16	43925001	43975000	0.469584	*SLC25A33*
22	48925001	48975000	0.469258	*PCBP4*
22	48925001	48975000	0.469258	*ACY1*
22	48925001	48975000	0.469258	*ABHD14B*
22	48925001	48975000	0.469258	*ABHD14A*
22	48925001	48975000	0.469258	*RPL29*
16	2925001	2975000	0.469206	*DSTYK*
2	26650001	26700000	0.469136	*PPIG*
2	26650001	26700000	0.469136	*FASTKD1*
19	27550001	27600000	0.46898	*CHD3*
19	27550001	27600000	0.46898	*CYB5D1*
19	27550001	27600000	0.46898	*RNF227*
3	97975001	98025000	0.468978	*SLC5A9*
16	2950001	3000000	0.468916	*DSTYK*
17	6875001	6925000	0.468462	*LRBA*
13	17425001	17475000	0.468433	*IL2RA*
19	27850001	27900000	0.468258	*CTC1*

19	27850001	27900000	0.468258	*PFAS*
19	38550001	38600000	0.46815	*SP2*
19	44900001	44950000	0.468125	*MAP3K14*
1	2275001	2325000	0.468004	*IL10RB*
1	2275001	2325000	0.468004	*IFNAR2*
19	46100001	46150000	0.467755	*KANSL1*
8	8950001	9000000	0.467692	*MSRA*
22	46125001	46175000	0.467137	*CACNA2D3*
28	8200001	8250000	0.467114	*B3GALNT2*
19	46150001	46200000	0.467032	*KANSL1*
19	50725001	50775000	0.467021	*CCDC57*
5	112400001	112450000	0.466988	*RANGAP1*
5	112400001	112450000	0.466988	*ZC3H7B*
5	112400001	112450000	0.466988	*U6*
5	58050001	58100000	0.466973	*OR6C202*
5	58050001	58100000	0.466973	*OR6C207*
21	65025001	65075000	0.466554	*EVL*
8	61325001	61375000	0.466371	*ZCCHC7*
16	41600001	41650000	0.465812	*TNFRSF8*
4	80575001	80625000	0.465162	*SUGCT*
16	41575001	41625000	0.465015	*TNFRSF8*
1	2175001	2225000	0.464915	*IFNAR1*
22	50250001	50300000	0.464577	*RBM6*
18	54200001	54250000	0.464537	*SAE1*
18	54200001	54250000	0.464537	*BBC3*
16	43900001	43950000	0.46451	*SLC25A33*
16	43900001	43950000	0.46451	*TMEM201*
14	23025001	23075000	0.463786	*TMEM68*
14	23025001	23075000	0.463786	*TGS1*
5	112525001	112575000	0.46299	*TOB2*
5	112525001	112575000	0.46299	*PHF5A*
5	112525001	112575000	0.46299	*ACO2*
18	14400001	14450000	0.462985	*ANKRD11*
8	39350001	39400000	0.462896	*JAK2*
8	39350001	39400000	0.462896	*INSL6*
16	43575001	43625000	0.462873	*NMNAT1*
16	43575001	43625000	0.462873	*LZIC*
21	65050001	65100000	0.462872	*EVL*
21	65050001	65100000	0.462872	*DEGS2*
19	23000001	23050000	0.462856	*RTN4RL1*
3	97925001	97975000	0.462743	*SPATA6*
3	97925001	97975000	0.462743	*SLC5A9*
1	22175001	22225000	0.462481	*NRIP1*
4	57625001	57675000	0.462375	*IMMP2L*
22	52650001	52700000	0.46228	*PRSS42*
26	45475001	45525000	0.462231	*FANK1*
26	45475001	45525000	0.462231	*ADAM12*
19	50750001	50800000	0.462068	*CCDC57*
19	50750001	50800000	0.462068	*FASN*
11	125001	175000	0.461928	*ZC3H6*
8	89575001	89625000	0.461813	*NXNL2*
19	27475001	27525000	0.461742	*DNAH2*

15	35900001	35950000	0.461694	*SOX6*
18	54175001	54225000	0.46155	*SAE1*
14	34000001	34050000	0.4615	*NCOA2*
22	49975001	50025000	0.461448	*NPRL2*
22	49975001	50025000	0.461448	*CACNA2D2*
22	49975001	50025000	0.461448	*RASSF1*
22	49975001	50025000	0.461448	*HYAL2*
22	49975001	50025000	0.461448	*ZMYND10*
22	49975001	50025000	0.461448	*CYB561D2*
14	4475001	4525000	0.461171	*FAM135B*
22	43575001	43625000	0.461037	*ARF4*
22	43575001	43625000	0.461037	*DENND6A*
1	149225001	149275000	0.460976	*RIPPLY3*
1	149225001	149275000	0.460976	*U6*
19	51250001	51300000	0.460799	*ACTG1*
7	90475001	90525000	0.460606	*ADGRV1*
22	41075001	41125000	0.46035	*FHIT*
4	69575001	69625000	0.460214	*SNX10*
19	52750001	52800000	0.460107	*CBX2*
19	52750001	52800000	0.460107	*ENPP7*
6	66725001	66775000	0.459998	*NIPAL1*
6	66725001	66775000	0.459998	*TXK*
22	50150001	50200000	0.459993	*SEMA3F*
22	50150001	50200000	0.459993	*GNAT1*
28	25850001	25900000	0.459912	*TSPAN15*
8	60000001	60050000	0.459869	*TMEM8B*
8	60000001	60050000	0.459869	*OR13E1*
29	48950001	49000000	0.459627	*KCNQ1*
16	65000001	65050000	0.459475	*COLGALT2*
3	80750001	80800000	0.459418	*CACHD1*
18	22025001	22075000	0.459268	*FTO*
18	22025001	22075000	0.459268	*RPGRIP1L*
7	90600001	90650000	0.459142	*ADGRV1*
2	26675001	26725000	0.459123	*FASTKD1*
2	26675001	26725000	0.459123	*PPIG*
29	5250001	5300000	0.459099	*TRIM64*
22	7200001	7250000	0.459078	*TRIM71*
8	76225001	76275000	0.459068	*RASEF*
7	11850001	11900000	0.458907	*ZSWIM4*
7	11850001	11900000	0.458907	*YJU2B*
7	11850001	11900000	0.458907	*MRI1*
7	11850001	11900000	0.458907	*C7H19orf53*
18	43350001	43400000	0.458782	*CEP89*
18	43350001	43400000	0.458782	*FAAP24*
14	10400001	10450000	0.458767	*ASAP1*
3	103625001	103675000	0.458639	*CCDC30*
14	34025001	34075000	0.458473	*NCOA2*
22	45900001	45950000	0.45813	*CACNA2D3*
3	36175001	36225000	0.458098	*NTNG1*
5	103825001	103875000	0.457761	*CHD4*
5	103825001	103875000	0.457761	*IFFO1*
5	103825001	103875000	0.457761	*NOP2*

5	103825001	103875000	0.457761	*GAPDH*
5	103825001	103875000	0.457761	*SCARNA11*
22	48550001	48600000	0.457087	*GLYCTK*
28	26575001	26625000	0.456728	*EIF4EBP2*
28	26575001	26625000	0.456728	*LRRC20*
8	11500001	11550000	0.456724	*NDUFB6*
8	11500001	11550000	0.456724	*TOPORS*
3	36200001	36250000	0.456512	*NTNG1*
1	22150001	22200000	0.456294	*NRIP1*
5	103875001	103925000	0.456277	*NCAPD2*
5	103875001	103925000	0.456277	*VAMP1*
5	103875001	103925000	0.456277	*MRPL51*
5	103875001	103925000	0.456277	*TAPBPL*
5	103875001	103925000	0.456277	*SCARNA10*
2	17775001	17825000	0.456036	*CCDC141*
24	57350001	57400000	0.456005	*NEDD4L*
3	107025001	107075000	0.455737	*RHBDL2*
22	48275001	48325000	0.455689	*NT5DC2*
22	48275001	48325000	0.455689	*PBRM1*
22	48275001	48325000	0.455689	*SMIM4*
28	28675001	28725000	0.455483	*MCU*
24	57300001	57350000	0.455101	*NEDD4L*
28	25425001	25475000	0.454977	*SRGN*
4	81025001	81075000	0.454876	*CDK13*
5	36500001	36550000	0.454791	*TMEM117*
8	82150001	82200000	0.454761	*PTCH1*
14	32500001	32550000	0.45472	*C14H8orf34*
22	30450001	30500000	0.454535	*FOXP1*
22	48250001	48300000	0.45451	*PBRM1*
22	48250001	48300000	0.45451	*NT5DC2*
22	48250001	48300000	0.45451	*SMIM4*
16	43875001	43925000	0.454132	*TMEM201*
16	43875001	43925000	0.454132	*SLC25A33*
16	43875001	43925000	0.454132	*U6*
8	61725001	61775000	0.454126	*FRMPD1*
28	28325001	28375000	0.453855	*DNAJB12*
28	28325001	28375000	0.453855	*DDIT4*
29	49175001	49225000	0.453824	*CD81*
29	49175001	49225000	0.453824	*TRPM5*
29	49175001	49225000	0.453824	*TSSC4*
22	50825001	50875000	0.453768	*IHO1*
22	50825001	50875000	0.453768	*C22H3orf84*
22	50825001	50875000	0.453768	*KLHDC8B*
22	50825001	50875000	0.453768	*CCDC71*
16	43825001	43875000	0.453741	*PIK3CD*
18	54150001	54200000	0.453317	*SAE1*
19	41200001	41250000	0.453224	*KRTAP1-1*
19	41200001	41250000	0.453224	*KRTAP3-1*
8	11575001	11625000	0.452882	*DDX58*
8	11575001	11625000	0.452882	*ACO1*
18	50400001	50450000	0.452819	*AXL*
18	50400001	50450000	0.452819	*HNRNPUL1*

15	35875001	35925000	0.452517	*SOX6*
18	15675001	15725000	0.452394	*ITFG1*
22	46225001	46275000	0.452179	*CACNA2D3*
5	36475001	36525000	0.452115	*TMEM117*
29	48925001	48975000	0.451998	*KCNQ1*
11	3725001	3775000	0.451993	*INPP4A*
15	51600001	51650000	0.45197	*RNF121*
15	51600001	51650000	0.45197	*NUMA1*
15	51600001	51650000	0.45197	*IL18BP*
16	45425001	45475000	0.451942	*TNFRSF9*
13	77750001	77800000	0.451914	*B4GALT5*
15	51575001	51625000	0.451821	*RNF121*
5	78175001	78225000	0.451816	*AMN1*
16	43850001	43900000	0.451748	*TMEM201*
16	43850001	43900000	0.451748	*PIK3CD*
16	43850001	43900000	0.451748	*U6*
14	1150001	1200000	0.45172	*ZC3H3*
22	45200001	45250000	0.451672	*ERC2*
11	44950001	45000000	0.45156	*U6*
2	18950001	19000000	0.451543	*PDE11A*
7	90425001	90475000	0.451518	*ADGRV1*
4	69900001	69950000	0.451468	*MIR148A*
20	20250001	20300000	0.451356	*PDE4D*
4	81000001	81050000	0.451256	*CDK13*
14	33800001	33850000	0.451236	*NCOA2*
14	33800001	33850000	0.451236	*PRDM14*
27	19975001	20025000	0.451149	*CNOT7*
27	19975001	20025000	0.451149	*ZDHHC2*
27	19975001	20025000	0.451149	*VPS37A*
23	16650001	16700000	0.45106	*CUL7*
23	16650001	16700000	0.45106	*KLC4*
23	16650001	16700000	0.45106	*KLHDC3*
23	16650001	16700000	0.45106	*RRP36*
23	16650001	16700000	0.45106	*MEA1*
23	16650001	16700000	0.45106	*MRPL2*
23	16650001	16700000	0.45106	*PPP2R5D*
19	42500001	42550000	0.451029	*CAVIN1*
28	325001	375000	0.451007	*OR5AS1*
10	27450001	27500000	0.450957	*OR4L1*
10	27450001	27500000	0.450957	*OR4L18*
10	27450001	27500000	0.450957	*OR4N5*
16	38725001	38775000	0.450895	*FMO2*
4	57600001	57650000	0.450802	*IMMP2L*
14	34100001	34150000	0.450799	*NCOA2*
14	34100001	34150000	0.450799	*U6*
14	34100001	34150000	0.450799	*U6*
8	71525001	71575000	0.450661	*ADAM28*
19	41225001	41275000	0.450631	*KRTAP1-1*
2	38875001	38925000	0.450536	*CYTIP*
2	17425001	17475000	0.450514	*ZNF385B*
23	18900001	18950000	0.450446	*RUNX2*
8	61300001	61350000	0.450187	*ZCCHC7*

3	36025001	36075000	0.450143	*NTNG1*
3	650001	700000	0.450123	*DCAF6*
7	53800001	53850000	0.44957	*ARHGAP26*
29	48750001	48800000	0.44947	*KCNQ1*
19	51200001	51250000	0.449365	*NPLOC4*
19	51200001	51250000	0.449365	*FAAP100*
19	51200001	51250000	0.449365	*FSCN2*
23	13600001	13650000	0.449293	*KIF6*
22	49000001	49050000	0.449051	*IQCF2*
22	49000001	49050000	0.449051	*IQCF1*
22	49000001	49050000	0.449051	*IQCF5*
19	46075001	46125000	0.448985	*KANSL1*
16	51200001	51250000	0.448956	*ACAP3*
16	51200001	51250000	0.448956	*UBE2J2*
16	51200001	51250000	0.448956	*INTS11*
16	51200001	51250000	0.448956	*SCNN1D*
16	51200001	51250000	0.448956	*PUSL1*
16	51200001	51250000	0.448956	*CPTP*
19	26300001	26350000	0.448901	*ZFP3*
19	26300001	26350000	0.448901	*SCIMP*
23	15100001	15150000	0.448863	*TREM1*
23	15100001	15150000	0.448863	*TREML2*
23	15100001	15150000	0.448863	*TREM2*
19	31100001	31150000	0.448837	*MYOCD*
20	20225001	20275000	0.44864	*PDE4D*
10	30900001	30950000	0.448626	*DPH6*
19	47425001	47475000	0.448576	*TANC2*
16	45375001	45425000	0.448445	*PARK7*
22	45250001	45300000	0.44832	*ERC2*
16	2975001	3025000	0.448251	*TMCC2*
16	2975001	3025000	0.448251	*DSTYK*
19	22975001	23025000	0.448234	*RTN4RL1*
28	28575001	28625000	0.448106	*MICU1*
14	10325001	10375000	0.447962	*ASAP1*
25	34025001	34075000	0.447896	*CCL26*
25	34025001	34075000	0.447896	*CCL24*
16	3225001	3275000	0.447885	*CDK18*
20	14700001	14750000	0.447836	*CWC27*
20	14700001	14750000	0.447836	*SREK1IP1*
28	25825001	25875000	0.447813	*TSPAN15*
4	55850001	55900000	0.447754	*IFRD1*
4	55850001	55900000	0.447754	*LSMEM1*
19	52025001	52075000	0.447687	*ENDOV*
19	52025001	52075000	0.447687	*NPTX1*
3	120850001	120900000	0.447629	*RTP5*
16	6475001	6525000	0.447528	*KCNT2*
19	26350001	26400000	0.447345	*KIF1C*
19	26350001	26400000	0.447345	*ZFP3*
4	80625001	80675000	0.44732	*SUGCT*
1	1875001	1925000	0.447303	*ITSN1*
1	1875001	1925000	0.447303	*CRYZL1*
28	15675001	15725000	0.44727	*ANK3*

20	69275001	69325000	0.447152	*IRX1*
22	53575001	53625000	0.447038	*SACM1L*
28	26600001	26650000	0.446972	*EIF4EBP2*
28	26600001	26650000	0.446972	*NODAL*
18	2575001	2625000	0.44694	*BCAR1*
18	2575001	2625000	0.44694	*CFDP1*
18	11475001	11525000	0.446938	*GSE1*
28	28275001	28325000	0.44686	*ASCC1*
28	28275001	28325000	0.44686	*ANAPC16*
28	28275001	28325000	0.44686	*DDIT4*
29	49150001	49200000	0.446803	*TRPM5*
29	49150001	49200000	0.446803	*TSSC4*
29	49150001	49200000	0.446803	*CD81*
1	149475001	149525000	0.446368	*VPS26C*
14	32475001	32525000	0.446343	*C14H8orf34*
21	41975001	42025000	0.4463	*GPR33*
19	23175001	23225000	0.446202	*SMG6*
20	20125001	20175000	0.446162	*PDE4D*
19	38575001	38625000	0.445944	*SCRN2*
19	38575001	38625000	0.445944	*SP6*
22	51175001	51225000	0.445919	*IP6K2*
22	51175001	51225000	0.445919	*NCKIPSD*
22	51175001	51225000	0.445919	*CELSR3*
19	27875001	27925000	0.445544	*PFAS*
19	27875001	27925000	0.445544	*SLC25A35*
19	27875001	27925000	0.445544	*RANGRF*
19	27875001	27925000	0.445544	*ARHGEF15*
14	9975001	10025000	0.445479	*ADCY8*
4	76725001	76775000	0.445394	*ZMIZ2*
4	76725001	76775000	0.445394	*OGDH*
22	35075001	35125000	0.445277	*SLC25A26*
19	33500001	33550000	0.445211	*SPECC1*
19	33500001	33550000	0.445211	*ADORA2B*
15	64425001	64475000	0.445176	*LMO2*
3	36000001	36050000	0.44515	*NTNG1*
4	69550001	69600000	0.444955	*SNX10*
22	7350001	7400000	0.444893	*GLB1*
8	89550001	89600000	0.44489	*NXNL2*
14	23000001	23050000	0.444614	*TMEM68*
22	31975001	32025000	0.444581	*FRMD4B*
7	17825001	17875000	0.44447	*TNFSF14*
18	22000001	22050000	0.444429	*RPGRIP1L*
18	22000001	22050000	0.444429	*FTO*
19	52725001	52775000	0.444402	*CBX2*
19	52725001	52775000	0.444402	*CBX8*
19	41100001	41150000	0.444361	*KRT23*
24	57325001	57375000	0.444347	*NEDD4L*
22	30300001	30350000	0.443991	*FOXP1*
2	38225001	38275000	0.44399	*UPP2*
22	48400001	48450000	0.44395	*NISCH*
22	48400001	48450000	0.44395	*SEMA3G*
22	48400001	48450000	0.44395	*TNNC1*

3	98050001	98100000	0.443941	*SKINT1*
29	48575001	48625000	0.443869	*CARS1*
29	48575001	48625000	0.443869	*NAP1L4*
29	48725001	48775000	0.44379	*KCNQ1*
29	48725001	48775000	0.44379	*CDKN1C*
14	34875001	34925000	0.443736	*EYA1*
25	34075001	34125000	0.443644	*RHBDD2*
21	65000001	65050000	0.443607	*EVL*
21	65000001	65050000	0.443607	*U1*
14	31000001	31050000	0.443455	*SGK3*
10	30425001	30475000	0.443417	*AQR*
10	30425001	30475000	0.443417	*ZNF770*
22	49950001	50000000	0.443311	*CACNA2D2*
22	49950001	50000000	0.443311	*NPRL2*
22	49950001	50000000	0.443311	*CYB561D2*
13	36275001	36325000	0.443307	*MPP7*
28	29225001	29275000	0.443087	*FAM149B1*
28	29225001	29275000	0.443087	*ECD*
4	10675001	10725000	0.44303	*VPS50*
16	2875001	2925000	0.442824	*RBBP5*
22	51125001	51175000	0.44272	*PRKAR2A*
22	50700001	50750000	0.442627	*RHOA*
22	50700001	50750000	0.442627	*TCTA*
8	86500001	86550000	0.442581	*AUH*
19	33475001	33525000	0.442375	*ADORA2B*
19	33475001	33525000	0.442375	*ZSWIM7*
19	33475001	33525000	0.442375	*SPECC1*
19	23150001	23200000	0.442153	*SMG6*
19	38950001	39000000	0.442054	*MRPL45*
19	38950001	39000000	0.442054	*GPR179*
6	66750001	66800000	0.44192	*TXK*
8	82625001	82675000	0.441856	*ERCC6L2*
14	32450001	32500000	0.441524	*C14H8orf34*
5	105125001	105175000	0.44152	*KCNA5*
18	54125001	54175000	0.441519	*ZC3H4*
18	54125001	54175000	0.441519	*SAE1*
22	43550001	43600000	0.441458	*DENND6A*
11	78225001	78275000	0.441191	*HS1BP3*
8	80175001	80225000	0.441096	*SNORA70*
13	77775001	77825000	0.441089	*B4GALT5*
10	30825001	30875000	0.440976	*DPH6*
10	30825001	30875000	0.440976	*U6*
19	47625001	47675000	0.440968	*TANC2*
11	104375001	104425000	0.440936	*MYMK*
11	104375001	104425000	0.440936	*ADAMTSL2*
8	61400001	61450000	0.440829	*ZCCHC7*
22	7300001	7350000	0.440723	*GLB1*
22	7300001	7350000	0.440723	*CCR4*
3	15925001	15975000	0.440563	*ADAR*
19	29725001	29775000	0.440538	*PIRT*
14	1125001	1175000	0.440536	*ZC3H3*
14	1125001	1175000	0.440536	*GSDMD*

14	1125001	1175000	0.440536	*MROH6*
14	1125001	1175000	0.440536	*NAPRT*
14	1125001	1175000	0.440536	*EEF1D*
22	44100001	44150000	0.440435	*ARHGEF3*
1	2525001	2575000	0.44008	*OLIG2*
21	64900001	64950000	0.439863	*EVL*
21	64900001	64950000	0.439863	*EML1*
6	91275001	91325000	0.439821	*CCDC158*
6	91275001	91325000	0.439821	*SHROOM3*
7	14200001	14250000	0.439818	*ZNF177*
5	53750001	53800000	0.439782	*SLC16A7*
15	64450001	64500000	0.43914	*LMO2*
5	110450001	110500000	0.438978	*CBX6*
15	51550001	51600000	0.438825	*RNF121*
8	6925001	6975000	0.43863	*GLRA3*
14	35025001	35075000	0.438561	*EYA1*
15	40450001	40500000	0.438508	*MICAL2*
16	6500001	6550000	0.438507	*KCNT2*
2	124700001	124750000	0.438467	*YTHDF2*
2	124700001	124750000	0.438467	*U6*
16	2900001	2950000	0.438372	*DSTYK*
16	2900001	2950000	0.438372	*RBBP5*
22	41100001	41150000	0.438334	*FHIT*
4	10075001	10125000	0.438195	*CDK6*
8	59950001	60000000	0.438065	*NPR2*
8	59950001	60000000	0.438065	*FAM221B*
8	59950001	60000000	0.438065	*SPAG8*
8	59950001	60000000	0.438065	*HINT2*
19	46050001	46100000	0.437782	*KANSL1*
2	17750001	17800000	0.437749	*SESTD1*
2	17750001	17800000	0.437749	*CCDC141*
4	55800001	55850000	0.437738	*LSMEM1*
19	31050001	31100000	0.437616	*MYOCD*
2	28000001	28050000	0.437564	*STK39*
16	3325001	3375000	0.437453	*MFSD4A*
16	3325001	3375000	0.437453	*ELK4*
14	33600001	33650000	0.437432	*SLCO5A1*
21	45150001	45200000	0.437344	*BAZ1A*
22	7075001	7125000	0.437192	*CNOT10*
19	52400001	52450000	0.437144	*CARD14*
19	52400001	52450000	0.437144	*SLC26A11*
19	52400001	52450000	0.437144	*SGSH*
5	111275001	111325000	0.437018	*GRAP2*
20	23075001	23125000	0.436971	*ANKRD55*
2	28025001	28075000	0.436898	*STK39*
18	21975001	22025000	0.436794	*RPGRIP1L*
16	71925001	71975000	0.436722	*RD3*
13	17700001	17750000	0.436512	*YME1L1*
13	17700001	17750000	0.436512	*ANKRD26*
2	124775001	124825000	0.436464	*TAF12*
2	124775001	124825000	0.436464	*GMEB1*
14	34075001	34125000	0.43643	*NCOA2*

14	34075001	34125000	0.43643	*U6*
22	51100001	51150000	0.436186	*PRKAR2A*
8	7050001	7100000	0.436176	*GLRA3*
3	98075001	98125000	0.435958	*SKINT1*
3	19625001	19675000	0.435953	*VPS72*
3	19625001	19675000	0.435953	*LYSMD1*
3	19625001	19675000	0.435953	*TMOD4*
3	19625001	19675000	0.435953	*TNFAIP8L2*
3	19625001	19675000	0.435953	*SCNM1*
3	19625001	19675000	0.435953	*SEMA6C*
16	51175001	51225000	0.435734	*DVL1*
16	51175001	51225000	0.435734	*INTS11*
16	51175001	51225000	0.435734	*ACAP3*
16	51175001	51225000	0.435734	*CPTP*
16	51175001	51225000	0.435734	*TAS1R3*
16	51175001	51225000	0.435734	*PUSL1*
16	51175001	51225000	0.435734	*MXRA8*
19	27500001	27550000	0.435711	*DNAH2*
19	27500001	27550000	0.435711	*KDM6B*
19	27500001	27550000	0.435711	*NAA38*
19	27500001	27550000	0.435711	*CYB5D1*
19	27500001	27550000	0.435711	*TMEM88*
5	112325001	112375000	0.435595	*EP300*
5	112325001	112375000	0.435595	*L3MBTL2*
18	11500001	11550000	0.435593	*GSE1*
2	47375001	47425000	0.435237	*EPC2*
8	72675001	72725000	0.435089	*DOCK5*
3	108875001	108925000	0.435083	*GRIK3*
8	76675001	76725000	0.43472	*FRMD3*
22	51150001	51200000	0.434711	*IP6K2*
22	51150001	51200000	0.434711	*PRKAR2A*
23	18875001	18925000	0.434674	*RUNX2*
8	79250001	79300000	0.434574	*GOLM1*
8	79250001	79300000	0.434574	*NAA35*
4	39550001	39600000	0.434224	*U6*
14	34900001	34950000	0.434205	*EYA1*
2	124800001	124850000	0.43414	*TAF12*
2	124800001	124850000	0.43414	*RAB42*
10	30875001	30925000	0.434096	*DPH6*
1	149425001	149475000	0.434074	*VPS26C*
1	149425001	149475000	0.434074	*TTC3*
5	69550001	69600000	0.433974	*NUAK1*
4	12350001	12400000	0.43397	*PPP1R9A*
7	21750001	21800000	0.433629	*RAD50*
15	40725001	40775000	0.433562	*USP47*
15	40725001	40775000	0.433562	*DKK3*
3	83525001	83575000	0.433432	*PATJ*
18	46475001	46525000	0.433317	*PRODH2*
18	46475001	46525000	0.433317	*ARHGAP33*
18	46475001	46525000	0.433317	*PROSER3*
22	47800001	47850000	0.433303	*RFT1*
11	78250001	78300000	0.433151	*HS1BP3*

7	90350001	90400000	0.433114	*ADGRV1*
19	38700001	38750000	0.433101	*TBX21*
19	38700001	38750000	0.433101	*TBKBP1*
11	48475001	48525000	0.432678	*REEP1*
29	9850001	9900000	0.432477	*SYTL2*
29	9850001	9900000	0.432477	*CREBZF*
29	9850001	9900000	0.432477	*TMEM126A*
29	9850001	9900000	0.432477	*CCDC89*
22	50325001	50375000	0.432317	*MST1R*
22	50325001	50375000	0.432317	*MON1A*
22	50325001	50375000	0.432317	*RBM6*
16	45475001	45525000	0.432239	*UTS2*
16	45500001	45550000	0.432133	*UTS2*
4	10050001	10100000	0.432049	*CDK6*
21	64925001	64975000	0.431965	*EVL*
16	43750001	43800000	0.431954	*CLSTN1*
16	43750001	43800000	0.431954	*PIK3CD*
8	39425001	39475000	0.431936	*JAK2*
19	26325001	26375000	0.431873	*ZFP3*
11	3750001	3800000	0.431853	*INPP4A*
11	3750001	3800000	0.431853	*COA5*
28	29300001	29350000	0.431817	*CFAP70*
8	62100001	62150000	0.431745	*SHB*
2	38925001	38975000	0.431555	*CYTIP*
11	3650001	3700000	0.431537	*INPP4A*
19	27525001	27575000	0.431523	*KDM6B*
19	27525001	27575000	0.431523	*CHD3*
19	27525001	27575000	0.431523	*CYB5D1*
19	27525001	27575000	0.431523	*NAA38*
19	27525001	27575000	0.431523	*TMEM88*
18	14375001	14425000	0.43151	*ANKRD11*
22	48300001	48350000	0.431486	*NT5DC2*
22	48300001	48350000	0.431486	*STAB1*
19	46025001	46075000	0.431405	*KANSL1*
2	18000001	18050000	0.431367	*CCDC141*
2	18000001	18050000	0.431367	*U6*
14	36850001	36900000	0.431202	*STAU2*
8	72525001	72575000	0.431176	*DOCK5*
28	25925001	25975000	0.431051	*FAM241B*
8	72700001	72750000	0.430973	*DOCK5*
8	72700001	72750000	0.430973	*KCTD9*
8	72700001	72750000	0.430973	*GNRH1*
22	47850001	47900000	0.430878	*SFMBT1*
22	47850001	47900000	0.430878	*RFT1*
8	61750001	61800000	0.430877	*FRMPD1*
23	13575001	13625000	0.430867	*KIF6*
3	97900001	97950000	0.430862	*SPATA6*
8	7075001	7125000	0.430436	*GLRA3*
22	50725001	50775000	0.430436	*RHOA*
22	50725001	50775000	0.430436	*USP4*
22	50725001	50775000	0.430436	*GPX1*
22	50725001	50775000	0.430436	*U6*

27	34800001	34850000	0.430287	*ADAM3A*
22	30325001	30375000	0.430173	*FOXP1*
5	17800001	17850000	0.430114	*C5H12orf50*
5	17800001	17850000	0.430114	*CEP290*
5	17800001	17850000	0.430114	*C5H12orf29*
14	34050001	34100000	0.430113	*NCOA2*
29	49100001	49150000	0.430031	*KCNQ1*
20	500001	550000	0.429825	*SLIT3*
19	51100001	51150000	0.429646	*HGS*
19	51100001	51150000	0.429646	*SLC25A10*
19	51100001	51150000	0.429646	*MRPL12*
19	51100001	51150000	0.429646	*ARL16*
19	51100001	51150000	0.429646	*CCDC137*
15	40475001	40525000	0.429611	*MICAL2*
16	45400001	45450000	0.429567	*PARK7*
16	45400001	45450000	0.429567	*TNFRSF9*
3	98450001	98500000	0.429563	*TRABD2B*
5	78200001	78250000	0.429501	*AMN1*
13	17200001	17250000	0.429386	*PFKFB3*
28	44250001	44300000	0.429323	*MARCHF8*
16	6525001	6575000	0.429232	*KCNT2*
7	14425001	14475000	0.429173	*OLFM2*
7	14425001	14475000	0.429173	*PIN1*
7	14425001	14475000	0.429173	*SNORA70*
27	19950001	20000000	0.429168	*VPS37A*
27	19950001	20000000	0.429168	*CNOT7*
18	22175001	22225000	0.429084	*FTO*
19	51475001	51525000	0.42879	*CEP131*
19	51475001	51525000	0.42879	*TEPSIN*
19	51475001	51525000	0.42879	*SLC38A10*
19	51475001	51525000	0.42879	*NDUFAF8*
29	925001	975000	0.428759	*VSTM5*
11	3775001	3825000	0.428455	*COA5*
11	3775001	3825000	0.428455	*UNC50*
8	72200001	72250000	0.428424	*NEFL*
6	67600001	67650000	0.428318	*CWH43*
28	15500001	15550000	0.428284	*CCDC6*
20	53900001	53950000	0.428253	*CDH18*
23	16225001	16275000	0.428234	*UBR2*
19	26400001	26450000	0.428081	*CAMTA2*
19	26400001	26450000	0.428081	*KIF1C*
19	26400001	26450000	0.428081	*ENO3*
19	26400001	26450000	0.428081	*INCA1*
19	26400001	26450000	0.428081	*SPAG7*
19	26400001	26450000	0.428081	*PFN1*
28	26300001	26350000	0.427988	*MACROH2A2*
28	26300001	26350000	0.427988	*AIFM2*
22	41050001	41100000	0.427913	*FHIT*
19	27400001	27450000	0.427894	*DNAH2*
19	27400001	27450000	0.427894	*EFNB3*
19	27400001	27450000	0.427894	*WRAP53*
7	90525001	90575000	0.427847	*ADGRV1*

11	44925001	44975000	0.427812	*U6*
14	1175001	1225000	0.427669	*ZC3H3*
19	44875001	44925000	0.427627	*FMNL1*
19	44875001	44925000	0.427627	*MAP3K14*
3	106400001	106450000	0.427476	*NT5C1A*
3	106400001	106450000	0.427476	*HEYL*
15	40775001	40825000	0.427432	*USP47*
5	104000001	104050000	0.427426	*SCNN1A*
5	104000001	104050000	0.427426	*TNFRSF1A*
5	104000001	104050000	0.427426	*PLEKHG6*
8	11625001	11675000	0.427254	*ACO1*
18	11750001	11800000	0.427213	*EMC8*
18	11750001	11800000	0.427213	*COX4I1*
22	50400001	50450000	0.427187	*TRAIP*
22	50400001	50450000	0.427187	*UBA7*
22	50400001	50450000	0.427187	*CDHR4*
22	50400001	50450000	0.427187	*IP6K1*
22	50400001	50450000	0.427187	*INKA1*
2	38200001	38250000	0.427136	*UPP2*
2	38200001	38250000	0.427136	*CCDC148*
28	44225001	44275000	0.426971	*MARCHF8*
20	31900001	31950000	0.426308	*GHR*
19	38725001	38775000	0.426156	*TBKBP1*
19	38725001	38775000	0.426156	*TBX21*
19	38725001	38775000	0.426156	*KPNB1*
4	57650001	57700000	0.425875	*IMMP2L*
22	46775001	46825000	0.425814	*CACNA2D3*
7	14450001	14500000	0.425616	*OLFM2*
7	14450001	14500000	0.425616	*SNORA70*
7	14450001	14500000	0.425616	*SNORA70*
8	78975001	79025000	0.425344	*AGTPBP1*
8	61475001	61525000	0.425289	*ZCCHC7*
24	300001	350000	0.42524	*PARD6G*
29	49500001	49550000	0.425213	*MRPL23*
8	82125001	82175000	0.425061	*PTCH1*
19	51450001	51500000	0.424771	*SLC38A10*
19	51450001	51500000	0.424771	*TEPSIN*
19	51450001	51500000	0.424771	*NDUFAF8*
28	25225001	25275000	0.424573	*KIFBP*
28	25225001	25275000	0.424573	*DDX21*
8	61025001	61075000	0.424556	*PAX5*
14	39950001	40000000	0.424484	*ZFHX4*
24	57450001	57500000	0.424471	*NEDD4L*
16	43400001	43450000	0.424469	*UBE4B*
29	49725001	49775000	0.424447	*IFITM10*
20	23050001	23100000	0.424404	*ANKRD55*
22	48100001	48150000	0.424326	*NEK4*
22	48100001	48150000	0.424326	*ITIH1*
8	79275001	79325000	0.42415	*GOLM1*
19	27825001	27875000	0.423786	*CTC1*
19	27825001	27875000	0.423786	*AURKB*
19	27825001	27875000	0.423786	*BORCS6*

5	78150001	78200000	0.423661	*AMN1*
21	45175001	45225000	0.423635	*BAZ1A*
4	81050001	81100000	0.423472	*CDK13*
28	33100001	33150000	0.423441	*KCNMA1*
29	49525001	49575000	0.423429	*MRPL23*
4	12425001	12475000	0.423428	*PPP1R9A*
22	51075001	51125000	0.423417	*PRKAR2A*
22	51075001	51125000	0.423417	*SLC25A20*
28	17600001	17650000	0.423375	*CABCOCO1*
22	49075001	49125000	0.422996	*GRM2*
22	49075001	49125000	0.422996	*IQCF6*
23	15475001	15525000	0.422908	*TFEB*
20	41900001	41950000	0.422897	*PDZD2*
2	26750001	26800000	0.422885	*BBS5*
2	26750001	26800000	0.422885	*KLHL41*
8	62125001	62175000	0.422865	*SHB*
18	51825001	51875000	0.422676	*CADM4*
18	51825001	51875000	0.422676	*PLAUR*
18	51825001	51875000	0.422676	*ZNF428*
18	51825001	51875000	0.422676	*SRRM5*
14	23250001	23300000	0.422406	*RPS20*
14	23250001	23300000	0.422406	*MOS*
14	23250001	23300000	0.422406	*U1*
7	7775001	7825000	0.422345	*AKAP8*
16	41450001	41500000	0.422177	*VPS13D*
16	41450001	41500000	0.422177	*TNFRSF1B*
8	72650001	72700000	0.422081	*DOCK5*
22	48525001	48575000	0.421993	*DNAH1*
8	11650001	11700000	0.421898	*ACO1*
29	47825001	47875000	0.421819	*SHANK2*
14	34850001	34900000	0.421729	*EYA1*
8	76700001	76750000	0.421726	*FRMD3*
7	7625001	7675000	0.42168	*U6*
20	23025001	23075000	0.421624	*ANKRD55*
11	55625001	55675000	0.421541	*CTNNA2*
23	16750001	16800000	0.421532	*PTK7*
23	16750001	16800000	0.421532	*CUL9*
23	16750001	16800000	0.421532	*SRF*
27	34825001	34875000	0.421519	*ADAM18*
11	19700001	19750000	0.421422	*PRKD3*
5	47625001	47675000	0.42141	*TMBIM4*
5	47625001	47675000	0.42141	*LLPH*
28	26775001	26825000	0.421391	*PRF1*
8	7525001	7575000	0.421301	*CTSB*
2	18925001	18975000	0.42126	*PDE11A*
21	45475001	45525000	0.421245	*PRORP*
21	45475001	45525000	0.421245	*U6*
28	26250001	26300000	0.421216	*MACROH2A2*
16	41525001	41575000	0.421178	*TNFRSF8*
16	41525001	41575000	0.421178	*TNFRSF1B*
5	17775001	17825000	0.4211	*C5H12orf50*
23	11100001	11150000	0.421097	*TMEM217*

23	11100001	11150000	0.421097	*TBC1D22B*
18	50375001	50425000	0.421048	*AXL*
18	50375001	50425000	0.421048	*CYP2S1*
8	61150001	61200000	0.420968	*PAX5*
18	2400001	2450000	0.420791	*ZNRF1*
26	26000001	26050000	0.420761	*SORCS3*
8	71500001	71550000	0.420758	*ADAM28*
14	44200001	44250000	0.420758	*PAG1*
22	30425001	30475000	0.420755	*FOXP1*
19	27675001	27725000	0.420689	*ALOX15B*
19	27675001	27725000	0.420689	*ALOX12B*
19	27675001	27725000	0.420689	*GUCY2D*
19	42250001	42300000	0.42068	*RAB5C*
19	42250001	42300000	0.42068	*KCNH4*
19	42250001	42300000	0.42068	*KAT2A*
19	42250001	42300000	0.42068	*HSPB9*
23	16550001	16600000	0.420598	*CNPY3*
23	16550001	16600000	0.420598	*GNMT*
23	16550001	16600000	0.420598	*PTCRA*
27	34775001	34825000	0.420585	*ADAM3A*
18	53400001	53450000	0.420468	*MYPOP*
18	53400001	53450000	0.420468	*NOVA2*
18	53400001	53450000	0.420468	*NANOS2*
19	44925001	44975000	0.420385	*MAP3K14*
16	45350001	45400000	0.420348	*ERRFI1*
4	57575001	57625000	0.420325	*IMMP2L*
4	12500001	12550000	0.420216	*PPP1R9A*
4	12500001	12550000	0.420216	*PON1*
10	30850001	30900000	0.420175	*DPH6*
7	14550001	14600000	0.420175	*COL5A3*
7	14550001	14600000	0.420175	*RDH8*
21	40175001	40225000	0.420012	*PRKD1*
14	35050001	35100000	0.41997	*EYA1*
20	14750001	14800000	0.419864	*SHISAL2B*
20	14750001	14800000	0.419864	*SREK1IP1*
4	80500001	80550000	0.419833	*SUGCT*
15	39875001	39925000	0.419768	*TEAD1*
16	65100001	65150000	0.419692	*TSEN15*
22	50500001	50550000	0.419623	*BSN*
22	50500001	50550000	0.419623	*APEH*
22	50500001	50550000	0.419623	*RNF123*
22	50500001	50550000	0.419623	*MST1*
23	15450001	15500000	0.419576	*MDFI*
23	15450001	15500000	0.419576	*TFEB*
20	48850001	48900000	0.419538	*CDH10*
1	149300001	149350000	0.419509	*TTC3*
1	149300001	149350000	0.419509	*PIGP*
2	23300001	23350000	0.419501	*CDCA7*
21	42100001	42150000	0.41943	*NUBPL*
20	14375001	14425000	0.419417	*U4*
13	17925001	17975000	0.41933	*PDSS1*
22	45275001	45325000	0.419243	*ERC2*

19	52050001	52100000	0.419179	*ENDOV*
19	52050001	52100000	0.419179	*NPTX1*
21	64975001	65025000	0.419166	*EVL*
21	64975001	65025000	0.419166	*U1*
2	47350001	47400000	0.419086	*KIF5C*
19	51275001	51325000	0.419022	*BAHCC1*
23	18850001	18900000	0.418982	*RUNX2*
18	46400001	46450000	0.418981	*KMT2B*
18	46400001	46450000	0.418981	*UPK1A*
18	46400001	46450000	0.418981	*ZBTB32*
19	23075001	23125000	0.418849	*SMG6*
19	23075001	23125000	0.418849	*HIC1*
22	48125001	48175000	0.418845	*NEK4*
22	48125001	48175000	0.418845	*GLT8D1*
22	48125001	48175000	0.418845	*GNL3*
22	48125001	48175000	0.418845	*SPCS1*
22	48125001	48175000	0.418845	*SNORD19B*
22	48125001	48175000	0.418845	*SNORD69*
22	48125001	48175000	0.418845	*SNORD19*
22	48125001	48175000	0.418845	*SNORD19C*
13	77850001	77900000	0.418718	*SLC9A8*
21	2775001	2825000	0.418711	*ATP10A*
4	80525001	80575000	0.418671	*SUGCT*
7	90075001	90125000	0.418631	*LYSMD3*
7	90075001	90125000	0.418631	*POLR3G*
19	31250001	31300000	0.418553	*ARHGAP44*
1	2500001	2550000	0.418526	*OLIG2*
19	23100001	23150000	0.418317	*SMG6*
19	23100001	23150000	0.418317	*U6*
8	82525001	82575000	0.418306	*ERCC6L2*
20	3100001	3150000	0.418257	*RANBP17*
19	50825001	50875000	0.418252	*RAC3*
19	50825001	50875000	0.418252	*DCXR*
19	50825001	50875000	0.418252	*DCXR*
19	50825001	50875000	0.418252	*RFNG*
18	51900001	51950000	0.418244	*SMG9*
18	51900001	51950000	0.418244	*IRGC*
23	18925001	18975000	0.418213	*RUNX2*
5	47850001	47900000	0.41812	*HMGA2*
28	25300001	25350000	0.418027	*KIFBP*
5	8975001	9025000	0.417988	*SYT1*
2	38250001	38300000	0.417973	*UPP2*
2	18750001	18800000	0.417922	*RBM45*
2	18750001	18800000	0.417922	*CYCT*
18	11800001	11850000	0.417886	*IRF8*
14	900001	950000	0.41788	*EPPK1*
5	108850001	108900000	0.417855	*ADA2*
23	16725001	16775000	0.417834	*PTK7*
8	16250001	16300000	0.41777	*LINGO2*
2	124675001	124725000	0.417764	*YTHDF2*
18	11725001	11775000	0.417715	*EMC8*
18	11725001	11775000	0.417715	*COX4I1*

19	50000001	50050000	0.417692	*FOXK2*
19	50000001	50050000	0.417692	*WDR45B*
21	24600001	24650000	0.417692	*SH3GL3*
23	15050001	15100000	0.417652	*TREM2*
23	15050001	15100000	0.417652	*TREML1*
4	10700001	10750000	0.417583	*VPS50*
1	145950001	146000000	0.417521	*MCM3AP*
1	145950001	146000000	0.417521	*C1H21orf58*
1	145950001	146000000	0.417521	*YBEY*
14	33825001	33875000	0.417429	*NCOA2*
20	14300001	14350000	0.417406	*ADAMTS6*
3	120625001	120675000	0.417342	*BOK*
3	120625001	120675000	0.417342	*THAP4*
3	107050001	107100000	0.417186	*RHBDL2*
2	47400001	47450000	0.417142	*EPC2*
18	14425001	14475000	0.417029	*ANKRD11*
18	14425001	14475000	0.417029	*SPG7*
3	108900001	108950000	0.417002	*GRIK3*
28	29075001	29125000	0.416867	*P4HA1*
16	3200001	3250000	0.41678	*CDK18*
16	51225001	51275000	0.416715	*UBE2J2*
16	51225001	51275000	0.416715	*SCNN1D*
16	51225001	51275000	0.416715	*SDF4*
16	51225001	51275000	0.416715	*ACAP3*
16	51225001	51275000	0.416715	*C1QTNF12*
16	51225001	51275000	0.416715	*B3GALT6*
19	41125001	41175000	0.416712	*KRT23*
19	41125001	41175000	0.416712	*KRT39*
5	47775001	47825000	0.416619	*HMGA2*
13	78000001	78050000	0.416539	*RNF114*
13	78000001	78050000	0.416539	*SNAI1*
13	78000001	78050000	0.416539	*SNORA70*
28	26550001	26600000	0.416256	*LRRC20*
8	39275001	39325000	0.415985	*PLGRKT*
15	64475001	64525000	0.415944	*LMO2*
5	111300001	111350000	0.415887	*GRAP2*
8	78950001	79000000	0.415874	*AGTPBP1*
19	43550001	43600000	0.415849	*MEOX1*
28	24850001	24900000	0.415706	*SLC25A16*
20	475001	525000	0.415698	*SLIT3*
7	89975001	90025000	0.41566	*MBLAC2*
14	33625001	33675000	0.415576	*SLCO5A1*
1	149450001	149500000	0.415547	*VPS26C*
7	7750001	7800000	0.415457	*AKAP8*
7	7750001	7800000	0.415457	*AKAP8L*
29	49200001	49250000	0.415398	*CD81*
29	9825001	9875000	0.415366	*SYTL2*
29	9825001	9875000	0.415366	*CCDC89*
19	12950001	13000000	0.415365	*MRM1*
15	79325001	79375000	0.415247	*OR8K62*
15	79325001	79375000	0.415247	*OR5AL8*
7	7900001	7950000	0.415217	*NOTCH3*

3	120925001	120975000	0.415185	*U6*
2	124850001	124900000	0.415084	*TRNAU1AP*
2	124850001	124900000	0.415084	*RCC1*
2	124850001	124900000	0.415084	*SNORA44*
2	124850001	124900000	0.415084	*SNORA61*
2	124850001	124900000	0.415084	*SNORD99*
20	14450001	14500000	0.415033	*CWC27*
8	91300001	91350000	0.415009	*GRIN3A*
8	91300001	91350000	0.415009	*RNF20*
19	12925001	12975000	0.414978	*DHRS11*
19	12925001	12975000	0.414978	*MRM1*
19	12925001	12975000	0.414978	*GGNBP2*
4	5225001	5275000	0.414966	*GRB10*
8	39325001	39375000	0.414924	*INSL6*
9	15475001	15525000	0.414775	*MYO6*
18	22050001	22100000	0.414716	*FTO*
19	28800001	28850000	0.414699	*STX8*
22	49925001	49975000	0.41469	*CACNA2D2*
14	33575001	33625000	0.41463	*SLCO5A1*
20	20275001	20325000	0.414619	*PDE4D*
28	26750001	26800000	0.414494	*PALD1*
28	26750001	26800000	0.414494	*PRF1*
28	15475001	15525000	0.41448	*CCDC6*
2	18900001	18950000	0.41439	*PDE11A*
3	19675001	19725000	0.414353	*GABPB2*
3	19675001	19725000	0.414353	*SEMA6C*
3	19675001	19725000	0.414353	*MLLT11*
18	2375001	2425000	0.414251	*ZNRF1*
5	108875001	108925000	0.41399	*ADA2*
19	41175001	41225000	0.413924	*KRT40*
19	41175001	41225000	0.413924	*KRTAP3-3*
19	41175001	41225000	0.413924	*KRTAP3-1*
14	44175001	44225000	0.41377	*PAG1*
27	34850001	34900000	0.413759	*ADAM18*
25	25700001	25750000	0.41375	*XPO6*
28	8050001	8100000	0.413533	*ARID4B*
28	8050001	8100000	0.413533	*GGPS1*
28	8050001	8100000	0.413533	*TBCE*
8	61575001	61625000	0.413471	*ZBTB5*
8	61575001	61625000	0.413471	*POLR1E*
8	61575001	61625000	0.413471	*U6*
16	3650001	3700000	0.413347	*SLC26A9*
22	7100001	7150000	0.413117	*CNOT10*
19	23025001	23075000	0.413052	*RTN4RL1*
19	23025001	23075000	0.413052	*DPH1*
19	23025001	23075000	0.413052	*OVCA2*
19	23025001	23075000	0.413052	*HIC1*
19	46000001	46050000	0.41305	*KANSL1*
19	46000001	46050000	0.41305	*MAPT*
28	250001	300000	0.412967	*OR5L20*
28	250001	300000	0.412967	*OR5D18K*
1	138925001	138975000	0.412901	*ATP2C1*

29	48500001	48550000	0.412834	*OSBPL5*
29	48500001	48550000	0.412834	*U6*
19	50700001	50750000	0.412829	*CCDC57*
4	69775001	69825000	0.412715	*NFE2L3*
19	44550001	44600000	0.412681	*EFTUD2*
19	44550001	44600000	0.412681	*GJC1*
19	44550001	44600000	0.412681	*HIGD1B*
22	43600001	43650000	0.412632	*ARF4*
22	43600001	43650000	0.412632	*PDE12*
4	12450001	12500000	0.412449	*PPP1R9A*
11	104400001	104450000	0.412434	*ADAMTSL2*
11	104400001	104450000	0.412434	*MYMK*
19	44075001	44125000	0.4124	*RUNDC3A*
19	44075001	44125000	0.4124	*SLC4A1*
16	3475001	3525000	0.412376	*NUCKS1*
16	3475001	3525000	0.412376	*RAB29*
20	14425001	14475000	0.412363	*CWC27*
2	8850001	8900000	0.412354	*TFPI*
2	8850001	8900000	0.412354	*CALCRL*
4	40600001	40650000	0.412206	*GNAT3*
14	1075001	1125000	0.412095	*EEF1D*
14	1075001	1125000	0.412095	*PYCR3*
14	1075001	1125000	0.412095	*GFUS*
14	1075001	1125000	0.412095	*TIGD5*
2	89075001	89125000	0.41204	*SGO2*
2	89075001	89125000	0.41204	*AOX1*
7	42850001	42900000	0.411932	*OR2AV11*
7	42850001	42900000	0.411932	*OR2AZ3*
22	49150001	49200000	0.411929	*RAD54L2*
22	49150001	49200000	0.411929	*TEX264*
18	11075001	11125000	0.411787	*ZDHHC7*
7	90000001	90050000	0.411737	*MBLAC2*
7	90000001	90050000	0.411737	*POLR3G*
7	7600001	7650000	0.411719	*U6*
11	100675001	100725000	0.411716	*HMCN2*
5	17750001	17800000	0.411594	*C5H12orf50*
1	156375001	156425000	0.411573	*KCNH8*
2	124875001	124925000	0.411568	*RCC1*
2	124875001	124925000	0.411568	*TRNAU1AP*
2	124875001	124925000	0.411568	*PHACTR4*
2	124875001	124925000	0.411568	*SNORA73*
2	124875001	124925000	0.411568	*SNORA73*
2	124875001	124925000	0.411568	*SNORA73*
19	28825001	28875000	0.411544	*STX8*
19	28825001	28875000	0.411544	*CFAP52*
4	5375001	5425000	0.411498	*DDC*
15	39925001	39975000	0.411492	*TEAD1*
16	3300001	3350000	0.411394	*MFSD4A*
3	625001	675000	0.411301	*DCAF6*
16	43625001	43675000	0.411118	*CTNNBIP1*
3	48275001	48325000	0.41108	*TLCD4*
22	49225001	49275000	0.410914	*RAD54L2*

20	53925001	53975000	0.410884	*CDH18*
18	22600001	22650000	0.410851	*IRX3*
19	51525001	51575000	0.410676	*AATK*
29	49225001	49275000	0.410609	*TSPAN32*
7	30975001	31025000	0.410524	*SNX2*
7	30975001	31025000	0.410524	*SNX24*
1	156350001	156400000	0.410462	*KCNH8*
18	46500001	46550000	0.410224	*NPHS1*
18	46500001	46550000	0.410224	*PRODH2*
18	46500001	46550000	0.410224	*ARHGAP33*
18	46500001	46550000	0.410224	*U6*
2	26725001	26775000	0.409976	*BBS5*
2	26725001	26775000	0.409976	*KLHL41*
2	26725001	26775000	0.409976	*FASTKD1*
29	950001	1000000	0.409874	*VSTM5*
3	120450001	120500000	0.409809	*HDLBP*
3	120450001	120500000	0.409809	*SEPTIN2*
22	47825001	47875000	0.409635	*RFT1*
2	47325001	47375000	0.409627	*KIF5C*
3	58475001	58525000	0.409601	*CCN1*
19	38975001	39025000	0.40959	*GPR179*
19	38975001	39025000	0.40959	*SOCS7*
19	38975001	39025000	0.40959	*MRPL45*
22	35175001	35225000	0.409464	*SLC25A26*
5	81850001	81900000	0.409404	*PTHLH*
2	124725001	124775000	0.409395	*GMEB1*
2	124725001	124775000	0.409395	*YTHDF2*
2	124725001	124775000	0.409395	*U6*
11	19675001	19725000	0.409377	*PRKD3*
11	19675001	19725000	0.409377	*NDUFAF7*
11	11500001	11550000	0.409256	*EMX1*
11	11500001	11550000	0.409256	*SPR*
3	103575001	103625000	0.40925	*CCDC30*
14	1200001	1250000	0.409186	*ZC3H3*
14	1200001	1250000	0.409186	*MAFA*
14	33975001	34025000	0.409141	*NCOA2*
5	108825001	108875000	0.409074	*ADA2*
5	108825001	108875000	0.409074	*HDHD5*
16	43675001	43725000	0.409053	*CTNNBIP1*
28	28875001	28925000	0.409015	*MCU*
28	28875001	28925000	0.409015	*OIT3*
28	28875001	28925000	0.409015	*U6*
20	3050001	3100000	0.408992	*RANBP17*
15	43200001	43250000	0.408976	*IPO7*
15	43200001	43250000	0.408976	*SNORA23*
22	33375001	33425000	0.408713	*TAFA1*
16	6750001	6800000	0.408705	*KCNT2*
25	25725001	25775000	0.408651	*XPO6*
20	14275001	14325000	0.408616	*ADAMTS6*
8	82550001	82600000	0.408538	*ERCC6L2*
19	23125001	23175000	0.408508	*SMG6*
19	23125001	23175000	0.408508	*U6*

8	61525001	61575000	0.408395	*GRHPR*
8	61525001	61575000	0.408395	*ZBTB5*
28	26650001	26700000	0.408386	*PALD1*
8	85875001	85925000	0.408122	*ROR2*
3	103725001	103775000	0.408058	*ZMYND12*
4	57675001	57725000	0.408005	*IMMP2L*
5	112550001	112600000	0.408002	*ACO2*
5	112550001	112600000	0.408002	*PHF5A*
5	110425001	110475000	0.407981	*NPTXR*
5	110425001	110475000	0.407981	*CBX6*
28	28250001	28300000	0.407856	*ASCC1*
28	28250001	28300000	0.407856	*ANAPC16*
6	91250001	91300000	0.407723	*CCDC158*
6	91250001	91300000	0.407723	*U6*
5	47600001	47650000	0.407712	*IRAK3*
5	47600001	47650000	0.407712	*TMBIM4*
13	77875001	77925000	0.407672	*SLC9A8*
29	28400001	28450000	0.407639	*CCDC15*
16	38575001	38625000	0.407596	*MROH9*
19	56250001	56300000	0.407494	*NUP85*
19	56250001	56300000	0.407494	*SUMO2*
16	43650001	43700000	0.407306	*CTNNBIP1*
8	7775001	7825000	0.407265	*BLK*
29	28150001	28200000	0.407258	*SPA17*
29	28150001	28200000	0.407258	*SIAE*
11	11875001	11925000	0.407208	*EXOC6B*
28	35450001	35500000	0.40719	*CL46*
11	103975001	104025000	0.407143	*NOTCH1*
23	16675001	16725000	0.407114	*PTK7*
23	16675001	16725000	0.407114	*KLC4*
23	16675001	16725000	0.407114	*CUL7*
23	16675001	16725000	0.407114	*MRPL2*
11	101325001	101375000	0.407067	*NUP214*
14	30975001	31025000	0.406931	*SGK3*
14	23225001	23275000	0.406918	*LYN*
10	32375001	32425000	0.40689	*CDIN1*
15	39900001	39950000	0.406836	*TEAD1*
4	85625001	85675000	0.406743	*CPED1*
14	10350001	10400000	0.406614	*ASAP1*
19	39175001	39225000	0.406536	*SRCIN1*
3	98000001	98050000	0.4065	*SLC5A9*
4	51875001	51925000	0.406214	*CAV1*
4	51875001	51925000	0.406214	*CAV2*
22	30625001	30675000	0.406203	*FOXP1*
16	6725001	6775000	0.406199	*KCNT2*
22	50800001	50850000	0.406126	*IHO1*
22	50800001	50850000	0.406126	*C22H3orf62*
22	50800001	50850000	0.406126	*C22H3orf84*
22	50800001	50850000	0.406126	*USP4*
3	106000001	106050000	0.406061	*PPT1*
3	106000001	106050000	0.406061	*CAP1*
8	11600001	11650000	0.406042	*ACO1*

8	11600001	11650000	0.406042	*DDX58*
19	52475001	52525000	0.405982	*CCDC40*
19	52475001	52525000	0.405982	*GAA*
19	52475001	52525000	0.405982	*EIF4A3*
11	68500001	68550000	0.405948	*TIA1*
11	68500001	68550000	0.405948	*C11H2orf42*
2	18875001	18925000	0.405885	*PDE11A*
1	1650001	1700000	0.405873	*ITSN1*
1	1650001	1700000	0.405873	*ATP5PO*
14	1050001	1100000	0.405774	*MAPK15*
14	1050001	1100000	0.405774	*GFUS*
14	1050001	1100000	0.405774	*CCDC166*
14	1050001	1100000	0.405774	*ZNF623*
11	11800001	11850000	0.40562	*EXOC6B*
19	44050001	44100000	0.405531	*SLC4A1*
19	51500001	51550000	0.405475	*CEP131*
19	51500001	51550000	0.405475	*AATK*
4	80550001	80600000	0.405397	*SUGCT*
16	38600001	38650000	0.405383	*MROH9*
16	38600001	38650000	0.405383	*FMO3*
7	53825001	53875000	0.405253	*ARHGAP26*
15	43225001	43275000	0.405209	*IPO7*
3	103750001	103800000	0.405184	*RIMKLA*
3	103750001	103800000	0.405184	*ZMYND12*
15	52700001	52750000	0.405177	*P2RY2*
22	33850001	33900000	0.405156	*SUCLG2*
15	62825001	62875000	0.405051	*RCN1*
28	25450001	25500000	0.404966	*SRGN*
3	103975001	104025000	0.404933	*FOXJ3*
3	103975001	104025000	0.404933	*GUCA2B*
3	103975001	104025000	0.404933	*GUCA2A*
4	81075001	81125000	0.404896	*CDK13*
12	19475001	19525000	0.404889	*MIR15A*
4	40625001	40675000	0.404626	*GNAT3*
29	48475001	48525000	0.40462	*OSBPL5*
8	94550001	94600000	0.404588	*NIPSNAP3A*
14	875001	925000	0.404518	*PLEC*
3	98025001	98075000	0.404515	*SKINT1*
11	48500001	48550000	0.404498	*REEP1*
8	72725001	72775000	0.404477	*KCTD9*
8	72725001	72775000	0.404477	*GNRH1*
28	4325001	4375000	0.404426	*TSNAX*
28	28050001	28100000	0.404201	*CHST3*
7	7700001	7750000	0.404123	*WIZ*
7	7700001	7750000	0.404123	*AKAP8L*
7	7700001	7750000	0.404123	*RASAL3*
6	57950001	58000000	0.403929	*KLF3*
23	11125001	11175000	0.40391	*TBC1D22B*
23	11125001	11175000	0.40391	*TMEM217*
8	7450001	7500000	0.403899	*DEFB134*
8	86950001	87000000	0.403893	*SYK*
11	68525001	68575000	0.40373	*TIA1*

14	9950001	10000000	0.403624	*ADCY8*
5	111575001	111625000	0.403594	*TNRC6B*
2	17800001	17850000	0.403458	*CCDC141*
22	42875001	42925000	0.403363	*KCTD6*
22	42875001	42925000	0.403363	*PDHB*
22	42875001	42925000	0.403363	*PXK*
22	48325001	48375000	0.40328	*STAB1*
22	48325001	48375000	0.40328	*NT5DC2*
22	48325001	48375000	0.40328	*NISCH*
14	81900001	81950000	0.403278	*SNTB1*
19	28650001	28700000	0.403211	*STX8*
28	28150001	28200000	0.403165	*ASCC1*
28	28150001	28200000	0.403165	*SPOCK2*
7	21725001	21775000	0.40308	*RAD50*
3	89575001	89625000	0.403046	*PLPP3*
22	47775001	47825000	0.403003	*RFT1*
22	47775001	47825000	0.403003	*PRKCD*
8	39250001	39300000	0.402971	*PLGRKT*
8	39250001	39300000	0.402971	*CD274*
19	47150001	47200000	0.402962	*MARCHF10*
2	18775001	18825000	0.402933	*CYCT*
2	18775001	18825000	0.402933	*RBM45*
14	1100001	1150000	0.402899	*EEF1D*
14	1100001	1150000	0.402899	*PYCR3*
14	1100001	1150000	0.402899	*GSDMD*
14	1100001	1150000	0.402899	*MROH6*
14	1100001	1150000	0.402899	*NAPRT*
14	1100001	1150000	0.402899	*TIGD5*
3	103900001	103950000	0.402836	*FOXJ3*
18	11825001	11875000	0.402828	*IRF8*
19	38850001	38900000	0.402807	*NPEPPS*
19	38850001	38900000	0.402807	*U6*
2	124750001	124800000	0.402743	*GMEB1*
29	5225001	5275000	0.402734	*TRIM64*
14	81875001	81925000	0.402649	*SNTB1*
3	35675001	35725000	0.402518	*VAV3*
16	6775001	6825000	0.402428	*KCNT2*
28	26625001	26675000	0.402333	*NODAL*
28	26625001	26675000	0.402333	*EIF4EBP2*
10	32400001	32450000	0.402322	*CDIN1*
28	15525001	15575000	0.402322	*CCDC6*
14	1225001	1275000	0.402301	*RHPN1*
14	1225001	1275000	0.402301	*MAFA*
7	12275001	12325000	0.402272	*CACNA1A*
18	52775001	52825000	0.402235	*PPP1R37*
18	52775001	52825000	0.402235	*NKPD1*
18	52775001	52825000	0.402235	*TRAPPC6A*
7	10475001	10525000	0.402228	*OR7A96*
7	10475001	10525000	0.402228	*OR7A91*
16	45650001	45700000	0.402169	*CAMTA1*
22	60325001	60375000	0.402118	*CHCHD6*
7	14225001	14275000	0.402067	*ZNF177*

3	119150001	119200000	0.402023	*OR9S35P*
3	119150001	119200000	0.402023	*OR9S43*
3	119150001	119200000	0.402023	*OR9S43B*
19	27575001	27625000	0.401904	*CHD3*
19	27575001	27625000	0.401904	*CNTROB*
19	27575001	27625000	0.401904	*KCNAB3*
19	27575001	27625000	0.401904	*RNF227*
19	27575001	27625000	0.401904	*TRAPPC1*
21	64950001	65000000	0.401847	*EVL*
3	120675001	120725000	0.401782	*THAP4*
3	120675001	120725000	0.401782	*ATG4B*
5	8950001	9000000	0.401773	*SYT1*
20	3125001	3175000	0.401738	*RANBP17*
20	3125001	3175000	0.401738	*TLX3*
8	70825001	70875000	0.401672	*NKX2-6*
8	85750001	85800000	0.401645	*SPTLC1*
29	49125001	49175000	0.401633	*KCNQ1*
29	49125001	49175000	0.401633	*TRPM5*
11	44975001	45025000	0.401551	*SLC5A7*
7	90550001	90600000	0.40153	*ADGRV1*
1	1725001	1775000	0.401457	*ITSN1*
26	34125001	34175000	0.401404	*CASP7*
26	34125001	34175000	0.401404	*NRAP*
18	2700001	2750000	0.401398	*CFDP1*
18	2700001	2750000	0.401398	*CFDP2*
22	34100001	34150000	0.401242	*SUCLG2*
22	52550001	52600000	0.401231	*CCDC12*
22	52550001	52600000	0.401231	*PTH1R*
4	40000001	40050000	0.401175	*SEMA3C*
11	14175001	14225000	0.401152	*XDH*
3	120525001	120575000	0.401113	*FARP2*
22	30275001	30325000	0.400958	*FOXP1*
2	18375001	18425000	0.400867	*PLEKHA3*
2	18375001	18425000	0.400867	*FKBP7*
3	725001	775000	0.400826	*DCAF6*
3	725001	775000	0.400826	*MPC2*
22	49250001	49300000	0.400624	*RAD54L2*
22	49250001	49300000	0.400624	*DCAF1*
4	39525001	39575000	0.400608	*U6*
1	56275001	56325000	0.400579	*CD96*
28	44375001	44425000	0.400568	*ALOX5*
18	2550001	2600000	0.400534	*BCAR1*
18	2550001	2600000	0.400534	*CTRB1*
23	13300001	13350000	0.40052	*KIF6*
26	22100001	22150000	0.400511	*BTRC*
18	49200001	49250000	0.40046	*EID2*
14	44225001	44275000	0.400408	*PAG1*
28	27750001	27800000	0.400383	*CDH23*
11	11550001	11600000	0.400277	*SPR*
14	36650001	36700000	0.400244	*RDH10*
15	64400001	64450000	0.40015	*FBXO3*
2	124650001	124700000	0.400065	*OPRD1*

23	47975001	48025000	0.40003	*SSR1*
7	11875001	11925000	0.399966	*YJU2B*
7	11875001	11925000	0.399966	*MRI1*
7	11875001	11925000	0.399966	*C7H19orf53*
8	10975001	11025000	0.399929	*ESCO2*
8	10975001	11025000	0.399929	*PBK*
8	10975001	11025000	0.399929	*CCDC25*
22	33400001	33450000	0.399772	*TAFA1*
13	70850001	70900000	0.399729	*PTPRT*
5	67450001	67500000	0.399683	*NT5DC3*
29	12775001	12825000	0.399675	*PRCP*
1	1625001	1675000	0.399648	*ITSN1*
1	1625001	1675000	0.399648	*ATP5PO*
18	34150001	34200000	0.399537	*CDH5*
18	34150001	34200000	0.399537	*BEAN1*
18	11175001	11225000	0.399509	*CIBAR2*
18	11175001	11225000	0.399509	*KIAA0513*
5	111650001	111700000	0.399454	*ADSL*
5	111650001	111700000	0.399454	*TNRC6B*
3	57150001	57200000	0.399421	*SELENOF*
17	6900001	6950000	0.399378	*LRBA*
19	38525001	38575000	0.399359	*SP2*
19	38525001	38575000	0.399359	*PNPO*
10	30925001	30975000	0.39933	*DPH6*
18	22575001	22625000	0.399248	*IRX3*
7	12850001	12900000	0.399236	*MAN2B1*
7	12850001	12900000	0.399236	*ZNF791*
3	78350001	78400000	0.399181	*MIER1*
3	120250001	120300000	0.39915	*SNED1*
2	124825001	124875000	0.399081	*TRNAU1AP*
2	124825001	124875000	0.399081	*TAF12*
2	124825001	124875000	0.399081	*RAB42*
2	124825001	124875000	0.399081	*SNORA44*
2	124825001	124875000	0.399081	*SNORA61*
2	124825001	124875000	0.399081	*SNORD99*
8	79300001	79350000	0.399065	*GOLM1*
13	77900001	77950000	0.399057	*SLC9A8*
16	43425001	43475000	0.399033	*UBE4B*
23	11150001	11200000	0.39895	*TBC1D22B*
5	104425001	104475000	0.398937	*ANO2*
1	138950001	139000000	0.398827	*ATP2C1*
7	21875001	21925000	0.398755	*IL5*
3	108800001	108850000	0.398619	*GRIK3*
11	12350001	12400000	0.398576	*CYP26B1*
22	48575001	48625000	0.398526	*GLYCTK*
8	65000001	65050000	0.398463	*STX17*
8	65000001	65050000	0.398463	*ERP44*
29	49550001	49600000	0.39841	*TNNT3*
29	49550001	49600000	0.39841	*PRR33*
29	49550001	49600000	0.39841	*LSP1*
7	14475001	14525000	0.398344	*OLFM2*
7	14475001	14525000	0.398344	*SNORA70*

14	44150001	44200000	0.398326	*PAG1*
14	36825001	36875000	0.3983	*STAU2*
21	45450001	45500000	0.398048	*PRORP*
21	45450001	45500000	0.398048	*U6*
11	12325001	12375000	0.397928	*EXOC6B*
19	50550001	50600000	0.397917	*CSNK1D*
7	19250001	19300000	0.397884	*ARRDC5*
7	19250001	19300000	0.397884	*PLIN3*
7	19250001	19300000	0.397884	*UHRF1*
11	11525001	11575000	0.39787	*SPR*
19	11325001	11375000	0.397835	*BRIP1*
4	12475001	12525000	0.397823	*PPP1R9A*
23	15650001	15700000	0.397823	*MED20*
23	15650001	15700000	0.397823	*USP49*
23	15650001	15700000	0.397823	*BYSL*
23	15650001	15700000	0.397823	*CCND3*
27	33275001	33325000	0.397643	*ASH2L*
27	33275001	33325000	0.397643	*STAR*
19	9400001	9450000	0.397628	*HSF5*
19	9400001	9450000	0.397628	*MTMR4*
2	17675001	17725000	0.397622	*SESTD1*
2	17700001	17750000	0.397584	*SESTD1*
19	27650001	27700000	0.397582	*GUCY2D*
19	27650001	27700000	0.397582	*ALOX15B*
10	65300001	65350000	0.397475	*DUOX2*
10	65300001	65350000	0.397475	*SORD*
10	65300001	65350000	0.397475	*DUOXA1*
10	65300001	65350000	0.397475	*DUOXA2*
12	15075001	15125000	0.39742	*NUFIP1*
4	44775001	44825000	0.397414	*RELN*
18	24875001	24925000	0.397314	*NUP93*
18	24875001	24925000	0.397314	*SLC12A3*
18	24875001	24925000	0.397314	*MIR138-2*
22	49350001	49400000	0.397174	*DCAF1*
22	49350001	49400000	0.397174	*DOCK3*
22	49350001	49400000	0.397174	*MANF*
22	49350001	49400000	0.397174	*RBM15B*
19	51075001	51125000	0.397169	*SLC25A10*
19	51075001	51125000	0.397169	*MRPL12*
19	51075001	51125000	0.397169	*HGS*
4	76750001	76800000	0.396907	*OGDH*
29	47850001	47900000	0.39685	*SHANK2*
16	43725001	43775000	0.396844	*CLSTN1*
3	119175001	119225000	0.396836	*OR9S35P*
11	98600001	98650000	0.396812	*FAM102A*
11	98600001	98650000	0.396812	*DPM2*
14	1350001	1400000	0.396732	*LY6H*
14	1350001	1400000	0.396732	*GPIHBP1*
3	97875001	97925000	0.396729	*SPATA6*
15	39975001	40025000	0.396503	*TEAD1*
28	27700001	27750000	0.396471	*CDH23*
11	7425001	7475000	0.396452	*TMEM182*

11	7425001	7475000	0.396452	*MFSD9*
8	29025001	29075000	0.396428	*TTC39B*
19	47850001	47900000	0.396425	*DCAF7*
19	47850001	47900000	0.396425	*KCNH6*
1	152800001	152850000	0.396377	*ANKRD28*
10	65275001	65325000	0.396374	*DUOX1*
10	65275001	65325000	0.396374	*DUOX2*
10	65275001	65325000	0.396374	*DUOXA1*
10	65275001	65325000	0.396374	*DUOXA2*
26	4775001	4825000	0.396319	*PCDH15*
3	31550001	31600000	0.396198	*INKA2*
3	31550001	31600000	0.396198	*RAP1A*
3	31550001	31600000	0.396198	*DDX20*
7	14400001	14450000	0.396094	*PIN1*
7	14400001	14450000	0.396094	*FBXL12*
7	14400001	14450000	0.396094	*OLFM2*
7	14400001	14450000	0.396094	*UBL5*
20	5575001	5625000	0.395961	*CPEB4*
13	17375001	17425000	0.395892	*RBM17*
1	2350001	2400000	0.395794	*IFNAR2*
19	26250001	26300000	0.395753	*SCIMP*
10	75425001	75475000	0.395748	*RHOJ*
14	33775001	33825000	0.395738	*PRDM14*
19	50775001	50825000	0.395713	*FASN*
19	50775001	50825000	0.395713	*GPS1*
19	50775001	50825000	0.395713	*DUS1L*
19	50775001	50825000	0.395713	*RFNG*
19	27750001	27800000	0.395664	*ALOXE3*
19	27750001	27800000	0.395664	*PER1*
19	27750001	27800000	0.395664	*VAMP2*
19	27750001	27800000	0.395664	*HES7*
19	27750001	27800000	0.395664	*U6*
22	45925001	45975000	0.395531	*CACNA2D3*
26	25950001	26000000	0.395493	*SORCS3*
20	30025001	30075000	0.395488	*MRPS30*
12	31275001	31325000	0.395395	*MTUS2*
2	19000001	19050000	0.395194	*PDE11A*
19	38825001	38875000	0.395069	*NPEPPS*
19	38825001	38875000	0.395069	*U6*
1	70625001	70675000	0.394995	*TFRC*
1	70625001	70675000	0.394995	*TNK2*
3	106050001	106100000	0.394987	*CAP1*
29	50375001	50425000	0.394977	*AP2A2*
20	3025001	3075000	0.394934	*RANBP17*
2	27225001	27275000	0.394876	*SPC25*
2	27225001	27275000	0.394876	*G6PC2*
2	27225001	27275000	0.394876	*NOSTRIN*
2	27225001	27275000	0.394876	*U6*
3	106025001	106075000	0.394863	*CAP1*
3	106025001	106075000	0.394863	*PPT1*
19	28925001	28975000	0.394852	*USP43*
19	28925001	28975000	0.394852	*DHRS7C*

22	46250001	46300000	0.394837	*CACNA2D3*
14	38800001	38850000	0.39477	*HNF4G*
8	72550001	72600000	0.39476	*DOCK5*
14	31025001	31075000	0.394695	*SGK3*
14	31025001	31075000	0.394695	*MCMDC2*
2	23325001	23375000	0.394671	*CDCA7*
22	34025001	34075000	0.394668	*SUCLG2*
16	32400001	32450000	0.39465	*HNRNPU*
16	32400001	32450000	0.39465	*COX20*
8	65025001	65075000	0.394631	*ERP44*
7	11275001	11325000	0.394615	*PKN1*
7	11275001	11325000	0.394615	*GIPC1*
7	11275001	11325000	0.394615	*PTGER1*
22	32475001	32525000	0.394597	*TMF1*
22	32475001	32525000	0.394597	*UBA3*
2	125075001	125125000	0.394422	*SESN2*
19	19150001	19200000	0.394352	*KSR1*
1	1750001	1800000	0.394339	*ITSN1*
3	119125001	119175000	0.394322	*OR9S43*
3	119125001	119175000	0.394322	*OR9S43B*
20	69325001	69375000	0.394292	*IRX1*
28	28950001	29000000	0.394121	*PLA2G12B*
11	11625001	11675000	0.394108	*EXOC6B*
29	12750001	12800000	0.394058	*PRCP*
8	80100001	80150000	0.394048	*GAS1*
4	80700001	80750000	0.394031	*SUGCT*
7	90575001	90625000	0.394003	*ADGRV1*
8	83400001	83450000	0.39395	*ZNF782*
13	22775001	22825000	0.393864	*MLLT10*
18	10825001	10875000	0.393861	*KLHL36*
18	10825001	10875000	0.393861	*USP10*
3	450001	500000	0.39386	*TIPRL*
22	53550001	53600000	0.393826	*SACM1L*
22	53550001	53600000	0.393826	*SLC6A20*
7	90450001	90500000	0.393709	*ADGRV1*
21	28100001	28150000	0.393707	*FAM189A1*
22	43075001	43125000	0.393642	*DNASE1L3*
22	43075001	43125000	0.393642	*ABHD6*
14	39800001	39850000	0.393589	*ZFHX4*
6	104275001	104325000	0.393582	*MSX1*
1	70650001	70700000	0.393503	*TFRC*
21	40150001	40200000	0.393497	*PRKD1*
7	11825001	11875000	0.393354	*ZSWIM4*
7	11825001	11875000	0.393354	*C7H19orf53*
19	42275001	42325000	0.393338	*KCNH4*
19	42275001	42325000	0.393338	*STAT5B*
19	42275001	42325000	0.393338	*RAB5C*
19	42275001	42325000	0.393338	*GHDC*
19	42275001	42325000	0.393338	*HCRT*
21	67650001	67700000	0.393306	*CDC42BPB*
29	28125001	28175000	0.393211	*SIAE*
29	28125001	28175000	0.393211	*SPA17*

8	72600001	72650000	0.393178	*DOCK5*
22	43025001	43075000	0.393167	*ABHD6*
22	43025001	43075000	0.393167	*U6*
14	32425001	32475000	0.39304	*C14H8orf34*
28	15650001	15700000	0.393004	*ANK3*
3	48250001	48300000	0.393	*TLCD4*
3	48250001	48300000	0.393	*RWDD3*
21	45200001	45250000	0.392905	*BAZ1A*
4	67950001	68000000	0.39282	*JAZF1*
22	44175001	44225000	0.392754	*ARHGEF3*
20	14775001	14825000	0.39274	*SHISAL2B*
20	31775001	31825000	0.392724	*CCDC152*
16	41975001	42025000	0.39269	*DRAXIN*
16	41975001	42025000	0.39269	*FBXO6*
16	41975001	42025000	0.39269	*MAD2L2*
16	41975001	42025000	0.39269	*FBXO44*
19	28950001	29000000	0.392688	*GLP2R*
19	28950001	29000000	0.392688	*DHRS7C*
16	71950001	72000000	0.392659	*TRAF5*
16	71950001	72000000	0.392659	*RD3*
11	11475001	11525000	0.392629	*SFXN5*
11	11475001	11525000	0.392629	*EMX1*
7	14525001	14575000	0.392609	*COL5A3*
19	39050001	39100000	0.392605	*ARHGAP23*
3	120275001	120325000	0.392571	*SNED1*
28	27725001	27775000	0.392514	*CDH23*
22	33825001	33875000	0.392506	*SUCLG2*
28	15700001	15750000	0.392499	*ANK3*
3	103275001	103325000	0.392366	*SLC2A1*
23	16625001	16675000	0.392221	*PPP2R5D*
23	16625001	16675000	0.392221	*CUL7*
23	16625001	16675000	0.392221	*KLHDC3*
23	16625001	16675000	0.392221	*RRP36*
23	16625001	16675000	0.392221	*MEA1*
23	16625001	16675000	0.392221	*PEX6*
29	28375001	28425000	0.392189	*HEPACAM*
29	28375001	28425000	0.392189	*CCDC15*
19	29000001	29050000	0.392177	*GLP2R*
19	29000001	29050000	0.392177	*RCVRN*
19	29000001	29050000	0.392177	*GAS7*
14	36625001	36675000	0.392159	*RDH10*
14	36625001	36675000	0.392159	*RPL7*
8	39300001	39350000	0.392154	*PLGRKT*
19	25375001	25425000	0.392086	*PITPNM3*
19	25375001	25425000	0.392086	*PIMREG*
19	25375001	25425000	0.392086	*AIPL1*
22	32500001	32550000	0.392035	*TMF1*
22	32500001	32550000	0.392035	*EOGT*
3	56925001	56975000	0.392021	*HS2ST1*
20	31875001	31925000	0.392008	*GHR*
2	89100001	89150000	0.392007	*AOX1*
7	21800001	21850000	0.392	*RAD50*

22	60350001	60400000	0.391987	*CHCHD6*
22	7250001	7300000	0.391983	*TRIM71*
4	69650001	69700000	0.391969	*SNX10*
11	101300001	101350000	0.391963	*NUP214*
11	101300001	101350000	0.391963	*AIF1L*
28	26150001	26200000	0.391865	*COL13A1*
29	24500001	24550000	0.391835	*PRMT3*
16	50775001	50825000	0.39172	*CFAP74*
24	2600001	2650000	0.39169	*ZNF516*
28	28600001	28650000	0.391677	*MICU1*
4	51900001	51950000	0.391548	*CAV2*
4	51900001	51950000	0.391548	*CAV1*
15	16825001	16875000	0.39152	*GUCY1A2*
11	103950001	104000000	0.391471	*NOTCH1*
4	12400001	12450000	0.391384	*PPP1R9A*
2	44325001	44375000	0.391351	*CACNB4*
2	44325001	44375000	0.391351	*ARL5A*
8	70675001	70725000	0.391209	*SLC25A37*
11	100700001	100750000	0.391201	*HMCN2*
18	24850001	24900000	0.391198	*NUP93*
24	2350001	2400000	0.391098	*ZNF236*
11	83175001	83225000	0.391097	*NBAS*
20	20425001	20475000	0.391055	*RAB3C*
15	62850001	62900000	0.391043	*RCN1*
8	61450001	61500000	0.391042	*ZCCHC7*
3	120475001	120525000	0.391029	*SEPTIN2*
3	120475001	120525000	0.391029	*FARP2*
3	120475001	120525000	0.391029	*HDLBP*
19	50025001	50075000	0.390995	*FOXK2*
16	38625001	38675000	0.390916	*FMO3*
20	14350001	14400000	0.39082	*U4*
19	43975001	44025000	0.390798	*HROB*
19	43975001	44025000	0.390798	*ASB16*
19	43975001	44025000	0.390798	*ATXN7L3*
19	43975001	44025000	0.390798	*TMUB2*
19	43975001	44025000	0.390798	*UBTF*
20	35450001	35500000	0.390773	*RICTOR*
20	3075001	3125000	0.390721	*RANBP17*
3	36150001	36200000	0.39069	*NTNG1*
16	43700001	43750000	0.39069	*CLSTN1*
13	40150001	40200000	0.390676	*RALGAPA2*
4	32100001	32150000	0.390645	*TRA2A*
16	51150001	51200000	0.390594	*DVL1*
16	51150001	51200000	0.390594	*MXRA8*
16	51150001	51200000	0.390594	*TAS1R3*
16	51150001	51200000	0.390594	*CPTP*
16	51150001	51200000	0.390594	*AURKAIP1*
5	112575001	112625000	0.390553	*ACO2*
5	112575001	112625000	0.390553	*POLR3H*
5	58075001	58125000	0.390507	*OR6C278*
5	58075001	58125000	0.390507	*OR6C207*
18	11100001	11150000	0.390496	*KIAA0513*

18	11100001	11150000	0.390496	*ZDHHC7*
2	125375001	125425000	0.390455	*SMPDL3B*
2	125375001	125425000	0.390455	*EYA3*
2	125375001	125425000	0.390455	*XKR8*
25	25100001	25150000	0.390452	*KATNIP*
5	81825001	81875000	0.39043	*PTHLH*
13	22750001	22800000	0.390409	*MLLT10*
3	101375001	101425000	0.390392	*RNF220*
3	101375001	101425000	0.390392	*TMEM53*
14	925001	975000	0.390378	*EPPK1*
14	925001	975000	0.390378	*NRBP2*
14	925001	975000	0.390378	*PUF60*
4	68000001	68050000	0.390358	*JAZF1*
15	40350001	40400000	0.390293	*MICAL2*
16	45575001	45625000	0.390261	*CAMTA1*
10	70900001	70950000	0.390237	*DACT1*
11	11600001	11650000	0.390159	*EXOC6B*
22	37150001	37200000	0.390097	*PRICKLE2*
19	28675001	28725000	0.39001	*STX8*
12	32675001	32725000	0.38997	*USP12*
22	47975001	48025000	0.389937	*TMEM110*
22	47975001	48025000	0.389937	*SFMBT1*
18	18575001	18625000	0.389913	*TENT4B*
4	80650001	80700000	0.389853	*SUGCT*
26	45500001	45550000	0.38984	*ADAM12*
26	45500001	45550000	0.38984	*FANK1*
12	19375001	19425000	0.389822	*SPRYD7*
14	15050001	15100000	0.389737	*NSMCE2*
14	15050001	15100000	0.389737	*WASHC5*
8	7625001	7675000	0.389671	*GATA4*
8	7625001	7675000	0.389671	*NEIL2*
6	95975001	96025000	0.389587	*PRKG2*
6	30675001	30725000	0.389534	*ATOH1*
3	119225001	119275000	0.38946	*OR9S42*
1	145975001	146025000	0.389432	*PCNT*
1	145975001	146025000	0.389432	*C1H21orf58*
1	145975001	146025000	0.389432	*YBEY*
11	11775001	11825000	0.389399	*EXOC6B*
5	59000001	59050000	0.389385	*OR6C266*
5	59000001	59050000	0.389385	*OR6C74*
18	11450001	11500000	0.389359	*GSE1*
26	25300001	25350000	0.389352	*SORCS3*
14	14825001	14875000	0.389265	*NSMCE2*
3	25675001	25725000	0.389232	*MAN1A2*
8	64825001	64875000	0.389217	*NR4A3*
3	475001	525000	0.389202	*GPR161*
15	39950001	40000000	0.3892	*TEAD1*
5	105725001	105775000	0.389184	*TIGAR*
5	105725001	105775000	0.389184	*CCND2*
5	105725001	105775000	0.389184	*FGF23*
19	38350001	38400000	0.389181	*SKAP1*
19	38350001	38400000	0.389181	*SNX11*

19	38350001	38400000	0.389181	*CBX1*
28	25150001	25200000	0.389108	*DDX50*
28	25150001	25200000	0.389108	*STOX1*
1	93375001	93425000	0.389105	*NLGN1*
22	46575001	46625000	0.389031	*CACNA2D3*
2	107275001	107325000	0.388961	*DNPEP*
2	107275001	107325000	0.388961	*RESP18*
8	64850001	64900000	0.388942	*NR4A3*
4	10100001	10150000	0.388859	*CDK6*
11	104350001	104400000	0.388814	*CACFD1*
11	104350001	104400000	0.388814	*SLC2A6*
20	20450001	20500000	0.388807	*RAB3C*
25	36375001	36425000	0.388792	*ZKSCAN1*
25	36375001	36425000	0.388792	*ZNF3*
7	93775001	93825000	0.388698	*KIAA0825*
2	125400001	125450000	0.388654	*SMPDL3B*
2	125400001	125450000	0.388654	*RPA2*
2	125400001	125450000	0.388654	*XKR8*
28	35425001	35475000	0.388587	*CL46*
28	8075001	8125000	0.388523	*TBCE*
28	8075001	8125000	0.388523	*GGPS1*
5	111600001	111650000	0.388517	*TNRC6B*
19	38200001	38250000	0.388504	*SKAP1*
14	1250001	1300000	0.388472	*TOP1MT*
14	1250001	1300000	0.388472	*RHPN1*
26	45450001	45500000	0.388375	*FANK1*
21	42075001	42125000	0.388372	*NUBPL*
28	28925001	28975000	0.388323	*PLA2G12B*
28	28925001	28975000	0.388323	*OIT3*
18	46550001	46600000	0.388261	*TYROBP*
18	46550001	46600000	0.388261	*APLP1*
18	46550001	46600000	0.388261	*KIRREL2*
18	46550001	46600000	0.388261	*NFKBID*
18	46550001	46600000	0.388261	*NPHS1*
18	46550001	46600000	0.388261	*HCST*
26	34150001	34200000	0.388168	*CASP7*
16	45600001	45650000	0.388146	*CAMTA1*
3	101900001	101950000	0.388127	*KLF17*
8	72575001	72625000	0.388105	*DOCK5*
5	104600001	104650000	0.388003	*ANO2*
8	62025001	62075000	0.387957	*SHB*
9	42625001	42675000	0.387945	*PDSS2*
23	13650001	13700000	0.387837	*DAAM2*
3	78375001	78425000	0.387559	*MIER1*
3	78375001	78425000	0.387559	*DNAI4*
28	25600001	25650000	0.387365	*HKDC1*
2	23400001	23450000	0.387319	*MAP3K20*
18	36825001	36875000	0.387261	*WWP2*
18	36825001	36875000	0.387261	*NOB1*
18	36825001	36875000	0.387261	*U6*
28	15450001	15500000	0.387236	*CCDC6*
21	45075001	45125000	0.38714	*BAZ1A*

21	45075001	45125000	0.38714	*CFL2*
22	49050001	49100000	0.387049	*IQCF6*
8	76725001	76775000	0.387038	*FRMD3*
19	11050001	11100000	0.386909	*MED13*
3	31525001	31575000	0.386874	*DDX20*
3	31525001	31575000	0.386874	*INKA2*
3	31525001	31575000	0.386874	*KCND3*
21	2725001	2775000	0.386859	*ATP10A*
13	17275001	17325000	0.386855	*PFKFB3*
22	48025001	48075000	0.386853	*TMEM110*
22	48025001	48075000	0.386853	*ITIH4*
22	48025001	48075000	0.386853	*MUSTN1*
22	53450001	53500000	0.386822	*LZTFL1*
22	53450001	53500000	0.386822	*SLC6A20*
11	11900001	11950000	0.386812	*EXOC6B*
1	152700001	152750000	0.386785	*ANKRD28*
14	39925001	39975000	0.386784	*ZFHX4*
7	11700001	11750000	0.386753	*RFX1*
7	11700001	11750000	0.386753	*DCAF15*
4	65600001	65650000	0.386749	*INMT*
4	65600001	65650000	0.386749	*CRHR2*
20	25375001	25425000	0.38673	*NDUFS4*
18	46450001	46500000	0.386686	*ARHGAP33*
18	46450001	46500000	0.386686	*PROSER3*
18	46450001	46500000	0.386686	*LIN37*
18	46450001	46500000	0.386686	*KMT2B*
18	46450001	46500000	0.386686	*IGFLR1*
18	46450001	46500000	0.386686	*HSPB6*
18	46450001	46500000	0.386686	*U2AF1L4*
18	46450001	46500000	0.386686	*PSENEN*
26	13200001	13250000	0.386626	*PPP1R3C*
5	58575001	58625000	0.386624	*OR6C7H*
5	58575001	58625000	0.386624	*OR6C1Q*
19	8300001	8350000	0.386546	*MSI2*
6	94850001	94900000	0.386513	*PRDM8*
22	30250001	30300000	0.386471	*FOXP1*
18	14500001	14550000	0.386446	*DPEP1*
18	14500001	14550000	0.386446	*CPNE7*
16	51050001	51100000	0.386422	*SSU72*
16	51050001	51100000	0.386422	*ATAD3A*
16	51050001	51100000	0.386422	*TMEM240*
3	89325001	89375000	0.38639	*FYB2*
12	32650001	32700000	0.386314	*USP12*
14	34825001	34875000	0.386228	*EYA1*
22	60375001	60425000	0.386156	*CHCHD6*
22	60375001	60425000	0.386156	*TXNRD3*
29	28425001	28475000	0.386125	*CCDC15*
16	2850001	2900000	0.386027	*RBBP5*
16	2850001	2900000	0.386027	*CNTN2*
16	2850001	2900000	0.386027	*TMEM81*
1	93850001	93900000	0.385992	*NLGN1*
24	2325001	2375000	0.385975	*ZNF236*

24	2325001	2375000	0.385975	*MBP*
11	48650001	48700000	0.38597	*IMMT*
11	48650001	48700000	0.38597	*MRPL35*
11	48650001	48700000	0.38597	*REEP1*
15	51775001	51825000	0.385891	*FOLR1*
15	51775001	51825000	0.385891	*FOLR3*
15	51775001	51825000	0.385891	*FOLR2*
19	28775001	28825000	0.385763	*STX8*
3	42350001	42400000	0.385756	*SLC30A7*
20	41975001	42025000	0.385654	*DROSHA*
20	41975001	42025000	0.385654	*C20H5orf22*
19	27375001	27425000	0.385622	*WRAP53*
19	27375001	27425000	0.385622	*TP53*
19	27375001	27425000	0.385622	*DNAH2*
19	27375001	27425000	0.385622	*EFNB3*
20	14475001	14525000	0.385592	*CWC27*
8	61550001	61600000	0.385553	*ZBTB5*
8	61550001	61600000	0.385553	*GRHPR*
8	61550001	61600000	0.385553	*U6*
21	15825001	15875000	0.385552	*SV2B*
8	72750001	72800000	0.385537	*KCTD9*
3	89300001	89350000	0.385503	*FYB2*
2	47300001	47350000	0.385481	*KIF5C*
18	55050001	55100000	0.385447	*TMEM143*
18	55050001	55100000	0.385447	*SYNGR4*
18	55050001	55100000	0.385447	*KDELR1*
18	55050001	55100000	0.385447	*GRIN2D*
18	46525001	46575000	0.385436	*NPHS1*
18	46525001	46575000	0.385436	*KIRREL2*
18	46525001	46575000	0.385436	*APLP1*
18	46525001	46575000	0.385436	*U6*
22	60300001	60350000	0.385413	*CHCHD6*
8	76400001	76450000	0.385376	*FRMD3*
7	7875001	7925000	0.385327	*NOTCH3*
7	7875001	7925000	0.385327	*BRD4*
7	7875001	7925000	0.385327	*EPHX3*
16	42000001	42050000	0.38528	*FBXO6*
16	42000001	42050000	0.38528	*FBXO2*
16	42000001	42050000	0.38528	*FBXO44*
16	42000001	42050000	0.38528	*MAD2L2*
3	119250001	119300000	0.385273	*OR9S23B*
3	119250001	119300000	0.385273	*OR9S42*
3	106375001	106425000	0.385207	*NT5C1A*
3	106375001	106425000	0.385207	*HPCAL4*
8	39450001	39500000	0.385195	*JAK2*
18	25450001	25500000	0.385131	*DOK4*
18	25450001	25500000	0.385131	*POLR2C*
18	25450001	25500000	0.385131	*COQ9*
18	25450001	25500000	0.385131	*CCDC102A*
2	43450001	43500000	0.385126	*PRPF40A*
2	43450001	43500000	0.385126	*ARL6IP6*
2	43450001	43500000	0.385126	*U6*

7	7650001	7700000	0.385075	*RASAL3*
7	7650001	7700000	0.385075	*PGLYRP2*
20	33675001	33725000	0.385067	*PRKAA1*
20	33675001	33725000	0.385067	*TTC33*
14	33525001	33575000	0.385028	*SLCO5A1*
7	7675001	7725000	0.385002	*WIZ*
7	7675001	7725000	0.385002	*RASAL3*
7	7675001	7725000	0.385002	*PGLYRP2*
8	64975001	65025000	0.384963	*STX17*
22	37175001	37225000	0.384864	*PRICKLE2*
3	101675001	101725000	0.384817	*ERI3*
6	65600001	65650000	0.38481	*GABRB1*
2	106725001	106775000	0.384802	*CYP27A1*
16	3375001	3425000	0.384786	*ELK4*
16	3375001	3425000	0.384786	*SLC45A3*
8	65050001	65100000	0.384749	*ERP44*
11	10700001	10750000	0.384748	*DGUOK*
11	10700001	10750000	0.384748	*ACTG2*
1	120900001	120950000	0.384724	*ZIC4*
1	120900001	120950000	0.384724	*ZIC1*
6	64600001	64650000	0.384616	*GABRG1*
1	6250001	6300000	0.384588	*GRIK1*
21	2900001	2950000	0.384511	*ATP10A*
21	2900001	2950000	0.384511	*U6*
22	42850001	42900000	0.384361	*KCTD6*
22	42850001	42900000	0.384361	*ACOX2*
19	51050001	51100000	0.384348	*GCGR*
19	51050001	51100000	0.384348	*MCRIP1*
19	51050001	51100000	0.384348	*PPP1R27*
6	87900001	87950000	0.384321	*SNORD42*
16	50375001	50425000	0.384236	*PLCH2*
8	76425001	76475000	0.384106	*FRMD3*
28	3675001	3725000	0.384071	*TTC13*
28	3675001	3725000	0.384071	*ARV1*
2	61750001	61800000	0.384063	*R3HDM1*
1	2725001	2775000	0.384042	*C1H21orf62*
16	32375001	32425000	0.384042	*HNRNPU*
16	32375001	32425000	0.384042	*COX20*
14	35075001	35125000	0.383967	*EYA1*
2	125750001	125800000	0.38394	*AHDC1*
1	145925001	145975000	0.383866	*MCM3AP*
1	145925001	145975000	0.383866	*YBEY*
15	36475001	36525000	0.383713	*SOX6*
22	49025001	49075000	0.383688	*IQCF2*
5	111450001	111500000	0.383633	*TNRC6B*
29	28800001	28850000	0.383573	*PKNOX2*
3	101925001	101975000	0.3835	*KLF17*
28	24825001	24875000	0.383469	*DNA2*
28	24825001	24875000	0.383469	*SLC25A16*
28	24825001	24875000	0.383469	*U6*
6	6250001	6300000	0.383391	*SYNPO2*
5	47200001	47250000	0.383366	*GRIP1*

16	1100001	1150000	0.38333	*FMOD*
4	81525001	81575000	0.383294	*POU6F2*
14	35925001	35975000	0.383273	*KCNB2*
3	98325001	98375000	0.383219	*TRABD2B*
22	50675001	50725000	0.383072	*RHOA*
22	50675001	50725000	0.383072	*AMT*
22	50675001	50725000	0.383072	*NICN1*
22	50675001	50725000	0.383072	*TCTA*
19	44475001	44525000	0.383002	*ADAM11*
19	44475001	44525000	0.383002	*DBF4B*
3	101275001	101325000	0.382958	*ARMH1*
3	101275001	101325000	0.382958	*U5*
19	32775001	32825000	0.382878	*TEKT3*
19	32775001	32825000	0.382878	*PMP22*
14	15025001	15075000	0.382839	*NSMCE2*
16	43550001	43600000	0.382734	*NMNAT1*
16	43550001	43600000	0.382734	*RBP7*
8	7000001	7050000	0.382677	*GLRA3*
22	49175001	49225000	0.382667	*RAD54L2*
11	82975001	83025000	0.382608	*NBAS*
11	82975001	83025000	0.382608	*PGGHG*
18	45550001	45600000	0.382578	*ZNF181*
18	45550001	45600000	0.382578	*ZNF599*
22	32450001	32500000	0.382565	*UBA3*
22	32450001	32500000	0.382565	*ARL6IP5*
22	32450001	32500000	0.382565	*TMF1*
29	49700001	49750000	0.382468	*IFITM10*
29	49700001	49750000	0.382468	*CTSD*
7	90025001	90075000	0.38239	*POLR3G*
7	90025001	90075000	0.38239	*MBLAC2*
6	2400001	2450000	0.382335	*NPY5R*
6	2400001	2450000	0.382335	*NPY1R*
8	62600001	62650000	0.382333	*TDRD7*
14	35100001	35150000	0.38232	*EYA1*
22	7125001	7175000	0.382283	*CNOT10*
15	78525001	78575000	0.382196	*OR5I1*
15	78525001	78575000	0.382196	*OR5W27*
14	69950001	70000000	0.382115	*RAD54B*
18	54100001	54150000	0.382043	*ZC3H4*
5	103800001	103850000	0.382036	*CHD4*
5	103800001	103850000	0.382036	*LPAR5*
5	103800001	103850000	0.382036	*NOP2*
5	103800001	103850000	0.382036	*SCARNA11*
2	17725001	17775000	0.381991	*SESTD1*
14	4500001	4550000	0.381919	*FAM135B*
6	30800001	30850000	0.381901	*GRID2*
19	11300001	11350000	0.381809	*BRIP1*
4	51850001	51900000	0.381804	*CAV1*
21	45125001	45175000	0.381706	*BAZ1A*
13	17725001	17775000	0.381693	*YME1L1*
13	17725001	17775000	0.381693	*MASTL*
3	120575001	120625000	0.381673	*FARP2*

3	120575001	120625000	0.381673	*STK25*
20	24250001	24300000	0.381567	*CSPG4B*
25	18800001	18850000	0.381556	*DNAH3*
10	75450001	75500000	0.381528	*RHOJ*
5	11300001	11350000	0.381504	*PPFIA2*
21	47625001	47675000	0.381461	*MIPOL1*
28	30825001	30875000	0.381413	*DUSP29*
28	30825001	30875000	0.381413	*DUSP13*
12	12675001	12725000	0.381317	*TNFSF11*
16	38650001	38700000	0.381213	*FMO3*
7	12625001	12675000	0.381211	*SYCE2*
7	12625001	12675000	0.381211	*FARSA*
7	12625001	12675000	0.381211	*GCDH*
7	12625001	12675000	0.381211	*KLF1*
22	30500001	30550000	0.381198	*FOXP1*
13	22725001	22775000	0.381177	*MLLT10*
13	22725001	22775000	0.381177	*SKIDA1*
13	17075001	17125000	0.381115	*PRKCQ*
14	850001	900000	0.38107	*PLEC*
22	33450001	33500000	0.38085	*TAFA1*
14	30950001	31000000	0.380846	*SGK3*
19	28075001	28125000	0.380832	*MYH10*
19	28075001	28125000	0.380832	*NDEL1*
14	31100001	31150000	0.380824	*PPP1R42*
14	31100001	31150000	0.380824	*MCMDC2*
14	31100001	31150000	0.380824	*TCF24*
14	31100001	31150000	0.380824	*SNORD87*
2	26600001	26650000	0.380819	*CFAP210*
2	26600001	26650000	0.380819	*KLHL23*
2	26600001	26650000	0.380819	*PHOSPHO2*
2	26600001	26650000	0.380819	*PPIG*
4	40575001	40625000	0.380782	*GNAT3*
4	10650001	10700000	0.380742	*VPS50*
9	42925001	42975000	0.380736	*MTRES1*
9	42925001	42975000	0.380736	*BEND3*
22	30350001	30400000	0.380688	*FOXP1*
4	119600001	119650000	0.380688	*VIPR2*
16	51075001	51125000	0.380686	*ATAD3A*
16	51075001	51125000	0.380686	*TMEM240*
16	51075001	51125000	0.380686	*VWA1*
16	51075001	51125000	0.380686	*TMEM88B*
16	51075001	51125000	0.380686	*SSU72*
28	33075001	33125000	0.380621	*KCNMA1*
7	21850001	21900000	0.380613	*RAD50*
7	21850001	21900000	0.380613	*IL5*
19	52425001	52475000	0.380551	*CARD14*
19	52425001	52475000	0.380551	*EIF4A3*
19	52425001	52475000	0.380551	*SGSH*
19	52425001	52475000	0.380551	*Metazoa_SRP*
2	107250001	107300000	0.38054	*PTPRN*
2	107250001	107300000	0.38054	*RESP18*
11	69325001	69375000	0.38045	*LCLAT1*

12	31250001	31300000	0.380404	*MTUS2*
23	15675001	15725000	0.380371	*MED20*
23	15675001	15725000	0.380371	*BYSL*
23	15675001	15725000	0.380371	*CCND3*
2	61675001	61725000	0.380362	*R3HDM1*
2	61675001	61725000	0.380362	*MIR128-1*
3	78325001	78375000	0.380349	*MIER1*
8	86550001	86600000	0.380311	*AUH*
25	25075001	25125000	0.380288	*KATNIP*
25	25075001	25125000	0.380288	*GTF3C1*
14	31750001	31800000	0.380207	*CPA6*
20	525001	575000	0.380198	*SLIT3*
20	525001	575000	0.380198	*MIR218-2*
8	62050001	62100000	0.380079	*SHB*
5	67525001	67575000	0.380071	*TTC41*
7	17750001	17800000	0.380049	*C3*
7	17750001	17800000	0.380049	*GPR108*
7	17750001	17800000	0.380049	*TRIP10*
22	10825001	10875000	0.380004	*GOLGA4*
7	12575001	12625000	0.37993	*RAD23A*
7	12575001	12625000	0.37993	*CALR*
7	12575001	12625000	0.37993	*GADD45GIP1*
7	12575001	12625000	0.37993	*NFIX*
1	93500001	93550000	0.379816	*NLGN1*
3	120425001	120475000	0.379635	*HDLBP*
1	2750001	2800000	0.379592	*C1H21orf62*
1	2750001	2800000	0.379592	*PAXBP1*
2	17650001	17700000	0.379448	*SESTD1*
3	25850001	25900000	0.379447	*MAN1A2*
3	20700001	20750000	0.379442	*SV2A*
3	20700001	20750000	0.379442	*H2AC20*
3	20700001	20750000	0.379442	*BOLA1*
3	20700001	20750000	0.379442	*H2AC19*
3	20700001	20750000	0.379442	*H2AC21*
5	112025001	112075000	0.379248	*SLC25A17*
5	112025001	112075000	0.379248	*U6*
24	50001	100000	0.379206	*OR5W32P*
22	53600001	53650000	0.379123	*SACM1L*
22	53600001	53650000	0.379123	*LIMD1*
6	91300001	91350000	0.379093	*SHROOM3*
19	56275001	56325000	0.379088	*JPT1*
19	56275001	56325000	0.379088	*SUMO2*
19	56275001	56325000	0.379088	*ARMC7*
19	56275001	56325000	0.379088	*NT5C*
2	43475001	43525000	0.379073	*PRPF40A*
4	119550001	119600000	0.379017	*DYNC2I1*
6	30700001	30750000	0.378957	*ATOH1*
7	93800001	93850000	0.378935	*KIAA0825*
19	51425001	51475000	0.378931	*SLC38A10*
15	47675001	47725000	0.378851	*OR52H9*
8	82600001	82650000	0.37881	*ERCC6L2*
1	156775001	156825000	0.378739	*KCNH8*

3	120700001	120750000	0.378688	*ATG4B*
3	120700001	120750000	0.378688	*THAP4*
3	120700001	120750000	0.378688	*DTYMK*
3	120700001	120750000	0.378688	*ING5*
22	6300001	6350000	0.378676	*OSBPL10*
6	96000001	96050000	0.378622	*PRKG2*
11	45725001	45775000	0.37862	*UXS1*
14	34750001	34800000	0.378598	*EYA1*
14	23050001	23100000	0.378411	*TGS1*
14	23050001	23100000	0.378411	*TMEM68*
14	39850001	39900000	0.378404	*ZFHX4*
7	21900001	21950000	0.378368	*IRF1*
3	103925001	103975000	0.3783	*FOXJ3*
6	114175001	114225000	0.378289	*AFAP1*
19	28975001	29025000	0.378286	*GLP2R*
11	14200001	14250000	0.378257	*XDH*
29	49975001	50025000	0.378252	*MOB2*
29	49975001	50025000	0.378252	*DUSP8*
8	64950001	65000000	0.378239	*STX17*
16	42025001	42075000	0.378201	*FBXO2*
16	42025001	42075000	0.378201	*FBXO44*
28	28125001	28175000	0.378176	*SPOCK2*
16	41550001	41600000	0.378132	*TNFRSF8*
7	12875001	12925000	0.378095	*ZNF791*
4	67975001	68025000	0.37804	*JAZF1*
19	51175001	51225000	0.378008	*NPLOC4*
19	51175001	51225000	0.378008	*FAAP100*
28	44600001	44650000	0.37781	*RASSF4*
28	44600001	44650000	0.37781	*TMEM72*
28	44600001	44650000	0.37781	*DEPP1*
28	25675001	25725000	0.37777	*HK1*
5	109175001	109225000	0.377753	*MICAL3*
2	43500001	43550000	0.377729	*PRPF40A*
2	43500001	43550000	0.377729	*FMNL2*
3	120400001	120450000	0.377703	*HDLBP*
16	4000001	4050000	0.377694	*SRGAP2*
27	35025001	35075000	0.377657	*IDO2*
27	35025001	35075000	0.377657	*IDO1*
14	825001	875000	0.377654	*PLEC*
14	825001	875000	0.377654	*PARP10*
14	825001	875000	0.377654	*GRINA*
23	16700001	16750000	0.377648	*PTK7*
23	16700001	16750000	0.377648	*KLC4*
2	18525001	18575000	0.377622	*OSBPL6*
1	146050001	146100000	0.377621	*PCNT*
19	50325001	50375000	0.377607	*SECTM1*
19	50325001	50375000	0.377607	*SECTM1A*
15	79300001	79350000	0.377565	*OR5AL8*
22	52600001	52650000	0.377558	*PTH1R*
22	52600001	52650000	0.377558	*MYL3*
15	40750001	40800000	0.377442	*USP47*
23	16825001	16875000	0.377438	*TTBK1*

14	32575001	32625000	0.377427	*C14H8orf34*
19	38325001	38375000	0.377415	*SKAP1*
8	20400001	20450000	0.377413	*ELAVL2*
14	14850001	14900000	0.37741	*NSMCE2*
3	120375001	120425000	0.377407	*PPP1R7*
3	120375001	120425000	0.377407	*HDLBP*
8	70800001	70850000	0.377378	*NKX2-6*
18	22325001	22375000	0.377327	*FTO*
15	51800001	51850000	0.377281	*INPPL1*
15	51800001	51850000	0.377281	*FOLR1*
15	51800001	51850000	0.377281	*PHOX2A*
15	51800001	51850000	0.377281	*FOLR2*
19	42225001	42275000	0.377236	*RAB5C*
19	42225001	42275000	0.377236	*DHX58*
19	42225001	42275000	0.377236	*ZNF385C*
19	42225001	42275000	0.377236	*KAT2A*
19	42225001	42275000	0.377236	*HSPB9*
7	89950001	90000000	0.377185	*CETN3*
6	89900001	89950000	0.377167	*PARM1*
29	24475001	24525000	0.377101	*PRMT3*
5	25700001	25750000	0.377051	*COPZ1*
5	25700001	25750000	0.377051	*ZNF385A*
5	25700001	25750000	0.377051	*GPR84*
22	43450001	43500000	0.377013	*SLMAP*
8	86975001	87025000	0.376988	*SYK*
13	30100001	30150000	0.376977	*ITGA8*
5	11325001	11375000	0.376958	*PPFIA2*
4	80975001	81025000	0.376954	*CDK13*
19	51550001	51600000	0.376919	*AATK*
19	51550001	51600000	0.376919	*BAIAP2*
2	22775001	22825000	0.376913	*SP3*
5	69700001	69750000	0.376899	*POLR3B*
5	69700001	69750000	0.376899	*TCP11L2*
3	78525001	78575000	0.376666	*DYNLT5*
3	51275001	51325000	0.376539	*BTBD8*
6	30325001	30375000	0.376449	*SMARCAD1*
28	29150001	29200000	0.376416	*P4HA1*
28	29150001	29200000	0.376416	*NUDT13*
6	8250001	8300000	0.376313	*TRAM1L1*
8	60250001	60300000	0.376275	*OR13C11*
8	60250001	60300000	0.376275	*OR13C7*
8	60250001	60300000	0.376275	*OR13C7L*
8	60250001	60300000	0.376275	*OR13C7B*
8	60250001	60300000	0.376275	*OR13C7C*
18	45525001	45575000	0.376211	*ZNF181*
18	45525001	45575000	0.376211	*ZNF599*
3	101825001	101875000	0.376201	*DMAP1*
3	101825001	101875000	0.376201	*ERI3*
1	45025001	45075000	0.376172	*TMEM45A*
20	3775001	3825000	0.376164	*FBXW11*
3	120500001	120550000	0.376096	*FARP2*
3	120500001	120550000	0.376096	*SEPTIN2*

12	15350001	15400000	0.376072	*GTF2F2*
22	44075001	44125000	0.375989	*ARHGEF3*
28	25650001	25700000	0.375925	*HK1*
14	69475001	69525000	0.375878	*INTS8*
10	75550001	75600000	0.375878	*RHOJ*
10	75550001	75600000	0.375878	*GPHB5*
22	30525001	30575000	0.375876	*FOXP1*
7	90050001	90100000	0.375835	*POLR3G*
7	90050001	90100000	0.375835	*LYSMD3*
7	14700001	14750000	0.375814	*DNMT1*
7	14700001	14750000	0.375814	*S1PR2*
12	32100001	32150000	0.375806	*PDX1*
5	78225001	78275000	0.375779	*AMN1*
5	78225001	78275000	0.375779	*ETFBKMT*
8	82700001	82750000	0.375739	*ERCC6L2*
28	26125001	26175000	0.375709	*COL13A1*
3	106075001	106125000	0.375706	*MFSD2A*
1	1600001	1650000	0.375679	*ATP5PO*
19	43525001	43575000	0.375653	*MEOX1*
28	17675001	17725000	0.375543	*CABCOCO1*
3	15975001	16025000	0.375538	*ADAR*
3	15975001	16025000	0.375538	*CHRNB2*
3	15975001	16025000	0.375538	*UBE2Q1*
28	28700001	28750000	0.375504	*MCU*
18	11850001	11900000	0.3755	*IRF8*
22	48425001	48475000	0.375482	*SEMA3G*
22	48425001	48475000	0.375482	*PHF7*
22	48425001	48475000	0.375482	*NISCH*
22	48425001	48475000	0.375482	*TNNC1*
18	21950001	22000000	0.375469	*RPGRIP1L*
11	55600001	55650000	0.375455	*CTNNA2*
5	113000001	113050000	0.375427	*WBP2NL*
5	113000001	113050000	0.375427	*NAGA*
5	113000001	113050000	0.375427	*PHETA2*
5	113000001	113050000	0.375427	*NDUFA6*
5	113000001	113050000	0.375427	*SMDT1*
19	49975001	50025000	0.375383	*WDR45B*
19	49975001	50025000	0.375383	*RAB40B*
21	67625001	67675000	0.375361	*CDC42BPB*
2	125125001	125175000	0.375332	*DNAJC8*
2	125125001	125175000	0.375332	*SESN2*
2	125125001	125175000	0.375332	*ATP5IF1*
8	70775001	70825000	0.375301	*NKX3-1*
8	70775001	70825000	0.375301	*NKX2-6*
20	25425001	25475000	0.375271	*NDUFS4*
3	103950001	104000000	0.375245	*FOXJ3*
8	72625001	72675000	0.375136	*DOCK5*
5	109150001	109200000	0.375136	*MICAL3*
5	109150001	109200000	0.375136	*BID*
19	29025001	29075000	0.374992	*GAS7*
19	29025001	29075000	0.374992	*RCVRN*
18	15650001	15700000	0.374942	*ITFG1*

7	53775001	53825000	0.374938	*ARHGAP26*
24	34425001	34475000	0.374902	*MIB1*
24	34425001	34475000	0.374902	*MIR1-2*
18	10700001	10750000	0.374892	*MEAK7*
5	67475001	67525000	0.374889	*NT5DC3*
5	67475001	67525000	0.374889	*TTC41*
18	52925001	52975000	0.374875	*MARK4*
18	52925001	52975000	0.374875	*CKM*
18	52925001	52975000	0.374875	*KLC3*
18	52925001	52975000	0.374875	*U6*
1	146025001	146075000	0.374867	*PCNT*
11	14150001	14200000	0.374867	*XDH*
11	14150001	14200000	0.374867	*FAM136A*
19	51775001	51825000	0.374866	*RPTOR*
29	28175001	28225000	0.374853	*NRGN*
19	47825001	47875000	0.374844	*KCNH6*
19	47825001	47875000	0.374844	*DCAF7*
12	19450001	19500000	0.374752	*TRIM13*
12	19450001	19500000	0.374752	*KCNRG*
20	35425001	35475000	0.374737	*RICTOR*
6	30825001	30875000	0.374724	*GRID2*
14	75350001	75400000	0.374691	*CNBD1*
8	82575001	82625000	0.374674	*ERCC6L2*
20	15200001	15250000	0.374672	*RNF180*
10	30950001	31000000	0.37462	*DPH6*
23	10750001	10800000	0.374605	*PPIL1*
23	10750001	10800000	0.374605	*CPNE5*
23	10750001	10800000	0.374605	*C23H6orf89*
14	23125001	23175000	0.374554	*LYN*
1	44950001	45000000	0.374532	*TMEM45A*
19	51750001	51800000	0.374484	*RPTOR*
19	30675001	30725000	0.374483	*MAP2K4*
14	4450001	4500000	0.374451	*FAM135B*
13	73050001	73100000	0.374434	*ADA*
19	52600001	52650000	0.374422	*TBC1D16*
16	50400001	50450000	0.374377	*PLCH2*
16	50400001	50450000	0.374377	*RER1*
16	50400001	50450000	0.374377	*PEX10*
13	1550001	1600000	0.374376	*PLCB1*
22	48775001	48825000	0.37436	*POC1A*
13	61800001	61850000	0.374357	*ASXL1*
4	81550001	81600000	0.374227	*POU6F2*
18	52950001	53000000	0.374224	*ERCC2*
18	52950001	53000000	0.374224	*CKM*
18	52950001	53000000	0.374224	*KLC3*
18	52950001	53000000	0.374224	*MARK4*
27	33950001	34000000	0.374215	*TACC1*
21	45100001	45150000	0.374201	*BAZ1A*
21	45100001	45150000	0.374201	*CFL2*
1	152425001	152475000	0.374164	*METTL6*
1	152425001	152475000	0.374164	*EAF1*
29	5275001	5325000	0.374123	*U6*

13	22800001	22850000	0.374091	*MLLT10*
19	11275001	11325000	0.374084	*BRIP1*
12	31300001	31350000	0.37406	*MTUS2*
19	9375001	9425000	0.374044	*HSF5*
19	9375001	9425000	0.374044	*RNF43*
22	46000001	46050000	0.374031	*CACNA2D3*
7	43950001	44000000	0.374031	*TCF3*
7	43950001	44000000	0.374031	*MBD3*
7	43950001	44000000	0.374031	*MEX3D*
7	43950001	44000000	0.374031	*UQCR11*
6	35800001	35850000	0.373972	*FAM13A*
6	67650001	67700000	0.373944	*CWH43*
22	42900001	42950000	0.373913	*PDHB*
22	42900001	42950000	0.373913	*PXK*
22	42900001	42950000	0.373913	*KCTD6*
14	35950001	36000000	0.373889	*KCNB2*
7	43725001	43775000	0.373882	*EFNA2*
28	26225001	26275000	0.373872	*COL13A1*
19	19075001	19125000	0.373855	*KSR1*
5	111625001	111675000	0.373818	*TNRC6B*
5	111625001	111675000	0.373818	*ADSL*
20	48875001	48925000	0.373799	*CDH10*
28	8225001	8275000	0.373732	*B3GALNT2*
15	74950001	75000000	0.373681	*TSPAN18*
19	44575001	44625000	0.373666	*EFTUD2*
19	44575001	44625000	0.373666	*GFAP*
19	44575001	44625000	0.373666	*CCDC103*
19	44575001	44625000	0.373666	*FAM187A*
18	2675001	2725000	0.373628	*CFDP1*
19	12225001	12275000	0.373596	*BCAS3*
6	19050001	19100000	0.373584	*TBCK*
22	49825001	49875000	0.373538	*CACNA2D2*
26	36475001	36525000	0.373516	*GFRA1*
1	93475001	93525000	0.373437	*NLGN1*
16	34000001	34050000	0.373399	*CEP170*
3	106425001	106475000	0.373354	*HEYL*
16	73125001	73175000	0.373209	*SYT14*
19	30700001	30750000	0.373194	*MAP2K4*
23	8325001	8375000	0.373194	*NUDT3*
23	8325001	8375000	0.373194	*SMIM29*
8	7550001	7600000	0.37319	*CTSB*
8	7550001	7600000	0.37319	*FDFT1*
4	119400001	119450000	0.37313	*ESYT2*
4	119400001	119450000	0.37313	*NCAPG2*
22	49200001	49250000	0.373069	*RAD54L2*
19	41075001	41125000	0.373006	*KRT23*
19	41075001	41125000	0.373006	*KRT20*
16	49400001	49450000	0.372988	*MEGF6*
16	49400001	49450000	0.372988	*WRAP73*
16	49400001	49450000	0.372988	*TP73*
16	49400001	49450000	0.372988	*TPRG1L*
7	93825001	93875000	0.372968	*KIAA0825*

16	3500001	3550000	0.372965	*SLC41A1*
16	3500001	3550000	0.372965	*RAB29*
22	43000001	43050000	0.372961	*ABHD6*
22	43000001	43050000	0.372961	*RPP14*
22	43000001	43050000	0.372961	*PXK*
22	43000001	43050000	0.372961	*U6*
16	39925001	39975000	0.372932	*C16H1orf105*
13	17100001	17150000	0.372876	*PRKCQ*
28	8150001	8200000	0.372855	*TBCE*
28	8150001	8200000	0.372855	*B3GALNT2*
13	57075001	57125000	0.372833	*EDN3*
14	39825001	39875000	0.372722	*ZFHX4*
2	65475001	65525000	0.372718	*ACTR3*
14	1375001	1425000	0.372662	*LY6E*
4	51925001	51975000	0.372632	*CAV2*
2	61725001	61775000	0.372496	*R3HDM1*
11	104475001	104525000	0.372454	*DBH*
11	104475001	104525000	0.372454	*FAM163B*
11	104475001	104525000	0.372454	*SARDH*
22	52525001	52575000	0.372451	*CCDC12*
20	38450001	38500000	0.372364	*SPEF2*
28	26725001	26775000	0.372306	*PALD1*
11	104500001	104550000	0.372278	*SARDH*
11	104500001	104550000	0.372278	*DBH*
3	49700001	49750000	0.372261	*BCAR3*
1	2200001	2250000	0.372257	*IFNAR1*
1	2200001	2250000	0.372257	*IL10RB*
15	62425001	62475000	0.372247	*ELP4*
19	44000001	44050000	0.372235	*UBTF*
19	44000001	44050000	0.372235	*ATXN7L3*
19	44000001	44050000	0.372235	*TMUB2*
4	73075001	73125000	0.372227	*U6*
1	6225001	6275000	0.372191	*GRIK1*
22	300001	350000	0.372165	*UBE2D4*
18	12375001	12425000	0.372143	*FOXC2*
18	12375001	12425000	0.372143	*FOXL1*
23	48000001	48050000	0.372124	*SSR1*
23	48000001	48050000	0.372124	*RREB1*
28	8125001	8175000	0.372112	*TBCE*
24	325001	375000	0.372076	*PARD6G*
3	79825001	79875000	0.372067	*LEPR*
23	16800001	16850000	0.372059	*CUL9*
23	16800001	16850000	0.372059	*DNPH1*
5	47275001	47325000	0.372043	*GRIP1*
14	56375001	56425000	0.37199	*RSPO2*
19	38225001	38275000	0.371982	*SKAP1*
14	24600001	24650000	0.371972	*UBXN2B*
15	38500001	38550000	0.371946	*SPON1*
13	39225001	39275000	0.371945	*SLC24A3*
29	48250001	48300000	0.371871	*DHCR7*
16	49375001	49425000	0.371827	*TP73*
16	49375001	49425000	0.371827	*WRAP73*

16	49375001	49425000	0.371827	*TPRG1L*
8	6975001	7025000	0.371794	*GLRA3*
8	62075001	62125000	0.371757	*SHB*
13	40175001	40225000	0.371717	*RALGAPA2*
5	75625001	75675000	0.371714	*RAC2*
5	75625001	75675000	0.371714	*C1QTNF6*
5	75625001	75675000	0.371714	*SSTR3*
1	93825001	93875000	0.371668	*NLGN1*
2	43625001	43675000	0.371664	*FMNL2*
14	1275001	1325000	0.371663	*TOP1MT*
14	1275001	1325000	0.371663	*ZNF696*
14	1275001	1325000	0.371663	*GLI4*
22	6900001	6950000	0.371645	*CMTM6*
2	44350001	44400000	0.371644	*ARL5A*
6	94700001	94750000	0.371638	*ANTXR2*
8	76250001	76300000	0.371574	*RASEF*
8	76450001	76500000	0.371571	*FRMD3*
3	102000001	102050000	0.371564	*SLC6A9*
3	16425001	16475000	0.371531	*NUP210L*
3	16425001	16475000	0.371531	*CREB3L4*
3	16425001	16475000	0.371531	*JTB*
3	16425001	16475000	0.371531	*RAB13*
3	16425001	16475000	0.371531	*SLC39A1*
3	16425001	16475000	0.371531	*RPS27*
5	10250001	10300000	0.371512	*MYF5*
5	10250001	10300000	0.371512	*MYF6*
5	10250001	10300000	0.371512	*PTPRQ*
19	11125001	11175000	0.371464	*MED13*
19	11125001	11175000	0.371464	*INTS2*
15	62075001	62125000	0.371454	*DCDC1*
14	4575001	4625000	0.371436	*FAM135B*
5	110600001	110650000	0.37138	*CBX7*
3	35975001	36025000	0.37136	*NTNG1*
22	38400001	38450000	0.371358	*CADPS*
6	8225001	8275000	0.371288	*TRAM1L1*
29	49250001	49300000	0.371277	*TSPAN32*
19	22825001	22875000	0.371256	*SMYD4*
19	22825001	22875000	0.371256	*SERPINF1*
19	22825001	22875000	0.371256	*SERPINF2*
19	22825001	22875000	0.371256	*WDR81*
5	53650001	53700000	0.371188	*SLC16A7*
28	26675001	26725000	0.371174	*PALD1*
16	6700001	6750000	0.371133	*KCNT2*
3	35650001	35700000	0.371131	*VAV3*
19	30725001	30775000	0.371124	*MAP2K4*
3	104000001	104050000	0.3711	*FOXJ3*
3	104000001	104050000	0.3711	*GUCA2B*
3	104000001	104050000	0.3711	*GUCA2A*
10	27400001	27450000	0.371006	*OR4K14*
21	45875001	45925000	0.370991	*RALGAPA1*
5	67550001	67600000	0.370975	*TTC41*
16	45625001	45675000	0.37095	*CAMTA1*

22	54950001	55000000	0.370904	*SLC6A11*
22	41025001	41075000	0.37088	*FHIT*
8	76650001	76700000	0.370862	*FRMD3*
2	89125001	89175000	0.37086	*AOX1*
3	101700001	101750000	0.370813	*ERI3*
20	65800001	65850000	0.370758	*ADCY2*
2	106250001	106300000	0.370695	*ARPC2*
2	106250001	106300000	0.370695	*AAMP*
2	106250001	106300000	0.370695	*GPBAR1*
2	106250001	106300000	0.370695	*PNKD*
22	45125001	45175000	0.370687	*ERC2*
16	43475001	43525000	0.370676	*UBE4B*
7	11300001	11350000	0.370646	*PKN1*
7	11300001	11350000	0.370646	*DDX39A*
7	11300001	11350000	0.370646	*PTGER1*
3	117650001	117700000	0.370565	*TRAF3IP1*
18	10550001	10600000	0.370529	*WFDC1*
16	50750001	50800000	0.3705	*CFAP74*
15	57400001	57450000	0.370495	*SLC5A12*
18	15525001	15575000	0.370487	*NETO2*
18	15525001	15575000	0.370487	*ITFG1*
6	91050001	91100000	0.370453	*SCARB2*
12	61150001	61200000	0.370396	*HTATSF1*
28	33125001	33175000	0.370392	*KCNMA1*
22	45300001	45350000	0.370335	*ERC2*
28	25175001	25225000	0.370322	*DDX50*
28	25175001	25225000	0.370322	*DDX21*
14	39875001	39925000	0.370316	*ZFHX4*
14	35000001	35050000	0.370304	*EYA1*
7	12550001	12600000	0.370287	*NFIX*
2	61775001	61825000	0.370283	*R3HDM1*
2	61775001	61825000	0.370283	*U6*
21	45950001	46000000	0.370187	*RALGAPA1*
21	45950001	46000000	0.370187	*U6*
16	33125001	33175000	0.370141	*ZBTB18*
22	43525001	43575000	0.370129	*DENND6A*
6	30300001	30350000	0.37008	*SMARCAD1*
6	30300001	30350000	0.37008	*HPGDS*
11	11850001	11900000	0.370036	*EXOC6B*
19	26875001	26925000	0.369986	*DLG4*
19	26875001	26925000	0.369986	*ASGR1*
19	26875001	26925000	0.369986	*ASGR2*
16	4550001	4600000	0.369984	*IL10*
16	38750001	38800000	0.369942	*FMO2*
18	25425001	25475000	0.369928	*CIAPIN1*
18	25425001	25475000	0.369928	*COQ9*
18	25425001	25475000	0.369928	*DOK4*
18	25425001	25475000	0.369928	*POLR2C*
29	28050001	28100000	0.369927	*PANX3*
29	28050001	28100000	0.369927	*OR8A1C*
14	23150001	23200000	0.369884	*LYN*
11	44900001	44950000	0.36985	*SULT1C3*

5	47225001	47275000	0.369808	*GRIP1*
20	25475001	25525000	0.369675	*NDUFS4*
23	11050001	11100000	0.369656	*PIM1*
19	28050001	28100000	0.369641	*NDEL1*
19	28050001	28100000	0.369641	*MYH10*
6	111800001	111850000	0.369502	*LDB2*
16	32425001	32475000	0.369485	*COX20*
19	20150001	20200000	0.369394	*FAM222B*
19	20150001	20200000	0.369394	*NEK8*
19	20150001	20200000	0.369394	*TRAF4*
19	20150001	20200000	0.369394	*TLCD1*
24	2575001	2625000	0.369358	*ZNF516*
16	6375001	6425000	0.36934	*KCNT2*
24	54625001	54675000	0.369311	*TCF4*
24	54625001	54675000	0.369311	*U2*
2	125100001	125150000	0.369301	*SESN2*
2	125100001	125150000	0.369301	*ATP5IF1*
17	6775001	6825000	0.369288	*LRBA*
3	49725001	49775000	0.369274	*BCAR3*
13	22875001	22925000	0.369269	*MLLT10*
5	17825001	17875000	0.36923	*CEP290*
5	17825001	17875000	0.36923	*C5H12orf29*
20	14325001	14375000	0.369209	*ADAMTS6*
11	98575001	98625000	0.369139	*FAM102A*
11	98575001	98625000	0.369139	*PIP5KL1*
11	98575001	98625000	0.369139	*ST6GALNAC4*
11	98575001	98625000	0.369139	*DPM2*
11	77900001	77950000	0.36912	*APOB*
3	51300001	51350000	0.36911	*BTBD8*
3	51300001	51350000	0.36911	*EPHX4*
4	80425001	80475000	0.369073	*SUGCT*
1	148700001	148750000	0.369058	*CLDN14*
1	42250001	42300000	0.369047	*GABRR3*
15	39800001	39850000	0.369011	*TEAD1*
8	65075001	65125000	0.368999	*ERP44*
15	52725001	52775000	0.368981	*P2RY2*
22	36725001	36775000	0.368922	*ADAMTS9*
28	17625001	17675000	0.36892	*CABCOCO1*
11	97475001	97525000	0.368873	*LMX1B*
1	146000001	146050000	0.368849	*PCNT*
22	60400001	60450000	0.368791	*TXNRD3*
22	60400001	60450000	0.368791	*C22H3orf22*
8	61825001	61875000	0.368758	*FRMPD1*
8	61825001	61875000	0.368758	*TRMT10B*
21	45500001	45550000	0.368729	*PRORP*
21	45500001	45550000	0.368729	*PSMA6*
23	37450001	37500000	0.36871	*E2F3*
10	88600001	88650000	0.368699	*VIPAS39*
10	88600001	88650000	0.368699	*NOXRED1*
10	88600001	88650000	0.368699	*AHSA1*
5	108800001	108850000	0.368689	*IL17RA*
5	108800001	108850000	0.368689	*HDHD5*

22	49325001	49375000	0.368676	*DCAF1*
22	49325001	49375000	0.368676	*RBM15B*
7	21825001	21875000	0.368662	*RAD50*
11	103675001	103725000	0.368643	*QSOX2*
11	103675001	103725000	0.368643	*LHX3*
18	36800001	36850000	0.368611	*WWP2*
18	36800001	36850000	0.368611	*NOB1*
18	36800001	36850000	0.368611	*U6*
20	42000001	42050000	0.368541	*DROSHA*
8	7650001	7700000	0.3685	*GATA4*
19	57925001	57975000	0.368484	*SDK2*
5	111675001	111725000	0.368454	*SGSM3*
5	111675001	111725000	0.368454	*ADSL*
5	111250001	111300000	0.36845	*GRAP2*
5	111250001	111300000	0.36845	*ENTHD1*
11	77875001	77925000	0.368342	*APOB*
11	375001	425000	0.368299	*MERTK*
7	12600001	12650000	0.368273	*FARSA*
7	12600001	12650000	0.368273	*SYCE2*
7	12600001	12650000	0.368273	*RAD23A*
7	12600001	12650000	0.368273	*CALR*
7	12600001	12650000	0.368273	*GADD45GIP1*
14	36875001	36925000	0.368252	*STAU2*
19	19125001	19175000	0.368252	*KSR1*
14	10300001	10350000	0.368233	*ASAP1*
3	120550001	120600000	0.368216	*FARP2*
3	42325001	42375000	0.36821	*SLC30A7*
3	42325001	42375000	0.36821	*U6*
28	15550001	15600000	0.368146	*CCDC6*
7	42875001	42925000	0.368137	*OR2AZ3*
19	40850001	40900000	0.368073	*KRT222*
19	40850001	40900000	0.368073	*SMARCE1*
24	61725001	61775000	0.368066	*SERPINB5*
23	16775001	16825000	0.367978	*CUL9*
23	16775001	16825000	0.367978	*SRF*
23	16775001	16825000	0.367978	*DNPH1*
3	20000001	20050000	0.367942	*HORMAD1*
3	20000001	20050000	0.367942	*GOLPH3L*
3	20000001	20050000	0.367942	*CTSS*
19	52000001	52050000	0.367921	*ENDOV*
19	52000001	52050000	0.367921	*RPTOR*
11	45700001	45750000	0.367902	*UXS1*
8	99950001	100000000	0.36788	*SVEP1*
19	22800001	22850000	0.36788	*WDR81*
19	22800001	22850000	0.36788	*SERPINF2*
19	22800001	22850000	0.36788	*TLCD2*
19	22800001	22850000	0.36788	*SERPINF1*
19	22800001	22850000	0.36788	*MIR22*
28	3700001	3750000	0.367851	*ARV1*
28	3700001	3750000	0.367851	*TTC13*
18	50425001	50475000	0.367824	*HNRNPUL1*
18	50425001	50475000	0.367824	*AXL*

5	5850001	5900000	0.367808	*OSBPL8*
7	90800001	90850000	0.367766	*ARRDC3*
13	78525001	78575000	0.367731	*PTPN1*
19	32800001	32850000	0.367726	*TEKT3*
9	81800001	81850000	0.367706	*UTRN*
4	59875001	59925000	0.367672	*ELMO1*
4	7400001	7450000	0.367653	*ABCA13*
28	41450001	41500000	0.367645	*BMPR1A*
20	53675001	53725000	0.367554	*CDH18*
29	12725001	12775000	0.367518	*PRCP*
29	12725001	12775000	0.367518	*DDIAS*
27	35050001	35100000	0.367516	*IDO2*
20	25400001	25450000	0.367514	*NDUFS4*
3	117675001	117725000	0.367503	*TRAF3IP1*
3	117675001	117725000	0.367503	*ASB1*
3	116550001	116600000	0.367463	*COPS8*
15	63950001	64000000	0.367452	*HIPK3*
15	63950001	64000000	0.367452	*KIAA1549L*
29	49375001	49425000	0.367315	*IGF2*
29	49375001	49425000	0.367315	*TH*
29	49375001	49425000	0.367315	*INS*
9	15500001	15550000	0.367251	*MYO6*
5	47575001	47625000	0.367239	*IRAK3*
5	104625001	104675000	0.367223	*ANO2*
29	49950001	50000000	0.367165	*DUSP8*
29	49950001	50000000	0.367165	*MOB2*
3	40525001	40575000	0.367136	*COL11A1*
15	53500001	53550000	0.367097	*C2CD3*
17	6800001	6850000	0.367082	*LRBA*
28	44200001	44250000	0.367081	*MARCHF8*
28	44200001	44250000	0.367081	*ZFAND4*
3	120225001	120275000	0.367078	*CROCC2*
4	5200001	5250000	0.366981	*GRB10*
7	43750001	43800000	0.366979	*PWWP3A*
4	76700001	76750000	0.366964	*ZMIZ2*
4	76700001	76750000	0.366964	*PPIA*
4	76700001	76750000	0.366964	*H2AZ2*
2	5775001	5825000	0.36695	*NEMP2*
2	5775001	5825000	0.36695	*MFSD6*
8	16275001	16325000	0.366913	*LINGO2*
4	80675001	80725000	0.366879	*SUGCT*
5	11225001	11275000	0.366811	*PPFIA2*
7	89900001	89950000	0.366806	*CETN3*
15	45250001	45300000	0.366727	*SYT9*
15	45250001	45300000	0.366727	*OLFML1*
19	49900001	49950000	0.366724	*TBCD*
19	49900001	49950000	0.366724	*FN3KRP*
19	49900001	49950000	0.366724	*SNORA73*
28	24775001	24825000	0.366654	*RUFY2*
28	24775001	24825000	0.366654	*DNA2*
4	12200001	12250000	0.366649	*PPP1R9A*
6	112075001	112125000	0.366639	*LDB2*

13	61775001	61825000	0.366621	*ASXL1*
13	61775001	61825000	0.366621	*KIF3B*
8	61700001	61750000	0.366571	*FRMPD1*
15	40700001	40750000	0.366516	*DKK3*
15	40700001	40750000	0.366516	*USP47*
3	25775001	25825000	0.366491	*MAN1A2*
19	12200001	12250000	0.366427	*BCAS3*
1	153225001	153275000	0.366425	*DPH3*
1	153225001	153275000	0.366425	*GALNT15*
1	153225001	153275000	0.366425	*OXNAD1*
13	62575001	62625000	0.366384	*BPIFA2C*
8	72850001	72900000	0.366383	*CDCA2*
5	109100001	109150000	0.366355	*BCL2L13*
5	109100001	109150000	0.366355	*BID*
22	44975001	45025000	0.366342	*ERC2*
15	51750001	51800000	0.36629	*FOLR1*
15	51750001	51800000	0.36629	*FOLR3*
22	41400001	41450000	0.366287	*FHIT*
19	33425001	33475000	0.366278	*TTC19*
19	33425001	33475000	0.366278	*NCOR1*
19	33425001	33475000	0.366278	*ZSWIM7*
20	53650001	53700000	0.366242	*CDH18*
28	44625001	44675000	0.366239	*TMEM72*
28	44625001	44675000	0.366239	*RASSF4*
22	44125001	44175000	0.366197	*ARHGEF3*
19	38800001	38850000	0.366178	*NPEPPS*
16	33100001	33150000	0.366137	*ZBTB18*
14	26425001	26475000	0.366073	*CHD7*
2	8825001	8875000	0.366058	*TFPI*
12	19350001	19400000	0.366039	*SPRYD7*
28	8175001	8225000	0.366033	*B3GALNT2*
28	8175001	8225000	0.366033	*TBCE*
2	126825001	126875000	0.366021	*CEP85*
2	126825001	126875000	0.366021	*UBXN11*
2	126825001	126875000	0.366021	*SH3BGRL3*
7	42825001	42875000	0.366011	*OR2AV11*
16	65125001	65175000	0.365982	*TSEN15*
16	3350001	3400000	0.365932	*ELK4*
16	3350001	3400000	0.365932	*MFSD4A*
15	40325001	40375000	0.365924	*PARVA*
15	40325001	40375000	0.365924	*MICAL2*
5	112650001	112700000	0.36588	*PMM1*
5	112650001	112700000	0.36588	*DESI1*
5	112650001	112700000	0.36588	*CSDC2*
2	125825001	125875000	0.365876	*WASF2*
5	47925001	47975000	0.365876	*HMGA2*
2	125825001	125875000	0.365876	*U6*
22	49450001	49500000	0.365851	*DOCK3*
19	28700001	28750000	0.365841	*STX8*
22	47150001	47200000	0.365841	*CACNA1D*
22	47150001	47200000	0.365841	*CHDH*
4	11950001	12000000	0.365793	*SGCE*

13	17450001	17500000	0.365763	*IL2RA*
18	2350001	2400000	0.365742	*ZNRF1*
5	104325001	104375000	0.365634	*VWF*
5	104325001	104375000	0.365634	*ANO2*
14	32600001	32650000	0.365569	*C14H8orf34*
25	20775001	20825000	0.365543	*USP31*
16	3525001	3575000	0.365536	*SLC41A1*
1	1675001	1725000	0.365528	*ITSN1*
14	38825001	38875000	0.365524	*HNF4G*
19	27250001	27300000	0.365471	*TNFSF12*
19	27250001	27300000	0.365471	*SENP3*
19	27250001	27300000	0.365471	*TNFSF13*
12	19500001	19550000	0.365453	*MIR15A*
8	29050001	29100000	0.365427	*TTC39B*
19	38925001	38975000	0.365412	*MRPL45*
19	38925001	38975000	0.365412	*NPEPPS*
1	156750001	156800000	0.365386	*KCNH8*
3	36300001	36350000	0.365369	*PRMT6*
5	77700001	77750000	0.365365	*BICD1*
27	34875001	34925000	0.365364	*ADAM18*
25	3575001	3625000	0.365323	*DNAJA3*
25	3575001	3625000	0.365323	*NMRAL1*
2	5750001	5800000	0.365304	*NEMP2*
19	24950001	25000000	0.365297	*SPNS3*
19	24950001	25000000	0.365297	*SPNS2*
1	146075001	146125000	0.365215	*PCNT*
1	146075001	146125000	0.365215	*DIP2A*
18	54225001	54275000	0.365201	*SAE1*
18	54225001	54275000	0.365201	*CCDC9*
18	54225001	54275000	0.365201	*BBC3*
5	77875001	77925000	0.365188	*RESF1*
3	36125001	36175000	0.365126	*NTNG1*
10	58025001	58075000	0.36511	*MYO5A*
10	58025001	58075000	0.36511	*MYO5C*
13	57100001	57150000	0.36503	*ZNF831*
5	11275001	11325000	0.364967	*PPFIA2*
6	112100001	112150000	0.364965	*LDB2*
8	90250001	90300000	0.364961	*TMEFF1*
1	143950001	144000000	0.364917	*AGPAT3*
1	107525001	107575000	0.364884	*SCHIP1*
1	146250001	146300000	0.36483	*S100B*
4	69675001	69725000	0.364827	*CBX3*
15	77725001	77775000	0.364825	*PTPRJ*
28	25075001	25125000	0.364821	*CCAR1*
28	25075001	25125000	0.364821	*STOX1*
19	10800001	10850000	0.364793	*TUBD1*
19	10800001	10850000	0.364793	*RPS6KB1*
19	10800001	10850000	0.364793	*VMP1*
19	10800001	10850000	0.364793	*MIR21*
5	58825001	58875000	0.364786	*OR6C1N*
29	50400001	50450000	0.364751	*AP2A2*
12	29200001	29250000	0.364726	*RXFP2*

8	69350001	69400000	0.364712	*DMTN*
8	69350001	69400000	0.364712	*FGF17*
8	69350001	69400000	0.364712	*NPM2*
22	48075001	48125000	0.364673	*ITIH1*
22	48075001	48125000	0.364673	*ITIH3*
22	48075001	48125000	0.364673	*ITIH4*
22	48075001	48125000	0.364673	*NEK4*
2	22600001	22650000	0.364617	*OLA1*
7	48450001	48500000	0.364541	*SPOCK1*
16	6550001	6600000	0.364469	*KCNT2*
14	33950001	34000000	0.364464	*NCOA2*
4	11975001	12025000	0.364457	*SGCE*
16	49450001	49500000	0.364446	*MEGF6*
19	44025001	44075000	0.364425	*UBTF*
19	44025001	44075000	0.364425	*SLC4A1*
15	50950001	51000000	0.364394	*RRM1*
15	50950001	51000000	0.364394	*OR55B1*
5	109125001	109175000	0.364383	*BID*
5	109125001	109175000	0.364383	*MICAL3*
5	109125001	109175000	0.364383	*BCL2L13*
4	67575001	67625000	0.364367	*CREB5*
14	35125001	35175000	0.364321	*EYA1*
21	28075001	28125000	0.364309	*FAM189A1*
21	28075001	28125000	0.364309	*APBA2*
20	325001	375000	0.36424	*PANK3*
29	48225001	48275000	0.364175	*ACTE1*
16	41950001	42000000	0.364159	*DRAXIN*
16	41950001	42000000	0.364159	*MAD2L2*
22	48500001	48550000	0.364154	*DNAH1*
3	101450001	101500000	0.364151	*RNF220*
2	47425001	47475000	0.364111	*EPC2*
8	90225001	90275000	0.364083	*TMEFF1*
11	11700001	11750000	0.364021	*EXOC6B*
19	44525001	44575000	0.364	*GJC1*
19	44525001	44575000	0.364	*EFTUD2*
19	44525001	44575000	0.364	*HIGD1B*
18	11575001	11625000	0.363992	*GSE1*
19	38875001	38925000	0.363936	*NPEPPS*
15	80875001	80925000	0.363936	*ZDHHC5*
15	80875001	80925000	0.363936	*YPEL4*
15	80875001	80925000	0.363936	*CLP1*
5	47150001	47200000	0.363903	*GRIP1*
2	89150001	89200000	0.363901	*AOX1*
15	79125001	79175000	0.363809	*OR8K67*
15	79125001	79175000	0.363809	*OR8K3B*
19	42850001	42900000	0.363776	*AOC2*
19	42850001	42900000	0.363776	*PSME3*
19	42850001	42900000	0.363776	*AOC3*
19	42850001	42900000	0.363776	*SAO*
19	42850001	42900000	0.363776	*U6*
8	80150001	80200000	0.363769	*SNORA70*
3	120350001	120400000	0.363747	*PPP1R7*

3	120350001	120400000	0.363747	*PASK*
19	52075001	52125000	0.363699	*ENDOV*
8	76750001	76800000	0.363648	*FRMD3*
2	22800001	22850000	0.36363	*SP3*
13	22900001	22950000	0.363536	*MLLT10*
19	33825001	33875000	0.363534	*ULK2*
23	18825001	18875000	0.363527	*RUNX2*
5	53675001	53725000	0.363522	*SLC16A7*
10	58050001	58100000	0.363508	*MYO5C*
8	72825001	72875000	0.363485	*CDCA2*
22	60225001	60275000	0.363484	*PLXNA1*
2	125850001	125900000	0.363477	*WASF2*
2	125850001	125900000	0.363477	*GPR3*
2	125850001	125900000	0.363477	*U6*
6	21875001	21925000	0.363472	*SLC9B1*
5	105100001	105150000	0.363407	*KCNA5*
5	77500001	77550000	0.363337	*BICD1*
19	47400001	47450000	0.363201	*TANC2*
22	35050001	35100000	0.363166	*SLC25A26*
22	35050001	35100000	0.363166	*LRIG1*
18	51850001	51900000	0.36315	*PLAUR*
18	51850001	51900000	0.36315	*CADM4*
20	450001	500000	0.363096	*SLIT3*
17	19475001	19525000	0.363084	*SLC7A11*
20	30050001	30100000	0.36305	*MRPS30*
19	39150001	39200000	0.363014	*SRCIN1*
18	14350001	14400000	0.362947	*ANKRD11*
22	49575001	49625000	0.362878	*DOCK3*
2	125775001	125825000	0.362864	*WASF2*
2	125775001	125825000	0.362864	*AHDC1*
22	37250001	37300000	0.36286	*PRICKLE2*
8	91275001	91325000	0.362843	*RNF20*
8	91275001	91325000	0.362843	*GRIN3A*
4	80475001	80525000	0.362812	*SUGCT*
3	120300001	120350000	0.362803	*SNED1*
3	120300001	120350000	0.362803	*PASK*
3	120300001	120350000	0.362803	*MTERF4*
3	94125001	94175000	0.362799	*ZFYVE9*
8	8100001	8150000	0.362708	*XKR6*
8	11000001	11050000	0.362705	*CCDC25*
8	11000001	11050000	0.362705	*ESCO2*
18	23200001	23250000	0.362685	*IRX5*
14	69450001	69500000	0.362669	*INTS8*
14	69450001	69500000	0.362669	*CCNE2*
15	79350001	79400000	0.362609	*OR8K62*
15	79350001	79400000	0.362609	*OR8K65P*
5	113150001	113200000	0.362489	*TCF20*
8	6275001	6325000	0.36248	*U6*
20	3750001	3800000	0.362462	*FBXW11*
3	500001	550000	0.362462	*GPR161*
6	110525001	110575000	0.362458	*C1QTNF7*
19	39075001	39125000	0.362451	*ARHGAP23*

16	49425001	49475000	0.362442	*MEGF6*
10	82275001	82325000	0.362438	*TTC9*
11	11650001	11700000	0.362427	*EXOC6B*
4	119375001	119425000	0.362398	*NCAPG2*
20	14800001	14850000	0.362358	*RGS7BP*
16	39900001	39950000	0.362346	*C16H1orf105*
16	39900001	39950000	0.362346	*DNM3*
16	39900001	39950000	0.362346	*PIGC*
12	29175001	29225000	0.36226	*RXFP2*
28	44350001	44400000	0.362248	*ALOX5*
22	46600001	46650000	0.362209	*CACNA2D3*
3	25350001	25400000	0.362147	*GDAP2*
15	76850001	76900000	0.362119	*CSTPP1*
8	78025001	78075000	0.362099	*NTRK2*
25	28300001	28350000	0.362089	*TMEM248*
5	104300001	104350000	0.362069	*VWF*
17	6950001	7000000	0.362025	*LRBA*
21	67675001	67725000	0.361929	*CDC42BPB*
16	71700001	71750000	0.361907	*LPGAT1*
6	67625001	67675000	0.361906	*CWH43*
19	25300001	25350000	0.361863	*PITPNM3*
8	8975001	9025000	0.361811	*MSRA*
14	37250001	37300000	0.361806	*LY96*
23	16525001	16575000	0.361779	*CNPY3*
23	16525001	16575000	0.361779	*PTCRA*
23	16525001	16575000	0.361779	*GNMT*
11	150001	200000	0.361775	*ZC3H6*
19	11075001	11125000	0.36172	*MED13*
14	33450001	33500000	0.361704	*SULF1*
14	33450001	33500000	0.361704	*SLCO5A1*
29	28075001	28125000	0.36169	*TBRG1*
29	28075001	28125000	0.36169	*SIAE*
29	28075001	28125000	0.36169	*PANX3*
2	18400001	18450000	0.361655	*PLEKHA3*
2	18400001	18450000	0.361655	*PRKRA*
2	18400001	18450000	0.361655	*FKBP7*
2	18400001	18450000	0.361655	*PJVK*
2	26375001	26425000	0.361646	*UBR3*
28	17550001	17600000	0.361638	*CABCOCO1*
29	26675001	26725000	0.361615	*OR10G9E*
21	24975001	25025000	0.36151	*BTBD1*
13	73675001	73725000	0.361509	*SDC4*
13	73675001	73725000	0.361509	*SYS1*
13	73675001	73725000	0.361509	*TP53TG5*
22	45175001	45225000	0.361477	*ERC2*
15	40600001	40650000	0.361475	*MICAL2*
22	45150001	45200000	0.361452	*ERC2*
22	6325001	6375000	0.36145	*OSBPL10*
4	69750001	69800000	0.361444	*NFE2L3*
18	11200001	11250000	0.361424	*CIBAR2*
15	62050001	62100000	0.3614	*DCDC1*
13	1575001	1625000	0.361389	*PLCB1*

10	75050001	75100000	0.36137	*KCNH5*
8	99850001	99900000	0.361272	*TXNDC8*
18	2750001	2800000	0.361266	*BCNT2*
18	2750001	2800000	0.361266	*CFDP2*
16	73100001	73150000	0.361263	*SERTAD4*
5	111550001	111600000	0.361251	*TNRC6B*
4	80725001	80775000	0.361239	*SUGCT*
13	11475001	11525000	0.361231	*CCDC3*
21	46050001	46100000	0.361225	*BRMS1L*
7	31000001	31050000	0.361215	*SNX2*
6	110550001	110600000	0.361145	*C1QTNF7*
15	43075001	43125000	0.36112	*WEE1*
15	43075001	43125000	0.36112	*ZNF143*
15	43075001	43125000	0.36112	*U6*
19	37775001	37825000	0.361103	*HOXB13*
5	108900001	108950000	0.361101	*CECR2*
15	53900001	53950000	0.361088	*POLD3*
19	27775001	27825000	0.360975	*PER1*
19	27775001	27825000	0.360975	*VAMP2*
19	27775001	27825000	0.360975	*TMEM107*
19	27775001	27825000	0.360975	*U8*
1	88150001	88200000	0.360931	*KCNMB2*
6	31300001	31350000	0.360926	*GRID2*
19	38600001	38650000	0.360923	*OSBPL7*
19	38600001	38650000	0.360923	*MRPL10*
19	38600001	38650000	0.360923	*LRRC46*
19	38600001	38650000	0.360923	*SCRN2*
19	38600001	38650000	0.360923	*SP6*
19	27025001	27075000	0.360901	*KCTD11*
19	27025001	27075000	0.360901	*NEURL4*
19	27025001	27075000	0.360901	*TNK1*
19	27025001	27075000	0.360901	*PLSCR3*
19	27025001	27075000	0.360901	*TMEM95*
5	110850001	110900000	0.360897	*MGAT3*
5	110850001	110900000	0.360897	*TAB1*
21	44925001	44975000	0.360888	*EAPP*
21	44925001	44975000	0.360888	*U1*
21	44925001	44975000	0.360888	*U1*
21	44925001	44975000	0.360888	*U1*
7	14675001	14725000	0.360824	*DNMT1*
7	14675001	14725000	0.360824	*EIF3G*
5	77475001	77525000	0.360822	*BICD1*
3	18275001	18325000	0.360722	*CRNN*
2	61650001	61700000	0.360691	*R3HDM1*
2	61650001	61700000	0.360691	*MIR128-1*
6	88600001	88650000	0.36067	*RASSF6*
15	38475001	38525000	0.360666	*SPON1*
24	56825001	56875000	0.360651	*NARS1*
24	56825001	56875000	0.360651	*ATP8B1*
24	56825001	56875000	0.360651	*U6*
11	19425001	19475000	0.360648	*HEATR5B*
8	90750001	90800000	0.360623	*PLPPR1*

3	525001	575000	0.360614	*GPR161*
3	525001	575000	0.360614	*DCAF6*
11	78150001	78200000	0.360513	*LDAH*
11	69300001	69350000	0.360507	*LCLAT1*
18	15700001	15750000	0.360494	*ITFG1*
4	119500001	119550000	0.36047	*DYNC2I1*
4	119500001	119550000	0.36047	*ESYT2*
4	119525001	119575000	0.360429	*DYNC2I1*
15	58050001	58100000	0.360314	*CCDC34*
15	58050001	58100000	0.360314	*LGR4*
1	146225001	146275000	0.360294	*DIP2A*
1	146225001	146275000	0.360294	*S100B*
2	124900001	124950000	0.360241	*PHACTR4*
2	124900001	124950000	0.360241	*RCC1*
2	124900001	124950000	0.360241	*SNORA73*
2	124900001	124950000	0.360241	*SNORA73*
2	124900001	124950000	0.360241	*SNORA73*
2	18350001	18400000	0.360188	*PLEKHA3*
8	65100001	65150000	0.360152	*ERP44*
8	65100001	65150000	0.360152	*INVS*
6	91025001	91075000	0.360147	*SCARB2*
6	91025001	91075000	0.360147	*NUP54*
19	27600001	27650000	0.36013	*CNTROB*
19	27600001	27650000	0.36013	*KCNAB3*
19	27600001	27650000	0.36013	*TRAPPC1*
15	40425001	40475000	0.360117	*MICAL2*
11	12675001	12725000	0.360085	*U6*
7	21775001	21825000	0.360079	*RAD50*
23	16600001	16650000	0.360034	*PPP2R5D*
23	16600001	16650000	0.360034	*PEX6*
18	10575001	10625000	0.360007	*ATP2C2*
18	10575001	10625000	0.360007	*WFDC1*
16	41900001	41950000	0.360006	*AGTRAP*
5	10225001	10275000	0.360001	*PTPRQ*
5	10225001	10275000	0.360001	*U6*
22	51650001	51700000	0.359992	*CDC25A*
22	51650001	51700000	0.359992	*CATHL6*
22	51650001	51700000	0.359992	*CATHL5*
16	3000001	3050000	0.359983	*TMCC2*
13	32025001	32075000	0.359961	*HACD1*
1	2700001	2750000	0.359946	*C1H21orf62*
1	142625001	142675000	0.359935	*TMPRSS3*
1	142625001	142675000	0.359935	*TFF1*
15	28550001	28600000	0.359934	*TMPRSS13*
13	17800001	17850000	0.359919	*ABI1*
13	17800001	17850000	0.359919	*ACBD5*
29	49575001	49625000	0.359875	*LSP1*
29	49575001	49625000	0.359875	*TNNT3*
29	49575001	49625000	0.359875	*PRR33*
21	65075001	65125000	0.359871	*DEGS2*
21	65075001	65125000	0.359871	*EVL*
20	35400001	35450000	0.359856	*RICTOR*

6	5850001	5900000	0.359824	*PDE5A*
28	24800001	24850000	0.359781	*DNA2*
28	24800001	24850000	0.359781	*RUFY2*
28	24800001	24850000	0.359781	*U6*
19	27725001	27775000	0.359756	*ALOXE3*
19	27725001	27775000	0.359756	*ALOX12B*
19	27725001	27775000	0.359756	*HES7*
19	27725001	27775000	0.359756	*U6*
2	9675001	9725000	0.359691	*ITGAV*
28	29050001	29100000	0.359613	*P4HA1*
20	33750001	33800000	0.359612	*PTGER4*
22	49525001	49575000	0.359611	*DOCK3*
19	47700001	47750000	0.359553	*TANC2*
28	3750001	3800000	0.359506	*FAM89A*
14	34125001	34175000	0.359494	*U6*
22	41500001	41550000	0.359488	*FHIT*
4	119625001	119675000	0.359453	*VIPR2*
20	15225001	15275000	0.359434	*RNF180*
22	48000001	48050000	0.359375	*TMEM110*
13	17225001	17275000	0.359373	*PFKFB3*
22	34050001	34100000	0.359323	*SUCLG2*
2	47525001	47575000	0.359311	*EPC2*
2	47525001	47575000	0.359311	*U6*
16	64675001	64725000	0.359277	*ARPC5*
16	64675001	64725000	0.359277	*APOBEC4*
3	120325001	120375000	0.359244	*PASK*
3	120325001	120375000	0.359244	*MTERF4*
3	120325001	120375000	0.359244	*PPP1R7*
19	51125001	51175000	0.359219	*HGS*
19	51125001	51175000	0.359219	*CCDC137*
19	51125001	51175000	0.359219	*TSPAN10*
19	51125001	51175000	0.359219	*NPLOC4*
19	51125001	51175000	0.359219	*OXLD1*
19	51125001	51175000	0.359219	*PDE6G*
19	51125001	51175000	0.359219	*ARL16*
1	144075001	144125000	0.359209	*TRAPPC10*
2	107225001	107275000	0.3592	*PTPRN*
2	107225001	107275000	0.3592	*DNAJB2*
2	107225001	107275000	0.3592	*RESP18*
1	152450001	152500000	0.359199	*COLQ*
1	152450001	152500000	0.359199	*EAF1*
1	152450001	152500000	0.359199	*METTL6*
19	33350001	33400000	0.359184	*NCOR1*
5	103975001	104025000	0.359182	*SCNN1A*
5	103975001	104025000	0.359182	*LTBR*
5	103975001	104025000	0.359182	*TNFRSF1A*
2	27200001	27250000	0.359144	*ABCB11*
2	27200001	27250000	0.359144	*G6PC2*
2	27200001	27250000	0.359144	*SPC25*
14	36250001	36300000	0.359138	*KCNB2*
28	3725001	3775000	0.359131	*FAM89A*
28	3725001	3775000	0.359131	*ARV1*

19	37950001	38000000	0.359075	*HOXB1*
20	3725001	3775000	0.359035	*FBXW11*
29	12700001	12750000	0.359032	*PRCP*
29	12700001	12750000	0.359032	*DDIAS*
18	46425001	46475000	0.358938	*KMT2B*
18	46425001	46475000	0.358938	*LIN37*
18	46425001	46475000	0.358938	*PROSER3*
18	46425001	46475000	0.358938	*ZBTB32*
18	46425001	46475000	0.358938	*IGFLR1*
18	46425001	46475000	0.358938	*HSPB6*
18	46425001	46475000	0.358938	*U2AF1L4*
18	46425001	46475000	0.358938	*PSENEN*
3	105975001	106025000	0.358936	*PPT1*
4	59950001	60000000	0.358934	*ELMO1*
8	62650001	62700000	0.358914	*TDRD7*
2	61700001	61750000	0.358804	*R3HDM1*
29	26600001	26650000	0.358781	*OR10G6*
29	26600001	26650000	0.358781	*OR8B72*
29	26600001	26650000	0.358781	*OR10S1*
29	26600001	26650000	0.358781	*OR10G9G*
29	26600001	26650000	0.358781	*SNORA71*
1	148725001	148775000	0.358703	*CLDN14*
18	11550001	11600000	0.358701	*GSE1*
13	22700001	22750000	0.358665	*SKIDA1*
13	22700001	22750000	0.358665	*MLLT10*
20	13975001	14025000	0.358605	*CENPK*
20	13975001	14025000	0.358605	*PPWD1*
22	33875001	33925000	0.358521	*SUCLG2*
24	275001	325000	0.358495	*OR5W29P*
14	24575001	24625000	0.35846	*UBXN2B*
8	71550001	71600000	0.3584	*ADAM28*
8	71550001	71600000	0.3584	*ADAMDEC1*
19	52500001	52550000	0.358371	*CCDC40*
19	52500001	52550000	0.358371	*TBC1D16*
13	17350001	17400000	0.358359	*PFKFB3*
1	153250001	153300000	0.358355	*OXNAD1*
1	153250001	153300000	0.358355	*DPH3*
16	50800001	50850000	0.358337	*CFAP74*
16	50800001	50850000	0.358337	*TMEM52*
16	50800001	50850000	0.358337	*U6*
10	36675001	36725000	0.358323	*EXD1*
10	36675001	36725000	0.358323	*CHP1*
7	14500001	14550000	0.358322	*OLFM2*
7	14500001	14550000	0.358322	*COL5A3*
24	54600001	54650000	0.358315	*TCF4*
8	8850001	8900000	0.358296	*MSRA*
16	43375001	43425000	0.358275	*UBE4B*
28	8100001	8150000	0.358268	*TBCE*
8	7100001	7150000	0.358255	*GLRA3*
3	21325001	21375000	0.358228	*HJV*
3	21325001	21375000	0.358228	*U1*
3	21325001	21375000	0.358228	*U1*

11	11675001	11725000	0.35821	*EXOC6B*
3	108925001	108975000	0.358192	*GRIK3*
21	1150001	1200000	0.358063	*MKRN3*
19	52775001	52825000	0.358058	*ENPP7*
8	60025001	60075000	0.358042	*TMEM8B*
8	60025001	60075000	0.358042	*OR13E10*
8	60025001	60075000	0.358042	*OR13E1*
28	17575001	17625000	0.358028	*CABCOCO1*
25	18750001	18800000	0.358016	*DNAH3*
7	12825001	12875000	0.358009	*MAN2B1*
7	12825001	12875000	0.358009	*WDR83OS*
8	7025001	7075000	0.357997	*GLRA3*
5	29025001	29075000	0.357977	*TMPRSS12*
22	42925001	42975000	0.357944	*PXK*
22	42925001	42975000	0.357944	*PDHB*
22	24950001	25000000	0.357928	*CNTN6*
26	36800001	36850000	0.357902	*CCDC172*
3	57125001	57175000	0.357854	*SELENOF*
3	57125001	57175000	0.357854	*HS2ST1*
7	14375001	14425000	0.357844	*FBXL12*
7	14375001	14425000	0.357844	*UBL5*
20	425001	475000	0.357804	*SLIT3*
5	109350001	109400000	0.357778	*TUBA8*
5	109350001	109400000	0.357778	*PEX26*
5	109350001	109400000	0.357778	*U6*
5	108575001	108625000	0.35775	*CACNA1C*
11	1625001	1675000	0.35774	*TPC3*
11	1625001	1675000	0.35774	*U6*
17	6925001	6975000	0.357725	*LRBA*
18	10625001	10675000	0.357709	*ATP2C2*
7	14575001	14625000	0.357689	*COL5A3*
7	14575001	14625000	0.357689	*RDH8*
22	30375001	30425000	0.357661	*FOXP1*
25	14375001	14425000	0.357656	*ABCC1*
28	26800001	26850000	0.357641	*ADAMTS14*
22	34000001	34050000	0.357634	*SUCLG2*
22	49100001	49150000	0.357565	*TEX264*
22	49100001	49150000	0.357565	*GRM2*
11	19400001	19450000	0.357541	*STRN*
11	19400001	19450000	0.357541	*HEATR5B*
5	77675001	77725000	0.357538	*BICD1*
3	101350001	101400000	0.357533	*RNF220*
3	101350001	101400000	0.357533	*TMEM53*
3	101350001	101400000	0.357533	*ARMH1*
11	46950001	47000000	0.357523	*PAX8*
18	22150001	22200000	0.357507	*FTO*
18	2425001	2475000	0.357504	*ZNRF1*
18	2425001	2475000	0.357504	*LDHD*
19	47200001	47250000	0.357465	*MARCHF10*
1	925001	975000	0.357455	*RCAN1*
3	92275001	92325000	0.35739	*HSPB11*
15	74600001	74650000	0.357376	*CD82*

7	43975001	44025000	0.357375	*TCF3*
7	43975001	44025000	0.357375	*UQCR11*
3	20675001	20725000	0.357374	*SV2A*
3	20675001	20725000	0.357374	*MTMR11*
3	20675001	20725000	0.357374	*H2AC20*
3	20675001	20725000	0.357374	*SF3B4*
3	20675001	20725000	0.357374	*BOLA1*
3	20675001	20725000	0.357374	*H2AC21*
2	47500001	47550000	0.357308	*EPC2*
2	47500001	47550000	0.357308	*U6*
13	22850001	22900000	0.35726	*MLLT10*
19	52375001	52425000	0.357252	*SLC26A11*
19	52375001	52425000	0.357252	*SGSH*
19	56925001	56975000	0.35725	*CD300A*
19	56925001	56975000	0.35725	*GPRC5C*
12	31625001	31675000	0.357218	*FLT1*
8	8575001	8625000	0.357193	*C8H8orf74*
7	21650001	21700000	0.357172	*KIF3A*
7	21650001	21700000	0.357172	*IL4*
15	40000001	40050000	0.357133	*TEAD1*
15	35850001	35900000	0.357101	*SOX6*
21	69775001	69825000	0.357094	*TMEM121*
21	69775001	69825000	0.357094	*U6*
11	101625001	101675000	0.357078	*PRRT1B*
11	101625001	101675000	0.357078	*POMT1*
11	101625001	101675000	0.357078	*UCK1*
28	28300001	28350000	0.357067	*DDIT4*
1	152350001	152400000	0.357058	*SH3BP5*
20	5600001	5650000	0.357055	*CPEB4*
14	39900001	39950000	0.357039	*ZFHX4*
25	25675001	25725000	0.357034	*XPO6*
19	38625001	38675000	0.356984	*OSBPL7*
19	38625001	38675000	0.356984	*MRPL10*
19	38625001	38675000	0.356984	*LRRC46*
19	38625001	38675000	0.356984	*SCRN2*
2	47075001	47125000	0.356972	*LYPD6B*
11	12300001	12350000	0.35696	*EXOC6B*
12	86650001	86700000	0.356885	*DCUN1D2*
12	86650001	86700000	0.356885	*TMCO3*
16	72950001	73000000	0.356857	*HHAT*
16	72950001	73000000	0.356857	*U5*
22	52575001	52625000	0.356852	*PTH1R*
22	52575001	52625000	0.356852	*CCDC12*
7	43925001	43975000	0.356849	*MBD3*
7	43925001	43975000	0.356849	*MEX3D*
22	37275001	37325000	0.356848	*PRICKLE2*
7	43375001	43425000	0.356846	*PLPPR3*
7	43375001	43425000	0.356846	*MED16*
7	43375001	43425000	0.356846	*PRTN3*
7	43375001	43425000	0.356846	*PTBP1*
7	43375001	43425000	0.356846	*ELANE*
7	43375001	43425000	0.356846	*AZU1*

7	43375001	43425000	0.356846	*CFD*
7	43375001	43425000	0.356846	*U6*
2	124925001	124975000	0.356839	*PHACTR4*
24	56925001	56975000	0.356805	*ATP8B1*
19	50300001	50350000	0.356795	*SECTM1A*
19	50300001	50350000	0.356795	*SECTM1*
2	7175001	7225000	0.356791	*COL5A2*
13	70825001	70875000	0.356779	*PTPRT*
28	7950001	8000000	0.356771	*ARID4B*
14	23275001	23325000	0.356766	*RPS20*
14	23275001	23325000	0.356766	*MOS*
14	23275001	23325000	0.356766	*U1*
22	49550001	49600000	0.356765	*DOCK3*
20	24175001	24225000	0.356748	*CSPG4B*
4	5250001	5300000	0.356739	*GRB10*
8	62625001	62675000	0.356727	*TDRD7*
3	93725001	93775000	0.356725	*GPX7*
3	93725001	93775000	0.356725	*SHISAL2A*
21	64875001	64925000	0.356685	*EML1*
21	64875001	64925000	0.356685	*EVL*
29	47800001	47850000	0.356678	*SHANK2*
11	98625001	98675000	0.356672	*FAM102A*
28	7975001	8025000	0.356666	*ARID4B*
16	50350001	50400000	0.356662	*PLCH2*
16	50350001	50400000	0.356662	*PANK4*
8	69325001	69375000	0.356617	*NPM2*
8	69325001	69375000	0.356617	*FGF17*
8	69325001	69375000	0.356617	*DMTN*
8	69325001	69375000	0.356617	*XPO7*
8	90300001	90350000	0.35656	*TMEFF1*
8	90300001	90350000	0.35656	*CAVIN4*
22	49375001	49425000	0.356559	*DOCK3*
22	49375001	49425000	0.356559	*MANF*
22	49375001	49425000	0.356559	*RBM15B*
17	6850001	6900000	0.356434	*LRBA*
28	24875001	24925000	0.35636	*SLC25A16*
28	24875001	24925000	0.35636	*TET1*
15	52425001	52475000	0.356316	*FCHSD2*
7	21625001	21675000	0.356218	*KIF3A*
18	2450001	2500000	0.356192	*LDHD*
18	2450001	2500000	0.356192	*ZNRF1*
18	2450001	2500000	0.356192	*ZFP1*
16	63425001	63475000	0.356146	*GLUL*
19	11350001	11400000	0.356144	*BRIP1*
4	119350001	119400000	0.356141	*NCAPG2*
2	23375001	23425000	0.356074	*MAP3K20*
8	76600001	76650000	0.356067	*FRMD3*
23	17700001	17750000	0.356066	*SLC29A1*
23	17700001	17750000	0.356066	*MYMX*
22	34075001	34125000	0.356052	*SUCLG2*
14	31700001	31750000	0.35605	*CPA6*
22	53475001	53525000	0.356037	*SLC6A20*

22	53475001	53525000	0.356037	*LZTFL1*
19	38375001	38425000	0.35602	*CBX1*
19	38375001	38425000	0.35602	*SNX11*
19	38375001	38425000	0.35602	*NFE2L1*
22	48050001	48100000	0.356013	*ITIH4*
22	48050001	48100000	0.356013	*ITIH3*
22	48050001	48100000	0.356013	*TMEM110*
22	48050001	48100000	0.356013	*MUSTN1*
22	48050001	48100000	0.356013	*ITIH1*
19	44100001	44150000	0.355935	*RUNDC3A*
19	44100001	44150000	0.355935	*SLC25A39*
19	44100001	44150000	0.355935	*GRN*
1	83700001	83750000	0.355933	*MCF2L2*
14	33350001	33400000	0.355883	*SULF1*
16	41925001	41975000	0.355849	*AGTRAP*
16	41925001	41975000	0.355849	*DRAXIN*
1	93450001	93500000	0.355832	*NLGN1*
21	64850001	64900000	0.355829	*EML1*
7	7725001	7775000	0.355824	*AKAP8L*
7	7725001	7775000	0.355824	*AKAP8*
7	7725001	7775000	0.355824	*WIZ*
22	37125001	37175000	0.355755	*PRICKLE2*
29	26575001	26625000	0.355743	*OR4D5*
29	26575001	26625000	0.355743	*OR8B72*
29	26575001	26625000	0.355743	*OR10S1*
29	26575001	26625000	0.355743	*SNORA71*
29	26575001	26625000	0.355743	*U6*
11	11825001	11875000	0.355724	*EXOC6B*
3	94150001	94200000	0.355704	*ZFYVE9*
6	31325001	31375000	0.355649	*GRID2*
18	56950001	57000000	0.355639	*KLK7*
18	56950001	57000000	0.355639	*KLK9*
18	56950001	57000000	0.355639	*KLK10*
18	56950001	57000000	0.355639	*KLK8*
18	56950001	57000000	0.355639	*KLK6*
18	56950001	57000000	0.355639	*KLK11*
28	8500001	8550000	0.355632	*LYST*
20	38025001	38075000	0.355615	*SKP2*
20	38025001	38075000	0.355615	*NADK2*
29	1450001	1500000	0.355604	*DEUP1*
19	34300001	34350000	0.355597	*SHMT1*
3	48675001	48725000	0.355589	*SLC44A3*
22	46750001	46800000	0.355543	*CACNA2D3*
11	78750001	78800000	0.355504	*LAPTM4A*
28	17650001	17700000	0.355492	*CABCOCO1*
26	4750001	4800000	0.355476	*PCDH15*
5	112675001	112725000	0.355435	*DESI1*
5	112675001	112725000	0.355435	*XRCC6*
22	33425001	33475000	0.355359	*TAFA1*
10	75475001	75525000	0.355352	*RHOJ*
5	65950001	66000000	0.355323	*PARPBP*
5	65950001	66000000	0.355323	*NUP37*

20	13950001	14000000	0.355318	*TRIM23*
20	13950001	14000000	0.355318	*PPWD1*
20	13950001	14000000	0.355318	*CENPK*
13	70875001	70925000	0.355246	*PTPRT*
2	65500001	65550000	0.355237	*ACTR3*
22	41125001	41175000	0.355216	*FHIT*
12	12700001	12750000	0.355176	*TNFSF11*
22	49900001	49950000	0.355158	*CACNA2D2*
19	11250001	11300000	0.355146	*BRIP1*
8	60225001	60275000	0.355144	*OR13C11*
8	60225001	60275000	0.355144	*OR13C7*
21	46000001	46050000	0.355125	*BRMS1L*
2	33875001	33925000	0.35512	*KCNH7*
4	62875001	62925000	0.355093	*BMPER*
8	61600001	61650000	0.355079	*FBXO10*
8	61600001	61650000	0.355079	*POLR1E*
19	23050001	23100000	0.35506	*SMG6*
19	23050001	23100000	0.35506	*DPH1*
19	23050001	23100000	0.35506	*HIC1*
19	23050001	23100000	0.35506	*RTN4RL1*
19	23050001	23100000	0.35506	*OVCA2*
8	84600001	84650000	0.354989	*WNK2*
20	25450001	25500000	0.354985	*NDUFS4*
13	78025001	78075000	0.354969	*SNAI1*
14	33500001	33550000	0.354947	*SLCO5A1*
18	11400001	11450000	0.354916	*GSE1*
8	61050001	61100000	0.354904	*PAX5*
13	22925001	22975000	0.354892	*MLLT10*
13	22925001	22975000	0.354892	*DNAJC1*
6	94675001	94725000	0.354875	*ANTXR2*
4	47075001	47125000	0.354861	*CDHR3*
22	43825001	43875000	0.354848	*APPL1*
22	43825001	43875000	0.354848	*ASB14*
22	43825001	43875000	0.354848	*DNAH12*
21	42125001	42175000	0.354839	*NUBPL*
4	39900001	39950000	0.354807	*SEMA3C*
15	80900001	80950000	0.354773	*ZDHHC5*
15	80900001	80950000	0.354773	*TMX2*
15	80900001	80950000	0.354773	*MED19*
15	80900001	80950000	0.354773	*CLP1*
22	18875001	18925000	0.354725	*GRM7*
18	10800001	10850000	0.354725	*COTL1*
18	10800001	10850000	0.354725	*KLHL36*
22	60250001	60300000	0.354724	*PLXNA1*
22	60250001	60300000	0.354724	*CHCHD6*
14	32625001	32675000	0.354723	*C14H8orf34*
21	65150001	65200000	0.35471	*YY1*
5	112000001	112050000	0.354663	*SLC25A17*
11	3800001	3850000	0.35465	*COA5*
11	3800001	3850000	0.35465	*UNC50*
11	3800001	3850000	0.35465	*MGAT4A*
5	103325001	103375000	0.354613	*C1R*

14	30875001	30925000	0.354591	*VCPIP1*
21	40125001	40175000	0.35459	*PRKD1*
29	47875001	47925000	0.354562	*SHANK2*
1	156700001	156750000	0.354554	*KCNH8*
4	119325001	119375000	0.354529	*NCAPG2*
6	112050001	112100000	0.354477	*LDB2*
3	78400001	78450000	0.354476	*DNAI4*
3	78400001	78450000	0.354476	*MIER1*
19	47650001	47700000	0.354462	*TANC2*
19	38650001	38700000	0.354371	*OSBPL7*
22	54925001	54975000	0.35432	*SLC6A11*
5	67425001	67475000	0.354289	*NT5DC3*
5	67425001	67475000	0.354289	*STAB2*
11	104300001	104350000	0.354272	*ADAMTS13*
11	104300001	104350000	0.354272	*REXO4*
11	104300001	104350000	0.354272	*STKLD1*
20	24150001	24200000	0.354259	*CSPG4B*
18	11375001	11425000	0.354256	*GSE1*
5	105750001	105800000	0.354248	*CCND2*
10	36375001	36425000	0.354246	*SPINT1*
10	36375001	36425000	0.354246	*PPP1R14D*
10	36375001	36425000	0.354246	*RHOV*
7	43775001	43825000	0.354214	*PWWP3A*
7	43775001	43825000	0.354214	*NDUFS7*
7	43775001	43825000	0.354214	*GAMT*
7	43775001	43825000	0.354214	*DAZAP1*
6	89800001	89850000	0.354211	*PARM1*
16	72175001	72225000	0.354208	*KCNH1*
23	16475001	16525000	0.354135	*BICRAL*
23	16475001	16525000	0.354135	*RPL7L1*
18	12350001	12400000	0.354131	*MTHFSD*
18	12350001	12400000	0.354131	*FOXC2*
18	12350001	12400000	0.354131	*FOXL1*
2	18800001	18850000	0.354125	*PDE11A*
2	18800001	18850000	0.354125	*CYCT*
21	64500001	64550000	0.354123	*CCDC85C*
21	64500001	64550000	0.354123	*CCNK*
3	103525001	103575000	0.354044	*PPIH*
3	103525001	103575000	0.354044	*YBX1*
3	103525001	103575000	0.354044	*CCDC30*
2	43650001	43700000	0.354012	*FMNL2*
2	27950001	28000000	0.353995	*STK39*
8	71700001	71750000	0.353955	*ADAM7*
15	15725001	15775000	0.353941	*AMOTL1*
6	35775001	35825000	0.353921	*FAM13A*
19	20175001	20225000	0.35389	*FAM222B*
7	20925001	20975000	0.353876	*GNG7*
7	20925001	20975000	0.353876	*SLC39A3*
7	20925001	20975000	0.353876	*DIRAS1*
5	110625001	110675000	0.353863	*PDGFB*
5	110825001	110875000	0.353838	*TAB1*
19	33900001	33950000	0.353805	*SLC47A2*

19	33900001	33950000	0.353805	*ALDH3A2*
24	700001	750000	0.353804	*CTDP1*
19	28100001	28150000	0.353754	*MYH10*
7	67800001	67850000	0.353738	*SGCD*
13	41500001	41550000	0.353676	*FOXA2*
21	65175001	65225000	0.353662	*YY1*
21	65175001	65225000	0.353662	*SLC25A29*
29	28825001	28875000	0.353645	*PKNOX2*
1	149575001	149625000	0.353641	*DYRK1A*
21	24950001	25000000	0.353604	*BTBD1*
21	24950001	25000000	0.353604	*TM6SF1*
16	50575001	50625000	0.353566	*PRKCZ*
16	50575001	50625000	0.353566	*FAAP20*
11	101375001	101425000	0.353562	*NUP214*
18	53075001	53125000	0.353556	*VASP*
18	53075001	53125000	0.353556	*RTN2*
18	53075001	53125000	0.353556	*FOSB*
18	53075001	53125000	0.353556	*PPM1N*
3	74100001	74150000	0.353547	*ZRANB2*
19	27625001	27675000	0.35353	*GUCY2D*
19	27625001	27675000	0.35353	*CNTROB*
1	142600001	142650000	0.353518	*TFF1*
1	142600001	142650000	0.353518	*TFF2*
11	98825001	98875000	0.353503	*DNM1*
11	98825001	98875000	0.353503	*GOLGA2*
18	56975001	57025000	0.353464	*KLK13*
18	56975001	57025000	0.353464	*KLK9*
18	56975001	57025000	0.353464	*KLK10*
18	56975001	57025000	0.353464	*KLK12*
18	56975001	57025000	0.353464	*KLK11*
18	56975001	57025000	0.353464	*KLK8*
22	49775001	49825000	0.353423	*HEMK1*
22	49775001	49825000	0.353423	*C22H3orf18*
28	33150001	33200000	0.353408	*KCNMA1*
22	50750001	50800000	0.353388	*USP4*
22	50750001	50800000	0.353388	*GPX1*
22	50750001	50800000	0.353388	*RHOA*
22	50750001	50800000	0.353388	*U6*
23	22400001	22450000	0.353372	*CRISP2*
23	22400001	22450000	0.353372	*CRISP3*
14	23200001	23250000	0.353341	*LYN*
28	7925001	7975000	0.353335	*ARID4B*
7	6900001	6950000	0.353277	*TPM4*
7	6900001	6950000	0.353277	*OR1AB2*
4	80775001	80825000	0.353253	*SUGCT*
2	120925001	120975000	0.35323	*SYNC*
2	120925001	120975000	0.35323	*RBBP4*
8	72175001	72225000	0.353204	*NEFM*
8	72175001	72225000	0.353204	*NEFL*
29	28100001	28150000	0.35319	*SIAE*
29	28100001	28150000	0.35319	*TBRG1*
1	42275001	42325000	0.353179	*GABRR3*

29	27050001	27100000	0.353176	*OR8G3L*
22	48700001	48750000	0.35316	*POC1A*
22	48700001	48750000	0.35316	*ALAS1*
23	13275001	13325000	0.353139	*KIF6*
14	4550001	4600000	0.353079	*FAM135B*
7	48475001	48525000	0.353071	*SPOCK1*
14	1025001	1075000	0.353028	*MAPK15*
14	1025001	1075000	0.353028	*FAM83H*
14	1025001	1075000	0.353028	*IQANK1*
14	1025001	1075000	0.353028	*CCDC166*
14	1025001	1075000	0.353028	*ZNF623*
2	125725001	125775000	0.353003	*AHDC1*
5	110875001	110925000	0.352992	*MGAT3*
14	37150001	37200000	0.35297	*UBE2W*
14	37150001	37200000	0.35297	*ELOC*
16	51125001	51175000	0.352957	*CCNL2*
16	51125001	51175000	0.352957	*AURKAIP1*
16	51125001	51175000	0.352957	*MRPL20*
16	51125001	51175000	0.352957	*ANKRD65*
16	51125001	51175000	0.352957	*MXRA8*
4	81250001	81300000	0.352898	*YAE1*
13	24375001	24425000	0.352896	*OTUD1*
25	25000001	25050000	0.35285	*GTF3C1*
25	25000001	25050000	0.35285	*IL21R*
10	75525001	75575000	0.352849	*RHOJ*
6	58425001	58475000	0.352832	*RFC1*
6	58425001	58475000	0.352832	*WDR19*
10	27575001	27625000	0.352823	*OR4F69*
10	27575001	27625000	0.352823	*OR4G10*
12	31450001	31500000	0.352808	*POMP*
29	48450001	48500000	0.352804	*OSBPL5*
15	40300001	40350000	0.352788	*PARVA*
15	52450001	52500000	0.352783	*FCHSD2*
19	12325001	12375000	0.352737	*PPM1D*
3	92150001	92200000	0.352726	*TCEANC2*
3	92150001	92200000	0.352726	*TMEM59*
3	92300001	92350000	0.352721	*YIPF1*
4	84725001	84775000	0.352719	*KCND2*
19	49925001	49975000	0.352707	*RAB40B*
19	49925001	49975000	0.352707	*FN3KRP*
19	49925001	49975000	0.352707	*TBCD*
28	25050001	25100000	0.352706	*CCAR1*
2	17400001	17450000	0.352706	*ZNF385B*
20	3800001	3850000	0.352646	*FBXW11*
20	34825001	34875000	0.352529	*U2*
3	103300001	103350000	0.352521	*SLC2A1*
8	69650001	69700000	0.352364	*SLC39A14*
8	69650001	69700000	0.352364	*PIWIL2*
5	82050001	82100000	0.352352	*MRPS35*
5	82050001	82100000	0.352352	*KLHL42*
5	82050001	82100000	0.352352	*MANSC4*
12	86625001	86675000	0.352327	*DCUN1D2*

22	49300001	49350000	0.352306	*DCAF1*
21	7025001	7075000	0.352297	*MEF2A*
7	43800001	43850000	0.352265	*DAZAP1*
7	43800001	43850000	0.352265	*NDUFS7*
7	43800001	43850000	0.352265	*PWWP3A*
7	43800001	43850000	0.352265	*GAMT*
7	43800001	43850000	0.352265	*RPS15*
1	146275001	146325000	0.352249	*S100B*
1	146275001	146325000	0.352249	*PRMT2*
19	33850001	33900000	0.35223	*ULK2*
19	33850001	33900000	0.35223	*ALDH3A1*
19	33850001	33900000	0.35223	*SLC47A2*
8	60950001	61000000	0.352214	*PAX5*
8	72775001	72825000	0.352198	*KCTD9*
8	72775001	72825000	0.352198	*CDCA2*
2	27925001	27975000	0.352181	*STK39*
28	25200001	25250000	0.352108	*DDX21*
28	25200001	25250000	0.352108	*DDX50*
28	25200001	25250000	0.352108	*KIFBP*
7	11675001	11725000	0.352096	*RFX1*
11	11925001	11975000	0.352007	*EXOC6B*
4	5350001	5400000	0.351988	*DDC*
19	43875001	43925000	0.351957	*LSM12*
19	43875001	43925000	0.351957	*HDAC5*
19	43875001	43925000	0.351957	*G6PC3*
8	99675001	99725000	0.351929	*PALM2*
16	79650001	79700000	0.351924	*TMEM9*
16	79650001	79700000	0.351924	*CACNA1S*
11	9100001	9150000	0.351912	*MRPS9*
19	30600001	30650000	0.351906	*ZNF18*
19	30600001	30650000	0.351906	*DNAH9*
19	30600001	30650000	0.351906	*MAP2K4*
5	104575001	104625000	0.351901	*ANO2*
9	32700001	32750000	0.351899	*SLC35F1*
2	126800001	126850000	0.351894	*UBXN11*
2	126800001	126850000	0.351894	*CEP85*
2	126800001	126850000	0.351894	*CRYBG2*
2	126800001	126850000	0.351894	*CD52*
2	126800001	126850000	0.351894	*SH3BGRL3*
7	43650001	43700000	0.351871	*STK11*
7	43650001	43700000	0.351871	*MIDN*
7	43650001	43700000	0.351871	*CBARP*
7	43650001	43700000	0.351871	*ATP5F1D*
13	72275001	72325000	0.351835	*GTSF1L*
18	52750001	52800000	0.351828	*PPP1R37*
18	52750001	52800000	0.351828	*GEMIN7*
18	52750001	52800000	0.351828	*ZNF296*
8	62425001	62475000	0.351822	*IGFBPL1*
8	62425001	62475000	0.351822	*ALDH1B1*
18	55800001	55850000	0.351781	*TEAD2*
18	55800001	55850000	0.351781	*SLC6A16*
18	55800001	55850000	0.351781	*CD37*

18	55800001	55850000	0.351781	*DKKL1*
10	36700001	36750000	0.351779	*CHP1*
10	36700001	36750000	0.351779	*EXD1*
6	88800001	88850000	0.351721	*CXCL8*
22	48800001	48850000	0.351703	*DUSP7*
22	48800001	48850000	0.351703	*POC1A*
8	8600001	8650000	0.351645	*C8H8orf74*
7	84175001	84225000	0.351608	*EDIL3*
18	14475001	14525000	0.3516	*CPNE7*
18	14475001	14525000	0.3516	*SPG7*
18	14475001	14525000	0.3516	*RPL13*
18	14475001	14525000	0.3516	*SNORD68*
19	39125001	39175000	0.351596	*SRCIN1*
19	39125001	39175000	0.351596	*ARHGAP23*
19	31175001	31225000	0.351536	*ARHGAP44*
19	31175001	31225000	0.351536	*MYOCD*
2	96025001	96075000	0.351534	*CCNYL1*
2	96025001	96075000	0.351534	*FZD5*
21	45925001	45975000	0.351511	*RALGAPA1*
14	34925001	34975000	0.351495	*EYA1*
8	20425001	20475000	0.351435	*ELAVL2*
18	10675001	10725000	0.351421	*MEAK7*
18	10675001	10725000	0.351421	*ATP2C2*
19	51150001	51200000	0.351396	*NPLOC4*
19	51150001	51200000	0.351396	*CCDC137*
19	51150001	51200000	0.351396	*TSPAN10*
19	51150001	51200000	0.351396	*OXLD1*
19	51150001	51200000	0.351396	*PDE6G*
15	41950001	42000000	0.351395	*IRAG1*
19	52450001	52500000	0.351342	*CARD14*
19	52450001	52500000	0.351342	*GAA*
19	52450001	52500000	0.351342	*EIF4A3*
19	52450001	52500000	0.351342	*Metazoa_SRP*
13	23875001	23925000	0.351311	*U6*
13	23875001	23925000	0.351311	*Y_RNA*
22	6800001	6850000	0.351304	*CMTM7*
22	6800001	6850000	0.351304	*CMTM8*
2	47450001	47500000	0.351298	*EPC2*
7	7850001	7900000	0.351281	*BRD4*
7	7850001	7900000	0.351281	*EPHX3*
20	13400001	13450000	0.351239	*SREK1*
28	15425001	15475000	0.351236	*CCDC6*
28	15425001	15475000	0.351236	*MRLN*
7	67775001	67825000	0.35123	*SGCD*
19	44500001	44550000	0.351209	*GJC1*
19	44500001	44550000	0.351209	*ADAM11*
18	14450001	14500000	0.351149	*SPG7*
18	14450001	14500000	0.351149	*RPL13*
18	14450001	14500000	0.351149	*SNORD68*
5	29650001	29700000	0.351142	*LIMA1*
12	33175001	33225000	0.351136	*RNF6*
15	41825001	41875000	0.351135	*EIF4G2*

14	22950001	23000000	0.351105	*XKR4*
8	83125001	83175000	0.351061	*HABP4*
8	50550001	50600000	0.351034	*RORB*
9	43300001	43350000	0.351026	*QRSL1*
9	43300001	43350000	0.351026	*RTN4IP1*
18	51925001	51975000	0.351015	*SMG9*
18	51925001	51975000	0.351015	*KCNN4*
18	51925001	51975000	0.351015	*IRGC*
5	110725001	110775000	0.350991	*RPL3*
5	110725001	110775000	0.350991	*SNORD83B*
5	110725001	110775000	0.350991	*SNORD83A*
5	110725001	110775000	0.350991	*SNORD43*
15	79150001	79200000	0.350923	*OR8K3B*
15	79150001	79200000	0.350923	*OR8K64*
14	36225001	36275000	0.350886	*KCNB2*
5	110800001	110850000	0.350859	*TAB1*
5	110800001	110850000	0.350859	*SYNGR1*
22	48475001	48525000	0.350792	*DNAH1*
22	48475001	48525000	0.350792	*BAP1*
22	48475001	48525000	0.350792	*PHF7*
8	61425001	61475000	0.350766	*ZCCHC7*
18	11425001	11475000	0.350757	*GSE1*
19	33325001	33375000	0.35071	*NCOR1*
19	33325001	33375000	0.35071	*PIGL*
14	14800001	14850000	0.350709	*NSMCE2*
11	65850001	65900000	0.350663	*ETAA1*
6	88625001	88675000	0.350603	*RASSF6*
5	29050001	29100000	0.350598	*TMPRSS12*
11	102475001	102525000	0.350594	*CFAP77*
11	102475001	102525000	0.350594	*TTF1*
28	27775001	27825000	0.350552	*CDH23*
19	39200001	39250000	0.350524	*SRCIN1*
11	71850001	71900000	0.350496	*RBKS*
19	44250001	44300000	0.350488	*GPATCH8*
2	10700001	10750000	0.350483	*FSIP2*
7	43325001	43375000	0.350452	*MISP*
7	43325001	43375000	0.350452	*PTBP1*
5	81800001	81850000	0.350427	*PTHLH*
21	30675001	30725000	0.350377	*WDR61*
14	33750001	33800000	0.350375	*PRDM14*
1	87250001	87300000	0.350359	*PEX5L*
24	225001	275000	0.350356	*OR9M1D*
5	113025001	113075000	0.3503	*NDUFA6*
5	113025001	113075000	0.3503	*NAGA*
5	113025001	113075000	0.3503	*PHETA2*
5	113025001	113075000	0.3503	*SMDT1*
29	5200001	5250000	0.350262	*TRIM77*
29	5200001	5250000	0.350262	*TRIM64*
28	25475001	25525000	0.350187	*SRGN*
18	52975001	53025000	0.350156	*ERCC2*
18	52975001	53025000	0.350156	*POLR1G*
18	52975001	53025000	0.350156	*PPP1R13L*

18	52975001	53025000	0.350156	*KLC3*
18	52975001	53025000	0.350156	*ERCC1*
3	101250001	101300000	0.350142	*KIF2C*
3	101250001	101300000	0.350142	*ARMH1*
3	101250001	101300000	0.350142	*U5*
27	34600001	34650000	0.350135	*ADAM2*
3	103650001	103700000	0.35012	*CCDC30*
4	80750001	80800000	0.350114	*SUGCT*
19	47075001	47125000	0.350099	*MRC2*
19	47075001	47125000	0.350099	*MARCHF10*
14	34800001	34850000	0.350095	*EYA1*
16	45325001	45375000	0.350081	*ERRFI1*
16	79175001	79225000	0.350059	*U6*
27	33250001	33300000	0.350013	*EIF4EBP1*
27	33250001	33300000	0.350013	*ASH2L*
15	79100001	79150000	0.349993	*OR8K67*
15	79100001	79150000	0.349993	*OR8J12*
15	79100001	79150000	0.349993	*OR8J2E*
11	28325001	28375000	0.349952	*PRKCE*
19	33925001	33975000	0.349927	*ALDH3A2*
20	14250001	14300000	0.349907	*ADAMTS6*
2	106225001	106275000	0.349889	*ARPC2*
19	47175001	47225000	0.349881	*MARCHF10*
13	40525001	40575000	0.349845	*KIZ*
14	34300001	34350000	0.349806	*LACTB2*
14	34300001	34350000	0.349806	*TRAM1*
14	23100001	23150000	0.349794	*LYN*
23	15575001	15625000	0.349788	*USP49*
23	15575001	15625000	0.349788	*FRS3*
23	15575001	15625000	0.349788	*PRICKLE4*
23	15575001	15625000	0.349788	*TOMM6*
3	78625001	78675000	0.349773	*SGIP1*
2	26400001	26450000	0.34977	*UBR3*
14	975001	1025000	0.349768	*SCRIB*
14	975001	1025000	0.349768	*IQANK1*
14	975001	1025000	0.349768	*PUF60*
19	38775001	38825000	0.349753	*KPNB1*
19	38775001	38825000	0.349753	*NPEPPS*
4	81500001	81550000	0.349741	*POU6F2*
15	41850001	41900000	0.349711	*EIF4G2*
15	41850001	41900000	0.349711	*CTR9*
15	41850001	41900000	0.349711	*SNORD97*
13	23000001	23050000	0.349705	*DNAJC1*
4	74950001	75000000	0.349695	*TNS3*
7	43400001	43450000	0.349688	*MED16*
7	43400001	43450000	0.349688	*R3HDM4*
7	43400001	43450000	0.349688	*PRTN3*
7	43400001	43450000	0.349688	*ELANE*
7	43400001	43450000	0.349688	*CFD*
7	43400001	43450000	0.349688	*U6*
23	16850001	16900000	0.349687	*TTBK1*
23	16850001	16900000	0.349687	*SLC22A7*

23	16850001	16900000	0.349687	*CRIP3*
2	47475001	47525000	0.349663	*EPC2*
2	61375001	61425000	0.349659	*DARS1*
3	119975001	120025000	0.34965	*GPR35*
3	119975001	120025000	0.34965	*CAPN10*
3	119975001	120025000	0.34965	*AQP12*
15	29225001	29275000	0.349638	*ARCN1*
15	29225001	29275000	0.349638	*PHLDB1*
19	44125001	44175000	0.349609	*FAM171A2*
19	44125001	44175000	0.349609	*GRN*
19	44125001	44175000	0.349609	*SLC25A39*
19	44125001	44175000	0.349609	*ITGA2B*
19	44125001	44175000	0.349609	*RUNDC3A*
3	93775001	93825000	0.349564	*TUT4*
3	93700001	93750000	0.349563	*SHISAL2A*
3	93700001	93750000	0.349563	*GPX7*
11	100725001	100775000	0.349542	*HMCN2*
11	100725001	100775000	0.349542	*ASS1*
11	49125001	49175000	0.3495	*ATOH8*
3	79800001	79850000	0.349472	*LEPR*
6	58125001	58175000	0.349437	*FAM114A1*
15	43175001	43225000	0.349328	*IPO7*
15	43175001	43225000	0.349328	*ZNF143*
15	43175001	43225000	0.349328	*SNORA23*
15	51625001	51675000	0.349307	*NUMA1*
15	51625001	51675000	0.349307	*RNF121*
15	51625001	51675000	0.349307	*IL18BP*
7	8000001	8050000	0.349292	*ILVBL*
7	8000001	8050000	0.349292	*SYDE1*
7	8000001	8050000	0.349292	*OR1I4*
7	8000001	8050000	0.349292	*OR1I1*
4	32125001	32175000	0.349263	*TRA2A*
3	120000001	120050000	0.34926	*GPR35*
3	120000001	120050000	0.34926	*KIF1A*
3	120000001	120050000	0.34926	*AQP12*
20	15300001	15350000	0.349249	*RNF180*
21	46025001	46075000	0.349205	*BRMS1L*
16	66800001	66850000	0.349164	*HMCN1*
8	72500001	72550000	0.349155	*DOCK5*
8	4225001	4275000	0.349154	*U6*
22	43875001	43925000	0.349146	*HESX1*
22	43875001	43925000	0.349146	*APPL1*
18	43300001	43350000	0.349135	*CEP89*
19	22950001	23000000	0.349133	*RTN4RL1*
1	1700001	1750000	0.349118	*ITSN1*
8	99825001	99875000	0.349109	*TXNDC8*
3	79450001	79500000	0.349024	*MGC137454*
6	91175001	91225000	0.348976	*STBD1*
19	42900001	42950000	0.348875	*SAO*
19	42900001	42950000	0.348875	*G6PC1*
19	47125001	47175000	0.348862	*MARCHF10*
5	111475001	111525000	0.348823	*TNRC6B*

20	3700001	3750000	0.348809	*FBXW11*
23	13325001	13375000	0.348677	*KIF6*
19	43850001	43900000	0.348674	*LSM12*
19	43850001	43900000	0.348674	*NAGS*
19	43850001	43900000	0.348674	*TMEM101*
8	99900001	99950000	0.348673	*SVEP1*
25	25775001	25825000	0.34864	*SBK1*
10	85900001	85950000	0.348618	*AREL1*
11	10450001	10500000	0.348611	*MOB1A*
28	26425001	26475000	0.348564	*PPA1*
28	26425001	26475000	0.348564	*NPFFR1*
19	19100001	19150000	0.348504	*KSR1*
22	44775001	44825000	0.348476	*ERC2*
23	8300001	8350000	0.348451	*HMGA1*
23	8300001	8350000	0.348451	*SMIM29*
22	47175001	47225000	0.34842	*CACNA1D*
7	10450001	10500000	0.34842	*OR7A10B*
7	10450001	10500000	0.34842	*OR7A96*
7	10450001	10500000	0.34842	*OR7A91*
4	81225001	81275000	0.348392	*RALA*
6	58350001	58400000	0.348376	*WDR19*
19	43950001	44000000	0.348341	*HROB*
19	43950001	44000000	0.348341	*ASB16*
19	43950001	44000000	0.348341	*HDAC5*
19	11150001	11200000	0.348308	*INTS2*
19	11150001	11200000	0.348308	*MED13*
3	775001	825000	0.348266	*ADCY10*
3	775001	825000	0.348266	*MPC2*
15	41075001	41125000	0.348254	*GALNT18*
13	24350001	24400000	0.348246	*OTUD1*
22	43675001	43725000	0.348243	*DNAH12*
4	119425001	119475000	0.348234	*ESYT2*
19	52800001	52850000	0.348231	*ENPP7*
28	35400001	35450000	0.348221	*CGN1*
26	22125001	22175000	0.348173	*BTRC*
29	44500001	44550000	0.348136	*DPP3*
29	44500001	44550000	0.348136	*PELI3*
16	51025001	51075000	0.348126	*SSU72*
16	51025001	51075000	0.348126	*FNDC10*
2	15675001	15725000	0.348105	*UBE2E3*
7	50475001	50525000	0.348046	*SIL1*
7	90200001	90250000	0.348009	*ADGRV1*
10	1775001	1825000	0.347979	*EPB41L4A*
10	1775001	1825000	0.347979	*SNORA13*
10	27900001	27950000	0.347907	*OR4F13*
8	62000001	62050000	0.347899	*SHB*
4	74975001	75025000	0.347896	*TNS3*
2	125800001	125850000	0.347886	*WASF2*
2	22825001	22875000	0.347886	*SP3*
20	24225001	24275000	0.347881	*CSPG4B*
11	101600001	101650000	0.34787	*POMT1*
11	101600001	101650000	0.34787	*PRRC2B*

11	101600001	101650000	0.34787	*UCK1*
11	101600001	101650000	0.34787	*SNORD62*
11	101600001	101650000	0.34787	*SNORD62*
18	55825001	55875000	0.347858	*KASH5*
18	55825001	55875000	0.347858	*TEAD2*
18	55825001	55875000	0.347858	*DKKL1*
18	55825001	55875000	0.347858	*CD37*
19	44450001	44500000	0.347852	*DBF4B*
19	44450001	44500000	0.347852	*ADAM11*
8	91325001	91375000	0.34785	*GRIN3A*
23	17725001	17775000	0.347828	*SLC29A1*
23	17725001	17775000	0.347828	*NFKBIE*
23	17725001	17775000	0.347828	*HSP90AB1*
23	17725001	17775000	0.347828	*SLC35B2*
23	17725001	17775000	0.347828	*MYMX*
8	61800001	61850000	0.347827	*FRMPD1*
22	7275001	7325000	0.347799	*CCR4*
8	97675001	97725000	0.347787	*U6*
5	65425001	65475000	0.347743	*MYBPC1*
15	28575001	28625000	0.347701	*TMPRSS13*
29	50475001	50525000	0.347651	*CHID1*
29	50475001	50525000	0.347651	*AP2A2*
29	50475001	50525000	0.347651	*TSPAN4*
2	9700001	9750000	0.347649	*ITGAV*
20	700001	750000	0.347567	*SLIT3*
11	62925001	62975000	0.347524	*AFTPH*
4	10025001	10075000	0.347516	*CDK6*
4	47125001	47175000	0.347514	*SYPL1*
16	43500001	43550000	0.347508	*RBP7*
16	43500001	43550000	0.347508	*UBE4B*
13	54325001	54375000	0.347501	*YTHDF1*
23	13675001	13725000	0.347492	*DAAM2*
11	3625001	3675000	0.347478	*INPP4A*
8	87250001	87300000	0.347461	*DIRAS2*
8	76625001	76675000	0.347458	*FRMD3*
12	21425001	21475000	0.347415	*NEK5*
26	45675001	45725000	0.347383	*ADAM12*
11	9075001	9125000	0.347351	*MRPS9*
5	65550001	65600000	0.34735	*GNPTAB*
21	45975001	46025000	0.347333	*RALGAPA1*
21	45975001	46025000	0.347333	*BRMS1L*
21	45975001	46025000	0.347333	*U6*
3	78675001	78725000	0.347321	*SGIP1*
15	36450001	36500000	0.347313	*SOX6*
6	111825001	111875000	0.347281	*LDB2*
19	31325001	31375000	0.347268	*ARHGAP44*
19	31325001	31375000	0.347268	*ELAC2*
28	26175001	26225000	0.347252	*COL13A1*
11	6175001	6225000	0.347245	*RNF149*
11	6175001	6225000	0.347245	*CNOT11*
11	6175001	6225000	0.347245	*SNORD89*
4	80450001	80500000	0.347242	*SUGCT*

19	28625001	28675000	0.347224	*NTN1*
19	28625001	28675000	0.347224	*STX8*
16	71725001	71775000	0.347215	*NEK2*
19	30625001	30675000	0.34719	*MAP2K4*
19	30625001	30675000	0.34719	*ZNF18*
3	25325001	25375000	0.347187	*GDAP2*
2	8875001	8925000	0.347183	*CALCRL*
2	8875001	8925000	0.347183	*TFPI*
8	90725001	90775000	0.347138	*PLPPR1*
3	21375001	21425000	0.347112	*RBM8A*
3	21375001	21425000	0.347112	*TXNIP*
3	21375001	21425000	0.347112	*POLR3GL*
3	21375001	21425000	0.347112	*ANKRD34A*
26	33575001	33625000	0.3471	*TCF7L2*
6	91325001	91375000	0.347096	*SHROOM3*
22	37100001	37150000	0.347084	*PRICKLE2*
11	64775001	64825000	0.347084	*MEIS1*
19	47875001	47925000	0.347075	*DCAF7*
19	47875001	47925000	0.347075	*MAP3K3*
19	47875001	47925000	0.347075	*TACO1*
25	36400001	36450000	0.347062	*ZKSCAN1*
25	36400001	36450000	0.347062	*AZGP1*
8	61850001	61900000	0.347057	*FRMPD1*
8	61850001	61900000	0.347057	*TRMT10B*
8	61850001	61900000	0.347057	*EXOSC3*
1	153200001	153250000	0.347046	*GALNT15*
16	51100001	51150000	0.347029	*CCNL2*
16	51100001	51150000	0.347029	*VWA1*
16	51100001	51150000	0.347029	*TMEM88B*
16	51100001	51150000	0.347029	*MRPL20*
16	51100001	51150000	0.347029	*ANKRD65*
16	51100001	51150000	0.347029	*AURKAIP1*
16	51100001	51150000	0.347029	*ATAD3A*
3	93925001	93975000	0.347006	*PRPF38A*
3	93925001	93975000	0.347006	*ORC1*
3	93925001	93975000	0.347006	*TUT4*
6	30025001	30075000	0.346978	*PDLIM5*
8	11475001	11525000	0.346977	*NDUFB6*
8	11475001	11525000	0.346977	*TOPORS*
19	28750001	28800000	0.346962	*STX8*
3	16400001	16450000	0.346958	*NUP210L*
3	16400001	16450000	0.346958	*RPS27*
19	12300001	12350000	0.346923	*PPM1D*
2	56375001	56425000	0.346921	*LRP1B*
14	33550001	33600000	0.346907	*SLCO5A1*
19	50675001	50725000	0.346902	*CCDC57*
16	4075001	4125000	0.34687	*SRGAP2*
19	44275001	44325000	0.346854	*GPATCH8*
29	12475001	12525000	0.34685	*PCF11*
5	104100001	104150000	0.346848	*CD9*
15	39675001	39725000	0.346825	*RASSF10*
11	11725001	11775000	0.346786	*EXOC6B*

18	53000001	53050000	0.346744	*POLR1G*
18	53000001	53050000	0.346744	*PPP1R13L*
18	53000001	53050000	0.346744	*ERCC1*
18	53000001	53050000	0.346744	*ERCC2*
6	85075001	85125000	0.346734	*UGT2A1*
28	26700001	26750000	0.346704	*PALD1*
10	27725001	27775000	0.346693	*OR4G9*
10	27725001	27775000	0.346693	*OR4G8*
16	50925001	50975000	0.346684	*NADK*
16	50925001	50975000	0.346684	*SLC35E2*
16	50925001	50975000	0.346684	*GNB1*
24	54650001	54700000	0.346619	*TCF4*
24	54650001	54700000	0.346619	*U2*
4	7375001	7425000	0.34659	*ABCA13*
28	23575001	23625000	0.346582	*CTNNA3*
20	38000001	38050000	0.346557	*NADK2*
20	38000001	38050000	0.346557	*SKP2*
22	48450001	48500000	0.346553	*PHF7*
22	48450001	48500000	0.346553	*DNAH1*
22	48450001	48500000	0.346553	*BAP1*
22	48450001	48500000	0.346553	*SEMA3G*
12	61175001	61225000	0.346543	*HTATSF1*
14	575001	625000	0.34654	*HSF1*
14	575001	625000	0.34654	*DGAT1*
14	575001	625000	0.34654	*SCRT1*
14	575001	625000	0.34654	*FBXL6*
14	575001	625000	0.34654	*SLC52A2*
14	575001	625000	0.34654	*TMEM249*
2	20250001	20300000	0.346525	*U6*
4	43725001	43775000	0.34652	*U1*
5	47175001	47225000	0.346492	*GRIP1*
11	56700001	56750000	0.346469	*REG3A*
6	104050001	104100000	0.346439	*STK32B*
6	65625001	65675000	0.346378	*GABRB1*
19	11100001	11150000	0.346344	*MED13*
19	26900001	26950000	0.346289	*DLG4*
19	26900001	26950000	0.346289	*DVL2*
19	26900001	26950000	0.346289	*ACADVL*
19	26900001	26950000	0.346289	*PHF23*
12	31600001	31650000	0.34626	*FLT1*
3	110925001	110975000	0.34626	*GJB5*
3	110925001	110975000	0.34626	*GJB4*
23	10725001	10775000	0.346238	*CPNE5*
23	10725001	10775000	0.346238	*PPIL1*
11	55575001	55625000	0.346214	*CTNNA2*
3	78650001	78700000	0.346159	*SGIP1*
7	11725001	11775000	0.346158	*DCAF15*
7	11725001	11775000	0.346158	*CC2D1A*
7	11725001	11775000	0.346158	*PODNL1*
7	11725001	11775000	0.346158	*RFX1*
6	96175001	96225000	0.346124	*RASGEF1B*
6	96175001	96225000	0.346124	*SNORA70*

18	3400001	3450000	0.346118	*CNTNAP4*
28	15350001	15400000	0.346096	*SLC16A9*
15	36300001	36350000	0.346091	*SOX6*
19	39100001	39150000	0.34607	*ARHGAP23*
19	39100001	39150000	0.34607	*SRCIN1*
8	83150001	83200000	0.346044	*HABP4*
8	83150001	83200000	0.346044	*CDC14B*
16	72475001	72525000	0.346018	*KCNH1*
6	104150001	104200000	0.345991	*CYTL1*
15	29200001	29250000	0.345967	*ARCN1*
15	29200001	29250000	0.345967	*IFT46*
15	29200001	29250000	0.345967	*TMEM25*
22	58350001	58400000	0.345949	*FBLN2*
9	84000001	84050000	0.345934	*ADGB*
22	49600001	49650000	0.34593	*DOCK3*
22	30600001	30650000	0.345867	*FOXP1*
12	32700001	32750000	0.345785	*USP12*
12	32075001	32125000	0.345759	*CDX2*
12	32075001	32125000	0.345759	*PDX1*
12	32075001	32125000	0.345759	*URAD*
26	36450001	36500000	0.345755	*GFRA1*
11	100600001	100650000	0.345753	*HMCN2*
22	60275001	60325000	0.345753	*CHCHD6*
22	60275001	60325000	0.345753	*PLXNA1*
3	113275001	113325000	0.34575	*UGT1A6*
16	50550001	50600000	0.345699	*SKI*
16	50550001	50600000	0.345699	*FAAP20*
22	30475001	30525000	0.345671	*FOXP1*
6	91225001	91275000	0.345658	*CCDC158*
6	91225001	91275000	0.345658	*U6*
11	56675001	56725000	0.345642	*REG3A*
19	44650001	44700000	0.345639	*C1QL1*
19	44650001	44700000	0.345639	*KIF18B*
8	85775001	85825000	0.345614	*SPTLC1*
11	78175001	78225000	0.345596	*HS1BP3*
11	78175001	78225000	0.345596	*LDAH*
11	78175001	78225000	0.345596	*GDF7*
8	98700001	98750000	0.345568	*FRRS1L*
8	98700001	98750000	0.345568	*EPB41L4B*
21	65275001	65325000	0.345547	*WDR25*
6	110475001	110525000	0.345518	*C1QTNF7*
18	52900001	52950000	0.34542	*MARK4*
18	52900001	52950000	0.34542	*EXOC3L2*
18	52900001	52950000	0.34542	*U6*
14	31725001	31775000	0.345419	*CPA6*
19	29050001	29100000	0.345408	*GAS7*
23	37325001	37375000	0.345401	*CDKAL1*
21	45375001	45425000	0.345397	*PRORP*
21	45375001	45425000	0.345397	*PPP2R3C*
22	53675001	53725000	0.345343	*LIMD1*
1	144050001	144100000	0.345328	*TRAPPC10*
22	51200001	51250000	0.34529	*CELSR3*

22	51200001	51250000	0.34529	*NCKIPSD*
22	51200001	51250000	0.34529	*SLC26A6*
22	7175001	7225000	0.345285	*TRIM71*
24	48225001	48275000	0.345263	*CTIF*
12	18700001	18750000	0.345232	*FNDC3A*
4	4550001	4600000	0.345215	*COBL*
19	30550001	30600000	0.345201	*DNAH9*
28	7175001	7225000	0.345201	*TARBP1*
28	7175001	7225000	0.345201	*COA6*
11	19725001	19775000	0.345184	*PRKD3*
14	1300001	1350000	0.345153	*GLI4*
14	1300001	1350000	0.345153	*ZNF696*
14	1300001	1350000	0.345153	*TOP1MT*
14	1300001	1350000	0.345153	*GPIHBP1*
16	65075001	65125000	0.345137	*TSEN15*
16	65075001	65125000	0.345137	*COLGALT2*
19	27050001	27100000	0.345119	*NLGN2*
19	27050001	27100000	0.345119	*TNK1*
19	27050001	27100000	0.345119	*KCTD11*
19	27050001	27100000	0.345119	*PLSCR3*
19	27050001	27100000	0.345119	*TMEM95*
19	27050001	27100000	0.345119	*TMEM256*
19	27050001	27100000	0.345119	*SPEM1*
14	23300001	23350000	0.345112	*PLAG1*
14	23300001	23350000	0.345112	*MOS*
14	23325001	23375000	0.345106	*PLAG1*
29	26650001	26700000	0.345087	*OR10G9G*
8	90325001	90375000	0.345045	*CAVIN4*
8	90325001	90375000	0.345045	*TMEFF1*
19	47725001	47775000	0.345037	*TANC2*
19	47725001	47775000	0.345037	*CYB561*
22	36900001	36950000	0.345022	*ADAMTS9*
19	11175001	11225000	0.344981	*INTS2*
1	19475001	19525000	0.344974	*CXADR*
3	550001	600000	0.344967	*DCAF6*
3	550001	600000	0.344967	*GPR161*
11	44875001	44925000	0.344952	*SULT1C3*
11	44875001	44925000	0.344952	*SULT1C2*
21	47775001	47825000	0.344901	*TTC6*
21	47775001	47825000	0.344901	*FOXA1*
3	22950001	23000000	0.344829	*PDE4DIP*
9	43325001	43375000	0.344804	*RTN4IP1*
9	43325001	43375000	0.344804	*QRSL1*
15	83425001	83475000	0.344802	*NCAPD3*
11	20625001	20675000	0.34477	*ATL2*
22	44200001	44250000	0.344764	*ARHGEF3*
16	79575001	79625000	0.344764	*CACNA1S*
16	79575001	79625000	0.344764	*KIF21B*
11	104450001	104500000	0.344737	*FAM163B*
11	104450001	104500000	0.344737	*DBH*
1	149700001	149750000	0.344711	*DYRK1A*
3	120050001	120100000	0.344688	*KIF1A*

21	25000001	25050000	0.344686	*BTBD1*
2	7325001	7375000	0.344672	*COL5A2*
1	144650001	144700000	0.344644	*UBE2G2*
1	144650001	144700000	0.344644	*SUMO3*
8	10900001	10950000	0.34464	*SCARA5*
8	11325001	11375000	0.34464	*TMEM215*
8	10900001	10950000	0.34464	*U6*
19	50525001	50575000	0.344614	*CD7*
28	44175001	44225000	0.344608	*ZFAND4*
5	110700001	110750000	0.344601	*RPL3*
5	110700001	110750000	0.344601	*SNORD83B*
5	110700001	110750000	0.344601	*SNORD83A*
2	43425001	43475000	0.344593	*ARL6IP6*
2	43425001	43475000	0.344593	*PRPF40A*
2	43425001	43475000	0.344593	*U6*
5	48925001	48975000	0.344577	*TBC1D30*
19	26775001	26825000	0.344546	*ALOX12*
19	26775001	26825000	0.344546	*BCL6B*
19	26775001	26825000	0.344546	*C19H17orf49*
19	26775001	26825000	0.344546	*SLC16A13*
19	26775001	26825000	0.344546	*RNASEK*
20	41950001	42000000	0.344531	*C20H5orf22*
20	41950001	42000000	0.344531	*DROSHA*
4	84750001	84800000	0.344506	*KCND2*
3	21350001	21400000	0.3445	*HJV*
3	21350001	21400000	0.3445	*TXNIP*
3	21350001	21400000	0.3445	*POLR3GL*
16	49350001	49400000	0.344474	*TP73*
5	108550001	108600000	0.344467	*CACNA1C*
25	13800001	13850000	0.344459	*MPV17L*
28	22750001	22800000	0.344452	*CTNNA3*
25	3600001	3650000	0.344443	*HMOX2*
25	3600001	3650000	0.344443	*NMRAL1*
25	3600001	3650000	0.344443	*DNAJA3*
7	8550001	8600000	0.344377	*OR7A117*
22	41425001	41475000	0.344357	*FHIT*
7	42150001	42200000	0.344348	*OR2G6*
15	22000001	22050000	0.34434	*SIK2*
19	26200001	26250000	0.344285	*RABEP1*
4	68025001	68075000	0.344278	*JAZF1*
3	79775001	79825000	0.344239	*LEPR*
15	74925001	74975000	0.344228	*TSPAN18*
6	94725001	94775000	0.344134	*ANTXR2*
4	119475001	119525000	0.344065	*ESYT2*
19	33400001	33450000	0.344062	*NCOR1*
19	33400001	33450000	0.344062	*TTC19*
22	43100001	43150000	0.344013	*FLNB*
22	43100001	43150000	0.344013	*DNASE1L3*
24	34400001	34450000	0.343981	*MIB1*
21	47425001	47475000	0.343968	*MIPOL1*
11	80950001	81000000	0.343965	*VSNL1*
13	22825001	22875000	0.343922	*MLLT10*

1	152400001	152450000	0.343905	*METTL6*
1	2475001	2525000	0.343874	*OLIG1*
5	112600001	112650000	0.343868	*ACO2*
5	112600001	112650000	0.343868	*POLR3H*
5	112600001	112650000	0.343868	*CSDC2*
5	47300001	47350000	0.343856	*GRIP1*
11	74525001	74575000	0.34385	*NCOA1*
12	17800001	17850000	0.343827	*SUCLA2*
14	36175001	36225000	0.343793	*KCNB2*
16	37600001	37650000	0.343776	*KIFAP3*
13	72050001	72100000	0.343771	*SRSF6*
19	50050001	50100000	0.343735	*FOXK2*
1	143925001	143975000	0.343728	*AGPAT3*
8	69375001	69425000	0.343719	*DMTN*
8	69375001	69425000	0.343719	*FHIP2B*
8	69375001	69425000	0.343719	*NUDT18*
6	58300001	58350000	0.343715	*KLHL5*
1	2050001	2100000	0.343656	*TMEM50B*
1	2050001	2100000	0.343656	*IFNGR2*
23	10775001	10825000	0.343653	*C23H6orf89*
23	10775001	10825000	0.343653	*PPIL1*
8	99700001	99750000	0.343643	*PALM2*
8	99700001	99750000	0.343643	*C8H9orf152*
20	35275001	35325000	0.343631	*FYB1*
20	38475001	38525000	0.343558	*SPEF2*
28	25625001	25675000	0.343532	*HKDC1*
24	56950001	57000000	0.343515	*ATP8B1*
28	44575001	44625000	0.343495	*RASSF4*
28	44575001	44625000	0.343495	*ZNF22*
28	44575001	44625000	0.343495	*DEPP1*
14	75325001	75375000	0.343482	*CNBD1*
19	45975001	46025000	0.34346	*MAPT*
19	45975001	46025000	0.34346	*KANSL1*
11	104600001	104650000	0.343443	*VAV2*
5	108525001	108575000	0.343442	*CACNA1C*
13	23025001	23075000	0.343412	*DNAJC1*
15	54625001	54675000	0.343407	*GDPD5*
14	34775001	34825000	0.343372	*EYA1*
14	36600001	36650000	0.343351	*RDH10*
14	36600001	36650000	0.343351	*C14H8orf89*
14	36600001	36650000	0.343351	*RPL7*
22	45100001	45150000	0.34335	*ERC2*
1	42300001	42350000	0.343335	*OR5AC31*
3	19575001	19625000	0.343333	*PIP5K1A*
3	19575001	19625000	0.343333	*VPS72*
18	12925001	12975000	0.343318	*FBXO31*
18	12925001	12975000	0.343318	*MAP1LC3B*
4	39975001	40025000	0.343315	*SEMA3C*
14	66375001	66425000	0.343312	*LAPTM4B*
20	30475001	30525000	0.343215	*FGF10*
7	90175001	90225000	0.343118	*ADGRV1*
19	33450001	33500000	0.343083	*TTC19*

19	33450001	33500000	0.343083	*ADORA2B*
19	33450001	33500000	0.343083	*ZSWIM7*
3	94100001	94150000	0.343056	*ZFYVE9*
22	47700001	47750000	0.343001	*TKT*
22	47700001	47750000	0.343001	*PRKCD*
5	111500001	111550000	0.342995	*TNRC6B*
20	40150001	40200000	0.342995	*ADAMTS12*
16	39600001	39650000	0.342976	*DNM3*
22	49500001	49550000	0.342976	*DOCK3*
10	75025001	75075000	0.342954	*KCNH5*
15	37775001	37825000	0.342931	*CALCB*
5	109250001	109300000	0.342889	*MICAL3*
8	7500001	7550000	0.342866	*DEFB136*
21	1250001	1300000	0.342845	*NDN*
22	60425001	60475000	0.342804	*TXNRD3*
22	60425001	60475000	0.342804	*UROC1*
22	60425001	60475000	0.342804	*CHST13*
22	60425001	60475000	0.342804	*C22H3orf22*
10	89025001	89075000	0.342792	*ADCK1*
21	45550001	45600000	0.342771	*PSMA6*
13	61375001	61425000	0.342765	*TTLL9*
13	61375001	61425000	0.342765	*DUSP15*
13	61375001	61425000	0.342765	*FOXS1*
18	10650001	10700000	0.342754	*ATP2C2*
18	10650001	10700000	0.342754	*MEAK7*
19	27700001	27750000	0.342716	*ALOX12B*
19	27700001	27750000	0.342716	*ALOXE3*
19	27700001	27750000	0.342716	*ALOX15B*
13	17325001	17375000	0.342706	*PFKFB3*
29	39350001	39400000	0.342699	*PAG6*
29	26625001	26675000	0.342673	*OR10G9G*
29	26625001	26675000	0.342673	*OR10G6*
11	48675001	48725000	0.342643	*IMMT*
14	1000001	1050000	0.342639	*IQANK1*
14	1000001	1050000	0.342639	*FAM83H*
14	1000001	1050000	0.342639	*MAPK15*
14	1000001	1050000	0.342639	*SCRIB*
21	28175001	28225000	0.342634	*FAM189A1*
7	43700001	43750000	0.342632	*EFNA2*
7	43700001	43750000	0.342632	*CIRBP*
7	43700001	43750000	0.342632	*FAM174C*
6	67525001	67575000	0.342593	*OCIAD2*
14	14925001	14975000	0.342573	*NSMCE2*
19	33375001	33425000	0.34256	*NCOR1*
28	42975001	43025000	0.342529	*ARHGAP22*
28	42975001	43025000	0.342529	*WDFY4*
8	8125001	8175000	0.342509	*XKR6*
8	8125001	8175000	0.342509	*Metazoa_SRP*
26	39325001	39375000	0.342482	*GRK5*
26	39325001	39375000	0.342482	*PRDX3*
26	39325001	39375000	0.342482	*SFXN4*
16	38500001	38550000	0.342474	*MROH9*

22	43850001	43900000	0.342402	*APPL1*
22	6925001	6975000	0.342402	*CMTM6*
22	43850001	43900000	0.342402	*HESX1*
22	6925001	6975000	0.342402	*DYNC1LI1*
16	39575001	39625000	0.342397	*DNM3*
15	50825001	50875000	0.342383	*OR52B4L*
3	98350001	98400000	0.342374	*TRABD2B*
13	12275001	12325000	0.342373	*UPF2*
13	12275001	12325000	0.342373	*DHTKD1*
4	85600001	85650000	0.342355	*CPED1*
5	108625001	108675000	0.342332	*CACNA1C*
1	144275001	144325000	0.342315	*PFKL*
1	144275001	144325000	0.342315	*AIRE*
1	143975001	144025000	0.342313	*AGPAT3*
26	4800001	4850000	0.342312	*PCDH15*
19	27800001	27850000	0.342268	*AURKB*
19	27800001	27850000	0.342268	*TMEM107*
19	27800001	27850000	0.342268	*BORCS6*
19	27800001	27850000	0.342268	*U8*
7	110250001	110300000	0.342232	*CAMK4*
22	52675001	52725000	0.342227	*PRSS42*
2	107200001	107250000	0.342223	*PTPRN*
2	107200001	107250000	0.342223	*DNAJB2*
2	107200001	107250000	0.342223	*TUBA1D*
2	107200001	107250000	0.342223	*TUBA4A*
8	64925001	64975000	0.342199	*STX17*
18	14575001	14625000	0.342184	*FANCA*
18	14575001	14625000	0.342184	*ZNF276*
18	14575001	14625000	0.342184	*VPS9D1*
18	14575001	14625000	0.342184	*SPATA2L*
18	14575001	14625000	0.342184	*CDK10*
18	52800001	52850000	0.342182	*NKPD1*
18	52800001	52850000	0.342182	*TRAPPC6A*
18	52800001	52850000	0.342182	*PPP1R37*
18	52800001	52850000	0.342182	*BLOC1S3*
23	15375001	15425000	0.342162	*FOXP4*
1	152550001	152600000	0.342153	*HACL1*
6	57975001	58025000	0.342102	*KLF3*
12	86600001	86650000	0.3421	*GRTP1*
12	86600001	86650000	0.3421	*LAMP1*
23	16175001	16225000	0.342099	*U6*
22	31350001	31400000	0.342087	*MDFIC2*
1	45050001	45100000	0.342083	*ADGRG7*
1	45050001	45100000	0.342083	*TMEM45A*
5	116275001	116325000	0.342073	*WNT7B*
10	88575001	88625000	0.342068	*NOXRED1*
10	88575001	88625000	0.342068	*SAMD15*
10	88575001	88625000	0.342068	*TMED8*
10	88575001	88625000	0.342068	*VIPAS39*
28	24675001	24725000	0.342046	*PBLD*
28	24675001	24725000	0.342046	*MYPN*
28	24675001	24725000	0.342046	*ATOH7*

27	34950001	35000000	0.342029	*ADAM18*
2	125000001	125050000	0.342027	*PHACTR4*
11	77925001	77975000	0.34202	*APOB*
19	26175001	26225000	0.342019	*RABEP1*
16	3625001	3675000	0.341992	*SLC26A9*
16	3625001	3675000	0.341992	*PM20D1*
2	16775001	16825000	0.341991	*CWC22*
10	80000001	80050000	0.34198	*RAD51B*
24	75001	125000	0.341979	*OR9M1*
24	75001	125000	0.341979	*OR5W32P*
5	112050001	112100000	0.341947	*SLC25A17*
5	112050001	112100000	0.341947	*ST13*
5	112050001	112100000	0.341947	*U6*
14	31675001	31725000	0.341913	*CPA6*
5	77725001	77775000	0.341896	*BICD1*
22	43050001	43100000	0.341894	*ABHD6*
22	43050001	43100000	0.341894	*DNASE1L3*
3	101400001	101450000	0.341868	*RNF220*
28	24750001	24800000	0.341857	*RUFY2*
28	24750001	24800000	0.341857	*HNRNPH3*
25	24750001	24800000	0.341849	*KDM8*
1	149850001	149900000	0.341834	*KCNJ6*
9	42600001	42650000	0.34183	*PDSS2*
9	84025001	84075000	0.341821	*ADGB*
7	43350001	43400000	0.341786	*PTBP1*
7	43350001	43400000	0.341786	*PLPPR3*
7	43350001	43400000	0.341786	*AZU1*
7	43350001	43400000	0.341786	*U6*
13	61500001	61550000	0.34174	*CCM2L*
13	61500001	61550000	0.34174	*XKR7*
4	119075001	119125000	0.34169	*PTPRN2*
24	19575001	19625000	0.341667	*CELF4*
6	89925001	89975000	0.341653	*PARM1*
14	31075001	31125000	0.341647	*MCMDC2*
14	31075001	31125000	0.341647	*SNORD87*
22	37075001	37125000	0.341616	*PRICKLE2*
7	43675001	43725000	0.341585	*MIDN*
7	43675001	43725000	0.341585	*CIRBP*
7	43675001	43725000	0.341585	*CBARP*
7	43675001	43725000	0.341585	*FAM174C*
7	43675001	43725000	0.341585	*EFNA2*
7	43675001	43725000	0.341585	*ATP5F1D*
18	53375001	53425000	0.341573	*MYPOP*
18	53375001	53425000	0.341573	*FOXA3*
18	53375001	53425000	0.341573	*SYMPK*
18	53375001	53425000	0.341573	*NANOS2*
19	26925001	26975000	0.341558	*DVL2*
19	26925001	26975000	0.341558	*ELP5*
19	26925001	26975000	0.341558	*DLG4*
19	26925001	26975000	0.341558	*CTDNEP1*
19	26925001	26975000	0.341558	*ACADVL*
19	26925001	26975000	0.341558	*PHF23*

19	26925001	26975000	0.341558	*CLDN7*
19	26925001	26975000	0.341558	*GABARAP*
4	59925001	59975000	0.341515	*ELMO1*
13	62600001	62650000	0.341482	*BPIFA2B*
18	14525001	14575000	0.341477	*DPEP1*
18	14525001	14575000	0.341477	*SPATA33*
18	14525001	14575000	0.341477	*CHMP1A*
18	14525001	14575000	0.341477	*CDK10*
5	65400001	65450000	0.341474	*MYBPC1*
8	71475001	71525000	0.341445	*ADAM28*
5	5825001	5875000	0.341443	*OSBPL8*
5	5825001	5875000	0.341443	*BBS10*
2	106950001	107000000	0.341439	*CFAP65*
11	46975001	47025000	0.341434	*PAX8*
5	69675001	69725000	0.341428	*TCP11L2*
5	69675001	69725000	0.341428	*POLR3B*
6	80800001	80850000	0.341395	*EPHA5*
6	24175001	24225000	0.341372	*EMCN*
18	14300001	14350000	0.34134	*ANKRD11*
3	23950001	24000000	0.341329	*WARS2*
13	73700001	73750000	0.341328	*DBNDD2*
13	73700001	73750000	0.341328	*SYS1*
13	73700001	73750000	0.341328	*TP53TG5*
13	73700001	73750000	0.341328	*PIGT*
15	60950001	61000000	0.341301	*FSHB*
15	63925001	63975000	0.341242	*HIPK3*
25	34100001	34150000	0.341231	*RHBDD2*
25	34100001	34150000	0.341231	*POR*
12	60650001	60700000	0.341203	*SLITRK6*
7	22125001	22175000	0.341194	*PDLIM4*
7	22125001	22175000	0.341194	*SLC22A4*
7	50875001	50925000	0.341193	*CXXC5*
1	2450001	2500000	0.341114	*OLIG1*
6	64625001	64675000	0.341113	*GABRG1*
22	35975001	36025000	0.341113	*MAGI1*
19	41050001	41100000	0.341055	*KRT20*
19	41050001	41100000	0.341055	*KRT12*
25	24975001	25025000	0.341045	*IL21R*
25	24975001	25025000	0.341045	*GTF3C1*
24	56725001	56775000	0.341026	*ONECUT2*
1	109200001	109250000	0.341024	*RSRC1*
22	44525001	44575000	0.340982	*ERC2*
7	44175001	44225000	0.340972	*ABHD17A*
7	44175001	44225000	0.340972	*KLF16*
7	44175001	44225000	0.340972	*SCAMP4*
24	19600001	19650000	0.340962	*CELF4*
5	58975001	59025000	0.340958	*OR6C74*
18	53100001	53150000	0.340956	*VASP*
18	53100001	53150000	0.340956	*PPM1N*
12	33525001	33575000	0.340952	*ATP8A2*
22	44500001	44550000	0.340939	*ERC2*
26	45175001	45225000	0.340934	*TEX36*

6	94750001	94800000	0.340913	*ANTXR2*
1	152725001	152775000	0.340881	*ANKRD28*
18	15500001	15550000	0.340877	*NETO2*
8	64875001	64925000	0.340873	*NR4A3*
3	78550001	78600000	0.340855	*DYNLT5*
3	78550001	78600000	0.340855	*SGIP1*
8	8875001	8925000	0.34083	*MSRA*
13	17250001	17300000	0.34082	*PFKFB3*
15	45275001	45325000	0.340819	*SYT9*
4	119050001	119100000	0.340804	*PTPRN2*
10	80825001	80875000	0.340801	*DCAF5*
3	40300001	40350000	0.340792	*COL11A1*
11	101400001	101450000	0.340778	*NUP214*
5	104025001	104075000	0.340776	*PLEKHG6*
5	104025001	104075000	0.340776	*TNFRSF1A*
22	57375001	57425000	0.340746	*FGD5*
20	67875001	67925000	0.340689	*ADAMTS16*
15	40825001	40875000	0.340687	*USP47*
15	40825001	40875000	0.340687	*Metazoa_SRP*
28	8475001	8525000	0.340673	*LYST*
3	19400001	19450000	0.34066	*SELENBP1*
25	25125001	25175000	0.340657	*KATNIP*
