# Supplementary material for: Identification of genomic diversity and selection signatures in Luxi cattle using whole-genome sequencing data
Source: Anim Biosci. 2024 Jan 20;37(3):461–70. doi: 10.5713/ab.23.0304 (PMC10915192; doi:10.5713/ab.23.0304)
Supplement: Supplementary file 7 [file ab-23-0304-Supplementary-Table-S7.pdf]

**Supplementary Table S7.** A summary of genes from XP-EHH in LUX.

| Chromosome | Starting position | Ending position | Xp-EHH | Gene |
|---|---|---|---|---|
| 7 | 43750001 | 43800000 | 4.3863 | *PWWP3A* |
| 21 | 2850001 | 2900000 | 4.242 | *ATP10A* |
| 20 | 500001 | 550000 | 4.1761 | *SLIT3* |
| 10 | 32400001 | 32450000 | 4.1333 | *CDIN1* |
| 1 | 2500001 | 2550000 | 4.0907 | *OLIG2* |
| 7 | 90800001 | 90850000 | 4.0823 | *ARRDC3* |
| 2 | 37200001 | 37250000 | 4.0773 | *TANC1* |
| 7 | 43800001 | 43850000 | 3.9619 | *DAZAP1* |
| 7 | 43800001 | 43850000 | 3.9619 | *NDUFS7* |
| 7 | 43800001 | 43850000 | 3.9619 | *PWWP3A* |
| 7 | 43800001 | 43850000 | 3.9619 | *GAMT* |
| 7 | 43800001 | 43850000 | 3.9619 | *RPS15* |
| 13 | 63900001 | 63950000 | 3.9549 | *PIGU* |
| 13 | 63900001 | 63950000 | 3.9549 | *DYNLRB1* |
| 13 | 63900001 | 63950000 | 3.9549 | *MAP1LC3A* |
| 21 | 2900001 | 2950000 | 3.9535 | *ATP10A* |
| 21 | 2900001 | 2950000 | 3.9535 | *U6* |
| 23 | 10900001 | 10950000 | 3.9353 | *FGD2* |
| 7 | 90850001 | 90900000 | 3.9219 | *ARRDC3* |
| 1 | 2450001 | 2500000 | 3.9129 | *OLIG1* |
| 7 | 43700001 | 43750000 | 3.8287 | *EFNA2* |
| 7 | 43700001 | 43750000 | 3.8287 | *CIRBP* |
| 7 | 43700001 | 43750000 | 3.8287 | *FAM174C* |
| 7 | 43850001 | 43900000 | 3.7642 | *APC2* |
| 7 | 43850001 | 43900000 | 3.7642 | *PCSK4* |
| 7 | 43850001 | 43900000 | 3.7642 | *C7H19orf25* |
| 7 | 43850001 | 43900000 | 3.7642 | *REEP6* |
| 13 | 63950001 | 64000000 | 3.7413 | *PIGU* |
| 2 | 37250001 | 37300000 | 3.7316 | *TANC1* |
| 13 | 64350001 | 64400000 | 3.7275 | *TRPC4AP* |
| 13 | 64350001 | 64400000 | 3.7275 | *EDEM2* |
| 26 | 26000001 | 26050000 | 3.7252 | *SORCS3* |
| 14 | 14900001 | 14950000 | 3.7231 | *NSMCE2* |
| 23 | 10850001 | 10900000 | 3.7059 | *MTCH1* |
| 23 | 10850001 | 10900000 | 3.7059 | *PI16* |
| 13 | 64200001 | 64250000 | 3.6995 | *ACSS2* |
| 13 | 64200001 | 64250000 | 3.6995 | *GSS* |
| 20 | 400001 | 450000 | 3.693 | *SLIT3* |
| 13 | 64400001 | 64450000 | 3.6848 | *EDEM2* |
| 13 | 64400001 | 64450000 | 3.6848 | *PROCR* |
| 13 | 63850001 | 63900000 | 3.6798 | *ITCH* |
| 13 | 63850001 | 63900000 | 3.6798 | *DYNLRB1* |
| 12 | 12700001 | 12750000 | 3.6667 | *TNFSF11* |
| 13 | 63800001 | 63850000 | 3.6551 | *ITCH* |
| 13 | 64550001 | 64600000 | 3.6472 | *UQCC1* |
| 13 | 64550001 | 64600000 | 3.6472 | *FAM83C* |
| 13 | 64550001 | 64600000 | 3.6472 | *EIF6* |
| 13 | 64500001 | 64550000 | 3.6451 | *MMP24* |
| 13 | 64500001 | 64550000 | 3.6451 | *EIF6* |
| 2 | 38800001 | 38850000 | 3.6419 | *ACVR1C* |

| | | | | |
|---|---|---|---|---|
| 13 | 64000001 | 64050000 | 3.6395 | *PIGU* |
| 13 | 64000001 | 64050000 | 3.6395 | *TP53INP2* |
| 2 | 37300001 | 37350000 | 3.63 | *TANC1* |
| 14 | 14750001 | 14800000 | 3.6186 | *TRIB1* |
| 21 | 2750001 | 2800000 | 3.6005 | *ATP10A* |
| 3 | 117600001 | 117650000 | 3.5876 | *TRAF3IP1* |
| 3 | 117550001 | 117600000 | 3.5821 | *PER2* |
| 3 | 117550001 | 117600000 | 3.5821 | *HES6* |
| 4 | 31650001 | 31700000 | 3.5766 | *FAM126A* |
| 15 | 40450001 | 40500000 | 3.5736 | *MICAL2* |
| 4 | 64650001 | 64700000 | 3.5621 | *PDE1C* |
| 18 | 50450001 | 50500000 | 3.5563 | *HNRNPUL1* |
| 18 | 50450001 | 50500000 | 3.5563 | *TGFB1* |
| 18 | 50450001 | 50500000 | 3.5563 | *CCDC97* |
| 3 | 117650001 | 117700000 | 3.5439 | *TRAF3IP1* |
| 14 | 14800001 | 14850000 | 3.5421 | *NSMCE2* |
| 13 | 64300001 | 64350000 | 3.5315 | *TRPC4AP* |
| 13 | 64300001 | 64350000 | 3.5315 | *MYH7B* |
| 4 | 64600001 | 64650000 | 3.5244 | *PDE1C* |
| 18 | 51900001 | 51950000 | 3.5097 | *SMG9* |
| 18 | 51900001 | 51950000 | 3.5097 | *IRGC* |
| 20 | 20450001 | 20500000 | 3.4887 | *RAB3C* |
| 19 | 10550001 | 10600000 | 3.4783 | *DHX40* |
| 19 | 10550001 | 10600000 | 3.4783 | *CLTC* |
| 4 | 64550001 | 64600000 | 3.4716 | *PDE1C* |
| 13 | 64150001 | 64200000 | 3.4664 | *GGT7* |
| 13 | 64150001 | 64200000 | 3.4664 | *ACSS2* |
| 14 | 15050001 | 15100000 | 3.4641 | *NSMCE2* |
| 14 | 15050001 | 15100000 | 3.4641 | *WASHC5* |
| 7 | 44000001 | 44050000 | 3.46 | *TCF3* |
| 8 | 90700001 | 90750000 | 3.4426 | *PLPPR1* |
| 14 | 14850001 | 14900000 | 3.4056 | *NSMCE2* |
| 20 | 450001 | 500000 | 3.4041 | *SLIT3* |
| 3 | 108850001 | 108900000 | 3.401 | *GRIK3* |
| 20 | 20400001 | 20450000 | 3.3977 | *RAB3C* |
| 15 | 40300001 | 40350000 | 3.3968 | *PARVA* |
| 18 | 14100001 | 14150000 | 3.3922 | *CBFA2T3* |
| 12 | 61150001 | 61200000 | 3.3878 | *HTATSF1* |
| 21 | 6400001 | 6450000 | 3.3861 | *ADAMTS17* |
| 9 | 15450001 | 15500000 | 3.3716 | *MYO6* |
| 3 | 108950001 | 109000000 | 3.3695 | *GRIK3* |
| 2 | 22850001 | 22900000 | 3.3474 | *SP3* |
| 21 | 2800001 | 2850000 | 3.3442 | *ATP10A* |
| 7 | 43650001 | 43700000 | 3.3295 | *STK11* |
| 7 | 43650001 | 43700000 | 3.3295 | *MIDN* |
| 7 | 43650001 | 43700000 | 3.3295 | *CBARP* |
| 7 | 43650001 | 43700000 | 3.3295 | *ATP5F1D* |
| 21 | 6350001 | 6400000 | 3.328 | *ADAMTS17* |
| 19 | 10600001 | 10650000 | 3.3222 | *CLTC* |
| 23 | 37250001 | 37300000 | 3.3063 | *CDKAL1* |
| 7 | 43950001 | 44000000 | 3.3059 | *TCF3* |
| 7 | 43950001 | 44000000 | 3.3059 | *MBD3* |

| | | | | |
|---|---|---|---|---|
| 7 | 43950001 | 44000000 | 3.3059 | *MEX3D* |
| 7 | 43950001 | 44000000 | 3.3059 | *UQCR11* |
| 17 | 65150001 | 65200000 | 3.2873 | *KIAA1671* |
| 25 | 13850001 | 13900000 | 3.2845 | *BMERB1* |
| 10 | 32350001 | 32400000 | 3.2788 | *CDIN1* |
| 18 | 13100001 | 13150000 | 3.2505 | *JPH3* |
| 14 | 23000001 | 23050000 | 3.2438 | *TMEM68* |
| 14 | 22950001 | 23000000 | 3.2432 | *XKR4* |
| 3 | 108900001 | 108950000 | 3.2402 | *GRIK3* |
| 18 | 50400001 | 50450000 | 3.2378 | *AXL* |
| 18 | 50400001 | 50450000 | 3.2378 | *HNRNPUL1* |
| 5 | 69450001 | 69500000 | 3.2263 | *NUAK1* |
| 10 | 32450001 | 32500000 | 3.2125 | *CDIN1* |
| 11 | 81000001 | 81050000 | 3.211 | *VSNL1* |
| 13 | 64100001 | 64150000 | 3.211 | *NCOA6* |
| 24 | 55950001 | 56000000 | 3.207 | *TXNL1* |
| 15 | 40700001 | 40750000 | 3.2069 | *DKK3* |
| 15 | 40700001 | 40750000 | 3.2069 | *USP47* |
| 2 | 36100001 | 36150000 | 3.1905 | *ITGB6* |
| 6 | 88600001 | 88650000 | 3.1885 | *RASSF6* |
| 26 | 24950001 | 25000000 | 3.1794 | *CFAP58* |
| 15 | 40400001 | 40450000 | 3.1618 | *MICAL2* |
| 8 | 90200001 | 90250000 | 3.1589 | *MSANTD3* |
| 8 | 90200001 | 90250000 | 3.1589 | *TMEFF1* |
| 20 | 14750001 | 14800000 | 3.1562 | *SHISAL2B* |
| 20 | 14750001 | 14800000 | 3.1562 | *SREK1IP1* |
| 13 | 64050001 | 64100000 | 3.1536 | *NCOA6* |
| 14 | 24550001 | 24600000 | 3.1398 | *UBXN2B* |
| 18 | 50500001 | 50550000 | 3.1321 | *EXOSC5* |
| 18 | 50500001 | 50550000 | 3.1321 | *BCKDHA* |
| 18 | 50500001 | 50550000 | 3.1321 | *TGFB1* |
| 18 | 50500001 | 50550000 | 3.1321 | *B9D2* |
| 18 | 50500001 | 50550000 | 3.1321 | *TMEM91* |
| 14 | 14950001 | 15000000 | 3.1251 | *NSMCE2* |
| 7 | 90650001 | 90700000 | 3.1235 | *ADGRV1* |
| 16 | 43550001 | 43600000 | 3.1115 | *NMNAT1* |
| 16 | 43550001 | 43600000 | 3.1115 | *RBP7* |
| 15 | 41050001 | 41100000 | 3.1035 | *GALNT18* |
| 16 | 43150001 | 43200000 | 3.0924 | *PGD* |
| 1 | 2700001 | 2750000 | 3.0804 | *C1H21orf62* |
| 14 | 22750001 | 22800000 | 3.0725 | *XKR4* |
| 14 | 43300001 | 43350000 | 3.0725 | *TPD52* |
| 28 | 15500001 | 15550000 | 3.0641 | *CCDC6* |
| 5 | 69500001 | 69550000 | 3.0565 | *NUAK1* |
| 5 | 66300001 | 66350000 | 3.055 | *U6* |
| 2 | 18850001 | 18900000 | 3.0501 | *PDE11A* |
| 24 | 55900001 | 55950000 | 3.0277 | *TXNL1* |
| 14 | 24400001 | 24450000 | 3.0266 | *FAM110B* |
| 4 | 31550001 | 31600000 | 3.0235 | *SNORD93* |
| 20 | 14700001 | 14750000 | 3.0172 | *CWC27* |
| 20 | 14700001 | 14750000 | 3.0172 | *SREK1IP1* |
| 7 | 43900001 | 43950000 | 3.0114 | *ADAMTSL5* |

| | | | | |
|---|---|---|---|---|
| 7 | 43900001 | 43950000 | 3.0114 | *MEX3D* |
| 7 | 43900001 | 43950000 | 3.0114 | *REEP6* |
| 15 | 63250001 | 63300000 | 3.0041 | *EIF3M* |
| 7 | 43600001 | 43650000 | 2.9953 | *SBNO2* |
| 18 | 51850001 | 51900000 | 2.9831 | *PLAUR* |
| 18 | 51850001 | 51900000 | 2.9831 | *CADM4* |
| 7 | 21600001 | 21650000 | 2.9801 | *KIF3A* |
| 19 | 11250001 | 11300000 | 2.9699 | *BRIP1* |
| 19 | 11200001 | 11250000 | 2.9604 | *INTS2* |
| 19 | 11200001 | 11250000 | 2.9604 | *BRIP1* |
| 21 | 49000001 | 49050000 | 2.9582 | *SEC23A* |
| 2 | 37150001 | 37200000 | 2.9572 | *TANC1* |
| 21 | 6100001 | 6150000 | 2.9541 | *CERS3* |
| 19 | 9450001 | 9500000 | 2.9534 | *MTMR4* |
| 19 | 9450001 | 9500000 | 2.9534 | *SEPTIN4* |
| 16 | 43650001 | 43700000 | 2.9469 | *CTNNBIP1* |
| 8 | 90050001 | 90100000 | 2.9413 | *CDK20* |
| 13 | 64250001 | 64300000 | 2.9376 | *MYH7B* |
| 13 | 64250001 | 64300000 | 2.9376 | *GSS* |
| 2 | 37450001 | 37500000 | 2.9361 | *DAPL1* |
| 21 | 1200001 | 1250000 | 2.9347 | *MAGEL2* |
| 20 | 14800001 | 14850000 | 2.9315 | *RGS7BP* |
| 3 | 109050001 | 109100000 | 2.9298 | *GRIK3* |
| 8 | 78000001 | 78050000 | 2.9269 | *NTRK2* |
| 18 | 52750001 | 52800000 | 2.9255 | *PPP1R37* |
| 18 | 52750001 | 52800000 | 2.9255 | *GEMIN7* |
| 18 | 52750001 | 52800000 | 2.9255 | *ZNF296* |
| 16 | 43600001 | 43650000 | 2.9199 | *CTNNBIP1* |
| 16 | 43600001 | 43650000 | 2.9199 | *LZIC* |
| 16 | 43600001 | 43650000 | 2.9199 | *NMNAT1* |
| 19 | 10500001 | 10550000 | 2.9183 | *DHX40* |
| 21 | 48350001 | 48400000 | 2.9175 | *CLEC14A* |
| 21 | 48350001 | 48400000 | 2.9175 | *SSTR1* |
| 11 | 100600001 | 100650000 | 2.913 | *HMCN2* |
| 6 | 87900001 | 87950000 | 2.9056 | *SNORD42* |
| 13 | 64600001 | 64650000 | 2.9048 | *UQCC1* |
| 2 | 43700001 | 43750000 | 2.8972 | *FMNL2* |
| 26 | 26050001 | 26100000 | 2.8962 | *SORCS3* |
| 4 | 31600001 | 31650000 | 2.895 | *FAM126A* |
| 27 | 37150001 | 37200000 | 2.8936 | *IKBKB* |
| 27 | 37150001 | 37200000 | 2.8936 | *POLB* |
| 29 | 48450001 | 48500000 | 2.8925 | *OSBPL5* |
| 27 | 33250001 | 33300000 | 2.8911 | *EIF4EBP1* |
| 27 | 33250001 | 33300000 | 2.8911 | *ASH2L* |
| 5 | 69600001 | 69650000 | 2.8911 | *CKAP4* |
| 5 | 82100001 | 82150000 | 2.8865 | *MRPS35* |
| 5 | 82100001 | 82150000 | 2.8865 | *PPFIBP1* |
| 5 | 82100001 | 82150000 | 2.8865 | *REP15* |
| 28 | 25350001 | 25400000 | 2.8763 | *U6* |
| 5 | 69650001 | 69700000 | 2.8757 | *TCP11L2* |
| 24 | 100001 | 150000 | 2.8678 | *OR9M1* |
| 14 | 23050001 | 23100000 | 2.8663 | *TGS1* |

| | | | | |
|---|---|---|---|---|
| 14 | 23050001 | 23100000 | 2.8663 | *TMEM68* |
| 7 | 43500001 | 43550000 | 2.8545 | *GRIN3B* |
| 7 | 43500001 | 43550000 | 2.8545 | *CNN2* |
| 7 | 43500001 | 43550000 | 2.8545 | *WDR18* |
| 7 | 43500001 | 43550000 | 2.8545 | *TMEM259* |
| 7 | 43500001 | 43550000 | 2.8545 | *ABCA7* |
| 7 | 38250001 | 38300000 | 2.8527 | *UNC5A* |
| 5 | 58550001 | 58600000 | 2.8437 | *OR6C75* |
| 5 | 58550001 | 58600000 | 2.8437 | *OR6C1Q* |
| 14 | 43350001 | 43400000 | 2.8382 | *TPD52* |
| 4 | 31700001 | 31750000 | 2.8373 | *FAM126A* |
| 1 | 42250001 | 42300000 | 2.8368 | *GABRR3* |
| 1 | 2750001 | 2800000 | 2.8202 | *C1H21orf62* |
| 1 | 2750001 | 2800000 | 2.8202 | *PAXBP1* |
| 28 | 650001 | 700000 | 2.8199 | *RHOU* |
| 2 | 22800001 | 22850000 | 2.8193 | *SP3* |
| 26 | 25950001 | 26000000 | 2.8164 | *SORCS3* |
| 29 | 42300001 | 42350000 | 2.814 | *MACROD1* |
| 29 | 42300001 | 42350000 | 2.814 | *OTUB1* |
| 29 | 42300001 | 42350000 | 2.814 | *COX8A* |
| 14 | 22800001 | 22850000 | 2.8115 | *XKR4* |
| 15 | 63100001 | 63150000 | 2.8113 | *WT1* |
| 21 | 1250001 | 1300000 | 2.8083 | *NDN* |
| 8 | 78050001 | 78100000 | 2.8081 | *NTRK2* |
| 19 | 51950001 | 52000000 | 2.808 | *RPTOR* |
| 28 | 25450001 | 25500000 | 2.8027 | *SRGN* |
| 8 | 75450001 | 75500000 | 2.8002 | *DCAF12* |
| 8 | 75450001 | 75500000 | 2.8002 | *UBAP2* |
| 13 | 62900001 | 62950000 | 2.7978 | *SNTA1* |
| 22 | 34100001 | 34150000 | 2.797 | *SUCLG2* |
| 6 | 91300001 | 91350000 | 2.7963 | *SHROOM3* |
| 14 | 23200001 | 23250000 | 2.7948 | *LYN* |
| 3 | 108800001 | 108850000 | 2.7944 | *GRIK3* |
| 15 | 23850001 | 23900000 | 2.7943 | *TTC12* |
| 27 | 37100001 | 37150000 | 2.79 | *IKBKB* |
| 14 | 15000001 | 15050000 | 2.7875 | *NSMCE2* |
| 23 | 10800001 | 10850000 | 2.7851 | *C23H6orf89* |
| 2 | 37350001 | 37400000 | 2.7699 | *TANC1* |
| 3 | 109000001 | 109050000 | 2.765 | *GRIK3* |
| 1 | 83700001 | 83750000 | 2.7643 | *MCF2L2* |
| 6 | 64600001 | 64650000 | 2.763 | *GABRG1* |
| 8 | 90150001 | 90200000 | 2.7615 | *MSANTD3* |
| 19 | 10650001 | 10700000 | 2.7552 | *VMP1* |
| 27 | 16350001 | 16400000 | 2.7552 | *MTNR1A* |
| 19 | 10650001 | 10700000 | 2.7552 | *CLTC* |
| 19 | 10650001 | 10700000 | 2.7552 | *PTRH2* |
| 23 | 10700001 | 10750000 | 2.7514 | *CPNE5* |
| 7 | 21650001 | 21700000 | 2.7503 | *KIF3A* |
| 7 | 21650001 | 21700000 | 2.7503 | *IL4* |
| 6 | 91250001 | 91300000 | 2.7456 | *CCDC158* |
| 6 | 91250001 | 91300000 | 2.7456 | *U6* |
| 23 | 9050001 | 9100000 | 2.745 | *TCP11* |

| | | | | |
|---|---|---|---|---|
| 23 | 9050001 | 9100000 | 2.745 | *ANKS1A* |
| 2 | 37500001 | 37550000 | 2.7397 | *DAPL1* |
| 9 | 68300001 | 68350000 | 2.736 | *SAMD3* |
| 16 | 4000001 | 4050000 | 2.7358 | *SRGAP2* |
| 2 | 14700001 | 14750000 | 2.7357 | *ITPRID2* |
| 12 | 15400001 | 15450000 | 2.7332 | *GTF2F2* |
| 6 | 35800001 | 35850000 | 2.7324 | *FAM13A* |
| 26 | 34500001 | 34550000 | 2.7308 | *ADRB1* |
| 3 | 117500001 | 117550000 | 2.7197 | *ILKAP* |
| 10 | 32300001 | 32350000 | 2.7153 | *CDIN1* |
| 1 | 149250001 | 149300000 | 2.7118 | *RIPPLY3* |
| 1 | 149250001 | 149300000 | 2.7118 | *U6* |
| 28 | 15450001 | 15500000 | 2.7115 | *CCDC6* |
| 13 | 62800001 | 62850000 | 2.7099 | *CDK5RAP1* |
| 13 | 62800001 | 62850000 | 2.7099 | *BPIFB5* |
| 7 | 90500001 | 90550000 | 2.7094 | *ADGRV1* |
| 5 | 53800001 | 53850000 | 2.7091 | *SLC16A7* |
| 16 | 4050001 | 4100000 | 2.7062 | *SRGAP2* |
| 1 | 149600001 | 149650000 | 2.7017 | *DYRK1A* |
| 13 | 62850001 | 62900000 | 2.695 | *CDK5RAP1* |
| 13 | 62850001 | 62900000 | 2.695 | *SNTA1* |
| 24 | 300001 | 350000 | 2.6932 | *PARD6G* |
| 23 | 33500001 | 33550000 | 2.6922 | *NRSN1* |
| 9 | 81800001 | 81850000 | 2.6819 | *UTRN* |
| 7 | 90450001 | 90500000 | 2.6786 | *ADGRV1* |
| 15 | 63150001 | 63200000 | 2.6781 | *WT1* |
| 22 | 33400001 | 33450000 | 2.6773 | *TAFA1* |
| 11 | 104350001 | 104400000 | 2.6736 | *CACFD1* |
| 11 | 104350001 | 104400000 | 2.6736 | *SLC2A6* |
| 6 | 65850001 | 65900000 | 2.6713 | *GABRB1* |
| 18 | 13000001 | 13050000 | 2.6705 | *ZCCHC14* |
| 23 | 13200001 | 13250000 | 2.669 | *KIF6* |
| 16 | 43700001 | 43750000 | 2.6688 | *CLSTN1* |
| 8 | 83850001 | 83900000 | 2.6647 | *IARS1* |
| 23 | 10750001 | 10800000 | 2.6601 | *PPIL1* |
| 23 | 10750001 | 10800000 | 2.6601 | *CPNE5* |
| 23 | 10750001 | 10800000 | 2.6601 | *C23H6orf89* |
| 6 | 2400001 | 2450000 | 2.6589 | *NPY5R* |
| 6 | 2400001 | 2450000 | 2.6589 | *NPY1R* |
| 7 | 44100001 | 44150000 | 2.6584 | *ATP8B3* |
| 7 | 44100001 | 44150000 | 2.6584 | *ONECUT3* |
| 6 | 88800001 | 88850000 | 2.6574 | *CXCL8* |
| 18 | 12900001 | 12950000 | 2.6564 | *FBXO31* |
| 8 | 90750001 | 90800000 | 2.6529 | *PLPPR1* |
| 18 | 12950001 | 13000000 | 2.651 | *FBXO31* |
| 18 | 12950001 | 13000000 | 2.651 | *MAP1LC3B* |
| 18 | 12950001 | 13000000 | 2.651 | *ZCCHC14* |
| 15 | 63300001 | 63350000 | 2.6508 | *EIF3M* |
| 18 | 52800001 | 52850000 | 2.6504 | *NKPD1* |
| 18 | 52800001 | 52850000 | 2.6504 | *TRAPPC6A* |
| 18 | 52800001 | 52850000 | 2.6504 | *PPP1R37* |
| 18 | 52800001 | 52850000 | 2.6504 | *BLOC1S3* |

| | | | | |
|---|---|---|---|---|
| 5 | 58600001 | 58650000 | 2.6501 | *OR6C7H* |
| 15 | 40650001 | 40700000 | 2.6427 | *DKK3* |
| 15 | 40350001 | 40400000 | 2.6416 | *MICAL2* |
| 27 | 33200001 | 33250000 | 2.6411 | *EIF4EBP1* |
| 27 | 33200001 | 33250000 | 2.6411 | *ADRB3* |
| 10 | 76200001 | 76250000 | 2.6347 | *SYNE2* |
| 14 | 24600001 | 24650000 | 2.6311 | *UBXN2B* |
| 5 | 66250001 | 66300000 | 2.6307 | *IGF1* |
| 14 | 23100001 | 23150000 | 2.6305 | *LYN* |
| 27 | 37050001 | 37100000 | 2.629 | *PLAT* |
| 7 | 21050001 | 21100000 | 2.6231 | *GNG7* |
| 26 | 19350001 | 19400000 | 2.6171 | *LOXL4* |
| 6 | 88550001 | 88600000 | 2.6116 | *AFM* |
| 5 | 105250001 | 105300000 | 2.607 | *KCNA1* |
| 29 | 42500001 | 42550000 | 2.6055 | *FERMT3* |
| 29 | 42500001 | 42550000 | 2.6055 | *STIP1* |
| 29 | 42500001 | 42550000 | 2.6055 | *DNAJC4* |
| 29 | 42500001 | 42550000 | 2.6055 | *NUDT22* |
| 29 | 42500001 | 42550000 | 2.6055 | *VEGFB* |
| 29 | 42500001 | 42550000 | 2.6055 | *FKBP2* |
| 29 | 42500001 | 42550000 | 2.6055 | *TRPT1* |
| 29 | 42500001 | 42550000 | 2.6055 | *PPP1R14B* |
| 8 | 71500001 | 71550000 | 2.6052 | *ADAM28* |
| 17 | 40400001 | 40450000 | 2.6034 | *RXFP1* |
| 26 | 9550001 | 9600000 | 2.6031 | *PTEN* |
| 26 | 9550001 | 9600000 | 2.6031 | *U6* |
| 2 | 36050001 | 36100000 | 2.6028 | *RBMS1* |
| 2 | 36050001 | 36100000 | 2.6028 | *ITGB6* |
| 13 | 63350001 | 63400000 | 2.6003 | *RALY* |
| 14 | 23150001 | 23200000 | 2.5958 | *LYN* |
| 7 | 43550001 | 43600000 | 2.5933 | *ARHGAP45* |
| 7 | 43550001 | 43600000 | 2.5933 | *ABCA7* |
| 7 | 43550001 | 43600000 | 2.5933 | *SBNO2* |
| 7 | 43550001 | 43600000 | 2.5933 | *POLR2E* |
| 7 | 43550001 | 43600000 | 2.5933 | *GPX4* |
| 4 | 13200001 | 13250000 | 2.5926 | *DYNC1I1* |
| 22 | 33350001 | 33400000 | 2.5917 | *TAFA1* |
| 2 | 18800001 | 18850000 | 2.5911 | *PDE11A* |
| 2 | 18800001 | 18850000 | 2.5911 | *CYCT* |
| 14 | 22700001 | 22750000 | 2.5878 | *XKR4* |
| 19 | 9500001 | 9550000 | 2.5856 | *TEX14* |
| 10 | 30400001 | 30450000 | 2.5853 | *AQR* |
| 24 | 650001 | 700000 | 2.5847 | *CTDP1* |
| 13 | 64800001 | 64850000 | 2.5803 | *FER1L4* |
| 13 | 64800001 | 64850000 | 2.5803 | *SPAG4* |
| 13 | 64800001 | 64850000 | 2.5803 | *CPNE1* |
| 16 | 42000001 | 42050000 | 2.5777 | *FBXO6* |
| 16 | 42000001 | 42050000 | 2.5777 | *FBXO2* |
| 16 | 42000001 | 42050000 | 2.5777 | *FBXO44* |
| 16 | 42000001 | 42050000 | 2.5777 | *MAD2L2* |
| 26 | 25000001 | 25050000 | 2.5771 | *CFAP58* |
| 28 | 25250001 | 25300000 | 2.5756 | *KIFBP* |

| | | | | |
|---|---|---|---|---|
| 4 | 10450001 | 10500000 | 2.575 | *SAMD9* |
| 5 | 82000001 | 82050000 | 2.5747 | *KLHL42* |
| 14 | 23300001 | 23350000 | 2.5741 | *PLAG1* |
| 14 | 23300001 | 23350000 | 2.5741 | *MOS* |
| 5 | 53750001 | 53800000 | 2.5731 | *SLC16A7* |
| 8 | 83950001 | 84000000 | 2.5701 | *CENPP* |
| 8 | 83950001 | 84000000 | 2.5701 | *NOL8* |
| 8 | 83950001 | 84000000 | 2.5701 | *U6* |
| 14 | 24350001 | 24400000 | 2.5695 | *FAM110B* |
| 14 | 44050001 | 44100000 | 2.5637 | *ZNF704* |
| 7 | 90600001 | 90650000 | 2.5605 | *ADGRV1* |
| 5 | 51750001 | 51800000 | 2.56 | *TAFA2* |
| 7 | 90550001 | 90600000 | 2.5574 | *ADGRV1* |
| 9 | 76050001 | 76100000 | 2.5574 | *ARFGEF3* |
| 17 | 71750001 | 71800000 | 2.5557 | *BCR* |
| 2 | 37100001 | 37150000 | 2.5548 | *WDSUB1* |
| 2 | 37100001 | 37150000 | 2.5548 | *TANC1* |
| 2 | 42050001 | 42100000 | 2.5525 | *GALNT13* |
| 6 | 94950001 | 95000000 | 2.5521 | *FGF5* |
| 5 | 105100001 | 105150000 | 2.5473 | *KCNA5* |
| 18 | 52900001 | 52950000 | 2.5447 | *MARK4* |
| 18 | 52900001 | 52950000 | 2.5447 | *EXOC3L2* |
| 18 | 52900001 | 52950000 | 2.5447 | *U6* |
| 25 | 3200001 | 3250000 | 2.5416 | *ADCY9* |
| 20 | 65800001 | 65850000 | 2.538 | *ADCY2* |
| 5 | 105300001 | 105350000 | 2.5379 | *KCNA6* |
| 8 | 77950001 | 78000000 | 2.537 | *NTRK2* |
| 15 | 25800001 | 25850000 | 2.5368 | *CADM1* |
| 21 | 65950001 | 66000000 | 2.5364 | *MIR409* |
| 21 | 65950001 | 66000000 | 2.5364 | *MIR369* |
| 16 | 71600001 | 71650000 | 2.5362 | *LPGAT1* |
| 11 | 81050001 | 81100000 | 2.5352 | *VSNL1* |
| 11 | 81050001 | 81100000 | 2.5352 | *RAD51AP2* |
| 5 | 41500001 | 41550000 | 2.5339 | *ABCD2* |
| 25 | 3500001 | 3550000 | 2.5332 | *CORO7* |
| 25 | 3500001 | 3550000 | 2.5332 | *VASN* |
| 25 | 3500001 | 3550000 | 2.5332 | *GLIS2* |
| 25 | 3500001 | 3550000 | 2.5332 | *PAM16* |
| 12 | 15250001 | 15300000 | 2.5318 | *GTF2F2* |
| 20 | 550001 | 600000 | 2.5301 | *SLIT3* |
| 20 | 550001 | 600000 | 2.5301 | *MIR218-2* |
| 9 | 76100001 | 76150000 | 2.5298 | *ARFGEF3* |
| 2 | 42100001 | 42150000 | 2.5285 | *GALNT13* |
| 11 | 80900001 | 80950000 | 2.5265 | *SMC6* |
| 11 | 80900001 | 80950000 | 2.5265 | *VSNL1* |
| 7 | 38300001 | 38350000 | 2.5231 | *UNC5A* |
| 7 | 38300001 | 38350000 | 2.5231 | *HK3* |
| 16 | 45450001 | 45500000 | 2.5229 | *TNFRSF9* |
| 2 | 37600001 | 37650000 | 2.5223 | *PKP4* |
| 6 | 19950001 | 20000000 | 2.5191 | *TET2* |
| 8 | 79300001 | 79350000 | 2.519 | *GOLM1* |
| 17 | 14400001 | 14450000 | 2.518 | *GAB1* |

| | | | | |
|---|---|---|---|---|
| 1 | 50000001 | 50050000 | 2.5176 | *ALCAM* |
| 8 | 75550001 | 75600000 | 2.5106 | *UBAP1* |
| 10 | 80950001 | 81000000 | 2.5105 | *EXD2* |
| 13 | 67800001 | 67850000 | 2.507 | *DHX35* |
| 10 | 72800001 | 72850000 | 2.5069 | *SIX1* |
| 29 | 42350001 | 42400000 | 2.5057 | *MACROD1* |
| 10 | 57900001 | 57950000 | 2.4996 | *MYO5A* |
| 9 | 76150001 | 76200000 | 2.4989 | *HEBP2* |
| 5 | 105350001 | 105400000 | 2.4984 | *KCNA6* |
| 15 | 23900001 | 23950000 | 2.4979 | *TTC12* |
| 9 | 11050001 | 11100000 | 2.4962 | *RIMS1* |
| 1 | 29100001 | 29150000 | 2.4933 | *GBE1* |
| 22 | 33450001 | 33500000 | 2.4932 | *TAFA1* |
| 3 | 23550001 | 23600000 | 2.4913 | *PHGDH* |
| 3 | 23550001 | 23600000 | 2.4913 | *HMGCS2* |
| 8 | 75500001 | 75550000 | 2.4886 | *DCAF12* |
| 5 | 82050001 | 82100000 | 2.4876 | *MRPS35* |
| 5 | 82050001 | 82100000 | 2.4876 | *KLHL42* |
| 5 | 82050001 | 82100000 | 2.4876 | *MANSC4* |
| 15 | 64450001 | 64500000 | 2.4819 | *LMO2* |
| 3 | 117700001 | 117750000 | 2.4809 | *ASB1* |
| 8 | 90300001 | 90350000 | 2.4787 | *TMEFF1* |
| 8 | 90300001 | 90350000 | 2.4787 | *CAVIN4* |
| 4 | 31500001 | 31550000 | 2.4781 | *TOMM7* |
| 20 | 20500001 | 20550000 | 2.4755 | *RAB3C* |
| 25 | 17950001 | 18000000 | 2.4753 | *GP2* |
| 11 | 4750001 | 4800000 | 2.4747 | *AFF3* |
| 5 | 58500001 | 58550000 | 2.4734 | *OR6C76* |
| 7 | 43400001 | 43450000 | 2.4713 | *MED16* |
| 7 | 43400001 | 43450000 | 2.4713 | *R3HDM4* |
| 7 | 43400001 | 43450000 | 2.4713 | *PRTN3* |
| 7 | 43400001 | 43450000 | 2.4713 | *ELANE* |
| 7 | 43400001 | 43450000 | 2.4713 | *CFD* |
| 7 | 43400001 | 43450000 | 2.4713 | *U6* |
| 2 | 22750001 | 22800000 | 2.4698 | *SP3* |
| 21 | 6150001 | 6200000 | 2.4683 | *CERS3* |
| 2 | 36150001 | 36200000 | 2.4645 | *ITGB6* |
| 10 | 30800001 | 30850000 | 2.4642 | *DPH6* |
| 10 | 30800001 | 30850000 | 2.4642 | *U6* |
| 20 | 15150001 | 15200000 | 2.4623 | *RNF180* |
| 17 | 20250001 | 20300000 | 2.4594 | *PCDH18* |
| 19 | 11150001 | 11200000 | 2.4593 | *INTS2* |
| 19 | 11150001 | 11200000 | 2.4593 | *MED13* |
| 1 | 83650001 | 83700000 | 2.4536 | *MCF2L2* |
| 2 | 18900001 | 18950000 | 2.4512 | *PDE11A* |
| 12 | 12000001 | 12050000 | 2.4479 | *VWA8* |
| 20 | 18000001 | 18050000 | 2.4464 | *ZSWIM6* |
| 28 | 25550001 | 25600000 | 2.446 | *SUPV3L1* |
| 28 | 25550001 | 25600000 | 2.446 | *HKDC1* |
| 28 | 25550001 | 25600000 | 2.446 | *VPS26A* |
| 28 | 25300001 | 25350000 | 2.4442 | *KIFBP* |
| 1 | 126850001 | 126900000 | 2.4441 | *TFDP2* |

| | | | | |
|---|---|---|---|---|
| 13 | 39700001 | 39750000 | 2.4425 | *CFAP61* |
| 8 | 89550001 | 89600000 | 2.4409 | *NXNL2* |
| 15 | 64400001 | 64450000 | 2.4365 | *FBXO3* |
| 20 | 20300001 | 20350000 | 2.4356 | *PDE4D* |
| 3 | 46300001 | 46350000 | 2.4331 | *DPYD* |
| 8 | 92550001 | 92600000 | 2.4317 | *CYLC2* |
| 8 | 92550001 | 92600000 | 2.4317 | *U6* |
| 8 | 93850001 | 93900000 | 2.4297 | *TOPORSL* |
| 9 | 10000001 | 10050000 | 2.4289 | *B3GAT2* |
| 15 | 43200001 | 43250000 | 2.4262 | *IPO7* |
| 15 | 43200001 | 43250000 | 2.4262 | *SNORA23* |
| 24 | 56850001 | 56900000 | 2.4156 | *ATP8B1* |
| 24 | 56850001 | 56900000 | 2.4156 | *U6* |
| 19 | 11050001 | 11100000 | 2.4151 | *MED13* |
| 21 | 49150001 | 49200000 | 2.4147 | *PNN* |
| 21 | 49150001 | 49200000 | 2.4147 | *TRAPPC6B* |
| 5 | 41550001 | 41600000 | 2.4144 | *ABCD2* |
| 15 | 41100001 | 41150000 | 2.4143 | *GALNT18* |
| 11 | 56700001 | 56750000 | 2.4141 | *REG3A* |
| 8 | 86500001 | 86550000 | 2.4139 | *AUH* |
| 21 | 49050001 | 49100000 | 2.4132 | *SEC23A* |
| 9 | 42700001 | 42750000 | 2.412 | *PDSS2* |
| 6 | 88150001 | 88200000 | 2.4105 | *ANKRD17* |
| 6 | 88150001 | 88200000 | 2.4105 | *COX18* |
| 5 | 58850001 | 58900000 | 2.41 | *OR6C1N* |
| 5 | 58850001 | 58900000 | 2.41 | *OR6C5* |
| 2 | 43650001 | 43700000 | 2.4074 | *FMNL2* |
| 16 | 42550001 | 42600000 | 2.4066 | *MASP2* |
| 16 | 42550001 | 42600000 | 2.4066 | *TARDBP* |
| 16 | 42550001 | 42600000 | 2.4066 | *SRM* |
| 14 | 23250001 | 23300000 | 2.4048 | *RPS20* |
| 14 | 23250001 | 23300000 | 2.4048 | *MOS* |
| 14 | 23250001 | 23300000 | 2.4048 | *U1* |
| 8 | 90950001 | 91000000 | 2.4035 | *PLPPR1* |
| 25 | 18000001 | 18050000 | 2.4033 | *UMOD* |
| 25 | 18000001 | 18050000 | 2.4033 | *PDILT* |
| 25 | 18000001 | 18050000 | 2.4033 | *GP2* |
| 5 | 82150001 | 82200000 | 2.4028 | *PPFIBP1* |
| 9 | 86150001 | 86200000 | 2.3999 | *UST* |
| 9 | 91700001 | 91750000 | 2.3998 | *TIAM2* |
| 15 | 77150001 | 77200000 | 2.3993 | *MADD* |
| 15 | 77150001 | 77200000 | 2.3993 | *MYBPC3* |
| 19 | 52000001 | 52050000 | 2.3982 | *ENDOV* |
| 19 | 52000001 | 52050000 | 2.3982 | *RPTOR* |
| 10 | 27650001 | 27700000 | 2.3974 | *OR4G18* |
| 10 | 27650001 | 27700000 | 2.3974 | *OR4K36* |
| 6 | 64550001 | 64600000 | 2.3971 | *GABRG1* |
| 9 | 42750001 | 42800000 | 2.3969 | *PDSS2* |
| 2 | 90150001 | 90200000 | 2.3966 | *TMEM237* |
| 2 | 90150001 | 90200000 | 2.3966 | *C2CD6* |
| 2 | 90150001 | 90200000 | 2.3966 | *MPP4* |
| 2 | 90150001 | 90200000 | 2.3966 | *U6* |

| | | | | |
|---|---|---|---|---|
| 6 | 88650001 | 88700000 | 2.3962 | *RASSF6* |
| 10 | 27600001 | 27650000 | 2.3945 | *OR4G10* |
| 10 | 27600001 | 27650000 | 2.3945 | *OR4F67B* |
| 7 | 38550001 | 38600000 | 2.3894 | *FGFR4* |
| 7 | 38550001 | 38600000 | 2.3894 | *ZNF346* |
| 19 | 31050001 | 31100000 | 2.3889 | *MYOCD* |
| 8 | 83900001 | 83950000 | 2.3864 | *NOL8* |
| 8 | 83900001 | 83950000 | 2.3864 | *IARS1* |
| 8 | 83900001 | 83950000 | 2.3864 | *SNORA84* |
| 6 | 4700001 | 4750000 | 2.3853 | *PRDM5* |
| 20 | 56700001 | 56750000 | 2.3841 | *ZNF622* |
| 14 | 22900001 | 22950000 | 2.383 | *XKR4* |
| 19 | 39700001 | 39750000 | 2.3824 | *STAC2* |
| 19 | 39700001 | 39750000 | 2.3824 | *FBXL20* |
| 7 | 44200001 | 44250000 | 2.3818 | *SCAMP4* |
| 7 | 44200001 | 44250000 | 2.3818 | *CSNK1G2* |
| 6 | 68600001 | 68650000 | 2.3809 | *SCFD2* |
| 21 | 6300001 | 6350000 | 2.3797 | *ADAMTS17* |
| 16 | 43750001 | 43800000 | 2.3675 | *CLSTN1* |
| 16 | 43750001 | 43800000 | 2.3675 | *PIK3CD* |
| 11 | 45800001 | 45850000 | 2.3662 | *ECRG4* |
| 2 | 30750001 | 30800000 | 2.3653 | *CSRNP3* |
| 14 | 23350001 | 23400000 | 2.3612 | *PLAG1* |
| 14 | 23350001 | 23400000 | 2.3612 | *CHCHD7* |
| 21 | 49100001 | 49150000 | 2.3601 | *GEMIN2* |
| 21 | 49100001 | 49150000 | 2.3601 | *TRAPPC6B* |
| 21 | 49100001 | 49150000 | 2.3601 | *SEC23A* |
| 29 | 42450001 | 42500000 | 2.359 | *MACROD1* |
| 29 | 42450001 | 42500000 | 2.359 | *STIP1* |
| 15 | 39950001 | 40000000 | 2.3586 | *TEAD1* |
| 21 | 6200001 | 6250000 | 2.3569 | *CERS3* |
| 17 | 65200001 | 65250000 | 2.3549 | *KIAA1671* |
| 17 | 61000001 | 61050000 | 2.3549 | *SDSL* |
| 17 | 61000001 | 61050000 | 2.3549 | *SDS* |
| 17 | 61000001 | 61050000 | 2.3549 | *PLBD2* |
| 15 | 40500001 | 40550000 | 2.3532 | *MICAL2* |
| 9 | 42650001 | 42700000 | 2.3529 | *PDSS2* |
| 15 | 1850001 | 1900000 | 2.3524 | *GRIA4* |
| 27 | 37000001 | 37050000 | 2.3501 | *AP3M2* |
| 18 | 52850001 | 52900000 | 2.3489 | *EXOC3L2* |
| 7 | 7650001 | 7700000 | 2.3477 | *RASAL3* |
| 7 | 7650001 | 7700000 | 2.3477 | *PGLYRP2* |
| 4 | 51900001 | 51950000 | 2.3466 | *CAV2* |
| 4 | 51900001 | 51950000 | 2.3466 | *CAV1* |
| 15 | 40000001 | 40050000 | 2.3462 | *TEAD1* |
| 4 | 71600001 | 71650000 | 2.3412 | *NPY* |
| 5 | 71450001 | 71500000 | 2.3411 | *TIMP3* |
| 7 | 21700001 | 21750000 | 2.3396 | *IL4* |
| 7 | 21700001 | 21750000 | 2.3396 | *IL13* |
| 8 | 75600001 | 75650000 | 2.339 | *UBAP1* |
| 16 | 43800001 | 43850000 | 2.3363 | *PIK3CD* |
| 16 | 43800001 | 43850000 | 2.3363 | *U6* |

| | | | | |
|---|---|---|---|---|
| 3 | 98450001 | 98500000 | 2.3351 | *TRABD2B* |
| 5 | 69550001 | 69600000 | 2.3315 | *NUAK1* |
| 1 | 46050001 | 46100000 | 2.3301 | *CEP97* |
| 1 | 46050001 | 46100000 | 2.3301 | *ZBTB11* |
| 1 | 46050001 | 46100000 | 2.3301 | *RPL24* |
| 23 | 11500001 | 11550000 | 2.3301 | *MDGA1* |
| 1 | 56050001 | 56100000 | 2.3298 | *NECTIN3* |
| 15 | 62850001 | 62900000 | 2.3297 | *RCN1* |
| 19 | 51900001 | 51950000 | 2.3296 | *RPTOR* |
| 9 | 11100001 | 11150000 | 2.328 | *RIMS1* |
| 6 | 62900001 | 62950000 | 2.3275 | *KCTD8* |
| 2 | 37650001 | 37700000 | 2.3254 | *PKP4* |
| 10 | 76550001 | 76600000 | 2.3243 | *MTHFD1* |
| 5 | 43650001 | 43700000 | 2.3241 | *RAB3IP* |
| 11 | 102450001 | 102500000 | 2.3227 | *TTF1* |
| 11 | 102450001 | 102500000 | 2.3227 | *CFAP77* |
| 1 | 149650001 | 149700000 | 2.3222 | *DYRK1A* |
| 12 | 15350001 | 15400000 | 2.3201 | *GTF2F2* |
| 25 | 20700001 | 20750000 | 2.3192 | *USP31* |
| 22 | 34050001 | 34100000 | 2.3187 | *SUCLG2* |
| 13 | 64650001 | 64700000 | 2.3174 | *UQCC1* |
| 13 | 64650001 | 64700000 | 2.3174 | *GDF5* |
| 13 | 64650001 | 64700000 | 2.3174 | *CEP250* |
| 5 | 47850001 | 47900000 | 2.3172 | *HMGA2* |
| 21 | 1150001 | 1200000 | 2.3166 | *MKRN3* |
| 9 | 42600001 | 42650000 | 2.3158 | *PDSS2* |
| 11 | 104300001 | 104350000 | 2.3099 | *ADAMTS13* |
| 11 | 104300001 | 104350000 | 2.3099 | *REXO4* |
| 11 | 104300001 | 104350000 | 2.3099 | *STKLD1* |
| 7 | 12600001 | 12650000 | 2.3091 | *FARSA* |
| 7 | 12600001 | 12650000 | 2.3091 | *SYCE2* |
| 7 | 12600001 | 12650000 | 2.3091 | *RAD23A* |
| 7 | 12600001 | 12650000 | 2.3091 | *CALR* |
| 7 | 12600001 | 12650000 | 2.3091 | *GADD45GIP1* |
| 10 | 37250001 | 37300000 | 2.3046 | *SPTBN5* |
| 10 | 37250001 | 37300000 | 2.3046 | *EHD4* |
| 7 | 12650001 | 12700000 | 2.3026 | *MAST1* |
| 7 | 12650001 | 12700000 | 2.3026 | *SYCE2* |
| 7 | 12650001 | 12700000 | 2.3026 | *GCDH* |
| 7 | 12650001 | 12700000 | 2.3026 | *KLF1* |
| 7 | 12650001 | 12700000 | 2.3026 | *DNASE2* |
| 17 | 40250001 | 40300000 | 2.3024 | *ETFDH* |
| 17 | 40250001 | 40300000 | 2.3024 | *PPID* |
| 2 | 71200001 | 71250000 | 2.3005 | *TMEM37* |
| 2 | 71200001 | 71250000 | 2.3005 | *SCTR* |
| 27 | 16400001 | 16450000 | 2.2987 | *FAT1* |
| 27 | 16400001 | 16450000 | 2.2987 | *MTNR1A* |
| 2 | 23700001 | 23750000 | 2.294 | *RAPGEF4* |
| 15 | 40800001 | 40850000 | 2.2931 | *USP47* |
| 10 | 76250001 | 76300000 | 2.2926 | *SYNE2* |
| 2 | 36450001 | 36500000 | 2.288 | *LY75* |
| 1 | 18550001 | 18600000 | 2.288 | *TMPRSS15* |

| | | | | |
|---|---|---|---|---|
| 29 | 24800001 | 24850000 | 2.2859 | *NAV2* |
| 7 | 23050001 | 23100000 | 2.2848 | *CDC42SE2* |
| 7 | 23050001 | 23100000 | 2.2848 | *RAPGEF6* |
| 19 | 11300001 | 11350000 | 2.2823 | *BRIP1* |
| 2 | 62100001 | 62150000 | 2.2806 | *RAB3GAP1* |
| 2 | 62100001 | 62150000 | 2.2806 | *ZRANB3* |
| 2 | 62200001 | 62250000 | 2.2765 | *RAB3GAP1* |
| 25 | 3650001 | 3700000 | 2.2764 | *CDIP1* |
| 25 | 3650001 | 3700000 | 2.2764 | *HMOX2* |
| 9 | 42550001 | 42600000 | 2.2755 | *SOBP* |
| 28 | 33150001 | 33200000 | 2.2744 | *KCNMA1* |
| 2 | 122200001 | 122250000 | 2.2728 | *SERINC2* |
| 16 | 44200001 | 44250000 | 2.2725 | *H6PD* |
| 3 | 118450001 | 118500000 | 2.2684 | *HDAC4* |
| 13 | 63650001 | 63700000 | 2.2683 | *AHCY* |
| 13 | 63650001 | 63700000 | 2.2683 | *ASIP* |
| 14 | 43250001 | 43300000 | 2.2673 | *TPD52* |
| 8 | 90250001 | 90300000 | 2.2671 | *TMEFF1* |
| 21 | 48300001 | 48350000 | 2.2636 | *SSTR1* |
| 1 | 2800001 | 2850000 | 2.2627 | *SYNJ1* |
| 1 | 2800001 | 2850000 | 2.2627 | *PAXBP1* |
| 14 | 34750001 | 34800000 | 2.2607 | *EYA1* |
| 19 | 31100001 | 31150000 | 2.26 | *MYOCD* |
| 19 | 52400001 | 52450000 | 2.2576 | *CARD14* |
| 19 | 52400001 | 52450000 | 2.2576 | *SLC26A11* |
| 19 | 52400001 | 52450000 | 2.2576 | *SGSH* |
| 25 | 18050001 | 18100000 | 2.2572 | *PDILT* |
| 25 | 18050001 | 18100000 | 2.2572 | *ACSM5* |
| 20 | 14850001 | 14900000 | 2.2568 | *RGS7BP* |
| 4 | 14400001 | 14450000 | 2.2543 | *DLX5* |
| 11 | 20700001 | 20750000 | 2.254 | *ATL2* |
| 20 | 65750001 | 65800000 | 2.2469 | *ADCY2* |
| 15 | 39750001 | 39800000 | 2.2456 | *TEAD1* |
| 25 | 25550001 | 25600000 | 2.2456 | *GSG1L* |
| 1 | 45900001 | 45950000 | 2.2444 | *SENP7* |
| 19 | 9400001 | 9450000 | 2.2406 | *HSF5* |
| 19 | 9400001 | 9450000 | 2.2406 | *MTMR4* |
| 8 | 91200001 | 91250000 | 2.2398 | *ALDOB* |
| 8 | 91200001 | 91250000 | 2.2398 | *PGAP4* |
| 9 | 10050001 | 10100000 | 2.2386 | *B3GAT2* |
| 29 | 49200001 | 49250000 | 2.2365 | *CD81* |
| 17 | 60950001 | 61000000 | 2.2349 | *LHX5* |
| 28 | 25500001 | 25550000 | 2.2321 | *VPS26A* |
| 4 | 16200001 | 16250000 | 2.2318 | *ICA1* |
| 20 | 17950001 | 18000000 | 2.2315 | *ZSWIM6* |
| 10 | 70750001 | 70800000 | 2.2312 | *KIAA0586* |
| 15 | 40950001 | 41000000 | 2.2311 | *U6* |
| 29 | 49100001 | 49150000 | 2.2273 | *KCNQ1* |
| 21 | 1900001 | 1950000 | 2.2266 | *SNRPN* |
| 11 | 4800001 | 4850000 | 2.2249 | *AFF3* |
| 8 | 90850001 | 90900000 | 2.2231 | *PLPPR1* |
| 10 | 45700001 | 45750000 | 2.2229 | *CSNK1G1* |

| | | | | |
|---|---|---|---|---|
| 10 | 76500001 | 76550000 | 2.2205 | *MTHFD1* |
| 3 | 67100001 | 67150000 | 2.2203 | *AK5* |
| 27 | 38300001 | 38350000 | 2.2179 | *SH2D4A* |
| 4 | 10500001 | 10550000 | 2.2165 | *HEPACAM2* |
| 1 | 149550001 | 149600000 | 2.2097 | *DYRK1A* |
| 12 | 15450001 | 15500000 | 2.2091 | *TPT1* |
| 10 | 26350001 | 26400000 | 2.2087 | *RNASE1* |
| 10 | 26350001 | 26400000 | 2.2087 | *RNASE4* |
| 10 | 26350001 | 26400000 | 2.2087 | *RNASE6* |
| 2 | 5850001 | 5900000 | 2.2069 | *MFSD6* |
| 8 | 10900001 | 10950000 | 2.2062 | *SCARA5* |
| 8 | 10900001 | 10950000 | 2.2062 | *U6* |
| 3 | 79100001 | 79150000 | 2.2051 | *PDE4B* |
| 2 | 97000001 | 97050000 | 2.2047 | *U6* |
| 4 | 64700001 | 64750000 | 2.2042 | *PDE1C* |
| 3 | 46050001 | 46100000 | 2.2021 | *DPYD* |
| 19 | 31750001 | 31800000 | 2.2013 | *HS3ST3A1* |
| 1 | 83250001 | 83300000 | 2.2004 | *PARL* |
| 7 | 19250001 | 19300000 | 2.1984 | *ARRDC5* |
| 7 | 19250001 | 19300000 | 2.1984 | *PLIN3* |
| 7 | 19250001 | 19300000 | 2.1984 | *UHRF1* |
| 6 | 30750001 | 30800000 | 2.1977 | *GRID2* |
| 6 | 91200001 | 91250000 | 2.1974 | *CCDC158* |
| 6 | 91200001 | 91250000 | 2.1974 | *STBD1* |
| 22 | 33300001 | 33350000 | 2.1969 | *TAFA1* |
| 11 | 100550001 | 100600000 | 2.196 | *HMCN2* |
| 1 | 87250001 | 87300000 | 2.1952 | *PEX5L* |
| 10 | 45300001 | 45350000 | 2.1952 | *RBPMS2* |
| 20 | 3850001 | 3900000 | 2.195 | *STK10* |
| 28 | 25600001 | 25650000 | 2.1946 | *HKDC1* |
| 16 | 43500001 | 43550000 | 2.1942 | *RBP7* |
| 16 | 43500001 | 43550000 | 2.1942 | *UBE4B* |
| 10 | 11150001 | 11200000 | 2.1935 | *SERINC5* |
| 10 | 30850001 | 30900000 | 2.1935 | *DPH6* |
| 25 | 13800001 | 13850000 | 2.1935 | *MPV17L* |
| 2 | 38900001 | 38950000 | 2.1934 | *CYTIP* |
| 2 | 17700001 | 17750000 | 2.1926 | *SESTD1* |
| 4 | 51850001 | 51900000 | 2.1924 | *CAV1* |
| 13 | 63700001 | 63750000 | 2.192 | *AHCY* |
| 11 | 99000001 | 99050000 | 2.1906 | *ODF2* |
| 11 | 99000001 | 99050000 | 2.1906 | *CERCAM* |
| 11 | 99000001 | 99050000 | 2.1906 | *URM1* |
| 11 | 99000001 | 99050000 | 2.1906 | *SNORA70* |
| 14 | 24800001 | 24850000 | 2.1905 | *NSMAF* |
| 8 | 71550001 | 71600000 | 2.1891 | *ADAM28* |
| 8 | 71550001 | 71600000 | 2.1891 | *ADAMDEC1* |
| 17 | 33050001 | 33100000 | 2.1879 | *ANKRD50* |
| 1 | 2350001 | 2400000 | 2.1853 | *IFNAR2* |
| 8 | 79400001 | 79450000 | 2.185 | *ISCA1* |
| 28 | 700001 | 750000 | 2.1842 | *RHOU* |
| 4 | 69650001 | 69700000 | 2.1828 | *SNX10* |
| 18 | 11150001 | 11200000 | 2.1818 | *KIAA0513* |

| | | | | |
|---|---|---|---|---|
| 18 | 11150001 | 11200000 | 2.1818 | *CIBAR2* |
| 14 | 75650001 | 75700000 | 2.1816 | *CNBD1* |
| 26 | 44550001 | 44600000 | 2.1815 | *CTBP2* |
| 13 | 11950001 | 12000000 | 2.1797 | *CAMK1D* |
| 13 | 11950001 | 12000000 | 2.1797 | *RNU6ATAC39P* |
| 1 | 2850001 | 2900000 | 2.1784 | *SYNJ1* |
| 1 | 18650001 | 18700000 | 2.1754 | *TMPRSS15* |
| 10 | 45750001 | 45800000 | 2.1754 | *CSNK1G1* |
| 1 | 45750001 | 45800000 | 2.1748 | *IMPG2* |
| 7 | 43450001 | 43500000 | 2.1739 | *ARID3A* |
| 7 | 43450001 | 43500000 | 2.1739 | *KISS1R* |
| 13 | 11900001 | 11950000 | 2.1721 | *CAMK1D* |
| 25 | 25700001 | 25750000 | 2.1714 | *XPO6* |
| 5 | 47200001 | 47250000 | 2.1709 | *GRIP1* |
| 19 | 11100001 | 11150000 | 2.1705 | *MED13* |
| 12 | 12050001 | 12100000 | 2.1702 | *VWA8* |
| 27 | 40300001 | 40350000 | 2.1689 | *RARB* |
| 6 | 62850001 | 62900000 | 2.1684 | *KCTD8* |
| 2 | 14750001 | 14800000 | 2.1681 | *ITPRID2* |
| 26 | 33250001 | 33300000 | 2.1666 | *VTI1A* |
| 10 | 26400001 | 26450000 | 2.1646 | *RNASE4* |
| 10 | 26400001 | 26450000 | 2.1646 | *ANG2* |
| 15 | 1900001 | 1950000 | 2.1593 | *GRIA4* |
| 25 | 25100001 | 25150000 | 2.1581 | *KATNIP* |
| 29 | 24750001 | 24800000 | 2.1561 | *DBX1* |
| 19 | 12050001 | 12100000 | 2.1557 | *BCAS3* |
| 22 | 13300001 | 13350000 | 2.1556 | *ZNF621* |
| 22 | 13300001 | 13350000 | 2.1556 | *ZNF619* |
| 10 | 76600001 | 76650000 | 2.1554 | *ZBTB25* |
| 10 | 76600001 | 76650000 | 2.1554 | *MTHFD1* |
| 10 | 76600001 | 76650000 | 2.1554 | *AKAP5* |
| 10 | 76600001 | 76650000 | 2.1554 | *ZBTB1* |
| 14 | 32400001 | 32450000 | 2.1551 | *C14H8orf34* |
| 5 | 53650001 | 53700000 | 2.1539 | *SLC16A7* |
| 28 | 15400001 | 15450000 | 2.1538 | *MRLN* |
| 1 | 49900001 | 49950000 | 2.153 | *ALCAM* |
| 8 | 90900001 | 90950000 | 2.1528 | *PLPPR1* |
| 10 | 57950001 | 58000000 | 2.1528 | *MYO5A* |
| 14 | 44300001 | 44350000 | 2.1515 | *PAG1* |
| 1 | 45850001 | 45900000 | 2.1511 | *SENP7* |
| 8 | 78250001 | 78300000 | 2.1482 | *NTRK2* |
| 6 | 68550001 | 68600000 | 2.1481 | *SCFD2* |
| 26 | 51850001 | 51900000 | 2.147 | *STK32C* |
| 26 | 51850001 | 51900000 | 2.147 | *DPYSL4* |
| 8 | 91000001 | 91050000 | 2.143 | *PLPPR1* |
| 7 | 38350001 | 38400000 | 2.1421 | *UIMC1* |
| 7 | 38350001 | 38400000 | 2.1421 | *HK3* |
| 8 | 76600001 | 76650000 | 2.1417 | *FRMD3* |
| 15 | 4450001 | 4500000 | 2.1405 | *PDGFD* |
| 7 | 22950001 | 23000000 | 2.14 | *RAPGEF6* |
| 15 | 4500001 | 4550000 | 2.1397 | *PDGFD* |
| 11 | 45750001 | 45800000 | 2.1391 | *UXS1* |

| | | | | |
|---|---|---|---|---|
| 11 | 45750001 | 45800000 | 2.1391 | *U5* |
| 29 | 42400001 | 42450000 | 2.1359 | *MACROD1* |
| 29 | 42400001 | 42450000 | 2.1359 | *FLRT1* |
| 17 | 9700001 | 9750000 | 2.1357 | *NR3C2* |
| 4 | 14050001 | 14100000 | 2.1357 | *SEM1* |
| 6 | 75200001 | 75250000 | 2.1353 | *Y_RNA* |
| 1 | 83550001 | 83600000 | 2.135 | *KLHL6* |
| 5 | 2050001 | 2100000 | 2.1348 | *TRHDE* |
| 14 | 23800001 | 23850000 | 2.1346 | *U6* |
| 1 | 46000001 | 46050000 | 2.1342 | *ZBTB11* |
| 1 | 46000001 | 46050000 | 2.1342 | *PCNP* |
| 2 | 38550001 | 38600000 | 2.1334 | *ACVR1* |
| 25 | 3550001 | 3600000 | 2.1321 | *DNAJA3* |
| 25 | 3550001 | 3600000 | 2.1321 | *CORO7* |
| 3 | 46250001 | 46300000 | 2.1316 | *DPYD* |
| 19 | 33900001 | 33950000 | 2.13 | *SLC47A2* |
| 19 | 33900001 | 33950000 | 2.13 | *ALDH3A2* |
| 8 | 73950001 | 74000000 | 2.1281 | *ADRA1A* |
| 2 | 106200001 | 106250000 | 2.1271 | *CXCR1* |
| 3 | 119300001 | 119350000 | 2.1253 | *OR9S44P* |
| 3 | 119300001 | 119350000 | 2.1253 | *OR9S36B* |
| 3 | 119300001 | 119350000 | 2.1253 | *OR9S39* |
| 3 | 78550001 | 78600000 | 2.1221 | *DYNLT5* |
| 3 | 78550001 | 78600000 | 2.1221 | *SGIP1* |
| 20 | 7350001 | 7400000 | 2.1205 | *U6* |
| 22 | 43100001 | 43150000 | 2.1185 | *FLNB* |
| 22 | 43100001 | 43150000 | 2.1185 | *DNASE1L3* |
| 7 | 13700001 | 13750000 | 2.1181 | *OR7E201* |
| 5 | 59900001 | 59950000 | 2.1143 | *TESPA1* |
| 4 | 16150001 | 16200000 | 2.1136 | *ICA1* |
| 4 | 16150001 | 16200000 | 2.1136 | *GLCCI1* |
| 8 | 18550001 | 18600000 | 2.1136 | *TUSC1* |
| 1 | 83500001 | 83550000 | 2.113 | *KLHL6* |
| 20 | 15200001 | 15250000 | 2.1122 | *RNF180* |
| 19 | 52050001 | 52100000 | 2.1101 | *ENDOV* |
| 19 | 52050001 | 52100000 | 2.1101 | *NPTX1* |
| 8 | 75400001 | 75450000 | 2.1087 | *UBAP2* |
| 22 | 49650001 | 49700000 | 2.108 | *DOCK3* |
| 4 | 31450001 | 31500000 | 2.1072 | *IL6* |
| 17 | 14450001 | 14500000 | 2.1069 | *GAB1* |
| 10 | 29450001 | 29500000 | 2.1059 | *FMN1* |
| 25 | 3400001 | 3450000 | 2.1043 | *SRL* |
| 25 | 3400001 | 3450000 | 2.1043 | *TFAP4* |
| 23 | 13250001 | 13300000 | 2.1024 | *KIF6* |
| 29 | 24850001 | 24900000 | 2.1014 | *NAV2* |
| 16 | 72100001 | 72150000 | 2.1004 | *RCOR3* |
| 16 | 71550001 | 71600000 | 2.0998 | *LPGAT1* |
| 10 | 88000001 | 88050000 | 2.0984 | *VASH1* |
| 10 | 88000001 | 88050000 | 2.0984 | *ANGEL1* |
| 2 | 35850001 | 35900000 | 2.0982 | *RBMS1* |
| 5 | 10250001 | 10300000 | 2.098 | *MYF5* |
| 5 | 10250001 | 10300000 | 2.098 | *MYF6* |

| | | | | |
|---|---|---|---|---|
| 5 | 10250001 | 10300000 | 2.098 | *PTPRQ* |
| 12 | 16800001 | 16850000 | 2.0977 | *HTR2A* |
| 1 | 83300001 | 83350000 | 2.0976 | *YEATS2* |
| 1 | 83300001 | 83350000 | 2.0976 | *MAP6D1* |
| 1 | 83300001 | 83350000 | 2.0976 | *PARL* |
| 15 | 1500001 | 1550000 | 2.0973 | *ANKRD49* |
| 15 | 1500001 | 1550000 | 2.0973 | *MRE11* |
| 5 | 53850001 | 53900000 | 2.0969 | *SLC16A7* |
| 13 | 62750001 | 62800000 | 2.0941 | *BPIFB1* |
| 13 | 62750001 | 62800000 | 2.0941 | *BPIFB5* |
| 15 | 39700001 | 39750000 | 2.0933 | *TEAD1* |
| 4 | 49700001 | 49750000 | 2.0922 | *NME8* |
| 4 | 49700001 | 49750000 | 2.0922 | *SFRP4* |
| 19 | 16900001 | 16950000 | 2.092 | *ASIC2* |
| 8 | 106100001 | 106150000 | 2.0896 | *ASTN2* |
| 10 | 29400001 | 29450000 | 2.0875 | *FMN1* |
| 17 | 55750001 | 55800000 | 2.0855 | *PRKAB1* |
| 17 | 55750001 | 55800000 | 2.0855 | *TMEM233* |
| 16 | 43850001 | 43900000 | 2.085 | *TMEM201* |
| 16 | 43850001 | 43900000 | 2.085 | *PIK3CD* |
| 16 | 43850001 | 43900000 | 2.085 | *U6* |
| 2 | 35950001 | 36000000 | 2.0849 | *RBMS1* |
| 16 | 45600001 | 45650000 | 2.0849 | *CAMTA1* |
| 23 | 9100001 | 9150000 | 2.0841 | *TCP11* |
| 10 | 72850001 | 72900000 | 2.0834 | *SIX4* |
| 10 | 72850001 | 72900000 | 2.0834 | *MNAT1* |
| 11 | 45700001 | 45750000 | 2.0805 | *UXS1* |
| 2 | 36950001 | 37000000 | 2.0801 | *BAZ2B* |
| 9 | 68250001 | 68300000 | 2.0788 | *L3MBTL3* |
| 9 | 68250001 | 68300000 | 2.0788 | *SAMD3* |
| 1 | 49950001 | 50000000 | 2.0787 | *ALCAM* |
| 18 | 14450001 | 14500000 | 2.0784 | *SPG7* |
| 18 | 14450001 | 14500000 | 2.0784 | *RPL13* |
| 18 | 14450001 | 14500000 | 2.0784 | *SNORD68* |
| 16 | 33800001 | 33850000 | 2.0783 | *SDCCAG8* |
| 11 | 20650001 | 20700000 | 2.0782 | *ATL2* |
| 29 | 24900001 | 24950000 | 2.0775 | *NAV2* |
| 10 | 32250001 | 32300000 | 2.0766 | *CDIN1* |
| 27 | 33300001 | 33350000 | 2.0747 | *ASH2L* |
| 27 | 33300001 | 33350000 | 2.0747 | *LSM1* |
| 27 | 33300001 | 33350000 | 2.0747 | *STAR* |
| 27 | 33300001 | 33350000 | 2.0747 | *BAG4* |
| 23 | 11050001 | 11100000 | 2.0734 | *PIM1* |
| 13 | 16650001 | 16700000 | 2.0719 | *U6* |
| 2 | 36400001 | 36450000 | 2.0699 | *PLA2R1* |
| 29 | 36300001 | 36350000 | 2.0698 | *APLP2* |
| 2 | 5900001 | 5950000 | 2.0697 | *INPP1* |
| 16 | 45500001 | 45550000 | 2.0696 | *UTS2* |
| 22 | 49700001 | 49750000 | 2.0678 | *MAPKAPK3* |
| 22 | 49700001 | 49750000 | 2.0678 | *CISH* |
| 29 | 35900001 | 35950000 | 2.0623 | *U6* |
| 15 | 40750001 | 40800000 | 2.0616 | *USP47* |

| | | | | |
|---|---|---|---|---|
| 10 | 32500001 | 32550000 | 2.0613 | *MEIS2* |
| 23 | 37300001 | 37350000 | 2.0606 | *CDKAL1* |
| 17 | 40350001 | 40400000 | 2.0599 | *RXFP1* |
| 5 | 66200001 | 66250000 | 2.0571 | *IGF1* |
| 19 | 11400001 | 11450000 | 2.0571 | *BRIP1* |
| 13 | 63100001 | 63150000 | 2.0558 | *NECAB3* |
| 13 | 63100001 | 63150000 | 2.0558 | *E2F1* |
| 13 | 63100001 | 63150000 | 2.0558 | *C13H20orf144* |
| 23 | 14300001 | 14350000 | 2.0533 | *LRFN2* |
| 23 | 14300001 | 14350000 | 2.0533 | *U6* |
| 19 | 31700001 | 31750000 | 2.0527 | *HS3ST3A1* |
| 20 | 20250001 | 20300000 | 2.0524 | *PDE4D* |
| 5 | 105550001 | 105600000 | 2.052 | *RAD51AP1* |
| 5 | 105550001 | 105600000 | 2.052 | *C5H12orf4* |
| 5 | 105550001 | 105600000 | 2.052 | *DYRK4* |
| 7 | 23000001 | 23050000 | 2.0517 | *RAPGEF6* |
| 3 | 119250001 | 119300000 | 2.0504 | *OR9S23B* |
| 3 | 119250001 | 119300000 | 2.0504 | *OR9S42* |
| 11 | 99400001 | 99450000 | 2.05 | *LRRC8A* |
| 11 | 99400001 | 99450000 | 2.05 | *PHYHD1* |
| 11 | 99400001 | 99450000 | 2.05 | *NUP188* |
| 11 | 99400001 | 99450000 | 2.05 | *DOLK* |
| 11 | 97600001 | 97650000 | 2.0484 | *ZBTB34* |
| 11 | 97600001 | 97650000 | 2.0484 | *ZBTB43* |
| 23 | 700001 | 750000 | 2.0482 | *KHDRBS2* |
| 12 | 15300001 | 15350000 | 2.0479 | *GTF2F2* |
| 12 | 15300001 | 15350000 | 2.0479 | *KCTD4* |
| 12 | 7900001 | 7950000 | 2.0462 | *U6* |
| 3 | 46000001 | 46050000 | 2.0461 | *DPYD* |
| 14 | 44150001 | 44200000 | 2.046 | *PAG1* |
| 1 | 66750001 | 66800000 | 2.046 | *CSTA* |
| 1 | 66750001 | 66800000 | 2.046 | *MIX23* |
| 12 | 13350001 | 13400000 | 2.0447 | *ENOX1* |
| 10 | 30450001 | 30500000 | 2.0446 | *ZNF770* |
| 4 | 87900001 | 87950000 | 2.0425 | *ASB15* |
| 23 | 7600001 | 7650000 | 2.0425 | *SYNGAP1* |
| 23 | 7600001 | 7650000 | 2.0425 | *ZBTB9* |
| 22 | 33250001 | 33300000 | 2.0412 | *TAFA1* |
| 12 | 48800001 | 48850000 | 2.0406 | *KLF12* |
| 3 | 23650001 | 23700000 | 2.0382 | *ZNF697* |
| 4 | 49450001 | 49500000 | 2.0379 | *NRCAM* |
| 13 | 67700001 | 67750000 | 2.0361 | *FAM83D* |
| 13 | 67700001 | 67750000 | 2.0361 | *DHX35* |
| 13 | 67700001 | 67750000 | 2.0361 | *PPP1R16B* |
| 29 | 49150001 | 49200000 | 2.0352 | *TRPM5* |
| 29 | 49150001 | 49200000 | 2.0352 | *TSSC4* |
| 29 | 49150001 | 49200000 | 2.0352 | *CD81* |
| 23 | 11150001 | 11200000 | 2.0351 | *TBC1D22B* |
| 2 | 62150001 | 62200000 | 2.035 | *RAB3GAP1* |
| 22 | 37900001 | 37950000 | 2.0349 | *SYNPR* |
| 2 | 38500001 | 38550000 | 2.0342 | *ACVR1* |
| 2 | 34150001 | 34200000 | 2.0342 | *IFIH1* |

| | | | | |
|---|---|---|---|---|
| 2 | 34150001 | 34200000 | 2.0342 | *FAP* |
| 5 | 58400001 | 58450000 | 2.0333 | *OR6C1R* |
| 5 | 58400001 | 58450000 | 2.0333 | *OR6C280* |
| 27 | 40150001 | 40200000 | 2.0312 | *TOP2B* |
| 27 | 40150001 | 40200000 | 2.0312 | *RARB* |
| 2 | 71050001 | 71100000 | 2.0309 | *STEAP3* |
| 10 | 70650001 | 70700000 | 2.0304 | *TIMM9* |
| 10 | 70650001 | 70700000 | 2.0304 | *TOMM20L* |
| 10 | 70650001 | 70700000 | 2.0304 | *ARID4A* |
| 10 | 70650001 | 70700000 | 2.0304 | *KIAA0586* |
| 5 | 43600001 | 43650000 | 2.0302 | *MYRFL* |
| 4 | 10100001 | 10150000 | 2.0281 | *CDK6* |
| 15 | 38500001 | 38550000 | 2.0281 | *SPON1* |
| 14 | 3200001 | 3250000 | 2.0277 | *TRAPPC9* |
| 11 | 45550001 | 45600000 | 2.0273 | *ST6GAL2* |
| 10 | 35700001 | 35750000 | 2.0271 | *BMF* |
| 5 | 66150001 | 66200000 | 2.0267 | *IGF1* |
| 11 | 99100001 | 99150000 | 2.0263 | *SPTAN1* |
| 22 | 35750001 | 35800000 | 2.0263 | *MAGI1* |
| 13 | 12600001 | 12650000 | 2.0263 | *USP6NL* |
| 22 | 13250001 | 13300000 | 2.0258 | *ENTPD3* |
| 22 | 13250001 | 13300000 | 2.0258 | *RPL14* |
| 3 | 46200001 | 46250000 | 2.0255 | *DPYD* |
| 13 | 39650001 | 39700000 | 2.0229 | *CFAP61* |
| 23 | 7550001 | 7600000 | 2.0227 | *KIFC1* |
| 23 | 7550001 | 7600000 | 2.0227 | *PHF1* |
| 23 | 7550001 | 7600000 | 2.0227 | *SYNGAP1* |
| 23 | 7550001 | 7600000 | 2.0227 | *CUTA* |
| 3 | 117450001 | 117500000 | 2.0225 | *ESPNL* |
| 3 | 117450001 | 117500000 | 2.0225 | *KLHL30* |
| 3 | 117450001 | 117500000 | 2.0225 | *ERFE* |
| 20 | 3900001 | 3950000 | 2.0224 | *STK10* |
| 7 | 44250001 | 44300000 | 2.0223 | *BTBD2* |
| 7 | 44250001 | 44300000 | 2.0223 | *CSNK1G2* |
| 18 | 10250001 | 10300000 | 2.0217 | *OSGIN1* |
| 18 | 10250001 | 10300000 | 2.0217 | *MLYCD* |
| 23 | 11200001 | 11250000 | 2.021 | *TBC1D22B* |
| 23 | 11200001 | 11250000 | 2.021 | *RNF8* |
| 8 | 91250001 | 91300000 | 2.0207 | *RNF20* |
| 24 | 56800001 | 56850000 | 2.0206 | *FECH* |
| 24 | 56800001 | 56850000 | 2.0206 | *NARS1* |
| 23 | 37450001 | 37500000 | 2.0188 | *E2F3* |
| 23 | 11100001 | 11150000 | 2.0186 | *TMEM217* |
| 23 | 11100001 | 11150000 | 2.0186 | *TBC1D22B* |
| 25 | 13900001 | 13950000 | 2.0171 | *BMERB1* |
| 25 | 35300001 | 35350000 | 2.014 | *COL26A1* |
| 12 | 60650001 | 60700000 | 2.0137 | *SLITRK6* |
| 15 | 29000001 | 29050000 | 2.0132 | *MPZL2* |
| 15 | 29000001 | 29050000 | 2.0132 | *CD3E* |
| 15 | 29000001 | 29050000 | 2.0132 | *UBE4A* |
| 15 | 29000001 | 29050000 | 2.0132 | *CD3G* |
| 15 | 29000001 | 29050000 | 2.0132 | *CD3D* |

| | | | | |
|---|---|---|---|---|
| 15 | 29000001 | 29050000 | 2.0132 | *U6* |
| 7 | 12550001 | 12600000 | 2.0106 | *NFIX* |
| 14 | 1150001 | 1200000 | 2.0096 | *ZC3H3* |
| 16 | 43300001 | 43350000 | 2.0092 | *U6* |
| 12 | 50550001 | 50600000 | 2.0076 | *COMMD6* |
| 12 | 50550001 | 50600000 | 2.0076 | *UCHL3* |
| 7 | 44300001 | 44350000 | 2.0074 | *BTBD2* |
| 15 | 1450001 | 1500000 | 2.0069 | *MRE11* |
| 4 | 12000001 | 12050000 | 2.0054 | *SGCE* |
| 2 | 80250001 | 80300000 | 2.0054 | *NABP1* |
| 13 | 39750001 | 39800000 | 2.0046 | *CFAP61* |
| 29 | 49000001 | 49050000 | 2.0041 | *KCNQ1* |
| 13 | 67750001 | 67800000 | 2.0035 | *DHX35* |
| 13 | 53600001 | 53650000 | 2.003 | *GINS1* |
| 24 | 56000001 | 56050000 | 2.0027 | *WDR7* |
| 1 | 55950001 | 56000000 | 2.0013 | *NECTIN3* |
| 10 | 64950001 | 65000000 | 2.0005 | *C10H15orf48* |
| 10 | 64950001 | 65000000 | 2.0005 | *SLC30A4* |
| 8 | 89600001 | 89650000 | 1.9996 | *NXNL2* |
| 8 | 89600001 | 89650000 | 1.9996 | *SPIN1* |
| 15 | 2000001 | 2050000 | 1.9994 | *GRIA4* |
| 24 | 250001 | 300000 | 1.9964 | *OR9M1D* |
| 24 | 250001 | 300000 | 1.9964 | *OR5W29P* |
| 26 | 9500001 | 9550000 | 1.9962 | *PTEN* |
| 7 | 6550001 | 6600000 | 1.9959 | *EPS15L1* |
| 18 | 52950001 | 53000000 | 1.9956 | *ERCC2* |
| 14 | 23850001 | 23900000 | 1.9956 | *BPNT2* |
| 18 | 52950001 | 53000000 | 1.9956 | *CKM* |
| 18 | 52950001 | 53000000 | 1.9956 | *KLC3* |
| 18 | 52950001 | 53000000 | 1.9956 | *MARK4* |
| 18 | 11450001 | 11500000 | 1.9951 | *GSE1* |
| 15 | 31350001 | 31400000 | 1.9946 | *GRIK4* |
| 29 | 12100001 | 12150000 | 1.9939 | *DLG2* |
| 14 | 44000001 | 44050000 | 1.9928 | *ZNF704* |
| 14 | 34800001 | 34850000 | 1.9921 | *EYA1* |
| 10 | 69200001 | 69250000 | 1.9917 | *OTX2* |
| 9 | 73000001 | 73050000 | 1.9915 | *HBS1L* |
| 9 | 73000001 | 73050000 | 1.9915 | *ALDH8A1* |
| 7 | 22900001 | 22950000 | 1.9908 | *RAPGEF6* |
| 9 | 34500001 | 34550000 | 1.9886 | *NT5DC1* |
| 9 | 34500001 | 34550000 | 1.9886 | *COL10A1* |
| 10 | 70700001 | 70750000 | 1.9874 | *KIAA0586* |
| 26 | 13400001 | 13450000 | 1.9864 | *TNKS2* |
| 26 | 13400001 | 13450000 | 1.9864 | *FGFBP3* |
| 1 | 106450001 | 106500000 | 1.9858 | *PPM1L* |
| 29 | 42250001 | 42300000 | 1.9856 | *NAA40* |
| 29 | 42250001 | 42300000 | 1.9856 | *RCOR2* |
| 29 | 42250001 | 42300000 | 1.9856 | *MARK2* |
| 26 | 9450001 | 9500000 | 1.9852 | *PTEN* |
| 8 | 94700001 | 94750000 | 1.9832 | *ABCA1* |
| 2 | 42150001 | 42200000 | 1.9828 | *GALNT13* |
| 4 | 14350001 | 14400000 | 1.9826 | *DLX6* |

| | | | | |
|---|---|---|---|---|
| 15 | 31300001 | 31350000 | 1.9823 | *GRIK4* |
| 9 | 13400001 | 13450000 | 1.9788 | *U6* |
| 16 | 66700001 | 66750000 | 1.9785 | *HMCN1* |
| 1 | 54050001 | 54100000 | 1.9782 | *DPPA2* |
| 1 | 54050001 | 54100000 | 1.9782 | *DPPA4* |
| 26 | 22100001 | 22150000 | 1.9777 | *BTRC* |
| 1 | 22900001 | 22950000 | 1.9745 | *ABCC13* |
| 1 | 22900001 | 22950000 | 1.9745 | *RBM11* |
| 22 | 37850001 | 37900000 | 1.9743 | *SYNPR* |
| 6 | 45150001 | 45200000 | 1.9739 | *SLC34A2* |
| 6 | 45150001 | 45200000 | 1.9739 | *U6* |
| 2 | 71000001 | 71050000 | 1.9738 | *STEAP3* |
| 15 | 48700001 | 48750000 | 1.9718 | *OR51F1* |
| 15 | 48700001 | 48750000 | 1.9718 | *OR51F4* |
| 7 | 46850001 | 46900000 | 1.9711 | *MACROH2A1* |
| 7 | 21550001 | 21600000 | 1.97 | *SEPTIN8* |
| 7 | 21550001 | 21600000 | 1.97 | *CCNI2* |
| 10 | 27850001 | 27900000 | 1.9696 | *OR4F73* |
| 10 | 27850001 | 27900000 | 1.9696 | *OR4F70* |
| 9 | 7800001 | 7850000 | 1.9695 | *ADGRB3* |
| 16 | 44350001 | 44400000 | 1.9693 | *GPR157* |
| 24 | 8300001 | 8350000 | 1.9675 | *CCDC102B* |
| 1 | 18600001 | 18650000 | 1.9671 | *TMPRSS15* |
| 10 | 30350001 | 30400000 | 1.9667 | *AQR* |
| 8 | 81700001 | 81750000 | 1.9652 | *FANCC* |
| 4 | 51700001 | 51750000 | 1.9644 | *MET* |
| 13 | 39000001 | 39050000 | 1.964 | *SLC24A3* |
| 11 | 80950001 | 81000000 | 1.9636 | *VSNL1* |
| 14 | 22850001 | 22900000 | 1.9629 | *XKR4* |
| 18 | 55200001 | 55250000 | 1.961 | *SULT2B1* |
| 18 | 55200001 | 55250000 | 1.961 | *FAM83E* |
| 5 | 47150001 | 47200000 | 1.9599 | *GRIP1* |
| 9 | 83700001 | 83750000 | 1.9597 | *GRM1* |
| 7 | 38100001 | 38150000 | 1.9597 | *TSPAN17* |
| 7 | 38100001 | 38150000 | 1.9597 | *EIF4E1B* |
| 7 | 38100001 | 38150000 | 1.9597 | *SNCB* |
| 14 | 34850001 | 34900000 | 1.9573 | *EYA1* |
| 15 | 31250001 | 31300000 | 1.9564 | *GRIK4* |
| 20 | 31800001 | 31850000 | 1.9556 | *CCDC152* |
| 16 | 44300001 | 44350000 | 1.9554 | *GPR157* |
| 16 | 44300001 | 44350000 | 1.9554 | *U1* |
| 10 | 46500001 | 46550000 | 1.9535 | *USP3* |
| 1 | 53950001 | 54000000 | 1.9532 | *U6* |
| 4 | 14450001 | 14500000 | 1.9489 | *SDHAF3* |
| 6 | 35850001 | 35900000 | 1.9486 | *FAM13A* |
| 11 | 99150001 | 99200000 | 1.9475 | *DYNC2I2* |
| 11 | 99150001 | 99200000 | 1.9475 | *SPTAN1* |
| 10 | 76700001 | 76750000 | 1.9468 | *PPP1R36* |
| 11 | 80850001 | 80900000 | 1.9464 | *SMC6* |
| 11 | 80850001 | 80900000 | 1.9464 | *GEN1* |
| 16 | 26000001 | 26050000 | 1.9462 | *HHIPL2* |
| 16 | 26000001 | 26050000 | 1.9462 | *TAF1A* |

| | | | | |
|---|---|---|---|---|
| 26 | 43750001 | 43800000 | 1.9461 | *CHST15* |
| 4 | 69600001 | 69650000 | 1.9454 | *SNX10* |
| 4 | 13550001 | 13600000 | 1.9451 | *SLC25A13* |
| 8 | 91150001 | 91200000 | 1.9439 | *ALDOB* |
| 8 | 91150001 | 91200000 | 1.9439 | *PGAP4* |
| 2 | 43750001 | 43800000 | 1.9431 | *FMNL2* |
| 26 | 24900001 | 24950000 | 1.9424 | *CFAP58* |
| 14 | 44200001 | 44250000 | 1.9419 | *PAG1* |
| 8 | 83800001 | 83850000 | 1.9404 | *IARS1* |
| 7 | 42100001 | 42150000 | 1.9404 | *OR2T27* |
| 8 | 83800001 | 83850000 | 1.9404 | *ZNF484* |
| 7 | 42100001 | 42150000 | 1.9404 | *OR2T2* |
| 7 | 42100001 | 42150000 | 1.9404 | *OR2T11* |
| 8 | 76750001 | 76800000 | 1.94 | *FRMD3* |
| 1 | 45700001 | 45750000 | 1.9394 | *IMPG2* |
| 12 | 21450001 | 21500000 | 1.9375 | *NEK5* |
| 17 | 20300001 | 20350000 | 1.9373 | *PCDH18* |
| 16 | 33750001 | 33800000 | 1.9369 | *SDCCAG8* |
| 17 | 33150001 | 33200000 | 1.9358 | *ANKRD50* |
| 28 | 3950001 | 4000000 | 1.9319 | *C28H1orf131* |
| 28 | 3950001 | 4000000 | 1.9319 | *TRIM67* |
| 1 | 45950001 | 46000000 | 1.9313 | *SENP7* |
| 1 | 45950001 | 46000000 | 1.9313 | *TRMT10C* |
| 18 | 22400001 | 22450000 | 1.9312 | *FTO* |
| 25 | 3250001 | 3300000 | 1.9312 | *ADCY9* |
| 29 | 12900001 | 12950000 | 1.9278 | *FAM181B* |
| 26 | 34300001 | 34350000 | 1.9261 | *NHLRC2* |
| 26 | 34300001 | 34350000 | 1.9261 | *DCLRE1A* |
| 3 | 78500001 | 78550000 | 1.9257 | *DYNLT5* |
| 3 | 78500001 | 78550000 | 1.9257 | *DNAI4* |
| 3 | 78500001 | 78550000 | 1.9257 | *INSL5* |
| 7 | 93750001 | 93800000 | 1.9256 | *KIAA0825* |
| 1 | 83600001 | 83650000 | 1.925 | *SNORA63* |
| 1 | 83600001 | 83650000 | 1.925 | *SNORA63* |
| 2 | 71300001 | 71350000 | 1.9246 | *SCTR* |
| 2 | 37050001 | 37100000 | 1.9228 | *WDSUB1* |
| 10 | 79550001 | 79600000 | 1.9223 | *RDH11* |
| 10 | 79550001 | 79600000 | 1.9223 | *RDH12* |
| 10 | 79550001 | 79600000 | 1.9223 | *VTI1B* |
| 10 | 79550001 | 79600000 | 1.9223 | *ZFYVE26* |
| 18 | 14200001 | 14250000 | 1.9196 | *ACSF3* |
| 12 | 13400001 | 13450000 | 1.9195 | *ENOX1* |
| 20 | 32600001 | 32650000 | 1.9182 | *RIMOC1* |
| 20 | 32600001 | 32650000 | 1.9182 | *FBXO4* |
| 14 | 66400001 | 66450000 | 1.9178 | *LAPTM4B* |
| 26 | 33200001 | 33250000 | 1.917 | *VTI1A* |
| 18 | 52400001 | 52450000 | 1.9169 | *PVR* |
| 18 | 52400001 | 52450000 | 1.9169 | *IGSF23* |
| 18 | 52400001 | 52450000 | 1.9169 | *CEACAM19* |
| 16 | 3500001 | 3550000 | 1.9166 | *SLC41A1* |
| 16 | 3500001 | 3550000 | 1.9166 | *RAB29* |
| 12 | 50600001 | 50650000 | 1.9159 | *UCHL3* |

| | | | | |
|---|---|---|---|---|
| 16 | 43100001 | 43150000 | 1.9155 | *CENPS* |
| 16 | 43100001 | 43150000 | 1.9155 | *PEX14* |
| 16 | 43100001 | 43150000 | 1.9155 | *DFFA* |
| 16 | 43100001 | 43150000 | 1.9155 | *CORT* |
| 7 | 22850001 | 22900000 | 1.9152 | *RAPGEF6* |
| 17 | 40300001 | 40350000 | 1.9152 | *ETFDH* |
| 17 | 40300001 | 40350000 | 1.9152 | *C17H4orf46* |
| 17 | 40300001 | 40350000 | 1.9152 | *RXFP1* |
| 10 | 62150001 | 62200000 | 1.9148 | *SLC12A1* |
| 10 | 62150001 | 62200000 | 1.9148 | *CTXN2* |
| 5 | 106450001 | 106500000 | 1.9145 | *TSPAN11* |
| 15 | 39800001 | 39850000 | 1.9131 | *TEAD1* |
| 26 | 43050001 | 43100000 | 1.9127 | *U6* |
| 7 | 18300001 | 18350000 | 1.9125 | *RFX2* |
| 15 | 39850001 | 39900000 | 1.9121 | *TEAD1* |
| 21 | 60300001 | 60350000 | 1.9113 | *SYNE3* |
| 2 | 42250001 | 42300000 | 1.9106 | *GALNT13* |
| 26 | 9400001 | 9450000 | 1.9095 | *ATAD1* |
| 22 | 48900001 | 48950000 | 1.9088 | *RPL29* |
| 22 | 48900001 | 48950000 | 1.9088 | *ACY1* |
| 2 | 62250001 | 62300000 | 1.9083 | *MAP3K19* |
| 9 | 42800001 | 42850000 | 1.9081 | *PDSS2* |
| 1 | 126800001 | 126850000 | 1.905 | *GK5* |
| 3 | 79750001 | 79800000 | 1.9036 | *LEPR* |
| 14 | 20650001 | 20700000 | 1.903 | *SNTG1* |
| 12 | 16300001 | 16350000 | 1.903 | *LRRC63* |
| 12 | 16300001 | 16350000 | 1.903 | *LCP1* |
| 18 | 22200001 | 22250000 | 1.9026 | *FTO* |
| 10 | 65300001 | 65350000 | 1.9019 | *DUOX2* |
| 10 | 65300001 | 65350000 | 1.9019 | *SORD* |
| 10 | 65300001 | 65350000 | 1.9019 | *DUOXA1* |
| 10 | 65300001 | 65350000 | 1.9019 | *DUOXA2* |
| 14 | 32550001 | 32600000 | 1.9018 | *C14H8orf34* |
| 29 | 47650001 | 47700000 | 1.9016 | *SHANK2* |
| 28 | 42250001 | 42300000 | 1.9016 | *GDF10* |
| 28 | 42250001 | 42300000 | 1.9016 | *RBP3* |
| 28 | 42250001 | 42300000 | 1.9016 | *GDF2* |
| 23 | 750001 | 800000 | 1.9007 | *KHDRBS2* |
| 7 | 12700001 | 12750000 | 1.9003 | *RNASEH2A* |
| 7 | 12700001 | 12750000 | 1.9003 | *MAST1* |
| 7 | 12700001 | 12750000 | 1.9003 | *PRDX2* |
| 7 | 12700001 | 12750000 | 1.9003 | *RTBDN* |
| 7 | 12700001 | 12750000 | 1.9003 | *HOOK2* |
| 7 | 12700001 | 12750000 | 1.9003 | *JUNB* |
| 7 | 51100001 | 51150000 | 1.899 | *NRG2* |
| 2 | 1150001 | 1200000 | 1.8988 | *CYFIP1* |
| 18 | 2500001 | 2550000 | 1.8987 | *ZFP1* |
| 18 | 2500001 | 2550000 | 1.8987 | *U6* |
| 18 | 46400001 | 46450000 | 1.8978 | *KMT2B* |
| 18 | 46400001 | 46450000 | 1.8978 | *UPK1A* |
| 18 | 46400001 | 46450000 | 1.8978 | *ZBTB32* |
| 16 | 66750001 | 66800000 | 1.8975 | *HMCN1* |

| | | | | |
|---|---|---|---|---|
| 7 | 42200001 | 42250000 | 1.8963 | *OR2T29* |
| 11 | 88400001 | 88450000 | 1.8945 | *MBOAT2* |
| 12 | 21750001 | 21800000 | 1.8927 | *SLC25A15* |
| 11 | 103050001 | 103100000 | 1.8926 | *RALGDS* |
| 11 | 103050001 | 103100000 | 1.8926 | *CEL* |
| 11 | 103050001 | 103100000 | 1.8926 | *GTF3C5* |
| 7 | 84100001 | 84150000 | 1.8916 | *EDIL3* |
| 20 | 14900001 | 14950000 | 1.8916 | *RGS7BP* |
| 4 | 72050001 | 72100000 | 1.8915 | *ADAM22* |
| 4 | 72050001 | 72100000 | 1.8915 | *FAM221A* |
| 3 | 18250001 | 18300000 | 1.8912 | *CRNN* |
| 20 | 5700001 | 5750000 | 1.8911 | *C20H5orf47* |
| 14 | 44250001 | 44300000 | 1.8906 | *PAG1* |
| 22 | 49550001 | 49600000 | 1.8877 | *DOCK3* |
| 24 | 41100001 | 41150000 | 1.8873 | *MTCL1* |
| 24 | 41100001 | 41150000 | 1.8873 | *RAB12* |
| 2 | 73300001 | 73350000 | 1.8869 | *CLASP1* |
| 9 | 8100001 | 8150000 | 1.8863 | *ADGRB3* |
| 1 | 83200001 | 83250000 | 1.8854 | *ABCC5* |
| 1 | 83200001 | 83250000 | 1.8854 | *PARL* |
| 1 | 56000001 | 56050000 | 1.8851 | *NECTIN3* |
| 16 | 43400001 | 43450000 | 1.8851 | *UBE4B* |
| 4 | 67950001 | 68000000 | 1.8847 | *JAZF1* |
| 15 | 1950001 | 2000000 | 1.8839 | *GRIA4* |
| 14 | 7100001 | 7150000 | 1.8834 | *ZFAT* |
| 24 | 57750001 | 57800000 | 1.8832 | *MALT1* |
| 16 | 65650001 | 65700000 | 1.8826 | *C16H1orf21* |
| 14 | 24650001 | 24700000 | 1.8821 | *CYP7A1* |
| 14 | 24650001 | 24700000 | 1.8821 | *U1* |
| 2 | 23750001 | 23800000 | 1.8817 | *RAPGEF4* |
| 25 | 41450001 | 41500000 | 1.8816 | *MICALL2* |
| 25 | 41450001 | 41500000 | 1.8816 | *INTS1* |
| 7 | 38050001 | 38100000 | 1.8808 | *CDHR2* |
| 7 | 38050001 | 38100000 | 1.8808 | *SNCB* |
| 7 | 38050001 | 38100000 | 1.8808 | *GPRIN1* |
| 21 | 45450001 | 45500000 | 1.8803 | *PRORP* |
| 21 | 45450001 | 45500000 | 1.8803 | *U6* |
| 10 | 37200001 | 37250000 | 1.8796 | *MAPKBP1* |
| 10 | 37200001 | 37250000 | 1.8796 | *PLA2G4B* |
| 10 | 37200001 | 37250000 | 1.8796 | *SPTBN5* |
| 10 | 37200001 | 37250000 | 1.8796 | *JMJD7* |
| 8 | 61600001 | 61650000 | 1.8793 | *FBXO10* |
| 8 | 61600001 | 61650000 | 1.8793 | *POLR1E* |
| 24 | 56700001 | 56750000 | 1.8786 | *ONECUT2* |
| 3 | 66000001 | 66050000 | 1.8768 | *IFI44* |
| 23 | 800001 | 850000 | 1.8761 | *KHDRBS2* |
| 7 | 6600001 | 6650000 | 1.8751 | *EPS15L1* |
| 10 | 46400001 | 46450000 | 1.8751 | *USP3* |
| 10 | 46400001 | 46450000 | 1.8751 | *FBXL22* |
| 16 | 1100001 | 1150000 | 1.8744 | *FMOD* |
| 24 | 700001 | 750000 | 1.8713 | *CTDP1* |
| 5 | 115250001 | 115300000 | 1.87 | *PRR5* |

| | | | | |
|---|---|---|---|---|
| 2 | 84550001 | 84600000 | 1.8699 | *DNAH7* |
| 18 | 11200001 | 11250000 | 1.8697 | *CIBAR2* |
| 9 | 44700001 | 44750000 | 1.8695 | *PREP* |
| 18 | 14900001 | 14950000 | 1.8694 | *SHCBP1* |
| 18 | 14250001 | 14300000 | 1.8686 | *CDH15* |
| 18 | 14250001 | 14300000 | 1.8686 | *SLC22A31* |
| 1 | 1800001 | 1850000 | 1.8671 | *ITSN1* |
| 18 | 14400001 | 14450000 | 1.8667 | *ANKRD11* |
| 26 | 33550001 | 33600000 | 1.8661 | *TCF7L2* |
| 7 | 44150001 | 44200000 | 1.8658 | *REXO1* |
| 7 | 44150001 | 44200000 | 1.8658 | *KLF16* |
| 7 | 44150001 | 44200000 | 1.8658 | *ABHD17A* |
| 2 | 31150001 | 31200000 | 1.8653 | *SCN3A* |
| 11 | 97650001 | 97700000 | 1.8651 | *RALGPS1* |
| 11 | 97650001 | 97700000 | 1.8651 | *ZBTB34* |
| 14 | 35400001 | 35450000 | 1.8646 | *MSC* |
| 5 | 17750001 | 17800000 | 1.864 | *C5H12orf50* |
| 25 | 3450001 | 3500000 | 1.8638 | *TFAP4* |
| 25 | 3450001 | 3500000 | 1.8638 | *GLIS2* |
| 26 | 33450001 | 33500000 | 1.8637 | *TCF7L2* |
| 12 | 13450001 | 13500000 | 1.8632 | *ENOX1* |
| 4 | 69700001 | 69750000 | 1.8622 | *CBX3* |
| 4 | 69700001 | 69750000 | 1.8622 | *HNRNPA2B1* |
| 4 | 69700001 | 69750000 | 1.8622 | *NFE2L3* |
| 20 | 14350001 | 14400000 | 1.8619 | *U4* |
| 18 | 14350001 | 14400000 | 1.8614 | *ANKRD11* |
| 26 | 35300001 | 35350000 | 1.8597 | *FHIP2A* |
| 26 | 13450001 | 13500000 | 1.8583 | *BTAF1* |
| 19 | 9900001 | 9950000 | 1.8579 | *PPM1E* |
| 19 | 9900001 | 9950000 | 1.8579 | *TRIM37* |
| 12 | 19200001 | 19250000 | 1.8555 | *EBPL* |
| 12 | 19200001 | 19250000 | 1.8555 | *KPNA3* |
| 16 | 72050001 | 72100000 | 1.8554 | *RCOR3* |
| 20 | 18400001 | 18450000 | 1.8554 | *ERCC8* |
| 4 | 12050001 | 12100000 | 1.8541 | *SGCE* |
| 4 | 12050001 | 12100000 | 1.8541 | *PEG10* |
| 4 | 12050001 | 12100000 | 1.8541 | *U6* |
| 1 | 50150001 | 50200000 | 1.8538 | *ALCAM* |
| 3 | 103650001 | 103700000 | 1.8534 | *CCDC30* |
| 7 | 38600001 | 38650000 | 1.8533 | *NSD1* |
| 7 | 38600001 | 38650000 | 1.8533 | *U6* |
| 14 | 9000001 | 9050000 | 1.8502 | *KCNQ3* |
| 2 | 71500001 | 71550000 | 1.8499 | *PTPN4* |
| 13 | 63250001 | 63300000 | 1.8489 | *CHMP4B* |
| 22 | 37450001 | 37500000 | 1.8479 | *ATXN7* |
| 12 | 13300001 | 13350000 | 1.8477 | *ENOX1* |
| 9 | 15650001 | 15700000 | 1.8475 | *IMPG1* |
| 15 | 1550001 | 1600000 | 1.8465 | *AASDHPPT* |
| 17 | 9750001 | 9800000 | 1.8462 | *NR3C2* |
| 3 | 86850001 | 86900000 | 1.846 | *U6* |
| 5 | 24800001 | 24850000 | 1.8459 | *FGD6* |
| 20 | 3950001 | 4000000 | 1.8441 | *STK10* |

| | | | | |
|---|---|---|---|---|
| 20 | 3950001 | 4000000 | 1.8441 | *EFCAB9* |
| 20 | 3950001 | 4000000 | 1.8441 | *UBTD2* |
| 14 | 20600001 | 20650000 | 1.844 | *SNTG1* |
| 4 | 68550001 | 68600000 | 1.8435 | *HIBADH* |
| 7 | 24250001 | 24300000 | 1.842 | *CHSY3* |
| 19 | 39650001 | 39700000 | 1.8419 | *CACNB1* |
| 19 | 39650001 | 39700000 | 1.8419 | *PLXDC1* |
| 19 | 39650001 | 39700000 | 1.8419 | *RPL19* |
| 20 | 17700001 | 17750000 | 1.8419 | *U6* |
| 14 | 32350001 | 32400000 | 1.8409 | *C14H8orf34* |
| 5 | 103850001 | 103900000 | 1.8407 | *NCAPD2* |
| 5 | 103850001 | 103900000 | 1.8407 | *IFFO1* |
| 5 | 103850001 | 103900000 | 1.8407 | *NOP2* |
| 5 | 103850001 | 103900000 | 1.8407 | *GAPDH* |
| 5 | 103850001 | 103900000 | 1.8407 | *SCARNA10* |
| 26 | 25900001 | 25950000 | 1.8393 | *SORCS3* |
| 8 | 79250001 | 79300000 | 1.8392 | *GOLM1* |
| 8 | 79250001 | 79300000 | 1.8392 | *NAA35* |
| 18 | 25200001 | 25250000 | 1.8374 | *RSPRY1* |
| 18 | 25200001 | 25250000 | 1.8374 | *PSME3IP1* |
| 9 | 83650001 | 83700000 | 1.8371 | *GRM1* |
| 2 | 38750001 | 38800000 | 1.8353 | *ACVR1C* |
| 10 | 11100001 | 11150000 | 1.8353 | *SERINC5* |
| 17 | 22400001 | 22450000 | 1.8351 | *U6* |
| 2 | 19150001 | 19200000 | 1.8344 | *PDE11A* |
| 13 | 39600001 | 39650000 | 1.8344 | *CRNKL1* |
| 13 | 39600001 | 39650000 | 1.8344 | *CFAP61* |
| 13 | 39600001 | 39650000 | 1.8344 | *NAA20* |
| 12 | 16400001 | 16450000 | 1.8338 | *RUBCNL* |
| 12 | 13500001 | 13550000 | 1.833 | *ENOX1* |
| 3 | 78650001 | 78700000 | 1.8325 | *SGIP1* |
| 5 | 59300001 | 59350000 | 1.8323 | *OR10U1* |
| 5 | 59300001 | 59350000 | 1.8323 | *OR10U5* |
| 5 | 59300001 | 59350000 | 1.8323 | *OR10U1B* |
| 5 | 59300001 | 59350000 | 1.8323 | *OR10A7* |
| 18 | 16750001 | 16800000 | 1.8314 | *SIAH1* |
| 18 | 16750001 | 16800000 | 1.8314 | *LONP2* |
| 3 | 117400001 | 117450000 | 1.8304 | *SCLY* |
| 3 | 117400001 | 117450000 | 1.8304 | *ESPNL* |
| 9 | 73050001 | 73100000 | 1.8295 | *HBS1L* |
| 4 | 33800001 | 33850000 | 1.828 | *GRM3* |
| 7 | 38400001 | 38450000 | 1.8277 | *UIMC1* |
| 20 | 18050001 | 18100000 | 1.8275 | *ZSWIM6* |
| 21 | 45750001 | 45800000 | 1.8271 | *RALGAPA1* |
| 21 | 45750001 | 45800000 | 1.8271 | *INSM2* |
| 27 | 36850001 | 36900000 | 1.8255 | *KAT6A* |
| 1 | 126900001 | 126950000 | 1.8245 | *TFDP2* |
| 10 | 79100001 | 79150000 | 1.8245 | *GARIN2* |
| 10 | 79100001 | 79150000 | 1.8245 | *PALS1* |
| 3 | 118400001 | 118450000 | 1.8243 | *HDAC4* |
| 21 | 45300001 | 45350000 | 1.823 | *SRP54* |
| 15 | 40850001 | 40900000 | 1.8229 | *Metazoa_SRP* |

| | | | | |
|---|---|---|---|---|
| 11 | 99450001 | 99500000 | 1.8225 | *NUP188* |
| 2 | 26500001 | 26550000 | 1.8225 | *UBR3* |
| 2 | 26500001 | 26550000 | 1.8225 | *METTL5* |
| 11 | 99450001 | 99500000 | 1.8225 | *SH3GLB2* |
| 2 | 26500001 | 26550000 | 1.8225 | *SSB* |
| 2 | 26500001 | 26550000 | 1.8225 | *U6* |
| 28 | 33100001 | 33150000 | 1.8224 | *KCNMA1* |
| 21 | 45350001 | 45400000 | 1.8223 | *PPP2R3C* |
| 21 | 45350001 | 45400000 | 1.8223 | *FAM177A1* |
| 21 | 45350001 | 45400000 | 1.8223 | *PRORP* |
| 16 | 42500001 | 42550000 | 1.8222 | *EXOSC10* |
| 16 | 42500001 | 42550000 | 1.8222 | *MTOR* |
| 2 | 26700001 | 26750000 | 1.8221 | *FASTKD1* |
| 2 | 26700001 | 26750000 | 1.8221 | *KLHL41* |
| 6 | 35750001 | 35800000 | 1.8216 | *FAM13A* |
| 16 | 43350001 | 43400000 | 1.8214 | *UBE4B* |
| 20 | 750001 | 800000 | 1.8207 | *SLIT3* |
| 25 | 3700001 | 3750000 | 1.8206 | *UBALD1* |
| 22 | 7750001 | 7800000 | 1.8204 | *CLASP2* |
| 15 | 39900001 | 39950000 | 1.8201 | *TEAD1* |
| 27 | 36950001 | 37000000 | 1.819 | *KAT6A* |
| 4 | 16850001 | 16900000 | 1.8168 | *NXPH1* |
| 5 | 53700001 | 53750000 | 1.8163 | *SLC16A7* |
| 7 | 8950001 | 9000000 | 1.8149 | *OR7A114* |
| 2 | 71150001 | 71200000 | 1.8145 | *C2H2orf76* |
| 2 | 71150001 | 71200000 | 1.8145 | *DBI* |
| 6 | 95150001 | 95200000 | 1.8142 | *CFAP299* |
| 6 | 2700001 | 2750000 | 1.8132 | *NAF1* |
| 6 | 2700001 | 2750000 | 1.8132 | *U6* |
| 11 | 26900001 | 26950000 | 1.8118 | *CAMKMT* |
| 29 | 18100001 | 18150000 | 1.8117 | *INTS4* |
| 5 | 35250001 | 35300000 | 1.8114 | *DBX2* |
| 7 | 38650001 | 38700000 | 1.811 | *NSD1* |
| 28 | 35450001 | 35500000 | 1.8108 | *CL46* |
| 17 | 14300001 | 14350000 | 1.8095 | *SMARCA5* |
| 17 | 1900001 | 1950000 | 1.8082 | *MAP9* |
| 2 | 37000001 | 37050000 | 1.808 | *BAZ2B* |
| 11 | 80750001 | 80800000 | 1.8079 | *MSGN1* |
| 18 | 48150001 | 48200000 | 1.8069 | *CATSPERG* |
| 18 | 48150001 | 48200000 | 1.8069 | *PSMD8* |
| 18 | 48150001 | 48200000 | 1.8069 | *GGN* |
| 18 | 48150001 | 48200000 | 1.8069 | *SPRED3* |
| 12 | 5200001 | 5250000 | 1.8065 | *PCDH17* |
| 10 | 70600001 | 70650000 | 1.8061 | *ARID4A* |
| 13 | 73050001 | 73100000 | 1.805 | *ADA* |
| 25 | 35350001 | 35400000 | 1.8037 | *COL26A1* |
| 12 | 14800001 | 14850000 | 1.8036 | *TSC22D1* |
| 6 | 3550001 | 3600000 | 1.8035 | *ANXA5* |
| 11 | 74400001 | 74450000 | 1.8031 | *ADCY3* |
| 11 | 74400001 | 74450000 | 1.8031 | *CENPO* |
| 11 | 74400001 | 74450000 | 1.8031 | *PTRHD1* |
| 11 | 74400001 | 74450000 | 1.8031 | *NCOA1* |

| | | | | |
|---|---|---|---|---|
| 19 | 51850001 | 51900000 | 1.803 | *RPTOR* |
| 13 | 39050001 | 39100000 | 1.8029 | *SLC24A3* |
| 20 | 17900001 | 17950000 | 1.8009 | *ZSWIM6* |
| 12 | 11950001 | 12000000 | 1.7999 | *VWA8* |
| 19 | 33850001 | 33900000 | 1.7998 | *ULK2* |
| 19 | 33850001 | 33900000 | 1.7998 | *ALDH3A1* |
| 19 | 33850001 | 33900000 | 1.7998 | *SLC47A2* |
| 15 | 43150001 | 43200000 | 1.7995 | *ZNF143* |
| 19 | 55100001 | 55150000 | 1.7995 | *ST6GALNAC1* |
| 15 | 43150001 | 43200000 | 1.7995 | *IPO7* |
| 5 | 17850001 | 17900000 | 1.7993 | *CEP290* |
| 22 | 48950001 | 49000000 | 1.7992 | *PCBP4* |
| 22 | 48950001 | 49000000 | 1.7992 | *RRP9* |
| 22 | 48950001 | 49000000 | 1.7992 | *PARP3* |
| 22 | 48950001 | 49000000 | 1.7992 | *ABHD14B* |
| 22 | 48950001 | 49000000 | 1.7992 | *ACY1* |
| 22 | 48950001 | 49000000 | 1.7992 | *ABHD14A* |
| 22 | 48950001 | 49000000 | 1.7992 | *IQCF2* |
| 22 | 48950001 | 49000000 | 1.7992 | *GPR62* |
| 15 | 29050001 | 29100000 | 1.799 | *MPZL2* |
| 15 | 29050001 | 29100000 | 1.799 | *UBE4A* |
| 15 | 29050001 | 29100000 | 1.799 | *ATP5MG* |
| 10 | 79600001 | 79650000 | 1.7986 | *ZFYVE26* |
| 9 | 15550001 | 15600000 | 1.7984 | *IMPG1* |
| 9 | 15550001 | 15600000 | 1.7984 | *MYO6* |
| 4 | 13500001 | 13550000 | 1.7978 | *SLC25A13* |
| 18 | 46450001 | 46500000 | 1.7978 | *ARHGAP33* |
| 18 | 46450001 | 46500000 | 1.7978 | *PROSER3* |
| 18 | 46450001 | 46500000 | 1.7978 | *LIN37* |
| 18 | 46450001 | 46500000 | 1.7978 | *KMT2B* |
| 18 | 46450001 | 46500000 | 1.7978 | *IGFLR1* |
| 18 | 46450001 | 46500000 | 1.7978 | *HSPB6* |
| 18 | 46450001 | 46500000 | 1.7978 | *U2AF1L4* |
| 18 | 46450001 | 46500000 | 1.7978 | *PSENEN* |
| 3 | 23750001 | 23800000 | 1.7972 | *HAO2* |
| 14 | 8950001 | 9000000 | 1.7943 | *KCNQ3* |
| 18 | 2750001 | 2800000 | 1.7924 | *BCNT2* |
| 18 | 2750001 | 2800000 | 1.7924 | *CFDP2* |
| 13 | 700001 | 750000 | 1.7922 | *TMX4* |
| 29 | 49050001 | 49100000 | 1.7919 | *KCNQ1* |
| 25 | 3350001 | 3400000 | 1.7919 | *SRL* |
| 4 | 40350001 | 40400000 | 1.791 | *CD36* |
| 21 | 45900001 | 45950000 | 1.7908 | *RALGAPA1* |
| 6 | 62800001 | 62850000 | 1.7908 | *KCTD8* |
| 29 | 28800001 | 28850000 | 1.7901 | *PKNOX2* |
| 22 | 42850001 | 42900000 | 1.7898 | *KCTD6* |
| 22 | 42850001 | 42900000 | 1.7898 | *ACOX2* |
| 8 | 76550001 | 76600000 | 1.7897 | *FRMD3* |
| 23 | 37400001 | 37450000 | 1.7897 | *CDKAL1* |
| 8 | 75650001 | 75700000 | 1.7896 | *KIF24* |
| 16 | 79650001 | 79700000 | 1.7896 | *TMEM9* |
| 16 | 79650001 | 79700000 | 1.7896 | *CACNA1S* |

| | | | | |
|---|---|---|---|---|
| 8 | 75650001 | 75700000 | 1.7896 | *UBAP1* |
| 25 | 20750001 | 20800000 | 1.789 | *USP31* |
| 9 | 81700001 | 81750000 | 1.7886 | *UTRN* |
| 5 | 77300001 | 77350000 | 1.7885 | *FGD4* |
| 14 | 75600001 | 75650000 | 1.7882 | *CNBD1* |
| 22 | 52900001 | 52950000 | 1.7881 | *LRRC2* |
| 22 | 52900001 | 52950000 | 1.7881 | *RTP3* |
| 2 | 17750001 | 17800000 | 1.7875 | *SESTD1* |
| 2 | 17750001 | 17800000 | 1.7875 | *CCDC141* |
| 8 | 98800001 | 98850000 | 1.7872 | *EPB41L4B* |
| 7 | 38750001 | 38800000 | 1.7865 | *NSD1* |
| 7 | 38750001 | 38800000 | 1.7865 | *MXD3* |
| 7 | 38750001 | 38800000 | 1.7865 | *RAB24* |
| 7 | 38750001 | 38800000 | 1.7865 | *PRELID1* |
| 1 | 83400001 | 83450000 | 1.7862 | *YEATS2* |
| 1 | 83400001 | 83450000 | 1.7862 | *KLHL24* |
| 25 | 1450001 | 1500000 | 1.7851 | *HS3ST6* |
| 26 | 8300001 | 8350000 | 1.784 | *PRKG1* |
| 3 | 43250001 | 43300000 | 1.7839 | *SLC35A3* |
| 3 | 43250001 | 43300000 | 1.7839 | *U5* |
| 4 | 49000001 | 49050000 | 1.7832 | *LAMB1* |
| 18 | 50550001 | 50600000 | 1.7823 | *BCKDHA* |
| 18 | 50550001 | 50600000 | 1.7823 | *DMAC2* |
| 18 | 50550001 | 50600000 | 1.7823 | *ERICH4* |
| 18 | 50550001 | 50600000 | 1.7823 | *B3GNT8* |
| 27 | 16500001 | 16550000 | 1.7821 | *FAT1* |
| 2 | 71100001 | 71150000 | 1.7818 | *C2H2orf76* |
| 7 | 93650001 | 93700000 | 1.7816 | *KIAA0825* |
| 2 | 110800001 | 110850000 | 1.7809 | *FARSB* |
| 2 | 110800001 | 110850000 | 1.7809 | *MOGAT1* |
| 7 | 43150001 | 43200000 | 1.7804 | *CDC34* |
| 7 | 43150001 | 43200000 | 1.7804 | *GZMM* |
| 7 | 43150001 | 43200000 | 1.7804 | *MADCAM1* |
| 7 | 43150001 | 43200000 | 1.7804 | *TPGS1* |
| 29 | 18150001 | 18200000 | 1.7802 | *AAMDC* |
| 29 | 18150001 | 18200000 | 1.7802 | *RSF1* |
| 2 | 41950001 | 42000000 | 1.7789 | *GALNT13* |
| 3 | 100700001 | 100750000 | 1.7773 | *TESK2* |
| 3 | 100700001 | 100750000 | 1.7773 | *TOE1* |
| 3 | 100700001 | 100750000 | 1.7773 | *MUTYH* |
| 3 | 100700001 | 100750000 | 1.7773 | *HPDL* |
| 24 | 45750001 | 45800000 | 1.7772 | *PSTPIP2* |
| 6 | 4750001 | 4800000 | 1.7756 | *PRDM5* |
| 4 | 87850001 | 87900000 | 1.7748 | *NDUFA5* |
| 4 | 87850001 | 87900000 | 1.7748 | *IQUB* |
| 11 | 101250001 | 101300000 | 1.7733 | *LAMC3* |
| 11 | 101250001 | 101300000 | 1.7733 | *AIF1L* |
| 16 | 79500001 | 79550000 | 1.7732 | *INAVA* |
| 16 | 79500001 | 79550000 | 1.7732 | *KIF21B* |
| 1 | 50100001 | 50150000 | 1.7717 | *ALCAM* |
| 2 | 47850001 | 47900000 | 1.7714 | *PTMA* |
| 2 | 15650001 | 15700000 | 1.7711 | *UBE2E3* |

| | | | | |
|---|---|---|---|---|
| 7 | 43050001 | 43100000 | 1.7705 | *THEG* |
| 7 | 43050001 | 43100000 | 1.7705 | *MIER2* |
| 10 | 65250001 | 65300000 | 1.7703 | *DUOX1* |
| 10 | 65250001 | 65300000 | 1.7703 | *SHF* |
| 10 | 65250001 | 65300000 | 1.7703 | *DUOXA1* |
| 21 | 68650001 | 68700000 | 1.7701 | *KIF26A* |
| 27 | 10350001 | 10400000 | 1.7699 | *SNORD22* |
| 2 | 113600001 | 113650000 | 1.7682 | *NYAP2* |
| 16 | 79600001 | 79650000 | 1.7678 | *CACNA1S* |
| 19 | 10100001 | 10150000 | 1.7674 | *SKA2* |
| 19 | 10100001 | 10150000 | 1.7674 | *PRR11* |
| 3 | 57450001 | 57500000 | 1.7663 | *CLCA4* |
| 5 | 6300001 | 6350000 | 1.7658 | *CSRP2* |
| 10 | 80650001 | 80700000 | 1.7649 | *ACTN1* |
| 6 | 117100001 | 117150000 | 1.7648 | *MAEA* |
| 6 | 117100001 | 117150000 | 1.7648 | *UVSSA* |
| 22 | 16350001 | 16400000 | 1.7646 | *ZKSCAN7* |
| 4 | 73400001 | 73450000 | 1.7644 | *ZNF804B* |
| 19 | 33750001 | 33800000 | 1.7643 | *AKAP10* |
| 16 | 45350001 | 45400000 | 1.7633 | *ERRFI1* |
| 6 | 60000001 | 60050000 | 1.763 | *APBB2* |
| 3 | 79150001 | 79200000 | 1.7628 | *PDE4B* |
| 2 | 30700001 | 30750000 | 1.7622 | *CSRNP3* |
| 23 | 850001 | 900000 | 1.7616 | *KHDRBS2* |
| 6 | 31300001 | 31350000 | 1.7615 | *GRID2* |
| 2 | 15700001 | 15750000 | 1.7614 | *UBE2E3* |
| 10 | 35750001 | 35800000 | 1.7613 | *BUB1B* |
| 10 | 35750001 | 35800000 | 1.7613 | *BMF* |
| 10 | 72900001 | 72950000 | 1.7589 | *MNAT1* |
| 7 | 13500001 | 13550000 | 1.7585 | *OR7G45* |
| 7 | 13500001 | 13550000 | 1.7585 | *OR7E192* |
| 20 | 5650001 | 5700000 | 1.7578 | *CPEB4* |
| 7 | 23100001 | 23150000 | 1.7556 | *CDC42SE2* |
| 19 | 43550001 | 43600000 | 1.7544 | *MEOX1* |
| 6 | 60050001 | 60100000 | 1.7539 | *APBB2* |
| 20 | 30550001 | 30600000 | 1.753 | *FGF10* |
| 13 | 53550001 | 53600000 | 1.7529 | *GINS1* |
| 16 | 79550001 | 79600000 | 1.7528 | *KIF21B* |
| 16 | 79550001 | 79600000 | 1.7528 | *CACNA1S* |
| 21 | 29000001 | 29050000 | 1.7524 | *PCSK6* |
| 5 | 60950001 | 61000000 | 1.7517 | *CFAP54* |
| 5 | 67450001 | 67500000 | 1.7517 | *NT5DC3* |
| 16 | 80550001 | 80600000 | 1.7515 | *LMOD1* |
| 16 | 80550001 | 80600000 | 1.7515 | *IPO9* |
| 16 | 80550001 | 80600000 | 1.7515 | *SHISA4* |
| 27 | 16450001 | 16500000 | 1.751 | *FAT1* |
| 19 | 10050001 | 10100000 | 1.7508 | *TRIM37* |
| 19 | 10050001 | 10100000 | 1.7508 | *SKA2* |
| 18 | 14500001 | 14550000 | 1.7502 | *DPEP1* |
| 18 | 14500001 | 14550000 | 1.7502 | *CPNE7* |
| 20 | 71100001 | 71150000 | 1.7489 | *CLPTM1L* |
| 20 | 71100001 | 71150000 | 1.7489 | *TERT* |

| | | | | |
|---|---|---|---|---|
| 2 | 37700001 | 37750000 | 1.7486 | *PKP4* |
| 25 | 1300001 | 1350000 | 1.7484 | *MAPK8IP3* |
| 25 | 1300001 | 1350000 | 1.7484 | *JPT2* |
| 25 | 1300001 | 1350000 | 1.7484 | *NME3* |
| 7 | 43200001 | 43250000 | 1.7477 | *HCN2* |
| 7 | 43200001 | 43250000 | 1.7477 | *POLRMT* |
| 7 | 43200001 | 43250000 | 1.7477 | *BSG* |
| 4 | 13100001 | 13150000 | 1.7476 | *DYNC1I1* |
| 9 | 42900001 | 42950000 | 1.7476 | *BEND3* |
| 9 | 42900001 | 42950000 | 1.7476 | *MTRES1* |
| 16 | 80800001 | 80850000 | 1.746 | *CSRP1* |
| 1 | 83850001 | 83900000 | 1.7453 | *MCF2L2* |
| 10 | 27750001 | 27800000 | 1.7446 | *OR4G9* |
| 10 | 27750001 | 27800000 | 1.7446 | *OR4G2* |
| 16 | 71950001 | 72000000 | 1.7441 | *TRAF5* |
| 16 | 71950001 | 72000000 | 1.7441 | *RD3* |
| 17 | 51750001 | 51800000 | 1.7426 | *CCDC92* |
| 17 | 51750001 | 51800000 | 1.7426 | *DNAH10* |
| 2 | 34650001 | 34700000 | 1.7414 | *SLC4A10* |
| 8 | 90800001 | 90850000 | 1.7406 | *PLPPR1* |
| 16 | 12550001 | 12600000 | 1.7395 | *RGS13* |
| 2 | 42200001 | 42250000 | 1.7394 | *GALNT13* |
| 12 | 18250001 | 18300000 | 1.7391 | *RCBTB2* |
| 15 | 1400001 | 1450000 | 1.739 | *MRE11* |
| 15 | 1400001 | 1450000 | 1.739 | *GPR83* |
| 9 | 42850001 | 42900000 | 1.7388 | *PDSS2* |
| 9 | 42850001 | 42900000 | 1.7388 | *BEND3* |
| 29 | 12750001 | 12800000 | 1.7383 | *PRCP* |
| 5 | 65400001 | 65450000 | 1.7379 | *MYBPC1* |
| 5 | 107000001 | 107050000 | 1.7366 | *WASHC1* |
| 5 | 107000001 | 107050000 | 1.7366 | *IQSEC3* |
| 8 | 84000001 | 84050000 | 1.7358 | *CENPP* |
| 8 | 84000001 | 84050000 | 1.7358 | *OGN* |
| 8 | 84000001 | 84050000 | 1.7358 | *OMD* |
| 20 | 14500001 | 14550000 | 1.7346 | *CWC27* |
| 8 | 39350001 | 39400000 | 1.734 | *JAK2* |
| 8 | 39350001 | 39400000 | 1.734 | *INSL6* |
| 12 | 15150001 | 15200000 | 1.7339 | *GPALPP1* |
| 12 | 15150001 | 15200000 | 1.7339 | *NUFIP1* |
| 1 | 118650001 | 118700000 | 1.7337 | *TM4SF4* |
| 20 | 3700001 | 3750000 | 1.733 | *FBXW11* |
| 3 | 29600001 | 29650000 | 1.7328 | *PHTF1* |
| 3 | 29600001 | 29650000 | 1.7328 | *RSBN1* |
| 18 | 22150001 | 22200000 | 1.7326 | *FTO* |
| 25 | 2900001 | 2950000 | 1.7315 | *CLUAP1* |
| 25 | 2900001 | 2950000 | 1.7315 | *NLRC3* |
| 3 | 29550001 | 29600000 | 1.7309 | *PTPN22* |
| 3 | 29550001 | 29600000 | 1.7309 | *RSBN1* |
| 16 | 80600001 | 80650000 | 1.7308 | *IPO9* |
| 16 | 80600001 | 80650000 | 1.7308 | *NAV1* |
| 26 | 13350001 | 13400000 | 1.7303 | *TNKS2* |
| 14 | 43200001 | 43250000 | 1.7298 | *MRPS28* |

| | | | | |
|---|---|---|---|---|
| 14 | 43200001 | 43250000 | 1.7298 | *TPD52* |
| 3 | 25250001 | 25300000 | 1.7284 | *WDR3* |
| 3 | 25250001 | 25300000 | 1.7284 | *SPAG17* |
| 3 | 25250001 | 25300000 | 1.7284 | *GDAP2* |
| 2 | 104000001 | 104050000 | 1.7271 | *MREG* |
| 4 | 11950001 | 12000000 | 1.7267 | *SGCE* |
| 8 | 41100001 | 41150000 | 1.7258 | *RFX3* |
| 2 | 39050001 | 39100000 | 1.7256 | *GALNT5* |
| 21 | 45050001 | 45100000 | 1.7255 | *CFL2* |
| 7 | 38700001 | 38750000 | 1.7254 | *NSD1* |
| 5 | 5850001 | 5900000 | 1.7247 | *OSBPL8* |
| 2 | 31300001 | 31350000 | 1.7242 | *SLC38A11* |
| 20 | 14650001 | 14700000 | 1.724 | *CWC27* |
| 5 | 107050001 | 107100000 | 1.7239 | *IQSEC3* |
| 3 | 78700001 | 78750000 | 1.7239 | *SGIP1* |
| 22 | 60250001 | 60300000 | 1.7234 | *PLXNA1* |
| 22 | 60250001 | 60300000 | 1.7234 | *CHCHD6* |
| 29 | 46600001 | 46650000 | 1.7228 | *SNORD14* |
| 25 | 900001 | 950000 | 1.7224 | *CACNA1H* |
| 13 | 63400001 | 63450000 | 1.7211 | *RALY* |
| 27 | 37950001 | 38000000 | 1.7211 | *CSGALNACT1* |
| 3 | 25850001 | 25900000 | 1.7211 | *MAN1A2* |
| 3 | 28000001 | 28050000 | 1.7197 | *NGF* |
| 7 | 6900001 | 6950000 | 1.7195 | *TPM4* |
| 7 | 6900001 | 6950000 | 1.7195 | *OR1AB2* |
| 26 | 22150001 | 22200000 | 1.7191 | *BTRC* |
| 2 | 1200001 | 1250000 | 1.7179 | *TUBGCP5* |
| 2 | 41800001 | 41850000 | 1.7177 | *GALNT13* |
| 26 | 8250001 | 8300000 | 1.7162 | *PRKG1* |
| 6 | 86450001 | 86500000 | 1.7161 | *SLC4A4* |
| 7 | 38500001 | 38550000 | 1.7155 | *ZNF346* |
| 7 | 38500001 | 38550000 | 1.7155 | *U6* |
| 25 | 21600001 | 21650000 | 1.7144 | *PRKCB* |
| 3 | 23700001 | 23750000 | 1.7144 | *HAO2* |
| 3 | 23700001 | 23750000 | 1.7144 | *HSD3B1* |
| 16 | 80650001 | 80700000 | 1.7139 | *NAV1* |
| 10 | 21500001 | 21550000 | 1.7136 | *MYH6* |
| 10 | 21500001 | 21550000 | 1.7136 | *MYH7* |
| 10 | 21500001 | 21550000 | 1.7136 | *CMTM5* |
| 10 | 21500001 | 21550000 | 1.7136 | *IL25* |
| 10 | 21500001 | 21550000 | 1.7136 | *MIR208B* |
| 10 | 21500001 | 21550000 | 1.7136 | *MIR208A* |
| 22 | 49000001 | 49050000 | 1.7126 | *IQCF2* |
| 22 | 49000001 | 49050000 | 1.7126 | *IQCF1* |
| 22 | 49000001 | 49050000 | 1.7126 | *IQCF5* |
| 27 | 37800001 | 37850000 | 1.7121 | *INTS10* |
| 18 | 11400001 | 11450000 | 1.712 | *GSE1* |
| 19 | 21600001 | 21650000 | 1.712 | *TRARG1* |
| 19 | 21600001 | 21650000 | 1.712 | *BHLHA9* |
| 24 | 24100001 | 24150000 | 1.7109 | *CCDC178* |
| 28 | 28550001 | 28600000 | 1.7109 | *MICU1* |
| 10 | 70900001 | 70950000 | 1.7096 | *DACT1* |

| | | | | |
|---|---|---|---|---|
| 18 | 14300001 | 14350000 | 1.7091 | *ANKRD11* |
| 9 | 80900001 | 80950000 | 1.7088 | *PEX3* |
| 9 | 80900001 | 80950000 | 1.7088 | *ADAT2* |
| 4 | 68500001 | 68550000 | 1.7084 | *HIBADH* |
| 26 | 21950001 | 22000000 | 1.7084 | *TLX1* |
| 11 | 97950001 | 98000000 | 1.7082 | *RALGPS1* |
| 23 | 5450001 | 5500000 | 1.708 | *FAM83B* |
| 8 | 88900001 | 88950000 | 1.708 | *SHC3* |
| 6 | 91050001 | 91100000 | 1.7077 | *SCARB2* |
| 20 | 13400001 | 13450000 | 1.7077 | *SREK1* |
| 14 | 25950001 | 26000000 | 1.7074 | *CA8* |
| 5 | 41450001 | 41500000 | 1.7067 | *ABCD2* |
| 6 | 30800001 | 30850000 | 1.7063 | *GRID2* |
| 5 | 105400001 | 105450000 | 1.7054 | *GALNT8* |
| 12 | 17800001 | 17850000 | 1.7053 | *SUCLA2* |
| 2 | 90200001 | 90250000 | 1.7053 | *MPP4* |
| 2 | 90200001 | 90250000 | 1.7053 | *ALS2* |
| 5 | 17800001 | 17850000 | 1.7047 | *C5H12orf50* |
| 5 | 17800001 | 17850000 | 1.7047 | *CEP290* |
| 5 | 17800001 | 17850000 | 1.7047 | *C5H12orf29* |
| 19 | 9350001 | 9400000 | 1.7036 | *RNF43* |
| 19 | 9350001 | 9400000 | 1.7036 | *HSF5* |
| 1 | 2900001 | 2950000 | 1.7031 | *SYNJ1* |
| 1 | 2900001 | 2950000 | 1.7031 | *CFAP298* |
| 8 | 79000001 | 79050000 | 1.7025 | *AGTPBP1* |
| 2 | 90100001 | 90150000 | 1.7012 | *C2CD6* |
| 2 | 4850001 | 4900000 | 1.7012 | *LIMS2* |
| 2 | 4850001 | 4900000 | 1.7012 | *MYO7B* |
| 2 | 4850001 | 4900000 | 1.7012 | *GPR17* |
| 2 | 18000001 | 18050000 | 1.7001 | *CCDC141* |
| 2 | 18000001 | 18050000 | 1.7001 | *U6* |
| 13 | 2500001 | 2550000 | 1.6995 | *LAMP5* |
| 13 | 2500001 | 2550000 | 1.6995 | *PLCB4* |
| 3 | 46150001 | 46200000 | 1.699 | *DPYD* |
| 26 | 10600001 | 10650000 | 1.6981 | *ACTA2* |
| 26 | 10600001 | 10650000 | 1.6981 | *STAMBPL1* |
| 7 | 19300001 | 19350000 | 1.6977 | *PLIN3* |
| 7 | 19300001 | 19350000 | 1.6977 | *TICAM1* |
| 2 | 26750001 | 26800000 | 1.6975 | *BBS5* |
| 2 | 26750001 | 26800000 | 1.6975 | *KLHL41* |
| 7 | 11700001 | 11750000 | 1.6961 | *RFX1* |
| 7 | 11700001 | 11750000 | 1.6961 | *DCAF15* |
| 18 | 25150001 | 25200000 | 1.6958 | *PSME3IP1* |
| 18 | 25150001 | 25200000 | 1.6958 | *CPNE2* |
| 20 | 600001 | 650000 | 1.6955 | *SLIT3* |
| 17 | 55550001 | 55600000 | 1.6955 | *CIT* |
| 9 | 34450001 | 34500000 | 1.694 | *NT5DC1* |
| 9 | 34450001 | 34500000 | 1.694 | *TSPYL4* |
| 1 | 56400001 | 56450000 | 1.6932 | *PLCXD2* |
| 1 | 56400001 | 56450000 | 1.6932 | *CD96* |
| 27 | 40250001 | 40300000 | 1.692 | *RARB* |
| 22 | 4400001 | 4450000 | 1.6914 | *RBMS3* |

| | | | | |
|---|---|---|---|---|
| 21 | 49300001 | 49350000 | 1.6902 | *FBXO33* |
| 1 | 43150001 | 43200000 | 1.6894 | *DCBLD2* |
| 7 | 48500001 | 48550000 | 1.6885 | *SPOCK1* |
| 2 | 41900001 | 41950000 | 1.6882 | *GALNT13* |
| 7 | 106450001 | 106500000 | 1.688 | *EFNA5* |
| 3 | 25800001 | 25850000 | 1.6875 | *MAN1A2* |
| 10 | 7800001 | 7850000 | 1.6874 | *IQGAP2* |
| 4 | 68450001 | 68500000 | 1.6874 | *HIBADH* |
| 10 | 7800001 | 7850000 | 1.6874 | *F2RL2* |
| 9 | 86200001 | 86250000 | 1.6872 | *UST* |
| 8 | 75150001 | 75200000 | 1.6866 | *AQP3* |
| 8 | 75150001 | 75200000 | 1.6866 | *AQP7* |
| 2 | 42000001 | 42050000 | 1.6859 | *GALNT13* |
| 18 | 39150001 | 39200000 | 1.6856 | *PKD1L3* |
| 18 | 39150001 | 39200000 | 1.6856 | *IST1* |
| 3 | 10900001 | 10950000 | 1.6853 | *OR6K5* |
| 3 | 10900001 | 10950000 | 1.6853 | *OR6K6* |
| 3 | 10900001 | 10950000 | 1.6853 | *OR6K4* |
| 3 | 10900001 | 10950000 | 1.6853 | *OR6N1* |
| 22 | 37100001 | 37150000 | 1.685 | *PRICKLE2* |
| 20 | 14950001 | 15000000 | 1.6849 | *RGS7BP* |
| 4 | 8550001 | 8600000 | 1.6844 | *CDK14* |
| 2 | 36250001 | 36300000 | 1.6839 | *PLA2R1* |
| 24 | 50001 | 100000 | 1.6833 | *OR5W32P* |
| 6 | 35650001 | 35700000 | 1.683 | *TIGD2* |
| 24 | 45600001 | 45650000 | 1.6827 | *EPG5* |
| 1 | 2300001 | 2350000 | 1.6821 | *IFNAR2* |
| 1 | 2300001 | 2350000 | 1.6821 | *IL10RB* |
| 14 | 24700001 | 24750000 | 1.6815 | *SDCBP* |
| 3 | 29500001 | 29550000 | 1.6814 | *PTPN22* |
| 3 | 29500001 | 29550000 | 1.6814 | *BCL2L15* |
| 16 | 65050001 | 65100000 | 1.6811 | *COLGALT2* |
| 19 | 52500001 | 52550000 | 1.6806 | *CCDC40* |
| 19 | 52500001 | 52550000 | 1.6806 | *TBC1D16* |
| 9 | 15500001 | 15550000 | 1.6791 | *MYO6* |
| 20 | 3100001 | 3150000 | 1.6786 | *RANBP17* |
| 13 | 54250001 | 54300000 | 1.678 | *COL20A1* |
| 13 | 54250001 | 54300000 | 1.678 | *ARFGAP1* |
| 13 | 54250001 | 54300000 | 1.678 | *CHRNA4* |
| 1 | 7400001 | 7450000 | 1.678 | *U6* |
| 1 | 38200001 | 38250000 | 1.6774 | *PROS1* |
| 5 | 107100001 | 107150000 | 1.6771 | *IQSEC3* |
| 19 | 12250001 | 12300000 | 1.6771 | *BCAS3* |
| 5 | 107100001 | 107150000 | 1.6771 | *SLC6A12* |
| 19 | 12250001 | 12300000 | 1.6771 | *PPM1D* |
| 19 | 12250001 | 12300000 | 1.6771 | *U6* |
| 17 | 19400001 | 19450000 | 1.677 | *SLC7A11* |
| 4 | 49300001 | 49350000 | 1.6766 | *NRCAM* |
| 8 | 78300001 | 78350000 | 1.6762 | *NTRK2* |
| 24 | 37400001 | 37450000 | 1.6752 | *MYL12B* |
| 24 | 37400001 | 37450000 | 1.6752 | *MYL12A* |
| 12 | 21400001 | 21450000 | 1.6749 | *ALG11* |

| | | | | |
|---|---|---|---|---|
| 12 | 21400001 | 21450000 | 1.6749 | *NEK5* |
| 26 | 13850001 | 13900000 | 1.6726 | *IDE* |
| 29 | 6150001 | 6200000 | 1.6718 | *NOX4* |
| 25 | 950001 | 1000000 | 1.6717 | *CACNA1H* |
| 25 | 950001 | 1000000 | 1.6717 | *TPSB2* |
| 3 | 57400001 | 57450000 | 1.6712 | *CLCA3* |
| 3 | 55700001 | 55750000 | 1.6712 | *U6* |
| 29 | 50450001 | 50500000 | 1.671 | *AP2A2* |
| 29 | 50450001 | 50500000 | 1.671 | *CHID1* |
| 8 | 39300001 | 39350000 | 1.6701 | *PLGRKT* |
| 20 | 18350001 | 18400000 | 1.6699 | *ERCC8* |
| 20 | 18350001 | 18400000 | 1.6699 | *NDUFAF2* |
| 7 | 83450001 | 83500000 | 1.6694 | *VCAN* |
| 3 | 43350001 | 43400000 | 1.6681 | *AGL* |
| 7 | 42150001 | 42200000 | 1.6675 | *OR2G6* |
| 12 | 13550001 | 13600000 | 1.6665 | *ENOX1* |
| 11 | 102500001 | 102550000 | 1.6661 | *CFAP77* |
| 19 | 31150001 | 31200000 | 1.6657 | *MYOCD* |
| 26 | 11400001 | 11450000 | 1.6656 | *KIF20B* |
| 3 | 116550001 | 116600000 | 1.6656 | *COPS8* |
| 7 | 13800001 | 13850000 | 1.6654 | *OR7E200* |
| 7 | 13800001 | 13850000 | 1.6654 | *OR7E202* |
| 14 | 1100001 | 1150000 | 1.6646 | *EEF1D* |
| 14 | 1100001 | 1150000 | 1.6646 | *PYCR3* |
| 14 | 1100001 | 1150000 | 1.6646 | *GSDMD* |
| 14 | 1100001 | 1150000 | 1.6646 | *MROH6* |
| 14 | 1100001 | 1150000 | 1.6646 | *NAPRT* |
| 14 | 1100001 | 1150000 | 1.6646 | *TIGD5* |
| 18 | 46550001 | 46600000 | 1.6645 | *TYROBP* |
| 18 | 46550001 | 46600000 | 1.6645 | *APLP1* |
| 18 | 46550001 | 46600000 | 1.6645 | *KIRREL2* |
| 18 | 46550001 | 46600000 | 1.6645 | *NFKBID* |
| 18 | 46550001 | 46600000 | 1.6645 | *NPHS1* |
| 18 | 46550001 | 46600000 | 1.6645 | *HCST* |
| 1 | 83450001 | 83500000 | 1.6643 | *KLHL24* |
| 13 | 63450001 | 63500000 | 1.6643 | *EIF2S2* |
| 13 | 63450001 | 63500000 | 1.6643 | *RALY* |
| 2 | 62000001 | 62050000 | 1.6629 | *ZRANB3* |
| 16 | 66950001 | 67000000 | 1.6624 | *HMCN1* |
| 2 | 17650001 | 17700000 | 1.661 | *SESTD1* |
| 8 | 106150001 | 106200000 | 1.6603 | *ASTN2* |
| 22 | 49600001 | 49650000 | 1.6585 | *DOCK3* |
| 26 | 35350001 | 35400000 | 1.6582 | *TRUB1* |
| 4 | 13600001 | 13650000 | 1.6579 | *SLC25A13* |
| 2 | 36200001 | 36250000 | 1.6579 | *ITGB6* |
| 18 | 53200001 | 53250000 | 1.6576 | *EML2* |
| 25 | 3150001 | 3200000 | 1.6576 | *CREBBP* |
| 10 | 65000001 | 65050000 | 1.6576 | *C10H15orf48* |
| 10 | 65000001 | 65050000 | 1.6576 | *SPATA5L1* |
| 18 | 53200001 | 53250000 | 1.6576 | *GIPR* |
| 20 | 3800001 | 3850000 | 1.6569 | *FBXW11* |
| 1 | 148700001 | 148750000 | 1.6568 | *CLDN14* |

| | | | | |
|---|---|---|---|---|
| 4 | 64000001 | 64050000 | 1.6561 | *AVL9* |
| 4 | 64000001 | 64050000 | 1.6561 | *LSM5* |
| 3 | 66050001 | 66100000 | 1.656 | *IFI44L* |
| 2 | 36500001 | 36550000 | 1.6558 | *LY75* |
| 6 | 35700001 | 35750000 | 1.6554 | *FAM13A* |
| 19 | 39950001 | 40000000 | 1.6551 | *NEUROD2* |
| 21 | 51500001 | 51550000 | 1.6548 | *LRFN5* |
| 19 | 12100001 | 12150000 | 1.6536 | *BCAS3* |
| 22 | 49500001 | 49550000 | 1.6535 | *DOCK3* |
| 28 | 35400001 | 35450000 | 1.6533 | *CGN1* |
| 8 | 75350001 | 75400000 | 1.6526 | *UBAP2* |
| 20 | 3750001 | 3800000 | 1.6526 | *FBXW11* |
| 8 | 75350001 | 75400000 | 1.6526 | *SNORD121A* |
| 5 | 43700001 | 43750000 | 1.6525 | *RAB3IP* |
| 25 | 18200001 | 18250000 | 1.6523 | *ACSM1* |
| 25 | 18200001 | 18250000 | 1.6523 | *ACSM2B* |
| 1 | 52750001 | 52800000 | 1.6521 | *CD47* |
| 18 | 11500001 | 11550000 | 1.6517 | *GSE1* |
| 3 | 120400001 | 120450000 | 1.6516 | *HDLBP* |
| 19 | 10850001 | 10900000 | 1.6505 | *RPS6KB1* |
| 19 | 10850001 | 10900000 | 1.6505 | *RNFT1* |
| 10 | 34150001 | 34200000 | 1.6491 | *RASGRP1* |
| 7 | 19100001 | 19150000 | 1.6487 | *KDM4B* |
| 6 | 44700001 | 44750000 | 1.6487 | *SEPSECS* |
| 18 | 2700001 | 2750000 | 1.647 | *CFDP1* |
| 18 | 2700001 | 2750000 | 1.647 | *CFDP2* |
| 21 | 8700001 | 8750000 | 1.6469 | *ARRDC4* |
| 5 | 67550001 | 67600000 | 1.6449 | *TTC41* |
| 2 | 30250001 | 30300000 | 1.6443 | *SCN1A* |
| 1 | 144500001 | 144550000 | 1.6442 | *TSPEAR* |
| 22 | 27250001 | 27300000 | 1.6437 | *CNTN3* |
| 18 | 43350001 | 43400000 | 1.6435 | *CEP89* |
| 18 | 43350001 | 43400000 | 1.6435 | *FAAP24* |
| 1 | 56350001 | 56400000 | 1.6433 | *CD96* |
| 20 | 14450001 | 14500000 | 1.6433 | *CWC27* |
| 20 | 31950001 | 32000000 | 1.6429 | *GHR* |
| 14 | 58100001 | 58150000 | 1.6428 | *OXR1* |
| 17 | 55700001 | 55750000 | 1.6425 | *CIT* |
| 6 | 60150001 | 60200000 | 1.6418 | *UCHL1* |
| 4 | 72100001 | 72150000 | 1.6401 | *ADAM22* |
| 1 | 60750001 | 60800000 | 1.6398 | *LSAMP* |
| 2 | 110850001 | 110900000 | 1.6398 | *MOGAT1* |
| 8 | 29100001 | 29150000 | 1.6398 | *TTC39B* |
| 21 | 51600001 | 51650000 | 1.6368 | *LRFN5* |
| 21 | 51600001 | 51650000 | 1.6368 | *U4* |
| 3 | 40300001 | 40350000 | 1.6352 | *COL11A1* |
| 11 | 99050001 | 99100000 | 1.6351 | *GLE1* |
| 11 | 99050001 | 99100000 | 1.6351 | *ODF2* |
| 10 | 69600001 | 69650000 | 1.6349 | *EXOC5* |
| 6 | 65950001 | 66000000 | 1.6346 | *GABRB1* |
| 6 | 65950001 | 66000000 | 1.6346 | *U6* |
| 25 | 18850001 | 18900000 | 1.6341 | *DNAH3* |

| | | | | |
|---|---|---|---|---|
| 2 | 23350001 | 23400000 | 1.6332 | *CDCA7* |
| 4 | 10050001 | 10100000 | 1.6329 | *CDK6* |
| 18 | 55800001 | 55850000 | 1.6323 | *TEAD2* |
| 18 | 55800001 | 55850000 | 1.6323 | *SLC6A16* |
| 18 | 55800001 | 55850000 | 1.6323 | *CD37* |
| 18 | 55800001 | 55850000 | 1.6323 | *DKKL1* |
| 2 | 44250001 | 44300000 | 1.6319 | *CACNB4* |
| 16 | 67000001 | 67050000 | 1.6319 | *HMCN1* |
| 18 | 46600001 | 46650000 | 1.6318 | *LRFN3* |
| 18 | 46600001 | 46650000 | 1.6318 | *TYROBP* |
| 7 | 54250001 | 54300000 | 1.6316 | *ARHGAP26* |
| 20 | 3650001 | 3700000 | 1.6307 | *FBXW11* |
| 20 | 32550001 | 32600000 | 1.6307 | *FBXO4* |
| 8 | 89650001 | 89700000 | 1.6304 | *SPIN1* |
| 23 | 9250001 | 9300000 | 1.6304 | *DEF6* |
| 23 | 9250001 | 9300000 | 1.6304 | *ZNF76* |
| 16 | 45400001 | 45450000 | 1.6281 | *PARK7* |
| 16 | 45400001 | 45450000 | 1.6281 | *TNFRSF9* |
| 11 | 80800001 | 80850000 | 1.6279 | *GEN1* |
| 10 | 92750001 | 92800000 | 1.6269 | *SEL1L* |
| 20 | 15250001 | 15300000 | 1.6261 | *RNF180* |
| 21 | 45850001 | 45900000 | 1.6258 | *RALGAPA1* |
| 8 | 78900001 | 78950000 | 1.6248 | *AGTPBP1* |
| 23 | 45050001 | 45100000 | 1.6243 | *NEDD9* |
| 13 | 65450001 | 65500000 | 1.624 | *DLGAP4* |
| 8 | 77150001 | 77200000 | 1.6238 | *KIF27* |
| 8 | 77150001 | 77200000 | 1.6238 | *C8H9orf64* |
| 4 | 10550001 | 10600000 | 1.6234 | *HEPACAM2* |
| 16 | 32400001 | 32450000 | 1.6233 | *HNRNPU* |
| 16 | 32400001 | 32450000 | 1.6233 | *COX20* |
| 4 | 69750001 | 69800000 | 1.623 | *NFE2L3* |
| 27 | 8050001 | 8100000 | 1.6227 | *VEGFC* |
| 9 | 71500001 | 71550000 | 1.6225 | *EYA4* |
| 21 | 65900001 | 65950000 | 1.6225 | *MIR382* |
| 21 | 65900001 | 65950000 | 1.6225 | *MIR487B* |
| 21 | 65900001 | 65950000 | 1.6225 | *MIR487A* |
| 21 | 65900001 | 65950000 | 1.6225 | *MIR154* |
| 21 | 65900001 | 65950000 | 1.6225 | *MIR494* |
| 15 | 20900001 | 20950000 | 1.6219 | *U6* |
| 2 | 36900001 | 36950000 | 1.6211 | *BAZ2B* |
| 26 | 9350001 | 9400000 | 1.6208 | *ATAD1* |
| 26 | 9350001 | 9400000 | 1.6208 | *PAPSS2* |
| 7 | 21800001 | 21850000 | 1.6207 | *RAD50* |
| 10 | 35650001 | 35700000 | 1.6197 | *EIF2AK4* |
| 10 | 35650001 | 35700000 | 1.6197 | *SRP14* |
| 29 | 18050001 | 18100000 | 1.6192 | *INTS4* |
| 12 | 15500001 | 15550000 | 1.619 | *SLC25A30* |
| 7 | 42000001 | 42050000 | 1.6186 | *OR2T4* |
| 29 | 18000001 | 18050000 | 1.6185 | *INTS4* |
| 29 | 18000001 | 18050000 | 1.6185 | *KCTD14* |
