# Supplementary material for: Identification of genomic diversity and selection signatures in Luxi cattle using whole-genome sequencing data
Source: Anim Biosci. 2024 Jan 20;37(3):461–70. doi: 10.5713/ab.23.0304 (PMC10915192; doi:10.5713/ab.23.0304)
Supplement: Supplementary file 8 [file ab-23-0304-Supplementary-Table-S8.pdf]

Supplementary Table S8. A summary of genes overlapped by *pi* , *F* st and XP-EHH methods in common regions.

Chromosome	Position start site(bp)	Position end site (bp)	Gene
1	87250001	87275000	*PEX5L*
2	22797327	22850000	*SP3*
5	58854800	58855759	*OR6C1N*
7	12550001	12575856	*NFIX*
7	43404172	43407636	*PRTN3*
7	43411277	43414454	*ELANE*
7	43416200	43418803	*CFD*
7	43418785	43436614	*MED16*
7	43436675	43436781	*U6*
7	43439368	43449545	*R3HDM4*
7	43656058	43670433	*STK11*
7	43673071	43679019	*CBARP*
7	43683704	43685876	*ATP5F1D*
7	43688529	43698292	*MIDN*
7	43707339	43712131	*CIRBP*
7	43713177	43716027	*FAM174C*
7	43722703	43725000	*EFNA2*
7	43800001	43804425	*PWWP3A*
7	43805788	43814284	*NDUFS7*
7	43814700	43817778	*GAMT*
7	43824822	43843948	*DAZAP1*
7	43846494	43848179	*RPS15*
7	43947454	43950000	*MEX3D*
7	43976251	43979203	*UQCR11*
7	43981884	44015020	*TCF3*
7	44175001	44182700	*KLF16*
7	44190928	44199228	*ABHD17A*
8	39351712	39362934	*INSL6*
8	39381277	39400000	*JAK2*
8	79000001	79025000	*AGTPBP1*
8	79250001	79255810	*NAA35*
8	79263894	79275000	*GOLM1*
9	42650001	42675000	*PDSS2*
14	14950001	14975000	*NSMCE2*
14	23330541	23375000	*PLAG1*
15	40374952	40375000	*MICAL2*
16	43425001	43450000	*UBE4B*
16	43747735	43750000	*CLSTN1*
19	11075001	11151673	*MED13*
19	11169421	11175000	*INTS2*
19	11300001	11350000	*BRIP1*
20	3700001	3725000	*FBXW11*
21	1252109	1253718	*NDN*
21	45875001	45900000	*RALGAPA1*
22	49600001	49650000	*DOCK3*
