# Supplementary material for: Identification of genomic diversity and selection signatures in Luxi cattle using whole-genome sequencing data
Source: Anim Biosci. 2024 Jan 20;37(3):461–70. doi: 10.5713/ab.23.0304 (PMC10915192; doi:10.5713/ab.23.0304)
Supplement: Supplementary file 9 [file ab-23-0304-Supplementary-Table-S9.pdf]

Supplementary Table S9. GO and KEGG enrichment analysis of LUX candidate genes by three methods.

KEGG PATHWAY Term	ID	Input number	Background number	*p* -Value	Corrected *p* -Value	Input
Oxidative phosphorylation	bta00190	3	140	0.001093807	0.038058112	ATP5F1D|UQCR11|NDUFS7
Parkinson disease	bta05012	3	150	0.001328493	0.038058112	ATP5F1D|UQCR11|NDUFS7
Alzheimer disease	bta05010	3	180	0.00221467	0.04119286	ATP5F1D|UQCR11|NDUFS7
Huntington disease	bta05016	3	198	0.002887695	0.051153452	ATP5F1D|UQCR11|NDUFS7
Thermogenesis	bta04714	3	239	0.004854508	0.06609911	ATP5F1D|UQCR11|NDUFS7
Adipocytokine signaling pathway	bta04920	2	72	0.004947426	0.06609911	STK11|JAK2
Thyroid hormone signaling pathway	bta04919	2	118	0.012553528	0.06609911	MED13|MED16
PI3K-Akt signaling pathway	bta04151	3	373	0.016056141	0.067007911	STK11|JAK2|EFNA2
Signaling pathways regulating pluripotency of stem cells	bta04550	2	139	0.017032924	0.067007911	JAK2|TCF3
Ubiquitin mediated proteolysis	bta04120	2	140	0.017260938	0.067007911	UBE4B|FBXW11
Non-alcoholic fatty liver disease (NAFLD)	bta04932	2	158	0.021584804	0.068890771	UQCR11|NDUFS7
Transcriptional misregulation in cancer	bta05202	2	188	0.029670777	0.077185517	ELANE|TCF3
Terpenoid backbone biosynthesis	bta00900	1	21	0.030684096	0.077649549	PDSS2
Circadian rhythm	bta04710	1	31	0.044325898	0.09521648	FBXW11
Homologous recombination	bta03440	1	42	0.059115563	0.112774306	BRIP1
Glycine, serine and threonine metabolism	bta00260	1	43	0.060448952	0.113000051	GAMT
Hedgehog signaling pathway	bta04340	1	47	0.065764095	0.116496396	FBXW11
Arginine and proline metabolism	bta00330	1	48	0.06708829	0.117720961	GAMT
Fanconi anemia pathway	bta03460	1	52	0.072366783	0.122365652	BRIP1
Staphylococcus aureus infection	bta05150	1	75	0.102157387	0.151623811	CFD
Leishmaniasis	bta05140	1	76	0.103431231	0.151623811	JAK2
EGFR tyrosine kinase inhibitor resistance	bta01521	1	80	0.108508998	0.153480408	JAK2
Prolactin signaling pathway	bta04917	1	83	0.1122989	0.156461389	JAK2
Peroxisome	bta04146	1	84	0.113558705	0.157626262	PEX5L
Complement and coagulation cascades	bta04610	1	87	0.117327661	0.161651444	CFD
Longevity regulating pathway	bta04211	1	90	0.121080982	0.164282485	STK11
Cardiac muscle contraction	bta04260	1	90	0.121080982	0.164282485	UQCR11
PD-L1 expression and PD-1 checkpoint pathway in cancer	bta05235	1	93	0.12481873	0.167023624	JAK2
mRNA surveillance pathway	bta03015	1	95	0.127301942	0.169735922	DAZAP1
Th1 and Th2 cell differentiation	bta04658	1	98	0.131013876	0.17221612	JAK2
AGE-RAGE signaling pathway in diabetic complications	bta04933	1	103	0.137166231	0.177790376	JAK2
Th17 cell differentiation	bta04659	1	113	0.149343718	0.187322875	JAK2
Toxoplasmosis	bta05145	1	113	0.149343718	0.187322875	JAK2

Cholinergic synapse	bta04725	1	114	0.150552204	0.187937651	JAK2
Metabolic pathways	bta01100	4	1468	0.153430999	0.189622364	ATP5F1D|UQCR11|GAMT|NDUFS7
Oocyte meiosis	bta04114	1	119	0.156569554	0.192224007	FBXW11
AMPK signaling pathway	bta04152	1	123	0.161353474	0.197445698	STK11
FoxO signaling pathway	bta04068	1	132	0.172020706	0.207765269	STK11
Autophagy - animal	bta04140	1	133	0.173197751	0.208509914	STK11
Hippo signaling pathway	bta04390	1	151	0.194107527	0.229231746	FBXW11
Retrograde endocannabinoid signaling	bta04723	1	152	0.195253928	0.22985589	NDUFS7
mTOR signaling pathway	bta04150	1	154	0.197541961	0.231815803	STK11
Ribosome	bta03010	1	160	0.204368045	0.236102213	RPS15
Wnt signaling pathway	bta04310	1	161	0.205500204	0.236675158	FBXW11
Protein processing in endoplasmic reticulum	bta04141	1	165	0.210013145	0.241126204	UBE4B
Cellular senescence	bta04218	1	166	0.211137467	0.241671193	FBXW11
Hepatitis B	bta05161	1	172	0.217850687	0.246323573	JAK2
Necroptosis	bta04217	1	177	0.223402432	0.251557334	JAK2
Influenza A	bta05164	1	178	0.224508159	0.251557334	JAK2
Tight junction	bta04530	1	178	0.224508159	0.251557334	STK11
Axon guidance	bta04360	1	180	0.226715006	0.253267214	EFNA2
Systemic lupus erythematosus	bta05322	1	182	0.228915725	0.254960029	ELANE
Chemokine signaling pathway	bta04062	1	189	0.236570231	0.262340208	JAK2
Tuberculosis	bta05152	1	198	0.24630288	0.269484327	JAK2
Jak-STAT signaling pathway	bta04630	1	202	0.250589546	0.272571085	JAK2
Kaposi sarcoma-associated herpesvirus infection	bta05167	1	206	0.254852422	0.276399712	JAK2
Rap1 signaling pathway	bta04015	1	216	0.26540638	0.284527877	EFNA2
Human T-cell leukemia virus 1 infection	bta05166	1	234	0.284037193	0.30189096	TCF3
Human immunodeficiency virus 1 infection	bta05170	1	234	0.284037193	0.30189096	FBXW11
Ras signaling pathway	bta04014	1	242	0.292168822	0.308769323	EFNA2
MAPK signaling pathway	bta04010	1	292	0.340987729	0.355314945	EFNA2
Herpes simplex virus 1 infection	bta05168	1	405	0.439530742	0.452923646	JAK2
Pathways in cancer	bta05200	1	539	0.537857413	0.543703689	JAK2

**Gene Ontology Term**	**ID**	**Input number**	**Background number**	***p* -Value**	**Corrected *p* -Value**	**Input**
nucleus	GO:0005634	13	2955	0.000148246	0.032311927	MIDN|UBE4B|GAMT|KLF16|TCF3|R3HDM4|MICAL2|NSMCE2|BRIP1|MEX3D|NFIX|PLAG1|RALGAPA1

thyroid hormone receptor binding	GO:0046966	2	14	0.000231509	0.032311927	MED13|MED16
positive regulation of mRNA splicing, via spliceosome	GO:0048026	2	16	0.000294645	0.032311927	CIRBP|DAZAP1
aerobic respiration	GO:0009060	2	20	0.000443263	0.032311927	ATP5F1D|NDUFS7
poly(U) RNA binding	GO:0008266	2	21	0.000485044	0.032311927	CIRBP|DAZAP1
cytosol	GO:0005829	11	2482	0.00052116	0.032311927	MIDN|DAZAP1|FBXW11|NDN|DOCK3|STK11|AGTPBP1|PDSS2|PEX5L|PLAG1|PRTN3
transcription initiation from RNA polymerase II promoter	GO:0006367	2	27	0.000774198	0.038058112	MED13|MED16
response to UV	GO:0009411	2	30	0.00094328	0.038058112	UBE4B|ELANE
RNA polymerase II cis-regulatory region sequence-specific DNA binding	GO:0000978	5	581	0.001366401	0.038058112	SP3|PLAG1|KLF16|TCF3|NDN
positive regulation of transcription by RNA polymerase II	GO:0045944	6	872	0.001383605	0.038058112	KLF16|TCF3|NDN|MED13|NFIX|PLAG1
serine-type endopeptidase activity	GO:0004252	3	154	0.001430552	0.038058112	ELANE|CFD|PRTN3
axon cytoplasm	GO:1904115	2	39	0.001546942	0.038058112	FBXW11|AGTPBP1
mediator complex	GO:0016592	2	40	0.001622836	0.038058112	MED13|MED16
transcription repressor complex	GO:0017053	2	41	0.001700477	0.038058112	ELANE|SP3
proteolysis	GO:0006508	4	368	0.001861104	0.038058112	ELANE|AGTPBP1|CFD|PRTN3
negative regulation of transcription by RNA polymerase II	GO:0000122	5	625	0.001877366	0.038058112	ELANE|SP3|KLF16|TCF3|NFIX
phagocytosis	GO:0006909	2	44	0.001943828	0.038058112	ELANE|PRTN3
DNA-binding transcription repressor activity, RNA polymerase II-specific	GO:0001227	3	218	0.003769241	0.063734443	SP3|KLF16|TCF3
transcription, DNA-templated	GO:0006351	2	70	0.004690191	0.06609911	PLAG1|TCF3
negative regulation of cell growth	GO:0030308	2	83	0.006475343	0.06609911	CIRBP|STK11
protein dephosphorylation	GO:0006470	2	94	0.008190368	0.06609911	STK11|FBXW11
granzyme-mediated apoptotic signaling pathway	GO:0008626	1	5	0.008461119	0.06609911	UBE4B
respirasome	GO:0070469	1	5	0.008461119	0.06609911	UQCR11
vesicle-mediated transport in synapse	GO:0099003	1	5	0.008461119	0.06609911	CLSTN1
positive regulation of maintenance of mitotic sister chromatid cohesion	GO:0034184	1	5	0.008461119	0.06609911	NSMCE2
mitochondrial proton-transporting ATP synthase complex assembly	GO:0033615	1	5	0.008461119	0.06609911	ATP5F1D
cell-cell junction maintenance	GO:0045217	1	5	0.008461119	0.06609911	PRTN3
small GTPase binding	GO:0031267	1	5	0.008461119	0.06609911	PEX5L
microtubule organizing center organization	GO:0031023	1	5	0.008461119	0.06609911	FBXW11
megakaryocyte differentiation	GO:0030219	1	5	0.008461119	0.06609911	SP3
N-terminal protein amino acid acetylation	GO:0006474	1	5	0.008461119	0.06609911	NAA35
Golgi localization	GO:0051645	1	5	0.008461119	0.06609911	STK11
N-terminal peptidyl-methionine acetylation	GO:0017196	1	5	0.008461119	0.06609911	NAA35
anoikis	GO:0043276	1	6	0.009864498	0.06609911	STK11
negative regulation of epithelial cell proliferation	GO:0060770	1	6	0.009864498	0.06609911	STK11

dendrite extension	GO:0097484	1	6	0.009864498	0.06609911	STK11
embryonic process involved in female pregnancy	GO:0060136	1	6	0.009864498	0.06609911	SP3
prostate gland growth	GO:0060736	1	6	0.009864498	0.06609911	PLAG1
NADH dehydrogenase activity	GO:0003954	1	6	0.009864498	0.06609911	NDUFS7
gland morphogenesis	GO:0022612	1	6	0.009864498	0.06609911	PLAG1
Smc5-Smc6 complex	GO:0030915	1	6	0.009864498	0.06609911	NSMCE2
positive regulation of glial cell proliferation	GO:0060252	1	6	0.009864498	0.06609911	PLAG1
macromolecule depalmitoylation	GO:0098734	1	6	0.009864498	0.06609911	ABHD17A
glial cell migration	GO:0008347	1	6	0.009864498	0.06609911	NDN
positive regulation of circadian rhythm	GO:0042753	1	7	0.01126594	0.06609911	FBXW11
protein depalmitoylation	GO:0002084	1	7	0.01126594	0.06609911	ABHD17A
S-adenosylmethionine-dependent methyltransferase activity	GO:0008757	1	7	0.01126594	0.06609911	GAMT
poly(G) binding	GO:0034046	1	7	0.01126594	0.06609911	DAZAP1
tyrosine phosphorylation of STAT protein	GO:0007260	1	7	0.01126594	0.06609911	JAK2
SUMO ligase activity	GO:0061665	1	7	0.01126594	0.06609911	NSMCE2
cellular response to UV-B	GO:0071493	1	8	0.012665446	0.06609911	STK11
cellular response to cold	GO:0070417	1	8	0.012665446	0.06609911	CIRBP
complement activation, alternative pathway	GO:0006957	1	8	0.012665446	0.06609911	CFD
vasculature development	GO:0001944	1	8	0.012665446	0.06609911	STK11
retrograde axonal transport	GO:0008090	1	8	0.012665446	0.06609911	FBXW11
leukocyte migration	GO:0050900	1	8	0.012665446	0.06609911	ELANE
positive regulation of immune response	GO:0050778	1	8	0.012665446	0.06609911	ELANE
ubiquitin-ubiquitin ligase activity	GO:0034450	1	8	0.012665446	0.06609911	UBE4B
proton transmembrane transporter activity	GO:0015078	1	8	0.012665446	0.06609911	ATP5F1D
positive regulation of mitotic metaphase/anaphase transition	GO:0045842	1	8	0.012665446	0.06609911	NSMCE2
isoprenoid biosynthetic process	GO:0008299	1	8	0.012665446	0.06609911	PDSS2
protein polyubiquitination	GO:0000209	2	119	0.012753176	0.06609911	UBE4B|FBXW11
positive regulation of ubiquitin-dependent protein catabolic process	GO:2000060	1	9	0.014063019	0.06609911	AGTPBP1
central nervous system neuron development	GO:0021954	1	9	0.014063019	0.06609911	AGTPBP1
granulocyte differentiation	GO:0030851	1	9	0.014063019	0.06609911	SP3
positive regulation of synaptic transmission	GO:0050806	1	9	0.014063019	0.06609911	CLSTN1
telomere maintenance via recombination	GO:0000722	1	9	0.014063019	0.06609911	NSMCE2
translation repressor activity	GO:0030371	1	9	0.014063019	0.06609911	CIRBP
actin filament depolymerization	GO:0030042	1	10	0.015458663	0.06609911	MICAL2
regulation of protein kinase B signaling	GO:0051896	1	10	0.015458663	0.06609911	STK11

maternal placenta development	GO:0001893	1	10	0.015458663	0.06609911	DAZAP1
positive regulation of proteolysis	GO:0045862	1	10	0.015458663	0.06609911	FBXW11
quinone binding	GO:0048038	1	10	0.015458663	0.06609911	NDUFS7
palmitoyl-(protein) hydrolase activity	GO:0008474	1	10	0.015458663	0.06609911	ABHD17A
mitochondrial ATP synthesis coupled proton transport	GO:0042776	1	10	0.015458663	0.06609911	ATP5F1D
vesicle transport along microtubule	GO:0047496	1	10	0.015458663	0.06609911	FBXW11
dopamine receptor signaling pathway	GO:0007212	1	10	0.015458663	0.06609911	KLF16
microtubule associated complex	GO:0005875	1	10	0.015458663	0.06609911	FBXW11
protein palmitoylation	GO:0018345	1	10	0.015458663	0.06609911	ABHD17A
neurotrophin TRK receptor signaling pathway	GO:0048011	1	10	0.015458663	0.06609911	NDN
centrosome	GO:0005813	3	375	0.016282836	0.067007911	FBXW11|PLAG1|NDN
positive thymic T cell selection	GO:0045059	1	11	0.016852379	0.067007911	STK11
RNA stem-loop binding	GO:0035613	1	11	0.016852379	0.067007911	DAZAP1
negative regulation of phagocytosis	GO:0050765	1	11	0.016852379	0.067007911	PRTN3
mitogen-activated protein kinase kinase kinase binding	GO:0031435	1	12	0.01824417	0.067007911	TCF3
monocyte differentiation	GO:0030224	1	12	0.01824417	0.067007911	SP3
nucleus organization	GO:0006997	1	12	0.01824417	0.067007911	GOLM1
negative regulation of TORC1 signaling	GO:1904262	1	12	0.01824417	0.067007911	STK11
oxidoreductase activity,	GO:0016709	1	12	0.01824417	0.067007911	MICAL2
cerebellar Purkinje cell differentiation	GO:0021702	1	12	0.01824417	0.067007911	AGTPBP1
enzyme binding	GO:0019899	2	147	0.018893401	0.067007911	UBE4B|PRTN3
natural killer cell differentiation	GO:0001779	1	13	0.019634039	0.067007911	SP3
embryonic placenta development	GO:0001892	1	13	0.019634039	0.067007911	SP3
seminiferous tubule development	GO:0072520	1	13	0.019634039	0.067007911	BRIP1
respiratory system process	GO:0003016	1	13	0.019634039	0.067007911	NDN
ubiquinone biosynthetic process	GO:0006744	1	13	0.019634039	0.067007911	PDSS2
neuromuscular process	GO:0050905	1	13	0.019634039	0.067007911	AGTPBP1
ventricular trabecula myocardium morphogenesis	GO:0003222	1	13	0.019634039	0.067007911	UBE4B
definitive hemopoiesis	GO:0060216	1	13	0.019634039	0.067007911	SP3
ribosomal small subunit assembly	GO:0000028	1	14	0.021021988	0.06800156	RPS15
bone remodeling	GO:0046849	1	14	0.021021988	0.06800156	EFNA2
trophectodermal cell differentiation	GO:0001829	1	14	0.021021988	0.06800156	SP3
axonal fasciculation	GO:0007413	1	14	0.021021988	0.06800156	NDN
nuclear migration	GO:0007097	1	14	0.021021988	0.06800156	FBXW11
stress granule assembly	GO:0034063	1	14	0.021021988	0.06800156	CIRBP

embryonic camera-type eye morphogenesis	GO:0048596	1	15	0.02240802	0.068890771	SP3
dynein complex binding	GO:0070840	1	15	0.02240802	0.068890771	FBXW11
regulation of dendrite morphogenesis	GO:0048814	1	15	0.02240802	0.068890771	STK11
intrinsic apoptotic signaling pathway by p53 class mediator	GO:0072332	1	15	0.02240802	0.068890771	STK11
tissue homeostasis	GO:0001894	1	15	0.02240802	0.068890771	STK11
transcription coactivator activity	GO:0003713	2	163	0.022857934	0.069697963	MED13|TCF3
olfactory bulb development	GO:0021772	1	16	0.023792137	0.070243452	AGTPBP1
microtubule plus-end binding	GO:0051010	1	16	0.023792137	0.070243452	FBXW11
regulation of postsynapse organization	GO:0099175	1	16	0.023792137	0.070243452	ABHD17A
protein localization to membrane	GO:0072657	1	16	0.023792137	0.070243452	ABHD17A
plasma membrane raft	GO:0044853	1	17	0.025174342	0.073162932	PRTN3
cellular senescence	GO:0090398	1	17	0.025174342	0.073162932	NSMCE2
regulation of small GTPase mediated signal transduction	GO:0051056	1	18	0.026554638	0.075407065	RALGAPA1
membrane protein ectodomain proteolysis	GO:0006509	1	18	0.026554638	0.075407065	PRTN3
negative regulation of NIK/NF-kappaB signaling	GO:1901223	1	18	0.026554638	0.075407065	FBXW11
mitochondrial proton-transporting ATP synthase complex	GO:0005753	1	19	0.027933028	0.076405046	ATP5F1D
regulation of multicellular organism growth	GO:0040014	1	19	0.027933028	0.076405046	GAMT
protein sumoylation	GO:0016925	1	19	0.027933028	0.076405046	NSMCE2
metallocarboxypeptidase activity	GO:0004181	1	19	0.027933028	0.076405046	AGTPBP1
bHLH transcription factor binding	GO:0043425	1	19	0.027933028	0.076405046	TCF3
postsynaptic density membrane	GO:0098839	1	20	0.029309513	0.076782667	ABHD17A
brain morphogenesis	GO:0048854	1	20	0.029309513	0.076782667	FBXW11
triglyceride homeostasis	GO:0070328	1	20	0.029309513	0.076782667	MED13
positive regulation of protein localization to nucleus	GO:1900182	1	20	0.029309513	0.076782667	STK11
positive regulation of smooth muscle cell proliferation	GO:0048661	1	20	0.029309513	0.076782667	ELANE
axon extension	GO:0048675	1	20	0.029309513	0.076782667	NDN
establishment of cell polarity	GO:0030010	1	21	0.030684096	0.077649549	STK11
mRNA 3'-UTR AU-rich region binding	GO:0035925	1	21	0.030684096	0.077649549	MEX3D
protein kinase activator activity	GO:0030295	1	21	0.030684096	0.077649549	STK11
ephrin receptor binding	GO:0046875	1	22	0.03205678	0.080034377	EFNA2
positive regulation of axonogenesis	GO:0050772	1	22	0.03205678	0.080034377	STK11
spermatogenesis	GO:0007283	2	198	0.032596023	0.080838136	DAZAP1|GAMT
NADH dehydrogenase (ubiquinone) activity	GO:0008137	1	23	0.033427568	0.08127487	NDUFS7
gamma-tubulin binding	GO:0043015	1	23	0.033427568	0.08127487	NDN
regulation of lipid metabolic process	GO:0019216	1	23	0.033427568	0.08127487	GOLM1

germ cell development	GO:0007281	1	24	0.034796461	0.083511507	FBXW11
positive regulation of transforming growth factor beta receptor	GO:0030511	1	24	0.034796461	0.083511507	STK11
osteoclast differentiation	GO:0030316	1	25	0.036163463	0.086235951	EFNA2
DNA binding	GO:0003677	3	517	0.037040595	0.087764977	BRIP1|NFIX|PLAG1
peroxisomal membrane	GO:0005778	1	26	0.037528577	0.087802708	PEX5L
protein monoubiquitination	GO:0006513	1	26	0.037528577	0.087802708	UBE4B
establishment of mitotic spindle orientation	GO:0000132	1	27	0.038891803	0.089861807	FBXW11
nucleotide-excision repair	GO:0006289	1	27	0.038891803	0.089861807	BRIP1
positive regulation of GTPase activity	GO:0043547	2	222	0.040054608	0.091866077	RALGAPA1|PRTN3
embryonic skeletal system development	GO:0048706	1	28	0.040253147	0.091866077	SP3
T cell differentiation	GO:0030217	1	29	0.041612608	0.093817517	SP3
cytokine binding	GO:0019955	1	29	0.041612608	0.093817517	ELANE
negative regulation of cell population proliferation	GO:0008285	2	228	0.042011745	0.094146802	AGTPBP1|NDN
methylation	GO:0032259	1	30	0.042970191	0.094585273	GAMT
regulation of cell growth	GO:0001558	1	30	0.042970191	0.094585273	CLSTN1
kinesin binding	GO:0019894	1	30	0.042970191	0.094585273	CLSTN1
response to ionizing radiation	GO:0010212	1	31	0.044325898	0.09521648	STK11
chromosome, telomeric region	GO:0000781	1	31	0.044325898	0.09521648	NSMCE2
adult walking behavior	GO:0007628	1	31	0.044325898	0.09521648	AGTPBP1
regulation of transcription by RNA polymerase II	GO:0006357	3	557	0.044536741	0.09521648	BRIP1|MED13|KLF16
polysome	GO:0005844	1	32	0.045679731	0.097102057	NAA35
nucleoplasm	GO:0005654	6	1867	0.046697801	0.098290954	DAZAP1|TCF3|NDN|CIRBP|STK11|BRIP1
sensory perception of pain	GO:0019233	1	33	0.047031693	0.098290954	NDN
ephrin receptor signaling pathway	GO:0048013	1	33	0.047031693	0.098290954	EFNA2
DNA helicase activity	GO:0003678	1	34	0.048381787	0.099989026	BRIP1
negative regulation of cold-induced thermogenesis	GO:0120163	1	34	0.048381787	0.099989026	STK11
protein heterodimerization activity	GO:0046982	2	250	0.049486666	0.10109052	PDSS2|TCF3
retina development in camera-type eye	GO:0060041	1	35	0.049730014	0.10109052	AGTPBP1
erythrocyte differentiation	GO:0030218	1	35	0.049730014	0.10109052	JAK2
liver development	GO:0001889	1	36	0.051076377	0.103263111	SP3
DNA-binding transcription factor activity	GO:0003700	2	258	0.052316573	0.103726422	PLAG1|TCF3
FAD binding	GO:0071949	1	37	0.05242088	0.103726422	MICAL2
tubulin binding	GO:0015631	1	37	0.05242088	0.103726422	AGTPBP1
4 iron, 4 sulfur cluster binding	GO:0051539	1	37	0.05242088	0.103726422	NDUFS7
positive regulation of autophagy	GO:0010508	1	38	0.053763524	0.105820269	STK11

amyloid-beta binding	GO:0001540	1	39	0.055104312	0.106956837	CLSTN1
SCF-dependent proteasomal ubiquitin-dependent protein catabolic process	GO:0031146	1	39	0.055104312	0.106956837	FBXW11
positive regulation of transcription, DNA-templated	GO:0045893	2	266	0.055203529	0.106956837	FBXW11|MED16
catalytic activity	GO:0003824	1	41	0.057780329	0.111369339	MED16
protein destabilization	GO:0031648	1	42	0.059115563	0.112774306	FBXW11
RNA polymerase II transcription regulator complex	GO:0090575	1	43	0.060448952	0.113000051	TCF3
repressing transcription factor binding	GO:0070491	1	43	0.060448952	0.113000051	TCF3
cytosolic small ribosomal subunit	GO:0022627	1	43	0.060448952	0.113000051	RPS15
E-box binding	GO:0070888	1	44	0.061780497	0.113213521	TCF3
heart looping	GO:0001947	1	44	0.061780497	0.113213521	MICAL2
activation of protein kinase activity	GO:0032147	1	44	0.061780497	0.113213521	STK11
mitochondrial respiratory chain complex I	GO:0005747	1	44	0.061780497	0.113213521	NDUFS7
nuclear chromatin	GO:0000790	2	285	0.062279544	0.11356858	KLF16|TCF3
mitochondrial respiratory chain complex I assembly	GO:0032981	1	45	0.063110201	0.113965993	NDUFS7
ossification	GO:0001503	1	45	0.063110201	0.113965993	SP3
spliceosomal complex	GO:0005681	1	46	0.064438066	0.115801741	CIRBP
DNA replication	GO:0006260	1	47	0.065764095	0.116496396	NFIX
electron transport chain	GO:0022900	1	47	0.065764095	0.116496396	UQCR11
positive regulation of synapse assembly	GO:0051965	1	48	0.06708829	0.117720961	CLSTN1
negative regulation of DNA-binding transcription factor activity	GO:0043433	1	49	0.068410654	0.118919454	MED13
cytoplasmic microtubule	GO:0005881	1	49	0.068410654	0.118919454	FBXW11
mitochondrion organization	GO:0007005	1	51	0.071049898	0.122363714	AGTPBP1
mRNA 3'-UTR binding	GO:0003730	1	51	0.071049898	0.122363714	DAZAP1
axonogenesis	GO:0007409	1	52	0.072366783	0.122365652	STK11
DNA duplex unwinding	GO:0032508	1	52	0.072366783	0.122365652	BRIP1
negative regulation of translation	GO:0017148	1	52	0.072366783	0.122365652	CIRBP
SCF ubiquitin ligase complex	GO:0019005	1	53	0.073681847	0.123991886	FBXW11
cell cycle arrest	GO:0007050	1	54	0.074995092	0.123991886	STK11
cytoplasmic stress granule	GO:0010494	1	54	0.074995092	0.123991886	CIRBP
lung development	GO:0030324	1	54	0.074995092	0.123991886	SP3
p53 binding	GO:0002039	1	54	0.074995092	0.123991886	STK11
positive regulation of neuron differentiation	GO:0045666	1	55	0.076306521	0.125048572	TCF3
canonical Wnt signaling pathway	GO:0060070	1	55	0.076306521	0.125048572	STK11
cell surface	GO:0009986	2	325	0.078108806	0.12709829	ELANE|CLSTN1
B cell differentiation	GO:0030183	1	57	0.078923938	0.12709829	SP3

secretory granule	GO:0030141	1	57	0.078923938	0.12709829	ELANE
ubiquitin-dependent ERAD pathway	GO:0030433	1	57	0.078923938	0.12709829	UBE4B
protein autoubiquitination	GO:0051865	1	58	0.080229932	0.12754502	UBE4B
double-strand break repair via homologous recombination	GO:0000724	1	58	0.080229932	0.12754502	NSMCE2
growth cone	GO:0030426	1	58	0.080229932	0.12754502	CBARP
animal organ morphogenesis	GO:0009887	1	59	0.081534119	0.128519883	GAMT
central nervous system development	GO:0007417	1	59	0.081534119	0.128519883	NDN
T cell receptor signaling pathway	GO:0050852	1	61	0.084137083	0.131508383	STK11
cytoskeleton organization	GO:0007010	1	61	0.084137083	0.131508383	MICAL2
response to lipopolysaccharide	GO:0032496	1	62	0.085435865	0.132979673	ELANE
post-embryonic development	GO:0009791	1	63	0.08673285	0.133658902	NDN
kinetochore	GO:0000776	1	64	0.088028041	0.133658902	FBXW11
PML body	GO:0016605	1	64	0.088028041	0.133658902	NSMCE2
positive regulation of DNA-binding transcription factor activity	GO:0051091	1	64	0.088028041	0.133658902	TCF3
ubiquitin ligase complex	GO:0000151	1	64	0.088028041	0.133658902	UBE4B
spermatid development	GO:0007286	1	64	0.088028041	0.133658902	BRIP1
cholesterol homeostasis	GO:0042632	1	65	0.089321439	0.135071444	MED13
regulation of transcription, DNA-templated	GO:0006355	2	360	0.092882195	0.139786358	PLAG1|RALGAPA1
cell projection	GO:0042995	1	68	0.093190905	0.139786358	NDN
protease binding	GO:0002020	1	69	0.094477159	0.141146599	ELANE
small GTPase mediated signal transduction	GO:0007264	1	76	0.103431231	0.151623811	DOCK3
DNA-binding transcription activator activity, RNA polymerase II-specific	GO:0001228	2	385	0.103896804	0.151623811	PLAG1|NDN
regulation of apoptotic process	GO:0042981	1	77	0.104703312	0.151623811	JAK2
Notch signaling pathway	GO:0007219	1	77	0.104703312	0.151623811	CFD
guanyl-nucleotide exchange factor activity	GO:0005085	1	77	0.104703312	0.151623811	DOCK3
DNA-binding transcription factor activity	GO:0000981	2	389	0.105691842	0.151623811	NFIX|KLF16
ATP binding	GO:0005524	4	1275	0.105747359	0.151623811	UBE4B|STK11|JAK2|BRIP1
endosome membrane	GO:0010008	1	78	0.105973631	0.151623811	ABHD17A
dendritic spine	GO:0043197	1	78	0.105973631	0.151623811	ABHD17A
response to bacterium	GO:0009617	1	79	0.107242193	0.152850942	CFD
multicellular organism growth	GO:0035264	1	80	0.108508998	0.153480408	PLAG1
neuron projection development	GO:0031175	1	82	0.11103735	0.155871298	UBE4B
hormone activity	GO:0005179	1	82	0.11103735	0.155871298	INSL6
transcription coregulator activity	GO:0003712	1	83	0.1122989	0.156461389	MED13
postsynaptic membrane	GO:0045211	1	85	0.114816765	0.158780061	CLSTN1

neuron migration	GO:0001764	1	88	0.118580502	0.162774711	NDN
proton transmembrane transport	GO:1902600	1	90	0.121080982	0.164282485	ATP5F1D
glucose homeostasis	GO:0042593	1	91	0.122328625	0.164282485	STK11
nuclear envelope	GO:0005635	1	91	0.122328625	0.164282485	FBXW11
cell population proliferation	GO:0008283	1	91	0.122328625	0.164282485	DAZAP1
transcription regulatory region sequence-specific DNA binding	GO:0000976	1	97	0.129778279	0.17221612	KLF16
negative regulation of canonical Wnt signaling pathway	GO:0090090	1	98	0.131013876	0.17221612	STK11
homophilic cell adhesion via plasma membrane adhesion molecules	GO:0007156	1	98	0.131013876	0.17221612	CLSTN1
negative regulation of gene expression	GO:0010629	1	99	0.132247761	0.17261813	PLAG1
ribonucleoprotein complex	GO:1990904	1	99	0.132247761	0.17261813	DAZAP1
extracellular region	GO:0005576	2	454	0.135964744	0.176849247	INSL6|CLSTN1
cytokine-mediated signaling pathway	GO:0019221	1	104	0.138391593	0.178137275	JAK2
heparin binding	GO:0008201	1	104	0.138391593	0.178137275	ELANE
ubiquitin-protein transferase activity	GO:0004842	1	105	0.139615256	0.179092674	NSMCE2
oxidoreductase activity	GO:0016491	1	109	0.144492978	0.184712673	MICAL2
cell cycle	GO:0007049	1	111	0.146921712	0.187174236	NSMCE2
heart development	GO:0007507	1	113	0.149343718	0.187322875	MICAL2
protein autophosphorylation	GO:0046777	1	113	0.149343718	0.187322875	STK11
membrane	GO:0016020	2	482	0.149556167	0.187322875	STK11|JAK2
axon guidance	GO:0007411	1	115	0.151759015	0.188659295	EFNA2
extracellular space	GO:0005615	3	952	0.152144593	0.188659295	ELANE|CFD|PRTN3
histone binding	GO:0042393	1	119	0.156569554	0.192224007	JAK2
postsynaptic density	GO:0014069	1	124	0.162545311	0.198251986	CLSTN1
protein dimerization activity	GO:0046983	1	127	0.166110917	0.201938762	FBXW11
translation	GO:0006412	1	128	0.167296158	0.202717168	RPS15
nucleolus	GO:0005730	2	551	0.184113862	0.220936634	MIDN|AGTPBP1
cellular response to DNA damage stimulus	GO:0006974	1	143	0.184878775	0.221141171	STK11
cell division	GO:0051301	1	144	0.186037983	0.221814518	NSMCE2
nuclear membrane	GO:0031965	1	147	0.189505966	0.225227538	BRIP1
transcription corepressor activity	GO:0003714	1	148	0.190658753	0.225875975	ELANE
receptor complex	GO:0043235	1	155	0.198683596	0.232294989	PEX5L
structural constituent of ribosome	GO:0003735	1	156	0.199823646	0.232294989	RPS15
neuronal cell body	GO:0043025	1	156	0.199823646	0.232294989	FBXW11
actin binding	GO:0003779	1	158	0.202099002	0.234208189	MICAL2
transcription regulator complex	GO:0005667	1	168	0.213381429	0.243490465	TCF3

signaling receptor binding	GO:0005102	1	171	0.2167357	0.24606898	PRTN3
cytoplasm	GO:0005737	6	2867	0.216964046	0.24606898	UBE4B|GAMT|TCF3|INTS2|BRIP1|RALGAPA1
cytoskeleton	GO:0005856	1	190	0.237657662	0.262340208	JAK2
magnesium ion binding	GO:0000287	1	190	0.237657662	0.262340208	STK11
GTPase activator activity	GO:0005096	1	194	0.241992295	0.266334715	RALGAPA1
zinc ion binding	GO:0008270	2	664	0.242788664	0.266422958	AGTPBP1|NSMCE2
neuron projection	GO:0043005	1	202	0.250589546	0.272571085	FBXW11
ubiquitin protein ligase activity	GO:0061630	1	211	0.260147761	0.281322579	FBXW11
protein serine/threonine kinase activity	GO:0004674	1	214	0.263307323	0.283913983	STK11
glutamatergic synapse	GO:0098978	1	215	0.264357582	0.284222603	CLSTN1
mitochondrial inner membrane	GO:0005743	1	231	0.280964396	0.300341251	UQCR11
nucleic acid binding	GO:0003676	1	235	0.285058609	0.302113398	R3HDM4
mitochondrion	GO:0005739	2	774	0.300759602	0.316947796	STK11|AGTPBP1
negative regulation of apoptotic process	GO:0043066	1	256	0.306182887	0.321751509	NAA35
negative regulation of transcription, DNA-templated	GO:0045892	1	265	0.315048595	0.33013543	FBXW11
intracellular signal transduction	GO:0035556	1	267	0.317003691	0.331251048	JAK2
nuclear speck	GO:0016607	1	299	0.347554688	0.361146212	PLAG1
protein ubiquitination	GO:0016567	1	311	0.3586641	0.371651936	NSMCE2
chromatin binding	GO:0003682	1	349	0.392640072	0.405728074	SP3
perinuclear region of cytoplasm	GO:0048471	1	411	0.444340098	0.456614686	MEX3D
RNA binding	GO:0003723	1	422	0.453052896	0.464285612	RPS15
signal transduction	GO:0007165	1	432	0.460857863	0.470986607	INSL6
metal ion binding	GO:0046872	2	1164	0.495425606	0.504926919	MICAL2|NDUFS7
protein homodimerization activity	GO:0042803	1	514	0.520889439	0.529384329	TCF3
oxidation-reduction process	GO:0055114	1	516	0.522268949	0.529384329	MICAL2
calcium ion binding	GO:0005509	1	560	0.551656221	0.556141231	CLSTN1
Golgi apparatus	GO:0005794	1	639	0.600056267	0.603299814	GOLM1
plasma membrane	GO:0005886	2	2339	0.854646139	0.856949767	ABHD17A|EFNA2
integral component of membrane	GO:0016021	3	3697	0.909415243	0.909415243	UQCR11|CBARP|CLSTN1
