# Supplementary material for: Identification of genomic diversity and selection signatures in Luxi cattle using whole-genome sequencing data
Source: Anim Biosci. 2024 Jan 20;37(3):461–70. doi: 10.5713/ab.23.0304 (PMC10915192; doi:10.5713/ab.23.0304)
Supplement: Supplementary file 10 [file ab-23-0304-Supplementary-Table-S10.pdf]

Supplementary Table S10. QTLs Overlapped with Candidate Selected Regions.

Chromosome	QTL traits	QTL start site(bp)	QTL end site (bp)	QTL information
1	Meat_and_Carcass_QTL	24549201	94402650	QTL_ID=12199
1	Reproduction_QTL	50246903	102544700	QTL_ID=3439
1	Milk_QTL	60296283	144690571	QTL_ID=21533
1	Health_QTL	67679502	112799170	QTL_ID=9947
1	Exterior_QTL	78446695	102247320	QTL_ID=7124
1	Reproduction_QTL	78446695	125412168	QTL_ID=5658
1	Health_QTL	78449771	101673070	QTL_ID=1736
1	Meat_and_Carcass_QTL	79656722	89336942	QTL_ID=10643
1	Milk_QTL	79656722	125412168	QTL_ID=2508
1	Production_QTL	85829913	93623311	QTL_ID=5266
2	Milk_Association	0	136230987	QTL_ID=9943
2	Exterior_Association	0	136230987	QTL_ID=9944
2	Milk_QTL	4078468	40208237	QTL_ID=1675
2	Meat_and_Carcass_QTL	6872895	104870360	QTL_ID=12152
2	Meat_and_Carcass_QTL	12597135	24443308	QTL_ID=1312
2	Production_QTL	12597135	32502936	QTL_ID=1549
2	Exterior_QTL	12597135	32502936	QTL_ID=1550
2	Milk_QTL	12597135	32502936	QTL_ID=2651
2	Production_QTL	12599250	27468313	QTL_ID=2613
2	Production_QTL	12599250	27468313	QTL_ID=2615
2	Production_QTL	16394257	26548119	QTL_ID=5379
2	Meat_and_Carcass_QTL	19175992	27468313	QTL_ID=4857
2	Production_QTL	20854552	27468313	QTL_ID=1304
2	Meat_and_Carcass_QTL	21725034	24443308	QTL_ID=10657
2	Meat_and_Carcass_QTL	22211574	63461640	QTL_ID=1322
5	Milk_QTL	4693754	92900908	QTL_ID=10437
5	Milk_QTL	6110736	90863996	QTL_ID=10435
5	Milk_QTL	7439157	93609399	QTL_ID=10436
5	Health_QTL	11185304	103882522	QTL_ID=9915
5	Milk_QTL	17889402	109284767	QTL_ID=21534
5	Production_QTL	26568420	66421050	QTL_ID=37668
5	Exterior_QTL	28561051	61718439	QTL_ID=10278
5	Milk_QTL	28561051	61718439	QTL_ID=10279
5	Milk_QTL	30199437	95911996	QTL_ID=10438
5	Milk_QTL	31882104	117786662	QTL_ID=9993
5	Meat_and_Carcass_QTL	36310174	66421050	QTL_ID=1362
5	Production_QTL	37195788	60221752	QTL_ID=4901
5	Production_QTL	40301636	63551660	QTL_ID=1301
5	Production_QTL	40301636	63551660	QTL_ID=1305
5	Reproduction_QTL	41623858	72620348	QTL_ID=1376
5	Health_QTL	46131633	70973105	QTL_ID=2659
5	Meat_and_Carcass_QTL	46406173	80449175	QTL_ID=1365
5	Milk_QTL	46406173	80449175	QTL_ID=4495
5	Meat_and_Carcass_QTL	46937542	62878594	QTL_ID=4904
5	Milk_QTL	47578726	62648334	QTL_ID=2429
5	Production_QTL	47823156	113358592	QTL_ID=3422
5	Health_QTL	49594384	63551660	QTL_ID=4973
5	Health_QTL	49860068	77491225	QTL_ID=5117
5	Meat_and_Carcass_QTL	54908068	70849120	QTL_ID=4903
5	Meat_and_Carcass_QTL	54908068	79705260	QTL_ID=1363

5	Meat_and_Carcass_QTL	56679296	61992980	QTL_ID=1364
5	Milk_QTL	57564910	66421050	QTL_ID=9997
5	Production_QTL	57715464	58919899	QTL_ID=1295
5	Reproduction_QTL	57715464	65712558	QTL_ID=10732
5	Meat_and_Carcass_QTL	57715464	65712558	QTL_ID=10731
5	Production_Association	58627646	63551660	QTL_ID=3406
5	Meat_and_Carcass_QTL	58627646	63551660	QTL_ID=3543
5	Reproduction_QTL	58627646	65712558	QTL_ID=1758
5	Reproduction_QTL	58627646	65712558	QTL_ID=1761
7	Milk_QTL	0	93365497	QTL_ID=21539
7	Reproduction_QTL	0	13682170	QTL_ID=2699
7	Reproduction_QTL	0	13682170	QTL_ID=2700
7	Milk_QTL	0	13682170	QTL_ID=3533
7	Milk_QTL	0	13682170	QTL_ID=3535
7	Reproduction_QTL	0	13682170	QTL_ID=10782
7	Reproduction_QTL	4002545	27119287	QTL_ID=5386
7	Health_QTL	4574337	94754136	QTL_ID=9916
7	Production_QTL	6869674	13682170	QTL_ID=10783
7	Production_QTL	6869674	26171745	QTL_ID=10786
7	Production_QTL	7923406	46805275	QTL_ID=5274
7	Production_QTL	9557098	39617031	QTL_ID=5275
7	Exterior_QTL	11435844	98021520	QTL_ID=3449
7	Health_QTL	12412818	12912814	QTL_ID=263363
7	Reproduction_QTL	20421150	57996066	QTL_ID=9927
7	Exterior_QTL	20739719	69693300	QTL_ID=7132
7	Reproduction_QTL	32126553	47826333	QTL_ID=3538
7	Meat_and_Carcass_QTL	35941224	57996066	QTL_ID=1327
7	Production_QTL	39126923	47826333	QTL_ID=10793
7	Production_QTL	39126923	47826333	QTL_ID=10792
7	Production_QTL	39126923	47826333	QTL_ID=10791
7	Meat_and_Carcass_QTL	39126923	47826333	QTL_ID=10790
7	Production_QTL	42067569	46805275	QTL_ID=5277
7	Milk_QTL	42475992	80867754	QTL_ID=3434
7	Production_QTL	43521554	46110956	QTL_ID=4350
7	Health_Association	43666502	43666506	QTL_ID=57601
7	Production_QTL	43684924	43848293	QTL_ID=4544
7	Milk_Association	43684924	43848293	QTL_ID=6185
7	Reproduction_Association	43807450	43970820	QTL_ID=10092
8	Meat_and_Carcass_QTL	9692980	41415460	QTL_ID=2548
8	Reproduction_QTL	24012155	47804015	QTL_ID=11443
8	Milk_QTL	24673040	51108440	QTL_ID=3438
8	Production_QTL	25140065	55540775	QTL_ID=7089
8	Production_QTL	36657088	44155929	QTL_ID=10827
8	Meat_and_Carcass_QTL	36657088	58184315	QTL_ID=10826
8	Production_QTL	36657088	58184315	QTL_ID=10825
8	Milk_Association	39359625	39359629	QTL_ID=215577
8	Reproduction_QTL	44155929	81703009	QTL_ID=11442
8	Exterior_QTL	62616650	104596066	QTL_ID=3599
8	Reproduction_QTL	62616650	104596066	QTL_ID=4657
8	Reproduction_QTL	62616650	104596066	QTL_ID=4658
8	Reproduction_QTL	74001496	81703009	QTL_ID=10833
8	Meat_and_Carcass_QTL	74001496	104596066	QTL_ID=10834

8	Milk_Association	79023037	79023041	QTL_ID=33791
9	Milk_QTL	11580914	70439207	QTL_ID=3747
9	Milk_QTL	19801092	94009774	QTL_ID=21540
9	Meat_and_Carcass_QTL	21917447	45097442	QTL_ID=4507
9	Exterior_QTL	28084852	45406266	QTL_ID=7135
9	Health_QTL	28084852	45406266	QTL_ID=66184
9	Exterior_QTL	30728025	74499340	QTL_ID=10292
9	Reproduction_QTL	30728025	74499340	QTL_ID=10293
9	Exterior_QTL	36332280	50865192	QTL_ID=3441
9	Milk_QTL	38584881	45406266	QTL_ID=1687
9	Exterior_QTL	38584881	45406266	QTL_ID=1688
9	Milk_QTL	38584881	45406266	QTL_ID=1689
9	Reproduction_QTL	38584881	51846163	QTL_ID=10854
9	Milk_QTL	39792929	45406266	QTL_ID=3754
9	Milk_QTL	39792929	70439207	QTL_ID=3745
9	Health_QTL	39792929	74081518	QTL_ID=10085
9	Health_QTL	41536879	82637770	QTL_ID=10076
9	Meat_and_Carcass_QTL	41782122	69031332	QTL_ID=4906
14	Meat_and_Carcass_QTL	0	17448178	QTL_ID=1332
14	Meat_and_Carcass_QTL	0	19034376	QTL_ID=1333
14	Milk_QTL	0	19827475	QTL_ID=3407
14	Milk_QTL	0	26418127	QTL_ID=3618
14	Production_QTL	0	26418127	QTL_ID=10954
14	Milk_QTL	0	33310158	QTL_ID=3408
14	Milk_QTL	0	55516930	QTL_ID=3413
14	Milk_QTL	0	82402986	QTL_ID=10099
14	Milk_QTL	0	82402986	QTL_ID=10100
14	Milk_QTL	0	82402986	QTL_ID=10101
14	Health_QTL	4044804	74154756	QTL_ID=9917
14	Milk_QTL	4064632	26418127	QTL_ID=3620
14	Meat_and_Carcass_QTL	4064632	34602909	QTL_ID=4833
14	Health_QTL	4064632	34602909	QTL_ID=66189
14	Reproduction_QTL	4837903	38703231	QTL_ID=5374
14	Production_QTL	4837903	67651344	QTL_ID=5375
14	Milk_QTL	8327539	48125247	QTL_ID=2604
14	Health_QTL	8327539	48125247	QTL_ID=3177
14	Meat_and_Carcass_QTL	8724089	15861980	QTL_ID=1370
14	Milk_QTL	11111316	20382644	QTL_ID=2675
14	Reproduction_QTL	11111316	26418127	QTL_ID=10958
14	Production_QTL	11111316	33072228	QTL_ID=2627
14	Production_QTL	14148886	33072228	QTL_ID=1734
14	Meat_and_Carcass_QTL	14148886	34602909	QTL_ID=10960
14	Meat_and_Carcass_QTL	14156817	16885077	QTL_ID=1408
14	Milk_Association	14961324	14961328	QTL_ID=248215
14	Milk_QTL	20382644	41534594	QTL_ID=2732
14	Milk_QTL	20382644	41534594	QTL_ID=2733
14	Health_QTL	20382644	41534594	QTL_ID=2734
14	Health_QTL	20382644	41534594	QTL_ID=2776
14	Meat_and_Carcass_QTL	23325171	24301941	QTL_ID=222287
14	Meat_and_Carcass_QTL	23330541	23375751	QTL_ID=223755
14	Health_Association	23338888	23338892	QTL_ID=71512
14	Exterior_Association	23338888	23338892	QTL_ID=95575

14	Exterior_Association	23338888	23338892	QTL_ID=95592
14	Exterior_Association	23338888	23338892	QTL_ID=95593
14	Production_Association	23338888	23338892	QTL_ID=102021
14	Production_Association	23338888	23338892	QTL_ID=102022
14	Production_Association	23338888	23338892	QTL_ID=102023
14	Production_Association	23338888	23338892	QTL_ID=102031
14	Production_Association	23338888	23338892	QTL_ID=102032
14	Production_Association	23338888	23338892	QTL_ID=102033
14	Meat_and_Carcass_Association	23338888	23338892	QTL_ID=125067
14	Meat_and_Carcass_Association	23338888	23338892	QTL_ID=125071
14	Meat_and_Carcass_Association	23338888	23338892	QTL_ID=125081
14	Exterior_Association	23338888	23338892	QTL_ID=154178
14	Exterior_Association	23338888	23338892	QTL_ID=154312
14	Exterior_Association	23338888	23338892	QTL_ID=154313
14	Exterior_Association	23338888	23338892	QTL_ID=154314
14	Milk_Association	23338888	23338892	QTL_ID=161530
14	Milk_Association	23338888	23338892	QTL_ID=161531
14	Milk_Association	23338888	23338892	QTL_ID=161532
14	Milk_Association	23338888	23338892	QTL_ID=161533
14	Milk_Association	23338888	23338892	QTL_ID=161534
14	Production_Association	23338888	23338892	QTL_ID=161535
14	Production_Association	23338888	23338892	QTL_ID=161536
14	Production_Association	23338888	23338892	QTL_ID=161537
14	Reproduction_Association	23338888	23338892	QTL_ID=169796
14	Production_Association	23338888	23338892	QTL_ID=172041
14	Production_Association	23338888	23338892	QTL_ID=180498
14	Production_Association	23338888	23338892	QTL_ID=182249
14	Production_Association	23338888	23338892	QTL_ID=184269
14	Production_Association	23338888	23338892	QTL_ID=186948
14	Meat_and_Carcass_Association	23338888	23338892	QTL_ID=190494
14	Meat_and_Carcass_Association	23338888	23338892	QTL_ID=191918
14	Exterior_Association	23338888	23338892	QTL_ID=214876
14	Production_Association	23343148	23343152	QTL_ID=20871
14	Production_Association	23343148	23343152	QTL_ID=20872
14	Production_Association	23343148	23343152	QTL_ID=20873
14	Meat_and_Carcass_Association	23343148	23343152	QTL_ID=36553
14	Production_Association	23343148	23343152	QTL_ID=185277
14	Production_Association	23343148	23343152	QTL_ID=188452
14	Meat_and_Carcass_Association	23343148	23343152	QTL_ID=190946
14	Production_Association	23346063	23346067	QTL_ID=185383
14	Production_Association	23346063	23346067	QTL_ID=188608
14	Meat_and_Carcass_Association	23346063	23346067	QTL_ID=190987
14	Production_Association	23354420	23354424	QTL_ID=184873
14	Production_Association	23354420	23354424	QTL_ID=187837
14	Meat_and_Carcass_Association	23354420	23354424	QTL_ID=190809
14	Production_Association	23354567	23354571	QTL_ID=185144
14	Production_Association	23354567	23354571	QTL_ID=188255
14	Meat_and_Carcass_Association	23354567	23354571	QTL_ID=190903
15	Exterior_QTL	12367579	42062169	QTL_ID=1699
15	Meat_and_Carcass_QTL	16273131	53468859	QTL_ID=1335
15	Milk_QTL	27896796	47037097	QTL_ID=21547
15	Meat_and_Carcass_QTL	29415621	42069918	QTL_ID=10995

15	Meat_and_Carcass_QTL	29415621	42069918	QTL_ID=10994
15	Production_QTL	29415621	42069918	QTL_ID=10993
15	Meat_and_Carcass_QTL	30275772	57056696	QTL_ID=12195
15	Exterior_QTL	37363108	42062169	QTL_ID=1596
15	Exterior_QTL	37363108	42062169	QTL_ID=1598
15	Exterior_QTL	37363108	42062169	QTL_ID=1601
15	Health_QTL	37363108	42062169	QTL_ID=2678
16	Meat_and_Carcass_QTL	21443600	47175920	QTL_ID=1353
16	Meat_and_Carcass_QTL	27447808	48891408	QTL_ID=1355
16	Meat_and_Carcass_QTL	29780871	46403950	QTL_ID=11022
16	Health_QTL	33023144	56525329	QTL_ID=5123
16	Meat_and_Carcass_QTL	33057453	46378218	QTL_ID=4837
16	Meat_and_Carcass_QTL	37509145	46403950	QTL_ID=11024
16	Production_QTL	37509145	55924908	QTL_ID=11025
19	Meat_and_Carcass_QTL	0	21420040	QTL_ID=1339
19	Milk_QTL	1389408	52681720	QTL_ID=10444
19	Milk_QTL	2373572	59397192	QTL_ID=10445
19	Health_QTL	2373572	61307628	QTL_ID=10446
19	Milk_QTL	6368120	53839560	QTL_ID=10027
19	Milk_QTL	8162772	34966768	QTL_ID=10443
19	Meat_and_Carcass_QTL	9262720	44576840	QTL_ID=10021
19	Reproduction_QTL	9285876	26584006	QTL_ID=4668
19	Meat_and_Carcass_QTL	9288192	22577880	QTL_ID=1396
19	Meat_and_Carcass_QTL	9288192	22577880	QTL_ID=1397
19	Meat_and_Carcass_QTL	9288192	22577880	QTL_ID=1398
19	Meat_and_Carcass_QTL	9288192	22577880	QTL_ID=1399
19	Health_QTL	10362668	22577880	QTL_ID=3623
19	Meat_and_Carcass_QTL	10877906	25073025	QTL_ID=11077
19	Production_QTL	10877906	25073025	QTL_ID=11075
19	Production_QTL	10877906	25073025	QTL_ID=11076
19	Production_QTL	10970534	11086318	QTL_ID=4452
19	Reproduction_Association	11102150	11102154	QTL_ID=146102
20	Meat_and_Carcass_QTL	0	7148862	QTL_ID=11094
20	Milk_QTL	0	17499005	QTL_ID=2750
20	Milk_QTL	0	27647821	QTL_ID=2564
20	Meat_and_Carcass_QTL	0	27647821	QTL_ID=12250
20	Health_QTL	0	27647821	QTL_ID=66202
20	Health_QTL	0	27647821	QTL_ID=66204
20	Health_QTL	0	27647821	QTL_ID=66205
20	Health_QTL	0	27647821	QTL_ID=66206
20	Health_QTL	0	27647821	QTL_ID=66208
20	Health_QTL	0	27647821	QTL_ID=66209
20	Health_QTL	0	27647821	QTL_ID=66210
20	Milk_QTL	0	71974585	QTL_ID=10103
20	Health_QTL	546708	10795320	QTL_ID=4561
20	Production_QTL	546708	16612990	QTL_ID=1357
20	Health_QTL	1735582	30372685	QTL_ID=9948
21	Meat_and_Carcass_QTL	0	9139379	QTL_ID=11114
21	Reproduction_QTL	0	10506951	QTL_ID=1721
21	Reproduction_QTL	0	31529191	QTL_ID=11389
21	Production_QTL	250165	9506289	QTL_ID=5300
21	Health_QTL	29928132	52267911	QTL_ID=5446

21	Milk_QTL	33438788	48949049	QTL_ID=10165
21	Production_QTL	34781343	52267911	QTL_ID=1358
21	Reproduction_QTL	35957121	51067117	QTL_ID=11126
21	Health_QTL	43470425	57888296	QTL_ID=66212
22	Meat_and_Carcass_QTL	37284662	57206606	QTL_ID=1315
22	Exterior_QTL	44203537	57206606	QTL_ID=1628
22	Milk_QTL	44203537	57206606	QTL_ID=2686
22	Reproduction_QTL	44203537	57206606	QTL_ID=4671
22	Production_QTL	44203537	57206606	QTL_ID=11151
22	Milk_QTL	44838170	55281324	QTL_ID=1536
22	Milk_QTL	44838170	55281324	QTL_ID=1537
22	Meat_and_Carcass_QTL	48797725	58889762	QTL_ID=11152
22	Production_QTL	48797725	60772965	QTL_ID=11155
